# Supplementary material for: Chemo-, regio- and stereoselective access to polysubstituted 1,3-dienes via Nickel-catalyzed four-component reactions
Source: Nat Commun. 2024 Jun 28;15:5479. doi: 10.1038/s41467-024-49870-1 (PMC11213876; doi:10.1038/s41467-024-49870-1)
Supplement: Supplementary file 1 — Supplementary Information [file 41467_2024_49870_MOESM1_ESM.pdf]

Supplementary information

for

Chemo-, regio- and stereoselective access to polysubstituted 1,3-dienes via Nickel-catalyzed four-component reactions

Shanglin Chen[1†], Ya-Nan Wang[2†], Jinhui Xie[1], Wangyang Li[1], Mingxing Ye[1], Xingxing Ma[1], Kai Yang[1], Shijun Li[3], Yu Lan[2,3*] and Qiuling Song[1,4*]

[1]Key Laboratory of Molecule Synthesis and Function Discovery, Fujian Province University, College of Chemistry at Fuzhou University, Fuzhou, Fujian, 350108, China

[2]Chongqing Key Laboratory of Theoretical and Computational Chemistry, School of Chemistry and Chemical Engineering, Chongqing University, Chongqing, 401331, China

[3]College of Chemistry and Institute of Green Catalysis, Zhengzhou University, Zhengzhou, 450001, China

[4]School of Chemistry and Chemical Engineering, Henan Normal University, Xinxiang, Henan, 453007, China

*Email: qsong@fzu.edu.cn; ylan@zzu.edu.cn

Table of Contents

Supplementary methods 3
1. General information 3
2. Optimization of reaction conditions 4
    2.1 The detailed analysis of the byproducts and the isomeric products 4
    2.2 Optimization of reaction conditions 7
3. Preparation of substrates 19
    3.1 Procedure for preparation of terminal alkenes 20
    3.2 Procedure for preparation of ester substituted terminal alkenes 22
    3.3 Procedure for preparation of terminal alkenes 24
4. General procedure for the synthesis of polysubstituted 1,3-dienes 24
    4.1 Procedure for preparation of polysubstituted 1,3-dienes 24
    4.2 Scale up synthesis 25
5. The characterization of polysubstituted 1,3-dienes 25
6. Synthesis and characterization of applications 57
7. Mechanism experiments 64
    7.1 Controlled experiments 64
    7.2 Radical inhibition reactions 65
    7.3 The influence of two ligands on the reaction was explored 66
    7.4 Two different alkynes were involved in the reaction 67
    7.5 Unsuccessful examples 69
8. Density Functional Theory (DFT) calculations 70
    8.1 Computational details 70
    8.2 All possible pathways for nickel-catalyzed cascade difunctionalization reaction 70
    8.3 A comparison of the energy barriers of the transition states for these two alkyne insertion pathways 71
    8.4 The directly transmetallation process in the absence of base. 71
9. X-ray crystallographic data 72
10. NMR spectrum of the new compounds 75
11. Supplementary references 185

Supplementary methods

1. General information

All operations were performed under an argon atmosphere unless otherwise specified. Flash column chromatography was performed over silica gel (200-300 mesh). ^{1}H NMR, ^{13}C NMR and ^{19}F NMR spectrum were recorded at ambient temperature using Bruker Ascend™ 400 (400 MHz) spectrometer or JNM-ECZ500R/S1 (500 MHz) spectrometer. ^{1}H NMR chemical shifts (in ppm) were referenced to $CDCl_3$ (δ = 7.26 ppm) as internal standards. ^{13}C NMR spectrum were obtained by using the same NMR spectrometers and were calibrated with $CDCl_3$ (δ = 77.16 ppm). The following abbreviations are used: s = singlet, d = doublet, t = triplet, q = quartet, dd, = double doublet, dt = double triplet, m = multiplet. GCMS data were obtained on SHIMADZU GCMS-QP2020 NX with EI mode. HRMS data were obtained on Thermo Scientific Orbitrap Elite Mass Spectrometer with an ESI source (Ion Trap) or Agilent 7250 QTOF with EI mode. GC analysis was performed at Shimadzu GC-2030. Analytical thin-layer chromatography (TLC) was carried out on Merck 60 F254 pre-coated silica gel plate (0.2 mm thickness). Visualization was accomplished by UV light (254 nm), also by Gas Chromatograph Mass spectrometer analysis (GCMS). Unless otherwise noted, materials obtained from commercial suppliers were used without further purification.

2. Optimization of reaction conditions

2.1 The detailed analysis of the byproducts and the isomeric products

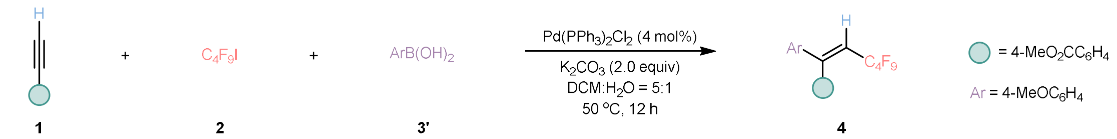

Supplementary Fig 1. Synthesis of compound 4.

To an oven-dried 10 mL Young's Tube vial equipped with a magnetic stir bar was added $Pd(PPh_3)_2Cl_2$ (0.04 mmol, 5.6 mg , 4 mol%), terminal alkynes (0.2 mmol, 32 mg, 1.0 equiv), 4-Methoxyphenylboronic acid (0.26 mmol, 39.5 mg, 1.3 equiv), K_2CO_3(0.4 mmol, 56 mg, 2.0 equiv), The vial was introduced in a argon-filled atmosphere, then perflfluoroalkyl iodides (0.4 mmol, 2.0 equiv), DCM and H_2O (v/v, 5:1) were added. Next, the reaction mixture was stirred at 50 °C in an oil bath at 660 rpm for 12 h. After the reaction was completed, the reaction mixture was extracted with DCM and the combined organic layers were dried over Na_2SO_4, filtered and concentrated under reduced pressure. The product was purified by column chromatography over silica gel.

methyl (*E*)-4-(3,3,4,4,5,5,6,6,6-nonafluoro-1-(4-methoxyphenyl)hex-1-en-1-yl)benzoate (4)

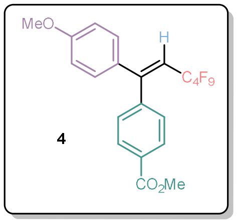

Compounds **4** was prepared according to the literature[1], **4** (68 mg, 0.14 mmol) was obtained as yellow liquid in 70% yield.

R_f= 0.5 (silica gel, petroleum ether/ethyl acetate = 12:1).

^{1}H NMR: (500 MHz, Chloroform-*d*) δ 8.07 – 8.05 (m, 2H), 7.31 – 7.29 (m, 2H), 7.16 – 7.13 (m, 2H), 6.86 – 6.83 (m, 2H), 6.06 (t, J = 14.7 Hz, 1H), 3.94 (s, 3H), 3.81 (s, 3H).

^{19}F NMR: (471 MHz, Chloroform-*d*) δ -80.9 (t, J = 10.1 Hz, 3F), -103.2 – -103.4 (m, 2F), -123.7 – -123.9 (m, 2F), -125.5 – -125.7 (m, 2F).

^{13}C NMR: (126 MHz, Chloroform-*d*) δ 166.9, 161.1, 152.8 (t, J = 4.4 Hz), 142.6, 132.2, 130.1, 129.4, 129.3, 129.2 (t, J = 2.9 Hz), 114.2, 111.3 (t, J = 21.3 Hz), 55.5, 52.3.

HRMS (EI): calc'd for $C_{21}H_{15}F_9O_3$ Exact Mass: 486.0877, found 486.0879.

In our current catalytic system, the yield of **4** was determined by gas chromatography (GC) using *n*-

dodecane as the internal standard.

According to the previous literature[2-3], we guess that the migratory insertion of alkyne with organometallic species with syn-selectivity would afford the corresponding two products (namely **5** and **5'**) shown below.

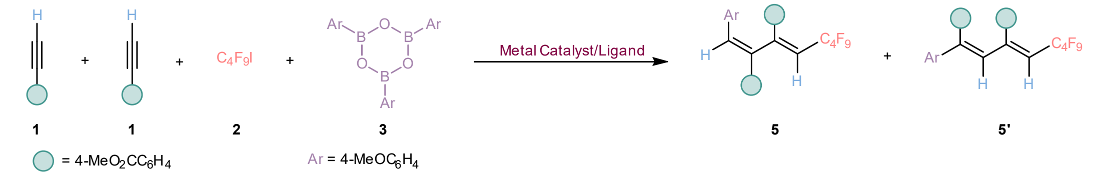

Supplementary Fig 2. Products 5 and 5' in our current reaction system

Supplementary Fig 3. The corresponding two products 5 and 5' were detected by GCMS

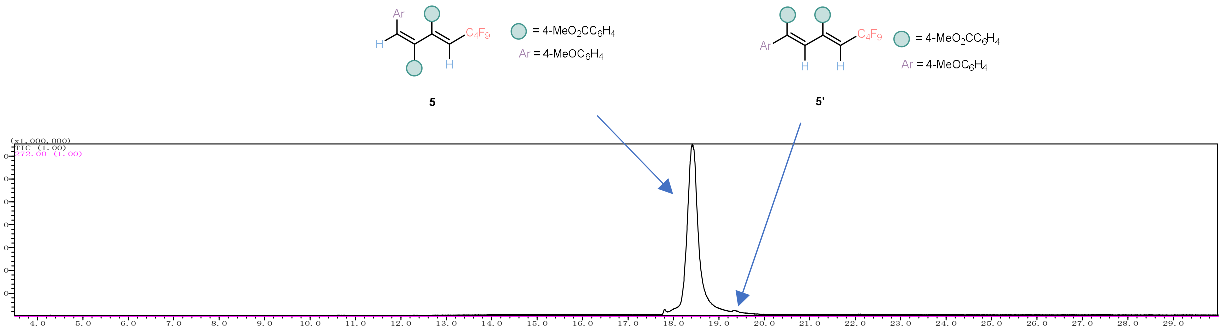

The peak time of **5** is 18.41 min.

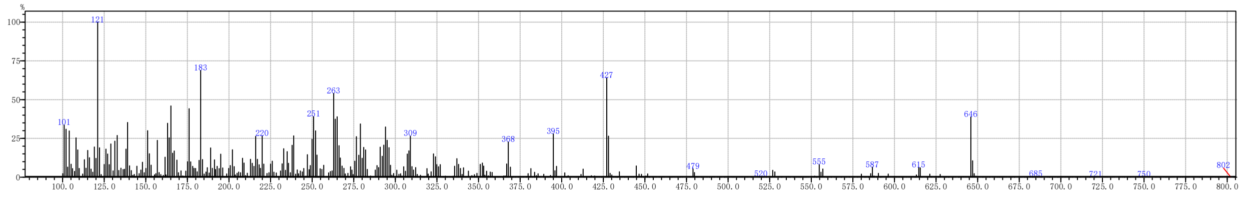

The peak time **5'** is 19.33 min

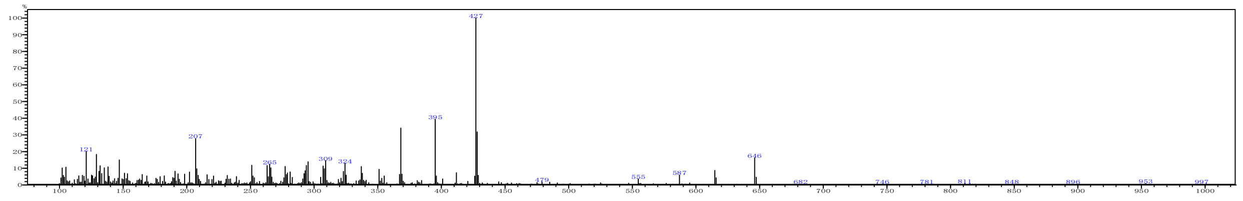

Supplementary Fig 4. The ratio of 5 and 5' were determined by GC

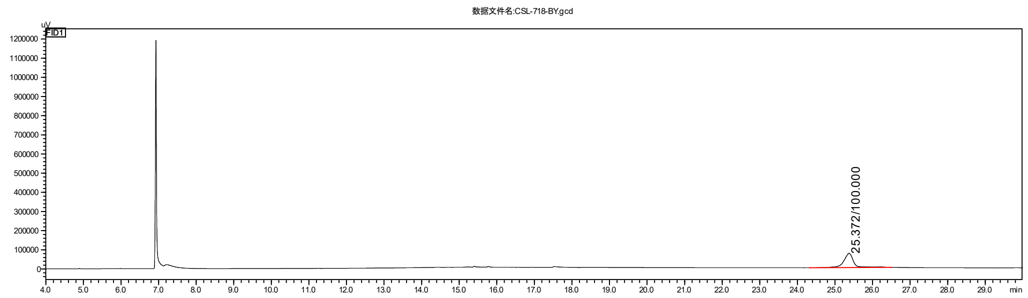

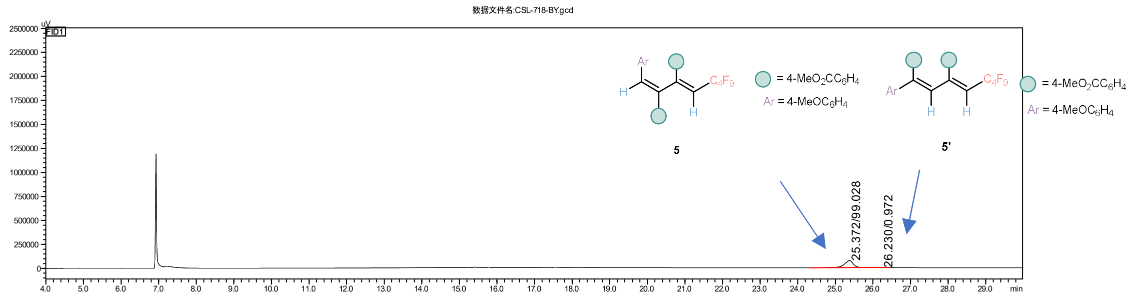

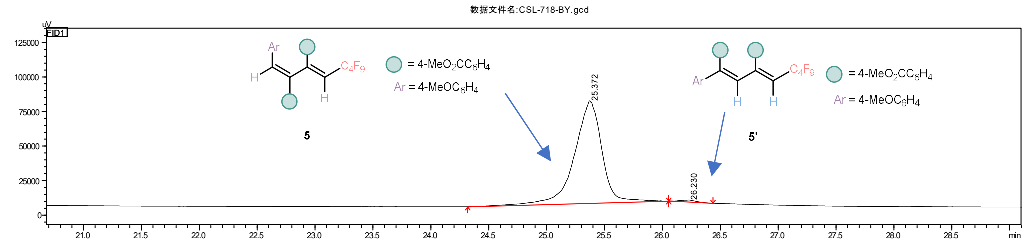

	retention time	area	height	K'	area ratio
1	25.372	1208864	73945	0.000	98.799
2	26.230	14691	1580	0.034	1.201

The ^{1}HNMR spectra data of the product is a set of peaks, which indicates that the reaction has good regioselectivity (namely the ratio of **5**:**5'** was greater than 95:5), Please see the spectra data of **5**, which is on page 81 of this Supplementary information.

All of the regioselectivity of polysubstituted 1,3-dienes obtained by our current strategy are determined by GC or ^{1}H NMR similarly. At the same time, the origins of good regioselectivity without the use of directing group-containing or electronically biased alkynes were elaborately elucidated by subsequent calculation (Please see the Supplementary Fig 28 for more details).

2.2 Optimization of reaction conditions

Supplementary Table 1. Pd source evaluation [a]

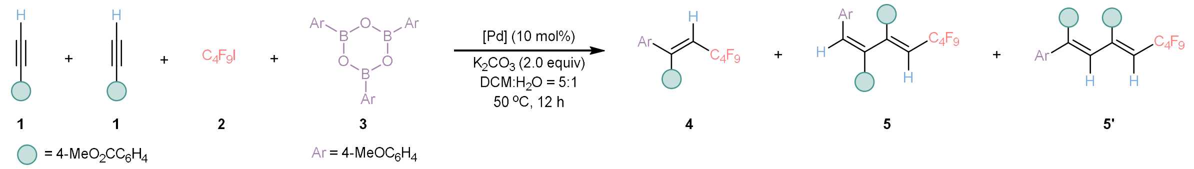

entry	Pd Catalyst	yield (%)[b]
1	$Pd(dba)_2$	**4** 7% + **5** 26%
2	$Pd(cod)Cl_2$	N.D.
3	$Pd(PPh_3)_4$	**4** 72% + **5** 29%
4	$Pd(PPh_3)_4$	**4** 61% + **5** 29%[c]
5	$Pd(PPh_3)_4$	**4** 7% + **5** 6%[d]
6	$Pd(CN)_2Cl_2$	**4** 6% + **5** 21%
7	$Pd(dppf)Cl_2$	**4** 37% + **5** 32%
8	$Pd(dppe)Cl_2$	**4** 2% + **5** 5%
9	$Pd(dppp)Cl_2$	**4** 25% + **5** 16%
10	$Pd(PPh_3)_2Cl_2$	**4** 71% + **5** 20%
11	$Pd(t\text{-}Bu_3P)_2$	**4** 2% + **5** N.D.
12	$PdBr_2$	**4** 55% + **5** 30%[e]
13	$PdCl_2$	**4** 6% + **5** 23%[e]
14	$Pd(OAc)_2$	**4** 5% + **5** 17%[e]

[a] Reaction conditions: the reaction was carried out with **1** (0.25 mmol), **2** (0.8 mmol), **3** (0.067 mmol), Pd catalyst (4 mol%), K_2CO_3 (0.4 mmol, 2.0 equiv) in 2 mL of mixed solvent (DCM:H_2O = 5:1) at 50 °C for 12 h. [b]Yields were determined by gas chromatography (GC) using *n*-dodecane as the internal standard. N.D.: No Detected. [c]The reaction was carried out with **1** (0.25 mmol), **2** (0.8 mmol), **3** (0.067 mmol), Pd catalyst (4 mol%), Cs_2CO_3 (0.2 mmol, 1.0 equiv) in 2 mL 1,4-dioxane at 80 °C for 12 h. [d]The reaction was carried out with **1** (0.25 mmol), **2** (0.8 mmol), **3** (0.067 mmol), Pd catalyst (4 mol%), K_3PO_4 (0.4 mmol, 2.0 equiv) in 2 mL toluene at 80 °C for 12 h. [e]The reaction was carried out with **1** (0.25 mmol), **2** (0.8 mmol), **3** (0.067 mmol), Pd catalyst (4 mol%), PPh_3 (5 mol%), K_2CO_3 (0.4 mmol, 2.0 equiv) in 2 mL of mixed solvent (DCM:H_2O = 5:1) at 50 °C for 12 h. Unless otherwise stated, the desired polysubstituted 1,3-dienes were obtained with regioselectivity greater than 95:5 (the ratio of **5**:**5'** was greater than 95:5), and the regioselectivity was detected by ^1H NMR analysis of desired products or GC analysis of desired products. N.D.: No Detected.

Supplementary Table 2. Fe source evaluation [a]

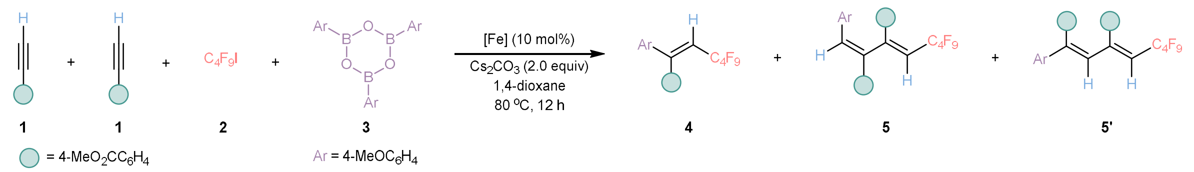

entry	Fe Catalyst	yield (%)[b]
1	$FeBr_2$	N.D.
2	$FeCl_2$	N.D.
3	$Fe(acac)_3$	N.D.
4	$Fe(OTf)_2$	N.D.
5	$Fe(OAc)_2$	N.D.

[a]Reaction conditions: the reaction was carried out with **1** (0.25 mmol), **2** (0.8 mmol), **3** (0.067 mmol), Fe catalyst (10 mol%), Cs_2CO_3 (0.4 mmol, 2.0 equiv) in 2 mL 1,4-dioxane at 80 °C for 12 h. [b]Yields were determined by gas chromatography (GC) using n-dodecane as the internal standard. Unless otherwise stated, the desired polysubstituted 1,3-dienes were obtained with regioselectivity greater than 95:5 (the ratio of **5**:**5**' was greater than 95:5), and the regioselectivity was detected by ^{1}H NMR analysis of desired products or GC analysis of desired products. N.D.: No Detected.

Supplementary Table 3. Cu source evaluation [a]

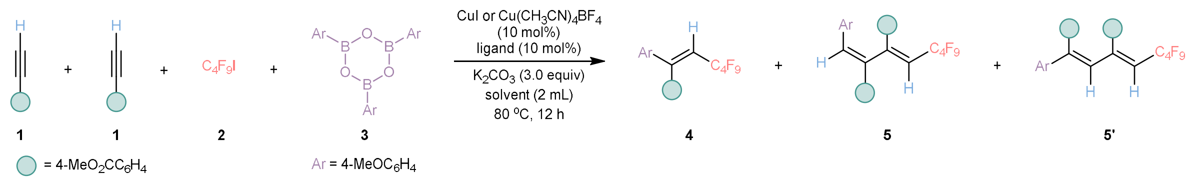

entry	ligand	yield (%)[b]
1	**L1**	N.D.[ac]
2	**L2**	N.D.[ac]
3	**L3**	N.D.[ac]
4	**L4**	N.D.[ac]
5	**L5**	N.D.[ac]
6	**L6**	N.D.[ac]

[a]Reaction conditions: the reaction was carried out with **1** (0.25 mmol), **2** (0.8 mmol), **3** (0.067 mmol), CuI (10 mol%), ligand (10 mol%), K_2CO_3 (0.6 mmol, 3.0 equiv) in 2 mL toluene at 50 °C for 12 h. [b]Yields were determined by gas chromatography (GC) using n-dodecane as the internal standard. [c]The reaction was carried out with **1** (0.25 mmol), **2** (0.8 mmol), **3** (0.067 mmol), $Cu(CH_3CN)_4BF_4$ (10 mol%), ligand (10 mol%), LPO (2.0 equiv), K_2CO_3 (0.4 mmol, 2.0 equiv) in 2 mL MeOH at 80 °C for 12 h. Unless otherwise stated, the desired polysubstituted 1,3-dienes were obtained with regioselectivity greater than 95:5 (the ratio of **5**:**5'** was greater than 95:5), and the regioselectivity was detected by ^{1}H NMR analysis of desired products or GC analysis of desired products. N.D.: No Detected.

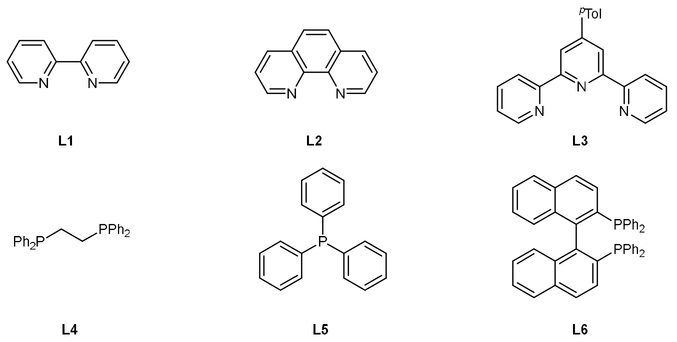

Supplementary Table 4. Ni source evaluation [a]

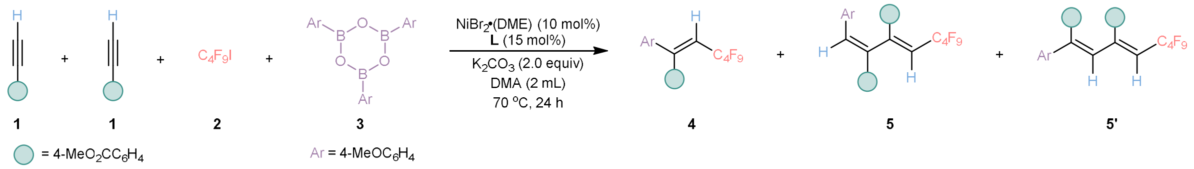

entry	ligand	yield (%)[b]
1	**L1**	**4** 5% + **5** 36%
2	**L2**	**4** 8% + **5** 3%
3	**L3**	**4** 8% + **5** 36%
4	**L4**	N.D.
5	**L5**	N.D.
6	**L6**	N.D.

[a]Reaction conditions: the reaction was carried out with **1** (0.25 mmol), **2** (0.8 mmol), **3** (0.067 mmol), Ni Catalyst (10 mol%), ligand (15 mol%), K_2CO_3 (0.4 mmol, 2.0 equiv) in 2 mL DMA at 70 °C for 24 h. [b]Yields were determined by gas chromatography (GC) using *n*-dodecane as the internal standard. Unless otherwise stated, the desired polysubstituted 1,3-dienes were obtained with regioselectivity greater than 95:5 (the ratio of **5**:**5'** was greater than 95:5), and the regioselectivity was detected by ^{1}H NMR analysis of desired products or GC analysis of desired products. N.D.: No Detected.

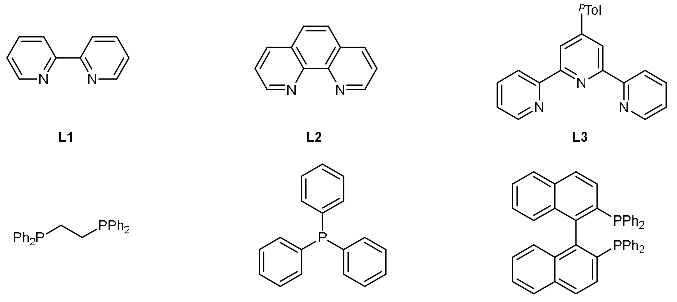

L1 L2 L3

L4 L5 L6

Supplementary Table 5. Ligand evaluation [a]

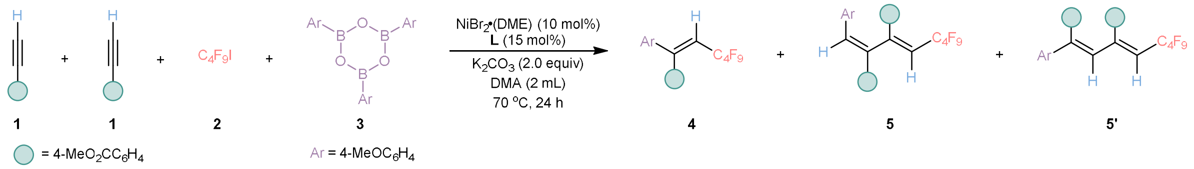

entry	ligand	**5** [%][b]
1	**L1**	36
2	**L7**	39
3	**L8**	37
4	**L9**	38
5	**L10**	Trace
6	**L11**	Trace
7	**L12**	Trace
8	**L13**	37

[a]Reaction conditions: the reaction was carried out with **1** (0.25 mmol), **2** (0.8 mmol), **3** (0.067 mmol), $NiBr_2$•(DME) (10 mol%), ligand (15 mol%), K_2CO_3 (0.4 mmol, 2.0 equiv) in 2 mL DMA at 70 °C for 24 h. [b]Yields were determined by gas chromatography (GC) using n-dodecane as the internal standard. Unless otherwise stated, the polysubstituted alkene **4** was obtained with the yield lower than 10%, the desired polysubstituted 1,3-dienes were obtained with regioselectivity greater than 95:5 (the ratio of **5**:**5'** was greater than 95:5), and the regioselectivity was detected by ^{1}H NMR analysis of desired products or GC analysis of desired products.

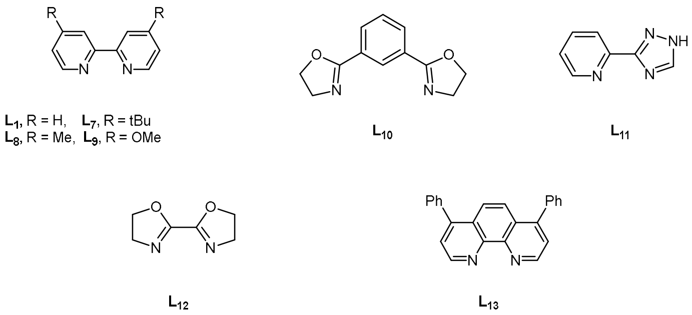

Supplementary Table 6. Ni source evaluation [a]

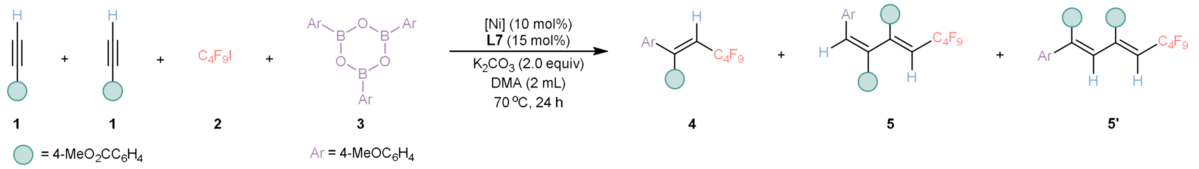

entry	nickel catalyst	**5** (%)[b]
1	$NiBr_2$•(DME)	39
2	$Ni(PCy_3)_2Cl_2$	54
3	$Ni(PCy_3)_2Br_2$	38
4	$Ni(PPh_3)Br_2$	35
5	$Ni(Py)_4Cl_2$	42
6	$Ni(acac)_2$	Trace
7	$Ni(OTf)_2$	37
8	$NiBr_2$•$6H_2O$	Trace
9	$NiBr_2$	Trace
10	$Ni(cod)_2$	9

[a]Reaction conditions: the reaction was carried out with **1** (0.25 mmol), **2** (0.8 mmol), **3** (0.067 mmol), Nickel catalyst (10 mol%), dtbpy (15 mol%), K_2CO_3 (0.4 mmol, 2.0 equiv) in 2 mL DMA at 70 °C for 24 h. [b]Yields were determined by gas chromatography (GC) using *n*-dodecane as the internal standard. Unless otherwise stated, the polysubstituted alkene **4** was obtained with the yield lower than 10%, the desired polysubstituted 1,3-dienes were obtained with regioselectivity greater than 95:5 (the ratio of **5**:**5'** was greater than 95:5), and the regioselectivity was detected by ^{1}H NMR analysis of desired products or GC analysis of desired products.

Supplementary Table 7. Solvent source evaluation [a]

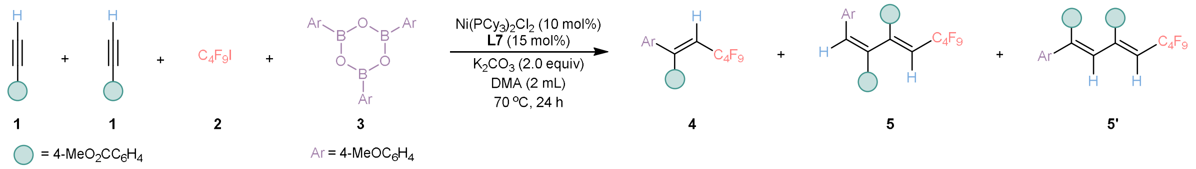

entry	Solvent	**5** (%)[b]
1	DMA	54
2	DMF	N.D.
3	NMP	42
4	1,4-dioxane	20
5	toluene	Trace
6	DME	23
7	CH_3CN	30
8	DMA:DME = 1:1, 3:1, 9:1	60, 58, 56
9	DMA:DCE = 1:1, 3:1, 9:1	48, 40, 42
10	DMA:NMP = 1:1, 3:1, 9:1	Trace, 11, 54

[a]Reaction conditions: the reaction was carried out with **1** (0.25 mmol), **2** (0.8 mmol), **3** (0.067 mmol), $Ni(PCy_3)_2Cl_2$ (10 mol%), dtbpy (15 mol%), K_2CO_3 (0.4 mmol, 2.0 equiv) in 2 mL solvent at 70 °C for 24 h. [b]Yields were determined by gas chromatography (GC) using n-dodecane as the internal standard. Unless otherwise stated, the polysubstituted alkene **4** was obtained with the yield lower than 10%, the desired polysubstituted 1,3-dienes were obtained with regioselectivity greater than 95:5 (the ratio of **5**:**5'** was greater than 95:5), and the regioselectivity was detected by ^{1}H NMR analysis of desired products or GC analysis of desired products. N.D.: No Detected.

Supplementary Table 8. Base Source evaluation [a]

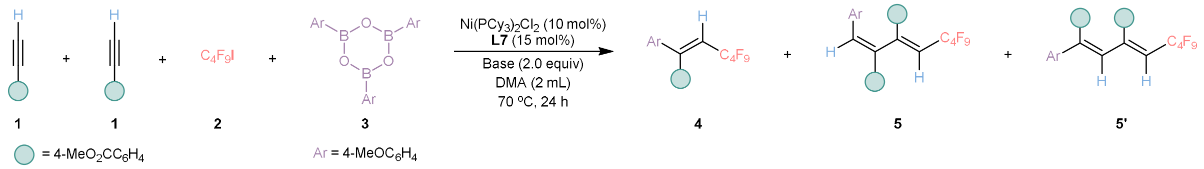

entry	base	**5** (%)[b]
1	K_2CO_3	60
2	Na_2CO_3	19
3	Li_2CO_3	Trace
4	Cs_2CO_3	N.D.
5	K_3PO_4	Trace
6	KF	Trace
7	CsF	N.D.
8	$KHCO_3$	34
9	$NaHCO_3$	47
10	KH_2PO_4	N.D.

[a]Reaction conditions: the reaction was carried out with **1** (0.25 mmol), **2** (0.8 mmol), **3** (0.067 mmol), $Ni(PCy_3)_2Cl_2$ (10 mol%), dtbpy (15 mol%), base (0.4 mmol, 2.0 equiv) in 2 mL of mixed solvent (DMA:DME = 1:1) at 70 °C for 24 h. [b]Yields were determined by gas chromatography (GC) using n-dodecane as the internal standard. Unless otherwise stated, the polysubstituted alkene **4** was obtained with the yield lower than 10%, the desired polysubstituted 1,3-dienes were obtained with regioselectivity greater than 95:5 (the ratio of **5**:**5'** was greater than 95:5), and the regioselectivity was detected by ^{1}H NMR analysis of desired products or GC analysis of desired products. N.D.: No Detected.

Supplementary Table 9. Temperature Source evaluation [a]

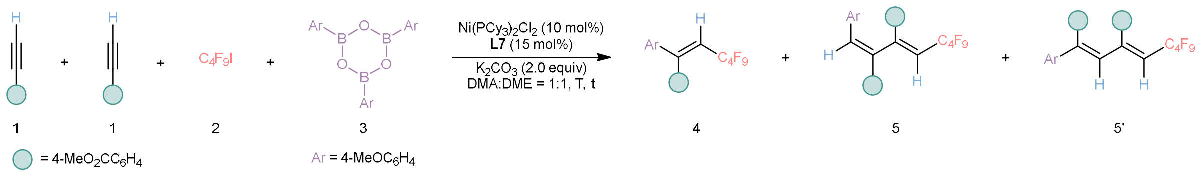

entry	temperature (°C)	**5** (%)[b]
1	70	65
2	60	67[c]
3	50	71[d]
4	45	74[d]
5	45	74[e]
6	40	65[d]
7	40	70[e]
8	30	N.D.

[a]Reaction conditions: the reaction was carried out with **1** (0.25 mmol), **2** (0.8 mmol), **3** (0.067 mmol), $Ni(PCy_3)_2Cl_2$ (10 mol%), dtbpy (15 mol%), K_2CO_3 (0.4 mmol, 2.0 equiv) in 2 mL of mixed solvent (DMA:DME = 1:1) for 24 h. [b]Yields were determined by gas chromatography (GC) using n-dodecane as the internal standard. Unless otherwise stated, the polysubstituted alkene **4** was obtained with the yield lower than 10%. [c] 48 h. [d] 72 h. [e] 96 h. Unless otherwise stated, the polysubstituted alkene **4** was obtained with the yield lower than 10%, the desired polysubstituted 1,3-dienes were obtained with regioselectivity greater than 95:5 (the ratio of **5**:**5'** was greater than 95:5), and the regioselectivity was detected by ^1H NMR analysis of desired products or GC analysis of desired products. N.D.: No Detected.

Supplementary Table 10. Reaction time evaluation [a]

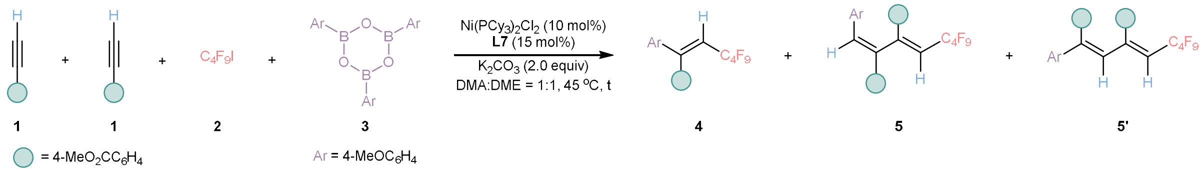

entry	time (h)	**5** (%)[b]
1	24	37
2	48	56
3	72	74
4	96	70

[a]Reaction conditions: the reaction was carried out with **1** (0.25 mmol), **2** (0.8 mmol), **3** (0.067 mmol), $Ni(PCy_3)_2Cl_2$ (10 mol%), dtbpy (15 mol%), K_2CO_3 (0.4 mmol, 2.0 equiv) in 2 mL of mixed solvent (DMA:DME = 1:1) at 45 °C. [b]Yields were determined by gas chromatography (GC) using n-dodecane as the internal standard. Unless otherwise stated, the polysubstituted alkene **4** was obtained with the yield lower than 10%, the desired polysubstituted 1,3-dienes were obtained with regioselectivity greater than 95:5 (the ratio of **5**:**5'** was greater than 95:5), and the regioselectivity was detected by ^1H NMR analysis of desired products or GC analysis of desired products.

Supplementary Table 11. Equivalent evaluation [a]

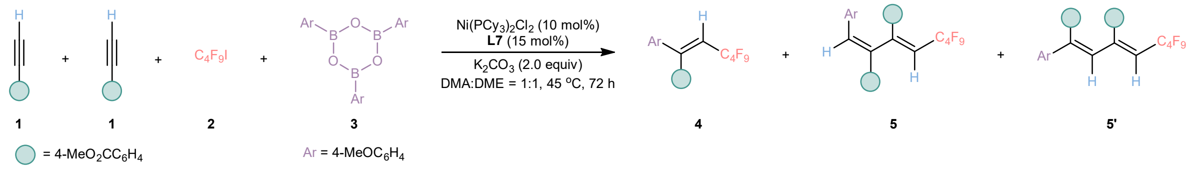

entry	**1** (x equiv)	**2** (y equiv)	**3** (z equiv)	**5** (%)[b]
1	1.25	1	1	Trace
2	1.25	2	1	34
3	1.25	3	1	64
4	1.25	4	1	74
5	1.25	5	1	67
6	1.3	4	1	72
7	1.35	4	1	76
8	1.4	4	1	73
9	1.45	4	1	70
10	1.5	4	1	72

[a]Reaction conditions: the reaction was carried out with **1** (x equiv), **2** (y equiv), **3** (0.067 mmol), $Ni(PCy_3)_2Cl_2$ (10 mol%), dtbpy (15 mol%), K_2CO_3 (0.4 mmol, 2.0 equiv) in 2 mL of mixed solvent (DMA:DME = 1:1) at 45 °C for 72 h. [b]Yields were determined by gas chromatography (GC) using n-dodecane as the internal standard. Unless otherwise stated, the polysubstituted alkene **4** was obtained with the yield lower than 10%, the desired polysubstituted 1,3-dienes were obtained with regioselectivity greater than 95:5 (the ratio of **5**:**5’** was greater than 95:5), and the regioselectivity was detected by ^{1}H NMR analysis of desired products or GC analysis of desired products.

Supplementary Table 12. Concentration evaluation [a]

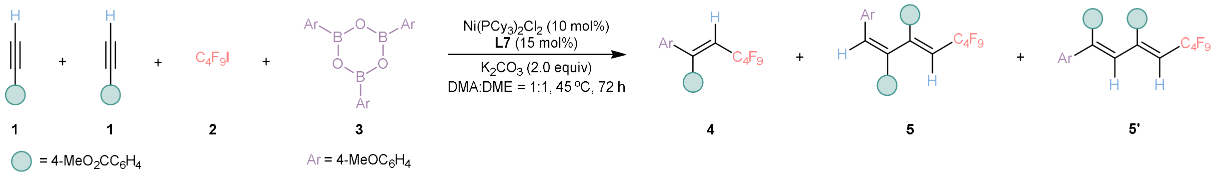

entry	concentration (M)	**5** (%)[b]
1	0.1	76
2	0.2	81 (71)[c]
3	0.3	70
4	0.4	54
5	0.5	52

[a]Reaction conditions: the reaction was carried out with **1** (0.27 mmol), **2** (0.8 mmol), **3** (0.2 mmol), $Ni(PCy_3)_2Cl_2$ (10 mol%), dtbpy (15 mol%), K_2CO_3 (0.4 mmol, 2.0 equiv) in the mixed solvent (DMA:DME = 1:1) at 45 °C for 72 h. [b]Yields were determined by gas chromatography (GC) using n-dodecane as the internal standard. Unless otherwise stated, the polysubstituted alkene **4** was obtained with the yield lower than 10%, the desired polysubstituted 1,3-dienes were obtained with regioselectivity greater than 95:5 (the ratio of **5**:**5'** was greater than 95:5), and the regioselectivity was detected by ^{1}H NMR analysis of desired products or GC analysis of desired products. [c]Isolated yield.

3. Preparation of substrates

Substrates involved in this work

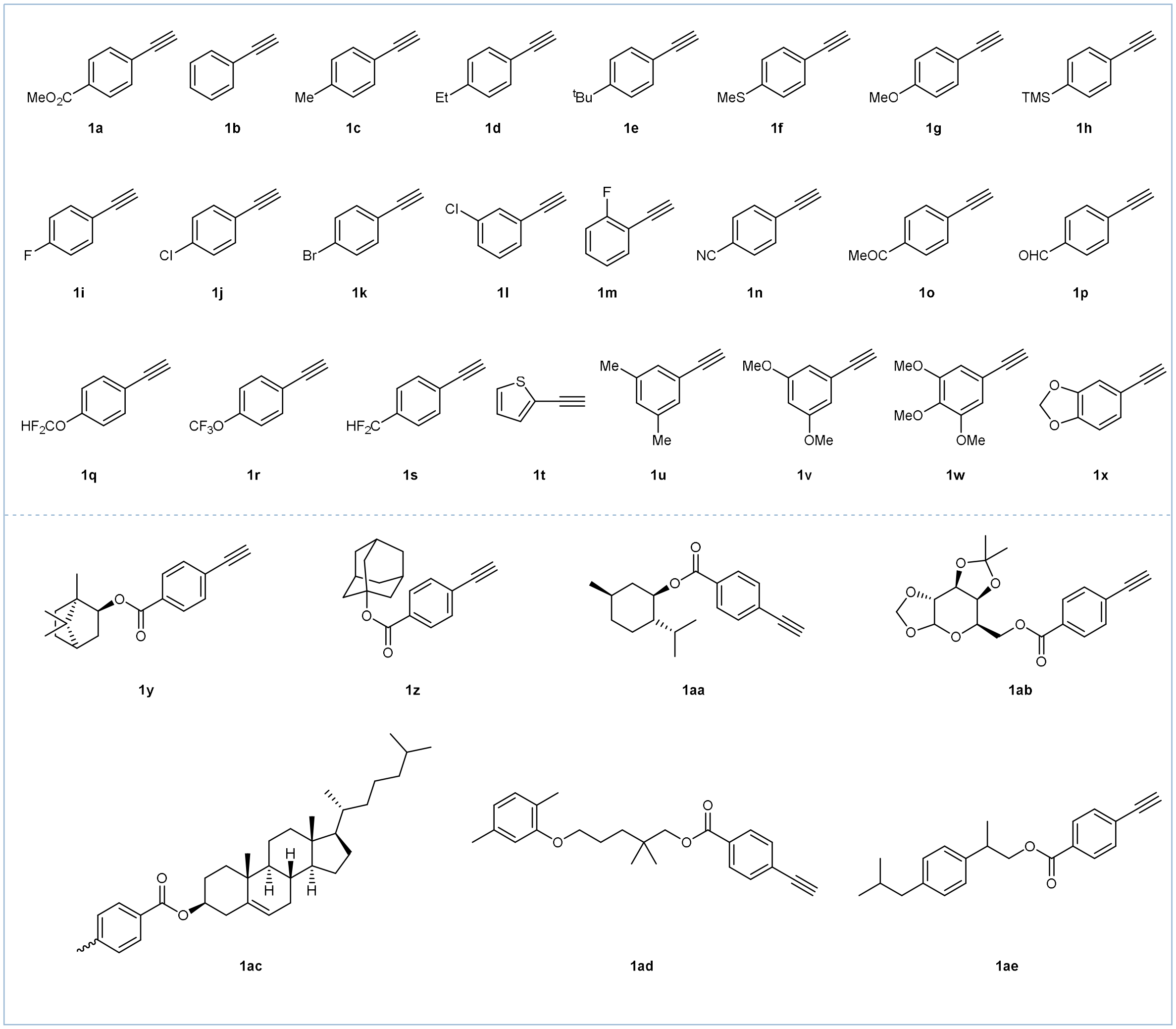

Supplementary Fig 5. Terminal alkynes were investigated in the manuscript. Terminal alkynes (**1a-1x, 1y**[4] and **1z**[4]) were known compounds. (*NOTE: The Terminal alkynes **1a-1x** were commercially available and were used without further purification.*) Compounds **1y-1z** were prepared according to the previous literatures, please see *ref.5*, **General procedure A** and **General procedure B** for more details.

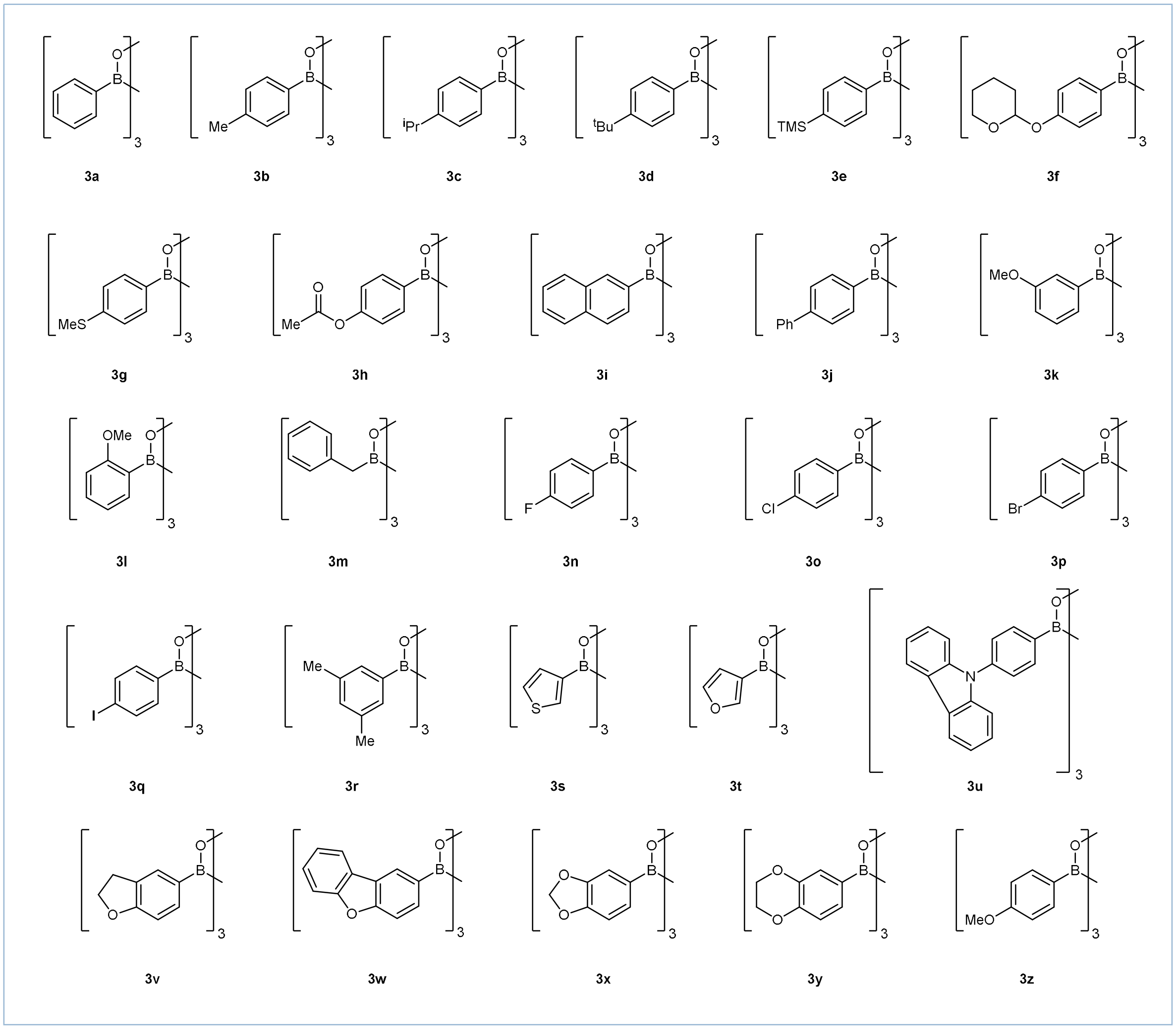

Supplementary Fig 6. Aryl boroxines were investigated in the manuscript. Aryl boroxines **3a**[6], **3b**[6], **3d**[6], **3e**[6], **3i**[6], **3k**[6], **3z**[6], **3g**[7], **3h**[7], **3j**[7], **3n**[7], **3o**[7], **3p**[7], **3q**[7], **3r**[7], **3w**[7], **3x**[7], **3c**[8], **3f**[9], **3l**[9], **3m**[10], **3t**[11], **3v**[11], **3y**[11], **3s**[12] were known compounds. New Compounds **3u** was prepared according to the literatures, please see *ref.6* and **General procedure C** for more details.

3.1 Procedure for preparation of terminal alkenes

General procedure A: Synthesis of ester substituted terminal alkynes (1y-1ae)[4,5]:

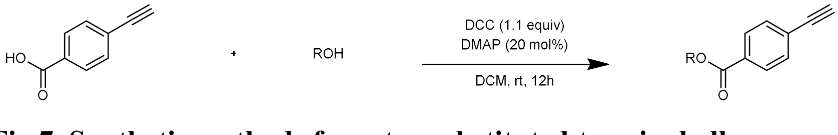

Supplementary Fig 7. Synthetic methods for ester substituted terminal alkynes

A 50 mL round bottom flask was equipped with a stirring bar (without argon atmosphere), 4-alkynobenzoic acid (5 mmol, 1.0 equiv), DCC (5.5 mmol, 1.1 equiv) and DMAP (20 mol%) were added. Then DCM (20 mL) and drug molecular alcohols (5.5mmol, 1.1 equiv) were added sequentially, the reaction mixture was stirred at rt for 12

h. After the reaction was completed, the reaction mixture was filtered through Celite pad and concentrated in vacuo. Then the crude residue was purified by flash silica gel chromatography (petroleum ether/ethyl acetate = 30:1 to 5:1) to afford the desired product.

(1*R*,2*R*,5*R*)-2-isopropyl-5-methylcyclohexyl 4-ethynylbenzoate (1aa)

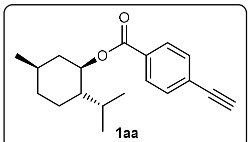

1aa

Following the general **procedure A**, 4-alkynobenzoic acid (5.0 mmol, 1.0 equiv) and L-Menthol (5.5 mmol, 1.1 equiv) were employed and stirred at rt for 12 h to afford **1aa** as a yellow oil in 78% yield (1.108 g).

$\mathbf{R}_f$ = 0.5 (silica gel, petroleum ether/ethyl acetate = 20:1).

^{1}**H NMR:** (500 MHz, Chloroform-*d*): δ 8.00 – 7.98 (m, 2H), 7.54 – 7.52 (m, 2H), 4.95 – 4.90 (m, 1H), 3.22 (s, 1H), 2.13 – 2.08 (m, 1H), 1.96 – 1.90 (m, 1H), 1.72 – 1.68 (m, 2H), 1.56 – 1.50 (m, 2H), 1.15 – 1.04 (m, 2H), 0.94 – 0.86 (m, 7H), 0.78 (d, J = 7.0 Hz, 3H).

^{13}**C NMR:** (126 MHz, Chloroform-*d*): δ 165.4, 132.1, 130.9, 129.5, 126.6, 83.0, 80.1, 77.2, 75.2, 47.3, 41.0, 34.3, 31.5, 26.6, 23.7, 22.1, 20.8, 16.6.

HRMS (ESI): calc'd for $C_{19}H_{24}NaO_2^+$ 307.1669, found 307.1665.

((3a*R*,5*R*,5a*S*,8a*S*,8b*R*)-7,7-dimethyltetrahydro-5H-bis([1,3]dioxolo)[4,5-b:4',5'-d]pyran-5-yl)methyl 4-ethynylbenzoate (1ab)

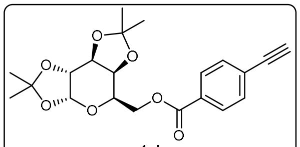

1ab

Following the general **procedure A**, 4-alkynobenzoic acid (5.0 mmol, 1.0 equiv) and 1,2,3,4-Di-O-isopropylidene-D-galactopyranose (5.5 mmol, 1.1 equiv) were employed and stirred at rt for 12 h to afford **1ab** as a white solid in 74% yield (1.436 g).

$\mathbf{R}_f$ = 0.5 (silica gel, petroleum ether/ethyl acetate = 5:1).

mp: 130 °C – 131 °C.

^{1}**H NMR:** δ 7.98 (d, J = 8.0 Hz, 2H), 7.51 (d, J = 7.8 Hz, 2H), 5.54 (d, J = 5.0 Hz, 1H), 4.63 (dd, J = 2.5, 8.0 Hz, 1H), 4.50 (dd, J = 4.7, 11.5 Hz, 1H), 4.41 (dd, J = 7.6, 11.5 Hz, 1H), 4.33 – 4.29 (m, 2H), 4.17 – 4.14 (m, 1H), 3.23 (s, 1H), 1.49 (s, 3H), 1.45 (s, 3H), 1.33 (s, 3H), 1.31 (s, 3H).

^{13}C NMR: δ 165.8, 132.1, 130.1, 129.6, 126.9, 109.7, 108.8, 96.3, 82.9, 80.3, 71.2, 70.8, 70.5, 66.15, 64.17, 26.04, 26.01, 25.0, 24.5.

HRMS (ESI): calc'd for $C_{21}H_{24}O_7^+$ 389.1595, found 389.1599.

(3*S*,8*S*,9*S*,10*R*,13*R*,14*S*,17*R*)-10,13-dimethyl-17-((*R*)-6-methylheptan-2-yl)-2,3,4,7,8,9,10,11,12,13,14,15,16,17-tetradecahydro-1H-cyclopenta[a]phenanthren-3-yl-4-ethynylbenzoate (1ac)

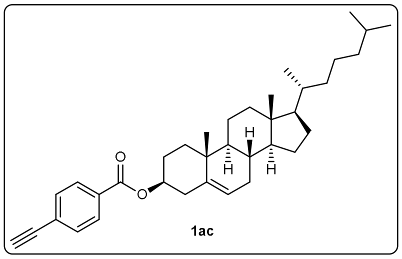

Following the general **procedure A**, 4-alkynobenzoic acid (5.0 mmol, 1.0 equiv) and drug molecular (5.5 mmol, 1.1 equiv) were employed and stirred at rt for 12 h to afford **1ac** as a white solid in 73% yield (1.876 g).

R*f* = 0.6 (silica gel, petroleum ether/ethyl acetate = 20:1).

mp: 110 – 112 °C.

^{1}H NMR: δ 8.00 – 7.98 (m, 2H), 7.54 – 7.52 (m, 2H), 5.42 – 5.40 (m, 1H), 4.88 – 4.82 (m, 1H), 3.22 (s, 1H), 2.46 (d, J = 7.4 Hz, 2H), 2.04 – 1.95 (m, 3H), 1.93 – 1.68 (m, 3H), 1.61 – 1.45 (m, 6H), 1.39 – 1.31 (m, 3H), 1.29 – 1.09 (m, 8H), 1.06 (s, 3H), 1.03 – 0.95 (m, 3H), 0.92 (d, J = 6.5 Hz, 3H), 0.88 (d, J = 2.2 Hz, 3H), 0.86 (d, J = 2.2 Hz, 3H), 0.68 (s, 3H).

^{13}C NMR: (126 MHz, Chloroform-d) δ 165.3, 139.6, 132.1, 130.9, 129.5, 126.6, 123.0, 83.0, 80.0, 75.0, 56.8, 56.3, 50.1, 42.4, 39.8, 39.6, 38.3, 37.1, 36.7, 36.3, 35.9, 32.03, 31.97, 28.4, 28.1, 28.0, 24.4, 24.0, 23.0, 22.7, 21.2, 19.5, 18.9, 12.0.

HRMS (ESI): calc'd for $C_{36}H_{51}O_2^+$ 515.3884, found 515.3882.

3.2 Procedure for preparation of ester substituted terminal alkenes

General procedure B: Synthesis of ester substituted terminal alkynes (1ad-1ae):

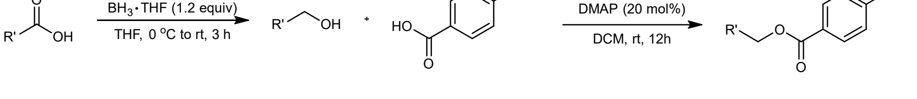

Supplementary Fig 8. Synthetic methods for ester substituted terminal alkynes originated from accessible acid.

To a 100 mL round bottom flask equipped with a stir bar was added drug molecular acid (5.5 mmol, 1.0 equiv) and THF (20 mL) (without argon atmosphere), The flask was cooled to 0°C. A 1 M solution of borane in% THF (6.6 mL, 6.6 mmol, 1.2 equiv) was added dropwise over 15 min. The reaction was warmed to rt and stirred at rt for 3 h.

After the reaction was completed, the reaction was cooled to 0 °C, and H_2O (10 mL) was added to quench the reaction. Then reaction mixture was diluted with EtOAc (25 mL*3) and the layers were separated. The organic layer was washed with saturated aqueous sodium chloride and then dried with Na_2SO_4. Next, the reaction was concentrated to give correspondingly ethanol (No purification required) as a yellow oil: R_f =0.2 (petroleum ether/ethyl acetate = 10:1). The next steps follow General procedure A to afford **1ad-1ae**.

5-(2,5-dimethylphenoxy)-2,2-dimethylpentyl 4-ethynylbenzoate (1ad)

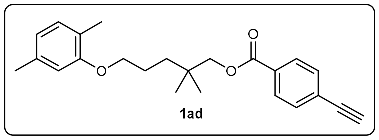

Following the general **procedure B**, 4-alkynobenzoic acid (5.0 mmol, 1.0 equiv) and drug molecular (5.5 mmol, 1.1 equiv) were employed and stirred at rt for 12 h to afford **1ad** as a white oil in 91% yield (1.656 g).

R_f = 0.4 (silica gel, petroleum ether/ethyl acetate = 20:1).

^{1}H NMR: δ 8.06 – 8.04 (m, 2H), 7.60 – 7.58 (m, 2H), 7.03 (d, J = 6.7 Hz, 1H), 6.69 (d, J = 7.6 Hz, 1H), 6.65 (s, 1H), 4.14 (s, 2H), 3.97 (t, J = 6.2 Hz, 2H), 3.28 (s, 1H), 2.34 (s, 3H), 2.20 (s, 3H), 1.89 – 1.83 (m, 2H), 1.63 – 1.59 (m, 2H), 1.10 (s, 6H).

^{13}C NMR: δ 165.9, 157.0, 136.5, 132.2, 130.5, 130.4, 129.5, 126.8, 123.5, 120.7, 111.9, 82.9, 80.2, 72.9, 68.2, 35.6, 34.0, 24.5, 24.2, 21.5, 15.8.

HRMS (ESI): calc'd for $C_{24}H_{29}O_3^+$ Exact Mass: 365.2111, found 365.2106.

2-(4-isobutylphenyl)propyl 4-ethynylbenzoate (1ae)

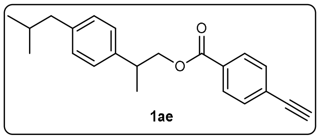

Following the general **procedure B**, 4-alkynobenzoic acid (5.0 mmol, 1.0 equiv) and drug molecular (5.5 mmol, 1.1 equiv) were employed and stirred at rt for 12 h to afford **1ae** as a white solid in 80% yield (1.280 g).

R_f = 0.5 (silica gel, petroleum ether/ethyl acetate = 20:1).

mp: 115.0 – 116.0 °C.

^{1}H NMR: δ 8.00 – 7.97 (m, 2H), 7.57 – 7.55 (m, 2H), 7.25 – 7.23 (m, 2H), 7.17 – 7.14 (m, 2H), 4.49 – 4.40 (m, 2H), 3.29 – 3.25 (m, 2H), 2.51 (d, J = 7.1 Hz, 2H), 1.94 – 1.86 (m, 1H), 1.43 (d, J = 7.0 Hz, 3H), 0.95 (d, J = 6.7 Hz, 6H).

^{13}C NMR: δ 165.7, 140.2, 140.1, 132.1, 130.4, 129.5, 129.3, 127.1, 126.7, 82.9, 80.2, 70.2, 45.1, 38.7, 30.2, 22.4, 22.4, 18.1.

HRMS (ESI): calc'd for $C_{22}H_{24}O_2Na^+$ Exact Mass: 343.1669, found 343.1667.

3.3 Procedure for preparation of terminal alkenes

General procedure C: Aryl boroxines Synthesis[4]

All aryl boroxines were synthesized from the corresponding commercially available boronic acids (which were used without further purification). The boronic acid (5 mmol) and toluene (20 mL) were refluxed in 110 °C for 3 h with the use of a Dean-Stark trap under an argon atmosphere. After removing the solvent and drying under high vacuum all aryl boroxines were directly used without further purification.

3,3'-(6-(4-(9H-carbazol-9-yl)phenyl)-1,3,5,2,4,6-trioxatriborinane-2,4-diyl)bis(9-phenyl-9H-carbazole) (3u)

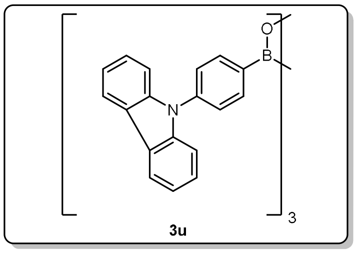

3u

Following the general **procedure C**, (4-(9H-Carbazol-9-yl) phenyl) boronic acid (5.0 mmol, 1.0 equiv) were employed and stirred at 110 °C for 3 h to afford **3u** as a faint yellow solid in 90% yield (1.210 g).

R*f* = 0.2 (silica gel, petroleum ether/ethyl acetate = 2:1).

mp: >255.0 °C.

¹H NMR: (500 MHz, DMSO-d_6): δ 8.29 – 8.21 (m, 4H), 7.68 (d, *J* = 7.8 Hz, 2H), 7.48 – 7.42 (m, 4H), 7.29 (t, *J* = 7.3 Hz, 2H).

¹³C NMR: (126 MHz, DMSO-d*6*): δ 140.1, 138.1, 136.0, 135.3, 126.3, 125.6, 122.8, 120.6, 120.1, 109.8.

HRMS (ESI): calc'd for $C_{54}H_{37}B_3N_3O_3^+$ Exact Mass: 808.3109 found 808.3113.

4. General procedure for the synthesis of polysubstituted 1,3-dienes

4.1 Procedure for preparation of polysubstituted 1,3-dienes

General procedure D: Synthesis and characterization of products

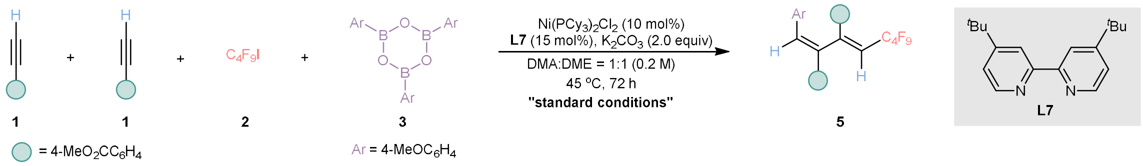

Supplementary Fig 9. Synthetic methods for polysubstituted 1,3-dienes

To an oven-dried 10 mL Young's Tube vial equipped with a magnetic stir bar was added **L7** (0.03 mmol, 8.0 mg, 15 mol%), $Ni(PCy_3)_2Cl_2$ (0.02 mmol, 13.8 mg, 10 mol%), terminal alkynes (0.54 mmol, 2.7 equiv), boroxine (0.067 mmol, 27 mg), K_2CO_3(0.4 mmol, 56 mg, 2.0 equiv), The vial was introduced in a argon-filled atmosphere,

then perflfluoroalkyl iodides (0.8 mmol, 4.0 equiv), anhydrous DMA (0.5 mL, 0.2 M) and anhydrous DME (0.5 mL, 0.2 M) were added. Next, the reaction mixture was stirred at 45 °C (or 50 °C) in an oil bath at 660 rpm for 72 h. After the reaction was completed, the reaction mixture was extracted with EtOAc and the combined organic layers were dried over Na_2SO_4, filtered and concentrated under reduced pressure. The product was purified by column chromatography over silica gel for each substrate.

4.2 Scale up synthesis

1 mmol scale-up reaction for synthesis of dimethyl 4,4'-((1*Z*,3*E*)-5,5,6,6,7,7,8,8,8-nonafluoro-1-(4-methoxyphenyl)octa-1,3-diene-2,3-diyl)dibenzoate

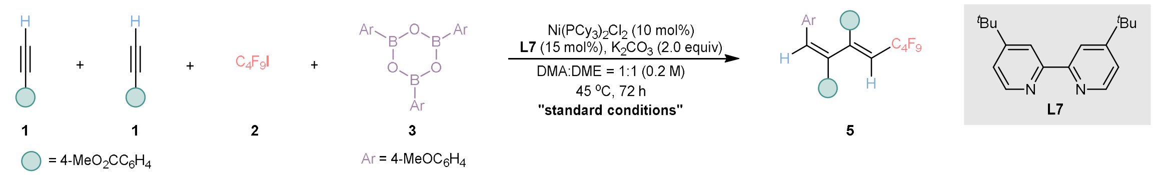

Supplementary Fig 10. Scale up reactions for polysubstituted 1,3-dienes

To an oven-dried 25 mL Young's Tube vial equipped with a magnetic stir bar was added **L7** (0.15 mmol, 42.5 mg, 15 mol%), $Ni(PCy_3)_2Cl_2$ (0.1 mmol, 69 mg, 10 mol%), Methyl 4-ethynylbenzoate (2.7 mmol, 2.7 equiv), 4-methoxyaryl boroxine (0.33 mmol, 270 mg), K_2CO_3 (2 mmol, 280 mg, 2.0 equiv), The vial was introduced in a argon-filled atmosphere, then perflfluoroalkyl iodides (4 mmol, 4.0 equiv), anhydrous DMA (2.5 mL, 0.2 M) and anhydrous DME (2.5 mL, 0.2 M) were added. Next, the reaction mixture was stirred at 45 °C in an oil bath at 660 rpm for 72 h. After the reaction was completed, the reaction mixture was extracted with EtOAc and the combined organic layers were dried over Na_2SO_4, filtered and concentrated under reduced pressure. The product was purified by column chromatography over silica gel (petroleum ether/ethyl acetate = 15:1) to afford compound **5** (420 mg, 0.65 mmol) as yellow liquid in 65% yield.

5. The characterization of polysubstituted 1,3-dienes

Dimethyl-4,4'-(*(1Z,3E)*-5,5,6,6,7,7,8,8,8-nonafluoro-1-(4-methoxyphenyl)octa-1,3-diene-2,3-diyl)dibenzoate (5)

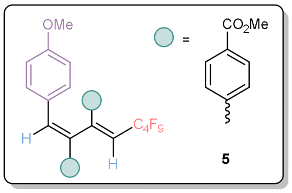

Following the general **procedure D**, **5** (91.7 mg, 0.142 mmol) was obtained as colorless liquid in 71% yield.

R_f = 0.3 (silica gel, petroleum ether/ethyl acetate = 8:1).

^{1}H NMR: (500 MHz, Chloroform-*d*) δ 7.96 – 7.94 (m, 2H), 7.91 – 7.88 (m, 2H), 7.49 – 7.42 (m, 6H), 7.00 (s, 1H), 6.87 – 6.84 (m, 2H), 6.02 (t, J = 15.2 Hz, 1H), 3.89 (s, 3H), 3.86 (s, 3H), 3.82 (s, 3H).

^{19}F NMR: (471 MHz, Chloroform-*d*) δ -80.9 (t, J = 9.7 Hz, 3F), -104.0 – -104.1 (m, 2F), -123.3 – -123.4 (m, 2F), -125.6 – -125.8 (m, 2F).

^{13}C NMR: (126 MHz, Chloroform-*d*) δ 166.7, 166.5, 159.9, 151.5 (t, J = 4.5 Hz), 144.7, 140.7, 139.5, 132.4, 130.9, 130.6, 130.0, 129.5, 129.3, 129.0 (t, J = 2.5 Hz), 128.0, 127.0, 118.7 (t, J = 20.7 Hz), 114.1, 55.4, 52.3, 52.2.

HRMS (EI): calc'd for $C_{31}H_{23}F_9O_5$ Exact Mass: 646.1402, found 646.1398.

(*(1Z,3E)*-5,5,6,6,7,7,8,8,8-nonafluoro-1-(4-methoxyphenyl)octa-1,3-diene-2,3-diyl)dibenzene (6)

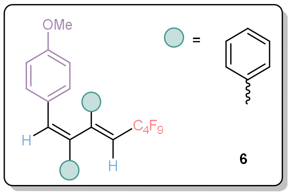

Following the general **procedure D, 6** (69 mg, 0.13 mmol) was obtained as colorless liquid in 65% yield.

R*f* = 0.2 (silica gel, petroleum ether).

^{1}H NMR: (500 MHz, Chloroform-*d*) δ 7.51 – 7.48 (m, 2H), 7.45 – 7.43 (m, 2H), 7.41 – 7.39 (m, 2H), 7.31 – 7.27 (m, 2H), 7.26 – 7.22 (m, 4H), 6.91 (s, 1H), 6.86 – 6.83 (m, 2H), 5.92 (t, J = 15.5 Hz, 1H), 3.81 (s, 3H).

^{19}F NMR: (471 MHz, Chloroform-*d*) δ -80.9 (t, J = 9.4 Hz, 3F), -103.6 – -103.9 (m, 2F), -123.4 – -123.6 (m, 2F), -125.6 – -125.7 (m, 2F).

^{13}C NMR: (126 MHz, Chloroform-*d*) δ 159.4, 152.9 (t, J = 4.3 Hz), 141.2, 140.5, 136.3, 130.7, 130.1, 129.2 (t, J = 3.0 Hz), 129.0, 128.8, 128.6, 128.0, 127.8, 127.1, 116.7 (t, J = 20.2 Hz), 114.0, 55.4.

HRMS (ESI): calc'd for $C_{27}H_{19}F_9ONa^+$ Exact Mass: 553.1184, found 553.1181.

4,4'-(*(1Z,3E)*-5,5,6,6,7,7,8,8,8-nonafluoro-1-(4-methoxyphenyl)octa-1,3-diene-2,3-diyl)bis(meth-ylbenzene) (7)

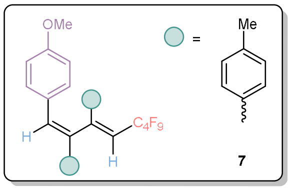

Following the general **procedure D**, **7** (68 mg, 0.122 mmol) was obtained as colorless liquid in 61% yield.

R*f* = 0.2 (silica gel, petroleum ether).

^{1}H NMR: (500 MHz, Chloroform-*d*) δ 7.49 – 7.46 (m, 2H), 7.35 (d, J = 7.9 Hz, 2H), 7.30 – 7.27 (m, 2H), 7.07 (dd, J = 7.8, 15.3 Hz, 4H), 6.88 (s, 1H), 6.84 – 6.81 (m, 2H), 5.88 (t, J = 15.7 Hz, 1H), 3.80 (s, 3H), 2.30 (s, 3H), 2.28 (s, 3H).

^{19}F NMR: (471 MHz, Chloroform-*d*) δ -80.9 (t, J = 9.3 Hz, 3F), -103.6 – -103.7 (m, 2F), -123.5 – -123.6 (m, 2F), -125.6 – -125.7 (m, 2F).

^{13}C NMR: (126 MHz, Chloroform-*d*) δ 159.3, 152.9 (t, J = 3.9 Hz), 141.1, 139.2, 137.6, 137.5, 133.3, 130.6, 129.3, 129.2 (t, J = 3.1 Hz), 129.0, 128.9, 128.8, 115.8 (t, J = 19.6 Hz), 113.9, 55.4, 21.4, 21.2.

HRMS (ESI): calc'd for $C_{29}H_{24}F_9O^+$ Exact Mass: 559.1678, found 559.1677.

4,4'-(*(1Z,3E)*-5,5,6,6,7,7,8,8,8-nonafluoro-1-(4-methoxyphenyl)octa-1,3-diene-2,3diyl)bis(ethylbenzene) (8)

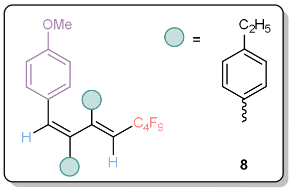

Following the general **procedure D**, **8** (70.3 mg, 0.12 mmol) was obtained as colorless liquid in 60% yield.

R*f* = 0.2 (silica gel, petroleum ether).

^{1}H NMR: (500 MHz, Chloroform-*d*) δ 7.51 – 7.48 (m, 2H), 7.41 (d, J = 8.0 Hz, 2H), 7.37 – 7.34 (m, 2H), 7.15 – 7.12 (m, 2H), 7.11 – 7.08 (m, 2H), 6.92 (s, 1H), 6.86 – 6.83 (m, 2H), 5.89 (t, J = 15.8 Hz, 1H), 3.81 (s, 3H), 2.64 – 2.58 (m, 4H), 1.24 – 1.19 (m, 6H).

^{19}F NMR: (471 MHz, Chloroform-*d*) δ -80.9 (t, J = 10.7 Hz, 3F), -103.6 (m, 2F), -123.6 (m, 2F), -125.6 (m, 2F).

^{13}C NMR: (126 MHz, Chloroform-*d*) δ 159.3, 153.0 (t, J = 4.7 Hz), 145.3, 144.0, 141.1, 137.8, 133.5, 130.7, 129.3 (t, J = 2.5 Hz), 129.0, 128.1, 127.6, 126.8, 115.7 (t, J = 20.0 Hz), 113.9, 55.4, 28.7, 28.6, 15.5, 15.1.

HRMS (EI): calc'd for $C_{31}H_{27}F_9O$ Exact Mass: 586.1918, found 586.1911.

4,4'-(*(1Z,3E)*-5,5,6,6,7,7,8,8,8-nonafluoro-1-(4-methoxyphenyl)octa-1,3-diene-2,3-diyl)bis(tert-butylbenzene) (9)

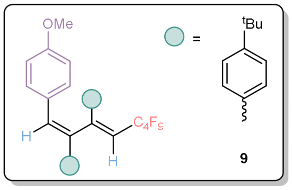

Following the general **procedure D**, **9** (77 mg, 0.12 mmol) was obtained as colorless liquid in 60% yield.

R*f* = 0.2 (silica gel, petroleum ether).

^{1}H NMR: (500 MHz, Chloroform-*d*) δ 7.47 (d, J = 8.7 Hz, 2H), 7.42 – 7.37 (m, 4H), 7.34 – 7.31 (m, 2H), 7.27 – 7.25 (m, 2H), 6.93 (s, 1H), 6.84 – 6.81 (m, 2H), 5.84 (t, J = 15.7 Hz, 1H), 3.80 (s, 3H), 1.30 (s, 9H), 1.27 (s, 9H).

^{19}F NMR: (471 MHz, Chloroform-*d*) δ -80.9 (t, J = 10.0 Hz, 3F), -103.65 – -103.71 (m, 2F), -123.59 – -124.65 (m, 2F), -125.56 – -125.63 (m, 2F).

^{13}C NMR: (126 MHz, Chloroform-*d*) δ 159.3, 153.0 (t, J = 4.2 Hz), 152.2, 150.9, 140.8, 137.5, 133.1, 130.7, 129.1 (t, J = 3.5 Hz), 129.0, 128.9, 126.3, 125.6, 125.0, 115.7 (t, J = 20.2 Hz), 113.9, 55.4, 34.8, 34.7, 31.4, 31.3.

HRMS (EI): calc'd for $C_{35}H_{35}F_9O$ Exact Mass: 642.2544, found 642.2550.

(((*1Z,3E*)-5,5,6,6,7,7,8,8,8-nonafluoro-1-(4-methoxyphenyl)octa-1,3-diene-2,3-diyl)bis(4,1-phenylene))bis(methylsulfane) (10)

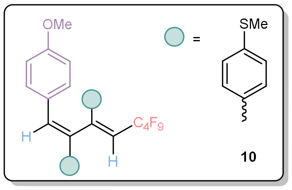

Following the general **procedure D**, **10** (76 mg, 0.122 mmol) was obtained as colorless liquid in 61% yield.

R$_f$ = 0.6 (silica gel, petroleum ether/ethyl acetate = 10:1).

^{1}H NMR: (500 MHz, Chloroform-*d*) δ 7.47 (d, J = 8.6 Hz, 2H), 7.39 (d, J = 8.4 Hz, 2H), 7.33 – 7.31 (m, 2H), 7.16 (d, J = 8.6 Hz, 2H), 7.11 (d, J = 8.6 Hz, 2H), 6.92 (s, 1H), 6.84 (d, J = 9.0 Hz, 2H), 5.90 (t, J = 15.6 Hz, 1H), 3.81 (s, 3H), 2.46 (s, 3H), 2.43 (s, 3H).

^{19}F NMR: (471 MHz, Chloroform-*d*) δ -80.9 (t, J = 10.0 Hz, 3F), -103.6 – -103.64 (m, 2F), -123.5 – -123.6 (m, 2F), -125.6 – -125.7 (m, 2F).

^{13}C NMR: (126 MHz, Chloroform-*d*) δ 159.4, 152.1 (t, J = 3.9 Hz), 140.4, 140.3, 138.3, 137.0, 132.4, 130.6, 129.7 (t, J = 3.3 Hz), 129.4, 128.6, 127.2, 126.4, 125.4, 116.1 (t, J = 20.3 Hz), 114.0, 55.4, 15.6, 15.1.

HRMS (EI): calc'd for $C_{29}H_{23}F_9OS_2$ Exact Mass: 622.1047, found 622.1036.

4,4',4''-((*1Z,3E*)-5,5,6,6,7,7,8,8,8-nonafluoroocta-1,3-diene-1,2,3-triyl)tris(methoxybenzene) (11)

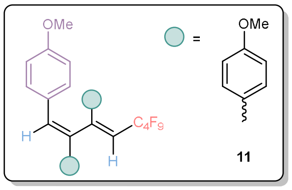

Following the general **procedure D**, **11** (66 mg, 0.112 mmol) was obtained as colorless liquid in 56% yield.

R$_f$ = 0.5 (silica gel, petroleum ether/ethyl acetate = 10:1).

^{1}H NMR: (500 MHz, Chloroform-*d*) δ 7.45 (dd, J = 8.6, 14.0 Hz, 4H), 7.34 (d, J = 7.0 Hz, 2H), 6.86 (s, 1H), 6.84 – 6.78 (m, 6H), 5.85 (t, J = 15.7 Hz, 1H), 3.81 (s, 3H), 3.78 (s, 3H), 3.76 (s, 3H).

^{19}F NMR: (471 MHz, Chloroform-*d*) δ -80.9 (t, J = 9.2 Hz, 3F), -103.49 (s, 2F), -123.6 – -123.7 (m, 2F), -125.6 – -125.7 (m, 2F).

^{13}C NMR: (126 MHz, Chloroform-*d*) δ 160.2, 159.3, 159.1, 152.6 (t, J = 4.5 Hz), 140.8, 133.0, 130.9 (t, J = 3.4 Hz), 130.5, 129.0, 128.4, 128.2, 128.1, 114.8 (t, J = 19.6 Hz), 114.0, 113.9, 113.5, 55.34, 55.30, 55.20.

HRMS (EI): calc'd for $C_{29}H_{23}F_9O_3$ Exact Mass: 590.1503, found 590.1500.

(((*1Z,3E*)-5,5,6,6,7,7,8,8,8-nonafluoro-1-(4-methoxyphenyl)octa-1,3-diene-2,3-diyl)bis(4,1-phenylene))bis(trimethylsilane) (12)

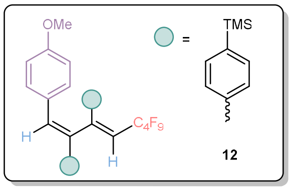

Following the general **procedure D**, **12** (83.5 mg, 0.124 mmol) was obtained as colorless liquid in 62% yield.

R*f* = 0.2 (silica gel, petroleum ether).

^{1}H NMR: (500 MHz, Chloroform-*d*) δ 7.52 – 7.49 (m, 2H), 7.47 – 7.41 (m, 8H), 6.97 (s, 1H), 6.86 – 6.83 (m, 2H), 5.91 (t, 1H), 3.81 (s, 3H), 0.26 (s, 9H), 0.24 (s, 9H).

^{19}F NMR: (471 MHz, Chloroform-*d*) δ -80.9 (t, J = 9.1 Hz, 3F), -103.7 – -103.8 (m, 2F), -123.5 – -123.6 (m, 2F), -125.6 – -125.7 (m, 2F).

^{13}C NMR: (126 MHz, Chloroform-*d*) δ 159.4, 152.8 (t, J = 4.5 Hz), 141.8, 140.7, 140.6, 140.1, 136.3, 133.7, 133.1, 130.8, 129.9, 128.8, 128.3 (t, J = 3.0 Hz), 125.9, 116.6 (t, J = 20.3 Hz), 114.0, 55.4, -1.1, -1.0.

HRMS (EI): calc'd for $C_{33}H_{35}F_9OSi_2$ Exact Mass: 674.2083, found 674.2089.

4,4'-((*1Z,3E*)-5,5,6,6,7,7,8,8,8-nonafluoro-1-(4-methoxyphenyl)octa-1,3-diene-2,3-diyl)bis(fluorobenzene) (13)

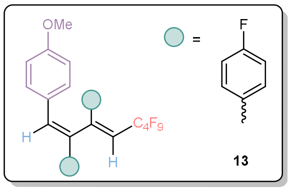

Following the general **procedure D**, **13** (72.4 mg, 0.128 mmol) was obtained as colorless liquid in 64% yield.

R*f* = 0.2 (silica gel, petroleum ether).

^{1}H NMR: (500 MHz, Chloroform-*d*) δ 7.46 – 7.42 (m, 2H), 7.41 – 7.38 (m, 2H), 7.36 – 7.32 (m, 2H), 7.01 – 6.91 (m, 4H), 6.86 (d, J = 2.5 Hz, 2H), 6.84 (s, 1H), 5.93 (t, J = 15.4 Hz, 1H), 3.81 (s, 3H).

^{19}F NMR: (471 MHz, Chloroform-*d*) δ -82.8 (s, 3F), -105.7 (s, 2F), -113.5 (s, F), -115.9 (s, F), -125.4 (s, 2F), -127.6 (s, 2F).

^{13}C NMR: (126 MHz, Chloroform-*d*) δ 163.1 (d, J = 249.5 Hz), δ 162.6 (d, J = 247.7 Hz), 159.6, 151.7 (t, J = 3.9 Hz), 140.0, 136.5, 132.1 (d, J = 3.6 Hz), 131.1 (d, J = 8.4 Hz), 130.6, 130.4, 128.8 (d, J = 8.0 Hz), 128.4, 117.0 (t, J = 20.5 Hz), 115.6 (d, J = 21.5 Hz), 115.3 (d, J = 21.6 Hz), 114.1, 55.4.

HRMS (EI): calc'd for $C_{27}H_{17}F_{11}O$ Exact Mass: 566.1104, found 566.1113.

4,4'-(*(1Z,3E)*-5,5,6,6,7,7,8,8,8-nonafluoro-1-(4-methoxyphenyl)octa-1,3-diene-2,3-diyl)bis(chlorobenzene) (14)

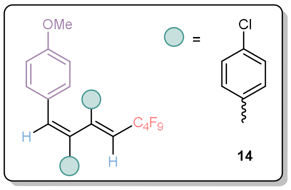

Following the general **procedure D**, **14** (76.5 mg, 0.128 mmol) was obtained as colorless liquid in 64% yield.

R*f* = 0.2 (silica gel, petroleum ether).

^{1}H NMR: (500 MHz, Chloroform-*d*) δ 7.47 (d, J = 7.5 Hz, 2H), 7.37 (d, J = 7.2 Hz, 2H), 7.33 – 7.24 (m, 6H), 6.92 (s, 1H), 6.87 (d, J = 6.9 Hz, 2H), 5.98 (t, J = 15.3 Hz, 1H), 3.83 (s, 3H).

^{19}F NMR: (471 MHz, Chloroform-*d*) δ -80.9 (t, J = 9.1 Hz, 3F), -103.8 – -103.9 (m, 2F), -123.4 – -123.5 (m, 2F), -125.6 – -125.7 (m, 2F).

^{13}C NMR: (126 MHz, Chloroform-*d*) δ 159.7, 151.4 (t, J = 4.3 Hz), 139.5, 138.7, 135.3, 134.5, 133.9, 131.0, 130.7, 130.5 (t, J = 2.8 Hz), 128.9, 128.5, 128.3, 128.2, 117.6 (t, J = 20.5 Hz), 114.1, 55.4.

HRMS (EI): calc'd for $C_{27}H_{17}Cl_2F_9O$ Exact Mass: 598.0513, found 598.0506.

4,4'-(*(1Z,3E)*-5,5,6,6,7,7,8,8,8-nonafluoro-1-(4-methoxyphenyl)octa-1,3-diene-2,3-diyl)bis(brombenzene) (15)

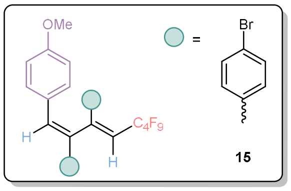

Following the general **procedure D**, **15** (90.5 mg, 0.132 mmol) was obtained as colorless liquid in 66% yield.

R*f* = 0.2 (silica gel, petroleum ether).

^{1}H NMR: (500 MHz, Chloroform-*d*) δ 7.47 – 7.44 (m, 2H), 7.44 – 7.41 (m, 2H), 7.40 – 7.38 (m, 2H), 7.29 (d, J = 8.3 Hz, 2H), 7.25 – 7.22 (m, 2H), 6.91 (s, 1H), 6.87 – 6.84 (m, 2H), 5.97 (t, J = 15.3 Hz, 1H), 3.82 (s, 3H).

^{19}F NMR: (471 MHz, Chloroform-*d*) δ -80.9 (t, J = 9.7 Hz, 3F), -103.8 – -103.9 (m, 2F), -123.4 – -123.5 (m, 2F), -125.6 – -125.7 (m, 2F).

^{13}C NMR: (126 MHz, Chloroform-*d*) δ 159.8, 151.3 (t, J = 3.9 Hz), 139.5, 139.2, 135.0, 131.9, 131.4, 131.0, 130.7, 128.6, 128.2, 123.7, 122.1, 117.7 (t, J = 20.5 Hz), 114.1, 55.4.

HRMS (EI): calc'd for $C_{27}H_{17}Br_2F_9O$ Exact Mass: 685.9502, found 685.9510.

3,3'-(*(1Z,3E)*-5,5,6,6,7,7,8,8,8-nonafluoro-1-(4-methoxyphenyl)octa-1,3-diene-2,3-diyl)bis(chlorobenzene) (16)

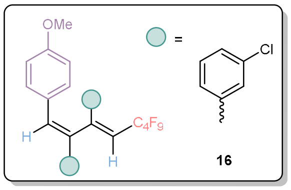

Following the general **procedure D**, **16** (79 mg, 0.132 mmol) was obtained as colorless liquid in 66% yield.

R*f* = 0.2 (silica gel, petroleum ether).

^{1}H NMR: (500 MHz, Chloroform-*d*) δ 7.46 – 7.42 (m, 2H), 7.38 (d, J = 6.3 Hz, 2H), 7.29 (d, J = 7.4 Hz, 1H), 7.25 – 7.22 (m, 4H), 7.22 – 7.17 (m, 1H), 6.93 (s, 1H), 6.87 – 6.84 (m, 2H), 5.97 (t, J = 15.2 Hz, 1H), 3.82 (s, 3H).

^{19}F NMR: (471 MHz, Chloroform-*d*) δ -80.9 (t, J = 9.0 Hz, 3F), -103.85 – -103.93 (m, 2F), -123.4 – -123.5 (m, 2F), -125.6 – -125.7 (m, 2F).

^{13}C NMR: (126 MHz, Chloroform-*d*) δ 159.8, 151.0 (t, J = 4.6 Hz), 142.2, 139.2, 137.8, 134.7, 134.1, 132.0, 130.8, 129.9 (t, J = 3.2 Hz), 129.4, 129.3, 128.9, 128.1, 127.4, 127.22, 127.18, 125.3, 118.4 (t, J = 21.4 Hz), 114.1, 55.4.

HRMS (EI): calc'd for $C_{27}H_{17}Cl_2F_9O$ Exact Mass: 598.0513, found 598.0509.

2,2'-(*(1E,3Z)*-5,5,6,6,7,7,8,8,8-nonafluoro-1-(4-methoxyphenyl)octa-1,3-diene-2,3-diyl)bis(fluorobenzene) (17)

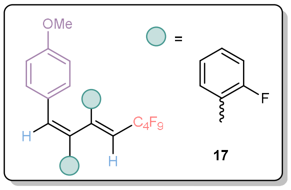

Following the general **procedure D**, **17** (58.9 mg, 0.104 mmol) was obtained as colorless liquid in 52% yield.

R*f* = 0.2 (silica gel, petroleum ether).

^{1}H NMR: (500 MHz, Chloroform-*d*) δ 7.50 – 7.47 (m, 2H), 7.32 – 7.15 (m, 4H), 7.08 – 7.05 (m, 1H), 7.02 – 6.95 (m, 2H), 6.92 – 6.84 (m, 3H), 6.82 (s, 1H), 6.06 (t, J = 15.0 Hz, 1H), 3.83 (s, 3H).

^{19}F NMR: (471 MHz, Chloroform-*d*) δ -81.0 (t, J = 9.1 Hz, 3F), -108.3 – -108.4 (m, 2F), -111.7 (s, F), -113.8 (s, F), -123.3 – -123.4 (m, 2F), -125.8 – -125.9 (m, 2F).

^{13}C NMR: (126 MHz, Chloroform-*d*) δ 160.2 (d, J = 248.1 Hz), 159.7 (d, J = 248.6 Hz), 159.5, 145.6 (t, J = 6.1 Hz), 135.3, 135.1, 131.3 (d, J = 3.1 Hz), 130.9, 130.8 (d, J = 2.2 Hz), 130.5 (d, J = 8.2 Hz), 129.8 (d, J = 8.4 Hz), 129.4 (d, J = 13.7 Hz), 128.3, 124.4 (d, J = 14.9 Hz), 124.1 (d, J = 3.6 Hz), 123.4 (d, J = 3.3 Hz), 119.3 (t, J = 20.5 Hz), 115.8 (d, J = 22.4 Hz), 115.2 (d, J = 22.3 Hz), 113.9, 55.4.

HRMS (ESI): calc'd for $C_{27}H_{17}F_{11}KO^{+}$ Exact Mass: 605.0735, found 605.0741.

4,4'-(*(1Z,3E)*-5,5,6,6,7,7,8,8,8-nonafluoro-1-(4-methoxyphenyl)octa-1,3-diene-2,3-diyl)diben-zonitrile (18)

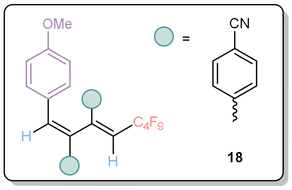

Following the general **procedure D**, **18** (69.6 mg, 0.12 mmol) was obtained as colorless liquid in 60% yield.

R*f* = 0.2 (silica gel, petroleum ether/ethyl acetate = 10:1).

^{1}H NMR: (500 MHz, Chloroform-*d*) δ 7.61 – 7.58 (m, 2H), 7.54 – 7.52 (m, 2H), 7.46 (d, J = 8.5 Hz, 4H), 7.44 – 7.41 (m, 2H), 7.02 (s, 1H), 6.87 – 6.84 (m, 2H), 6.06 (t, J = 15.1 Hz, 1H), 3.83 – 3.81 (m, 3H).

^{19}F NMR: (471 MHz, Chloroform-*d*) δ -80.9 (t, J = 9.1 Hz, 3F), -104.0 – -104.1 (m, 2F), -123.28 – -123.34 (m, 2F), -125.6 – -125.7 (m, 2F).

^{13}C NMR: (126 MHz, Chloroform-*d*) δ 160.3, 150.2 (t, J = 4.4 Hz), 144.6, 140.6, 138.2, 134.1, 132.6, 131.9, 130.9, 129.6 (t, J = 3.6 Hz), 127.6, 127.4, 120.0 (t, J = 21.0 Hz), 118.6, 118.2, 114.3, 113.1, 111.7, 55.5.

HRMS (EI): calc'd for $C_{29}H_{17}F_9N_2O$ Exact Mass: 580.1197, found 580.1188.

1,1'-((*(1Z,3E)*-5,5,6,6,7,7,8,8,8-nonafluoro-1-(4-methoxyphenyl)octa-1,3-diene-2,3-diyl)bis(4,1-phenylene))bis(ethan-1-one) (19)

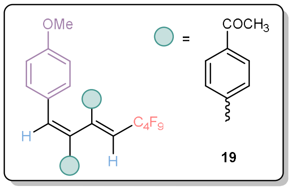

Following the general **procedure D, 19** (79.8 mg, 0.13 mmol) was obtained as colorless liquid in 65% yield.

R*f* = 0.2 (silica gel, petroleum ether/ethyl acetate = 10:1).

^{1}H NMR: (500 MHz, Chloroform-*d*) δ 7.89 – 7.86 (m, 2H), 7.83 – 7.81 (m, 2H), 7.51 – 7.45 (m, 6H), 7.03 (s, 1H), 6.87 – 6.84 (m, 2H), 6.04 (t, J = 15.2 Hz, 1H), 3.81 (s, 3H), 2.55 (s, 3H), 2.52 (s, 3H).

^{19}F NMR: (471 MHz, Chloroform-*d*) δ -80.9 (t, J = 9.0 Hz, 3F), -104.0 – -104.1 (m, 2F), -123.3 – -123.4 (m, 2F), -125.6 – -125.7 (m, 2F).

^{13}C NMR: (126 MHz, Chloroform-*d*) δ 197.5, 197.4, 160.0, 151.3 (t, J = 4.3 Hz), 144.7, 140.7, 139.2, 137.3, 136.3, 132.5, 130.9, 129.3 (t, J = 3.0 Hz), 128.8, 128.1, 127.9, 127.1, 118.7 (t, J = 20.5 Hz), 114.1, 55.4, 26.6.

HRMS (EI): calc'd for $C_{31}H_{23}F_9O_3$ Exact Mass: 614.1503, found 614.1497.

4,4'-(*(1Z,3E)*-5,5,6,6,7,7,8,8,8-nonafluoro-1-(4-methoxyphenyl)octa-1,3-diene-2,3-diyl)diben- zaldehyde (20)

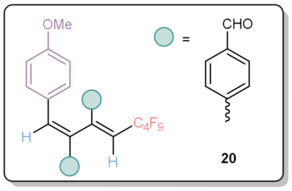

Following the general **procedure D**, **20** (60.9 mg, 0.104 mmol) was obtained as colorless liquid in 52% yield.

R*f* = 0.2 (silica gel, petroleum ether/ethyl acetate = 10:1).

^{1}H NMR: (500 MHz, Chloroform-*d*) δ 9.95 (s, 1H), 9.93 (s, 1H), 7.82 – 7.80 (m, 2H), 7.76 – 7.74 (m, 2H), 7.57 – 7.53 (m, 4H), 7.50 – 7.47 (m, 2H), 7.06 (s, 1H), 6.88 – 6.85 (m, 2H), 6.08 (t, J = 15.2 Hz, 1H), 3.82 (s, 3H).

^{19}F NMR: (471 MHz, Chloroform-*d*) δ -80.9 (t, J = 10.3 Hz, 3F), -104.0 – -104.1 (m, 2F), -123.28 – -123.34 (m, 2F), -125.6 – -125.7 (m, 2F).

^{13}C NMR: (126 MHz, Chloroform-*d*) δ 191.6, 160.1, 151.1 (t, J = 4.0 Hz), 146.2, 142.1, 139.1, 136.4, 135.7, 133.4, 131.0, 130.2, 129.7 (t, J = 3.0 Hz), 129.4, 127.8, 127.6, 119.4 (t, J = 20.8 Hz), 114.2, 55.4.

HRMS (EI): calc'd for $C_{29}H_{19}F_9O_3$ Exact Mass: 586.1190, found 586.1199.

4,4'-(*(1Z,3E)*-5,5,6,6,7,7,8,8,8-nonafluoro-1-(4-methoxyphenyl)octa-1,3-diene-2,3-diyl)bis((difluoromethyl)benzene) (21)

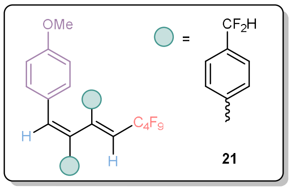

Following the general **procedure D**, **21** (69.3 mg, 0.11 mmol) was obtained as colorless liquid in 55% yield.

R*f* = 0.2 (silica gel, petroleum ether).

^{1}H NMR: (500 MHz, Chloroform-*d*) δ 7.51 – 7.43 (m, 8H), 7.39 (d, J = 8.3 Hz, 2H), 6.97 (s, 1H), 6.88 – 6.85 (m, 2H), 6.59 (td, J = 16.1, 56.4 Hz, 2H), 6.02 (t, J = 15.4 Hz, 1H), 3.82 (s, 3H).

^{19}F NMR: (471 MHz, Chloroform-*d*) δ -80.9 (t, J = 9.3 Hz, 3F), -103.9 – -104.0 (m, 2F), -110.7 (s, F), -110.8 (s, F), -111.5 (s, F), -111.7 (s, F), -123.4 – -123.5 (m, 2F), -125.6 – -125.8 (m, 2F).

^{13}C NMR: (126 MHz, Chloroform-*d*) δ 159.8, 151.5 (t, J = 4.4 Hz), 142.6, 139.6, 138.5 (t, J = 2.0 Hz), 134.9 (t, J = 22.6 Hz), 133.8 (t, J = 22.9 Hz), 131.9, 130.8, 129.4 (t, J = 2.8 Hz), 128.1, 127.3, 126.0 (t, J = 6.0 Hz), 125.4 (t, J = 5.9 Hz), 118.3 (t, J = 20.1 Hz), 114.6 (t, J = 238.8 Hz), 114.4 (t, J = 239.3 Hz), 114.1, 55.4.

HRMS (EI): calc'd for $C_{29}H_{19}F_{13}O$ Exact Mass: 630.1228, found 630.1235.

4,4'-(*(1Z,3E)*-5,5,6,6,7,7,8,8,8-nonafluoro-1-(4-methoxyphenyl)octa-1,3-diene-2,3-diyl)bis((difluoromethoxy)benzene) (22)

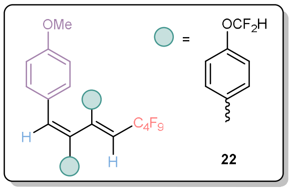

Following the general **procedure D**, **22** (92.6 mg, 0.14 mmol) was obtained as colorless liquid in 70% yield.

R$_f$ = 0.3 (silica gel, petroleum ether/ethyl acetate = 30:1).

^{1}H NMR: (500 MHz, Chloroform-*d*) δ 7.46 – 7.42 (m, 4H), 7.39 – 7.36 (m, 2H), 7.07 – 7.04 (m, 2H), 7.01 – 6.98 (m, 2H), 6.90 (s, 1H), 6.86 – 6.83 (m, 2H), 6.48 (td, J = 4.4, 73.7 Hz, 2H), 5.95 (t, J = 15.5 Hz, 1H), 3.81 (s, 3H).

^{19}F NMR: (471 MHz, Chloroform-*d*) δ -80.8 (s, 2F), -80.9 (t, J = 9.4 Hz, 3F), -81.1 (s, 2F), -103.2 – -103.9 (m, 2F), -123.5 – -123.6 (m, 2F), -125.7 – -125.8 (m, 2F).

^{13}C NMR: (126 MHz, Chloroform-*d*) δ 159.7, 151.8 (t, J = 2.6 Hz), 151.5 (t, J = 3.7 Hz), 150.9 (t, J = 2.7 Hz), 139.6, 137.5, 133.1, 130.8 (t, J = 3.3 Hz), 130.74, 130.65, 128.4, 128.3, 119.6, 118.7, 117.2 (t, J = 20.4 Hz), 115.9 (t, J = 260.0 Hz), 115.8 (t, J = 259.9 Hz), 114.1, 55.4.

HRMS (EI): calc'd for $C_{29}H_{19}F_{13}O_3$Exact Mass: 662.1127, found: 662.1136.

4,4'-(*(1Z,3E)*-5,5,6,6,7,7,8,8,8-nonafluoro-1-(4-methoxyphenyl)octa-1,3-diene-2,3-diyl)bis((tri- -fluoromethoxy)benzene) (23)

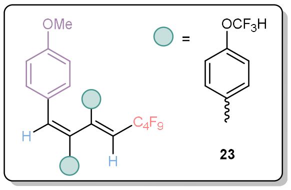

Following the general **procedure D**, **23** (97.7 mg, 0.14 mmol) was obtained as colorless liquid in 70% yield.

R$_f$ = 0.3 (silica gel, petroleum ether/ethyl acetate = 30:1).

^{1}H NMR: (500 MHz, Chloroform-*d*) δ 7.43 – 7.38 (m, 6H), 7.15 (d, J = 7.8 Hz, 2H), 7.08 (d, J = 7.9 Hz, 2H), 6.91 (s, 1H), 6.85 – 6.82 (m, 2H), 5.95 (t, J = 15.2 Hz, 1H), 3.81 (s, 3H).

^{19}F NMR: (471 MHz, Chloroform-*d*) δ -57.65 (s, 3F), -57.74 (s, 3F), -80.9 (t, J = 9.4 Hz, 3F), -103.9 – -104.0 (m, 2F), -123.5 – -123.6 (m, 2F), -125.6 – -125.7 (m, 2F).

^{13}C NMR: (126 MHz, Chloroform-*d*) δ 159.8, 151.2 (t, J = 5.3 Hz), 149.7, 148.9, 139.4, 139.0, 134.5, 131.5, 130.7 (t, J = 3.3 Hz), 130.7, 128.4, 128.2, 121.1, 120.48 (q, J = 258.0 Hz), 120.52 (q, J = 256.8 Hz), 120.3, 117.9 (t, J = 20.5 Hz), 114.1, 55.4.

HRMS (EI): calc'd for $C_{29}H_{17}F_{15}O_3$ Exact Mass: 698.0938, found 698.0933.

2,2'-(*(1E,3Z)*-5,5,6,6,7,7,8,8,8-nonafluoro-1-(4-methoxyphenyl)octa-1,3-diene-2,3-diyl)dithiophene (24)

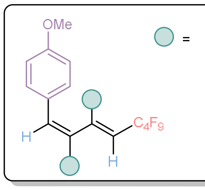

24

Following the general **procedure D**, **24** (56.4 mg, 0.104 mmol) was obtained as colorless liquid in 52% yield.

R*f* = 0.2 (silica gel, petroleum ether).

^{1}H NMR: (500 MHz, Chloroform-*d*) δ 7.52 (d, J = 2.8 Hz, 1H), 7.45 – 7.42 (m, 2H), 7.37 (d, J = 5.0 Hz, 1H), 7.19 – 7.18 (m, 1H), 7.02 – 7.00 (m, 1H), 6.98 (s, 1H), 6.97 – 6.94 (m, 2H), 6.84 – 6.81 (m, 2H), 5.79 (t, J = 16.0 Hz, 1H), 3.80 (s, 3H).

^{19}F NMR: (471 MHz, Chloroform-*d*) δ -80.9 (t, J = 9.4 Hz, 3F), -105.4 (s, 2F), -123.8 – -123.9 (m, 2F), -125.5 – -125.6 (m, 2F).

^{13}C NMR: (126 MHz, Chloroform-*d*) δ 159.5, 145.2, 144.6 (t, J = 4.4 Hz), 137.3, 134.5, 131.5 (t, J = 5.7 Hz), 130.8, 129.6, 127.9, 127.8, 127.7, 127.6, 125.3, 124.8, 114.1, 114.0 (t, J = 21.9 Hz), 55.4.

HRMS (ESI): calc'd for $C_{23}H_{16}F_9OS_2^+$ Exact Mass: 543.0493, found 543.0497.

5,5'-(*(1Z,3E)*-5,5,6,6,7,7,8,8,8-nonafluoro-1-(4-methoxyphenyl)octa-1,3-diene-2,3-diyl)bis(1,3-dimethylbenzene) (25)

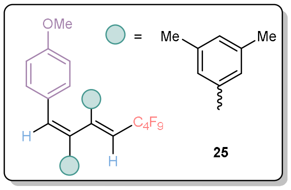

25

Following the general **procedure D**, **25** (71.5 mg, 0.122 mmol) was obtained as colorless liquid in 61% yield.

R*f* = 0.2 (silica gel, petroleum ether).

^{1}H NMR: (500 MHz, Chloroform-*d*) δ 7.46 (d, J = 8.0 Hz, 2H), 7.03 (d, J = 4.9 Hz, 4H), 6.88 (d, J = 5.3 Hz, 3H), 6.82 (d, J = 9.1 Hz, 2H), 5.85 (t, J = 15.6 Hz, 1H), 3.80 (s, 3H), 2.28 (s, 6H), 2.23 (s, 6H).

^{19}F NMR: (471 MHz, Chloroform-*d*) δ -80.9 (t, J = 9.4 Hz, 3F), -103.5 – -103.6 (m, 2F), -123.5 – -123.6 (m, 2F), -125.6 – -125.7 (m, 2F).

^{13}C NMR: (126 MHz, Chloroform-*d*) δ 159.2, 153.3 (t, J = 3.7 Hz), 141.5, 140.5, 137.9, 137.3, 136.3, 130.8, 130.7, 129.6, 129.5, 129.0, 127.0 (t, J = 3.4 Hz), 125.0, 116.1 (t, J = 20.4 Hz), 113.9, 55.4, 21.5, 21.4.

HRMS (EI): calc'd for $C_{31}H_{27}F_9O$ Exact Mass: 586.1918, found 586.1925.

5,5'-(*(1Z,3E)*-5,5,6,6,7,7,8,8,8-nonafluoro-1-(4-methoxyphenyl)octa-1,3-diene-2,3-diyl)bis(1,3-dimethoxybenzene) (26)

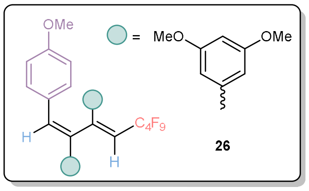

Following the general **procedure D**, **26** (65 mg, 0.1 mmol) was obtained as colorless liquid in 50% yield.

R*f* = 0.4 (silica gel, petroleum ether/ethyl acetate = 10:1).

^{1}H NMR: (500 MHz, Chloroform-*d*) δ 7.49 – 7.46 (m, 2H), 6.90 (s, 1H), 6.85 – 6.82 (m, 2H), 6.59 (d, J = 2.2 Hz, 2H), 6.55 (d, J = 2.2 Hz, 2H), 6.37 – 6.35 (m, 2H), 5.89 (t, J = 15.4 Hz, 1H), 3.81 (s, 3H), 3.76 (s, 6H), 3.70 (s, 6H).

^{19}F NMR: (471 MHz, Chloroform-*d*) δ -80.9 (t, J = 9.4 Hz, 3F), -104.0 – -104.1 (m, 2F), -123.47 – -123.53 (m, 2F), -125.5 – -125.8 (m, 2F).

^{13}C NMR: (126 MHz, Chloroform-*d*) δ 160.8, 160.3, 159.5, 152.4 (t, J = 3.4 Hz), 142.4, 140.8, 138.0, 130.7, 130.3, 128.5, 117.0 (t, J = 20.4 Hz), 114.0, 107.5 (t, J = 2.8 Hz), 105.4, 101.1, 100.0, 55.42, 55.39.

HRMS (ESI): calc'd for $C_{31}H_{28}F_9O_5^+$ Exact Mass: 651.1788, found 651.1787.

5,5'-(*(1Z,3E)*-5,5,6,6,7,7,8,8,8-nonafluoro-1-(4-methoxyphenyl)octa-1,3-diene-2,3-diyl)bis(1,2,3-trimethoxybenzene) (27)

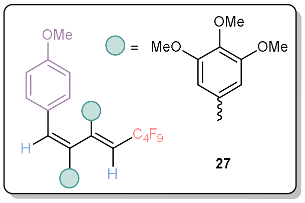

Following the general **procedure D**, **27** (49.7 mg, 0.07 mmol) was obtained as colorless liquid in 35% yield.

R*f* = 0.2 (silica gel, petroleum ether/ethyl acetate = 5:1).

^{1}H NMR:(500 MHz, Chloroform-*d*) δ 7.46 – 7.43 (m, 2H), 6.89 (s, 1H), 6.85 – 6.82 (m, 2H), 6.66 (s, 2H), 6.64 (s, 2H), 5.86 (t, J = 15.6 Hz, 1H), 3.84 (s, 3H), 3.84 (s, 6H), 3.83 (s, 3H), 3.80 (s, 3H), 3.72 (s, 6H).

^{19}F NMR:(471 MHz, Chloroform-*d*) δ -80.9 (t, J = 9.4 Hz, 3F), -103.85 – -103.94 (m, 2F), -123.45 – -123.53 (m, 2F), -125.5 – -125.6 (m, 2F).

^{13}C NMR:(126 MHz, Chloroform-*d*) δ 159.5, 153.3, 152.7, 141.0, 138.9, 138.1, 136.2, 131.5, 131.3 (t, J = 2.4 Hz), 130.6, 129.6, 128.6, 116.3 (t, J = 20.2 Hz), 114.0, 106.8 (t, J = 3.1 Hz), 104.3, 61.1, 61.0, 56.2, 56.1, 55.4.

HRMS (ESI): calc'd for $C_{33}H_{31}F_9O_7$ Exact Mass: 710.1926, found 710.1922.

5,5'-(*(1Z,3E)*-5,5,6,6,7,7,8,8,8-nonafluoro-1-(4-methoxyphenyl)octa-1,3-diene-2,3-diyl)bis(benzo[d][1,3]dioxole) (28)

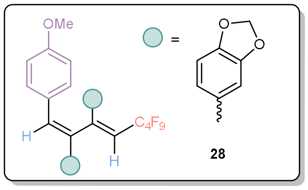

Following the general **procedure D**, **28** (69.2 mg, 0.112 mmol) was obtained as colorless liquid in 56% yield.

R*f* = 0.5 (silica gel, petroleum ether/ethyl acetate = 10:1).

^{1}H NMR: (500 MHz, Chloroform-*d*) δ 7.46 – 7.43 (m, 2H), 7.02 – 7.00 (m, 1H), 6.96 (s, 1H), 6.88 – 6.82 (m, 5H), 6.74 – 6.70 (m, 2H), 5.93 (d, J = 1.2 Hz, 4H), 5.85 (t, J = 15.6 Hz, 1H), 3.80 (s, 3H).

^{19}F NMR: (471 MHz, Chloroform-*d*) δ -80.9 (t, J = 9.4 Hz, 3F), -103.4 – -103.5 (m, 2F), -123.6 – -123.7 (m, 2F), -125.6 – -125.7 (m, 2F).

^{13}C NMR: (126 MHz, Chloroform-*d*) δ 159.3, 152.2 (t, J = 3.8 Hz), 148.5, 148.0, 147.6, 147.5, 140.7, 134.7, 130.6, 129.8, 129.2, 128.7, 123.8 (t, J = 3.0 Hz), 120.9, 115.6 (t, J = 20.0 Hz), 114.0, 109.7, 108.4, 108.1, 107.3, 101.4, 101.3, 55.4.

HRMS (EI): calc'd for $C_{29}H_{19}F_9O_5$ Exact Mass: 618.1089, found 618.1095.

Dimethyl-4,4'-(*(1Z,3E)*-5,5,6,6,7,7,8,8,8-nonafluoro-1-phenylocta-1,3-diene-2,3-diyl)dibenzoate (29)

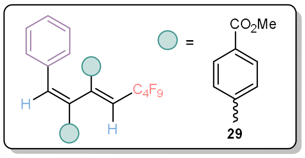

Following the general **procedure D**, **29** (83.8 mg, 0.136 mmol) was obtained as colorless liquid in 68% yield.

R*f* = 0.4 (silica gel, petroleum ether/ethyl acetate = 10:1).

^{1}H NMR: (500 MHz, Chloroform-*d*) δ 7.98 – 7.95 (m, 2H), 7.90 – 7.88 (m, 2H), 7.50 – 7.43 (m, 6H), 7.34 – 7.27 (m, 3H), 7.07 (s, 1H), 5.99 (t, J = 15.0 Hz, 1H), 3.89 (s, 3H), 3.86 (s, 3H).

^{19}F NMR: (471 MHz, Chloroform-*d*) δ -81.0 (t, J = 9.4 Hz, 3F), -104.1 – -104.2 (m, 2F), -123.4 – -123.5 (m, 2F), -125.7 – -125.8 (m, 2F).

^{13}C NMR: (126 MHz, Chloroform-*d*) δ 166.7, 166.5, 151.1 (t, J = 3.8 Hz), 144.4, 141.7, 140.6, 135.5, 133.1, 130.6, 130.0, 129.8, 129.3, 129.2, 129.1 (t, J = 3.3 Hz), 128.7, 128.5, 127.3, 118.9 (t, J = 21.0 Hz), 52.28, 52.27.

HRMS (ESI): calc'd for $C_{30}H_{21}F_9KO_4^+$ Exact Mass: 655.0928, found 655.0926.

Dimethyl-4,4'-(*(1Z,3E)*-5,5,6,6,7,7,8,8,8-nonafluoro-1-(*p*-tolyl)octa-1,3-diene-2,3-diyl)dibenzoate (30)

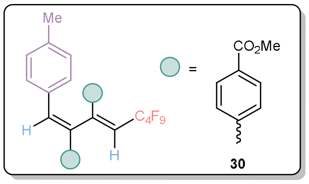

Following the general **procedure D**, **30** (88 mg, 0.14 mmol) was obtained as colorless liquid in 70% yield.

R$_f$ = 0.4 (silica gel, petroleum ether/ethyl acetate = 10:1).

^{1}H NMR: (500 MHz, Chloroform-*d*) δ 7.96 – 7.94 (m, 2H), 7.90 – 7.87 (m, 2H), 7.45 – 7.40 (m, 6H), 7.13 (d, J = 7.9 Hz, 2H), 7.02 (s, 1H), 6.00 (t, J = 15.2 Hz, 1H), 3.89 (s, 3H), 3.86 (s, 3H), 2.35 (s, 3H).

^{19}F NMR: (471 MHz, Chloroform-*d*) δ -82.4 (t, J = 9.3 Hz, 3F), -105.65 – -105.74 (m, 2F), -124.9 – -125.0 (m, 2F), -127.2 – -127.3 (m, 2F).

^{13}C NMR: (126 MHz, Chloroform-*d*) δ 166.8, 166.6, 151.2 (t, J = 4.6 Hz), 144.6, 140.7, 138.8, 132.9, 132.6, 130.6, 130.0, 129.6, 129.4, 129.30, 129.26, 129.1 (t, J = 3.3 Hz), 127.2, 118.7 (t, J = 20.4 Hz), 52.30, 52.27, 21.4.

HRMS (ESI): calc'd for $C_{31}H_{24}F_9O_4^+$ Exact Mass: 631.1525, found 631.1523.

Dimethyl-4,4'-(*(1Z,3E)*-5,5,6,6,7,7,8,8,8-nonafluoro-1-(4-isopropylphenyl)octa-1,3-diene-2,3-diyl)dibenzoate (31)

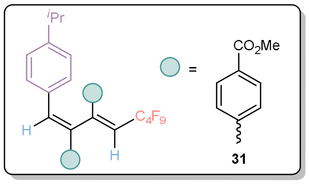

Following the general **procedure D**, **31** (92 mg, 0.14 mmol) was obtained as colorless liquid in 70% yield.

R$_f$ = 0.4 (silica gel, petroleum ether/ethyl acetate = 10:1).

^{1}H NMR: (500 MHz, Chloroform-*d*) δ 7.96 – 7.94 (m, 2H), 7.90 – 7.88 (m, 2H), 7.46 – 7.42 (m, 6H), 7.20 – 7.17 (m, 2H), 7.05 (s, 1H), 5.99 (t, J = 15.2 Hz, 1H), 3.89 (s, 3H), 3.86 (s, 3H), 2.97 – 2.81 (m, 1H), 1.25 (s, 3H), 1.23 (s, 3H).

^{19}F NMR: (471 MHz, Chloroform-*d*) δ -81.0 (t, J = 9.4 Hz, 3F), -103.9 – -104.0 (m, 2F), -123.45 – -123.52 (m, 2F), -125.7 – -125.8 (m, 2F).

^{13}C NMR: (126 MHz, Chloroform-*d*) δ 166.7, 166.5, 151.2 (t, J = 4.7 Hz), 149.7, 144.5, 140.9, 140.7, 133.10, 133.06, 130.6, 130.0, 129.6, 129.3, 129.1 (t, J = 3.5 Hz), 127.2, 126.8, 118.8 (t, J = 20.7 Hz), 52.22, 52.19, 34.1, 23.8.

HRMS (EI): calc'd for $C_{33}H_{27}F_9O_4$ Exact Mass: 658.1766, found 658.1763.

Dimethyl-4,4'-(*(1Z,3E)*-1-(4-(tert-butyl)phenyl)-5,5,6,6,7,7,8,8,8-nonafluoroocta-1,3-diene-2,3-diyl)dibenzoate (32)

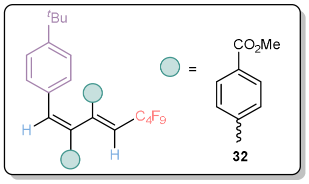

Following the general **procedure D**, **32** (95.4 mg, 0.142 mmol) was obtained as colorless liquid in 71% yield.

R*f* = 0.4 (silica gel, petroleum ether/ethyl acetate = 10:1).

^{1}H NMR: (500 MHz, Chloroform-*d*) δ 7.97 – 7.95 (m, 2H), 7.91 – 7.89 (m, 2H), 7.49 – 7.42 (m, 6H), 7.37 – 7.34 (m, 2H), 7.07 (s, 1H), 6.00 (t, J = 15.2 Hz, 1H), 3.89 (s, 3H), 3.86 (s, 3H), 1.32 (s, 9H).

^{19}F NMR: (471 MHz, Chloroform-*d*) δ -81.0 (t, J = 9.4 Hz, 3F), -103.8 – -103.9 (m, 2F), -123.47 – -123.54 (m, 2F), -125.76 – -125.82 (m, 2F).

^{13}C NMR: (126 MHz, Chloroform-*d*) δ 166.7, 151.9, 151.2 (t, J = 4.4 Hz), 144.5, 141.0, 140.7, 133.0, 132.7, 130.0, 129.3, 129.2 (t, J = 2.5 Hz), 129.0, 127.2, 125.6, 118.8 (t, J = 20.9 Hz), 77.4, 77.2, 76.9, 52.3, 52.2, 34.8, 31.2.

HRMS (EI): calc'd for $C_{34}H_{30}F_9O_4^+$ Exact Mass: 673.1995, found 673.2000.

Dimethyl-4,4'-(*(1Z,3E)*-5,5,6,6,7,7,8,8,8-nonafluoro-1-(4-(trimethylsilyl)phenyl)octa-1,3-diene-2,3-diyl)dibenzoate (33)

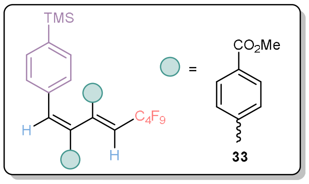

Following the general **procedure D**, **33** (97.7 mg, 0.142 mmol) was obtained as colorless liquid in 71% yield.

R*f* = 0.4 (silica gel, petroleum ether/ethyl acetate = 10:1).

^{1}H NMR: (500 MHz, Chloroform-*d*) δ 7.97 – 7.94 (m, 2H), 7.90 – 7.88 (m, 2H), 7.48 (s, 4H), 7.46 – 7.42 (m, 4H), 7.07 (s, 1H), 5.98 (t, J = 15.1 Hz, 1H), 3.89 (s, 3H), 3.87 (s, 3H), 0.26 (s, 9H).

^{19}F NMR: (471 MHz, Chloroform-*d*) δ -81.0 (t, J = 9.4 Hz, 3F), -103.9 – -104.0 (m, 2F), -123.5 – -123.6 (m, 2F), -125.76 – -125.82 (m, 2F).

^{13}C NMR: (126 MHz, Chloroform-*d*) δ 166.7, 166.5, 151.0 (t, J = 4.1 Hz), 144.3, 141.8, 141.4, 140.6, 135.8, 133.7, 133.2, 130.6, 130.0, 129.8, 129.3, 129.1 (t, J = 3.1 Hz), 128.4, 127.3, 118.9 (t, J = 20.5 Hz), 52.30, 52.29, -1.2.

HRMS (EI): calc'd for $C_{33}H_{29}F_9O_4Si$ Exact Mass: 688.1691, found 688.1682.

Dimethyl-4,4'-(*(1Z,3E)*-5,5,6,6,7,7,8,8,8-nonafluoro-1-(4-((tetrahydro-2H-pyran-2-yl)oxy)phen- yl)octa-1,3-diene-2,3-diyl)dibenzoate (34)

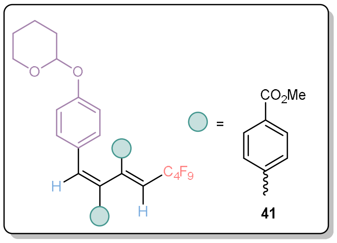

Following the general **procedure D**, **34** (91.6 mg, 0.128 mmol) was obtained as colorless liquid in 64% yield.

R*f* = 0.15 (silica gel, petroleum ether/ethyl acetate = 5:1).

^{1}H NMR: (500 MHz, Chloroform-*d*) δ 7.96 – 7.88 (m, 4H), 7.47 – 7.42 (m, 6H), 7.02 – 6.99 (m, 3H), 6.02 (t, J = 15.1 Hz, 1H), 5.43 (t, J = 3.2 Hz, 1H), 3.88 (s, 3H), 3.85 (s, 3H), 3.62 – 3.58 (m, 1H), 2.02 – 1.96 (m, 1H), 1.88 – 1.85 (m, 2H), 1.73 – 1.57 (m, 4H).

^{19}F NMR: (471 MHz, Chloroform-*d*) δ -80.9 (t, J = 9.4 Hz, 3F), -103.7 – -103.8 (m, 2F), -123.8 – -123.9 (m, 2F), -125.7 – -125.8 (m, 2F).

^{13}C NMR: (126 MHz, Chloroform-*d*) δ 166.7, 166.5, 157.4, 151.4 (t, J = 4.2 Hz), 144.7, 140.7, 139.7, 132.5, 130.7, 130.6, 130.0, 129.5, 129.3, 129.1 (t, J = 2.5 Hz), 128.8, 127.1, 118.7 (t, J = 20.5 Hz), 116.5, 96.3, 62.0, 52.24, 52.20, 30.3, 25.2, 18.7.

HRMS (ESI): calc'd for $C_{35}H_{30}F_{9}O_{6}^{+}$ Exact Mass: 717.1893, found 717.1889.

Dimethyl-4'-(*(1Z,3E)*-5,5,6,6,7,7,8,8,8-nonafluoro-1-(4-(methylthio)phenyl)octa-1,3-diene-2,3-diyl)dibenzoate (35)

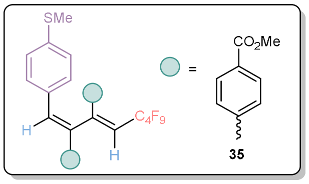

Following the general **procedure D**, **35** (90 mg, 0.136 mmol) was obtained as colorless liquid in 68% yield.

R*f* = 0.3 (silica gel, petroleum ether/ethyl acetate = 10:1).

^{1}H NMR: (500 MHz, Chloroform-*d*) δ 7.97 – 7.89 (m, 4H), 7.45 – 7.43 (m, 6H), 7.20 – 7.17 (m, 2H), 7.00 (s, 1H), 6.01 (t, J = 15.2 Hz, 1H), 3.88 (s, 3H), 3.86 (s, 3H), 2.48 (s, 3H).

^{19}F NMR: (471 MHz, Chloroform-*d*) δ -80.9 (t, J = 9.4 Hz, 3F), -104.1 – -104.2 (m, 2F), -123.3 – -123.4 (m, 2F), -125.7 – -125.8 (m, 2F).

^{13}C NMR: (126 MHz, Chloroform-*d*) δ 166.7, 166.5, 151.2 (t, J = 4.3 Hz), 144.4, 140.9, 140.5, 139.7, 132.2, 132.0, 130.7, 130.0, 129.7, 129.3, 129.0 (t, J = 3.1 Hz), 127.1, 126.3, 118.8 (t, J = 20.7 Hz), 52.29, 52.25, 15.5.

HRMS (EI): calc'd for $C_{31}H_{23}F_{9}O_{4}S$ Exact Mass: 662.1173, found 662.1182.

Dimethyl-4,4'-(*(1Z,3E)*-1-(4-acetoxyphenyl)-5,5,6,6,7,7,8,8,8-nonafluoroocta-1,3-diene-2,3-diyl)dibenzoate (36)

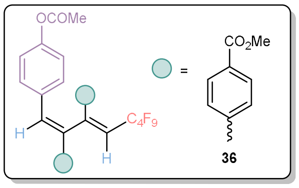

Following the general **procedure D**, **36** (80.9 mg, 0.12 mmol) was obtained as colorless liquid in 60% yield.

R$_f$ = 0.3 (silica gel, petroleum ether/ethyl acetate = 5:1).

^{1}H NMR: (500 MHz, Chloroform-*d*) δ 7.98 – 7.88 (m, 4H), 7.52 – 7.50 (m, 2H), 7.45 – 7.41 (m, 4H), 7.08 – 7.02 (m, 3H), 6.00 (t, J = 15.1 Hz, 1H), 3.89 (s, 3H), 3.86 (s, 3H), 2.30 (s, 3H).

^{19}F NMR: (471 MHz, Chloroform-*d*) δ -80.9 (t, J = 9.4 Hz, 3F), -104.15 – -104.24 (m, 2F), -123.3 – -123.4 (m, 2F), -125.65 – -125.73 (m, 2F).

^{13}C NMR: (126 MHz, Chloroform-*d*) δ 169.2, 166.6, 166.4, 151.0 (t, J = 4.6 Hz), 150.7, 144.2, 141.7, 140.4, 133.1, 131.8, 130.7, 130.3, 130.0, 129.8, 129.3, 129.0 (t, J = 2.8 Hz), 127.2, 121.9, 118.9 (t, J = 21.0 Hz), 52.23, 52.21, 21.1.

HRMS (EI): calc'd for $C_{32}H_{23}F_9O_6$ Exact Mass: 674.1351, found 674.1342.

Dimethyl-4,4'-(*(1Z,3E)*-5,5,6,6,7,7,8,8,8-nonafluoro-1-(naphthalen-2-yl)octa-1,3-diene-2,3-diyl)dibenzoate (37)

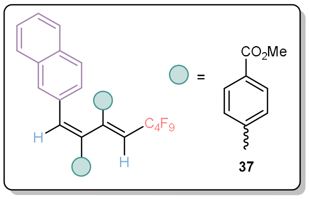

Following the general **procedure D**, **37** (86.5 mg, 0.13 mmol) was obtained as colorless liquid in 65% yield.

R$_f$ = 0.4 (silica gel, petroleum ether/ethyl acetate = 10:1).

^{1}H NMR: (500 MHz, Chloroform-*d*) δ 8.02 – 7.98 (m, 3H), 7.90 – 7.88 (m, 2H), 7.82 – 7.77 (m, 3H), 7.66 – 7.64 (m, 1H), 7.52 – 7.47 (m, 6H), 7.22 (s, 1H), 6.08 (t, J = 15.1 Hz, 1H), 3.90 (s, 3H), 3.85 (s, 3H).

^{19}F NMR: (471 MHz, Chloroform-*d*) δ -81.0 (t, J = 9.4 Hz, 3F), -104.05 – -104.14 (m, 2F), -123.26 – -123.33 (m, 2F), -125.7 – -125.8 (m, 2F).

^{13}C NMR: (126 MHz, Chloroform-*d*) δ 166.7, 166.5, 151.3 (t, J = 3.6 Hz), 144.5, 141.7, 140.6, 133.4, 133.2, 132.9, 130.6, 130.1, 129.8, 129.6, 129.3, 129.0 (t, J = 3.3 Hz), 128.3, 127.8, 127.3, 126.9, 126.7, 126.2, 119.0 (t, J = 20.9 Hz), 52.3.

HRMS (EI): calc'd for $C_{34}H_{23}F_9O_4$ Exact Mass: 666.1453, found 666.1451.

Dimethyl-4,4'-(*(1Z,3E)*-1-([1,1'-biphenyl]-4-yl)-5,5,6,6,7,7,8,8,8-nonafluoroocta-1,3-diene-2,3-diyl)dibenzoate (38)

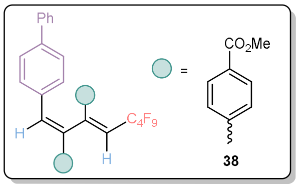

Following the general **procedure D**, **38** (87.2 mg, 0.126 mmol) was obtained as colorless liquid in 63% yield.

R$_f$ = 0.4 (silica gel, petroleum ether/ethyl acetate = 10:1).

^{1}H NMR: (500 MHz, Chloroform-*d*) δ 8.00 – 7.92 (m, 4H), 7.62 – 7.58 (m, 6H), 7.51 – 7.45 (m, 6H), 7.39 – 7.36 (m, 1H), 7.11 (s, 1H), 6.07 (t, J = 15.1 Hz, 1H), 3.90 (s, 3H), 3.87 (s, 3H).

^{19}F NMR: (471 MHz, Chloroform-*d*) δ -80.9 (t, J = 9.4 Hz, 3F), -104.0 – -104.1 (m, 2F), -123.3 – -123.4 (m, 2F), -125.6 – -125.7 (m, 2F).

^{13}C NMR: (126 MHz, Chloroform-*d*) δ 166.7, 166.5, 151.2 (t, J = 4.2 Hz), 144.4, 141.6, 141.4, 140.5, 140.4, 134.4, 132.5, 130.7, 130.1, 129.8, 129.4, 129.1 (t, J = 3.3 Hz), 129.0, 127.8, 127.3, 127.2, 127.1, 118.9 (t, J = 20.7 Hz), 52.28, 52.26.

HRMS (ESI): calc'd for $C_{36}H_{25}F_9KO_4^+$ Exact Mass: 731.1241, found 731.1235.

Dimethyl-4,4'-(*(1Z,3E)*-5,5,6,6,7,7,8,8,8-nonafluoro-1-(3-methoxyphenyl)octa-1,3-diene-2,3-diyl)dibenzoate (39)

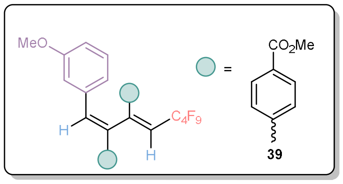

Following the general **procedure D**, **39** (80.1 mg, 0.124 mmol) was obtained as colorless liquid in 62% yield.

R$_f$ = 0.3 (silica gel, petroleum ether/ethyl acetate = 8:1).

^{1}H NMR: (500 MHz, Chloroform-*d*) δ 7.97 – 7.88 (m, 4H), 7.45 – 7.43 (m, 4H), 7.24 (t, J = 7.9 Hz, 1H), 7.08 – 7.02 (m, 3H), 6.85 – 6.82 (m, 1H), 5.99 (t, J = 15.3 Hz, 1H), 3.89 (s, 3H), 3.86 (s, 3H), 3.74 (s, 3H).

^{19}F NMR: (471 MHz, Chloroform-*d*) δ -80.9 (t, J = 9.4 Hz, 3F), -104.0 – -104.1 (m, 2F), -123.4 – -123.5 (m, 2F), -125.7 – -125.8 (m, 2F).

^{13}C NMR: (126 MHz, Chloroform-*d*) δ 166.7, 166.5, 159.7, 151.1 (t, J = 4.5 Hz), 144.3, 141.8, 140.5, 136.8, 132.9, 130.6, 130.0, 129.8, 129.7, 129.3, 129.1 (t, J = 3.1 Hz), 127.3, 121.9, 118.9 (t, J = 20.6 Hz), 114.5, 114.3, 55.1, 52.23, 52.22.

HRMS (EI): calc'd for $C_{31}H_{23}F_9O_5$ Exact Mass: 646.1402, found 646.1395.

Dimethyl-4,4'-(*(1Z,3E)*-5,5,6,6,7,7,8,8,8-nonafluoro-1-(2-methoxyphenyl)octa-1,3-diene-2,3-diyl)dibenzoate (40)

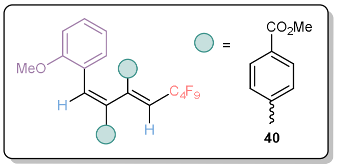

Following the general **procedure D**, **40** (46.5 mg, 0.072 mmol) was obtained as colorless liquid in 36% yield.

R*f* = 0.3 (silica gel, petroleum ether/ethyl acetate = 8:1).

^{1}H NMR: (500 MHz, Chloroform-*d*) δ 7.93 – 7.81 (m, 4H), 7.43 – 7.36 (m, 5H), 7.24 – 7.22 (m, 1H), 7.16 (s, 1H), 6.86 (t, J = 7.5 Hz, 1H), 6.78 (d, J = 8.4 Hz, 1H), 5.87 (t, J = 15.3 Hz, 1H), 3.87 (s, 3H), 3.84 (s, 3H), 3.71 (s, 3H).

^{19}F NMR: (471 MHz, Chloroform-*d*) δ -81.0 (t, J = 9.4 Hz, 3F), -103.7 – -103.8 (m, 2F), -123.5 – -123.6 (m, 2F), -125.7 – -125.8 (m, 2F).

^{13}C NMR: (126 MHz, Chloroform-*d*) δ 166.8, 166.7, 157.3, 151.5 (t, J = 4.8 Hz), 145.1, 142.2, 141.1, 130.2, 129.9, 129.7, 129.5, 129.3 (t, J = 2.5 Hz), 129.0, 127.6, 124.9, 120.5, 118.4 (t, J = 21.3 Hz), 110.7, 55.3, 52.3.

HRMS (EI): calc'd for $C_{31}H_{24}F_9O_5^+$ Exact Mass: 647.1475, found 647.1481.

Dimethyl-4,4'-((2Z,4E)-6,6,7,7,8,8,9,9,9-nonafluoro-1-phenylnona-2,4-diene-3,4-diyl)dibenzoate (41)

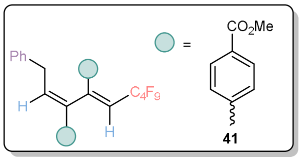

Following the general **procedure D**, **41** (50.4 mg, 0.08 mmol) was obtained as colorless liquid in 40% yield.

R*f* = 0.4 (silica gel, petroleum ether/ethyl acetate = 10:1).

^{1}H NMR:(500 MHz, Chloroform-*d*) δ 7.98 – 7.89 (m, 4H), 7.47 – 7.42 (m, 7H), 7.01 (s, 1H), 6.95 – 6.92 (m, 2H), 6.04 (t, J = 15.1 Hz, 1H), 5.09 (s, 2H), 3.89 (s, 3H), 3.87 (s, 3H).

^{19}F NMR:(471 MHz, Chloroform-*d*) δ -80.8 (t, J = 9.4 Hz, 3F), -103.95 – -104.04 (m, 2F), -123.3 – -123.4 (m, 2F), -125.6 – -125.7 (m, 2F).

^{13}C NMR:(126 MHz, Chloroform-*d*) δ 166.7, 166.5, 159.0, 151.4 (t, J = 4.5 Hz), 144.7, 140.7, 139.6, 136.7, 132.4, 130.9, 130.0, 129.3, 129.0 (t, J = 2.8 Hz), 128.7, 127.5, 127.0, 118.7 (t, J = 20.7 Hz), 115.0, 70.1, 52.3, 52.2.

HRMS (EI): calc'd for $C_{31}H_{23}F_9O_4$ Exact Mass: 630.1453, found 630.1459.

Dimethyl-4,4'-(*(1Z,3E)*-5,5,6,6,7,7,8,8,8-nonafluoro-1-(4-fluorophenyl)octa-1,3-diene-2,3-diyl)dibenzoate (42)

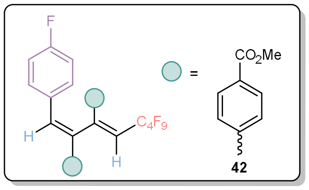

Following the general **procedure D**, **42** (50.7mg, 0.08 mmol) was obtained as colorless liquid in 40% yield.

R$_f$ = 0.4 (silica gel, petroleum ether/ethyl acetate = 10:1).

^{1}H NMR: (500 MHz, Chloroform-*d*) (500 MHz, Chloroform-*d*) δ 7.98 – 7.95 (m, 2H), 7.91 – 7.88 (m, 2H), 7.48 – 7.41 (m, 6H), 7.03 – 6.99 (m, 3H), 5.97 (t, J = 15.1 Hz, 1H), 3.89 (s, 3H), 3.87 (s, 3H).

^{19}F NMR: (471 MHz, Chloroform-*d*) δ -80.9 (t, J = 9.4 Hz, 3F), -104.0 – -104.1 (m, 2F), -112.07 – -112.13 (m, F), -123.4 – -123.5 (m, 2F), -125.7 – -125.8 (m, 2F).

^{13}C NMR: (126 MHz, Chloroform-*d*) δ 166.7, 166.5, 162.7 (d, J = 249.7 Hz), 151.0 (t, J = 4.3 Hz), 144.3, 141.6, 140.4, 131.8, 131.7 (d, J = 3.5 Hz), 131.0 (d, J = 8.3 Hz), 130.7, 130.1, 129.9, 129.4, 129.1 (t, J = 2.8 Hz), 127.2, 119.1 (t, J = 20.8 Hz), 115.8 (d, J = 21.7 Hz), 52.34, 52.31.

HRMS (ESI): calc'd for $C_{30}H_{21}F_{10}O_4^+$ Exact Mass: 635.1275, found 635.1277.

Dimethyl-4,4'-(*(1Z,3E)*-1-(4-chlorophenyl)-5,5,6,6,7,7,8,8,8-nonafluoroocta-1,3-diene-2,3-diyl)dibenzoate (43)

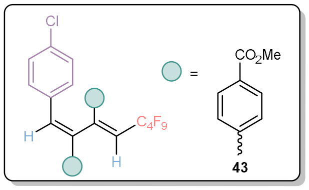

Following the general **procedure D**, **43** (54.6mg, 0.084 mmol) was obtained as colorless liquid in 42% yield.

R$_f$ = 0.4 (silica gel, petroleum ether/ethyl acetate = 10:1).

^{1}H NMR: (500 MHz, Chloroform-*d*) δ 7.98 – 7.89 (m, 4H), 7.45 – 7.41 (m, 6H), 7.31 – 7.28 (m, 2H), 7.00 (s, 1H), 5.98 (t, J = 15.1 Hz, 1H), 3.89 (s, 3H), 3.86 (s, 3H).

^{19}F NMR: (471 MHz, Chloroform-*d*) δ -80.9 (t, J = 9.4 Hz, 3F), -104.15 – -104.24 (m, 2F), -123.4 – -123.5 (m, 2F), -125.7 – -125.8 (m, 2F).

^{13}C NMR: (126 MHz, Chloroform-*d*) δ 166.6, 166.5, 150.9 (t, J = 4.6 Hz), 144.1, 142.3, 140.3, 134.4, 133.9, 131.6, 130.8, 130.5, 130.1, 130.0, 129.4, 129.0 (t, J = 3.2 Hz), 128.9, 127.3, 119.1 (t, J = 21.0 Hz), 52.33, 52.31.

HRMS (EI): calc'd for $C_{30}H_{20}ClF_9O_4$ Exact Mass: 650.0906, found 650.0903.

Dimethyl-4,4'-(*(1Z,3E)*-1-(4-bromophenyl)-5,5,6,6,7,7,8,8,8-nonafluoroocta-1,3-diene-2,3-diyl)dibenzoate (44)

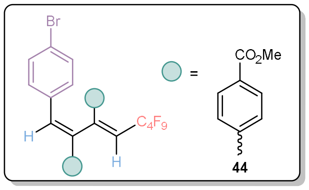

Following the general **procedure D**, **44** (62.5 mg, 0.09 mmol) was obtained as colorless liquid in 45% yield.

R$_f$ = 0.4 (silica gel, petroleum ether/ethyl acetate = 10:1).

^{1}H NMR: (500 MHz, Chloroform-*d*) δ 7.98 – 7.95 (m, 2H), 7.91 – 7.88 (m, 2H), 7.46 – 7.40 (m, 6H), 7.37 – 7.34 (m, 2H), 6.98 (s, 1H), 5.97 (t, J = 15.0 Hz, 1H), 3.89 (s, 3H), 3.87 (s, 3H).

^{19}F NMR: (471 MHz, Chloroform-*d*) δ -80.9 (t, J = 9.4 Hz, 3F), -104.2 – -104.3 (m, 2F), -123.4 – -123.5 (m, 2F), -125.7 – -125.8 (m, 2F).

^{13}C NMR: (126 MHz, Chloroform-*d*) δ 166.6, 166.4, 150.8 (t, J = 3.8 Hz), 144.1, 142.4, 140.2, 134.4, 131.9, 131.6, 130.8, 130.7, 130.1, 130.0, 129.4, 129.0 (t, J = 2.9 Hz), 127.2, 122.6, 119.0 (t, J = 21.2 Hz), 52.34, 52.32.

HRMS (EI): calc'd for $C_{30}H_{20}BrF_9O_4$ Exact Mass: 694.0401, found 694.0410.

Dimethyl-4,4'-(*(1Z,3E)*-5,5,6,6,7,7,8,8,8-nonafluoro-1-(4-iodophenyl)octa-1,3-diene-2,3-diyl)dibenzoate (45)

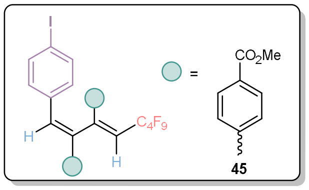

Following the general **procedure D**, **45** (74.2 mg, 0.1 mmol) was obtained as colorless liquid in 50% yield.

R$_f$ = 0.4 (silica gel, petroleum ether/ethyl acetate = 10:1).

^{1}H NMR: (500 MHz, Chloroform-*d*) δ 7.98 – 7.88 (m, 4H), 7.67 – 7.64 (m, 1H), 7.46 – 7.34 (m, 6H), 7.23 – 7.21 (m, 1H), 6.97 (d, J = 10.3 Hz, 1H), 5.97 (t, J = 14.3 Hz, 1H), 3.89 (s, 3H), 3.87 (s, 3H).

^{19}F NMR: (471 MHz, Chloroform-*d*) δ -80.86 – -80.92 (m, 3F), -104.2 – -104.3 (m, 2F), -123.4 – -123.5 (m, 2F), -125.7 – -125.8 (m, 2F).

^{13}C NMR: (126 MHz, Chloroform-*d*) δ 166.6, 166.4, 150.8 (t, J = 3.9 Hz), 144.1, 142.5, 140.2, 137.9, 134.9, 134.4, 131.9, 131.7, 130.8, 130.7, 130.1, 129.4, 129.0 (t, J = 3.1 Hz), 127.2, 119.0 (t, J = 21.1 Hz), 52.34, 52.31.

HRMS (EI): calc'd for $C_{30}H_{20}F_9IO_4$ Exact Mass: 742.0263, found 742.0269.

Dimethyl-4,4'-(*(1Z,3E)*-1-(3,5-dimethylphenyl)-5,5,6,6,7,7,8,8,8-nonafluoroocta-1,3-diene-2,3-diyl)dibenzoate (46)

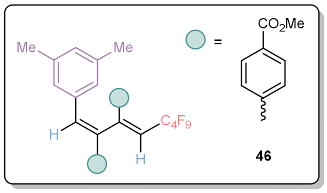

46

Following the general **procedure D**, **46** (91.4 mg, 0.142 mmol) was obtained as colorless liquid in 71% yield.

R$_f$ = 0.4 (silica gel, petroleum ether/ethyl acetate = 10:1).

^{1}H NMR: (500 MHz, Chloroform-*d*) δ 7.97 – 7.89 (m, 4H), 7.44 (d, J = 8.6 Hz, 4H), 7.14 (s, 2H), 7.00 (s, 1H), 6.93 (s, 1H), 6.01 (t, J = 15.2 Hz, 1H), 3.89 (s, 3H), 3.87 (s, 3H), 2.27 (s, 6H).

^{19}F NMR: (471 MHz, Chloroform-*d*) δ -81.0 (t, J = 9.4 Hz, 3F), -104.1 – -104.2 (m, 2F), -123.4 – -123.5 (m, 2F), -125.7 – -125.8 (m, 2F).

^{13}C NMR: (126 MHz, Chloroform-*d*) δ 166.7, 166.6, 151.4 (t, J = 4.3 Hz), 144.6, 141.0, 140.8, 138.2, 135.2, 133.2, 130.5, 130.3, 130.0, 129.6, 129.2, 129.0 (d, J = 3.8 Hz), 127.20, 127.18, 118.8 (t, J = 20.6 Hz), 52.24, 52.22, 21.2.

HRMS (EI): calc'd for $C_{32}H_{25}F_9O_4$ Exact Mass: 644.1609, found 644.1603.

Dimethyl-4,4'-(*(1Z,3E)*-5,5,6,6,7,7,8,8,8-nonafluoro-1-(thiophen-3-yl)octa-1,3-diene-2,3-diyl)dibenzoate (47)

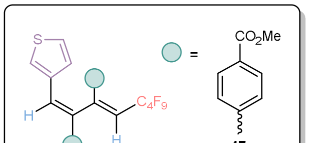

47

Following the general **procedure D**, **47** (37.3 mg, 0.06 mmol) was obtained as colorless liquid in 30% yield.

R$_f$ = 0.3 (silica gel, petroleum ether/ethyl acetate = 10:1).

^{1}H NMR: (500 MHz, Chloroform-*d*) δ 7.96 – 7.89 (m, 4H), 7.49 – 7.41 (m, 5H), 7.35 – 7.30 (m, 2H), 7.06 (s, 1H), 6.09 (t, J = 15.1 Hz, 1H), 3.88 (s, 3H), 3.86 (s, 3H).

^{19}F NMR: (471 MHz, Chloroform-*d*) δ -80.9 (t, J = 9.4 Hz, 3F), -104.2 – -104.3 (m, 2F), -123.2 – -123.3 (m, 2F), -125.6 – -125.7 (m, 2F).

^{13}C NMR: (126 MHz, Chloroform-*d*) δ 166.7, 166.5, 151.5 (t, J = 4.3 Hz), 143.7, 140.3, 139.8, 137.1, 130.7, 130.0, 129.6, 129.4, 128.8 (t, J = 3.1 Hz), 127.5, 126.94, 126.86, 126.3, 126.0, 118.7 (t, J = 20.7 Hz), 52.3, 52.2.

HRMS (EI): calc'd for $C_{28}H_{19}F_9O_4S$ Exact Mass: 622.0860, found 622.0865.

Dimethyl-4,4'-(*(1Z,3E)*-5,5,6,6,7,7,8,8,8-nonafluoro-1-(furan-3-yl)octa-1,3-diene-2,3-diyl)dibenzoate (48)

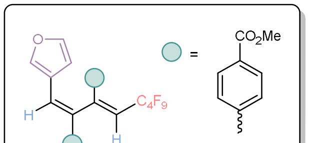

48

Following the general **procedure D**, **48** (36.4 mg, 0.06 mmol) was obtained as colorless liquid in 30% yield.

R*f* = 0.3 (silica gel, petroleum ether/ethyl acetate = 10:1).

^{1}H NMR: (500 MHz, Chloroform-*d*) δ 7.95 – 7.90 (m, 4H), 7.70 – 7.69 (m, 1H), 7.43 – 7.38 (m, 5H), 6.89 (s, 1H), 6.69 (d, J = 1.6 Hz, 1H), 6.12 (t, J = 15.0 Hz, 1H), 3.88 (s, 3H), 3.86 (s, 3H).

^{19}F NMR: (471 MHz, Chloroform-*d*) δ -80.9 (t, J = 9.4 Hz, 3F), -104.3 – -104.4 (m, 2F), -123.15 – -123.24 (m, 2F), -125.6 – -125.7 (m, 2F).

^{13}C NMR: (126 MHz, Chloroform-*d*) δ 166.7, 166.5, 151.4 (t, J = 3.8 Hz), 144.4, 144.0, 143.3, 140.2, 139.7, 130.7, 130.0, 129.6, 129.5, 128.7 (t, J = 2.9 Hz), 126.7, 122.1, 121.4, 118.6 (t, J = 20.7 Hz), 109.4, 52.34, 52.28.

HRMS (EI): calc'd for $C_{28}H_{19}F_9O_5$ Exact Mass: 606.1089, found 606.1098.

Dimethyl-4,4'-(*(1Z,3E)*-1-(4-(9H-carbazol-9-yl)phenyl)-5,5,6,6,7,7,8,8,8-nonafluoroocta-1,3-diene-2,3-diyl)dibenzoate (49)

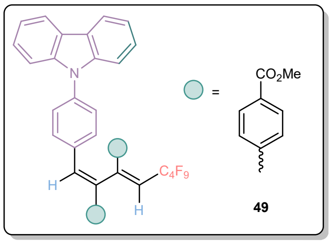

Following the general **procedure D**, **49** (78.2 mg, 0.1 mmol) was obtained as colorless liquid in 50% yield.

R*f* = 0.3 (silica gel, petroleum ether/ethyl acetate = 10:1).

^{1}H NMR: (500 MHz, Chloroform-*d*) δ 8.17 – 8.15 (m, 2H), 8.04 – 7.95 (m, 4H), 7.72 – 7.69 (m, 2H), 7.56 – 7.51 (m, 6H), 7.45 – 7.39 (m, 4H), 7.33 – 7.30 (m, 2H), 7.19 (s, 1H), 6.11 (t, J = 15.0 Hz, 1H), 3.93 (s, 3H), 3.89 (s, 3H).

^{19}F NMR: (471 MHz, Chloroform-*d*) δ -80.8 (t, J = 9.2 Hz, 3F), -103.6 – -103.7 (m, 2F), -123.15 – -123.22 (m, 2F), -125.6 – -125.7 (m, 2F).

^{13}C NMR: (126 MHz, Chloroform-*d*) δ 166.7, 166.4, 151.2 (t, J = 3.8 Hz), 144.3, 142.6, 140.7, 140.4, 137.7, 134.6, 132.3, 130.8, 130.7, 130.1, 130.0, 129.4, 129.3 (t, J = 4.3 Hz), 127.4, 127.1, 126.2, 123.7, 120.5, 120.3, 119.2 (t, J = 19.0 Hz), 109.8, 52.4.

HRMS (ESI): calc'd for $C_{42}H_{29}F_9NO_4^+$ Exact Mass: 782.1947, found 782.1941.

Dimethyl-4,4'-(*(1Z,3E)*-1-(2,3-dihydrobenzofuran-6-yl)-5,5,6,6,7,7,8,8,8-nonafluoroocta-1,3-diene-2,3-diyl)dibenzoate (50)

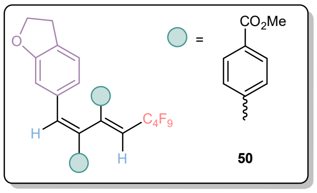

Following the general **procedure D**, **50** (65.8 mg, 0.1 mmol) was obtained as colorless liquid in 50% yield.

R*f* = 0.3 (silica gel, petroleum ether/ethyl acetate = 10:1).

^{1}H NMR: (500 MHz, Chloroform-*d*) δ 7.96 – 7.89 (m, 4H), 7.48 – 7.41 (m, 5H), 7.28 – 7.26 (m, 1H), 7.00 (s, 1H), 6.74 (d, J = 8.3 Hz, 1H), 6.05 (t, J = 15.3 Hz, 1H), 4.58 (t, J = 8.7 Hz, 1H), 3.87 (s, 3H), 3.85 (s, 3H), 3.14 (t, J = 8.6 Hz, 2H).

^{19}F NMR: (471 MHz, Chloroform-*d*) δ -81.0 (t, J = 94.0 Hz, 3F), -103.9 – -104.0 (m, 2F), -123.4 – -123.5 (m, 2F), -125.7 – -125.8 (m, 2F).

^{13}C NMR: (126 MHz, Chloroform-*d*) δ 166.7, 166.5, 160.6, 151.7 (t, J = 4.4 Hz), 144.8, 140.8, 138.9, 132.9, 130.6, 130.0, 129.4, 129.3, 129.1 (t, J = 3.4 Hz), 128.0, 127.7, 127.0, 125.6, 118.6 (t, J = 20.8 Hz), 109.5, 71.7, 52.23, 52.16, 29.4.

HRMS (EI): calc'd for $C_{32}H_{23}F_9O_5$ Exact Mass: 658.1402, found 658.1393.

Dimethyl-4,4'-(*(1Z,3E)*-1-(dibenzo[b,d]furan-4-yl)-5,5,6,6,7,7,8,8,8-nonafluoroocta-1,3-diene-2,3-diyl)dibenzoate (51)

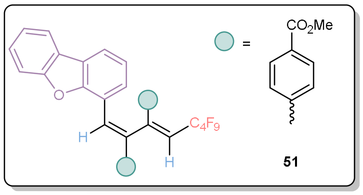

Following the general **procedure D**, **51** (66.4 mg, 0.094 mmol) was obtained as colorless liquid in 47% yield.

R*f* = 0.3 (silica gel, petroleum ether/ethyl acetate = 10:1).

^{1}H NMR: (500 MHz, Chloroform-*d*) δ 8.03 – 8.00 (m, 2H), 7.95 – 7.93 (m, 1H), 7.89 – 7.85 (m, 3H), 7.62 – 7.60 (m, 1H), 7.56 – 7.51 (m, 4H), 7.45 – 7.43 (m, 3H), 7.36 – 7.33 (m, 1H), 7.31 – 7.28 (m, 1H), 6.01 (t, J = 15.1 Hz, 1H), 3.91 (s, 3H), 3.84 (s, 3H).

^{19}F NMR: (471 MHz, Chloroform-*d*) δ -81.0 (t, J = 9.4 Hz, 3F), -103.9 – -104.0 (m, 2F), -123.6 – -123.7 (m, 2F), -125.8 – -125.9 (m, 2F).

^{13}C NMR: (126 MHz, Chloroform-*d*) δ 166.7, 166.5, 156.0, 153.8, 151.5 (t, J = 3.9 Hz), 144.5, 143.8, 140.7, 130.4, 130.0, 129.9, 129.4 (t, J = 2.5 Hz), 129.0, 127.7, 127.6, 127.0, 124.8, 124.0, 123.1, 122.85, 120.94, 120.8, 120.5, 118.5 (t, J = 20.8 Hz), 111.7, 52.3, 52.20.

HRMS (EI): calc'd for $C_{36}H_{23}F_9O_5$ Exact Mass: 706.1402, found 706.1408.

Dimethyl-4,4'-(*(1Z,3E)*-1-(benzo[d][1,3]dioxol-4-yl)-5,5,6,6,7,7,8,8,8-nonafluoroocta-1,3-diene-2,3-diyl)dibenzoate (52)

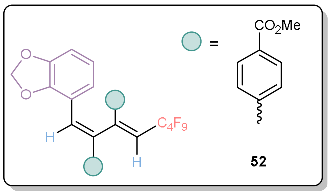

Following the general **procedure D**, **52** (60.7 mg, 0.092 mmol) was obtained as colorless liquid in 46% yield.

R*f* = 0.2 (silica gel, petroleum ether/ethyl acetate = 10:1).

^{1}H NMR: (500 MHz, Chloroform-*d*) δ 7.97 – 7.89 (m, 4H), 7.45 – 7.41 (m, 4H), 7.01 – 6.96 (m, 3H), 6.77 (d, J = 8.0 Hz, 1H), 6.02 (t, J = 15.3 Hz, 1H), 5.94 (s, 2H), 3.88 (s, 3H), 3.85 (s, 3H).

^{19}F NMR: (471 MHz, Chloroform-*d*) δ -80.9 (t, J = 9.4 Hz, 3F), -104.0 – -104.1 (m, 2F), -123.4 – -123.5 (m, 2F), -125.7 – -125.8 (m, 2F).

^{13}C NMR: (126 MHz, Chloroform-*d*) δ 166.7, 166.5, 151.3 (t, J = 3.9 Hz), 148.0, 144.6, 140.6, 140.2, 132.63, 130.60, 130.0, 129.59, 129.57, 129.3, 129.1 (t, J = 2.5 Hz), 127.1, 124.3, 118.9 (t, J = 20.8 Hz), 108.8, 108.5, 101.5, 52.24, 52.20.

HRMS (EI): calc'd for $C_{31}H_{21}F_9O_6$ Exact Mass: 660.1194, found 660.1200.

Dimethyl-4,4'-(*(1Z,3E)*-1-(2,3-dihydrobenzo[b][1,4]dioxin-5-yl)-5,5,6,6,7,7,8,8,8-nonafluoroocta-1,3-diene-2,3-diyl)dibenzoate (53)

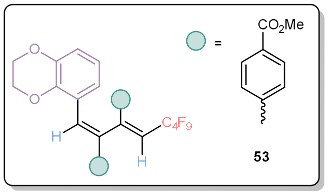

Following the general **procedure D**, **53** (76.8 mg, 0.114 mmol) was obtained as colorless liquid in 57% yield.

R*f* = 0.15 (silica gel, petroleum ether/ethyl acetate = 10:1).

^{1}H NMR: (500 MHz, Chloroform-*d*) δ 7.96 – 7.89 (m, 4H), 7.46 – 7.41 (m, 4H), 7.04 – 6.99 (m, 2H), 6.93 (s, 1H), 6.80 (d, J = 8.4 Hz, 1H), 6.02 (t, J = 15.2 Hz, 1H), 4.26 – 4.21 (m, 4H), 3.88 (s, 3H), 3.85 (s, 3H).

^{19}F NMR: (471 MHz, Chloroform-*d*) δ -80.9 (t, J = 9.4 Hz, 3F), -104.0 – -104.1 (m, 2F), -123.4 – -123.5 (m, 2F), -125.7 – -125.8 (m, 2F).

^{13}C NMR: (126 MHz, Chloroform-*d*) δ 166.7, 166.5, 151.3 (t, J = 4.7 Hz), 144.7, 144.1, 143.5, 140.7, 140.1, 132.4, 130.5, 130.0, 129.5, 129.2, 129.1 (t, J = 2.9 Hz), 128.9, 127.1, 123.0, 118.8 (t, J = 20.8 Hz), 118.2, 117.4, 64.6, 64.3, 52.23, 52.20.

HRMS (EI): calc'd for $C_{32}H_{23}F_9O_6$ Exact Mass: 674.1351, found 674.1359.

Dimethyl-4,4'-(*(1Z,3E)*-5,5,6,6,7,7,7-heptafluoro-1-(4-(methylthio)phenyl)hepta-1,3-diene-2,3-diyl)dibenzoate (54)

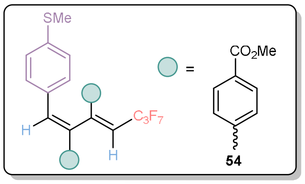

Following the general **procedure D**, **54** (79.5 mg, 0.13 mmol) was obtained as colorless liquid in 65% yield.

R*f* = 0.3 (silica gel, petroleum ether/ethyl acetate = 10:1).

^{1}H NMR: (500 MHz, Chloroform-*d*) δ 7.97 – 7.89 (m, 4H), 7.47 – 7.42 (m, 6H), 7.20 – 7.17 (m, 2H), 7.00 (s, 1H), 6.02 (t, J = 15.2 Hz, 1H), 3.88 (s, 3H), 3.85 (s, 3H), 2.47 (s, 3H).

^{19}F NMR: (471 MHz, Chloroform-*d*) δ -80.1 (t, J = 9.3 Hz, 3F), -104.6 – -104.7 (m, 2F), -126.9 (s, 2F).

^{13}C NMR: (126 MHz, Chloroform-*d*) δ 166.6, 166.4, 151.3 (t, J = 4.2 Hz), 144.3, 140.8, 140.4, 139.7, 132.2, 131.9, 130.7, 130.0, 129.7, 129.3, 129.0 (t, J = 3.5 Hz), 127.1, 126.2, 118.6 (t, J = 20.6 Hz), 52.3, 52.2, 15.5.

HRMS (EI): calc'd for $C_{30}H_{23}F_7O_4S$ Exact Mass: 612.1205, found 612.1209.

Dimethyl-4,4'-(*(1Z,3E)*-5,6,6,6-tetrafluoro-1-(4-(methylthio)phenyl)-5-(trifluoromethyl)hexa-1,3-diene-2,3-diyl)dibenzoate (55)

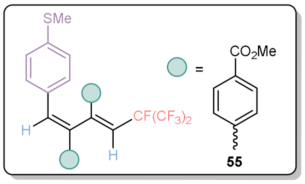

Following the general **procedure D**, **55** (79.5 mg, 0.13 mmol) was obtained as colorless liquid in 65% yield.

R*f* = 0.3 (silica gel, petroleum ether/ethyl acetate = 10:1).

^{1}H NMR: (500 MHz, Chloroform-*d*) δ 7.99 – 7.96 (m, 2H), 7.85 – 7.82 (m, 2H), 7.46 – 7.42 (m, 4H), 7.33 – 7.30 (m, 2H), 7.22 – 7.19 (m, 2H), 6.90 (s, 1H), 5.85 (d, J = 28.0 Hz, 1H), 3.89 (s, 3H), 3.85 (s, 3H), 2.50 (s, 3H).

^{19}F NMR: (471 MHz, Chloroform-*d*) δ -75.63 (s, 3F), -75.65 (s, 3F), -183.4 – -183.5 (m, 1F).

^{13}C NMR: (126 MHz, Chloroform-*d*) δ 166.7, 166.6, 149.8, 144.7, 141.7, 140.7 (t, J = 1.8 Hz), 139.5, 132.1, 131.6, 130.1, 130.0, 129.7, 129.6, 128.9, 128.7 (t, J = 4.6 Hz), 127.3, 126.3, 116.3 (t, J = 14.0 Hz), 52.27, 52.25, 15.6.

HRMS (EI): calc'd for $C_{30}H_{23}F_7O_4S$ Exact Mass: 612.1205, found 612.1196.

Dimethyl-4,4'-(*(1Z,3E)*-5,5,6,6,7,7,8,8,9,9,10,10,10-tridecafluoro-1-(4-(methylthio)phenyl)deca-1,3-diene-2,3-diyl)dibenzoate (56)

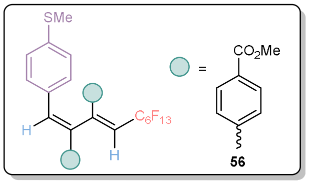

Following the general **procedure D**, **56** (94.5 mg, 0.124 mmol) was obtained as colorless liquid in 62% yield.

R*f* = 0.3 (silica gel, petroleum ether/ethyl acetate = 10:1).

^{1}H NMR: (500 MHz, Chloroform-*d*) δ 7.97 – 7.88 (m, 4H), 7.45 – 7.42 (m, 6H), 7.20 – 7.17 (m, 2H), 7.00 (s, 1H), 6.01 (t, J = 15.1 Hz, 1H), 3.89 (s, 3H), 3.86 (s, 3H), 2.48 (s, 3H).

^{19}F NMR: (471 MHz, Chloroform-*d*)) δ -80.7 (t, J = 9.4 Hz, 3F), -103.9 – -104.0 (m, 2F), -121.5 – -121.6 (m, 2F), -122.36 – -122.44 (m, 2F), -122.7 – -122.8 (m, 2F), -126.0 – -126.1 (m, 2F).

^{13}C NMR: (126 MHz, Chloroform-*d*) δ 166.7, 166.5, 151.2 (t, J = 4.6 Hz), 144.4, 140.9, 140.5, 139.8, 132.2, 132.0, 130.7, 130.0, 129.7, 129.4, 129.1 (t, J = 2.5 Hz), 127.1, 126.2, 118.9 (t, J = 20.8 Hz), 52.31, 52.28, 15.5.

HRMS (EI): calc'd for $C_{33}H_{23}F_{13}O_4S$ Exact Mass: 762.1109, found 762.1101.

Dimethyl-4,4'-(*(1Z,3E)*-5,5,6,6,7,7,8,8-octafluoro-8-iodo-1-(4-(methylthio)phenyl)octa-1,3-diene-2,3-diyl)dibenzoate (57)

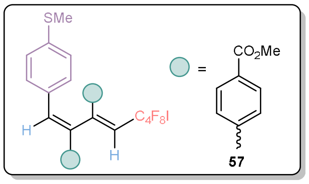

Following the general **procedure D**, **57** (77 mg, 0.1 mmol) was obtained as colorless liquid in 50% yield.

R*f* = 0.3 (silica gel, petroleum ether/ethyl acetate = 10:1).

^{1}H NMR:(500 MHz, Chloroform-*d*) δ 7.96 – 7.88 (m, 4H), 7.45 – 7.42 (m, 6H), 7.18 (d, J = 8.3 Hz, 2H), 6.99 (s, 1H), 6.00 (t, J = 15.1 Hz, 1H), 3.89 (s, 3H), 3.86 (s, 3H), 2.48 (s, 3H).

^{19}F NMR:(471 MHz, Chloroform-*d*) δ -58.5 (t, J = 14.4 Hz, 2F), -103.9 – -104.0 (m, 2F), -112.38 – -112.44 (m, 2F), -121.6 – -121.7 (m, 2F).

^{13}C NMR:(126 MHz, Chloroform-*d*) δ 166.7, 166.5, 150.8 (t, J = 4.1 Hz), 144.4, 140.9, 140.5, 139.6, 132.1, 132.0, 130.6, 130.0, 129.7, 129.6, 129.3, 129.1 (t, J = 3.1 Hz), 127.1, 126.2, 119.1 (t, J = 21.1 Hz), 52.30, 52.27, 15.5.

HRMS (EI): calc'd for $C_{31}H_{23}F_8IO_4S$ Exact Mass: 770.0234, found 770.0229.

Dimethyl-4,4'-(*(1Z,3E)*-5,5,6,6,7,7,8,8,9,9,10,10,11,11,12,12,12-heptadecafluoro-1-(4-(methylthio)phenyl)dodeca-1,3-diene-2,3-diyl)dibenzoate (58)

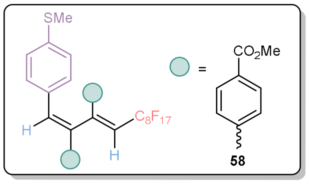

Following the general **procedure D**, **58** (101.7 mg, 0.118 mmol) was obtained as colorless solid in 59% yield.

R*f* = 0.3 (silica gel, petroleum ether/ethyl acetate = 10:1).

mp: 103 – 105 ^{o}C.

^{1}H NMR: (500 MHz, Chloroform-*d*) δ 7.97 – 7.88 (m, 4H), 7.45 – 7.42 (m, 6H), 7.20 – 7.17 (m, 2H), 6.99 (s, 1H), 6.01 (t, J = 15.1 Hz, 1H), 3.89 (s, 3H), 3.86 (s, 3H), 2.48 (s, 3H).

^{19}F NMR: (471 MHz, Chloroform-*d*) δ -80.8 (t, J = 9.4 Hz, 3F), -103.9 – -104.0 (m, 2F), -121.2 – -121.5 (m, 2F), -121.8 – -121.9 (m, 4F), -122.4 – -122.5 (m, 2F), -122.6 – -122.8 (m, 2F), -126.1 – -126.2 (m, 2F).

^{13}C NMR: (126 MHz, Chloroform-*d*) δ 166.7, 166.5, 151.2 (t, J = 3.7 Hz), 144.4, 140.9, 140.5, 139.8, 132.2, 132.0, 130.7, 130.0, 129.7, 129.3, 129.0 (t, J = 3.4 Hz), 127.1, 126.2, 118.9 (t, J = 20.8 Hz), 52.21, 52.18, 15.4.

HRMS (EI): calc'd for $C_{35}H_{23}F_{17}O_4S$ Exact Mass: 862.1046, found 862.1054.

Dimethyl-4,4'-(*(1Z,3E)*-5,5,6,6,7,7,8,8,9,9,10,10,11,11,12,12,13,13,14,14,14-henicosafluoro-1-(4-(methylthio)phenyl)tetradeca-1,3-diene-2,3-diyl)dibenzoate (59)

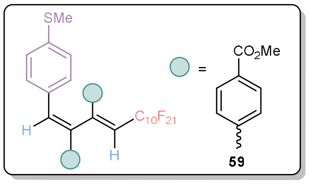

Following the general **procedure D**, **59** (107.7 mg, 0.112 mmol) was obtained as colorless solid in 56% yield.

R*f* = 0.3 (silica gel, petroleum ether/ethyl acetate = 10:1).

mp: 85 – 87 ^{o}C.

^{1}H NMR: (500 MHz, Chloroform-*d*) δ 7.97 – 7.88 (m, 4H), 7.45 – 7.42 (m, 6H), 7.20 – 7.17 (m, 2H), 6.99 (s, 1H), 6.01 (t, J = 15.1 Hz, 1H), 3.89 (s, 3H), 3.86 (s, 3H), 2.48 (s, 3H).

^{19}F NMR: (471 MHz, Chloroform-*d*) δ -80.7 (t, J = 9.1 Hz, 3F), -103.9 – -104.0 (m, 2F), -121.3 – -121.4 (m, 2F), -121.5 – -121.8 (m, 8F), -122.3 – -122.4 (m, 2F), -122.6 – -122.7 (m, 2F), -126.0 – -126.1 (m, 2F).

^{13}C NMR: (126 MHz, Chloroform-*d*) δ 166.7, 166.5, 151.2 (t, J = 4.5 Hz), 144.4, 140.9, 140.5, 139.8, 132.2, 132.0, 130.7, 130.0, 129.7, 129.3, 129.1 (t, J = 2.8 Hz), 127.1, 126.2, 118.9 (t, J = 21.1 Hz), 52.23, 52.20, 15.3.

HRMS (EI): calc'd for $C_{37}H_{23}F_{21}O_4S$ Exact Mass: 962.0982, found 962.0989.

bis(1,7,7-trimethylbicyclo[2.2.1]heptan-2-yl)4,4'-(*(1Z,3E)*-5,5,6,6,7,7,8,8,8-nonafluoro-1-(4-methoxyphenyl)octa-1,3-diene-2,3-diyl)dibenzoate (60)

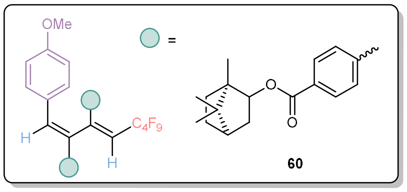

60

Following the general **procedure D**, **60** (106.8 mg, 0.12 mmol) was obtained as colorless liquid in 60% yield.

R*f* = 0.3 (silica gel, petroleum ether/ethyl acetate = 10:1).

^{1}H NMR: (500 MHz, Chloroform-*d*) δ 7.99 – 7.94 (m, 4H), 7.51 (d, J = 8.4 Hz, 4H), 7.46 (d, J = 8.4 Hz, 2H), 7.01 (s, 1H), 6.88 – 6.85 (m, 2H), 6.04 (t, J = 15.3 Hz, 1H), 5.10 – 5.05 (m, 2H), 3.83 (s, 3H), 2.49 – 2.41 (m, 2H), 2.13 – 2.05 (m, 2H), 1.84 – 1.71 (m, 4H), 1.43 – 1.25 (m, 4H), 1.11 – 1.05 (m, 2H), 0.96 (s, 3H), 0.94 (s, 3H), 0.91 (s, 3H), 0.90 (s, 6H), 0.88 (s, 3H).

^{19}F NMR: (471 MHz, Chloroform-*d*) δ -80.9 (t, J = 9.4 Hz, 3F), -104.0 – -104.1 (m, 2F), -123.3 – -123.4 (m, 2F), -125.6 – -125.7 (m, 2F).

^{13}C NMR: (126 MHz, Chloroform-*d*) δ 166.5, 166.3, 159.9, 151.4 (t, J = 3.9 Hz), 144.3, 140.4, 139.5, 132.1, 131.4, 130.9, 130.2, 130.0, 129.4, 129.1, 126.9, 118.5 (t, J = 22.2 Hz), 114.2, 81.0, 80.9, 80.87, 80.81, 80.77, 80.7, 55.5, 49.2, 48.0, 45.1, 37.02, 36.98, 28.20, 28.17, 27.52, 27.47, 19.84, 19.82, 19.03, 19.01, 13.74, 13.72.

HRMS (ESI): calc'd for $C_{49}H_{52}F_9O_5^+$ Exact Mass:891.3666, found 891.3663.

(3*r*,5*r*,7*r*)-adamantan-1-yl)methyl-3-(*(1Z,3E)*-2-(4-((((3*r*,5*r*,7*r*)-adamantan-1-yl)methoxycarbonyl)phenyl)-5,5,6,6,7,7,8,8,8-nonafluoro-1-(4-methoxyphenyl)octa-1,3-dien-3-yl)benzoate (61)

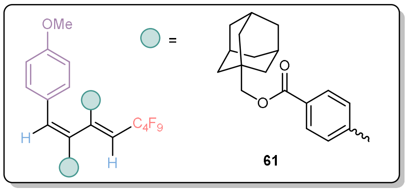

61

Following the general **procedure D**, **61** (118.9 mg, 0.13 mmol) was obtained as colorless liquid in 65% yield.

R*f* = 0.3 (silica gel, petroleum ether/ethyl acetate = 10:1).

^{1}H NMR: (500 MHz, Chloroform-*d*) δ 7.99 – 7.93 (m, 4H), 7.51 (d, J = 8.5 Hz, 4H), 7.45 (d, J = 6.8 Hz, 2H), 7.01 (s, 1H), 6.88 – 6.85 (m, 2H), 6.04 (t, J = 15.2 Hz, 1H), 3.89 (s, 2H), 3.87 (s, 2H), 3.82 (s, 3H), 2.02 – 1.99 (m, 6H), 1.77 – 1.66 (m, 12H), 1.63 – 1.60 (m, 12H).

^{19}F NMR: (471 MHz, Chloroform-*d*) δ -80.9 (t, J = 10.1 Hz, 3F), -104.0 – -104.1 (m, 2F), -123.3 – -123.4 (m, 2F), -125.6 – -125.7 (m, 2F).

^{13}C NMR: (126 MHz, Chloroform-*d*) δ 166.4, 166.1, 159.9, 151.4 (t, J = 3.3 Hz), 144.4, 140.5, 139.5, 132.2, 131.1,

130.9, 130.0, 129.9, 129.3, 129.1 (t, J = 3.2 Hz), 128.0, 126.9, 118.5 (t, J = 20.7 Hz), 114.2, 74.8, 74.6, 55.4, 39.53, 39.50, 37.08, 37.06, 33.6, 33.5, 28.17, 28.15.

HRMS (ESI): calc'd for $C_{51}H_{52}F_9O_5^+$ Exact Mass: 915.3666, found 915.3665.

bis(((3*aR*,*5R*,5*aS*,8*aS*,8*bR*)-2,2,7,7-tetramethyltetrahydro-5H-bis([1,3]dioxolo)[4,5-b:4',5'-d]pyran-5-yl)methyl)-4,4'-(*(1Z,3E)*-5,5,6,6,7,7,8,8,8-nonafluoro-1-(4-methoxyphenyl)octa-1,3-diene-2,3-diyl)dibenzoate (62)

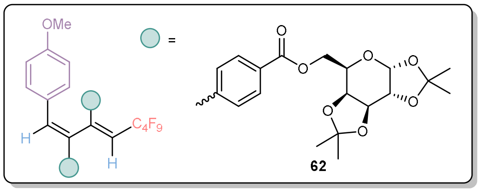

Following the general **procedure D**, **62** (110 mg, 0.1 mmol) was obtained as colorless liquid in 50% yield.

R*f* = 0.3 (silica gel, petroleum ether/ethyl acetate = 3:1).

^{1}H NMR: (500 MHz, Chloroform-*d*) δ 7.96 (d, J = 8.2 Hz, 2H), 7.91 (d, J = 8.2 Hz, 2H), 7.48 – 7.41 (m, 6H), 6.99 (s, 1H), 6.85 (d, J = 8.9 Hz, 2H), 6.01 (t, J = 15.2 Hz, 1H), 5.54 (dd, J = 5.0, 7.5 Hz, 2H), 4.65 – 4.62 (m, 2H), 4.50 – 4.28 (m, 8H), 4.17 – 4.11 (m, 2H), 3.80 (s, 3H), 1.50 (s, 3H), 1.49 (s, 3H), 1.46 (s, 3H), 1.44 (s, 3H), 1.34 (s, 3H), 1.33 (s, 3H), 1.32 (s, 3H), 1.31 (s, 3H).

^{19}F NMR: (471 MHz, Chloroform-*d*) δ -80.9 (t, J = 9.1 Hz, 3F), -104.0 – -104.1 (m, 2F), -123.3 – -123.4 (m, 2F), -125.6 – -125.7 (m, 2F).

^{13}C NMR: (126 MHz, Chloroform-*d*) δ 166.0, 165.8, 159.9, 151.4 (t, J = 3.8 Hz), 144.7, 140.7, 139.5, 132.3, 130.8, 130.5, 130.1, 129.5, 129.4, 129.0 (t, J = 2.5 Hz), 128.0, 127.0, 118.6 (t, J = 21.4 Hz), 114.1, 109.8, 108.9, 96.42, 96.41, 71.21, 71.17, 70.8, 70.61, 70.58, 66.2, 66.1, 64.04, 63.99, 55.4, 26.11, 26.05, 25.04, 24.6.

HRMS (ESI): calc'd for $C_{53}H_{56}F_9O_{15}^+$ Exact Mass: 1103.3470, found 1103.3474.

bis(5-(2,5-dimethylphenoxy)-2,2-dimethylpentyl)-4,4'-(*(1Z,3E)*-5,5,6,6,7,7,8,8,8-nonafluoro-1-(4-methoxyphenyl)octa-1,3-diene-2,3-diyl)dibenzoate (63)

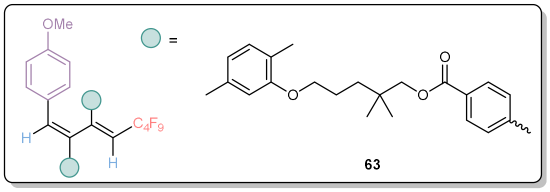

Following the general **procedure D**, **63** (75.9 mg, 0.072 mmol) was obtained as colorless liquid in 36% yield.

R*f* = 0.2 (silica gel, petroleum ether/ethyl acetate = 10:1).

^{1}H NMR: (500 MHz, Chloroform-*d*) δ 8.02 – 7.95 (m, 4H), 7.55 – 7.52 (m, 4H), 7.49 – 7.47 (m, 2H), 7.04 (s, 1H),

7.00 (dd, J = 7.4, 11.4 Hz, 2H), 6.91 – 6.88 (m, 2H), 6.68 – 6.63 (m, 4H), 6.07 (t, J = 15.2 Hz, 1H), 4.09 (s, 2H), 4.06 (s, 2H), 3.97 – 3.93 (m, 4H), 3.84 (s, 3H), 2.32 (s, 3H), 2.32 (s, 3H), 2.17 (s, 3H), 2.13 (s, 3H), 1.87 – 1.79 (m, 4H), 1.60 – 1.54 (m, 4H), 1.07 (s, 6H), 1.05 (s, 6H).

^{19}F NMR: (471 MHz, Chloroform-*d*) δ -80.9 (t, J = 9.4 Hz, 3F), -104.0 – -104.1 (m, 2F), -123.3 – -123.4 (m, 2F), -125.6 – -125.7 (m, 2F).

^{13}C NMR: (126 MHz, Chloroform-*d*) δ 166.2, 166.0, 159.9, 157.10, 157.07, 144.5, 140.6, 139.4, 136.57, 136.55, 132.3, 131.0, 130.9, 130.4, 130.0, 129.8, 129.4, 129.1 (t, J = 3.3 Hz), 128.0, 126.9, 123.6, 120.79, 120.77, 118.5 (t, J = 21.0 Hz), 114.2, 112.02, 111.99, 72.92, 72.85, 68.35, 68.29, 55.4, 35.7, 35.6, 34.01, 33.97, 24.52, 24.49, 24.28, 24.25, 21.5, 15.84, 15.79.

HRMS (ESI): calc'd for $C_{59}H_{64}F_9O_7^+$ Exact Mass: 1055.4503, found 1055.4507.

bis((1*R*,2*R*,5*R*)-2-isopropyl-5-methylcyclohexyl)-4,4'-(*(1Z,3E)*-5,5,6,6,7,7,8,8,8-nonafluoro-1-(4-methoxyphenyl)octa-1,3-diene-2,3-diyl)dibenzoate (64)

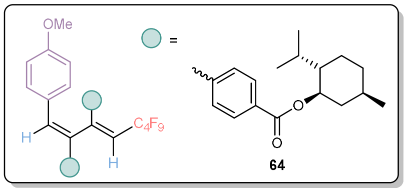

Following the general **procedure D**, **64** (116 mg, 0.13 mmol) was obtained as colorless liquid in 65% yield.

R*f* = 0.3 (silica gel, petroleum ether/ethyl acetate = 10:1).

^{1}H NMR: (500 MHz, Chloroform-*d*) δ 7.95 (dd, J = 8.0, 20.6 Hz, 4H), 7.52 – 7.49 (m, 4H), 7.45 (d, J = 8.3 Hz, 2H), 7.01 (s, 1H), 6.88 – 6.85 (m, 2H), 6.04 (t, J = 15.3 Hz, 1H), 4.94 – 4.87 (m, 2H), 3.82 (s, 3H), 2.13 – 2.06 (m, 2H), 1.99 – 1.89 (m, 2H), 1.75 – 1.69 (m, 4H), 1.57 – 1.51 (m, 4H), 1.11 – 1.04 (m, 4H), 0.93 – 0.90 (m, 14H), 0.78 (t, J = 7.2 Hz, 6H).

^{19}F NMR: (471 MHz, Chloroform-*d*) δ -80.9 (t, J = 9.4 Hz, 3F), -104.0 (d, J = 78.9 Hz, 2F), -123.35 – -123.41 (m, 2F), -125.6 – -125.7 (m, 2F).

^{13}C NMR: (126 MHz, Chloroform-*d*) δ 165.8, 165.6, 159.9, 151.4 (t, J = 3.9 Hz), 144.3, 140.3, 139.5, 132.1 (t, J = 6.3 Hz), 131.4, 130.90, 130.86, 130.2, 130.0, 129.4, 129.0 (t, J = 3.8 Hz), 128.0, 126.8, 118.4 (t, J = 20.3 Hz), 114.2, 75.1, 55.4, 47.4, 41.1, 41.0, 34.4, 31.6, 26.6, 26.4, 23.7, 23.6, 22.14, 22.12, 20.91, 20.89, 16.6, 16.5.

HRMS (ESI): calc'd for $C_{49}H_{56}F_9O_5^+$ Exact Mass: 895.3979, found 895.3982.

bis(2-(4-isobutylphenyl)propyl)-4,4'-(*(1Z,3E)*-5,5,6,6,7,7,8,8,8-nonafluoro-1-(4-methoxyphenyl)octa-1,3-diene-2,3-diyl)dibenzoate (65)

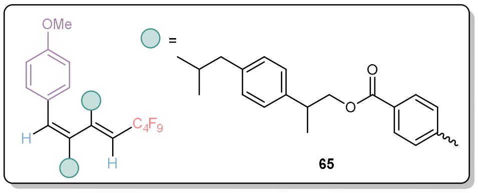

Following the general **procedure D**, **65** (119.8 mg, 0.124 mmol) was obtained as colorless liquid in 62% yield.

R$_f$ = 0.3 (silica gel, petroleum ether/ethyl acetate = 10:1).

^{1}H NMR: (500 MHz, Chloroform-*d*) δ 7.93 – 7.86 (m, 4H), 7.52 – 7.43 (m, 6H), 7.22 – 7.11 (m, 8H), 7.01 (s, 1H), 6.88 (d, J = 8.7 Hz, 2H), 6.05 (t, 1H), 4.43 – 4.31 (m, 4H), 3.84 (s, 3H), 3.25 – 3.18 (m, 2H), 2.48 (dd, J = 4.3, 6.9 Hz, 4H), 1.92 – 1.82 (m, 2H), 1.39 (dd, J = 7.3, 8.9 Hz, 6H), 0.94 – 0.89 (m, 12H).

^{19}F NMR: (471 MHz, Chloroform-*d*) δ -80.9 (t, J = 9.4 Hz, 3F), -103.95 – -104.04 (m, 2F), -123.3 – -123.4 (m, 2F), -125.6 – -125.7 (m, 2F).

^{13}C NMR: (126 MHz, Chloroform-*d*) δ 166.2, 165.9, 159.9, 151.4 (t, J = 4.8 Hz), 144.5, 140.6, 140.4, 140.3, 140.2, 139.5, 132.3, 130.9, 130.0, 129.7, 129.4, 129.3, 129.0 (t, J = 3.1 Hz), 128.0, 127.14, 127.11, 126.9, 118.6 (t, J = 21.6 Hz), 114.2, 70.3, 70.2, 55.4, 45.1, 38.8, 38.7, 30.3, 22.49, 22.48, 22.46, 22.45, 18.2.

HRMS (ESI): calc'd for $C_{55}H_{56}F_9O_5^+$ Exact Mass: 967.3979, found 967.3973.

bis((3*S*,8*S*,9*S*,10*R*,13*R*,14*S*,17*R*)-10,13-dimethyl-17-((*R*)-6-methylheptan-2-yl)-2,3,4,7,8,9,10,11,12,13,14,15,16,17-tetradecahydro-1H-cyclopenta[a]phenanthren-3-yl)-4,4'-(*(1Z,3E)*-5,5,6,6,7,7,8,8,8-nonafluoro-1-(4-methoxyphenyl)octa-1,3-diene-2,3-diyl)dibenzoate (66)

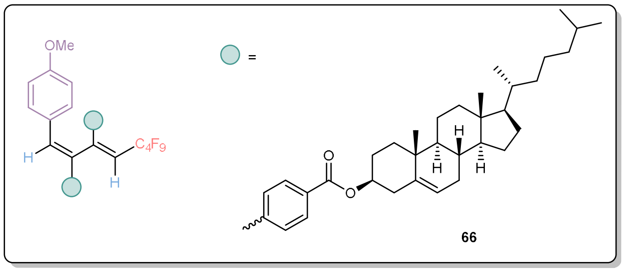

Following the general **procedure D**, **66** (178.7 mg, 0.13 mmol) was obtained as colorless liquid in 66% yield.

R$_f$ = 0.3 (silica gel, petroleum ether/ethyl acetate = 10:1).

^{1}H NMR: (500 MHz, Chloroform-*d*) δ 7.93 (dd, J = 8.5, 20.4 Hz, 4H), 7.47 (dd, J = 8.6, 16.7 Hz, 4H), 7.42 (d, J = 8.5 Hz, 2H), 6.99 (s, 1H), 6.87 – 6.84 (m, 2H), 6.02 (t, J = 15.2 Hz, 1H), 5.41 – 5.39 (m, 2H), 4.86 – 4.77 (m, 2H), 3.82 (s, 3H), 2.42 (dd, J = 8.1, 13.2 Hz, 4H), 2.04 – 1.80 (m, 11H), 1.75 – 1.64 (m, 2H), 1.61 – 1.35 (m, 18H), 1.24 – 1.11 (m, 15H), 1.06 (s, 3H), 1.04 (s, 3H), 1.02 – 0.96 (m, 6H), 0.92 (dd, J = 2.0, 6.6 Hz, 6H), 0.90 – 0.83 (m, 12H), 0.69 (s, 3H), 0.68 (s, 3H).

^{19}F NMR: (471 MHz, Chloroform-*d*) δ -80.9 (t, J = 9.4 Hz, 3F), -104.1 (s, 2F), -123.3 – -123.4 (m, 2F), -125.6 – -125.7 (m, 2F).

^{13}**C NMR:** (126 MHz, Chloroform-*d*) δ 165.7, 165.4, 159.9, 151.5 (t, J = 4.5 Hz), 144.4, 140.5, 139.74, 139.67, 139.58, 132.2, 131.3, 130.9, 130.2, 130.0, 129.3, 129.0 (t, J = 2.5 Hz), 128.1, 126.9, 122.99, 122.96, 118.5 (t, J = 20.6 Hz), 114.2, 74.9, 74.8, 56.8, 56.3, 55.5, 50.2, 42.5, 39.9, 39.7, 38.33, 38.29, 37.15, 37.12, 36.77, 36.76, 36.3, 35.9, 32.1, 32.0, 28.4, 28.2, 28.00, 27.95, 24.4, 24.0, 23.0, 22.7, 21.2, 19.50, 19.47, 18.9, 12.0.

HRMS (ESI): calc'd for $C_{83}H_{108}F_9O_5^+$ Exact Mass: 1355.8048, found 1355.8056.

6. Synthesis and characterization of applications

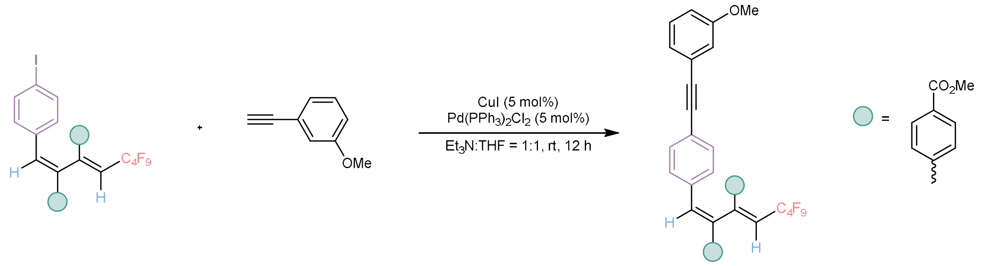

45

67

Supplementary Fig 11. Synthetic methods for Sonogashira cross-coupling reaction

To an oven-dried 10 mL Young's Tube vial equipped with a magnetic stir bar was added **45** (0.05 mmol, 1.0 equiv), 3-ethynylanisole (0.15 mmol, 3.0 equiv), $PdCl_2(PPh_3)_2$ (10 mol%, 0.7 mg), CuI (5 mol%, 0.5 mg), then Et_3N (0.5 mL) and THF (0.5 mL) were added. The resulting reaction mixture was stirring at room temperature for 12 h. After the reaction was completed, the reaction mixture was extracted with EtOAc and the combined organic layers were dried over Na_2SO_4, filtered and concentrated under reduced pressure. The product was purified by column chromatography over silica gel (petroleum ether/ethyl acetate = 20:1-10:1) to afford compound **67** (33.6 mg, 0.045 mmol) as yellow liquid in 90% yield.

Dimethyl-4,4'-((1*Z*,3*E*)-5,5,6,6,7,7,8,8,8-nonafluoro-1-(4-((3-methoxyphenyl)ethynyl)phenyl) octa-1,3-diene-2,3-diyl)dibenzoate (67)

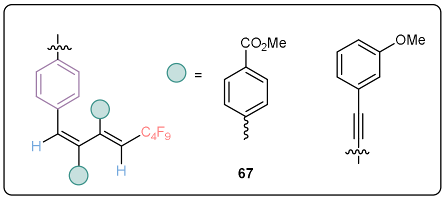

67

R*f* = 0.3 (silica gel, petroleum ether/ethyl acetate = 10:1).

^{1}**H NMR:** (500 MHz, Chloroform-*d*) δ 7.97 – 7.87 (m, 4H), 7.47 – 7.41 (m, 8H), 7.24 – 7.23 (m, 1H), 7.13 – 7.11 (m, 1H), 7.05 – 7.04 (m, 1H), 7.01 (s, 1H), 6.90 – 6.88 (m, 1H), 6.00 (t, J = 15.1 Hz, 1H), 3.88 (s, 3H), 3.85 (s, 3H), 3.81 (s, 3H).

^{19}**F NMR:** (471 MHz, Chloroform-*d*) δ -80.9 (t, J = 10.4 Hz, 3F), -104.2 – -104.3 (m, 2F), -123.3 – -123.4 (m, 2F), -125.6 – -125.7 (m, 2F).

^{13}**C NMR:** (126 MHz, Chloroform-*d*) δ 166.6, 166.5, 159.5, 151.0 (t, J = 4.2 Hz), 144.2, 142.2, 140.3, 135.3, 132.1, 131.9, 130.7, 130.1, 129.6, 129.4, 129.3, 129.0 (t, J = 3.1 Hz), 127.2, 124.3, 124.1, 123.4, 119.0 (t, J = 20.1 Hz), 116.5, 115.3, 91.0, 89.0, 77.4, 77.2, 76.9, 55.4, 52.30, 52.28.

HRMS (EI): calc'd for $C_{39}H_{28}F_9O_5^+$ Exact Mass: 747.1788, found 747.1783.

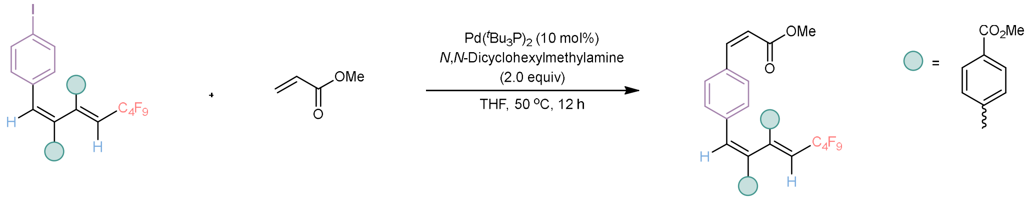

45 **68**

Supplementary Fig 12. Synthetic methods for Heck cross-coupling reaction

To an oven-dried 10 mL Young's Tube vial equipped with a magnetic stir bar was added **45** (0.05 mmol, 1.0 equiv), Pd(^{t}Bu$_3$P)$_2$ (10 mol%, 2.6 mg), and *N*, *N*-dicyclohexylmethylamine (0.1 mmol, 2.0 equiv), methyl acrylate (0.25 mmol, 5.0 equiv), then THF (1 mL) was added. The resulting reaction mixture was stirring at 50 °C for 12 h. After the reaction was completed, the reaction mixture was filtered and concentrated under reduced pressure. The product was purified by column chromatography over silica gel (petroleum ether/ethyl acetate = 15:1-8:1) to afford compound **68** (33.6 mg, 0.048 mmol) as yellow liquid in 96% yield.

Dimethyl-4,4'-((1*Z*,3*E*)-5,5,6,6,7,7,8,8,8-nonafluoro-1-(4-((*E*)-3-methoxy-3-oxoprop-1-en-1-yl)phenyl)octa-1,3-diene-2,3-diyl)dibenzoate (68).

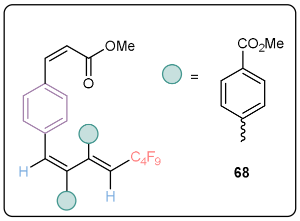

R$_f$ = 0.2 (silica gel, petroleum ether/ethyl acetate = 10:1).

^{1}**H NMR:** (500 MHz, Chloroform-*d*)) δ 7.97 – 7.88 (m, 4H), 7.65 (d, J = 16.0 Hz, 1H), 7.53 – 7.43 (m, 8H), 7.04 (s, 1H), 6.44 (d, J = 16.0 Hz, 1H), 6.00 (t, J = 15.1 Hz, 1H), 3.87 (s, 3H), 3.84 (s, 3H), 3.79 (s, 3H).

^{19}**F NMR:** (471 MHz, Chloroform-*d*) δ -81.0 (t, J = 9.9 Hz, 3F), -104.15 – -104.24 (m, 2F), -123.4 – -123.5 (m, 2F), -125.7 – -125.8 (m, 2F).

^{13}**C NMR:** (126 MHz, Chloroform-*d*) δ 167.4, 166.6, 166.4, 150.9 (t, J = 4.0 Hz), 144.0, 143.9, 142.7, 140.2, 137.3, 134.5, 132.0, 130.8, 130.04, 130.00, 129.7, 129.4, 129.0 (t, J = 3.6 Hz), 128.3, 127.3, 119.0 (t, J = 20.8 Hz), 118.5,

52.3, 52.2, 51.8.

HRMS (EI): calc'd for $C_{34}H_{26}F_9O_6^+$ Exact Mass: 701.1580, found 701.1579.

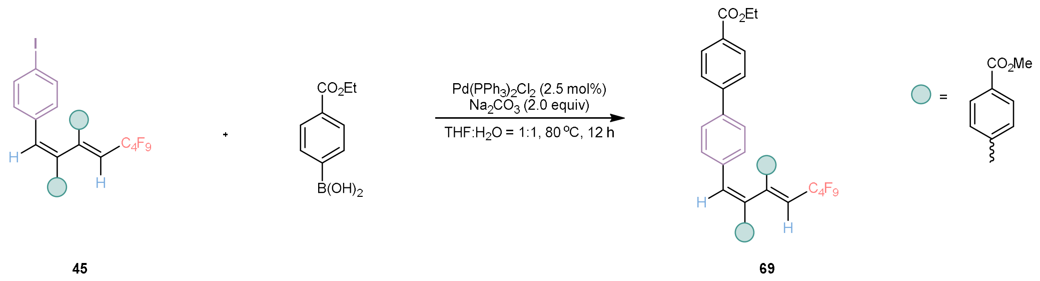

Supplementary Fig 13. Synthetic methods for Suzuki cross-coupling reaction

To an oven-dried 10 mL Young's Tube vial equipped with a magnetic stir bar was added **45** (0.05 mmol, 1.0 equiv), $PdCl_2(PPh_3)_2$ (10 mol%, 0.9 mg), (4-Ethoxycarbonylphenyl)boronic acid (0.075 mmol, 1.5 equiv), Na_2CO_3 (0.1 mmol， 2.0 equiv), then THF (0.5 mL) and H_2O (0.5 mL) were added. The resulting reaction mixture was stirring at 80 °C for 12 h. After the reaction was completed, the reaction mixture was extracted with EtOAc and the combined organic layers were dried over Na_2SO_4, filtered and concentrated under reduced pressure. The product was purified by column chromatography over silica gel (petroleum ether/ethyl acetate = 15:1-8:1) to afford compound **69** (23.7 mg, 0.031 mmol) as yellow liquid in 62% yield.

Dimethyl-4,4'-((1*Z*,3*E*)-1-(4'-(ethoxycarbonyl)-[1,1'-biphenyl]-4-yl)-5,5,6,6,7,7,8,8,8-nonafluoroocta-1,3-diene-2,3-diyl)dibenzoate (69)

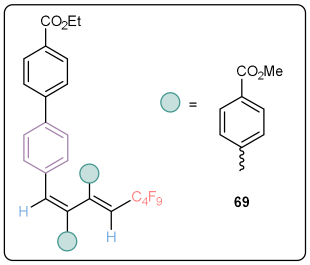

R*f* = 0.2 (silica gel, petroleum ether/ethyl acetate = 10:1).

^{1}H NMR: (500 MHz, Chloroform-*d*)) δ 8.13 – 8.11 (m, 2H), 7.99 – 7.97 (m, 2H), 7.92 – 7.89 (m, 2H), 7.66 – 7.64 (m, 2H), 7.62 – 7.58 (m, 4H), 7.48 – 7.46 (m, 4H), 7.10 (s, 1H), 6.03 (t, J = 15.0 Hz, 1H), 4.41 (q, J = 7.1 Hz, 2H), 3.90 (s, 3H), 3.87 (s, 3H), 1.42 (t, J = 7.1 Hz, 3H).

^{19}F NMR: (471 MHz, Chloroform-*d*) δ -80.9 (t, J = 9.4 Hz, 3F), -104.1 – -104.2 (m, 2F), -123.38 – -123.44 (m, 2F), -125.7 – -125.8 (m, 2F).

^{13}C NMR: (126 MHz, Chloroform-*d*) δ 166.7, 166.6, 166.5, 151.0 (t, J = 3.8 Hz), 144.7, 144.2, 142.1, 140.4, 140.1, 135.3, 132.3, 131.9, 130.7, 130.3, 130.1, 129.8, 129.4, 129.1 (t, J = 3.2 Hz), 127.5, 127.3, 127.0, 119.0 (t, J = 20.4

Hz), 115.3, 61.2, 52.34, 52.32, 14.5.

HRMS (EI): calc'd for $C_{39}H_{30}F_9O_6^+$ Exact Mass: 765.1893, found 765.1898.

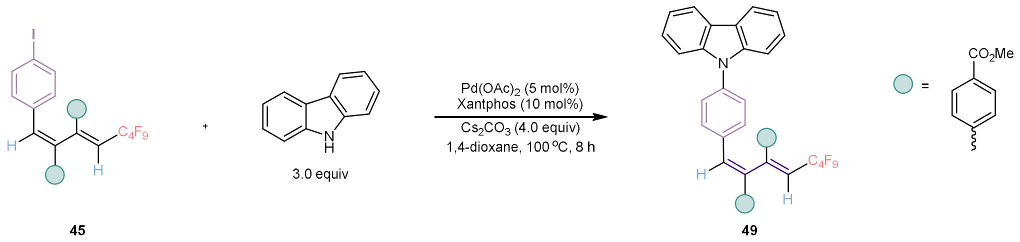

Supplementary Fig 14. Synthetic methods for Buchwald-Hartwig cross-coupling reaction

To an oven-dried 10 mL Young's Tube vial equipped with a magnetic stir bar was added **45** (0.05 mmol, 1.0 equiv), $Pd(OAc)_2$ (5 mol%, 0.6 mg), Xantphos (10 mol%, 2.9 mg), Cs_2CO_3 (0.2 mmol, 4.0 equiv), then 1,4-dioxane (0.5 mL) was added. The resulting reaction mixture was stirring at 100 °C for 12 h. After the reaction was completed, the reaction mixture was extracted with EtOAc and the combined organic layers were dried over Na_2SO_4, filtered and concentrated under reduced pressure. The product was purified by column chromatography over silica gel (petroleum ether/ethyl acetate = 20:1) to afford compound **49** (36 mg, 0.046 mmol) as colorless liquid in 92% yield.

Dimethyl-4,4'-(*(1Z,3E)*-1-(4-(9H-carbazol-9-yl)phenyl)-5,5,6,6,7,7,8,8,8-nonafluoroocta-1,3-diene-2,3-diyl)dibenzoate (49)

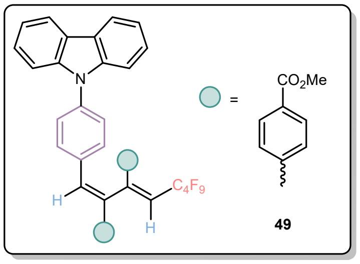

49

R_f = 0.3 (silica gel, petroleum ether/ethyl acetate = 10:1).

^{1}H NMR: (500 MHz, Chloroform-*d*) δ 8.17 – 8.15 (m, 2H), 8.04 – 7.95 (m, 4H), 7.72 – 7.69 (m, 2H), 7.56 – 7.51 (m, 6H), 7.45 – 7.39 (m, 4H), 7.33 – 7.30 (m, 2H), 7.19 (s, 1H), 6.11 (t, J = 15.0 Hz, 1H), 3.93 (s, 3H), 3.89 (s, 3H).

^{19}F NMR: (471 MHz, Chloroform-*d*) δ -80.8 (t, J = 9.2 Hz, 3F), -103.6 – -103.7 (m, 2F), -123.15 – -123.22 (m, 2F), -125.6 – -125.7 (m, 2F).

^{13}C NMR: (126 MHz, Chloroform-*d*) δ 166.7, 166.4, 151.2 (t, J = 3.8 Hz), 144.3, 142.6, 140.7, 140.4, 137.7, 134.6, 132.3, 130.8, 130.7, 130.1, 130.0, 129.4, 129.3 (t, J = 4.3 Hz), 127.4, 127.1, 126.2, 123.7, 120.5, 120.3, 119.2 (t, J = 19.0 Hz), 109.8, 52.4.

HRMS (ESI): calc'd for $C_{42}H_{29}F_9NO_4^+$ Exact Mass: 782.1947, found 782.1941.

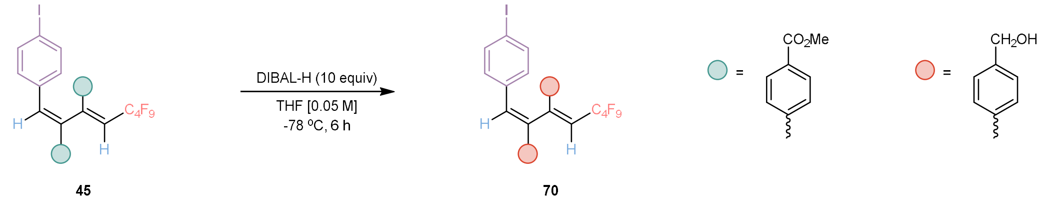

Supplementary Fig 15. Synthetic methods for reduction reaction

To an oven-dried 10 mL Young's Tube vial equipped with a magnetic stir bar, **45** (0.05 mmol, 1.0 equiv) and THF (0.5 mL) were added, then a solution of DIBAL-H (0.5 mmol, 1.0 M in n-hexane) was slowly added at -78 ^{o}C. The resulting reaction mixture was stirring at -78 ^{o}C for 6 h. After the reaction was completed, the reaction was quenched with a saturated aqueous NH_4Cl solution and extracted with EtOAc and the combined organic layers were dried over Na_2SO_4, filtered and concentrated under reduced pressure. The product was purified by column chromatography over silica gel (petroleum ether/ethyl acetate = 10:1-5:1) to afford compound **70** (30.9 mg, 0.045 mmol) as colorless liquid in 90% yield.

(((1*Z*,3*E*)-5,5,6,6,7,7,8,8,8-nonafluoro-1-(4-iodophenyl)octa-1,3-diene-2,3-diyl)bis(4,1-phenylene))dimethanol (70)

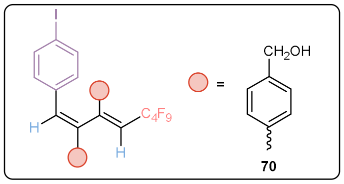

R*f* = 0.2 (silica gel, petroleum ether/ethyl acetate = 2:1).

^{1}H NMR: (500 MHz, Chloroform-*d*) δ 7.64 – 7.61 (m, 2H), 7.40 – 7.36 (m, 4H), 7.25 – 7.17 (m, 6H), 6.87 (s, 1H), 5.89 (t, J = 14.6 Hz, 1H), 4.55 – 4.50 (m, 4H), 2.18 (s, 2H).

^{19}F NMR: (471 MHz, Chloroform-*d*) δ -80.9 (t, J = 10.0 Hz, 3F), -103.8 – -103.9 (m, 2F), -123.55 – -123.61 (m, 2F), -125.65 – -125.74 (m, 2F).

^{13}C NMR: (126 MHz, Chloroform-*d*) δ 151.8 (t, J = 4.1 Hz), 143.5, 142.0, 141.0, 139.1, 137.7, 135.4, 135.0, 130.8, 129.4, 129.3 (t, J = 3.4 Hz), 127.3, 126.6, 117.0 (t, J = 20.3 Hz), 93.5, 77.2, 64.7, 64.6.

HRMS (ESI): calc'd for $C_{28}H_{20}F_9IO_2Na^+$ Exact Mass: 709.0256, found 709.0255.

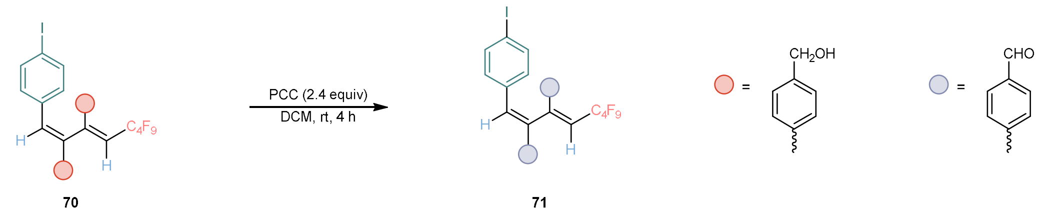

Supplementary Fig 16. Synthetic methods for oxidation reaction

To an oven-dried 10 mL Young's Tube vial equipped with a magnetic stir bar was **45** (0.05 mmol, 1.0 equiv), PCC (0.12 mmol, 2.4 equiv), then DCM (1 mL) was added. The resulting reaction mixture was stirring at room temperature for 4 h. After the reaction was completed, the reaction mixture was filtered and concentrated under reduced pressure, and the product was purified by column chromatography over silica gel (petroleum ether/ethyl acetate = 20:1-10:1) to afford compound **71** (29 mg, 0.04 mmol) as colorless liquid in 85% yield.

4,4'-((1*Z*,3*E*)-5,5,6,6,7,7,8,8,8-nonafluoro-1-(4-iodophenyl)octa-1,3-diene-2,3-diyl)dibenzaldehyde (71)

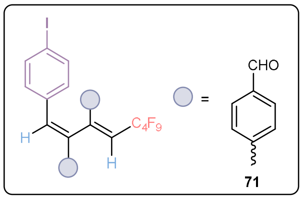

R*f* = 0.2 (silica gel, petroleum ether/ethyl acetate = 10:1).

^{1}H NMR: (500 MHz, Chloroform-*d*) δ 9.96 (s, 1H), 9.93 (s, 1H), 7.82 (d, J = 8.0 Hz, 2H), 7.75 (d, J = 8.1 Hz, 2H), 7.69 – 7.63 (m, 2H), 7.55 (d, J = 7.9 Hz, 2H), 7.51 (d, J = 8.0 Hz, 2H), 7.22 (d, J = 8.4 Hz, 2H), 7.02 (s, 1H), 6.02 (t, J = 15.0 Hz, 1H).

^{19}F NMR: (471 MHz, Chloroform-*d*) (471 MHz, Chloroform-*d*) δ -80.9 (t, J = 10.0 Hz, 3F), -104.2 – -104.3 (m, 2F), -123.36 – -123.42 (m, 2F), -125.7 – -125.8 (m, 2F).

^{13}C NMR: (126 MHz, Chloroform-*d*) δ 191.49, 191.46, 150.4 (t, J = 4.4 Hz), 145.5, 142.0, 141.6, 137.9, 136.5, 136.1, 134.7, 132.7, 130.8, 130.2, 129.6 (t, J = 3.0 Hz), 129.4, 127.8, 119.7 (t, J = 21.3 Hz), 94.6.

HRMS (ESI): calc'd for $C_{28}H_{17}F_9IO_2^+$ Exact Mass: 683.0124, found 683.0131.

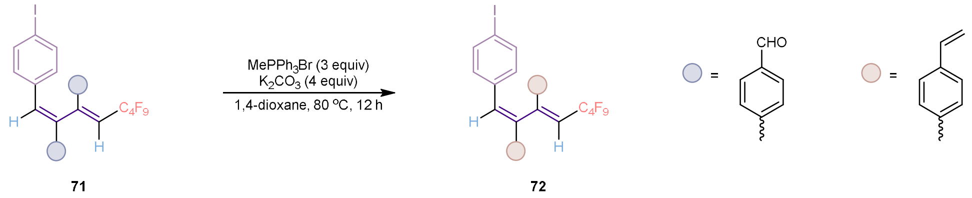

Supplementary Fig 17. Synthetic methods for Wittig reaction

To an oven-dried 10 mL Young's Tube vial equipped with a magnetic stir bar was added **45** (0.05 mmol, 1.0 equiv), $MePPh_3Br$ (0.15 mmol, 3.0 equiv), K_2CO_3 (0.2 mmol, 4.0 equiv), then 1,4-dioxane (1 mL) was added. The resulting reaction mixture was stirring at 80 °C for 12 h. After the reaction was completed, the reaction mixture was extracted with EtOAc and the combined organic layers were dried over Na_2SO_4, filtered and concentrated under reduced pressure. The product was purified by column chromatography over silica gel (petroleum ether) to afford compound **72** (30.5 mg, 0.045 mmol) as colorless liquid in 90% yield.

4,4'-((1*Z*,3*E*)-5,5,6,6,7,7,8,8,8-nonafluoro-1-(4-iodophenyl)octa-1,3-diene-2,3-diyl)bis(vinylbenzene) (72)

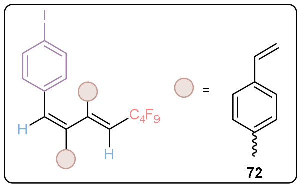

R*f* = 0.5 (silica gel, petroleum ether).

^{1}H NMR: (500 MHz, Chloroform-*d*) δ 7.65 – 7.62 (m, 2H), 7.40 – 7.34 (m, 6H), 7.31 – 7.28 (m, 2H), 7.25 – 7.23 (m, 2H), 6.91 (s, 1H), 6.70 – 6.61 (m, 2H), 5.88 (t, J = 15.3 Hz, 1H), 5.76 – 5.72 (m, 2H), 5.26 (dd, J = 1.1, 10.9 Hz, 2H).

^{19}F NMR: (471 MHz, Chloroform-*d*) δ -80.9 (t, J = 8.9 Hz, 3F), -103.8 – -103.9 (m, 2F), -123.5 – -123.6 (m, 2F), -125.6 – -125.7 (m, 2F).

^{13}C NMR: (126 MHz, Chloroform-*d*) δ 151.8 (t, J = 4.7 Hz), 143.5, 139.2, 138.5, 137.7, 137.6, 136.2, 136.2, 135.5, 135.1, 130.9, 129.5 (t, J = 3.3 Hz), 129.1, 127.3, 126.6, 126.0, 116.8 (t, J = 20.3 Hz), 115.2, 114.6, 93.5.

HRMS (ESI): calc'd for $C_{30}H_{21}F_9I^+$ Exact Mass: 679.0539, found 679.0536.

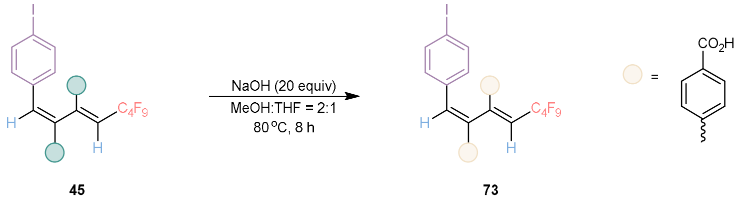

Supplementary Fig 18. Synthetic methods for hydrolysis

To an oven-dried 10 mL Young's Tube vial equipped with a magnetic stir bar was added **45** (0.05 mmol, 1.0 equiv), NaOH (1 mmol, 20 equiv), then MeOH (0.8 mL) and THF (0.4 mL) were added (without argon atmosphere). The resulting reaction mixture was stirring at 80 °C for 8 h. After the reaction was completed, the reaction mixture was extracted with EtOAc and the combined organic layers were dried over Na_2SO_4, filtered and concentrated under reduced pressure. The product was purified by column chromatography over silica gel (ethyl acetate) to afford compound **73** (30.7 mg, 0.043 mmol) as yellow liquid in 86% yield.

4,4'-((1Z,3E)-5,5,6,6,7,7,8,8,8-nonafluoro-1-(4-iodophenyl)octa-1,3-diene-2,3-diyl)dibenzoic acid (73)

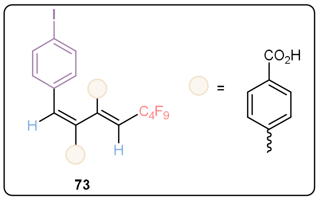

R$_f$ = 0.1 (silica gel, ethyl acetate).

^{1}H NMR: (500 MHz, Chloroform-*d*) δ 8.00 – 7.96 (m, 4H), 7.67 (d, J = 8.0 Hz, 2H), 7.45 (dd, J = 8.1, 24.1 Hz, 4H), 7.25 (d, J = 8.4 Hz, 2H), 7.00 (s, 1H), 6.01 (t, J = 15.1 Hz, 1H).

^{19}F NMR: (471 MHz, Chloroform-*d*) δ -81.0 (t, J = 9.6 Hz, 3F), -104.3 – -104.4 (m, 2F), -123.4 – -123.5 (m, 2F), -125.7 – -125.8 (m, 2F).

^{13}C NMR: (126 MHz, Chloroform-*d*) δ 172.0, 171.8, 150.3 (t, J = 5.3 Hz), 144.7, 142.2, 141.0, 138.0, 134.8, 132.3, 130.81, 130.76, 130.2, 130.0, 129.2 (t, J = 2.5 Hz), 129.0, 127.4, 119.2 (t, J = 20.7 Hz), 94.5.

HRMS (ESI): calc'd for $C_{28}H_{16}F_9IO_4Na^+$ Exact Mass: 736.9842, found 736.9847.

7. Mechanism experiments

7.1 Controlled experiments

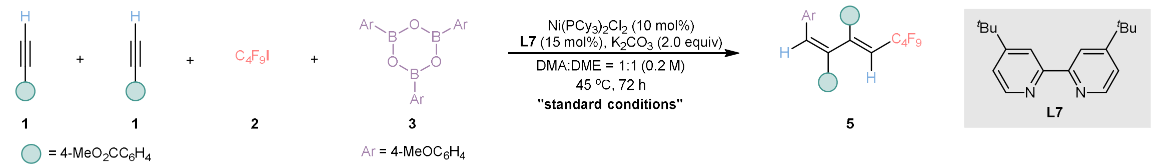

To an oven-dried 10 mL Young's Tube vial equipped with a magnetic stir bar was added **L7** (0.03 mmol, 8.05 mg, 15 mol%), $Ni(PCy_3)_2Cl_2$ (0.02 mmol, 13.8 mg, 10 mol%), terminal alkynes (0.54 mmol, 2.7 equiv), aryl boroxine (0.2 mmol, 27 mg), K_2CO_3 (0.4 mmol, 56 mg, 2.0 equiv), then the vial was introduced in a argon-filled atmosphere, perflfluoroalkyl iodides (0.8 mmol, 4.0 equiv), anhydrous DMA (0.5 mL, 0.2 M) and anhydrous DME (0.5 mL, 0.2 M) were added. The resulting reaction mixture was stirred at 45 °C in an oil bath at 660 rpm for 72 h. After the reaction was completed, the reaction yield was detected by GC analysis with *n*-dodecane as internal standard, the results and GCMS spectrum for different controlled experiments are shown in **Supplementary Table 13.**

Supplementary Table 13. Controlled experiments

entry	variation from standard conditions	yield[a]
1	none	81 %
2	w/o Ni catalyst	N.D.
3	w/o Ligand	< 5%
4	w/o K_2CO_3	N.D.

[a] Yields were determined by gas chromatography (GC) using *n*-dodecane as the internal standard.

Supplementary Fig 19. Standard conditions (Detected by GCMS)

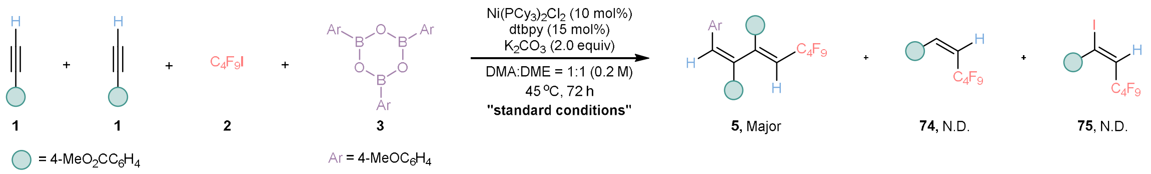

Supplementary Fig 20. In the absence of Ni catalyst (Detected by GCMS)

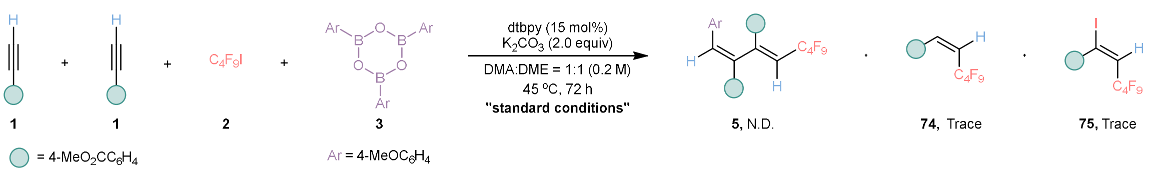

Supplementary Fig 21. In the absence of ligand (Detected by GCMS)

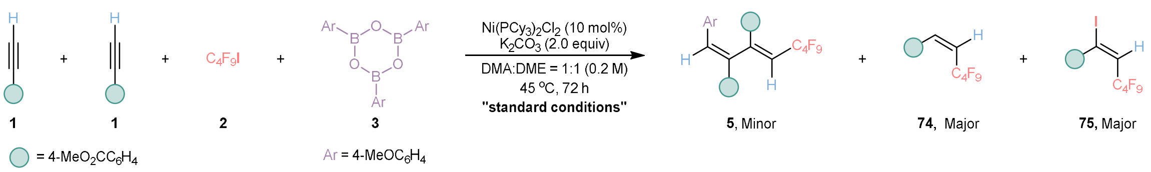

Supplementary Fig 22. In the absence of base (Detected by GCMS)

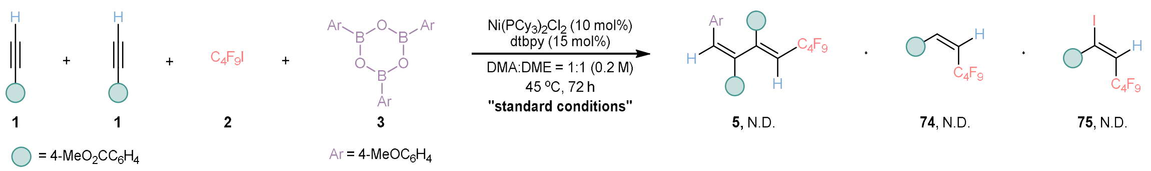

7.2 Radical inhibition reactions

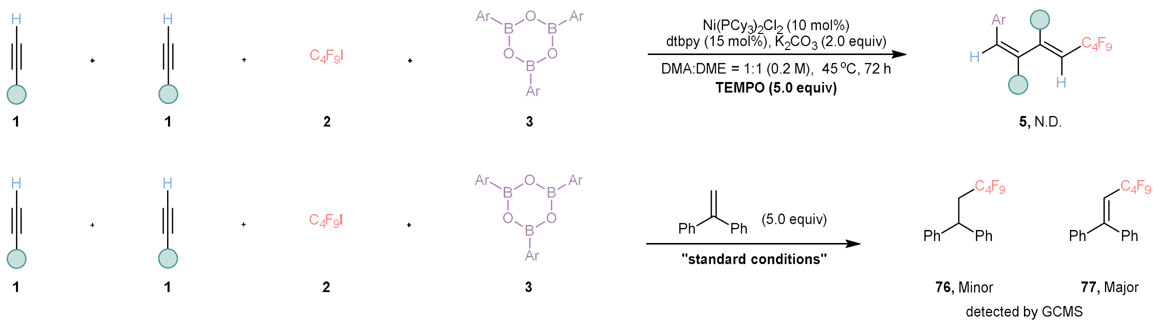

Supplementary Fig 23. Radical inhibition reactions

Addition of 1,1-diphenylethylene

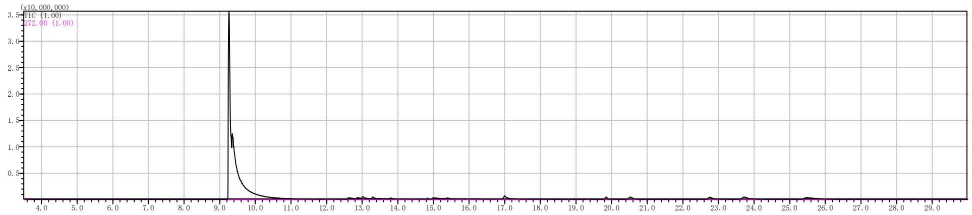

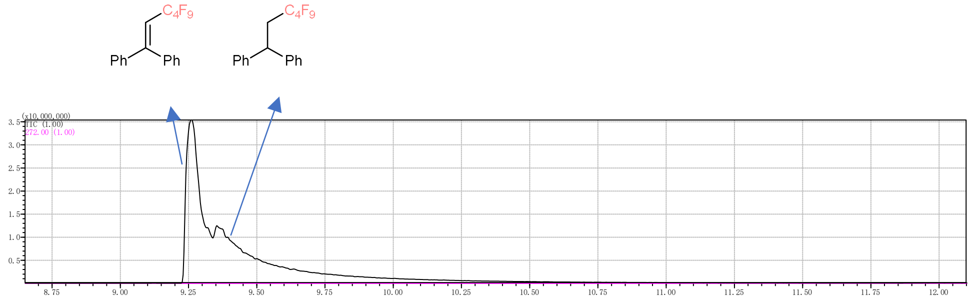

Left peak

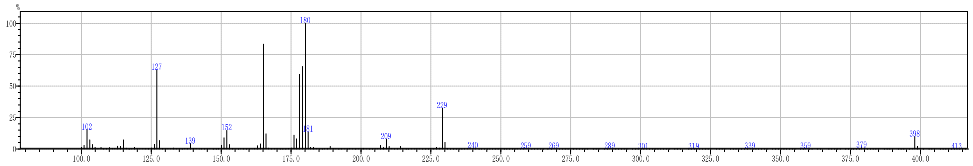

Right peak

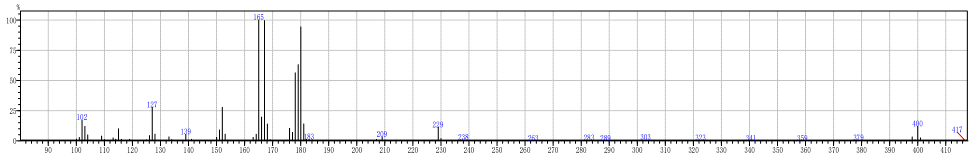

7.3 The influence of two ligands on the reaction was explored

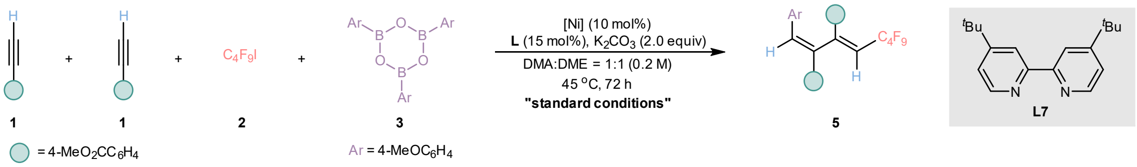

To an oven-dried 10 mL Young's Tube vial equipped with a magnetic stir bar was added ligand (0.03 mmol, 15 mol%), Nickel catalyst (0.02 mmol, 10 mol%), terminal alkynes (0.54 mmol, 2.7 equiv), boroxine (0.667 mmol, 27 mg), K_2CO_3 (0.4 mmol, 56 mg, 2.0 equiv), The vial was introduced in a argon-filled atmosphere, perflfluoroalkyl iodides (0.8 mmol, 4.0 equiv), anhydrous DMA (0.5 mL, 0.2 M) and anhydrous DME (0.5 mL, 0.2 M) were added. Then the reaction mixture was stirred at 45 °C in an oil bath at 660 rpm for 72 h. After the reaction was completed, the reaction yield was detected by GC analysis with *n*-dodecane as internal standard, the results for different controlled experiments are shown in **Supplementary Table 14.**

Supplementary Table 14. Controlled experiments

entry	variation from standard conditions	yield[a]
1	$Ni(PCy_3)_2Cl_2$ (10 mol%) + dtbpy (15 mol%)	81 %
2	$Ni(dtbpy)Cl_2$ (10 mol%)	81 %
3	$Ni(dtbpy)Cl_2$ (10 mol%) + PCy_3 (10 mol%)	81%
4	$NiCl_2$ (10 mol%) + dtbpy (15 mol%)	Trace
5	$NiCl_2$ (10 mol%) + PCy_3 (15 mol%)	Trace

[a] Yields were determined by gas chromatography (GC) using *n*-dodecane as the internal standard.

7.4 Two different alkynes were involved in the reaction

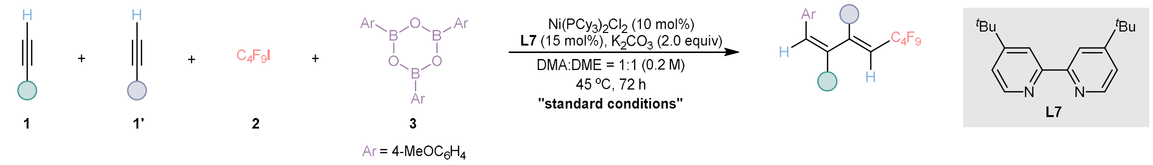

To an oven-dried 10 mL Young's Tube vial equipped with a magnetic stir bar was added **L7** (0.03 mmol, 8.05 mg, 15 mol%), $Ni(PCy_3)_2Cl_2$ (0.02 mmol, 13.8 mg, 10 mol%), terminal alkynes **1** (0.27 mmol, 1.35 equiv) and terminal alkynes **1'** (0.27 mmol, 1.35 equiv), boroxine (0.667 mmol, 27 mg), K_2CO_3 (0.4 mmol, 56 mg, 2.0 equiv), The vial was introduced in a argon-filled atmosphere, perflfluoroalkyl iodides (0.8 mmol, 4.0 equiv), anhydrous DMA (0.5 mL, 0.2 M) and anhydrous DME (0.5 mL, 0.2 M) were added. Then the reaction mixture was stirred at 45 °C in an oil bath at 660 rpm for 72 h. After the reaction was complete, the reaction yield was detected by GCMS.

Supplementary Fig 24. Two different alkynes were involved in our reaction system

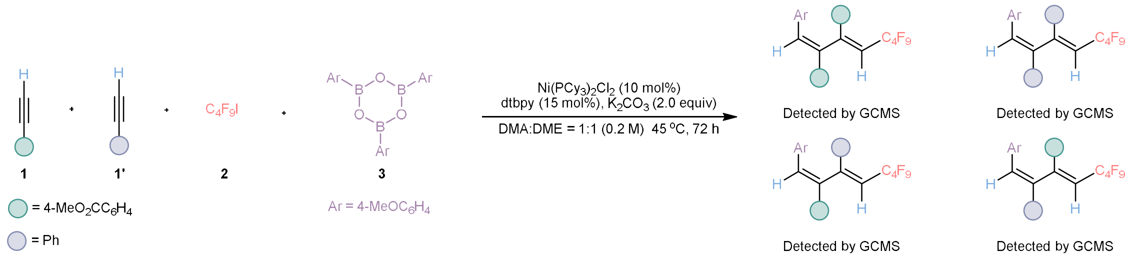

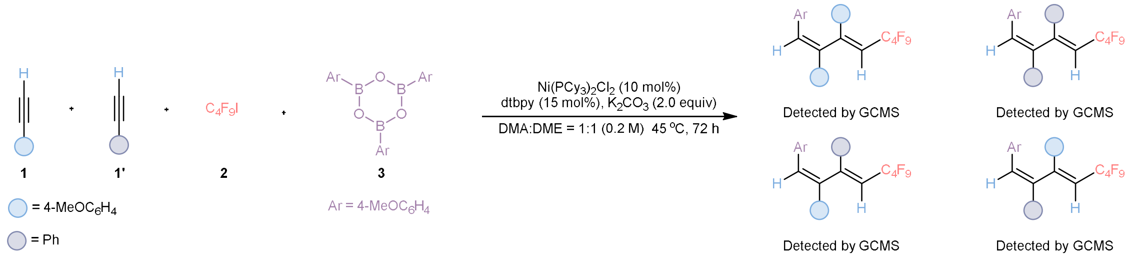

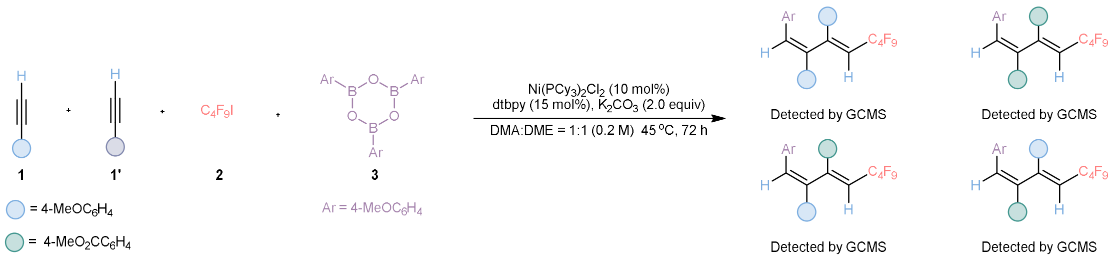

The use of largely excessive of one alkyne

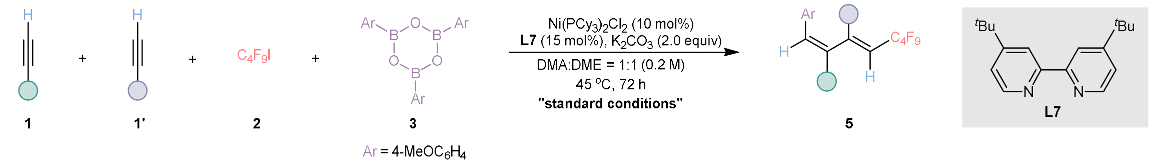

To an oven-dried 10 mL Young's Tube vial equipped with a magnetic stir bar was added **L7** (0.03 mmol, 8.05 mg, 15 mol%), $Ni(PCy_3)_2Cl_2$ (0.02 mmol, 13.8 mg, 10 mol%), terminal alkynes **1** (0.2 mmol, 1.0 equiv) and terminal alkynes **1'** (0.60 mmol, 3.0 equiv) or terminal alkynes **1** (0.6 mmol, 3.0 equiv) and terminal alkynes **1'** (0.20 mmol, 1.0 equiv), boroxine (0.667 mmol, 27 mg), K_2CO_3 (0.4 mmol, 56 mg, 2.0 equiv), The vial was introduced in a argon-filled atmosphere, perflfluoroalkyl iodides (0.8 mmol, 4.0 equiv), anhydrous DMA (0.5 mL, 0.2 M) and anhydrous DME (0.5 mL, 0.2 M) were added. Then the reaction mixture was stirred at 45 °C in an oil bath at 660 rpm for 72 h. After the reaction was complete, the reaction yield was detected by GCMS.

Supplementary Fig 25. The use of largely excessive of one alkyne

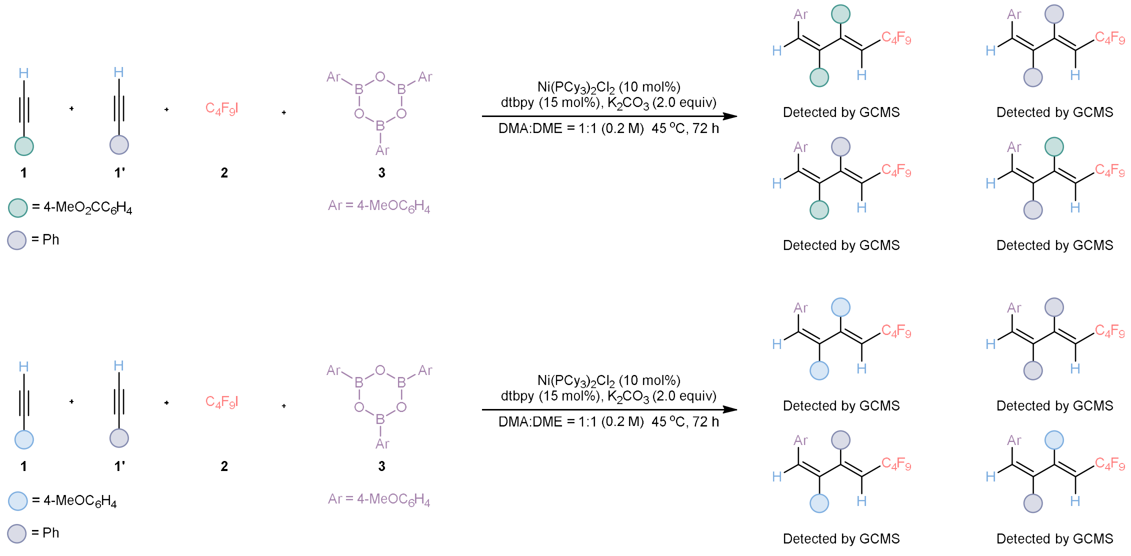

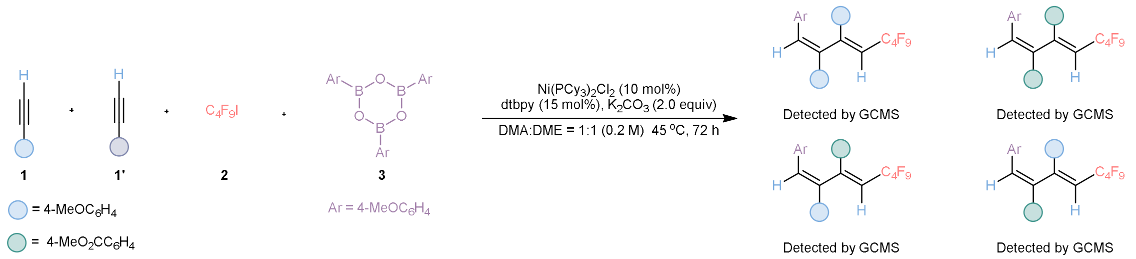

7.5 Unsuccessful examples

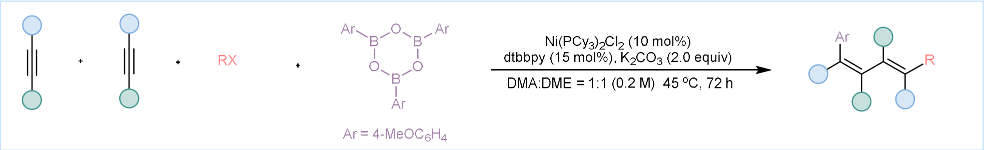

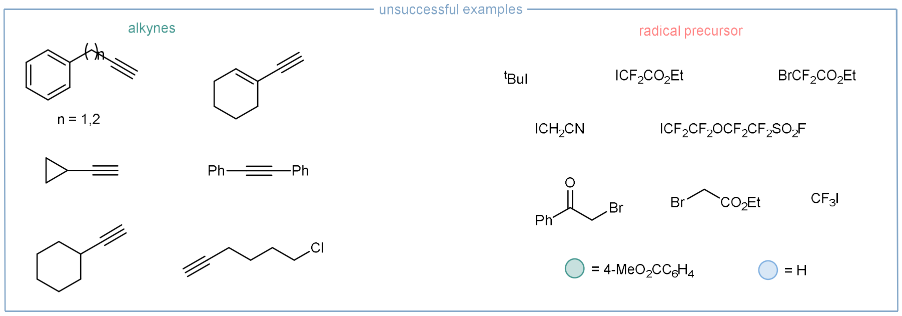

Supplementary Fig 26. Incompatible substrate in our reaction system

8. Density Functional Theory (DFT) calculations

8.1 Computational details

All the calculations were performed with the Gaussian 09 suite of programs by using DFT, and all geometry optimizations were completed at the M06/6-31G(d,p)(SDD for Ni,K,I)/SMD_{DCM} level. All geometry optimization calculations were followed by frequency calculations to verify that the structures of the stationary points are the expected ones. Moreover, we further refined the energy by employing the single-point energy calculations at the M06/6-311++G(d,p)(SDD for Ni,K,I)/SMD_{DCM} level based on the optimized structures at the M06/6-31G(d,p)(SDD for Ni,K,I)/SMD_{DCM} level.

8.2 All possible pathways for nickel-catalyzed cascade difunctionalization reaction

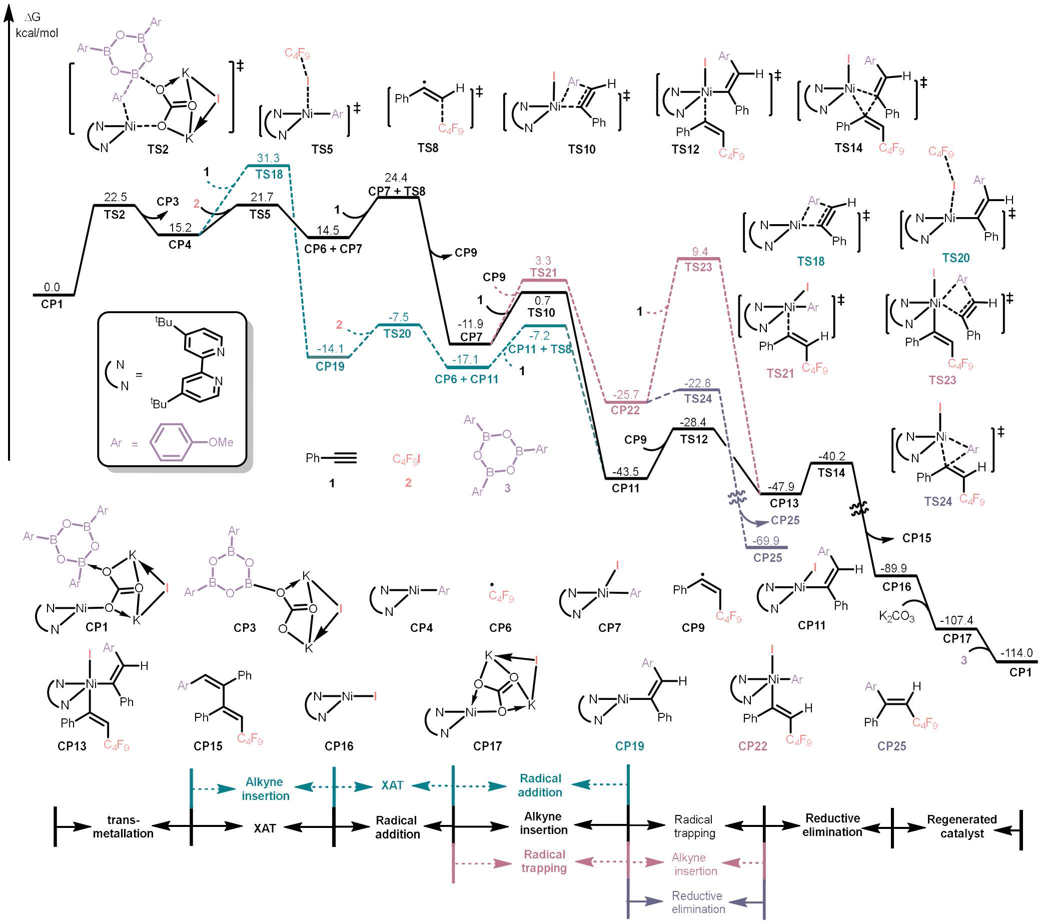

Supplementary Fig 27. Free energy profiles for all possible reaction pathways.

8.3 A comparison of the energy barriers of the transition states for these two alkyne insertion pathways.

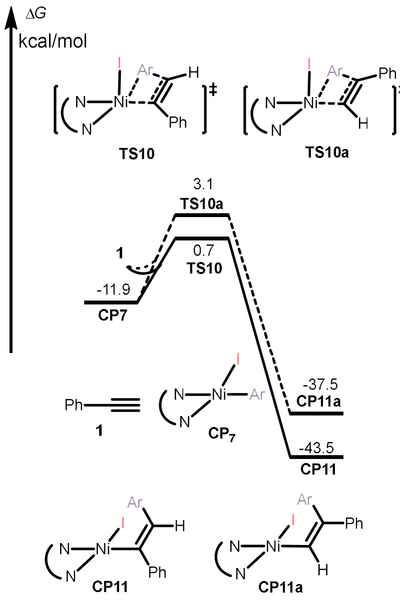

Supplementary Fig 28. Free energy profiles for alkyne 1,2-insertion and 2,1-insertion step.
As shown in Figure 4, the subsequent 1,2- and 2,1-insertion pathways have been evaluated via transition states **TS10** and **TS10a**, respectively. The relative free energy of the 2,1-insertion transition state **TS10a** is 15.0 kcal/mol, which is 2.4 kcal/mol higher than that **TS10**.

8.4 The directly transmetallation process in the absence of base.

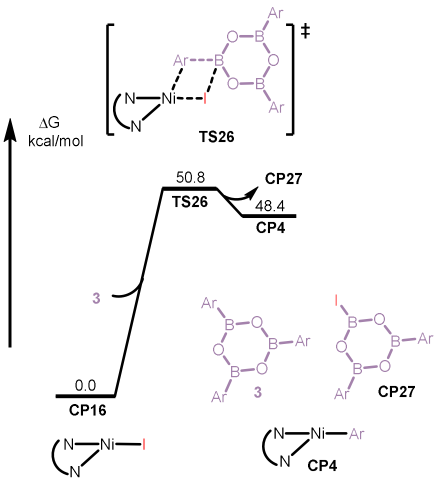

Supplementary Fig 29. The free energy profile for the transmetallation process in the absence of base.

As shown in Figure 5, we also tried to find out the directly transmetallation. We can find that the Ni(I) species and aryl boroxine undertake directly transmetallation process via the transition state **TS26** with a very high free energy barrier of 50.8 kcal/mol in the absence of base.

9. X-ray crystallographic data

Supplementary Table 15 Crystal data and structure refinement for **58.** Thermal ellipsoids are drawn at 50% probability level.

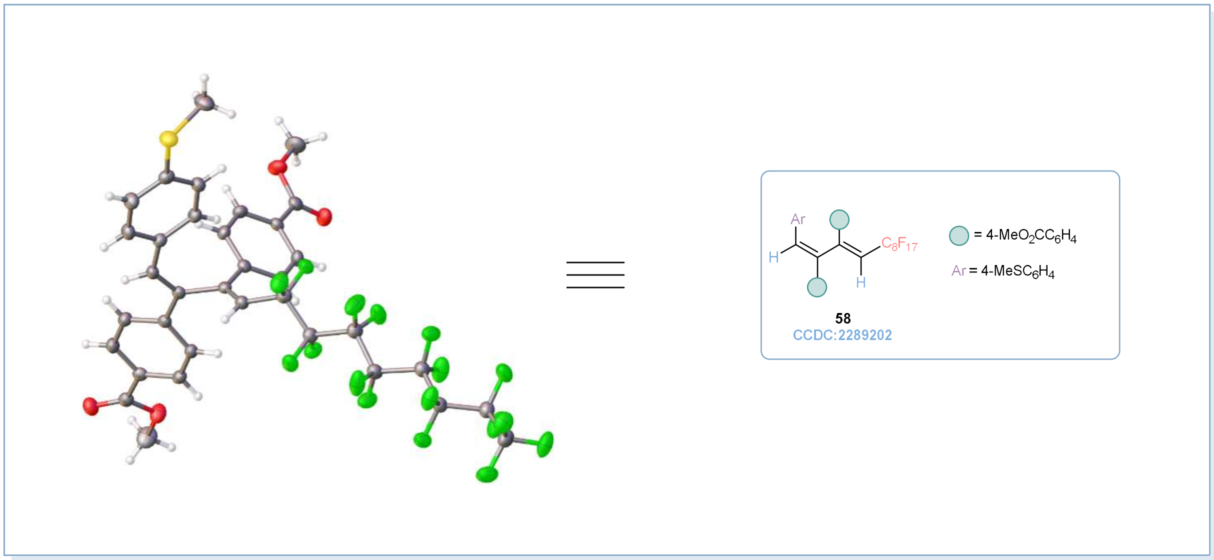

Supplementary Table 15. Crystal data and structure refinement for CSL5293_0m.

Identification code	CSL5293_0m
Empirical formula	$C_{35}H_{23}F_{17}O_4S$
Formula weight	862.59
Temperature/K	150.0
Crystal system	triclinic
Space group	P-1
a/Å	7.8326(18)
b/Å	12.014(3)
c/Å	19.565(4)
α/°	98.884(8)
β/°	97.530(8)
γ/°	101.507(8)
Volume/Å^3	1757.3(7)
Z	2
ρ_{calc}g/cm^3	1.630
μ/mm^{-1}	0.222
F(000)	868.0
Crystal size/mm^3	0.15 × 0.14 × 0.11
Radiation	MoKα (λ = 0.71073)
2Θ range for data collection/°	3.764 to 57.192
Index ranges	$-10 \leq h \leq 10$, $-16 \leq k \leq 16$, $-26 \leq l \leq 26$
Reflections collected	46464
Independent reflections	8872 [R_{int} = 0.0579, R_{sigma} = 0.0704]

Data/restraints/parameters	8872/0/517
Goodness-of-fit on F^2	1.045
Final R indexes [I>=2σ (I)]	$R_1 = 0.0451$, $wR_2 = 0.0894$
Final R indexes [all data]	$R_1 = 0.1025$, $wR_2 = 0.1039$
Largest diff. peak/hole / e $Å^{-3}$	0.28/-0.37

Supplementary Table 16. Crystal data and structure refinement for **59.** Thermal ellipsoids are drawn at 50% probability level.

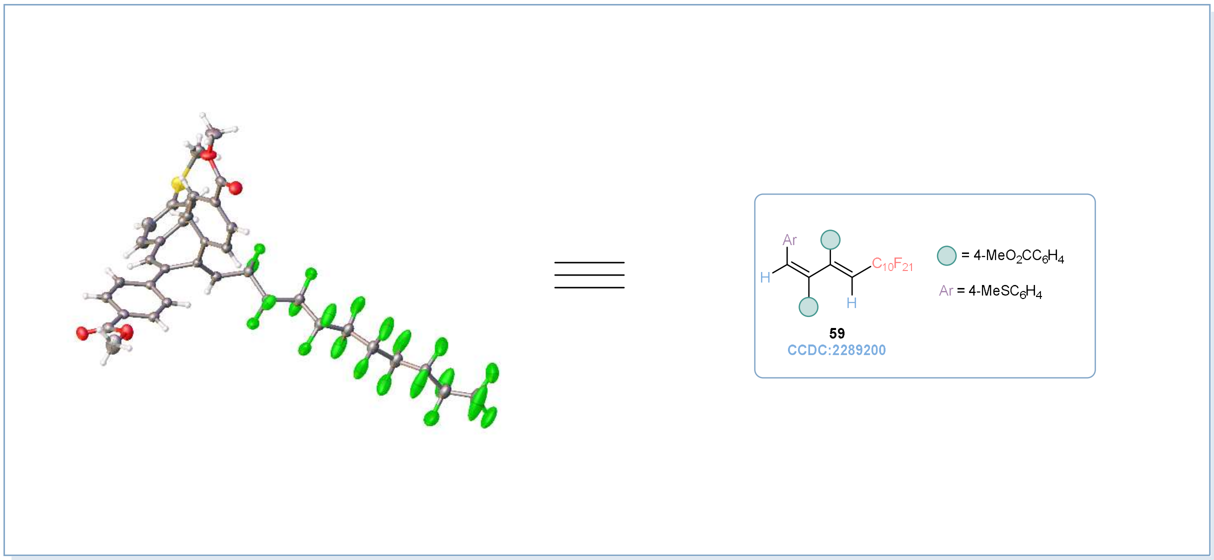

Supplementary Table 16. Crystal data and structure refinement for CSL530_0m.

Identification code	CSL530_0m
Empirical formula	$C_{37}H_{23}F_{21}O_4S$
Formula weight	962.61
Temperature/K	150
Crystal system	triclinic
Space group	P-1
a/Å	7.9307(12)
b/Å	12.0074(16)
c/Å	21.277(3)
α/°	100.243(5)
β/°	92.299(6)
γ/°	100.316(5)
Volume/$Å^3$	1956.2(5)
Z	2
$\rho_{calc}g/cm^3$	1.634
μ/mm^{-1}	0.223
F(000)	964.0
Crystal size/mm^3	0.15 × 0.13 × 0.12
Radiation	MoKα (λ = 0.71073)

2Θ range for data collection/°	4.326 to 56.234
Index ranges	$-10 \leq h \leq 10$, $-15 \leq k \leq 15$, $-28 \leq l \leq 28$
Reflections collected	43574
Independent reflections	9468 [R_{int} = 0.0517, R_{sigma} = 0.0516]
Data/restraints/parameters	9468/0/571
Goodness-of-fit on F^2	1.058
Final R indexes [I>=2σ (I)]	R_1 = 0.0689, wR_2 = 0.1840
Final R indexes [all data]	R_1 = 0.1112, wR_2 = 0.2072
Largest diff. peak/hole / e Å^{-3}	0.97/-0.71

10. NMR spectrum of the new compounds

^{1}H NMR spectrum of **1aa** (500 MHz, Chloroform-*d*)

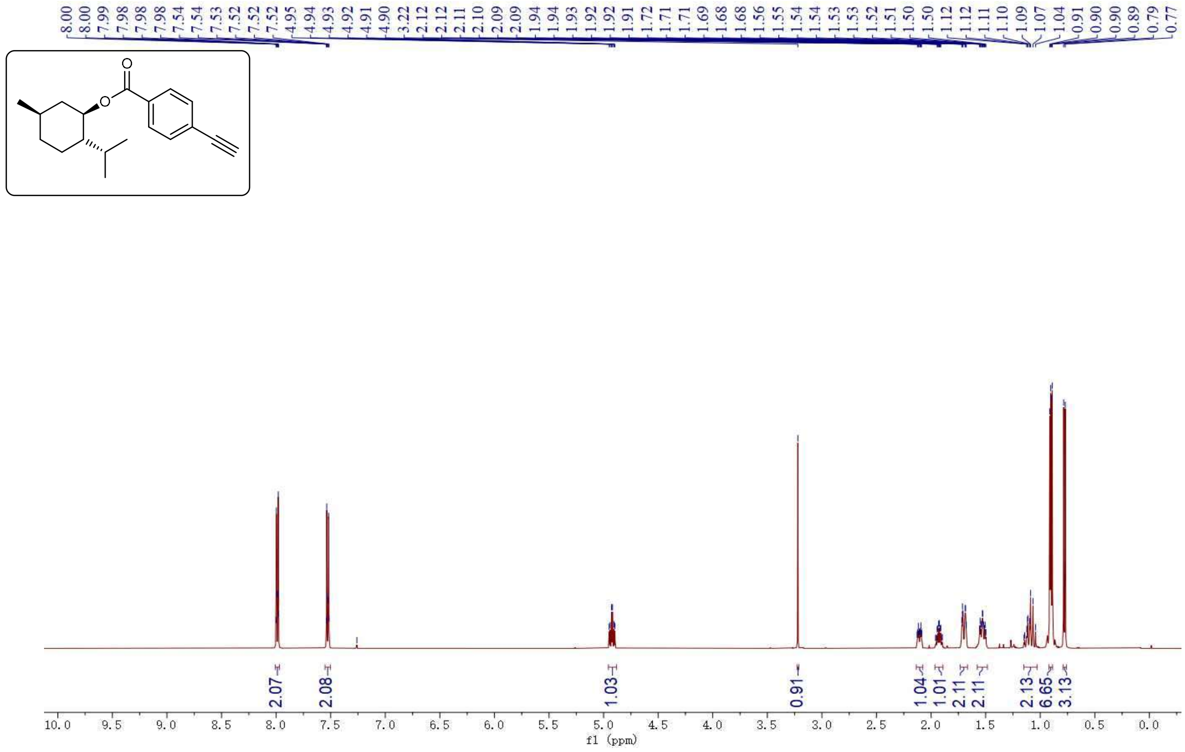

^{13}C NMR spectrum of **1aa** (126 MHz, Chloroform- *d*)

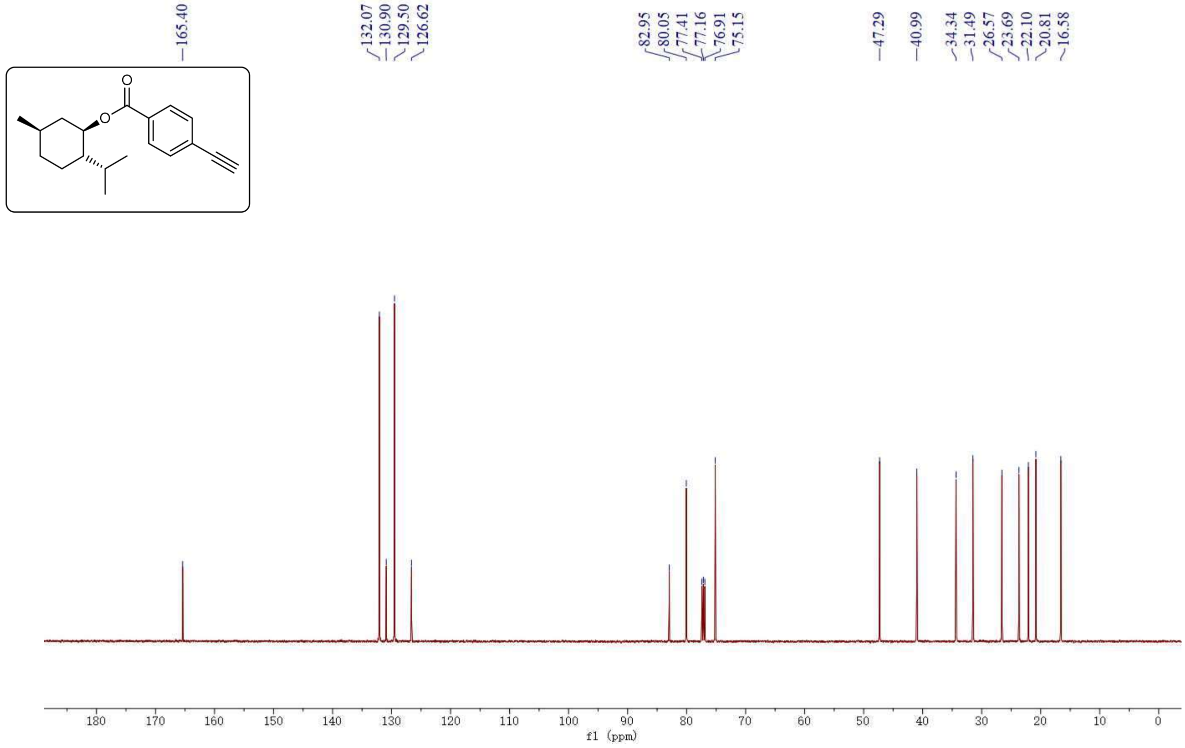

^{1}**H NMR** spectrum of **1ab** (500 MHz, Chloroform-*d*)

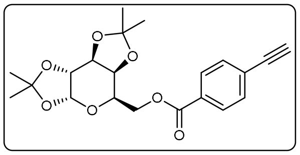

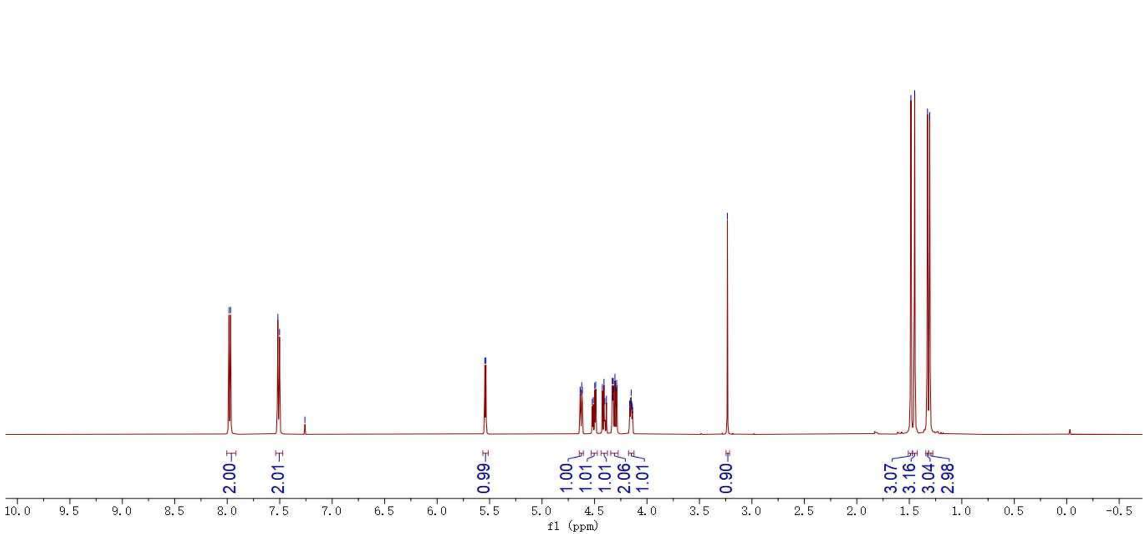

^{13}**C NMR** spectrum of **1ab** (126 MHz, Chloroform-*d*)

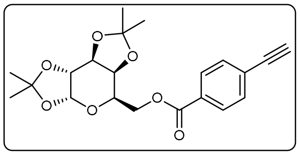

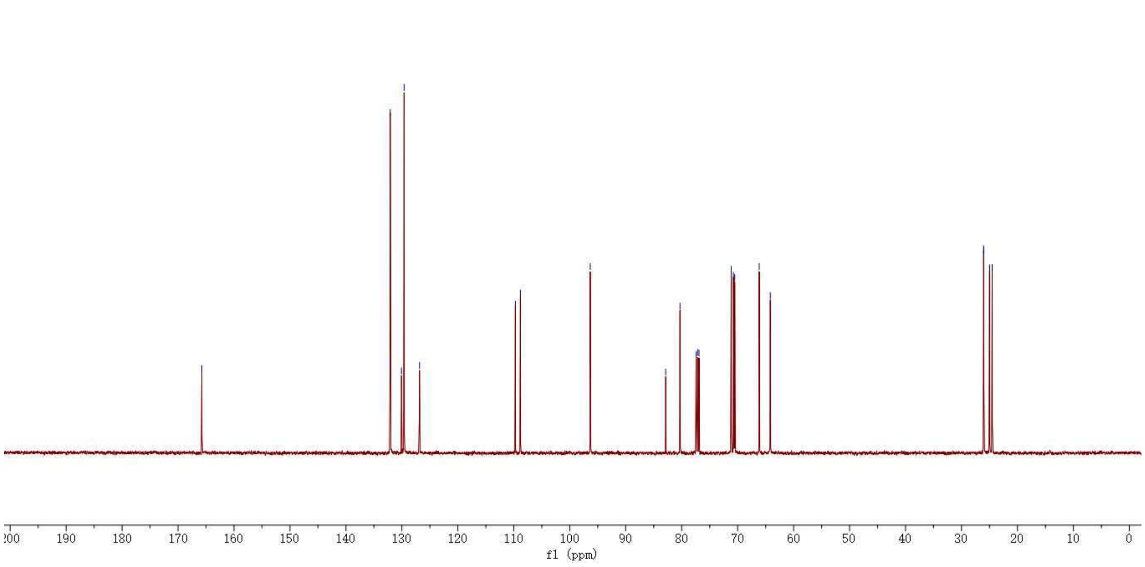

^{1}H NMR spectrum of **1ac** (500 MHz, Chloroform-*d*)

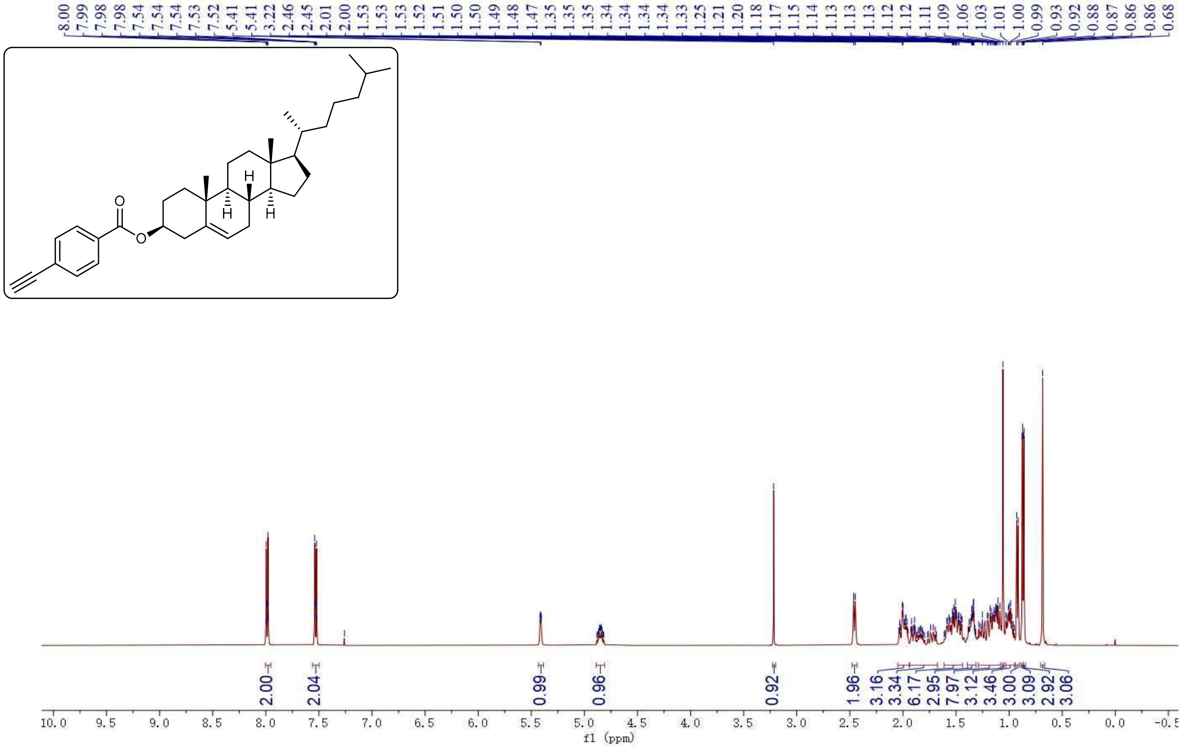

^{13}C NMR spectrum of **1ac** (126 MHz, Chloroform-*d*)

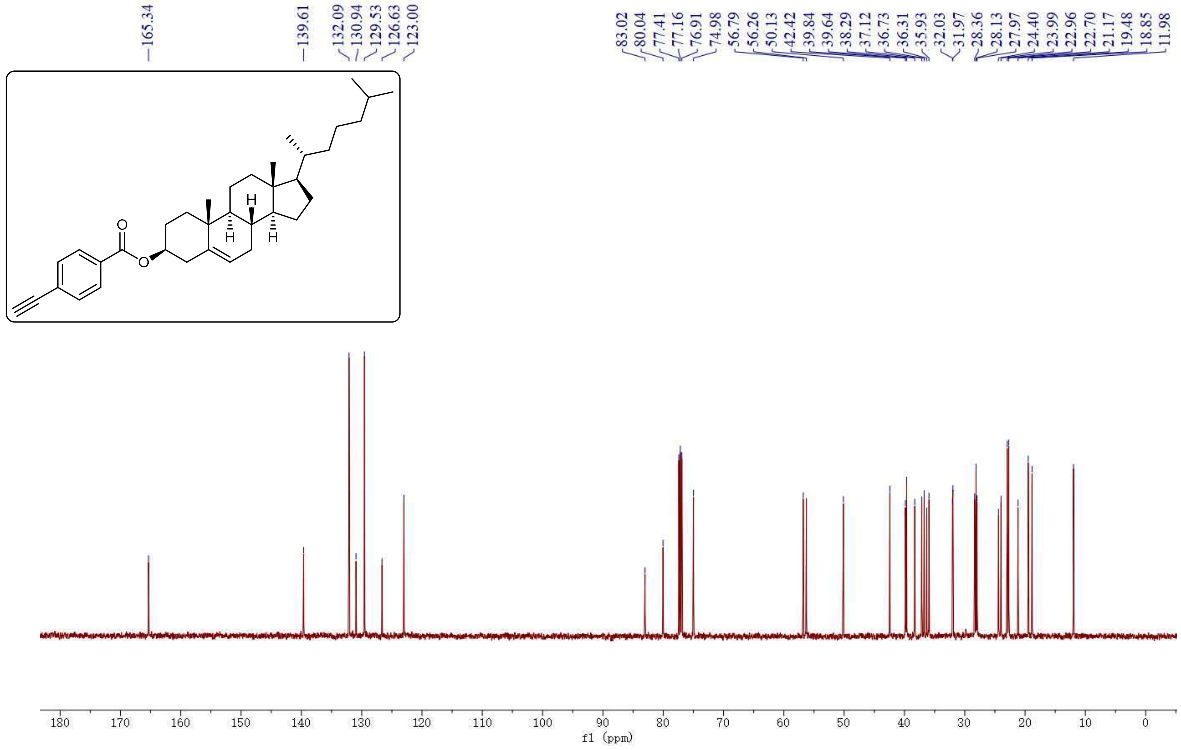

^{1}H NMR spectrum of **1ad** (500 MHz, Chloroform-*d*)

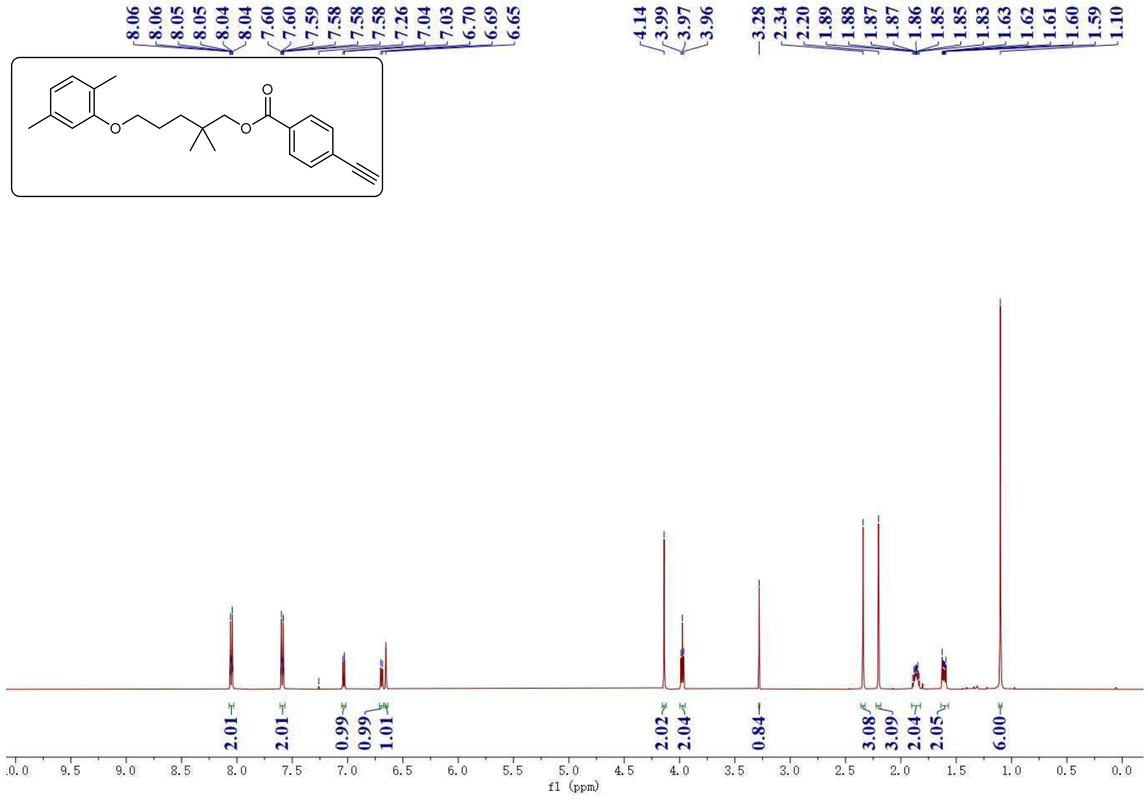

^{13}C NMR spectrum of **1ad** (126 MHz, Chloroform-*d*)

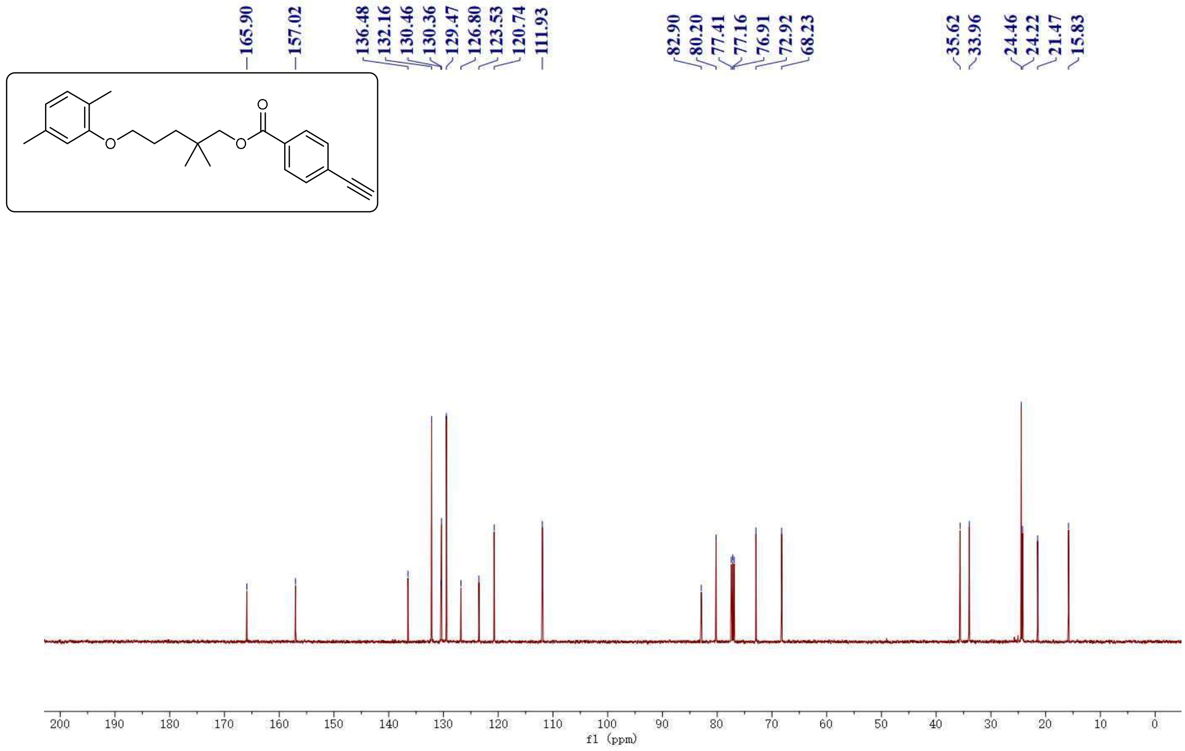

^{1}H NMR spectrum of **1ae** (500 MHz, Chloroform-*d*)

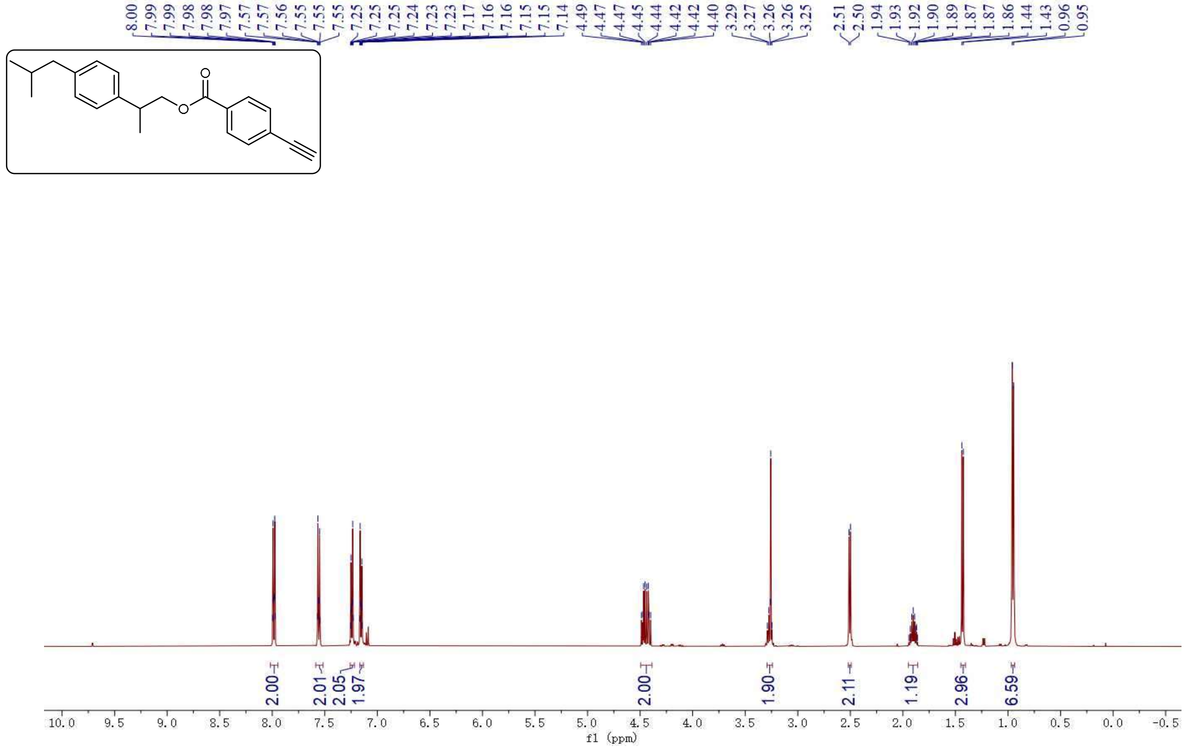

^{13}C NMR spectrum of **1ae** (126 MHz, Chloroform-*d*)

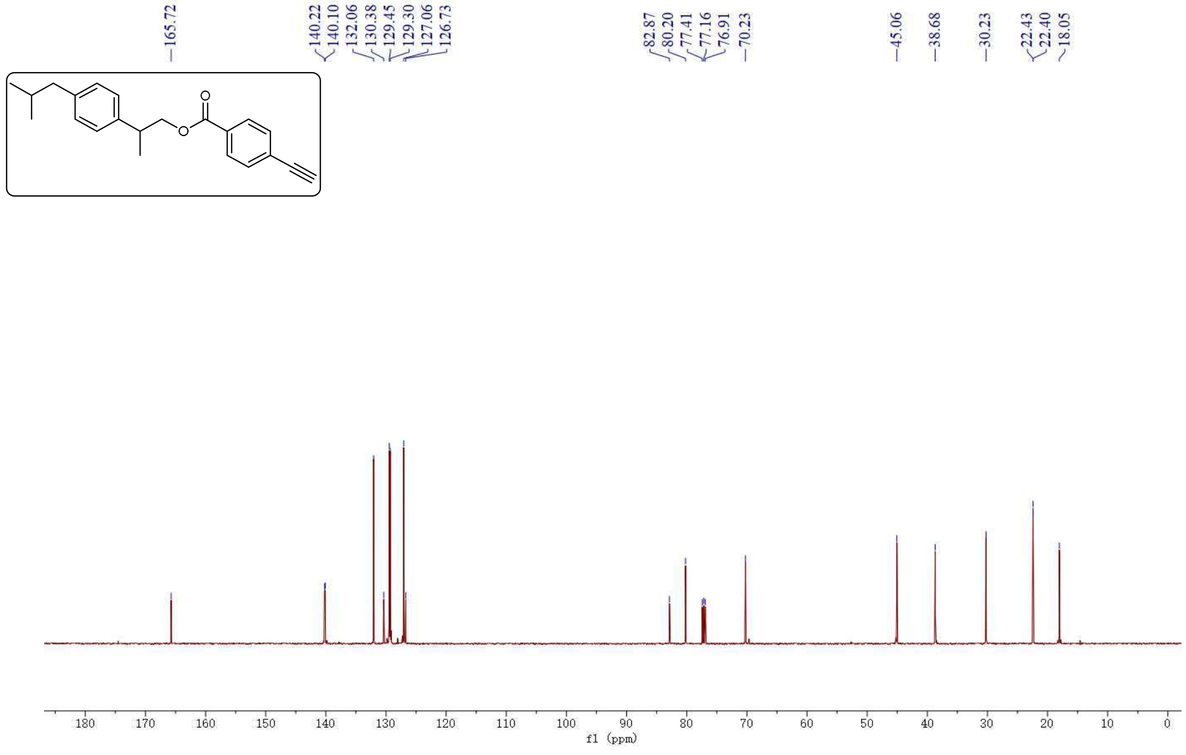

^{1}H NMR spectrum of **4** (500 MHz, Chloroform-*d*)

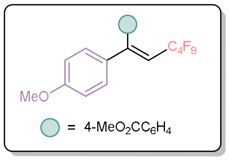

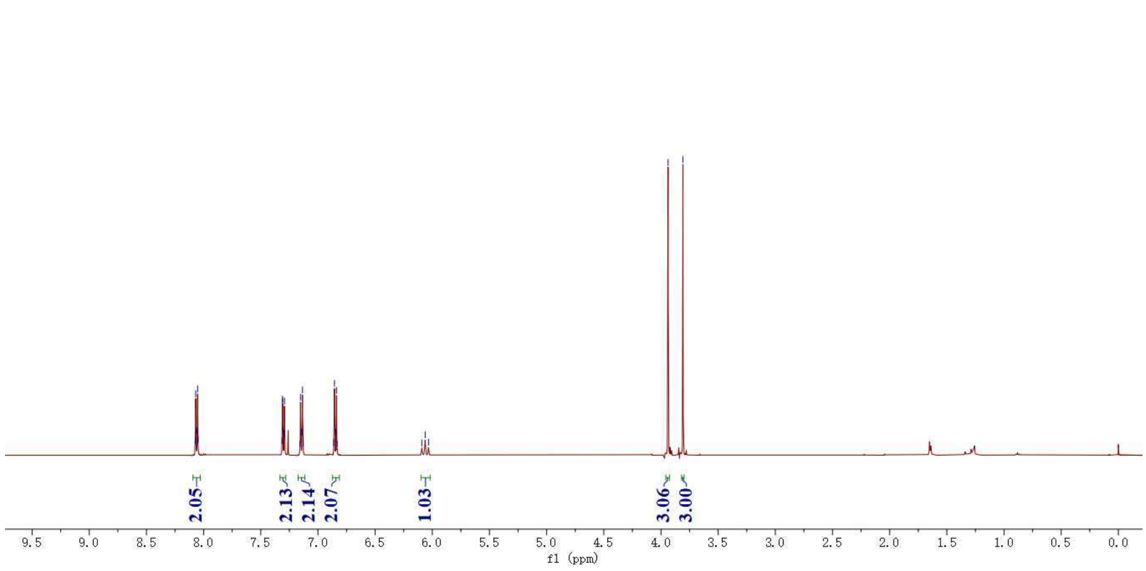

^{19}F NMR Spectrum of **4** (471 MHz, Chloroform-*d*)

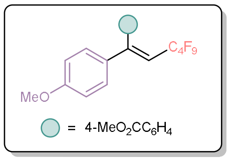

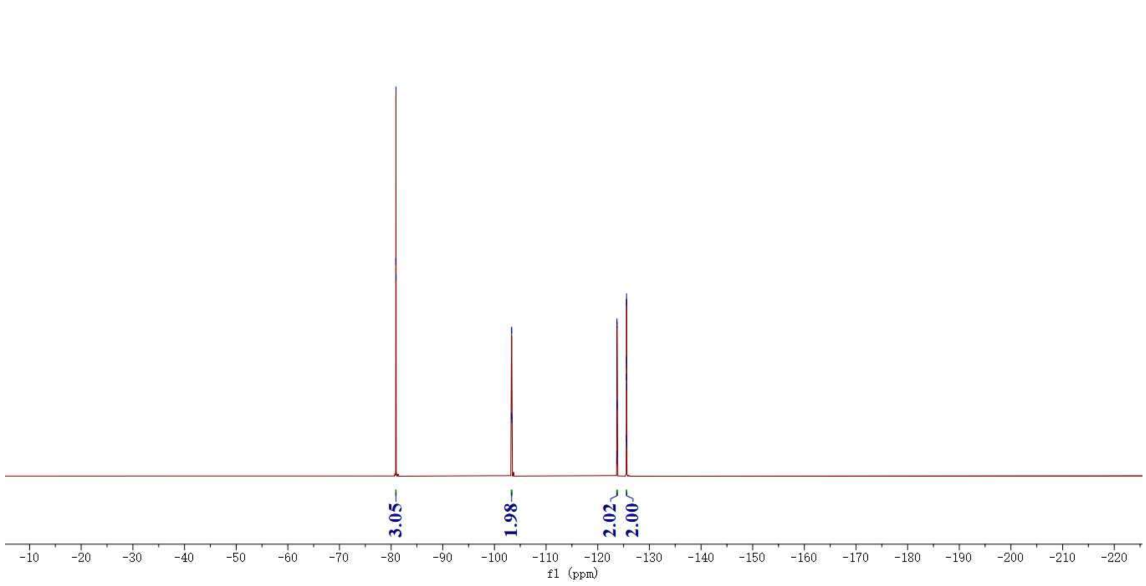

^{13}**C NMR** spectrum of **4** (126 MHz, Chloroform-*d*)

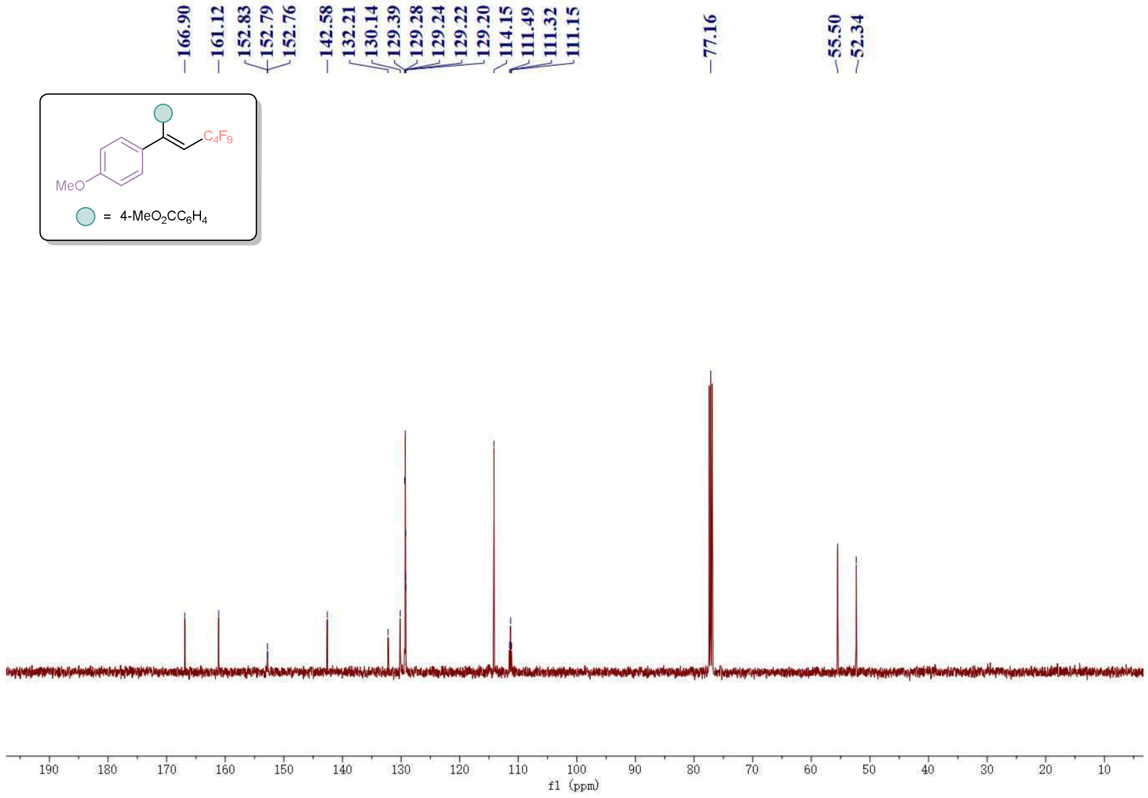

^{1}**H NMR** spectrum of **5** (500 MHz, Chloroform-*d*)

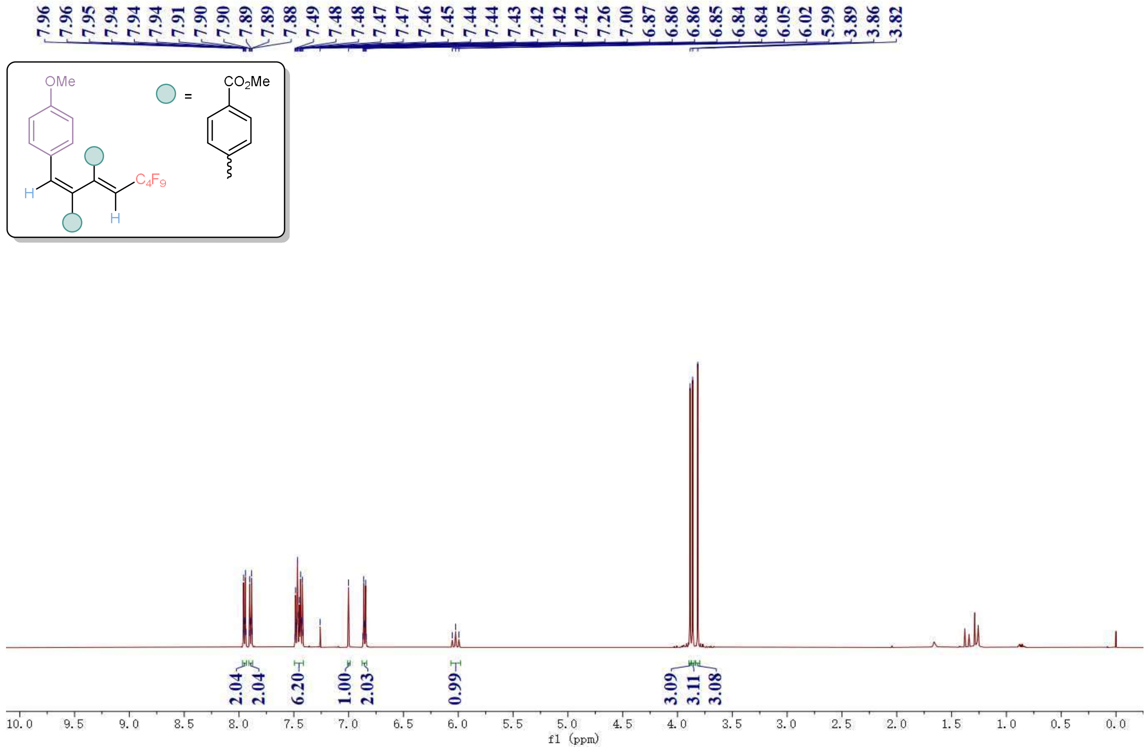

^{19}F **NMR** Spectrum of **5** (471 MHz, Chloroform-*d*)

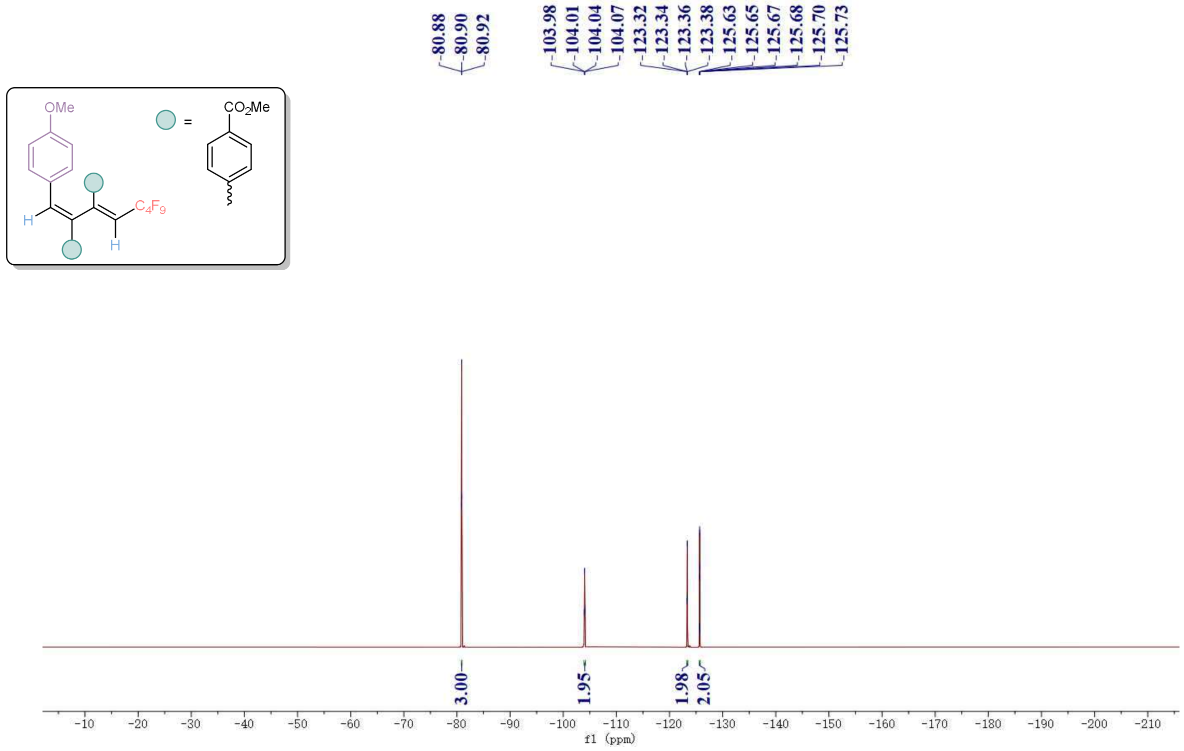

^{13}C **NMR** spectrum of **5** (126 MHz, Chloroform-*d*)

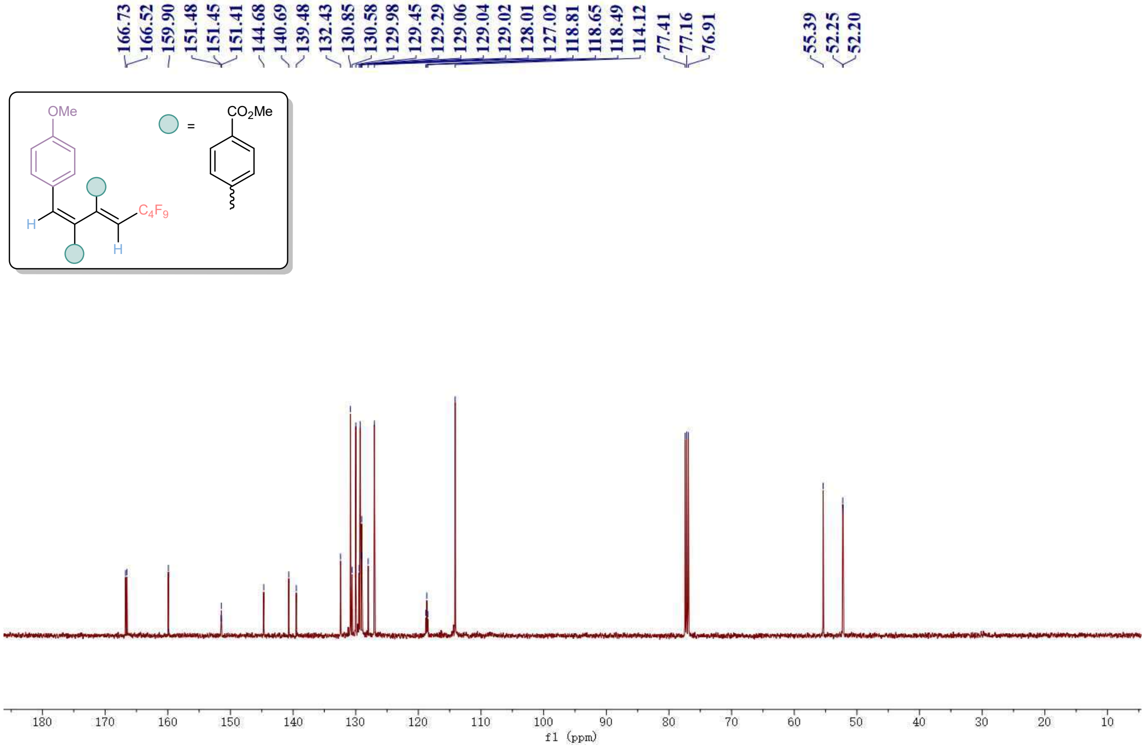

^{1}H NMR spectrum of **6** (500 MHz, Chloroform-*d*)

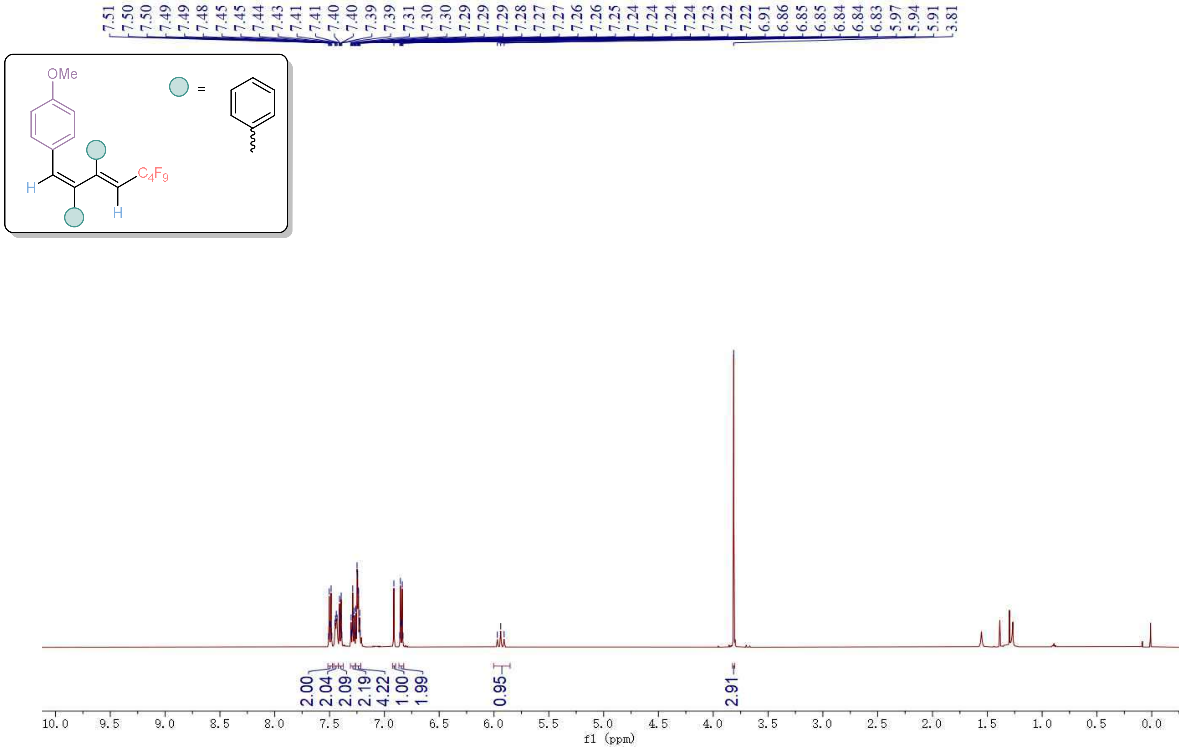

^{19}F NMR Spectrum of **6** (471 MHz, Chloroform-*d*)

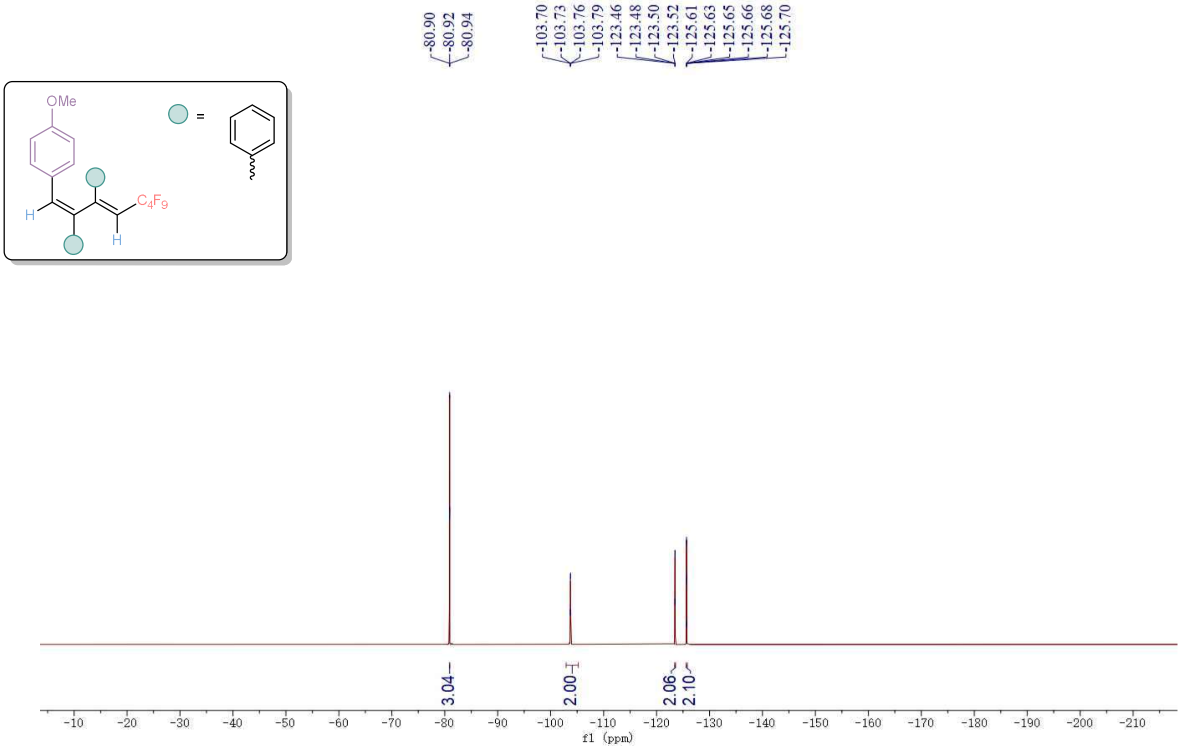

^{13}C **NMR** spectrum of **6** (126 MHz, Chloroform-*d*)

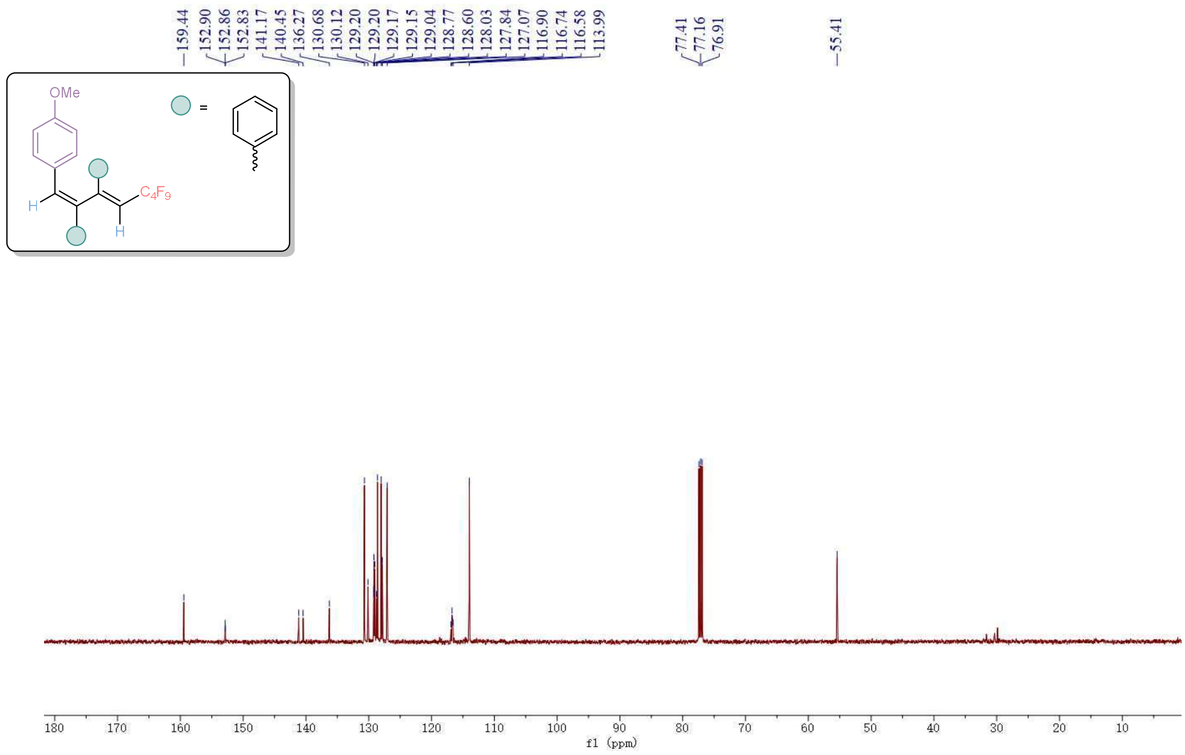

^{1}H **NMR** spectrum of **7** (500 MHz, Chloroform-*d*)

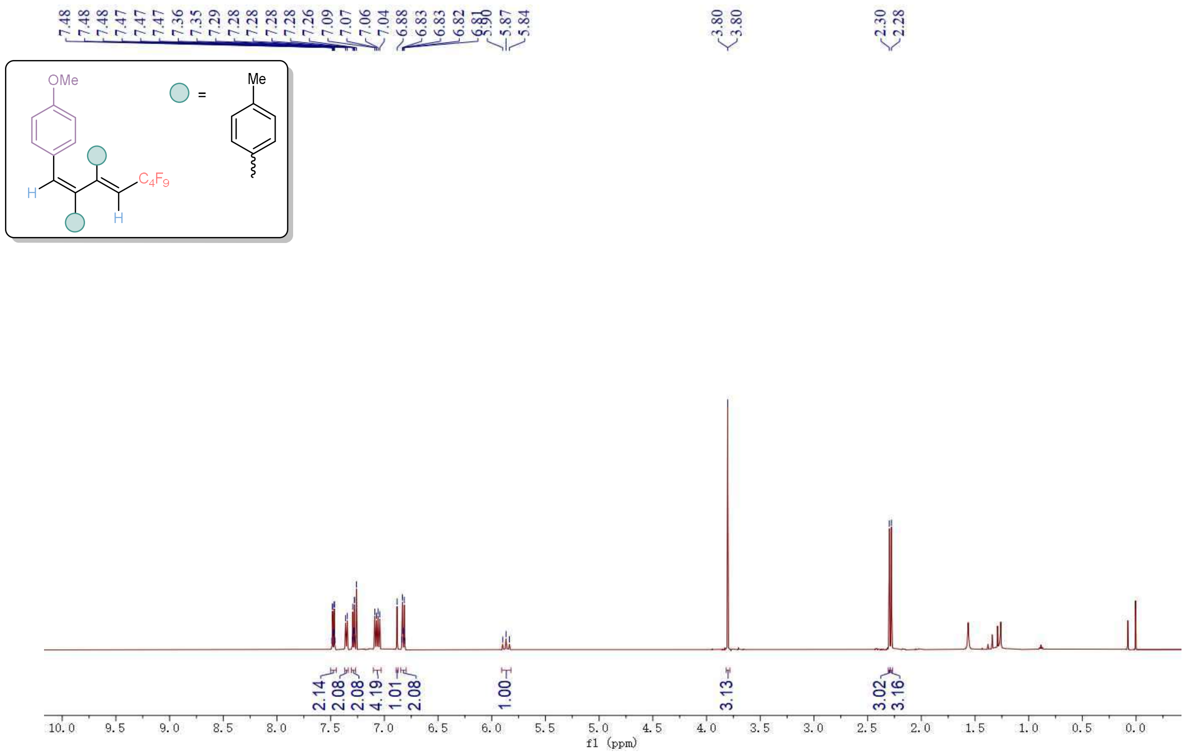

[19]**F NMR** Spectrum of **7** (471 MHz, Chloroform-*d*)

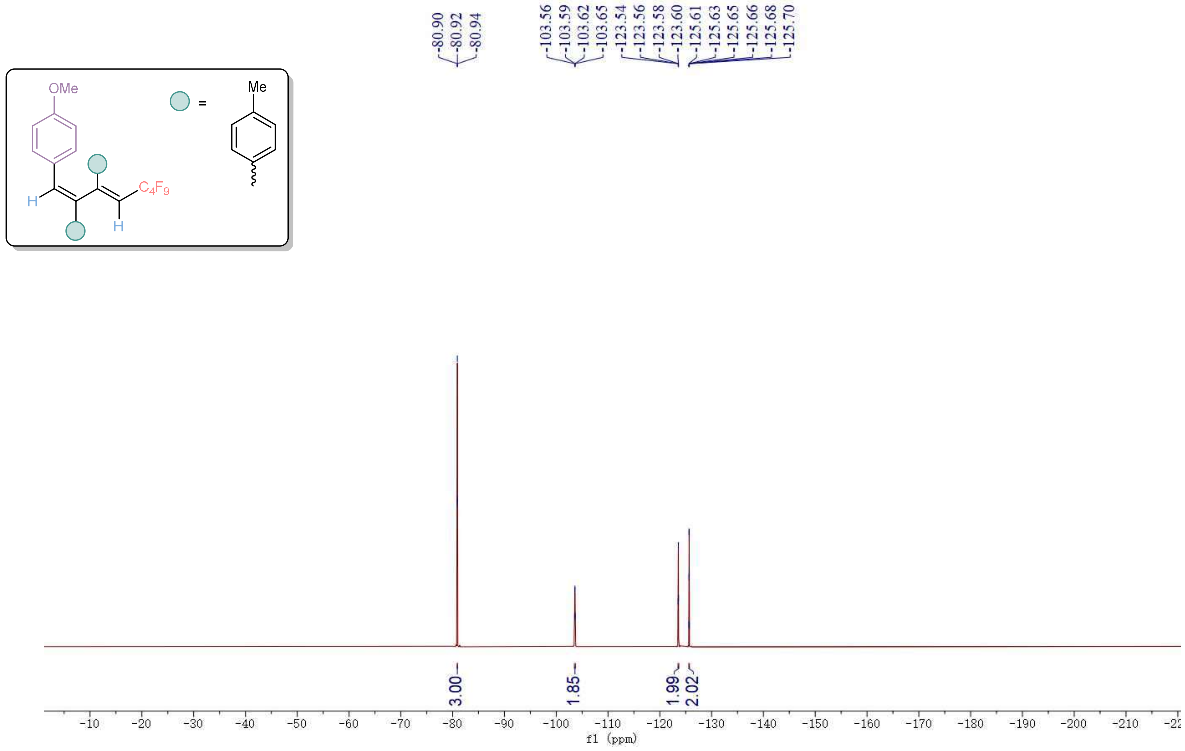

[13]**C NMR** spectrum of **7** (126 MHz, Chloroform-*d*)

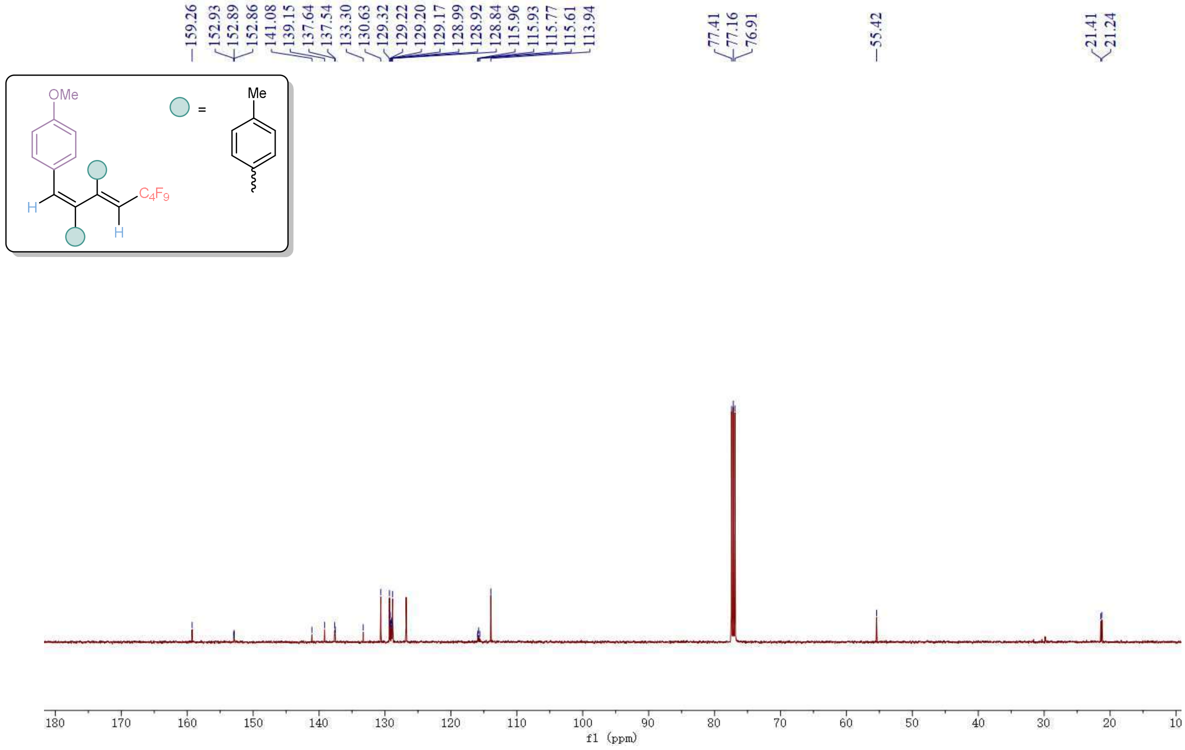

[1]**H NMR** spectrum of **8** (500 MHz, Chloroform-*d*)

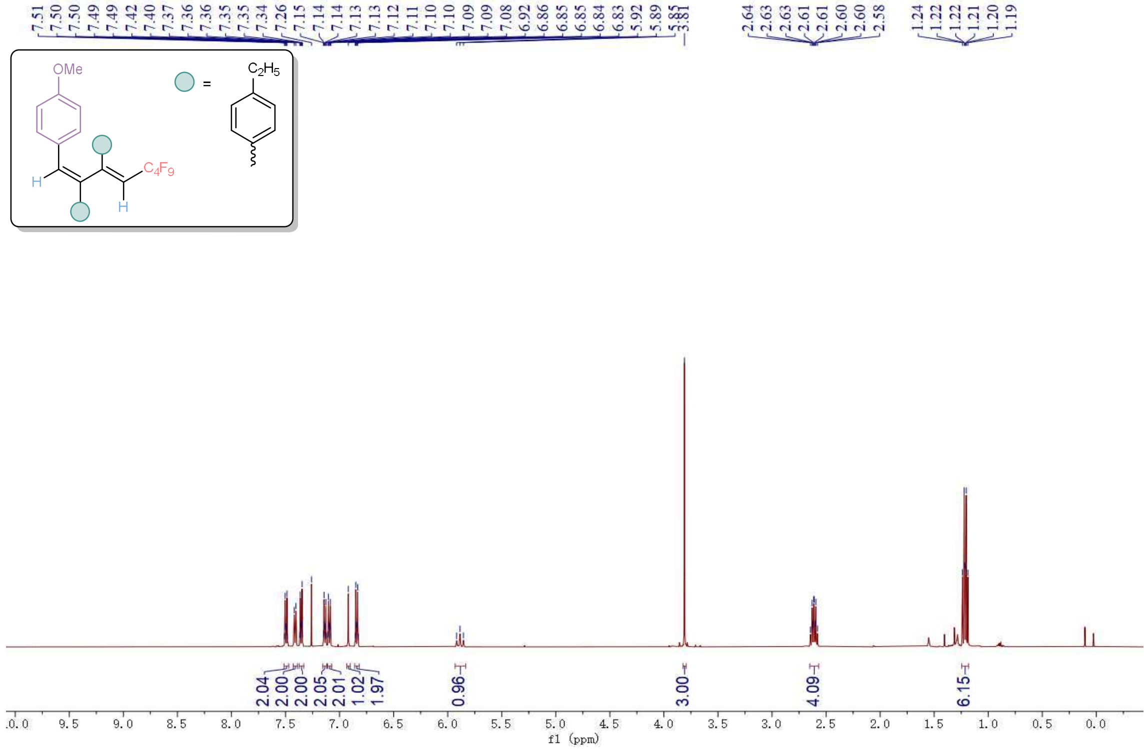

[19]**F NMR** Spectrum of **8** (471 MHz, Chloroform-*d*)

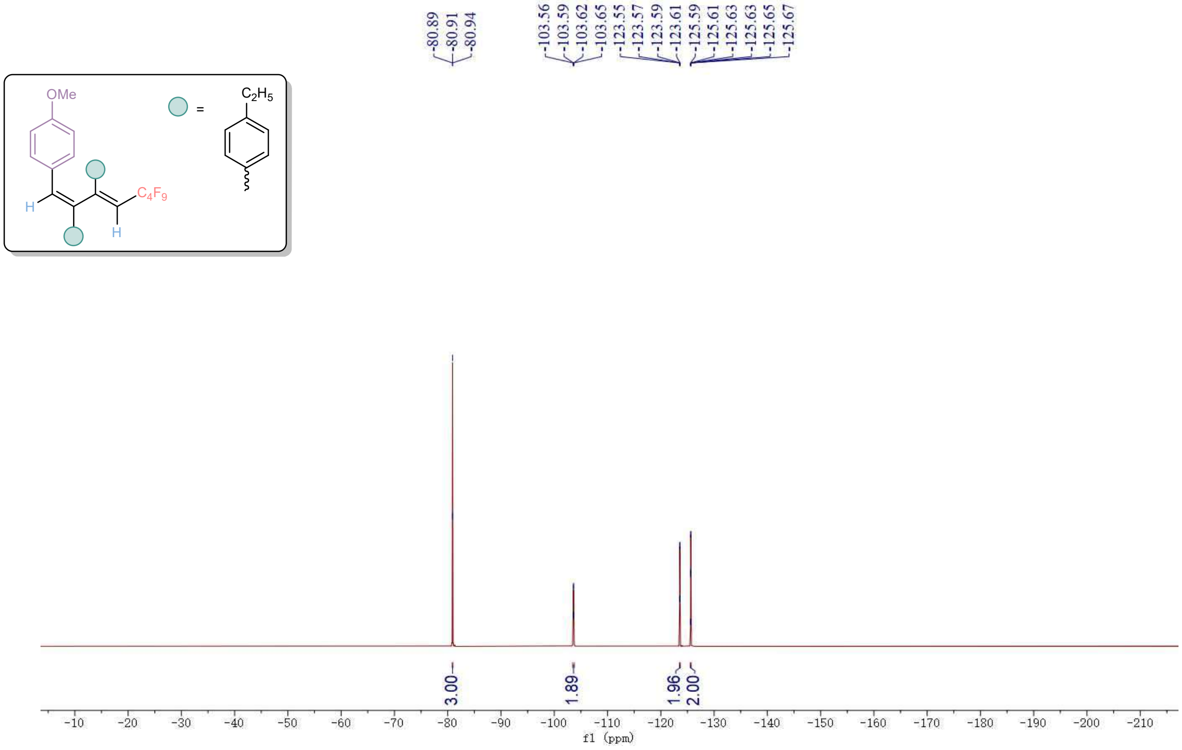

^{13}**C NMR** spectrum of **8** (126 MHz, Chloroform-*d*)

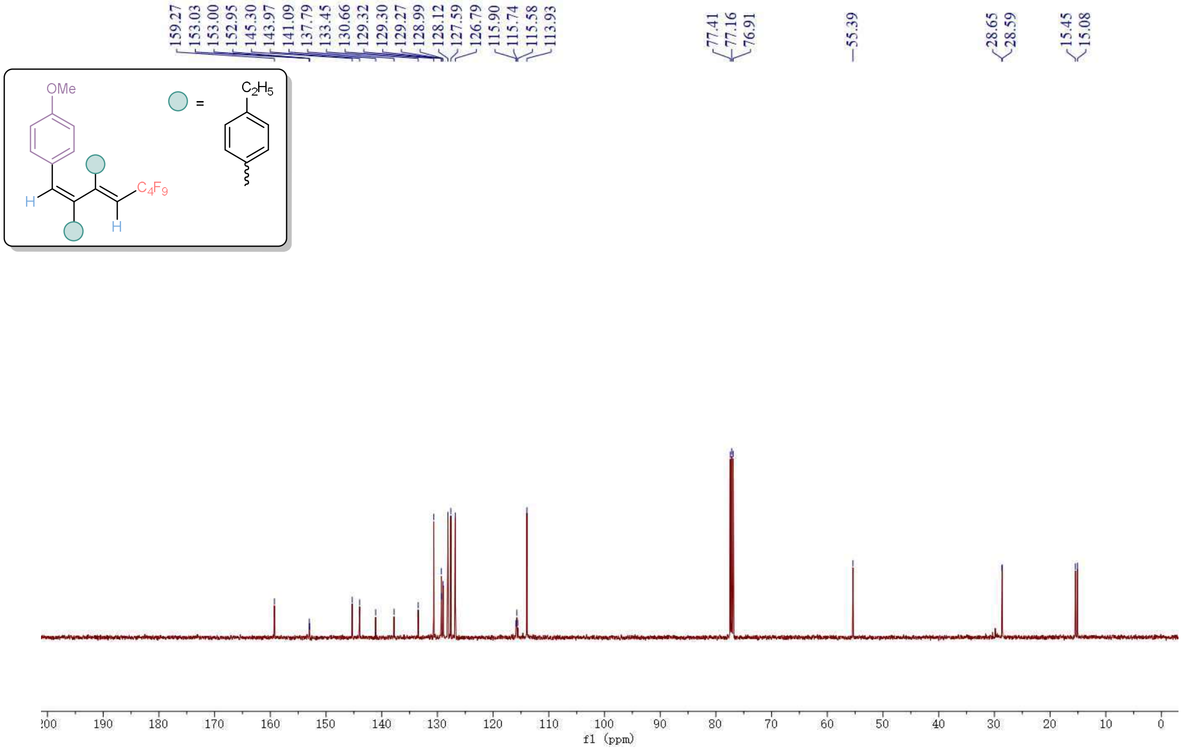

^{1}**H NMR** spectrum of **9** (500 MHz, Chloroform-*d*)

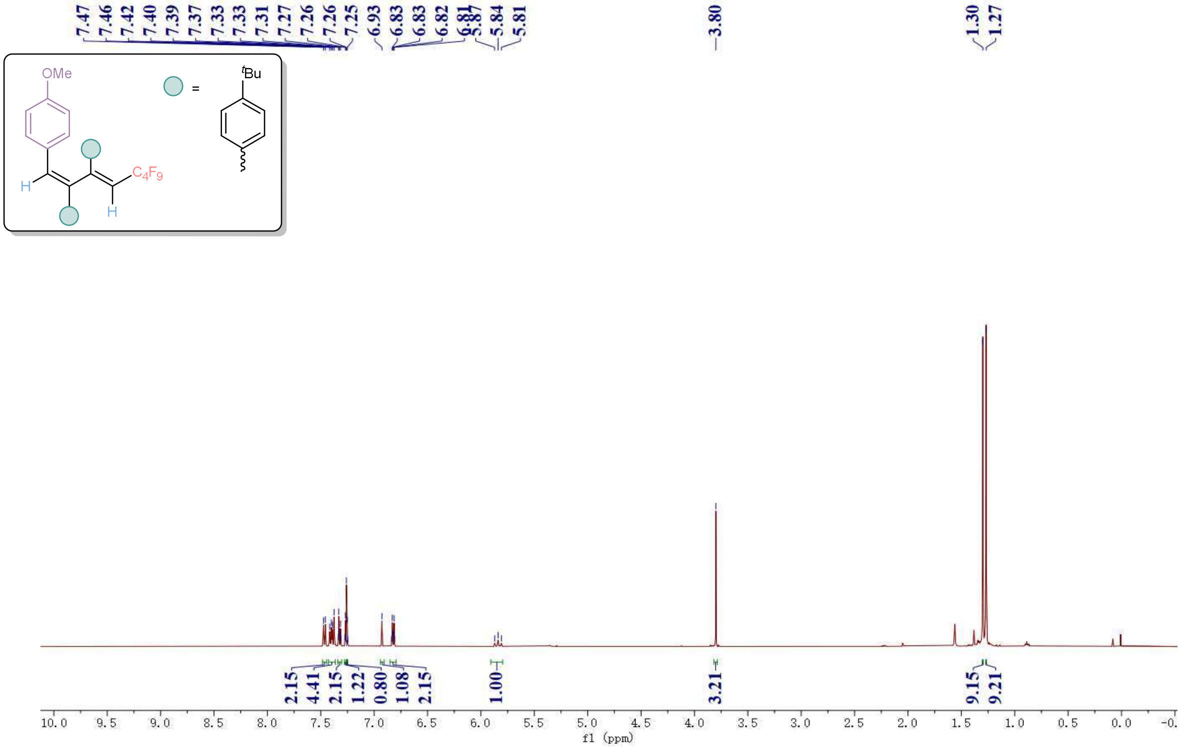

[19]**F NMR** Spectrum of **9** (471 MHz, Chloroform-*d*)

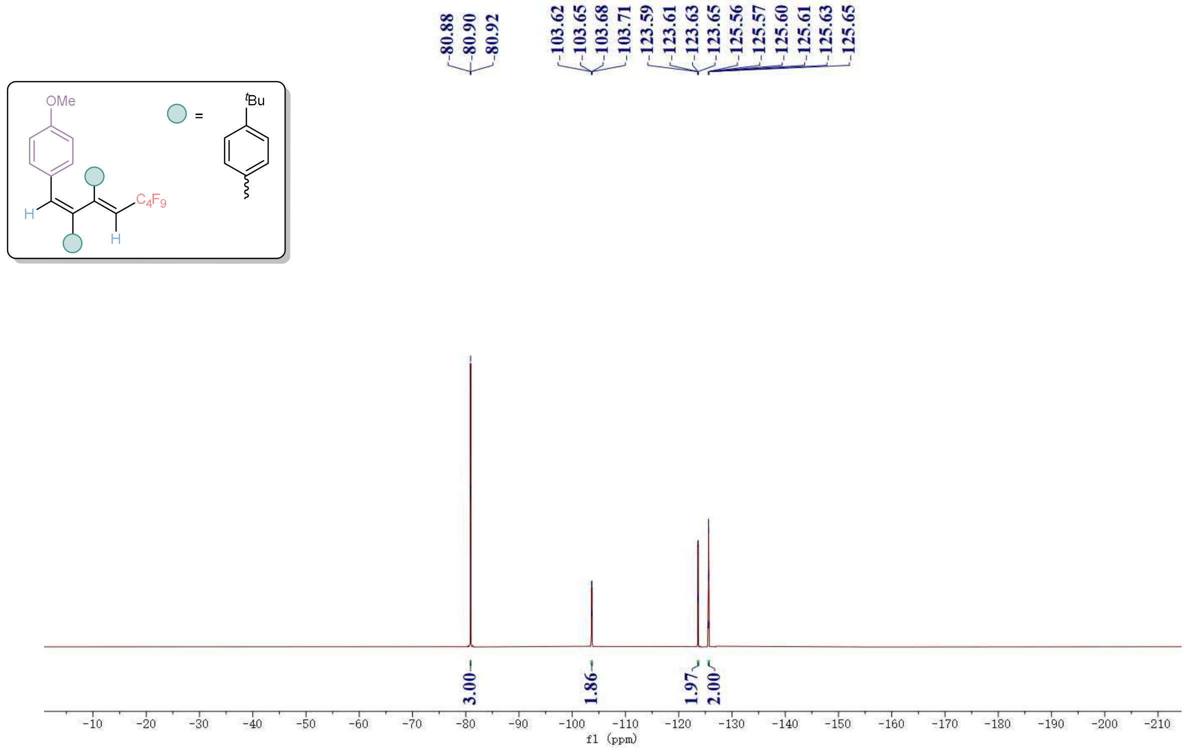

[13]**C NMR** spectrum of **9** (126 MHz, Chloroform-*d*)

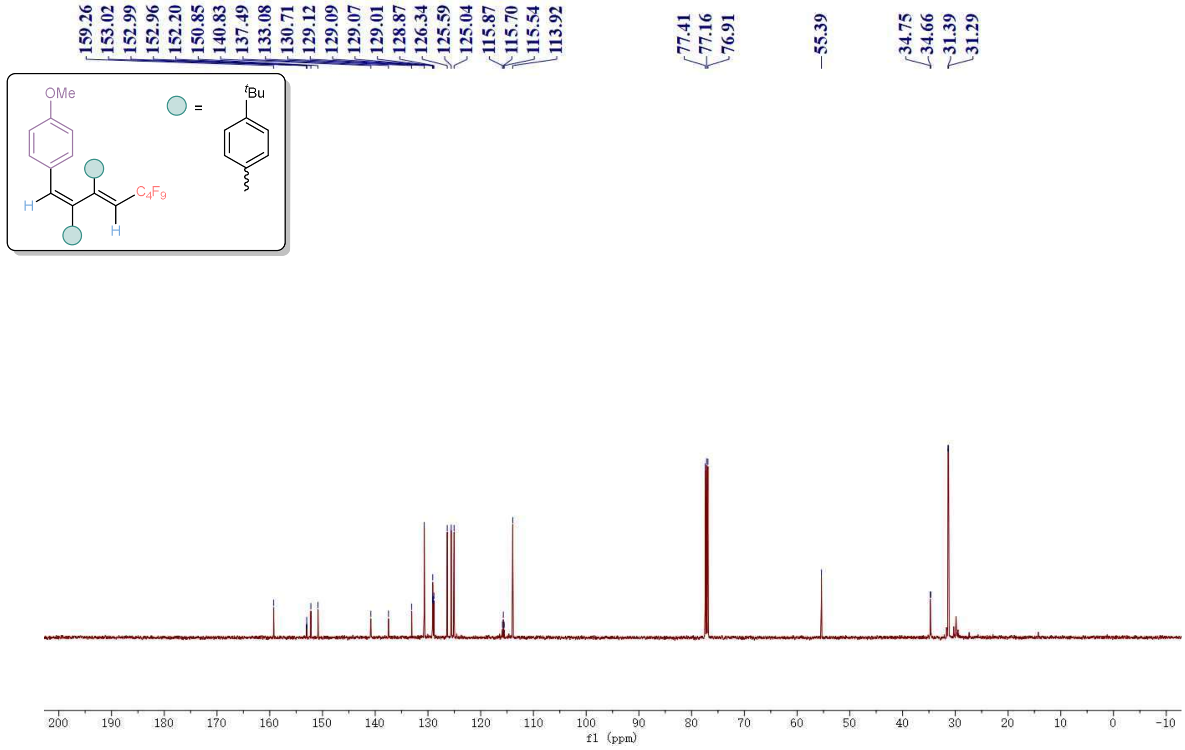

^{1}H NMR spectrum of **10** (500 MHz, Chloroform-*d*)

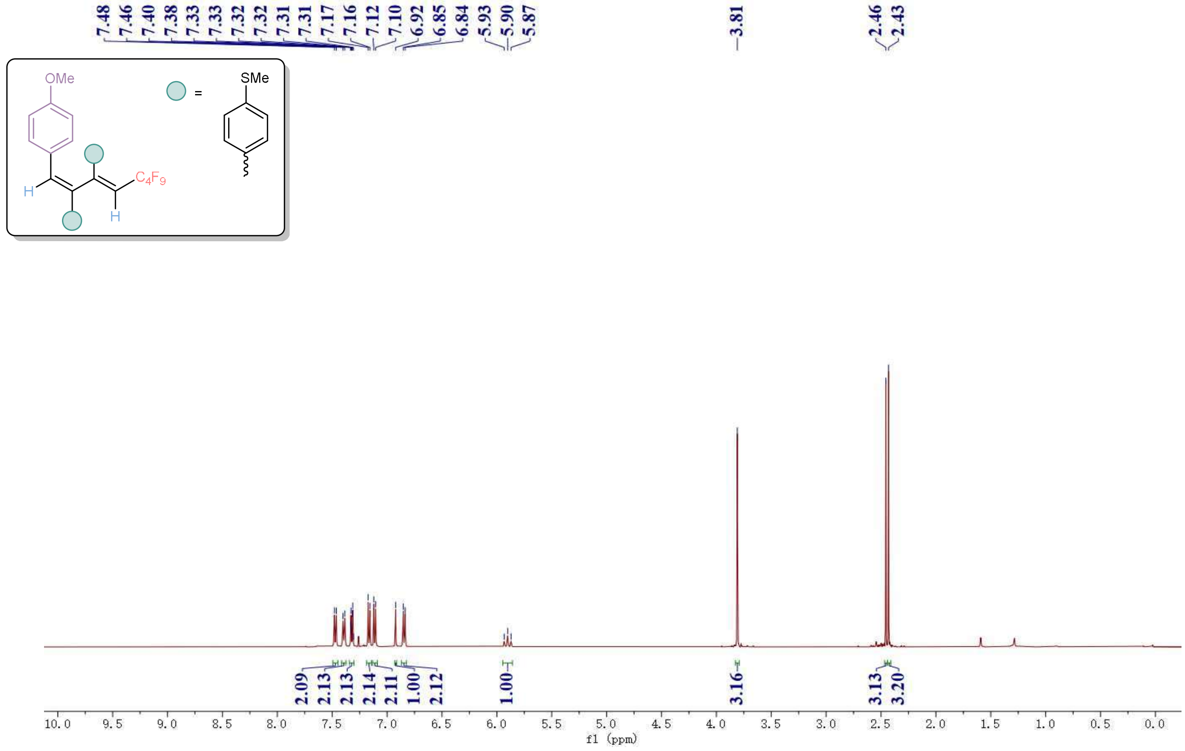

^{19}F NMR Spectrum of **10** (471 MHz, Chloroform-*d*)

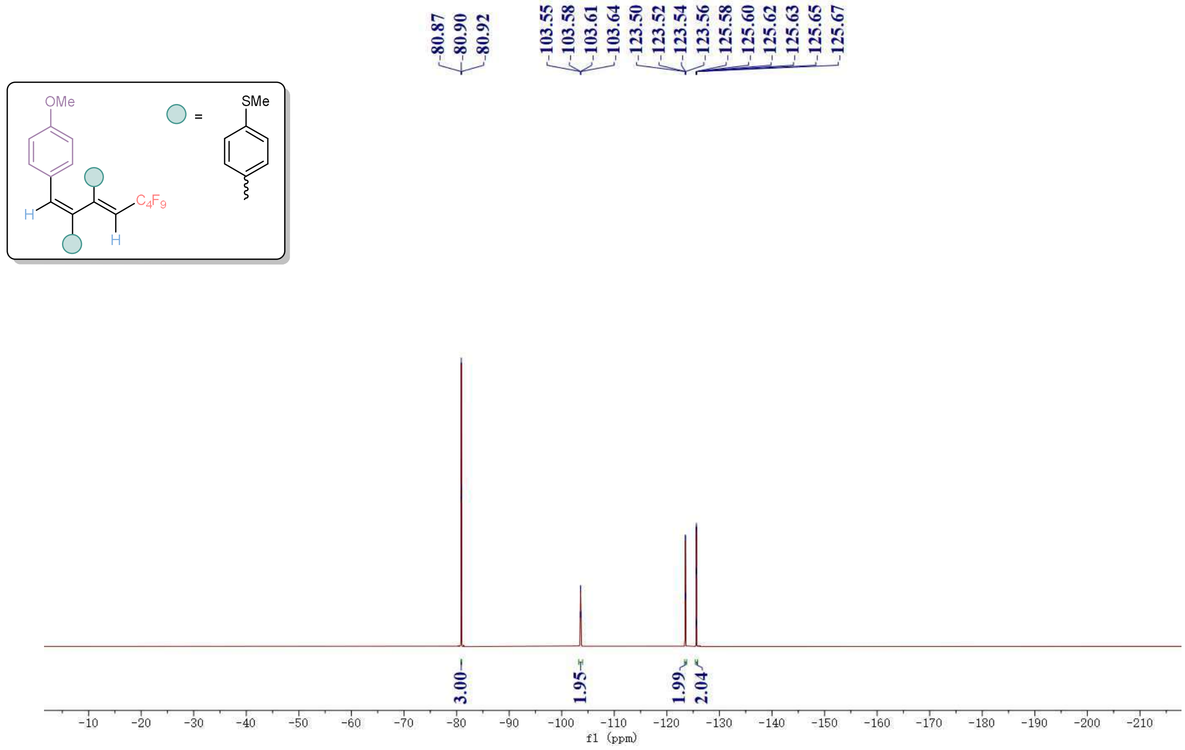

^{13}C NMR spectrum of **10** (126 MHz, Chloroform-d)

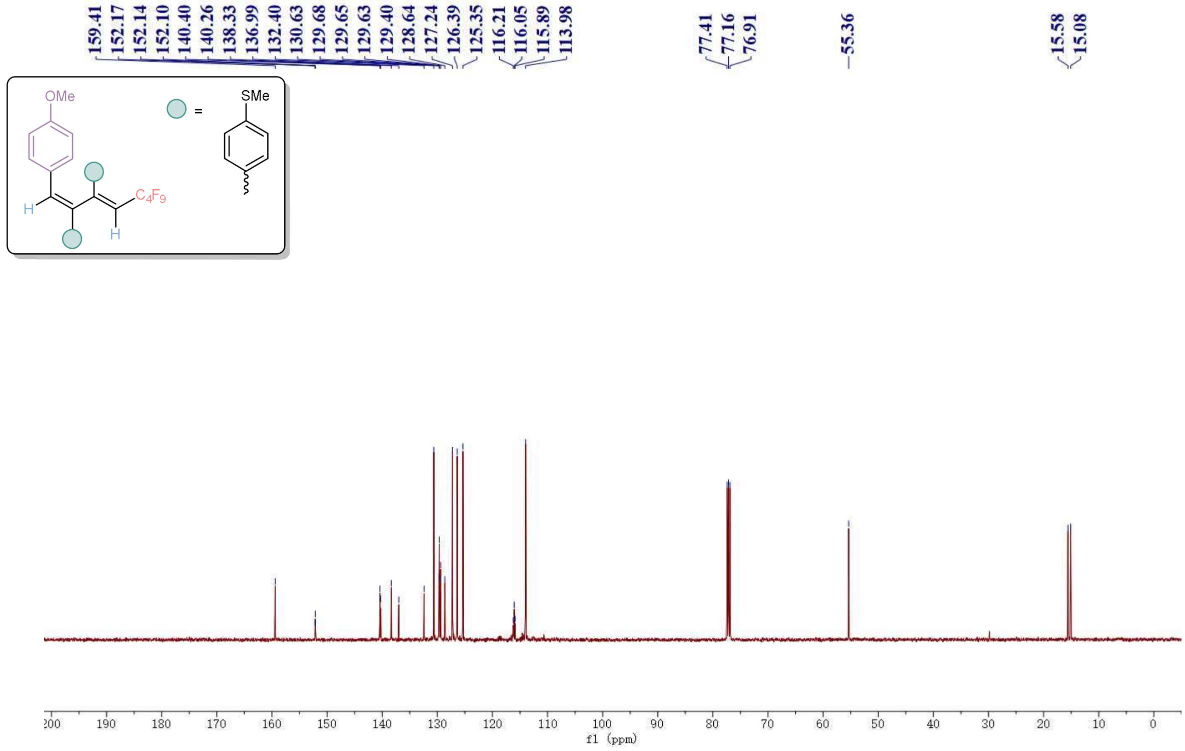

^{1}H NMR spectrum of **11** (500 MHz, Chloroform-*d*)

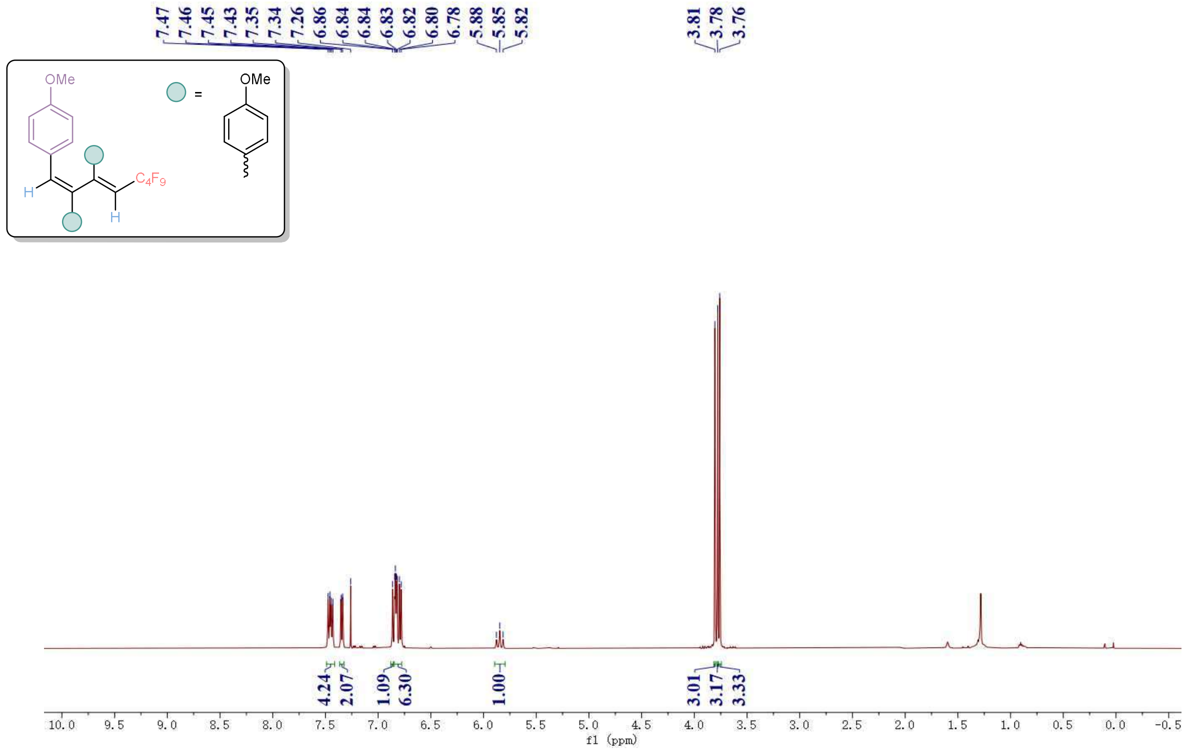

^{19}**F NMR** Spectrum of **11** (471 MHz, Chloroform-*d*)

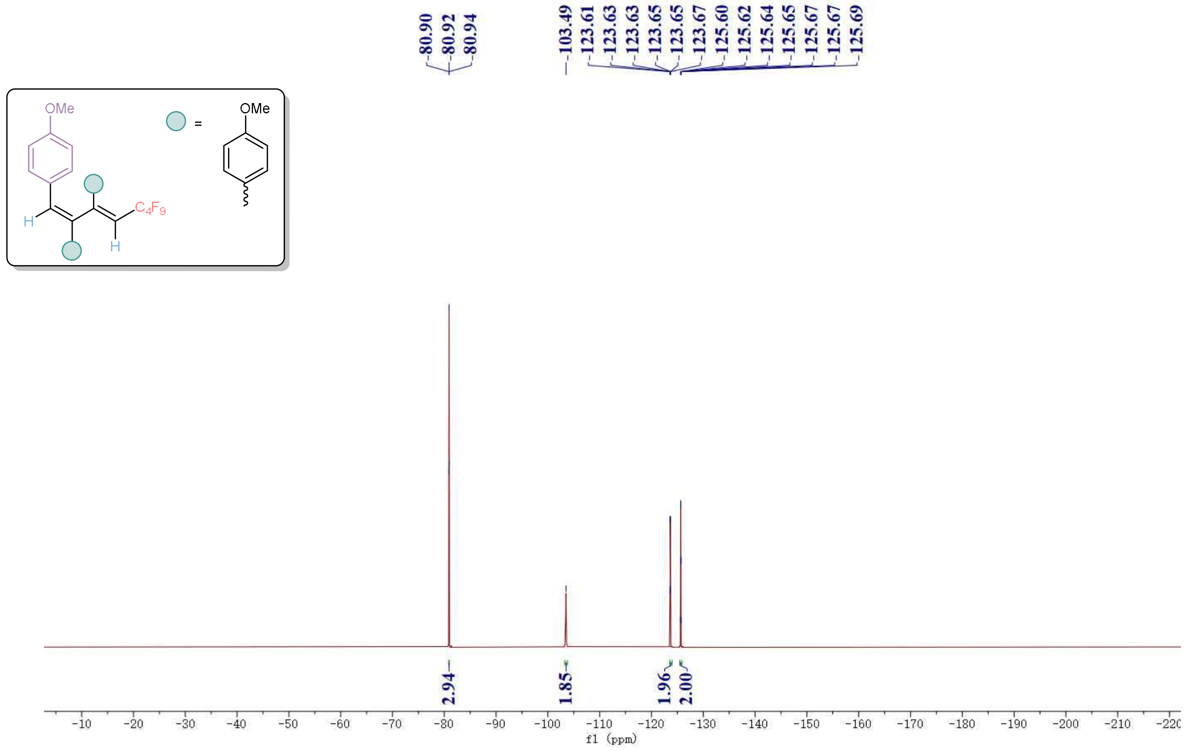

^{13}**C NMR** spectrum of **11** (126 MHz, Chloroform-*d*)

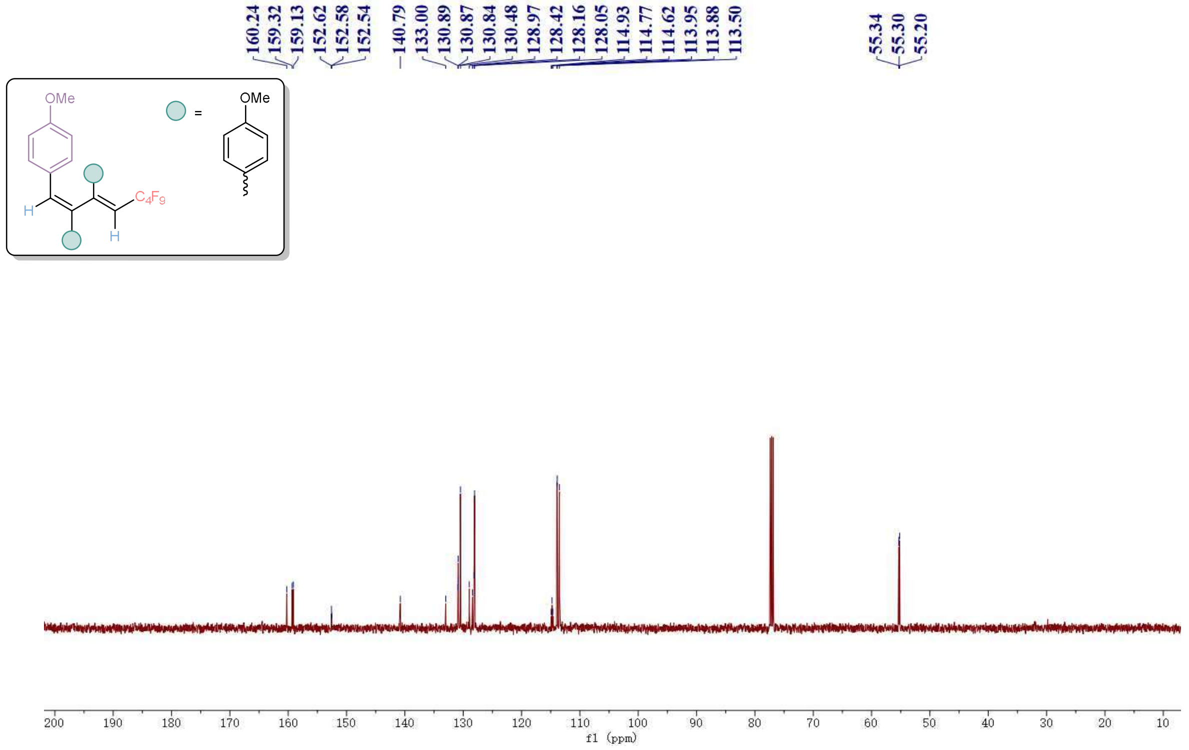

^{1}H NMR spectrum of 12 (500 MHz, Chloroform-*d*)

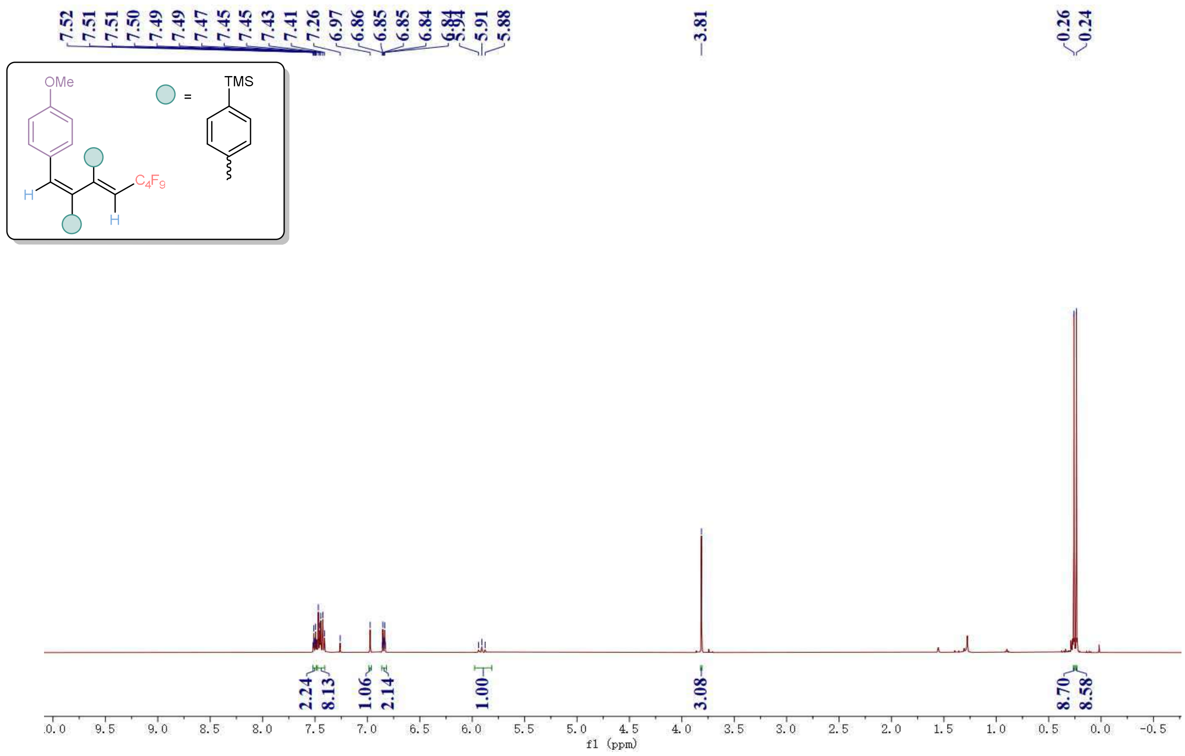

^{19}F NMR Spectrum of 12 (471 MHz, Chloroform-*d*)

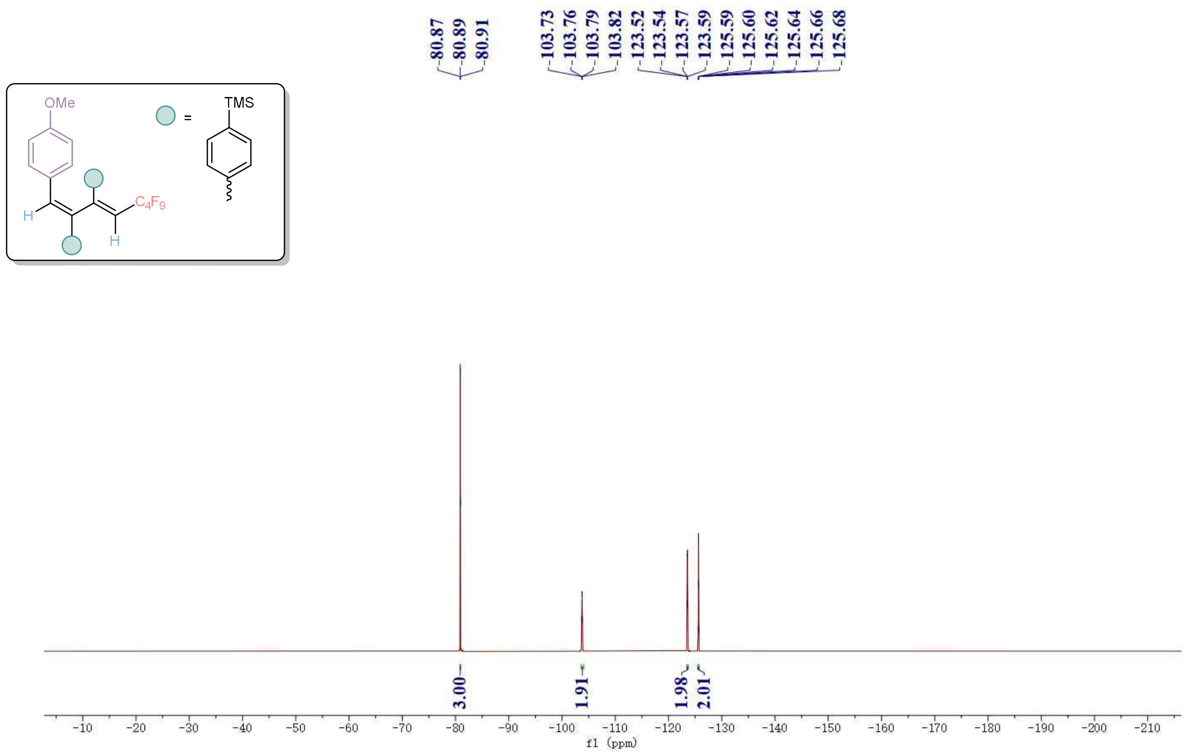

^{13}**C NMR** spectrum of **12** (126 MHz, Chloroform-*d*)

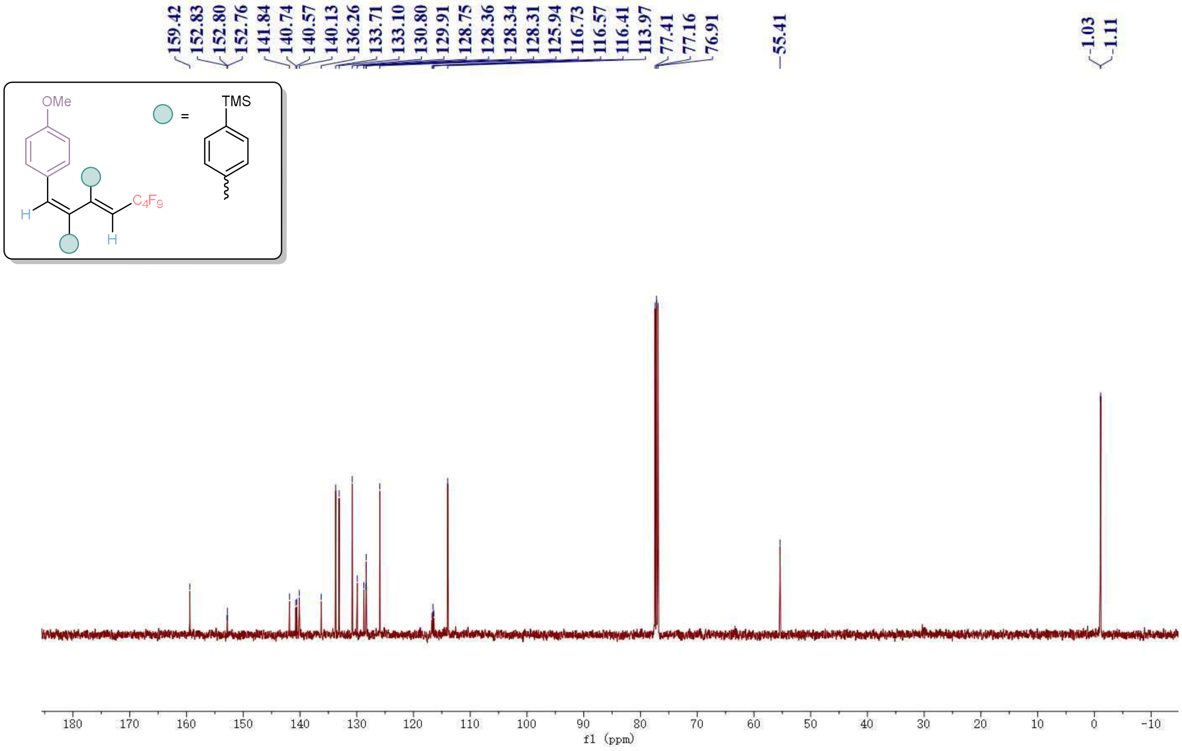

^{1}**H NMR** spectrum of **13** (500 MHz, Chloroform-*d*)

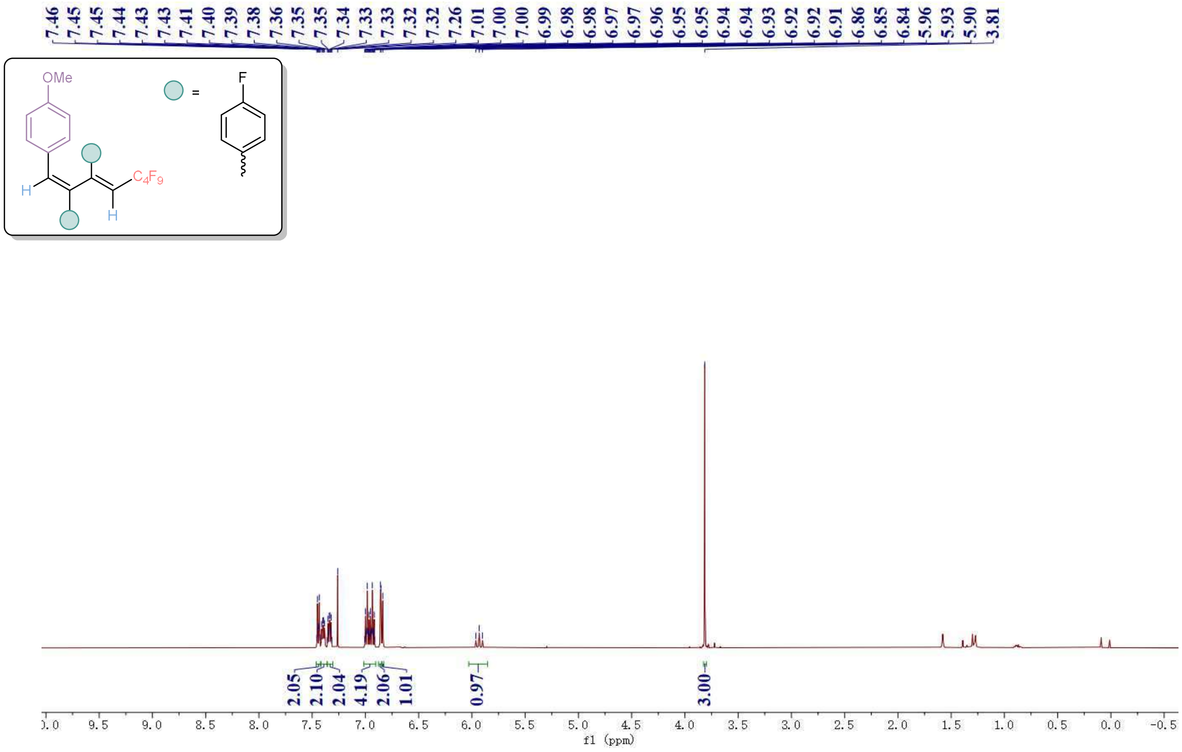

^{19}F **NMR** Spectrum of **13** (471 MHz, Chloroform-*d*)

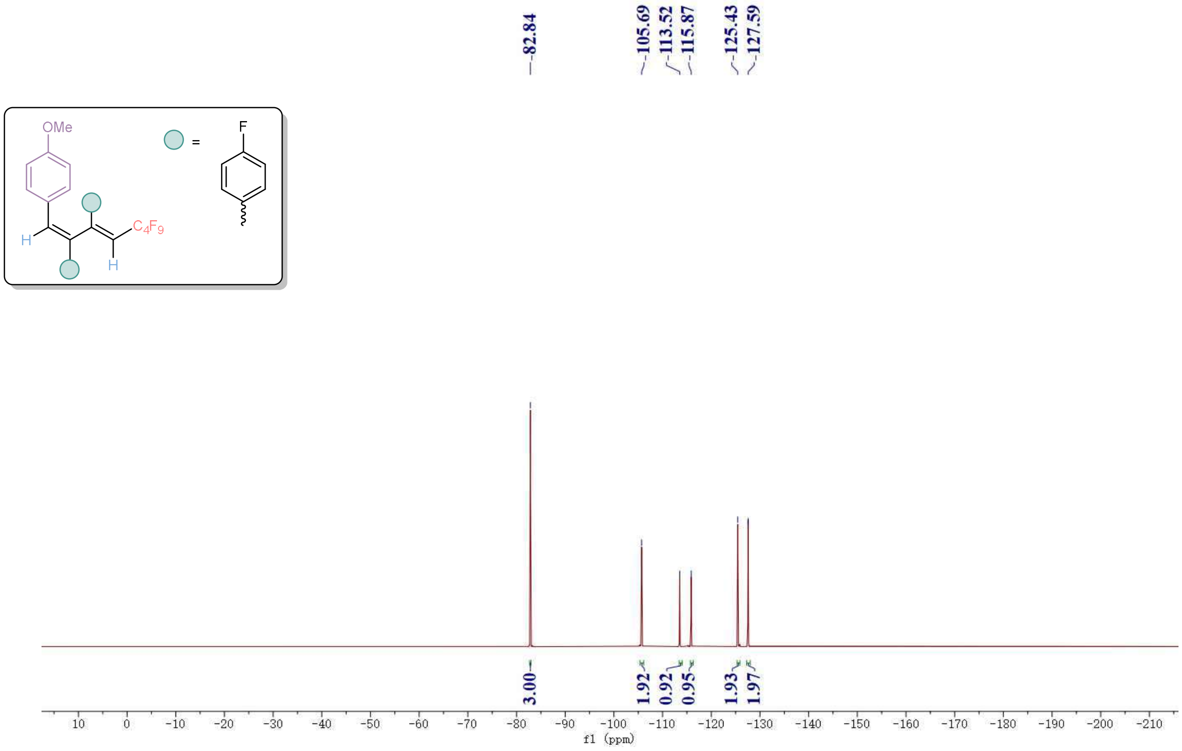

^{13}C **NMR** spectrum of **13** (126 MHz, Chloroform-*d*)

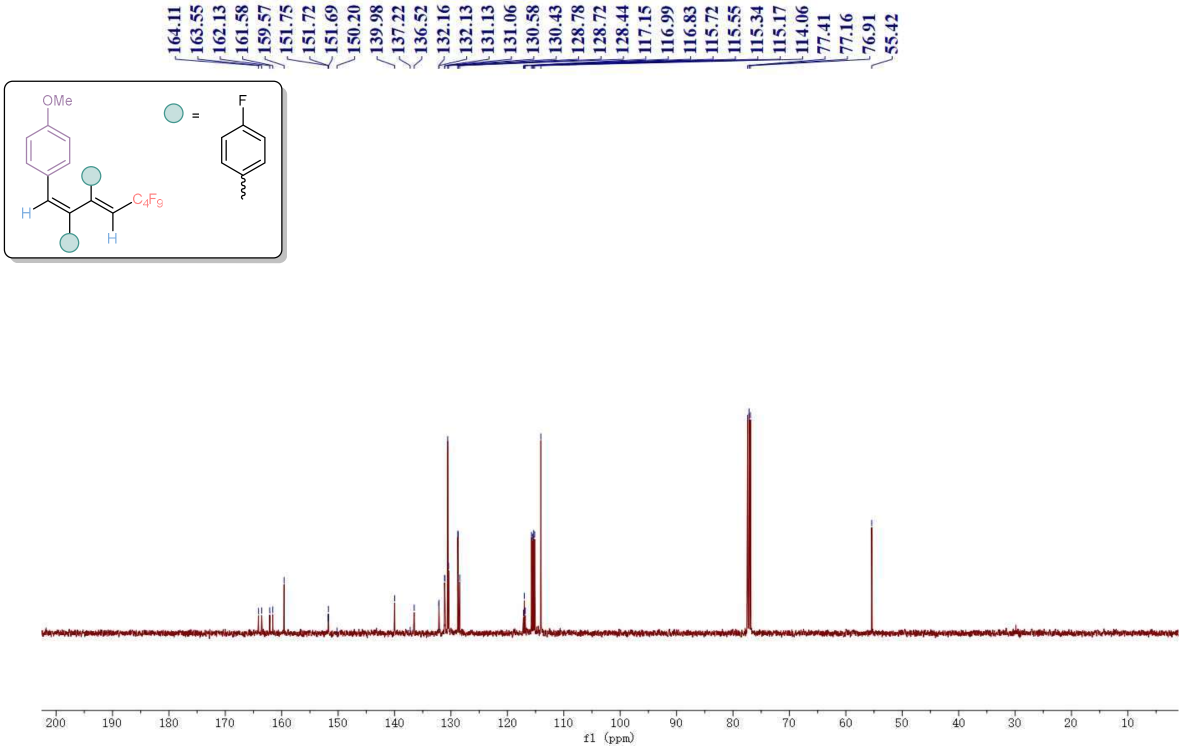

^{1}H NMR spectrum of **14** (500 MHz, Chloroform-*d*)

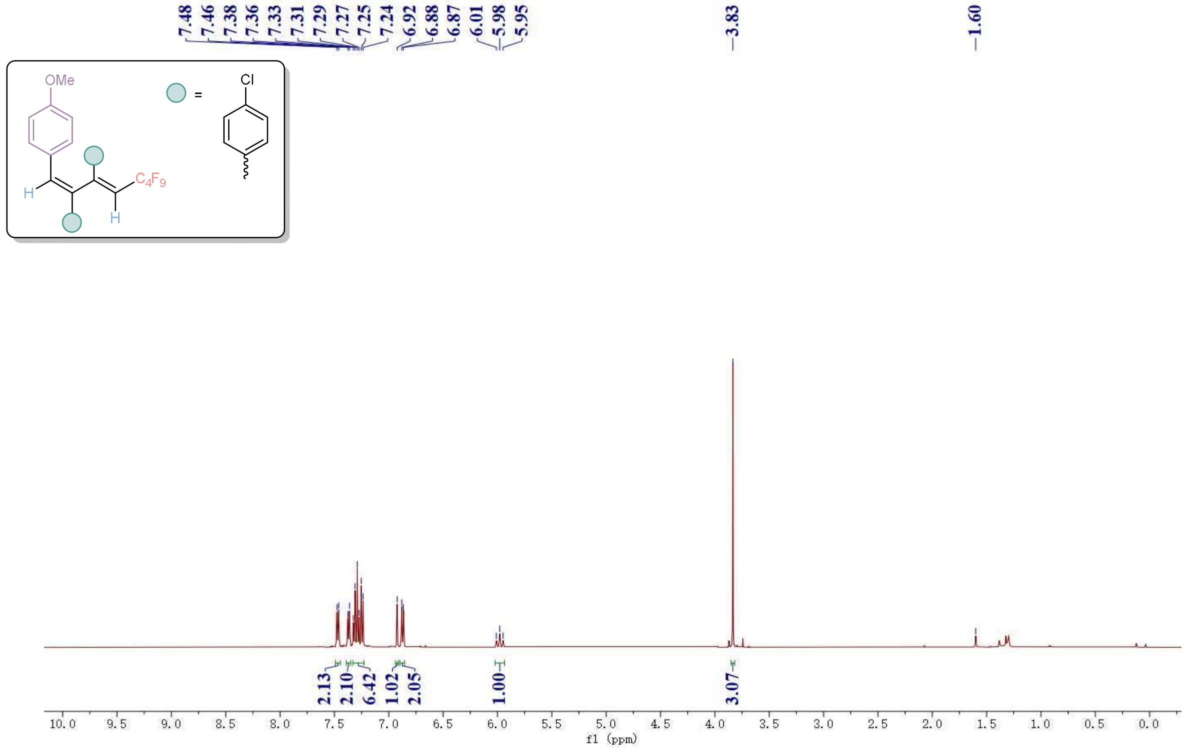

^{19}F NMR Spectrum of **14** (471 MHz, Chloroform-*d*)

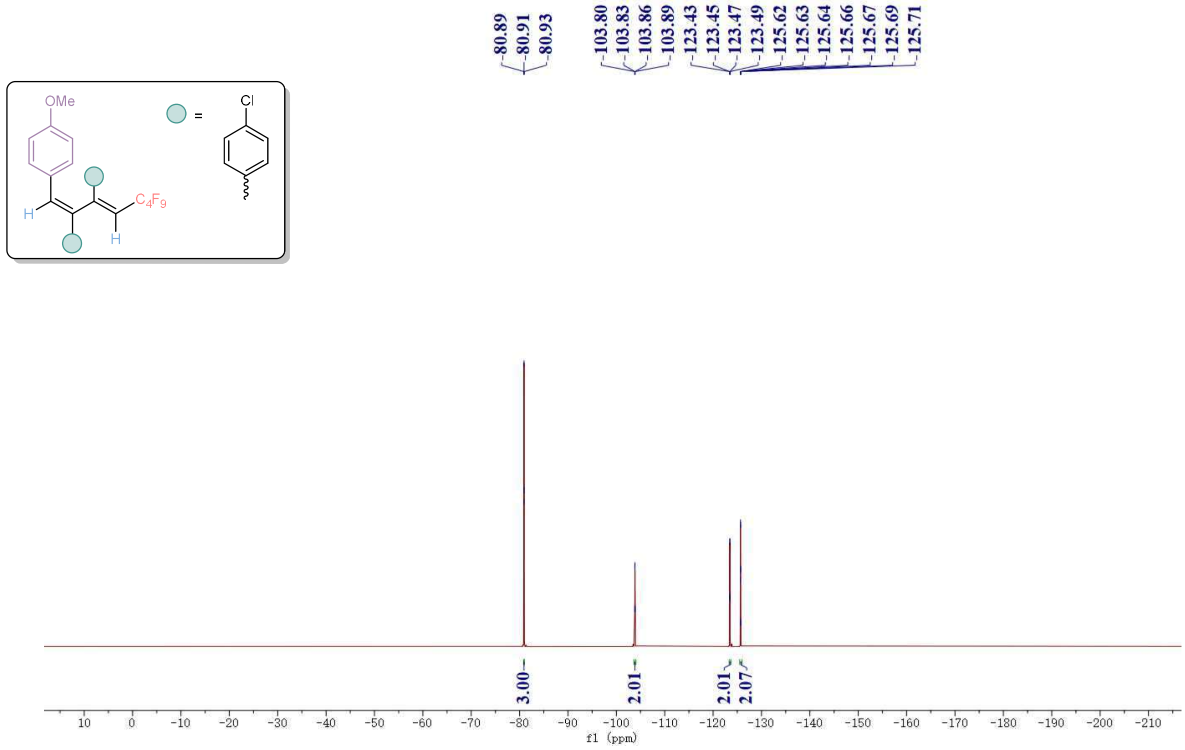

^{13}C NMR spectrum of **14** (126 MHz, Chloroform-*d*)

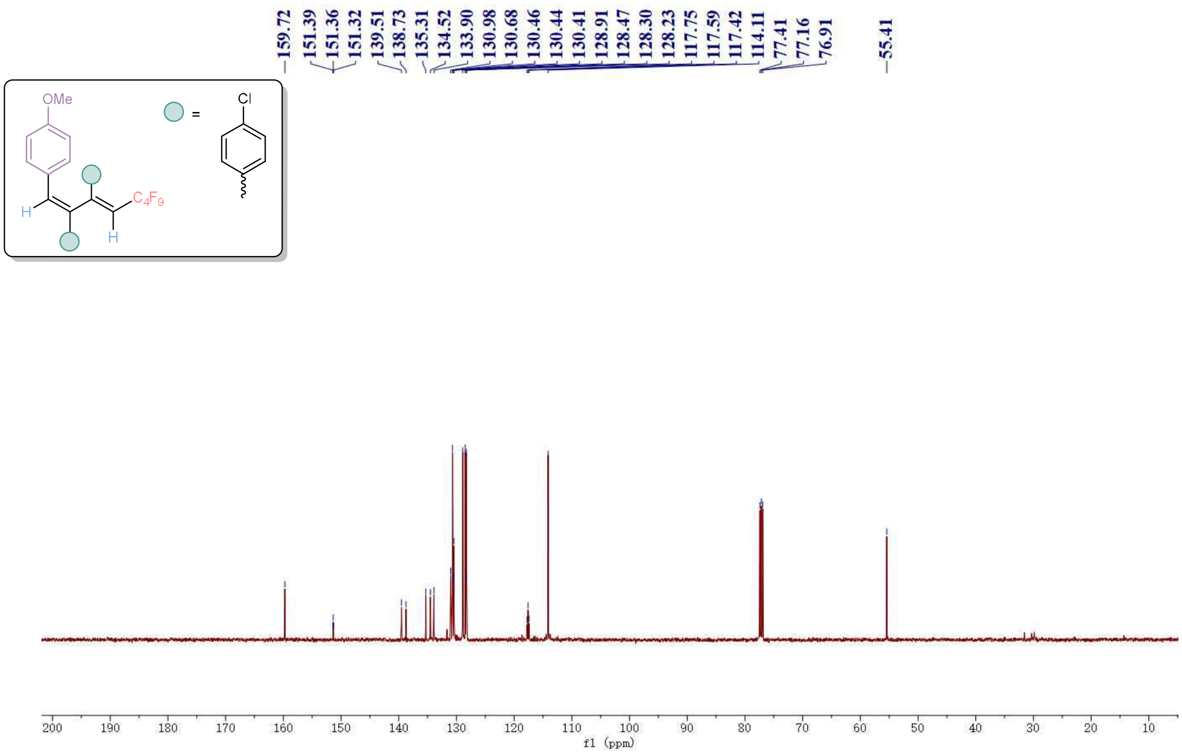

^{1}H NMR spectrum of **15** (500 MHz, Chloroform-*d*)

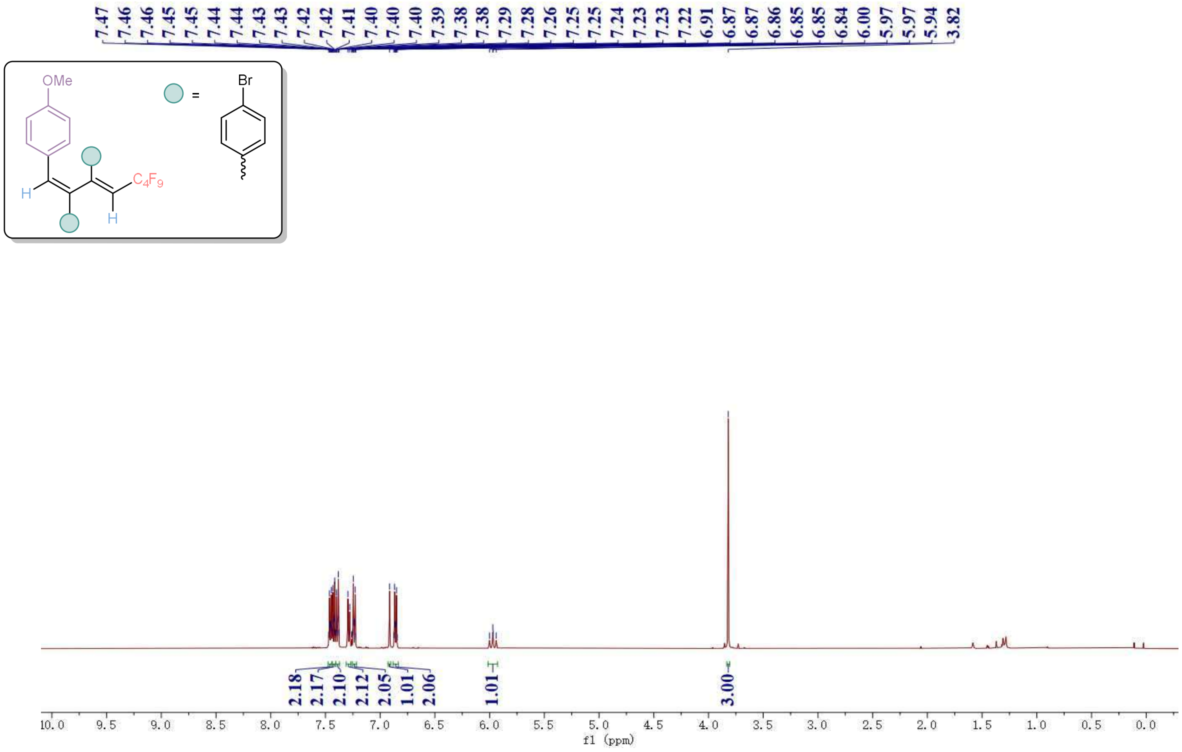

^{19}F NMR Spectrum of **15** (471 MHz, Chloroform-*d*)

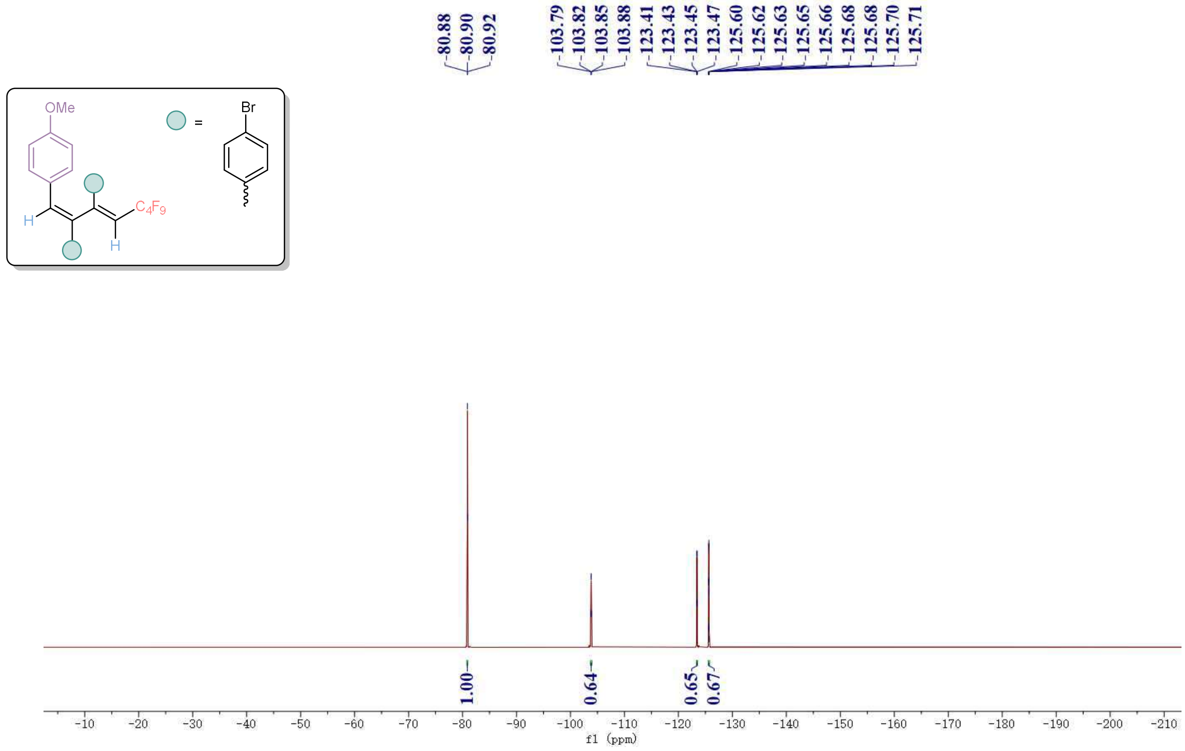

^{13}C NMR spectrum of **15** (126 MHz, Chloroform-d)

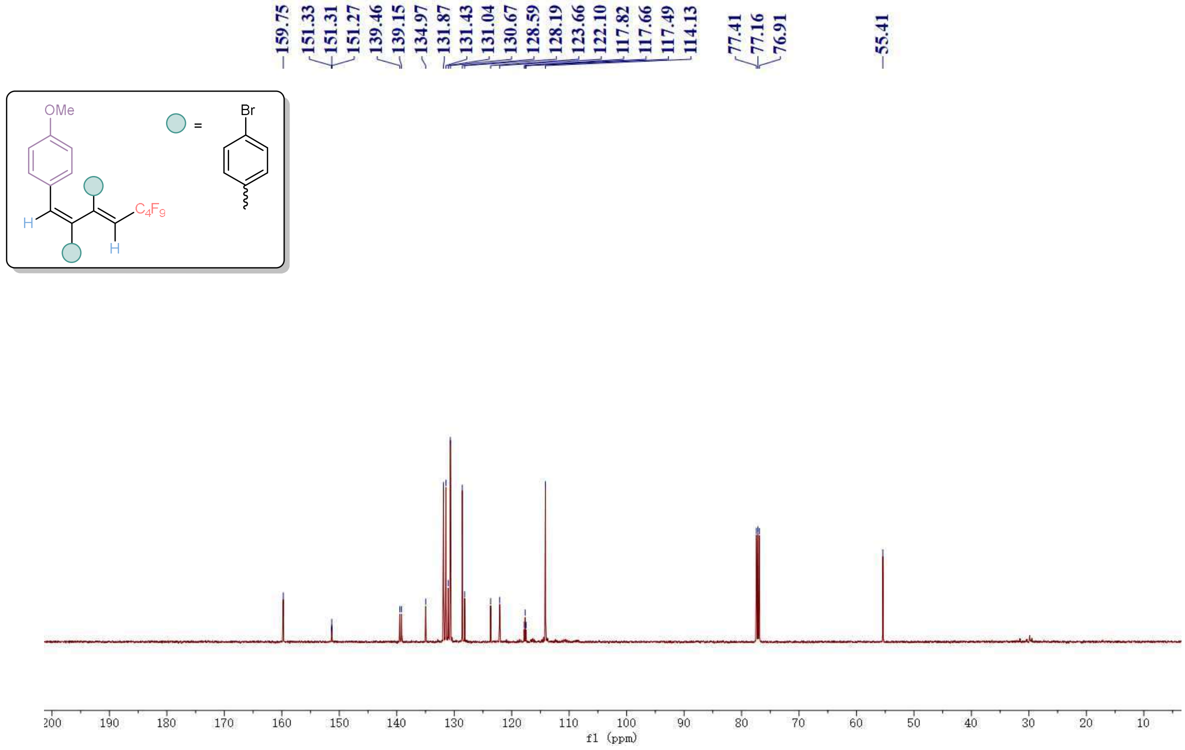

^{1}**H NMR** spectrum of **16** (500 MHz, Chloroform-*d*)

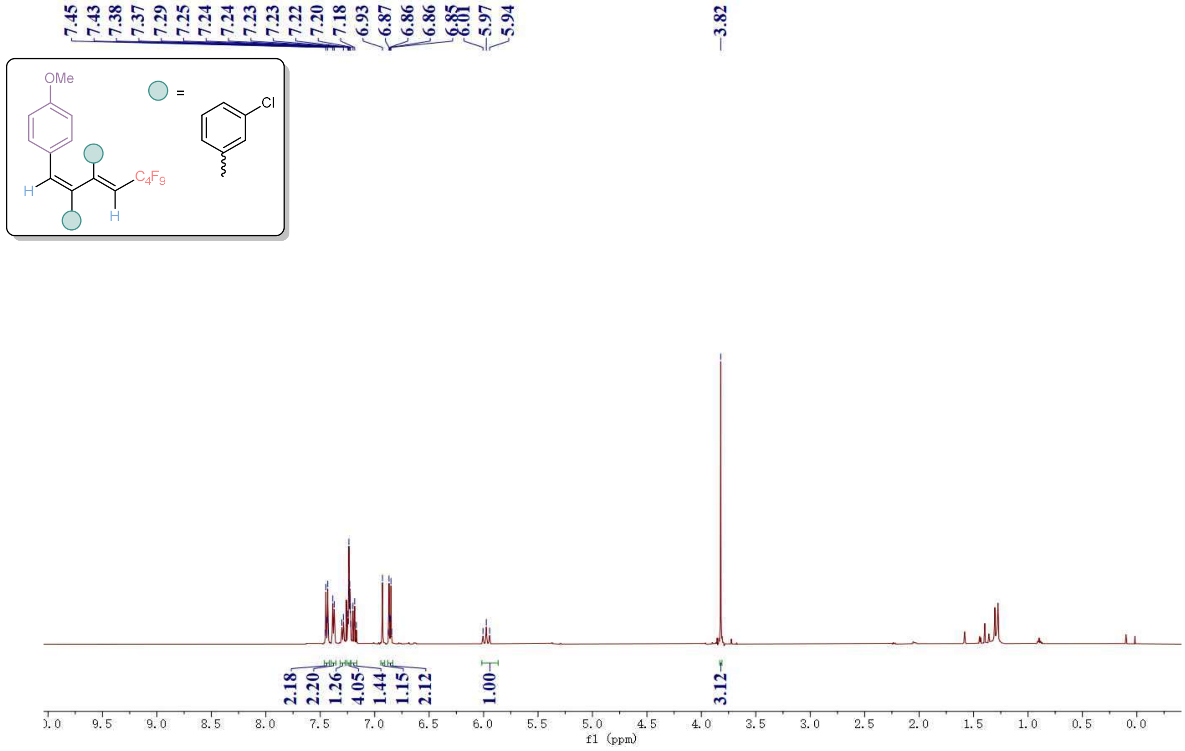

^{19}**F NMR** Spectrum of **16** (471 MHz, Chloroform-*d*)

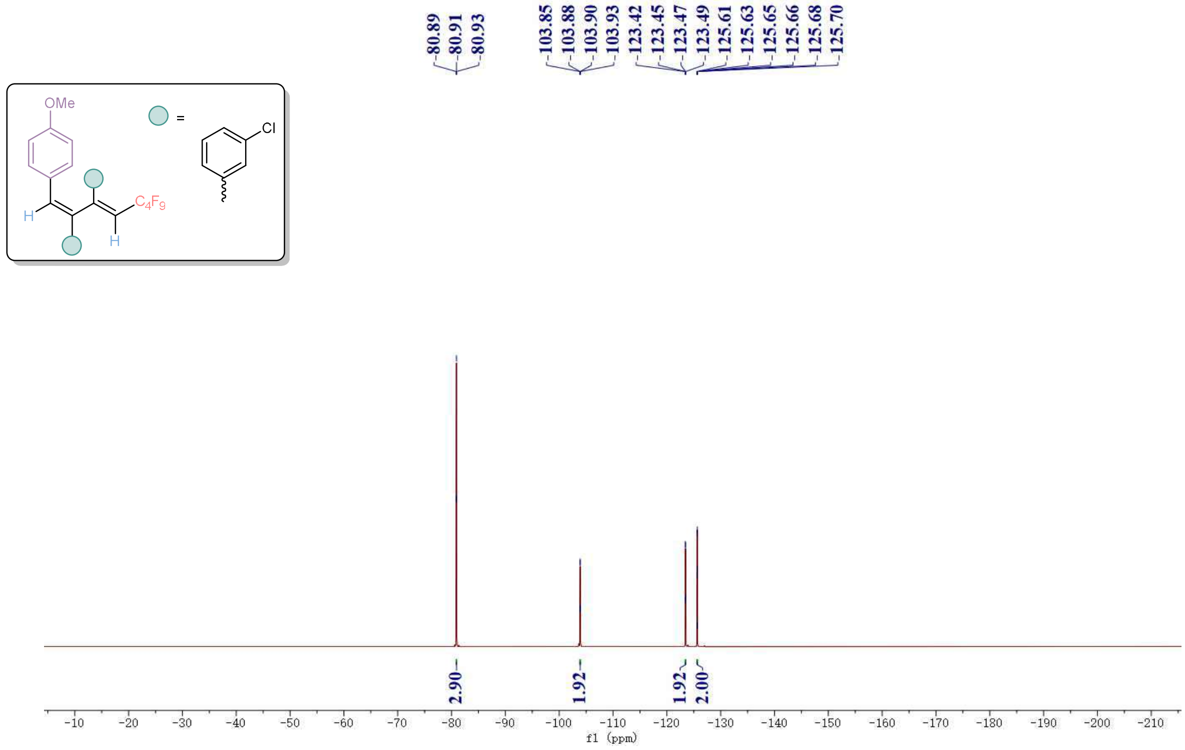

^{13}C NMR spectrum of **16** (126 MHz, Chloroform-*d*)

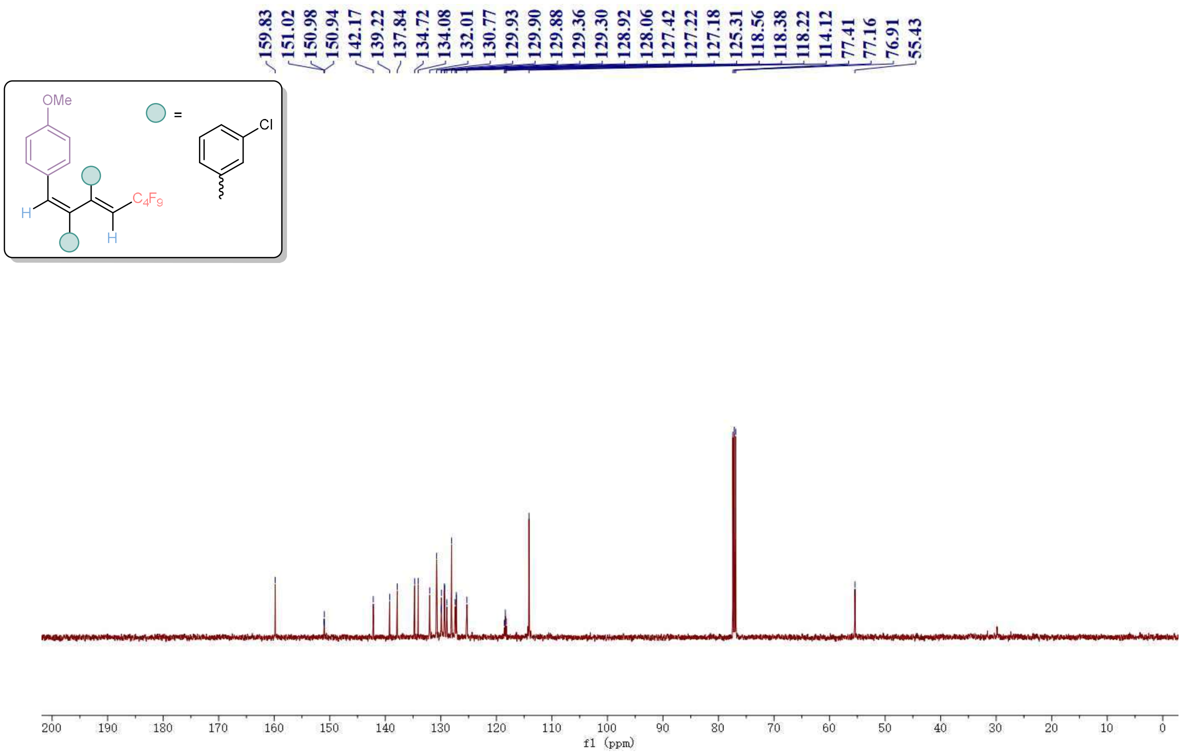

^{1}H NMR spectrum of **17** (500 MHz, Chloroform-*d*)

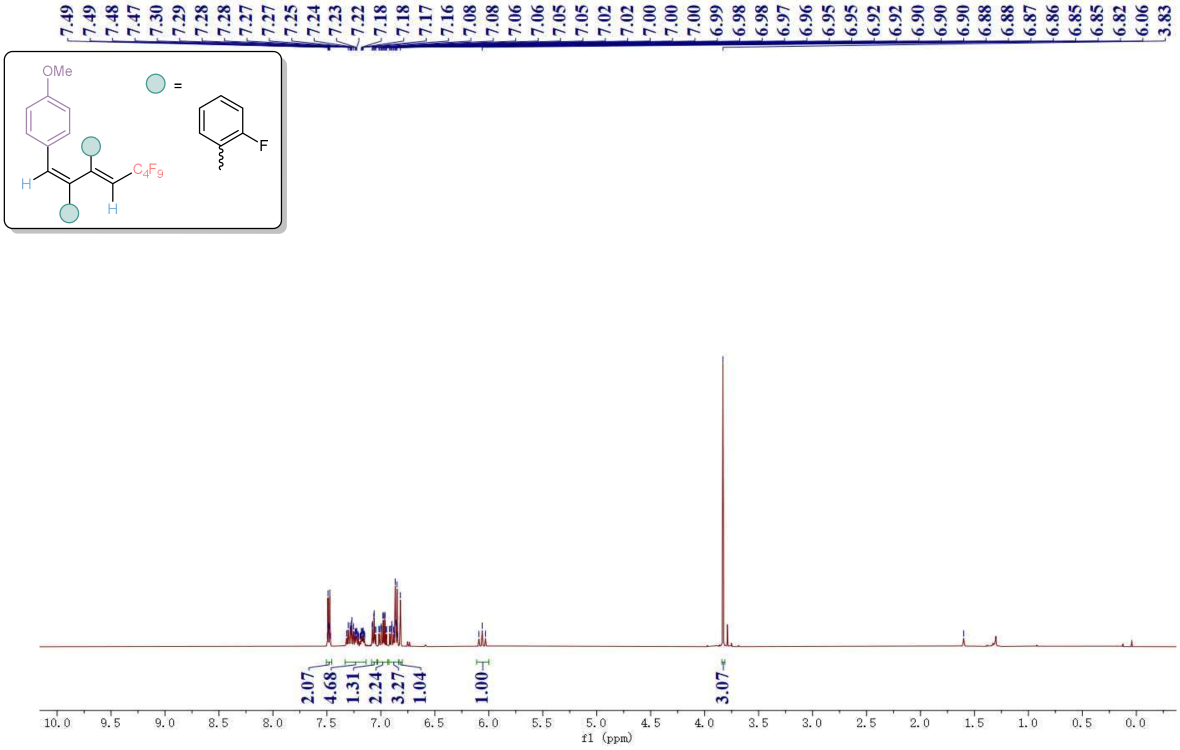

[19]**F NMR** Spectrum of **17** (471 MHz, Chloroform-*d*)

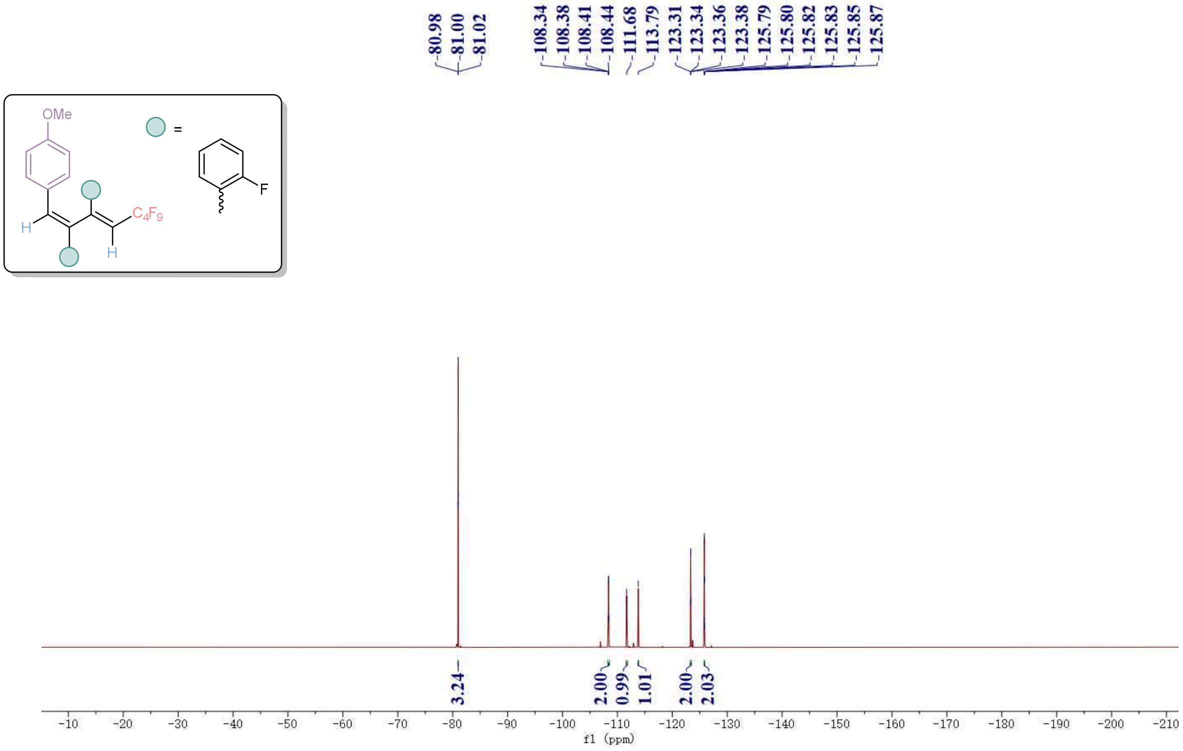

[13]**C NMR** spectrum of **17** (126 MHz, Chloroform-*d*)

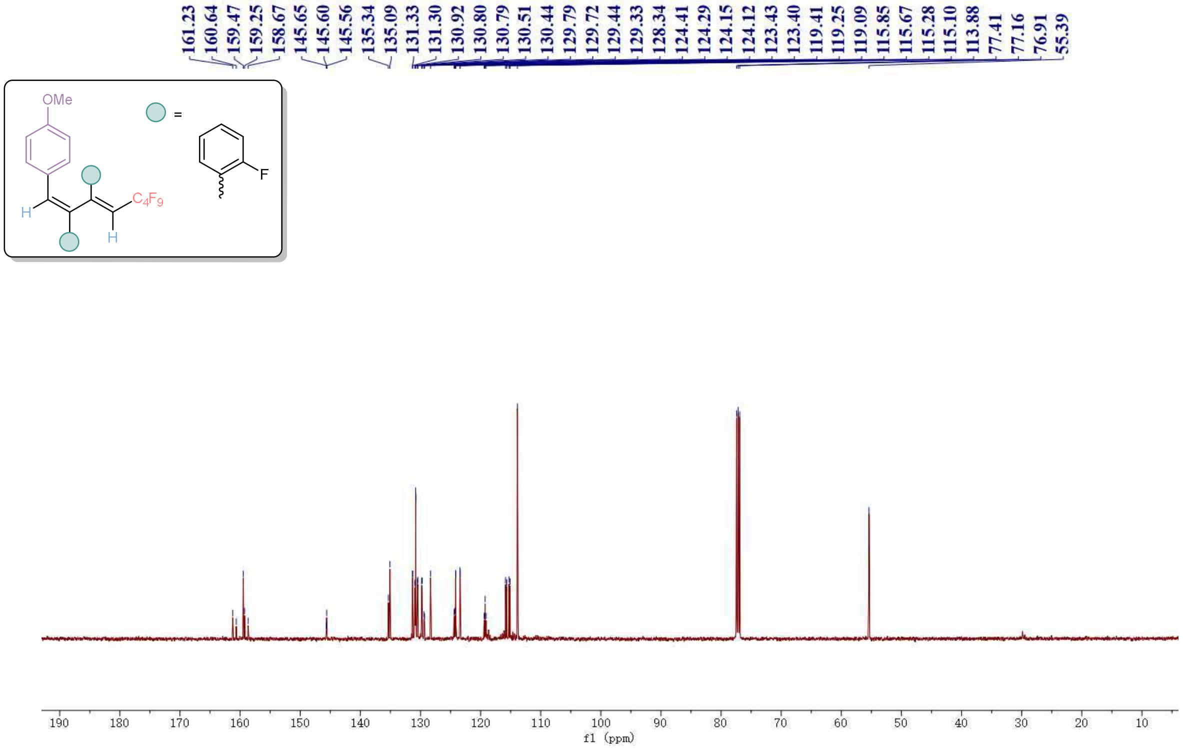

^{1}H NMR spectrum of **18** (500 MHz, Chloroform-*d*)

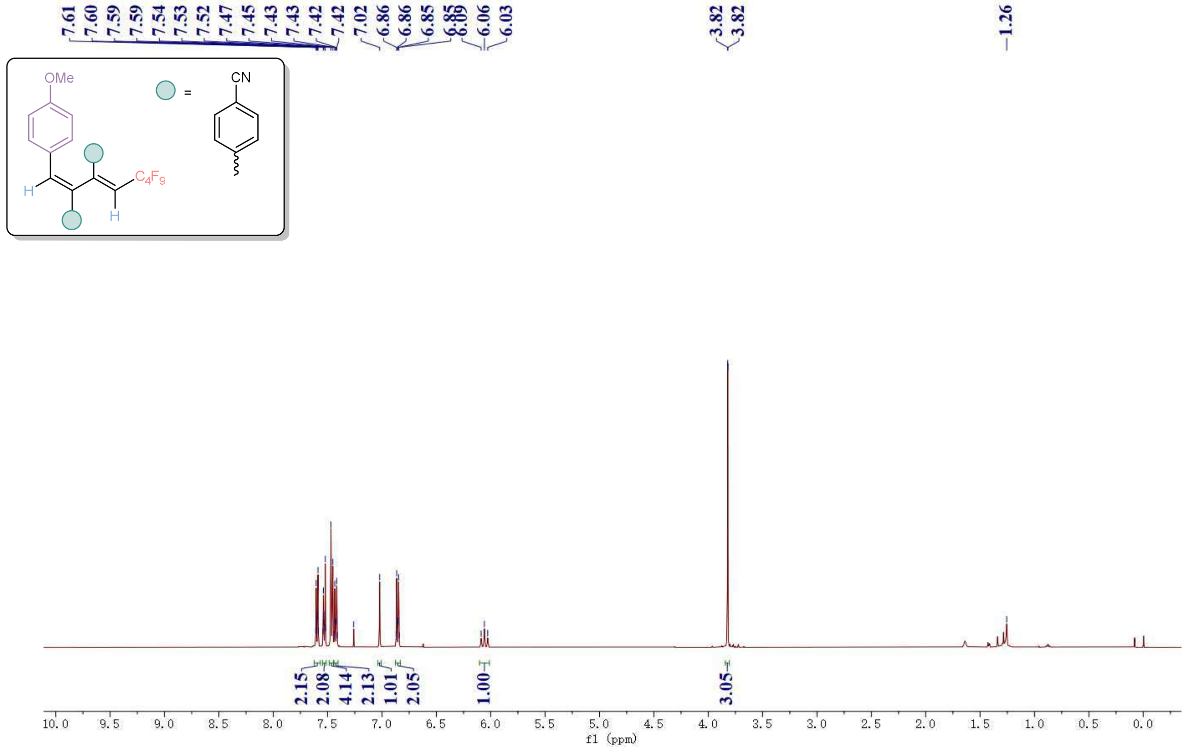

^{19}F NMR Spectrum of **18** (471 MHz, Chloroform-*d*)

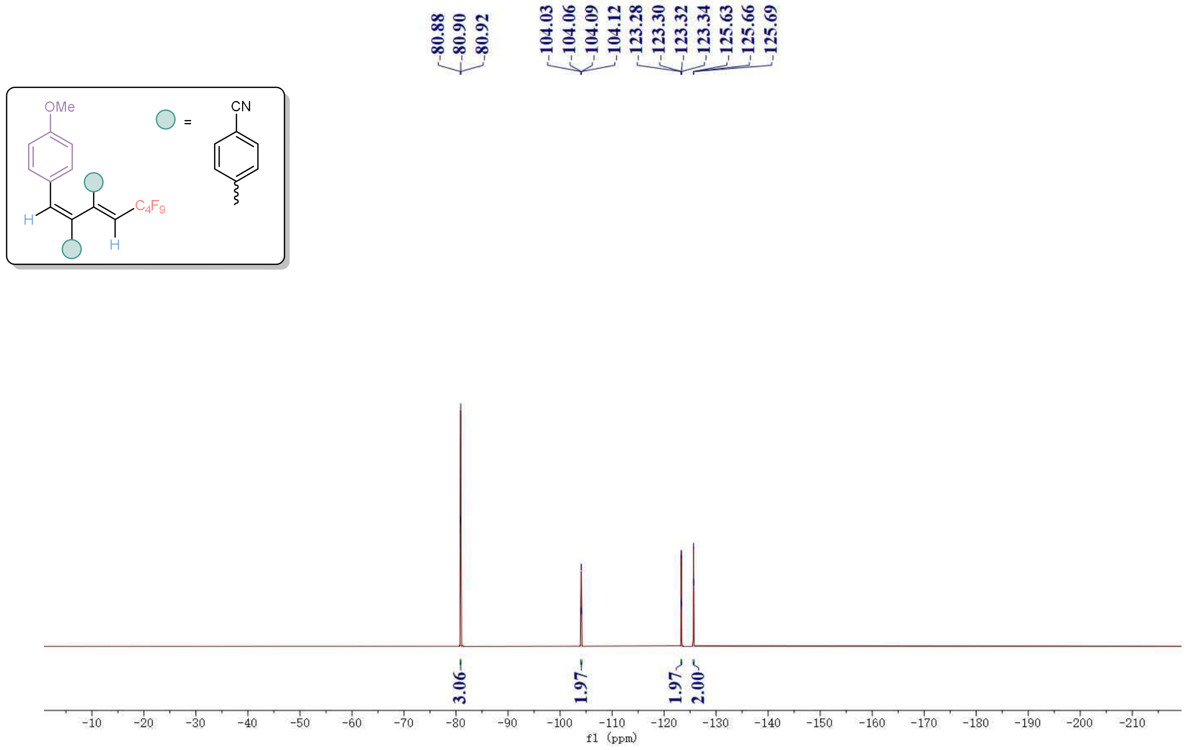

^{13}C NMR spectrum of **18** (126 MHz, Chloroform-*d*)

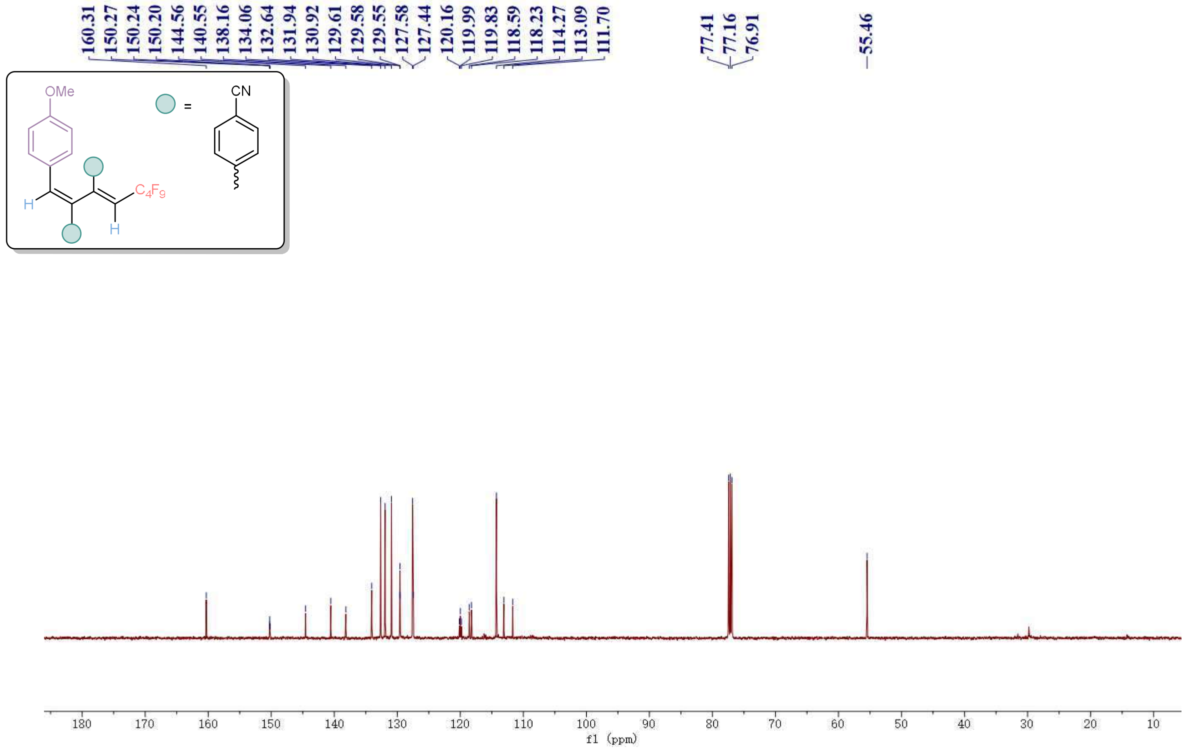

^{1}H NMR spectrum of **19** (500 MHz, Chloroform-*d*)

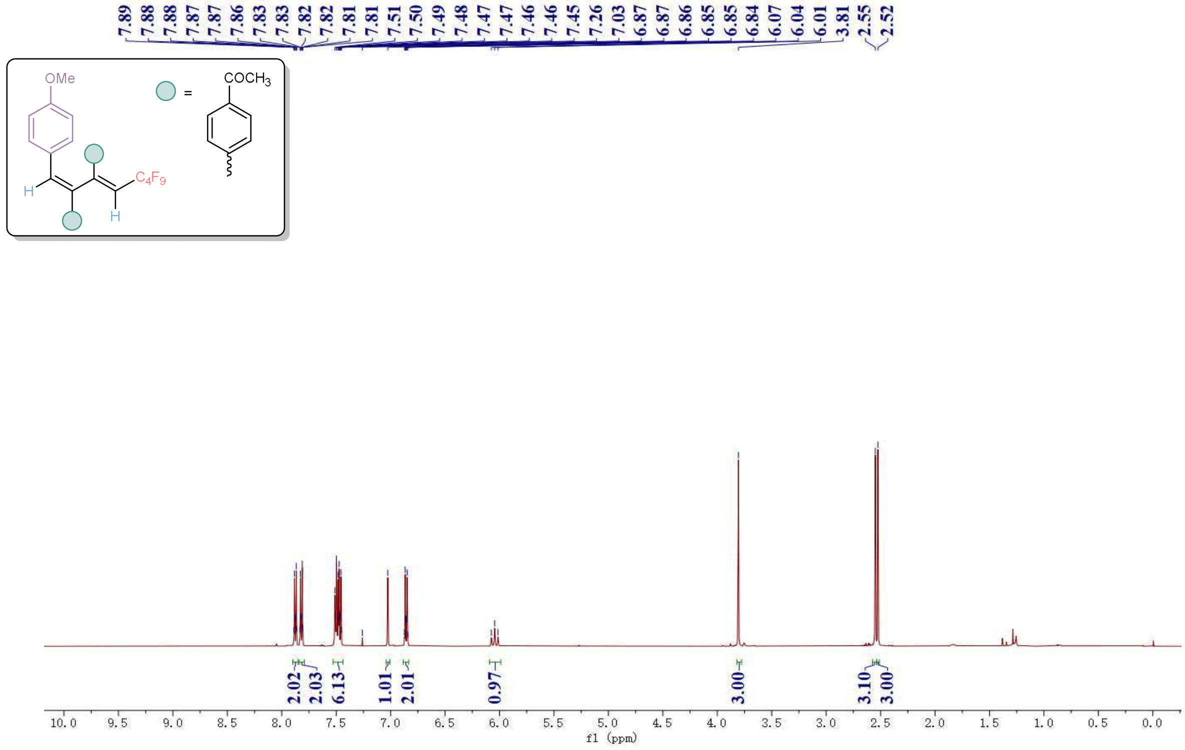

^{19}F NMR Spectrum of **19** (471 MHz, Chloroform-*d*)

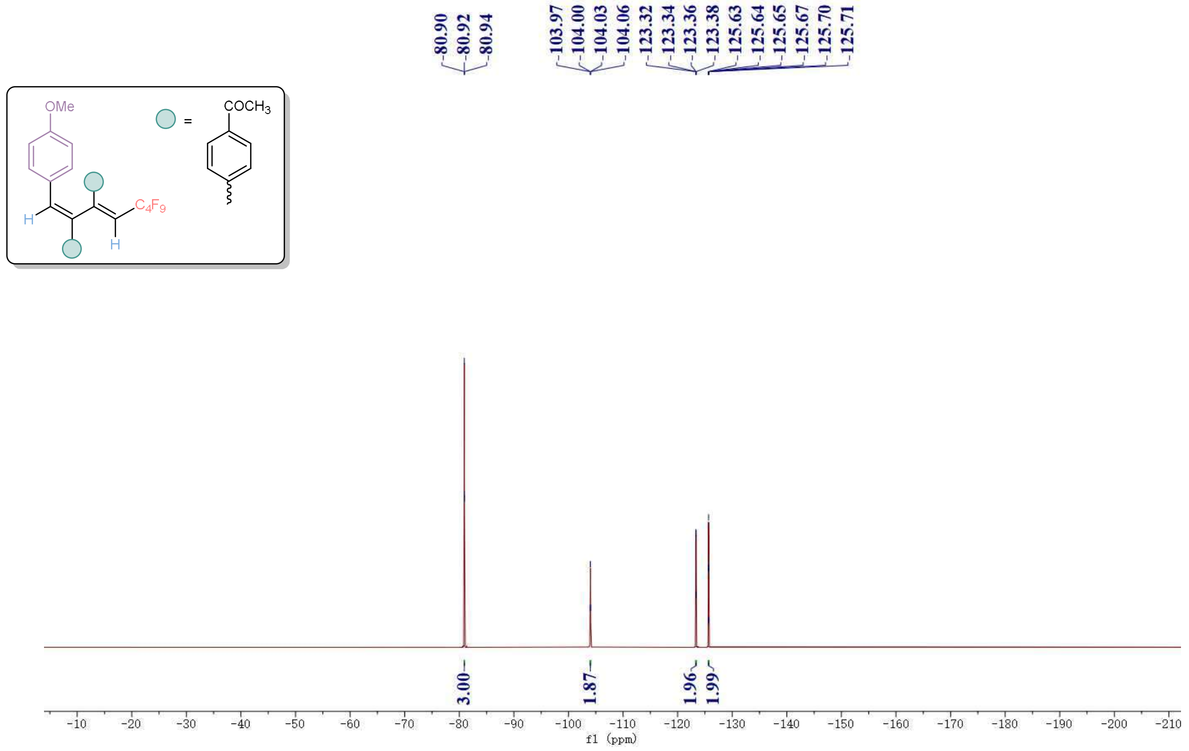

^{13}C NMR spectrum of **19** (126 MHz, Chloroform-*d*)

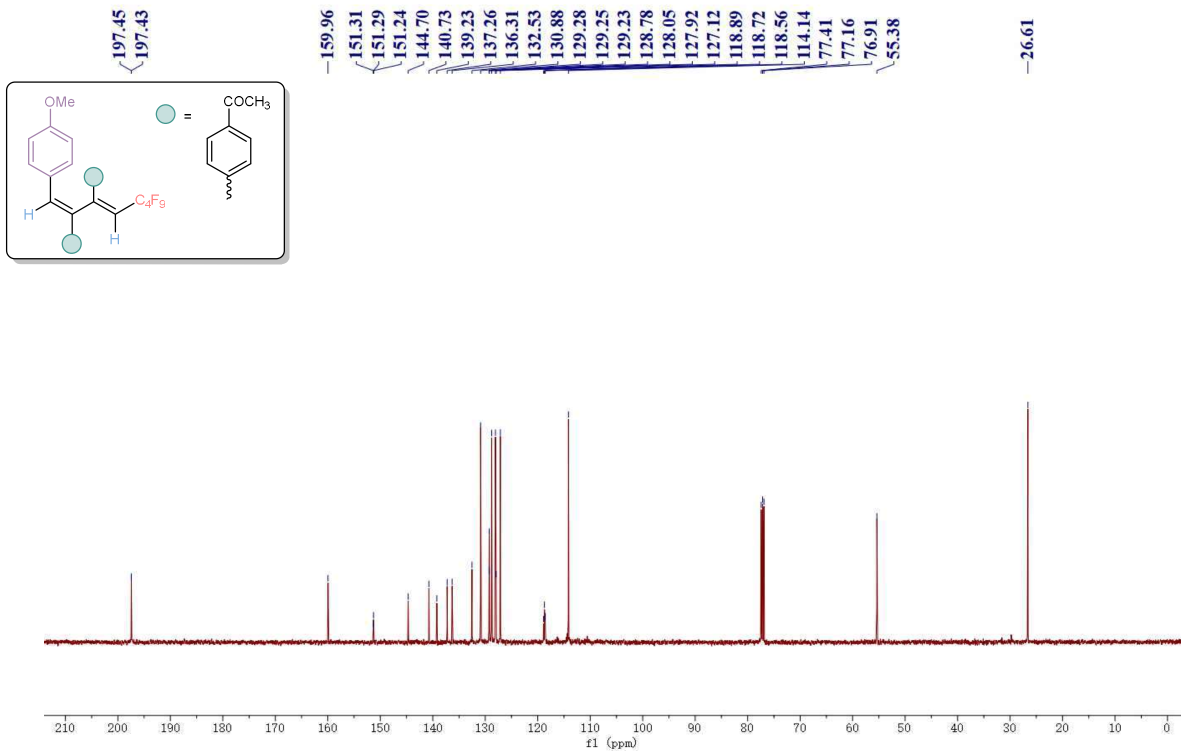

^{1}H NMR spectrum of **20** (500 MHz, Chloroform-*d*)

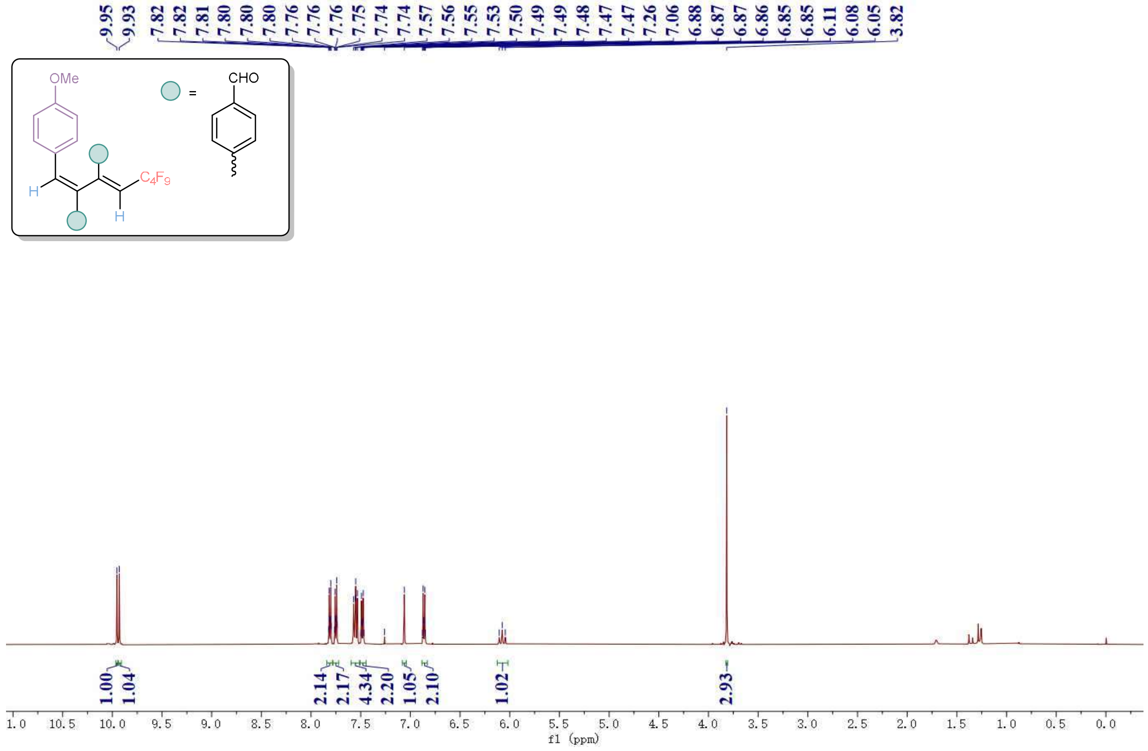

^{19}F NMR Spectrum of **20** (471 MHz, Chloroform-*d*)

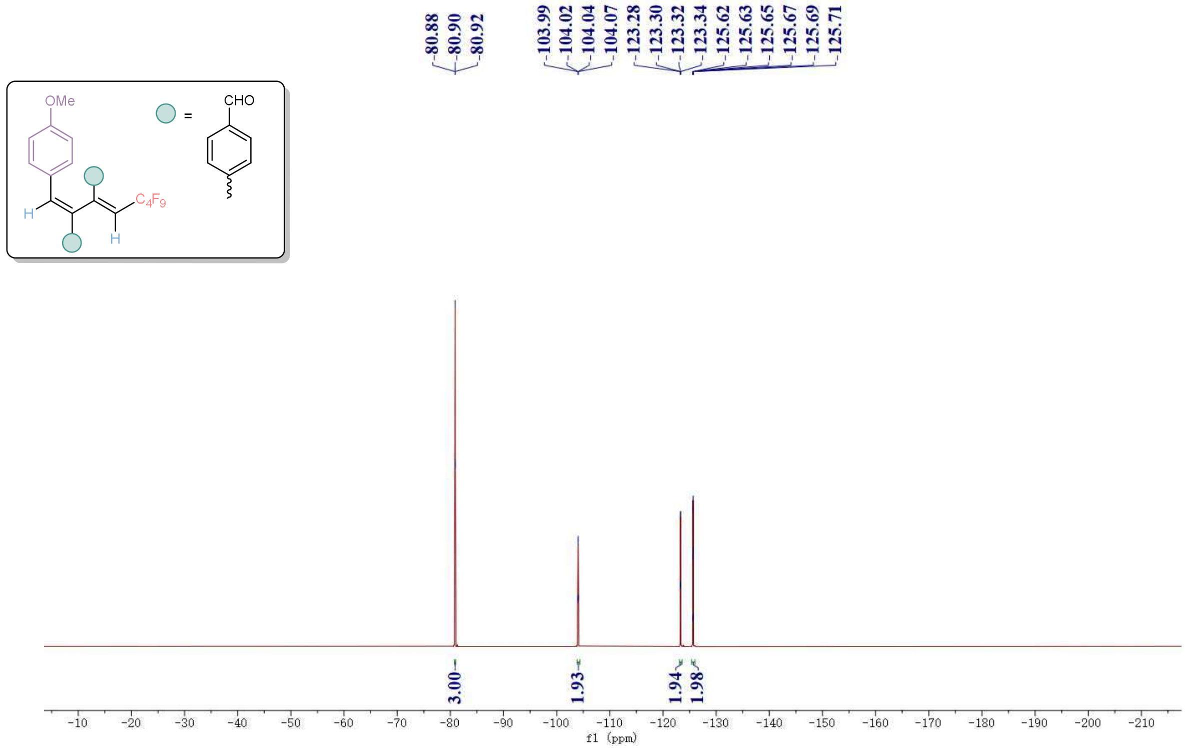

^{13}C NMR spectrum of 20 (126 MHz, Chloroform-d)

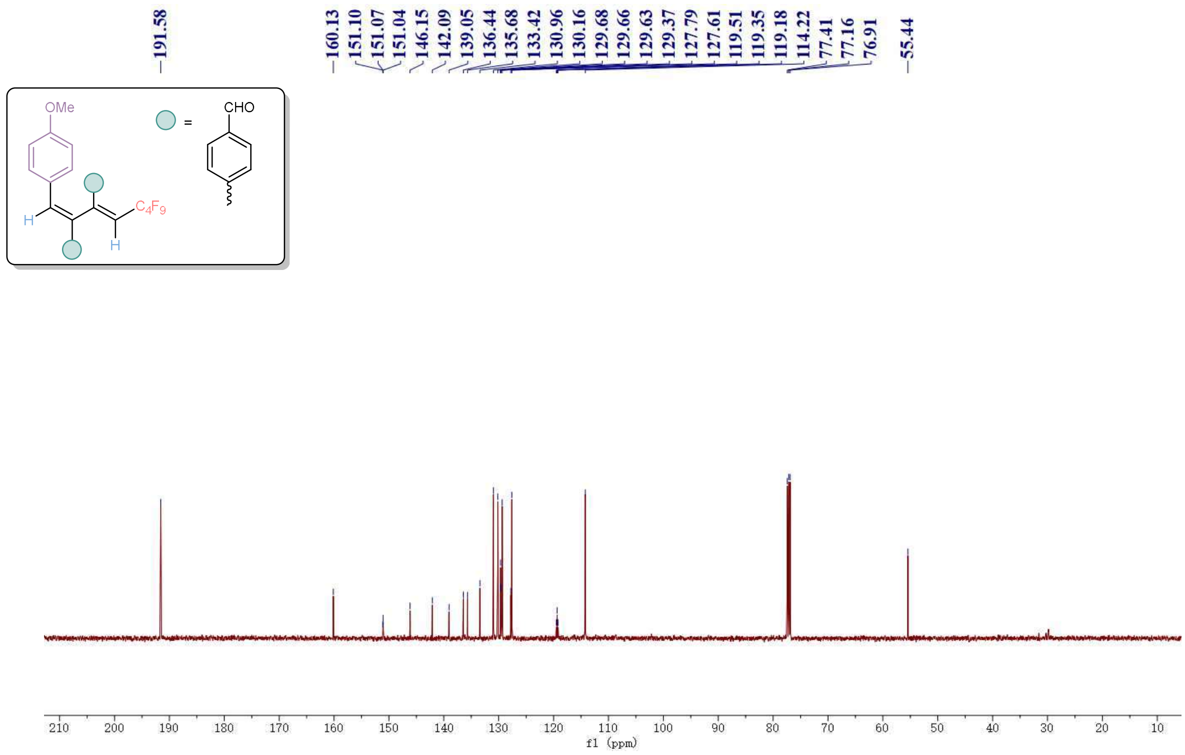

^{1}H NMR spectrum of 21 (500 MHz, Chloroform-d)

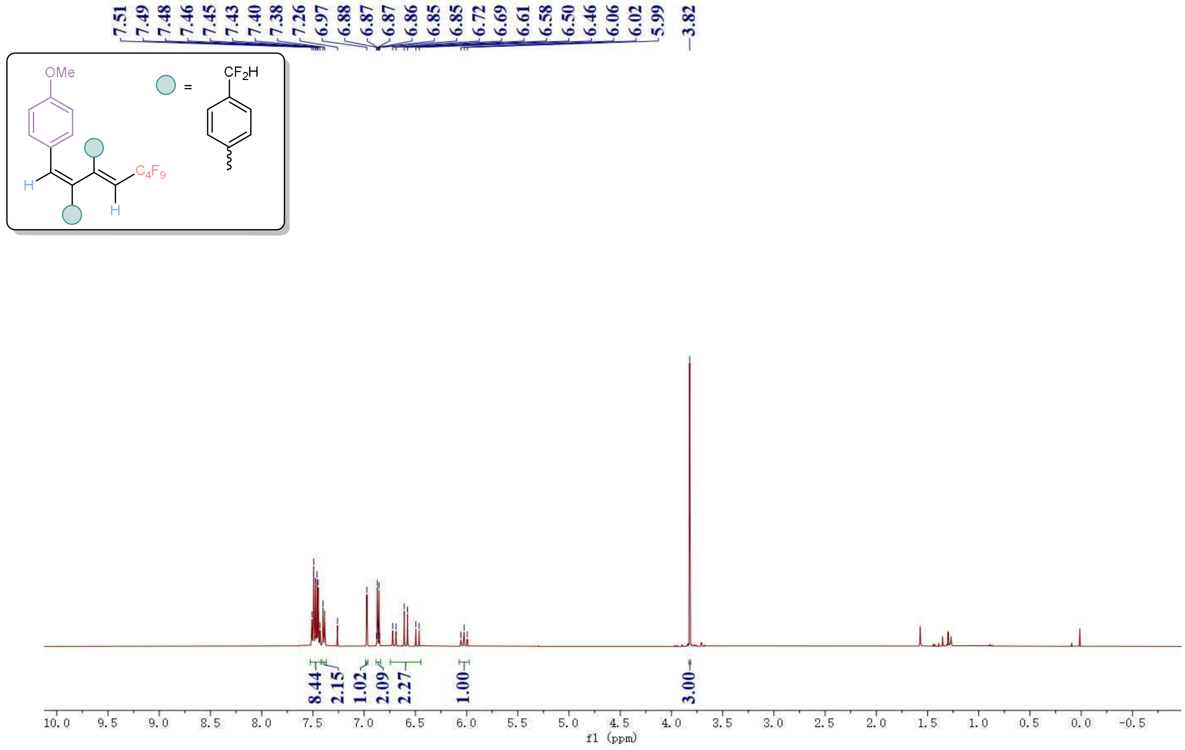

^{19}**F NMR** Spectrum of **21** (471 MHz, Chloroform-*d*)

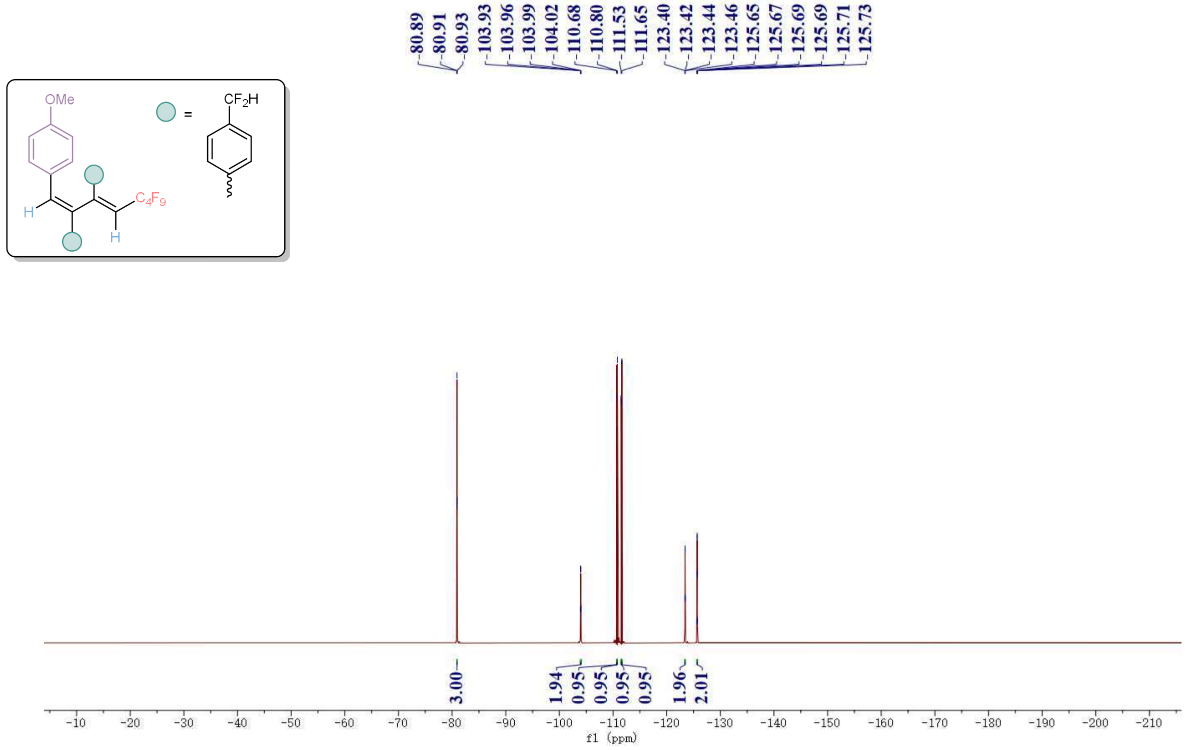

^{13}**C NMR** spectrum of **21** (126 MHz, Chloroform-*d*)

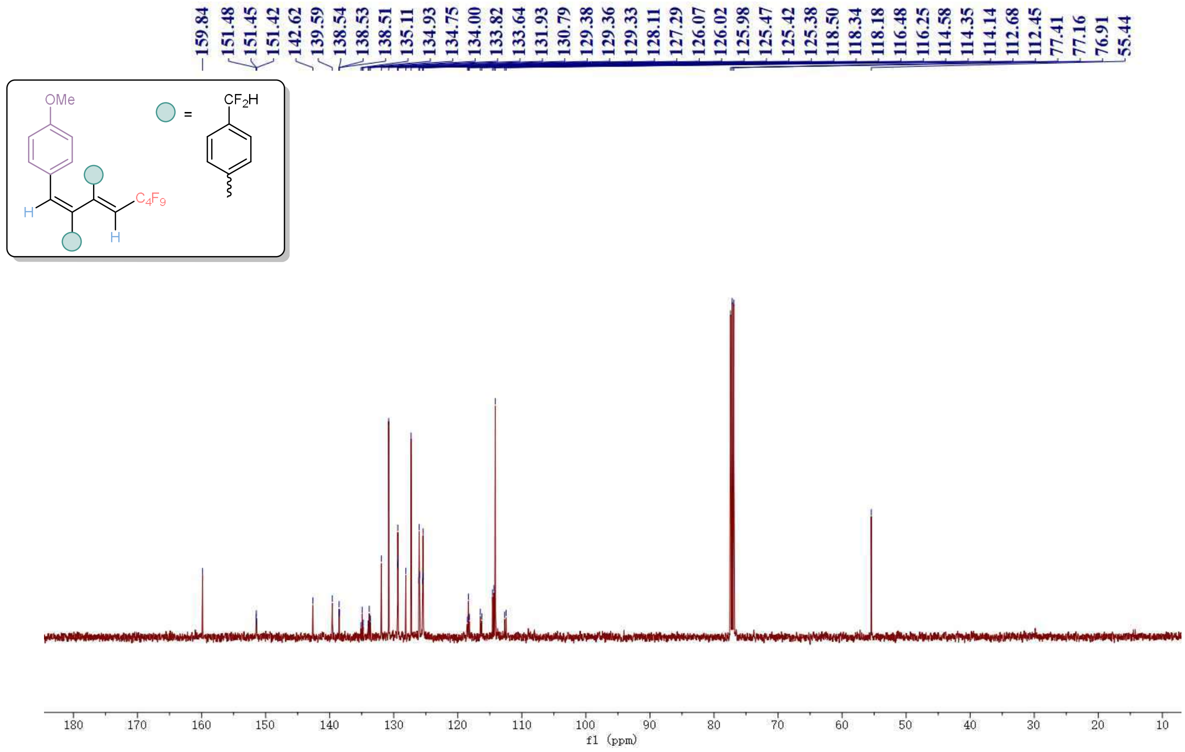

^{1}H NMR spectrum of **22** (500 MHz, Chloroform-*d*)

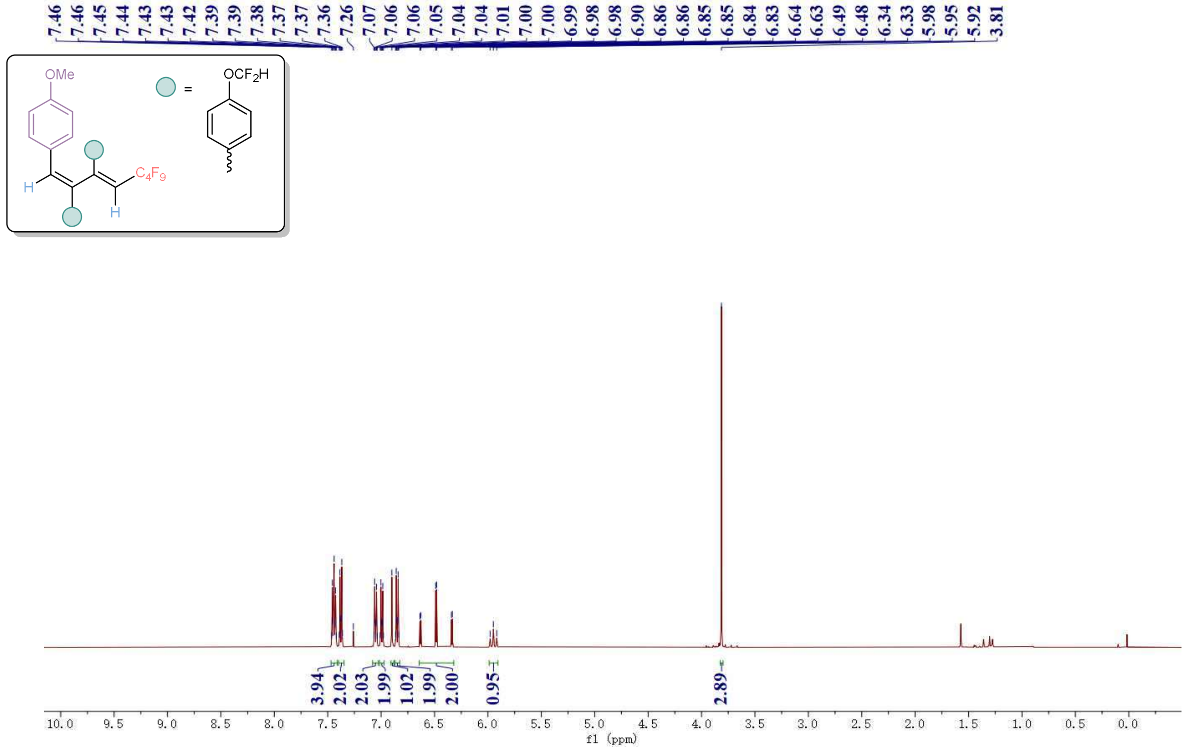

^{19}F NMR Spectrum of **22** (471 MHz, Chloroform-*d*)

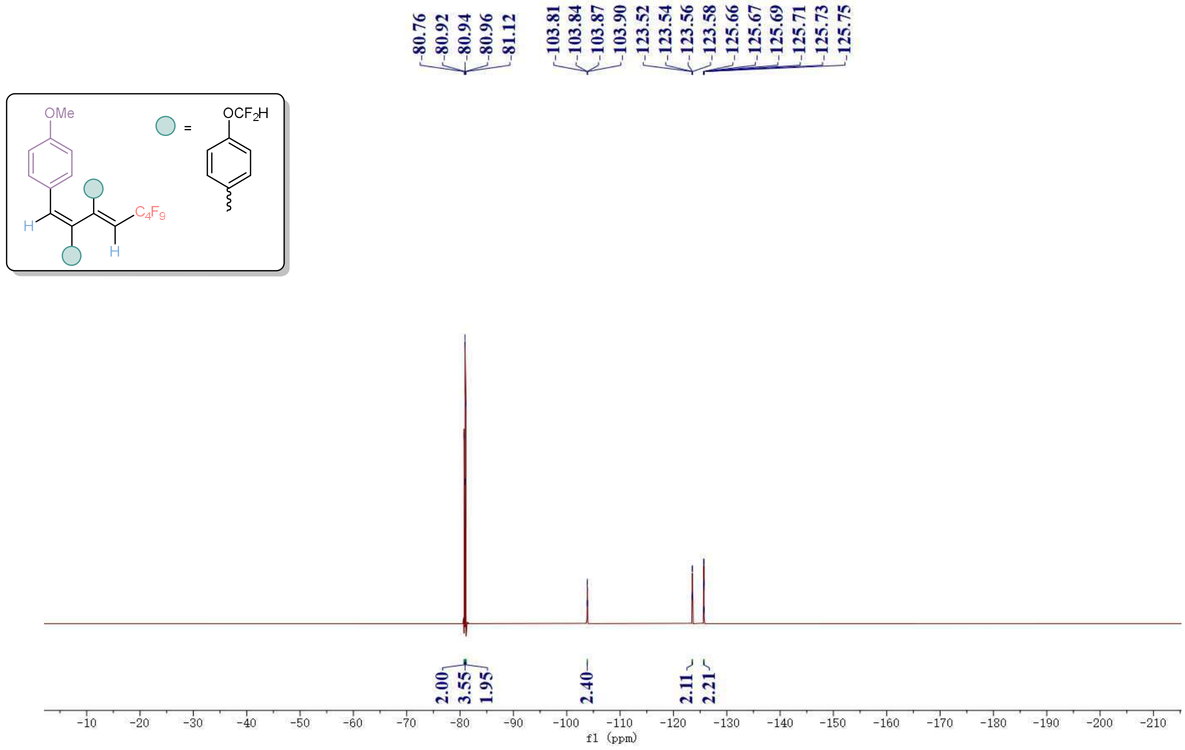

^{13}C NMR spectrum of **22** (126 MHz, Chloroform-d)

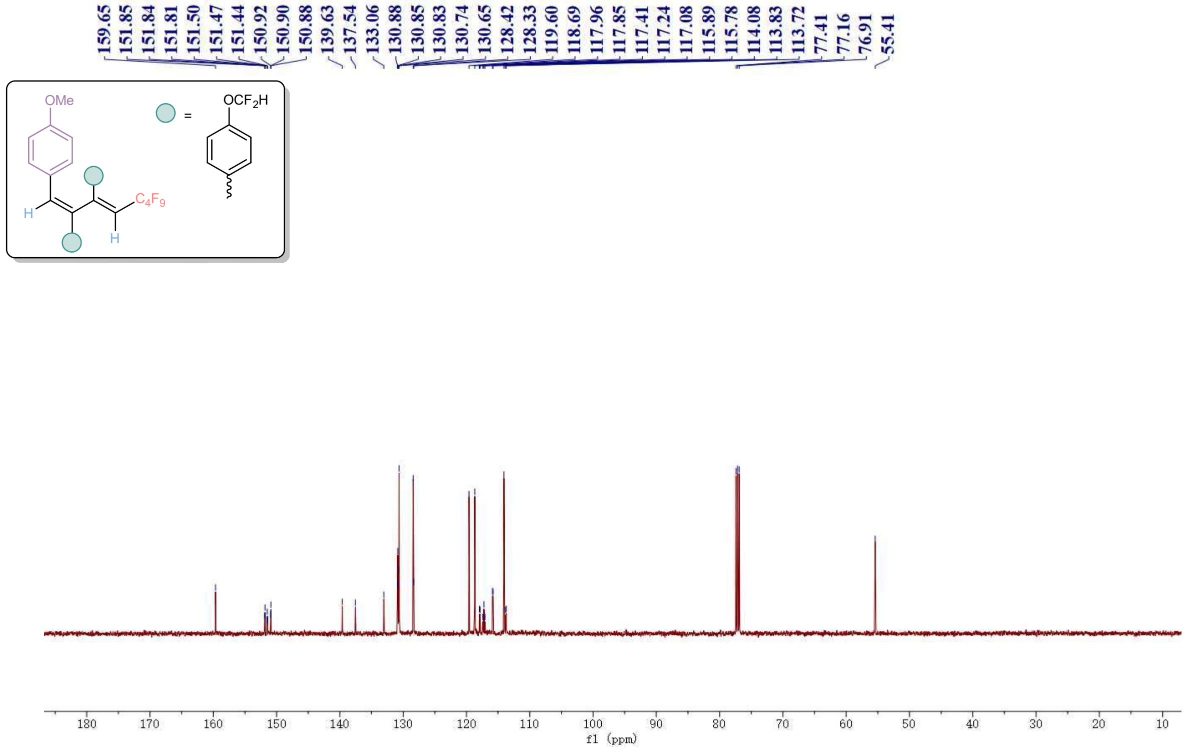

^{1}H NMR spectrum of **23** (500 MHz, Chloroform-*d*)

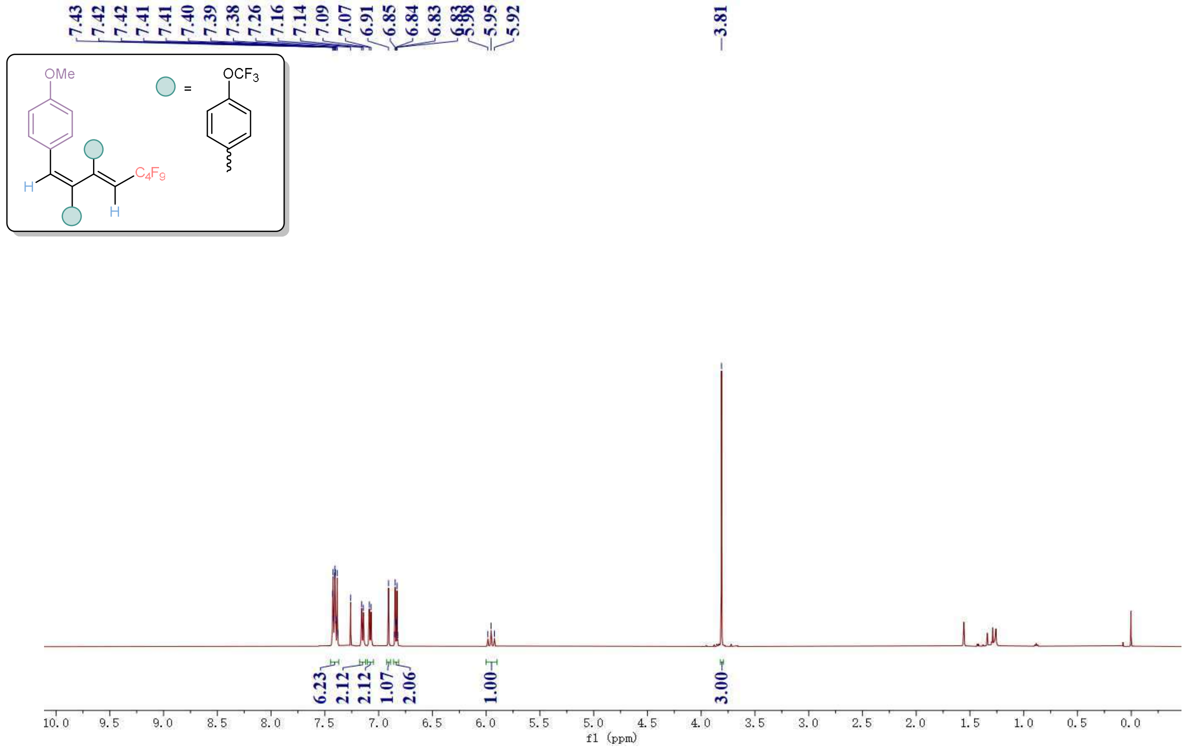

^{19}F NMR Spectrum of **23** (471 MHz, Chloroform-*d*)

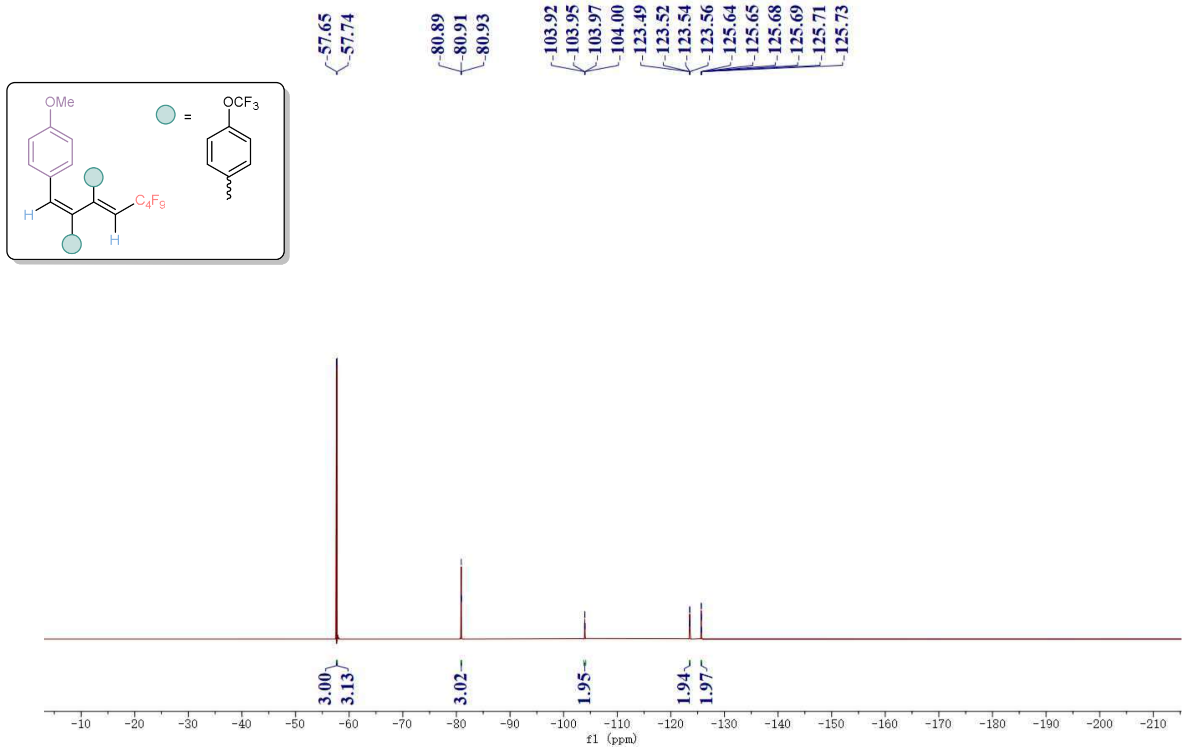

^{13}C NMR spectrum of **23** (126 MHz, Chloroform-*d*)

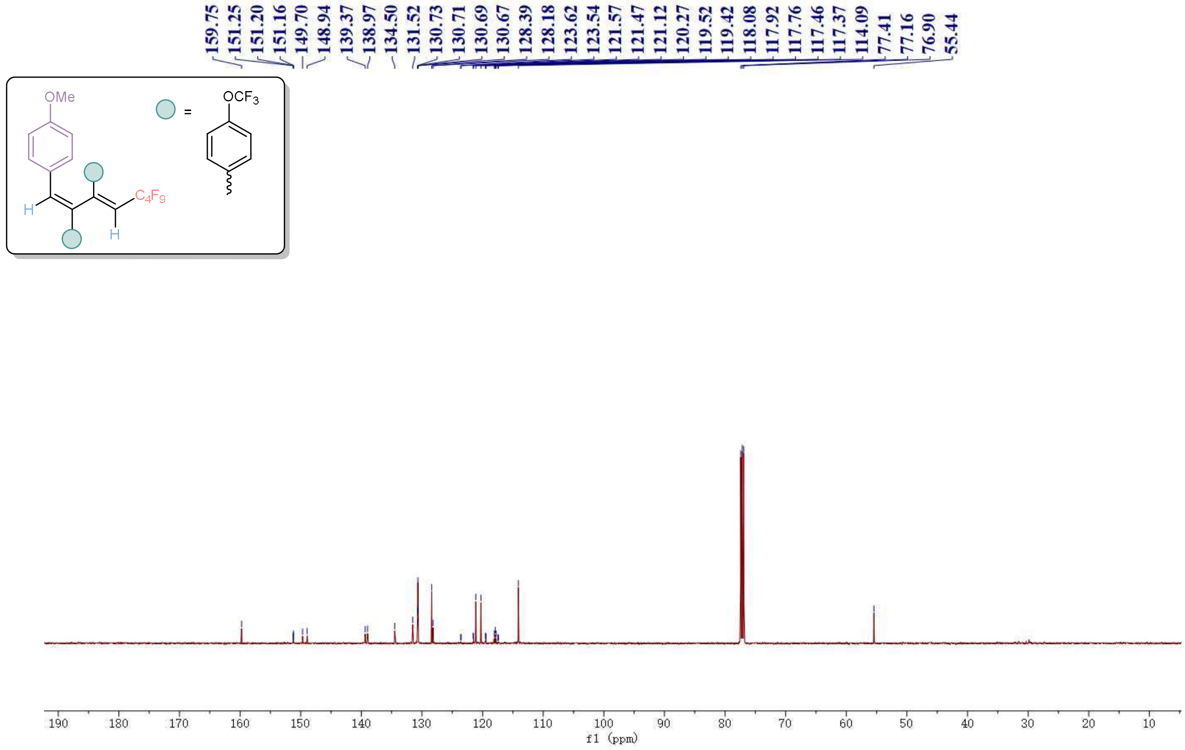

^{1}H NMR spectrum of **24** (500 MHz, Chloroform-*d*)

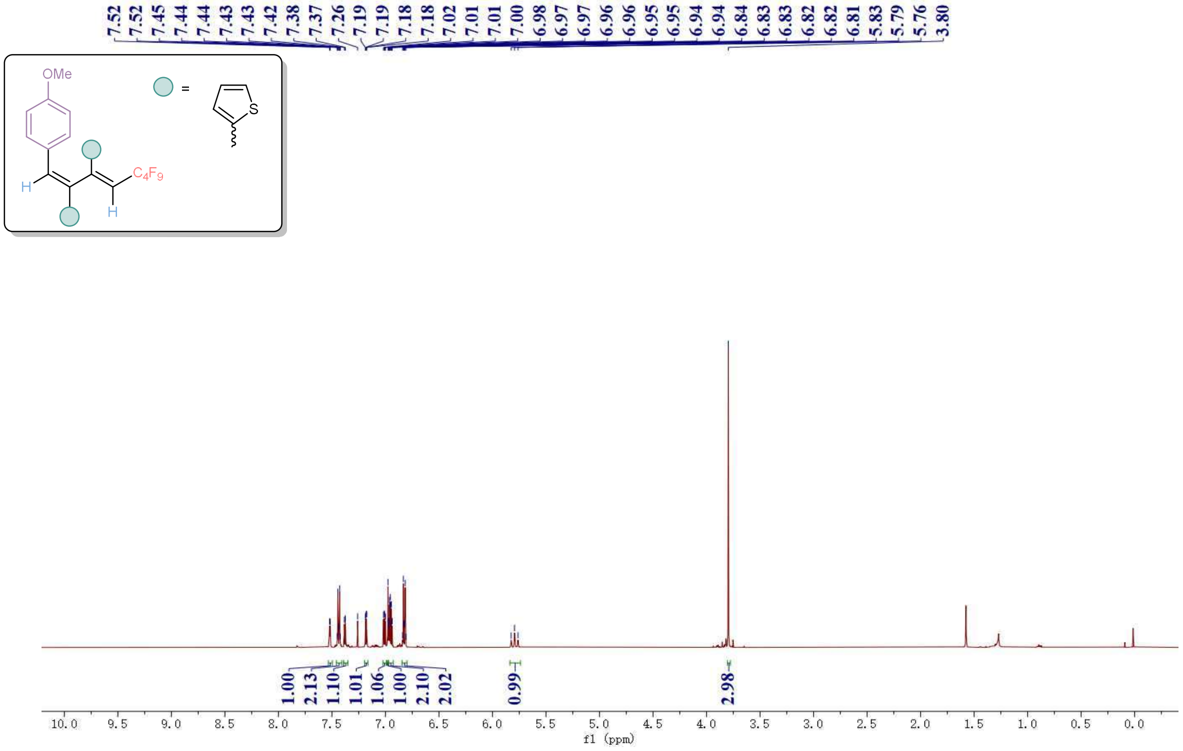

^{19}F NMR Spectrum of **24** (471 MHz, Chloroform-*d*)

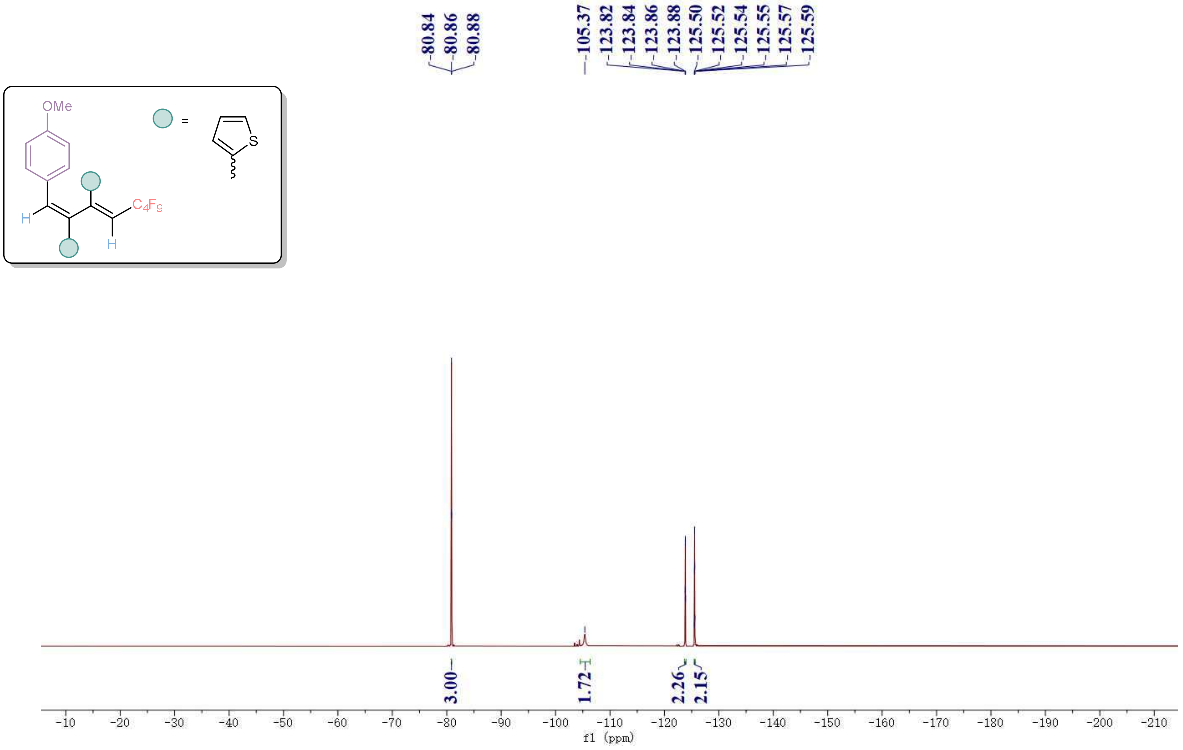

^{13}C **NMR** spectrum of **24** (126 MHz, Chloroform-*d*)

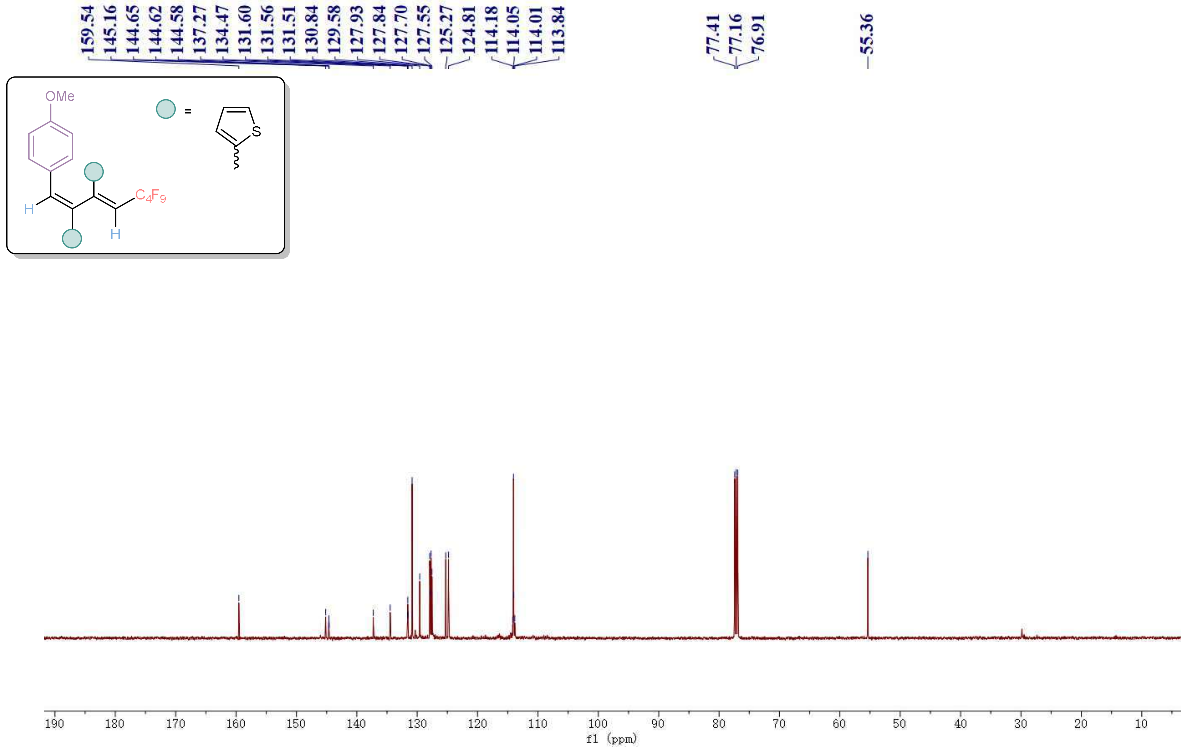

^{1}H **NMR** spectrum of **25** (500 MHz, Chloroform-*d*)

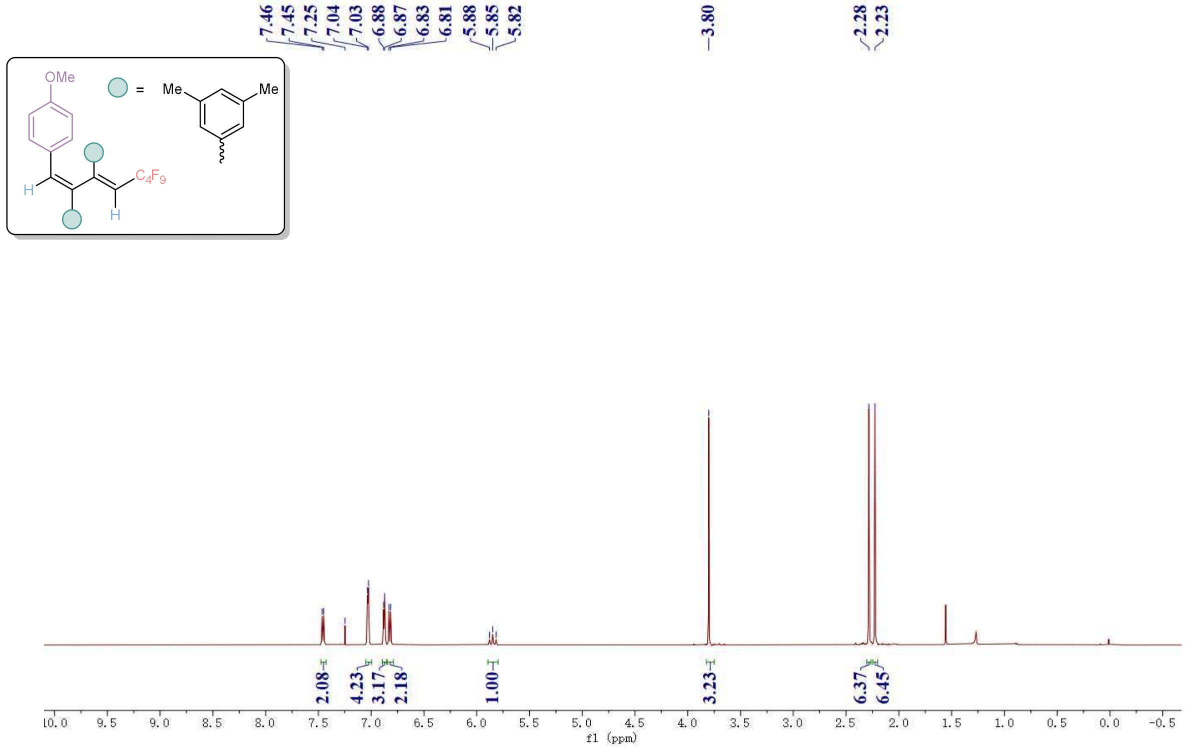

^{19}F NMR Spectrum of **25** (471 MHz, Chloroform-*d*)

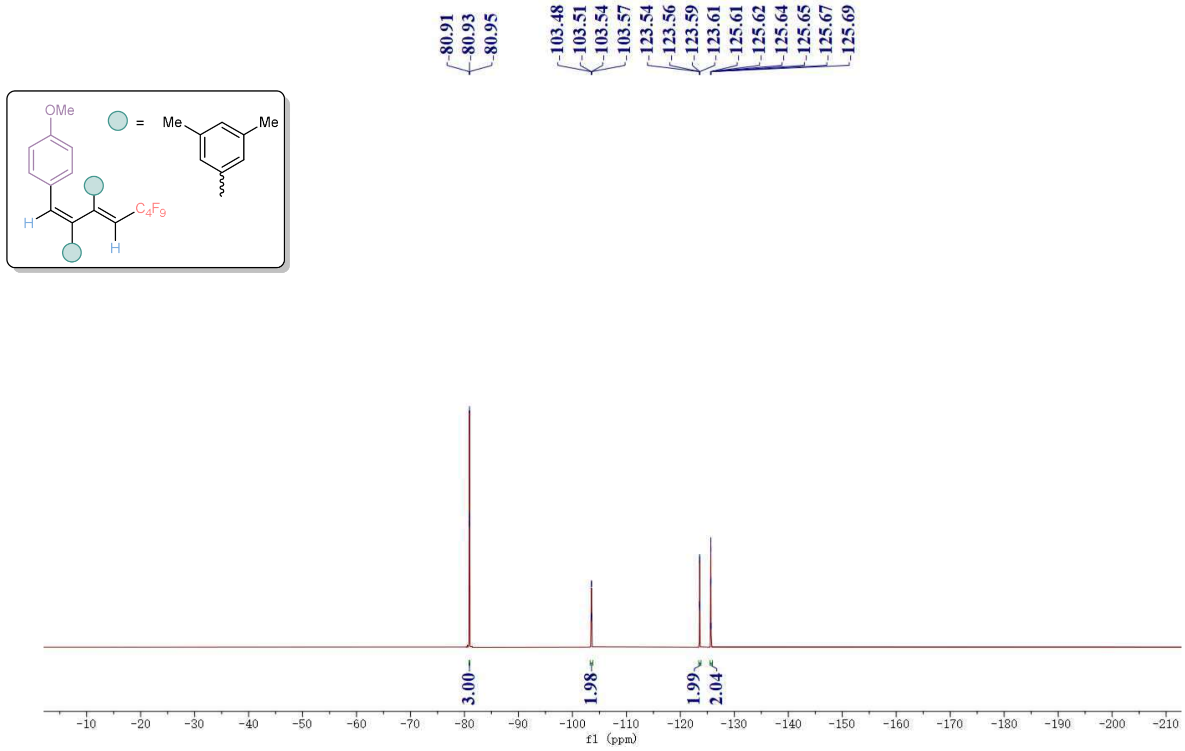

^{13}C NMR spectrum of **25** (126 MHz, Chloroform-*d*)

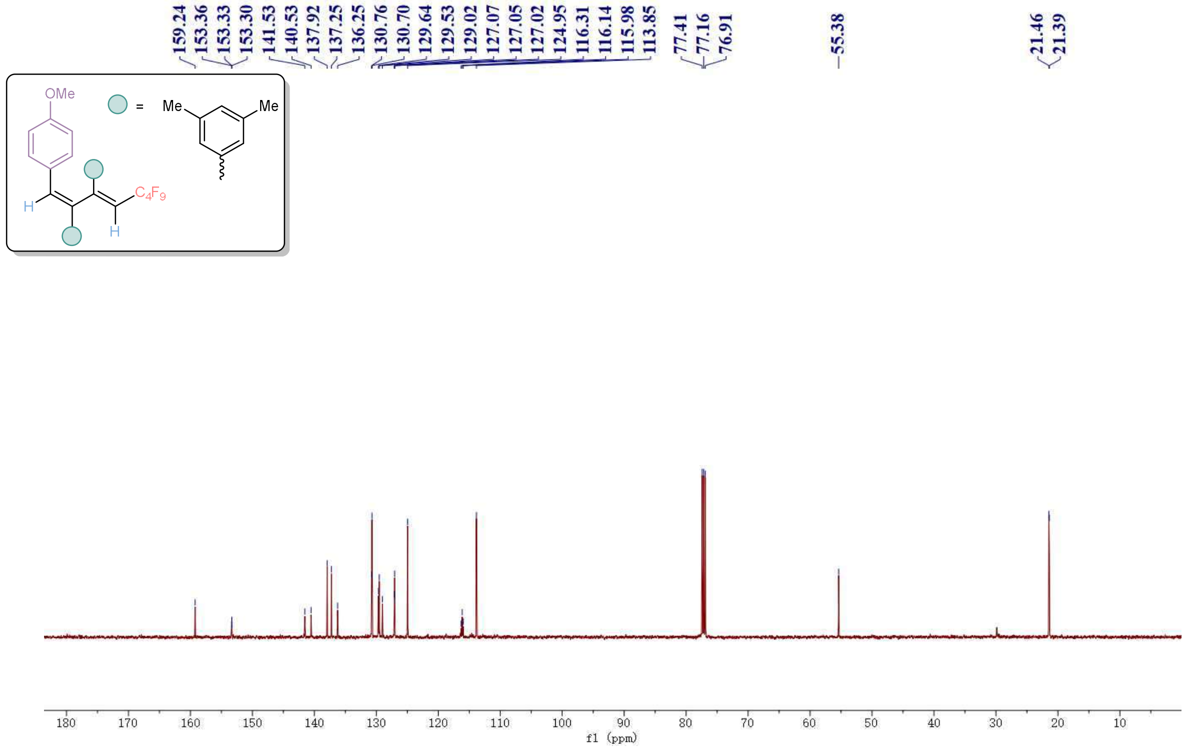

^{1}H NMR spectrum of **26** (500 MHz, Chloroform-*d*)

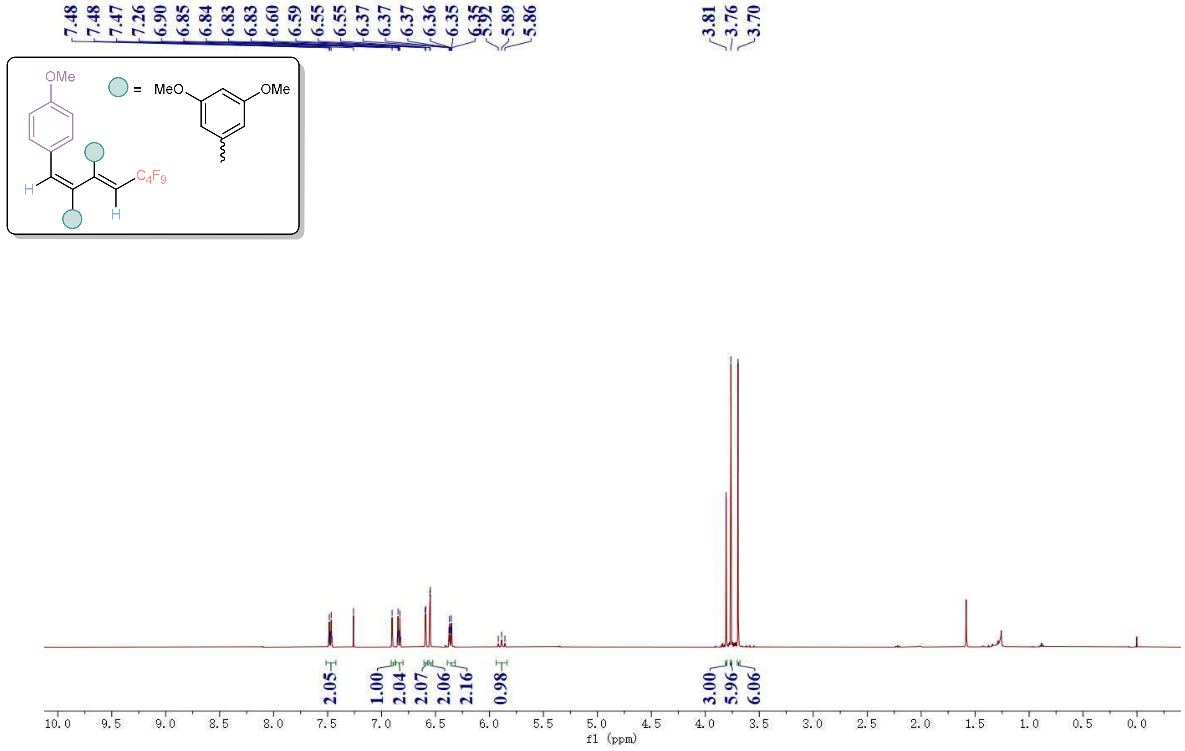

^{19}F NMR Spectrum of **26** (471 MHz, Chloroform-*d*)

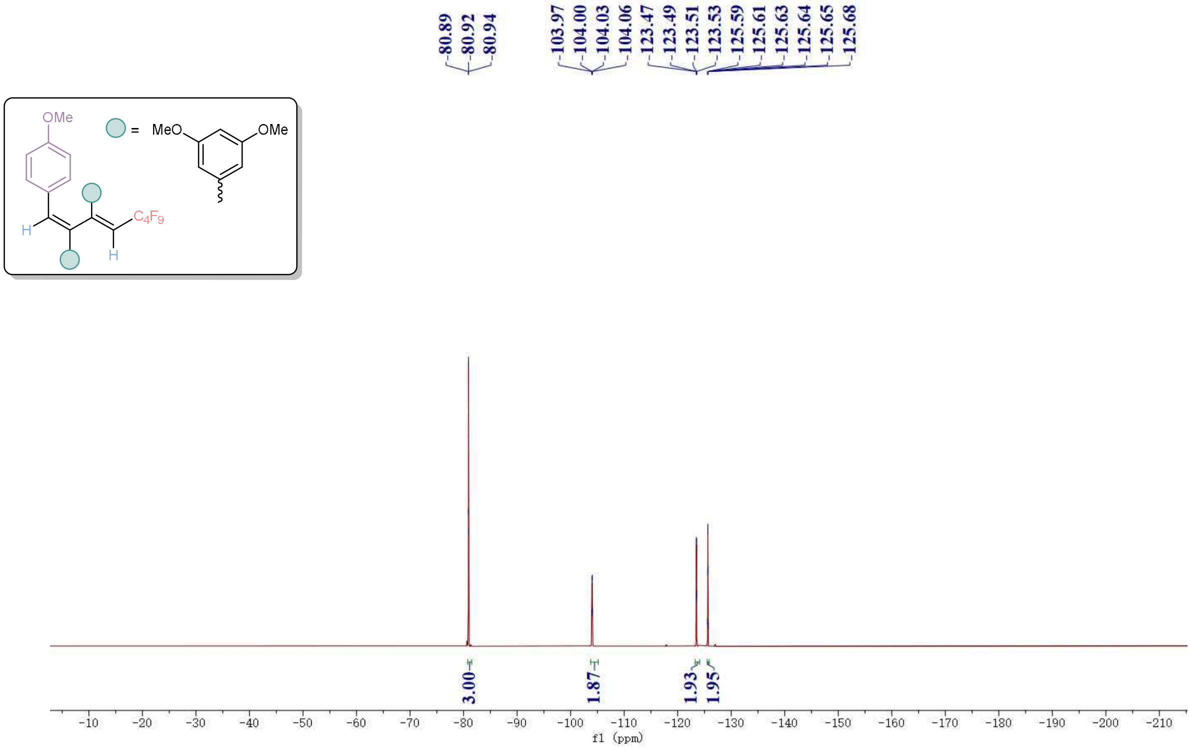

^{13}C NMR spectrum of **26** (126 MHz, Chloroform-*d*)

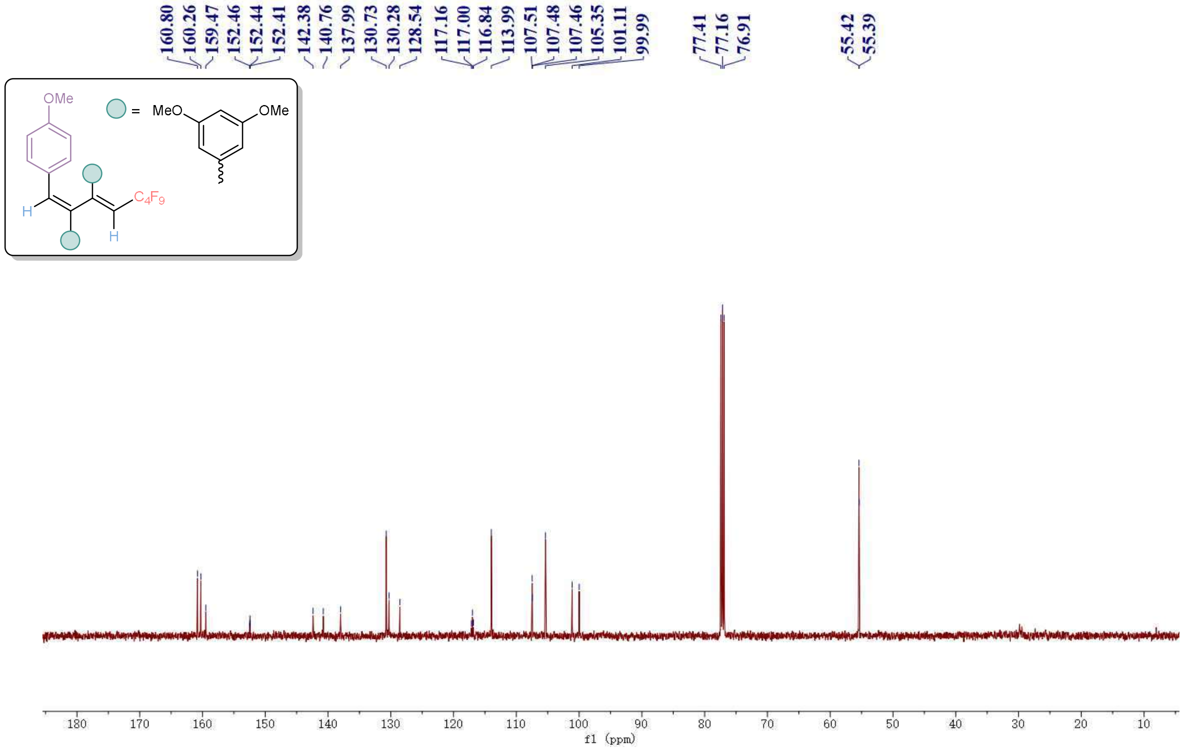

^{1}H NMR spectrum of **27** (500 MHz, Chloroform-*d*)

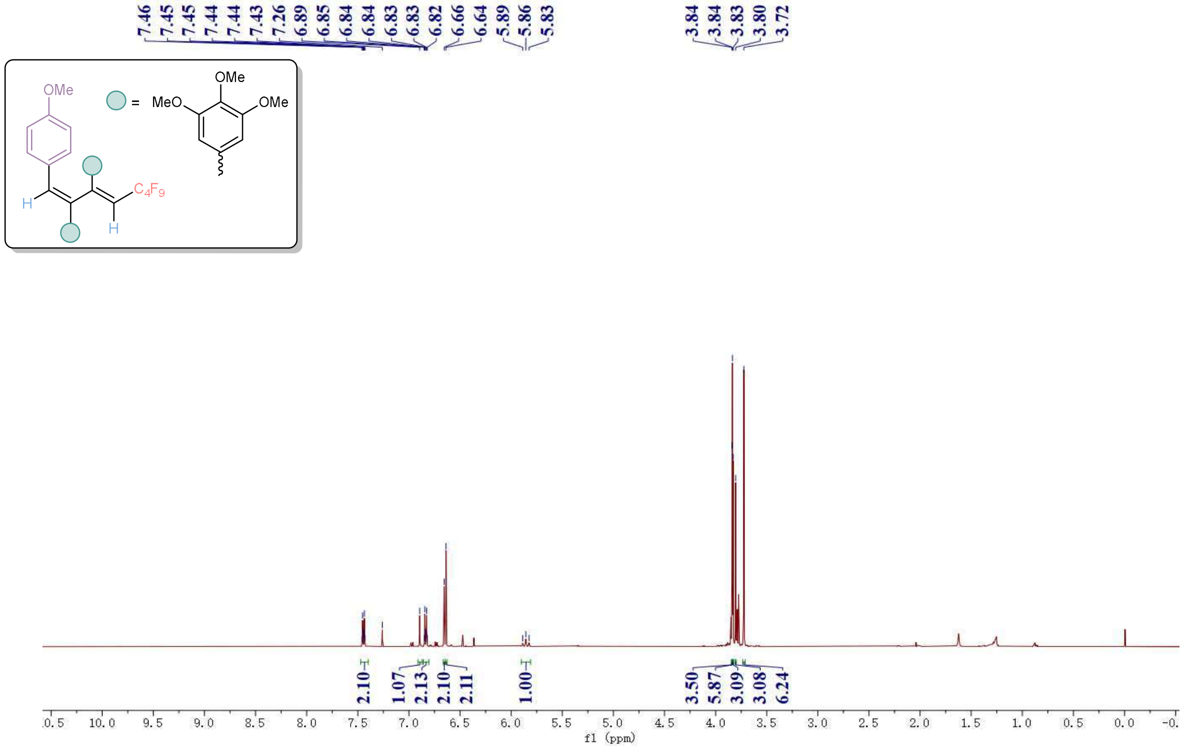

^{19}F NMR Spectrum of **27** (471 MHz, Chloroform-*d*)

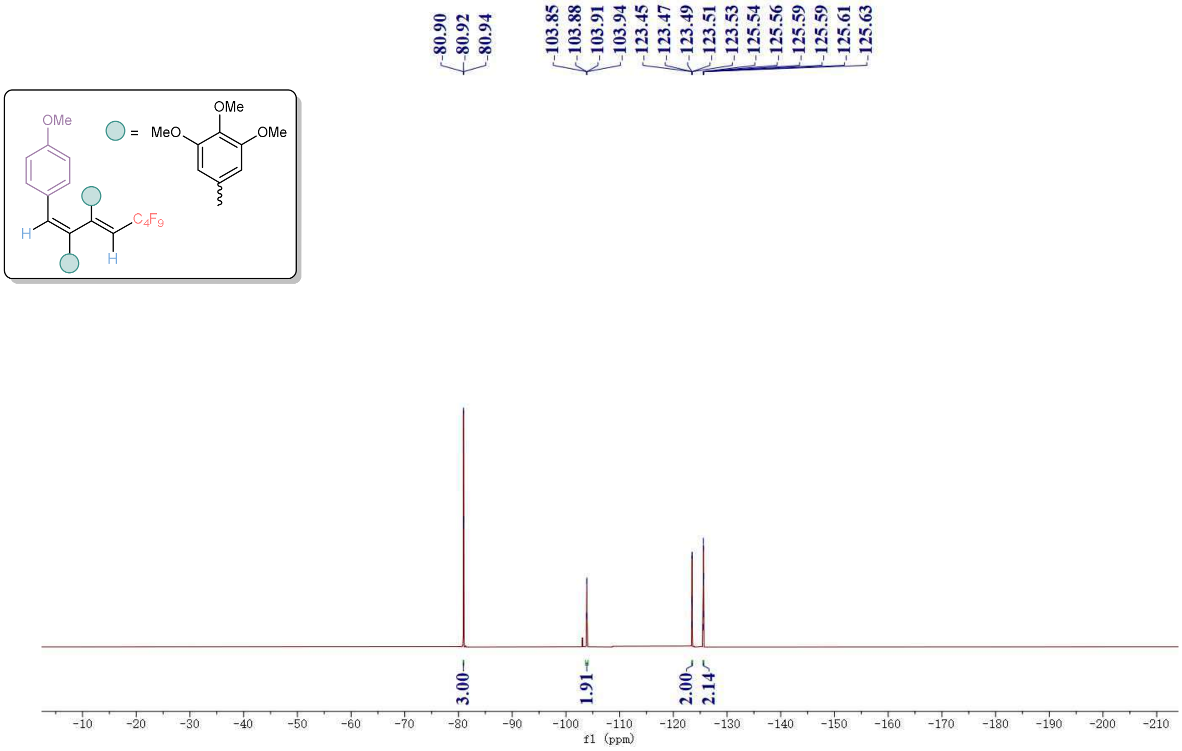

^{13}C NMR spectrum of **27** (126 MHz, Chloroform-*d*)

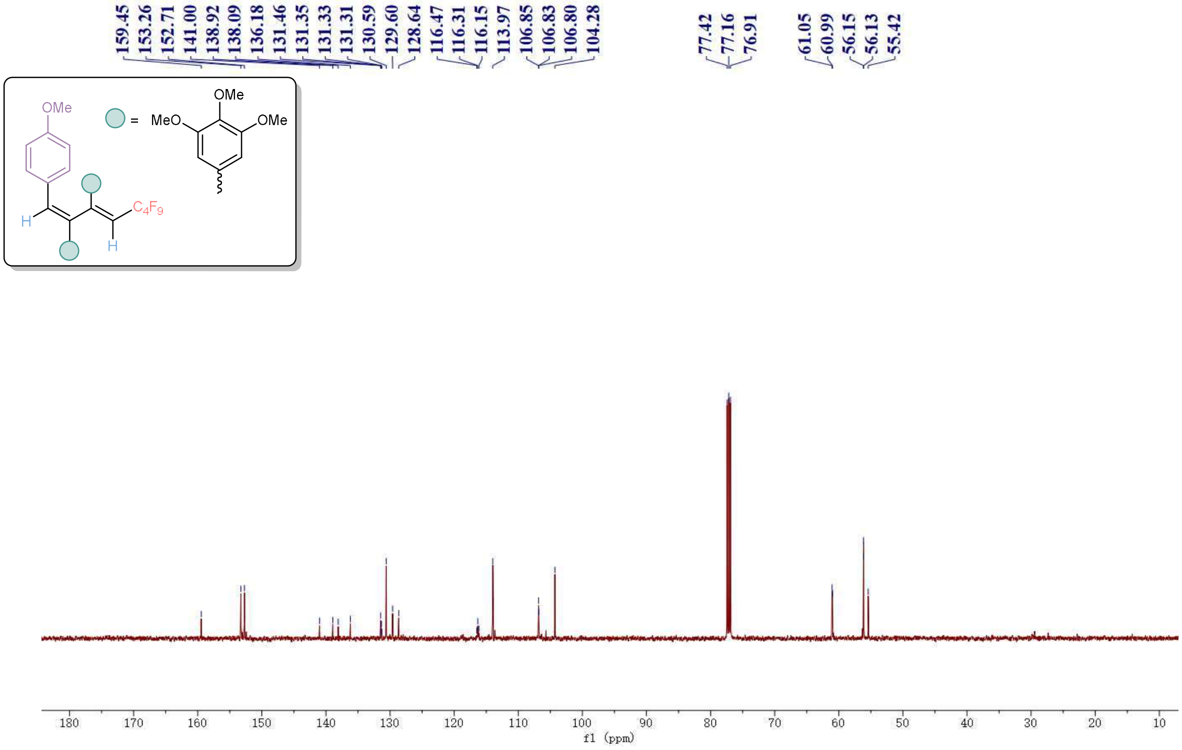

^{1}H NMR spectrum of **28** (500 MHz, Chloroform-*d*)

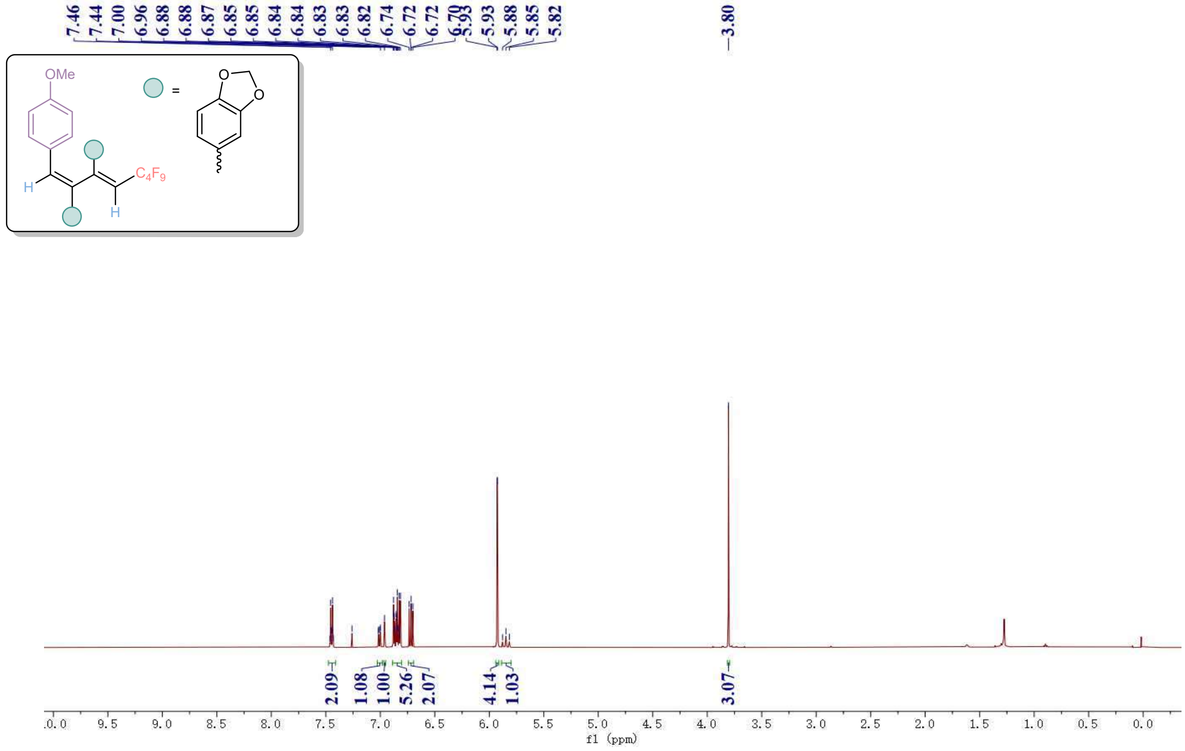

^{19}F NMR Spectrum of **28** (471 MHz, Chloroform-*d*)

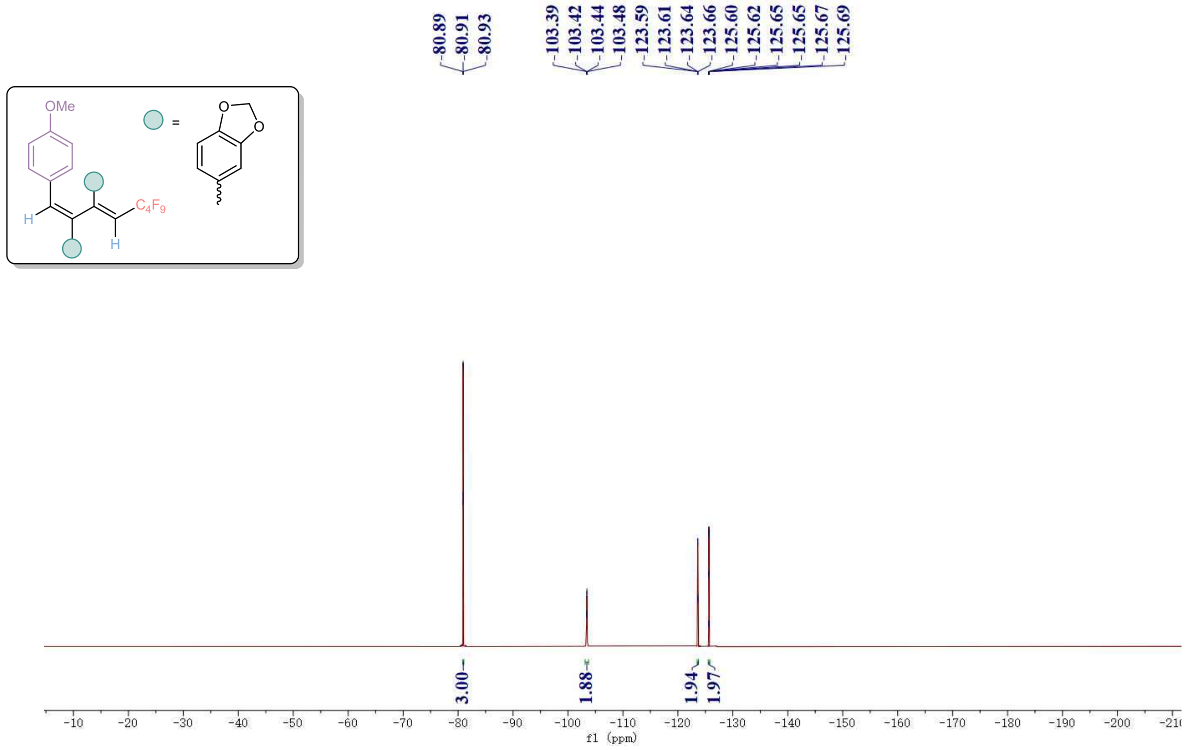

^{13}**C NMR** spectrum of **28** (126 MHz, Chloroform-*d*)

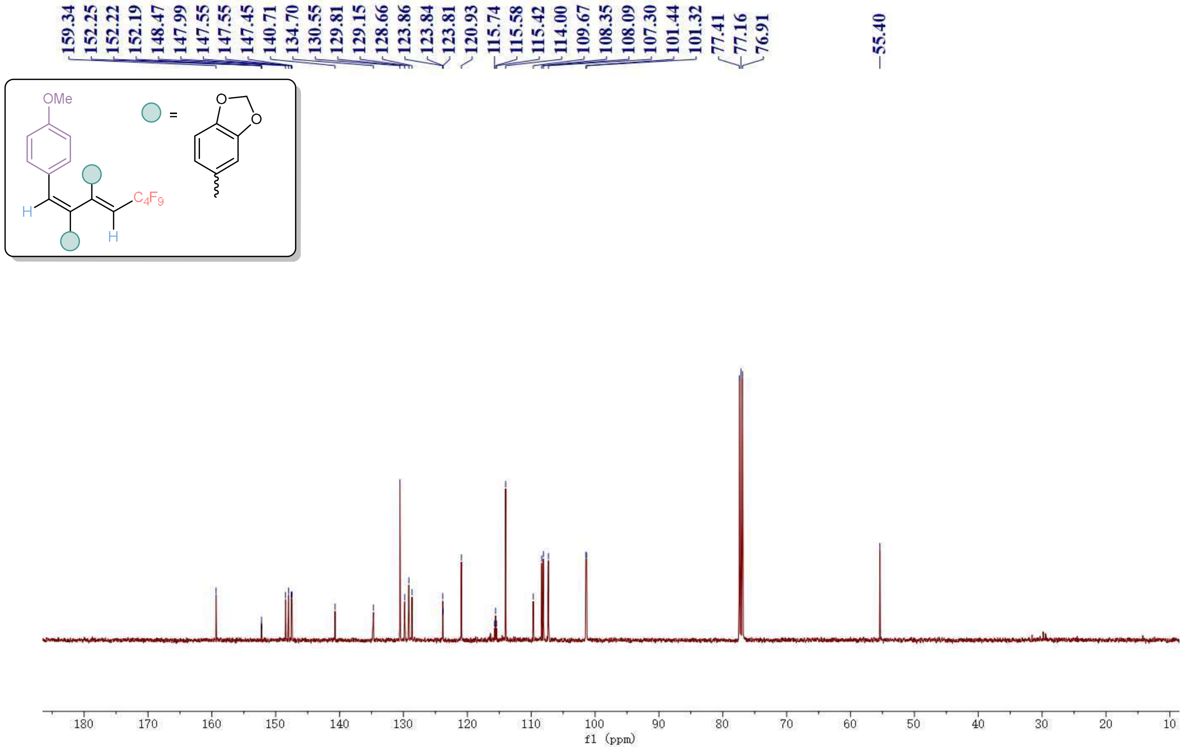

^{1}**H NMR** spectrum of **29** (500 MHz, Chloroform-*d*)

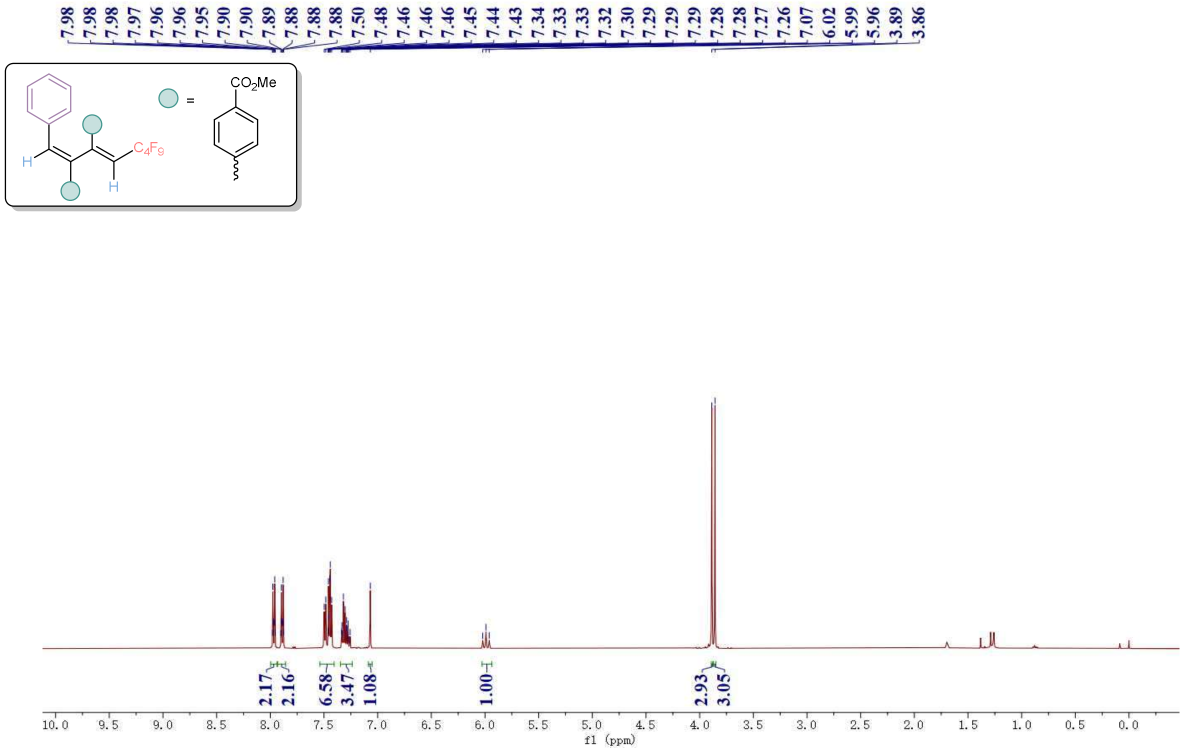

^{19}F NMR Spectrum of **29** (471 MHz, Chloroform-*d*)

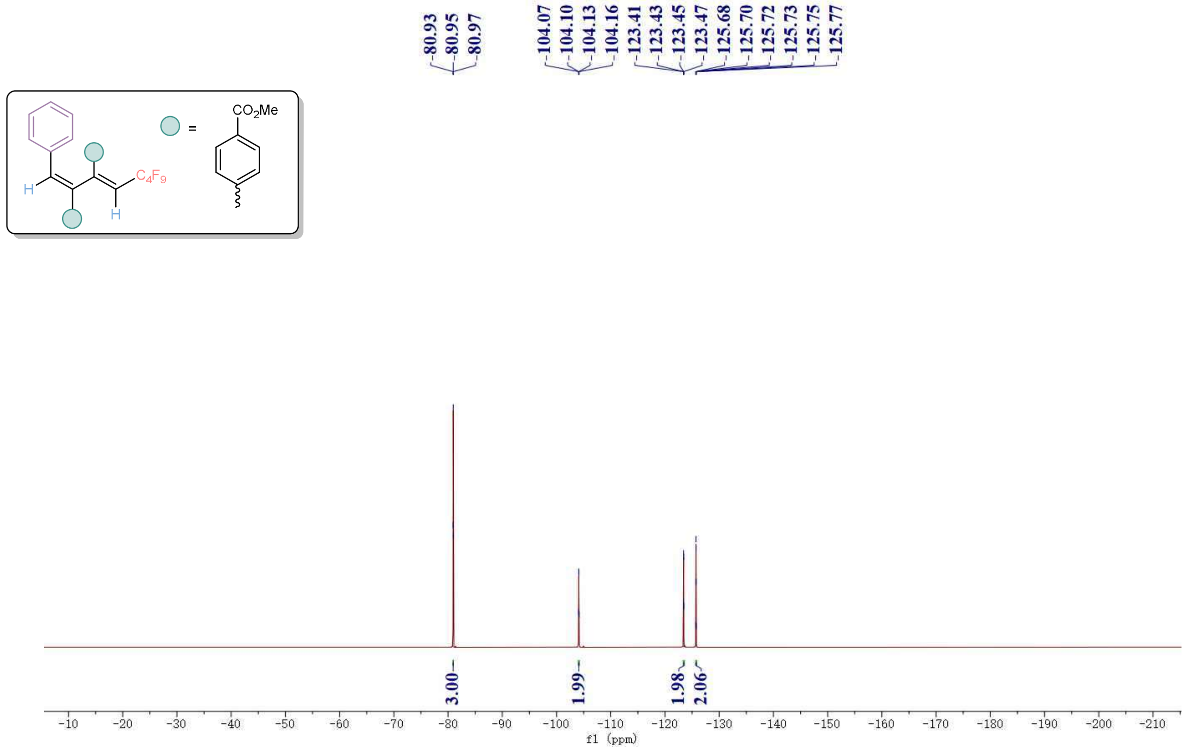

^{13}C NMR spectrum of **29** (126 MHz, Chloroform-*d*)

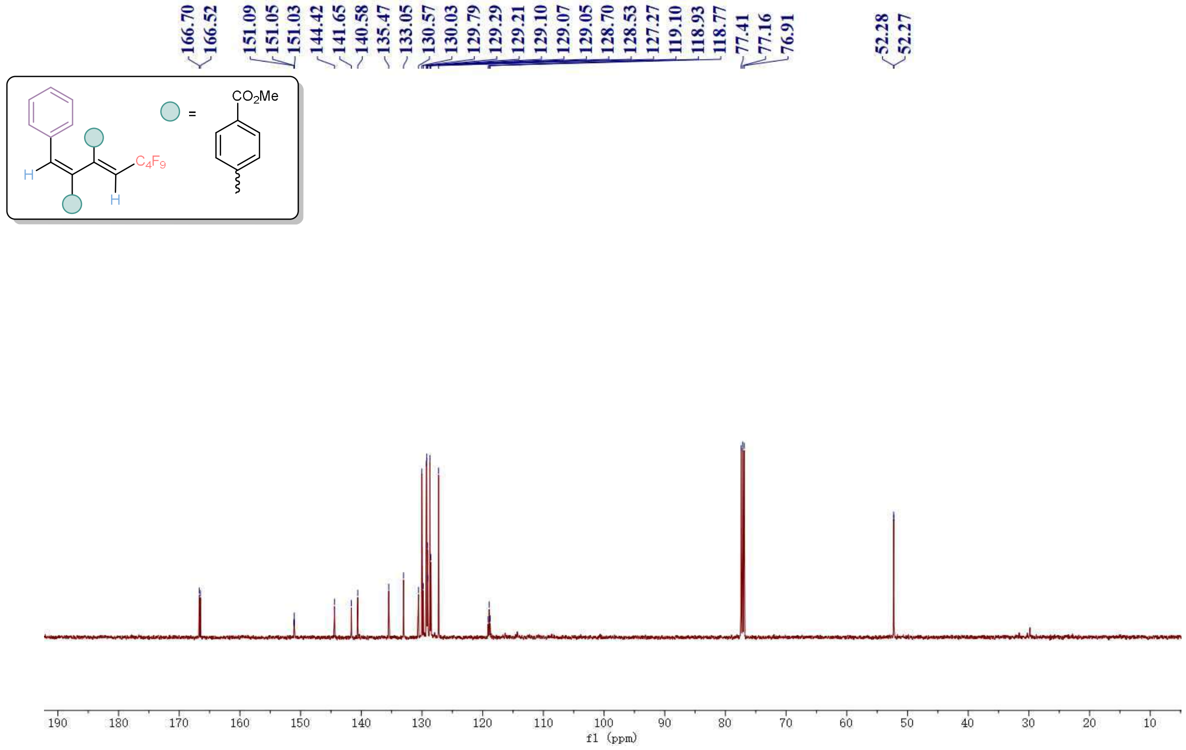

^{1}H NMR spectrum of **30** (500 MHz, Chloroform-*d*)

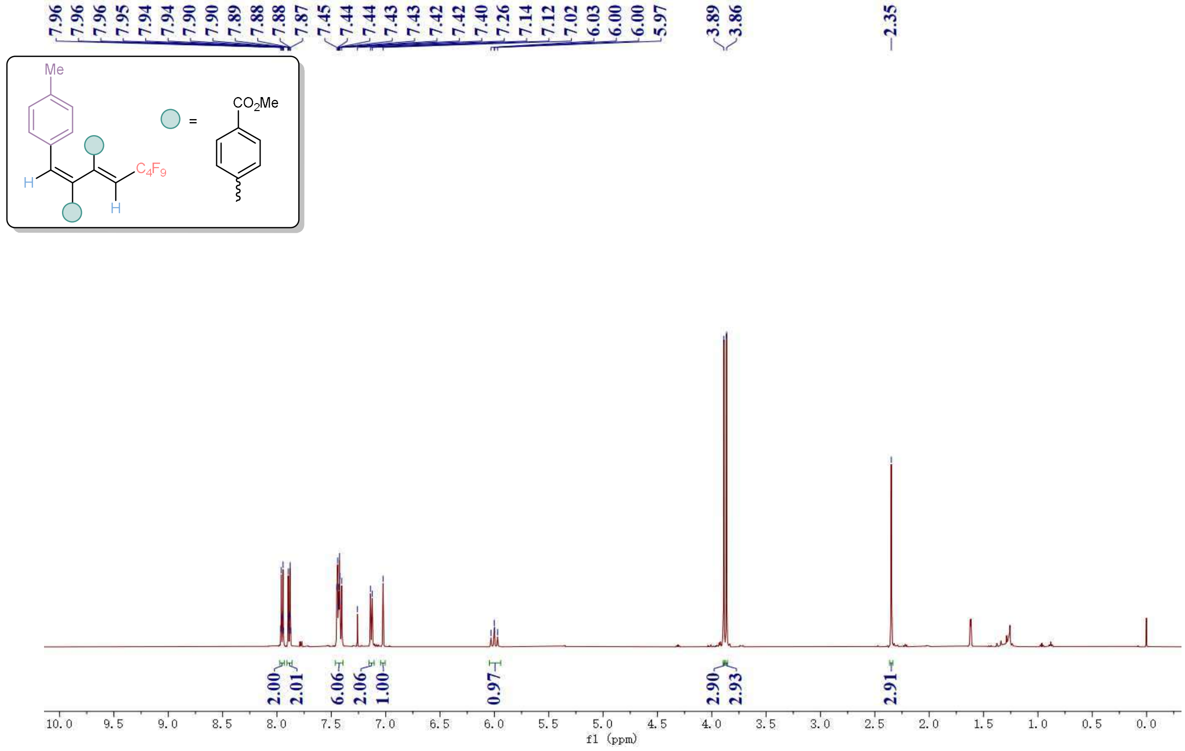

^{19}F NMR Spectrum of **30** (471 MHz, Chloroform-*d*)

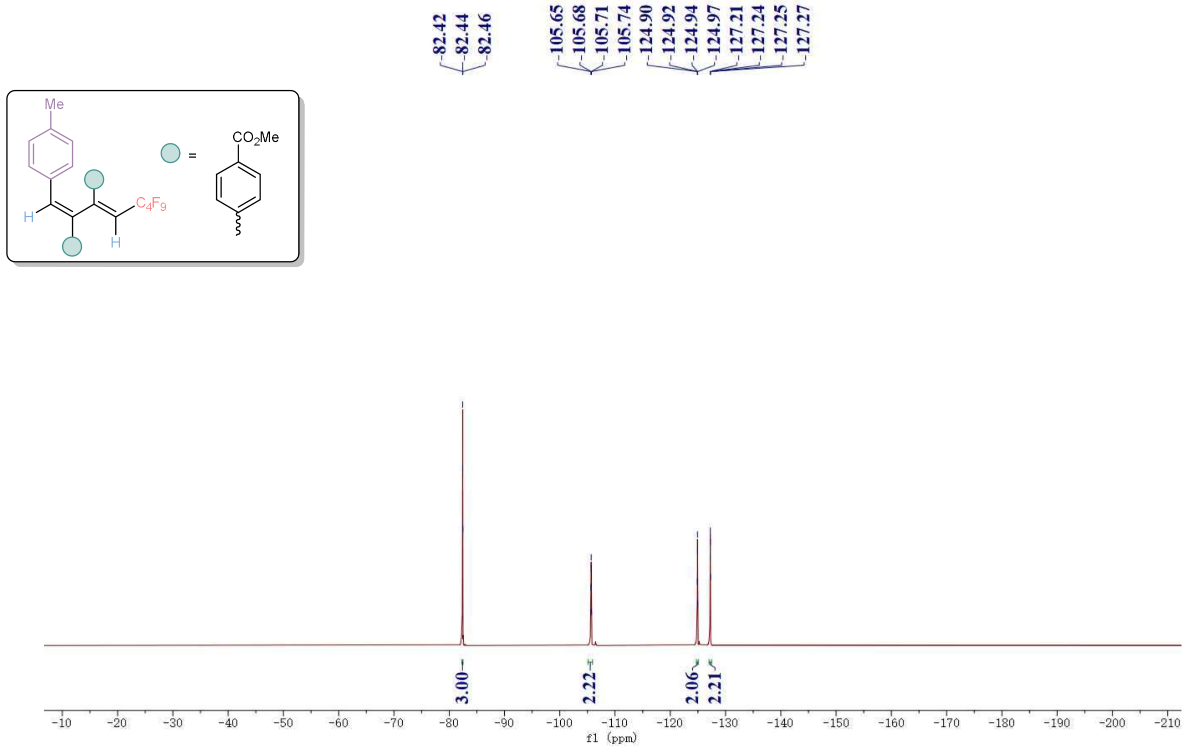

^{13}**C NMR** spectrum of **30** (126 MHz, Chloroform-*d*)

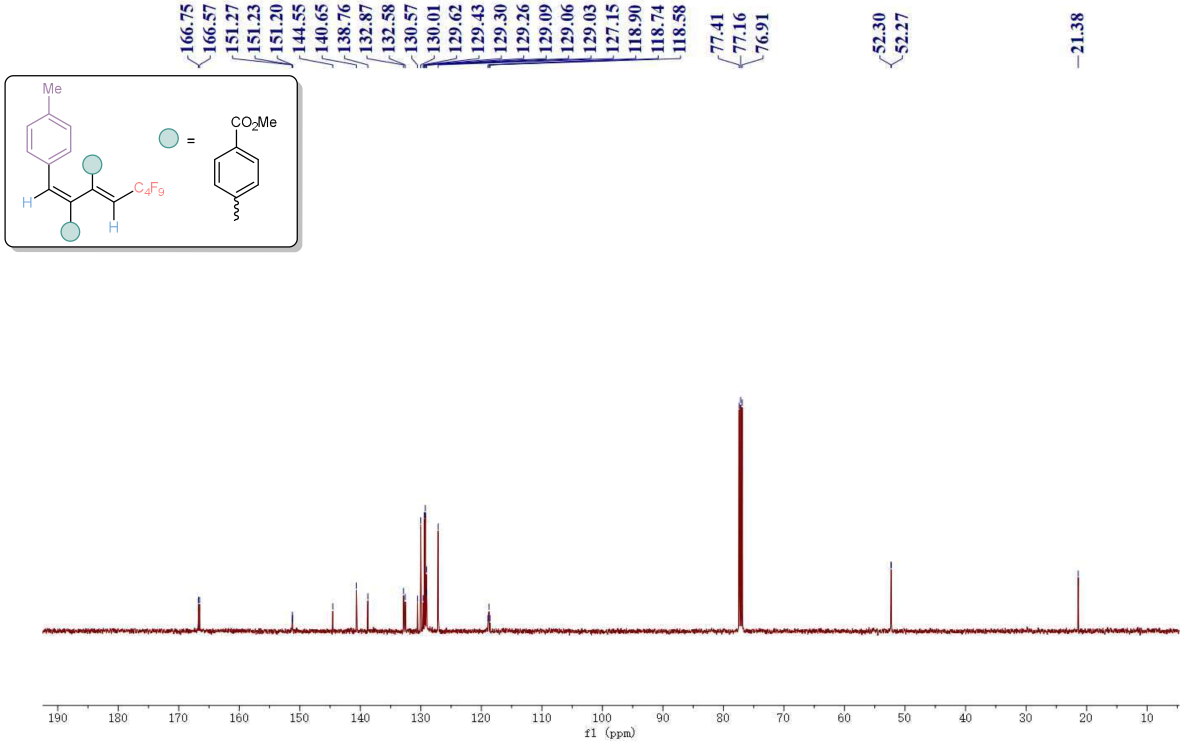

^{1}**H NMR** spectrum of **31** (500 MHz, Chloroform-*d*)

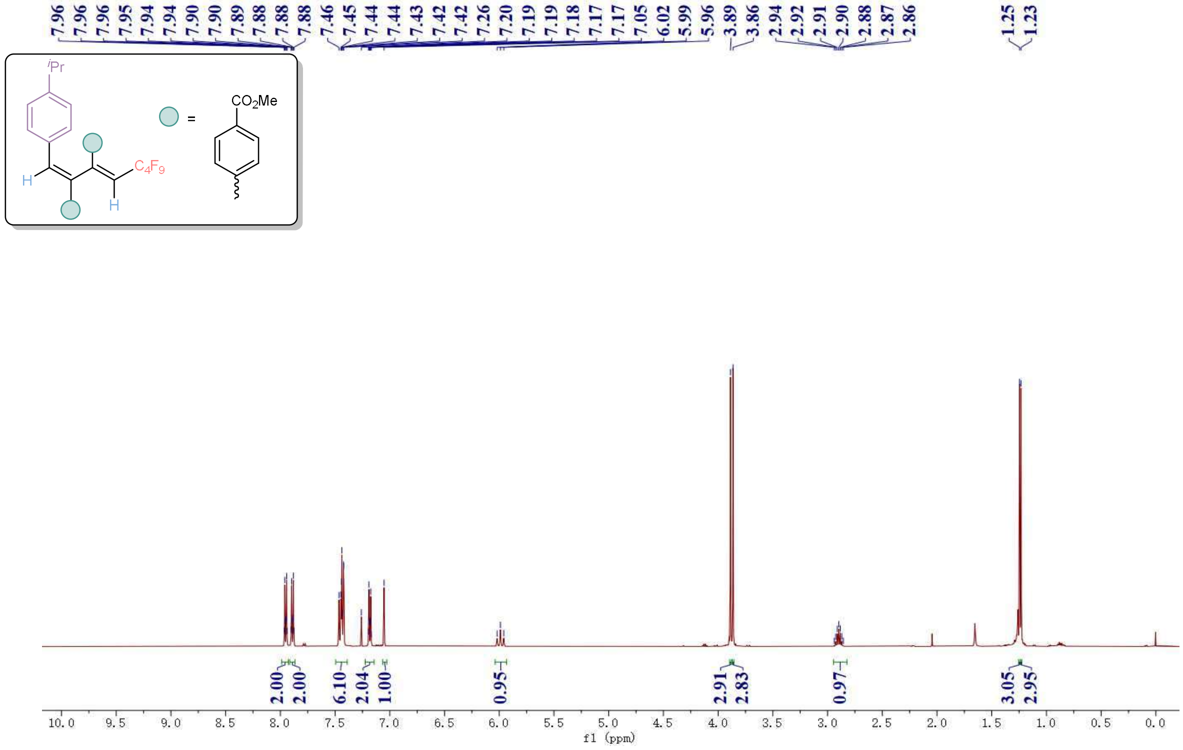

^{19}F NMR Spectrum of **31** (471 MHz, Chloroform-*d*)

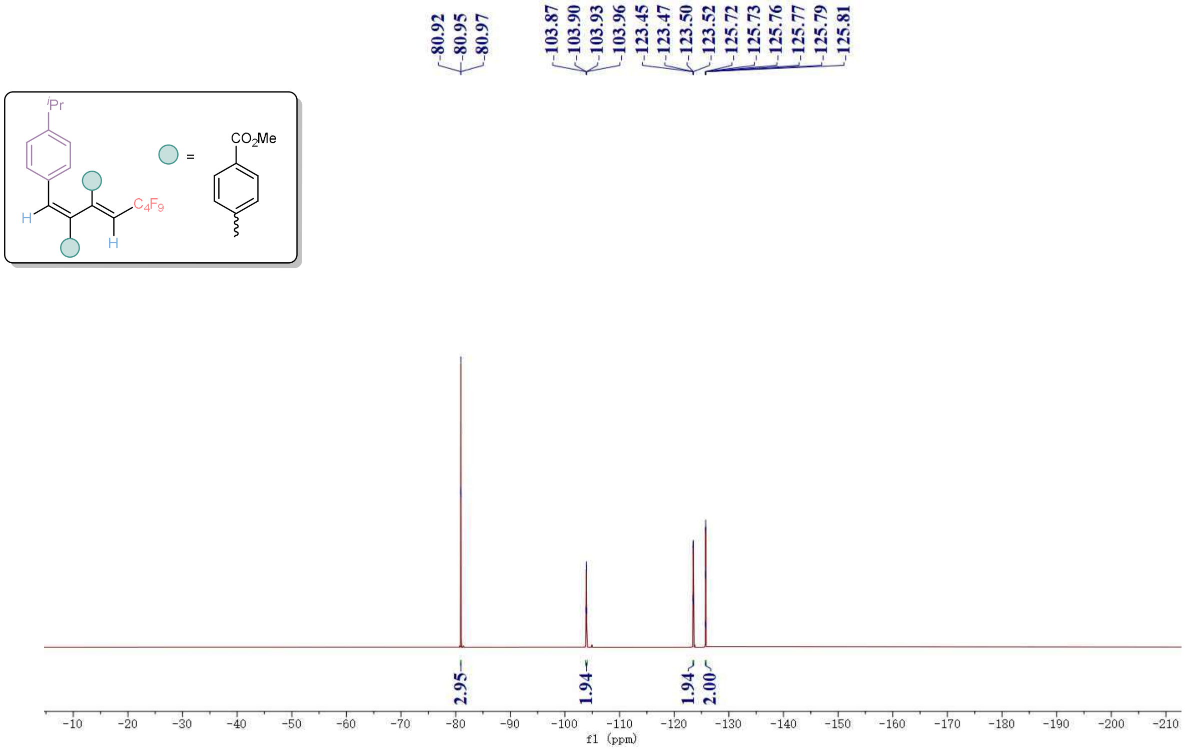

^{13}C NMR spectrum of **31** (126 MHz, Chloroform-*d*)

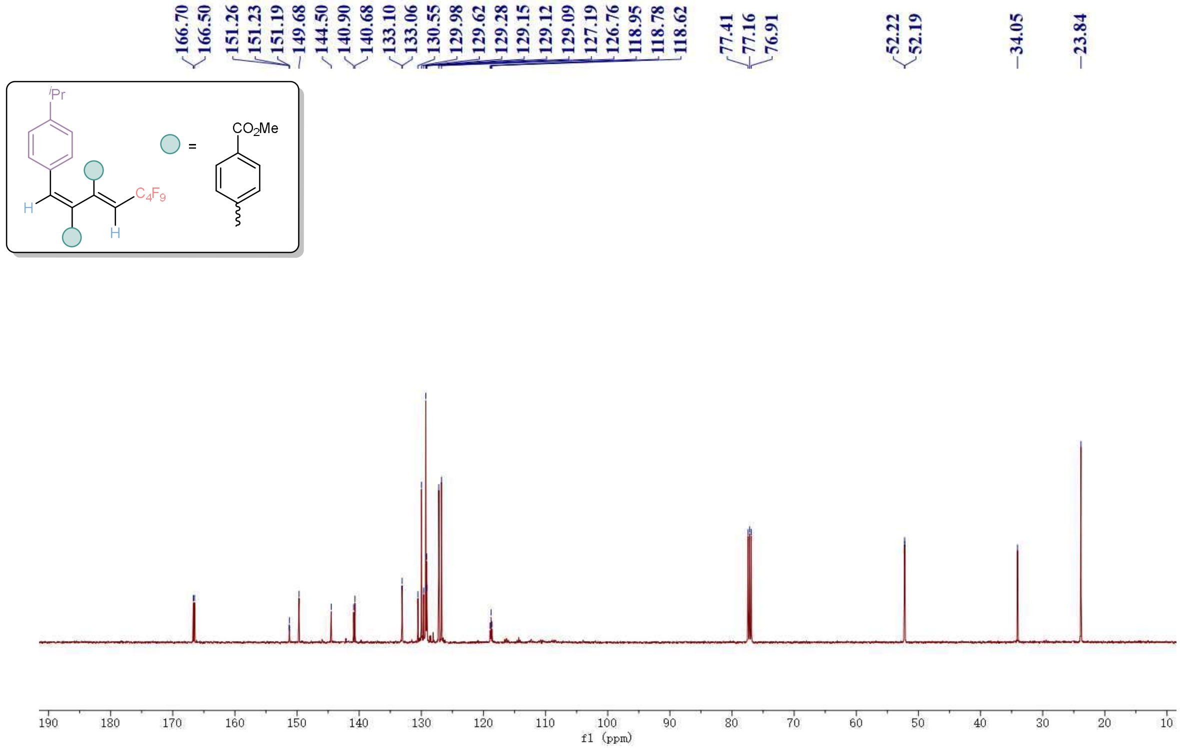

^{1}**H NMR** spectrum of **32** (500 MHz, Chloroform-*d*)

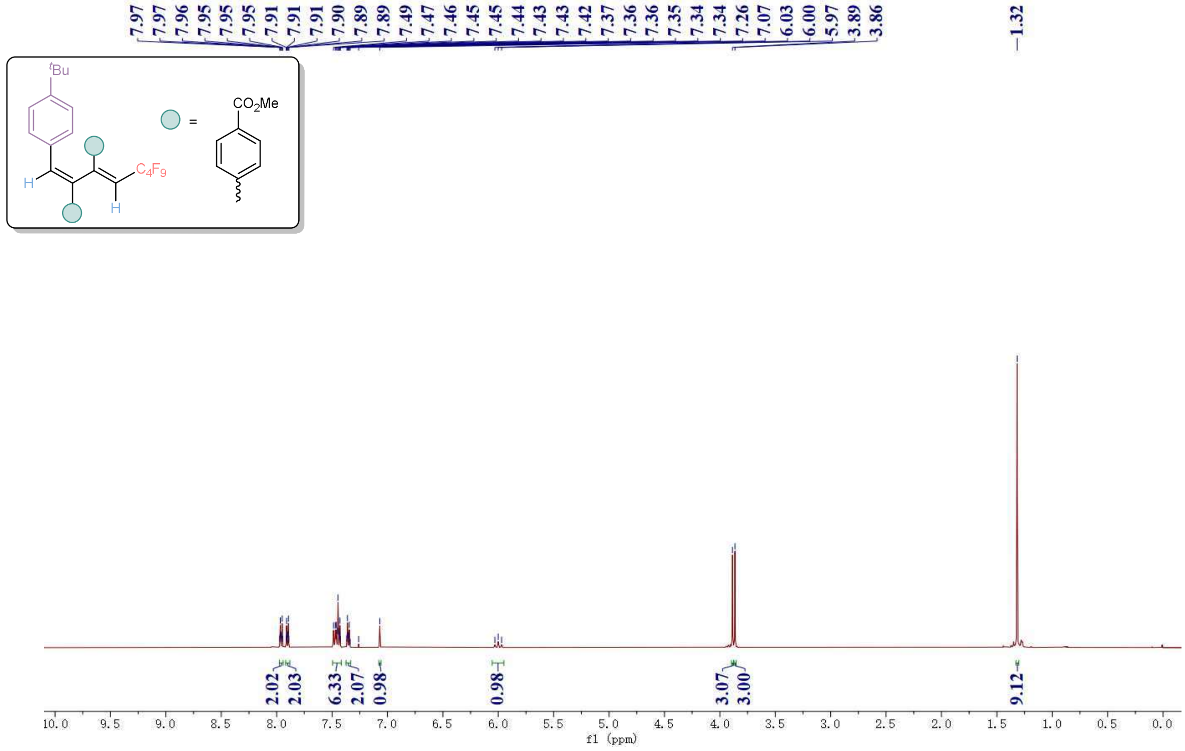

^{19}**F NMR** Spectrum of **32** (471 MHz, Chloroform-*d*)

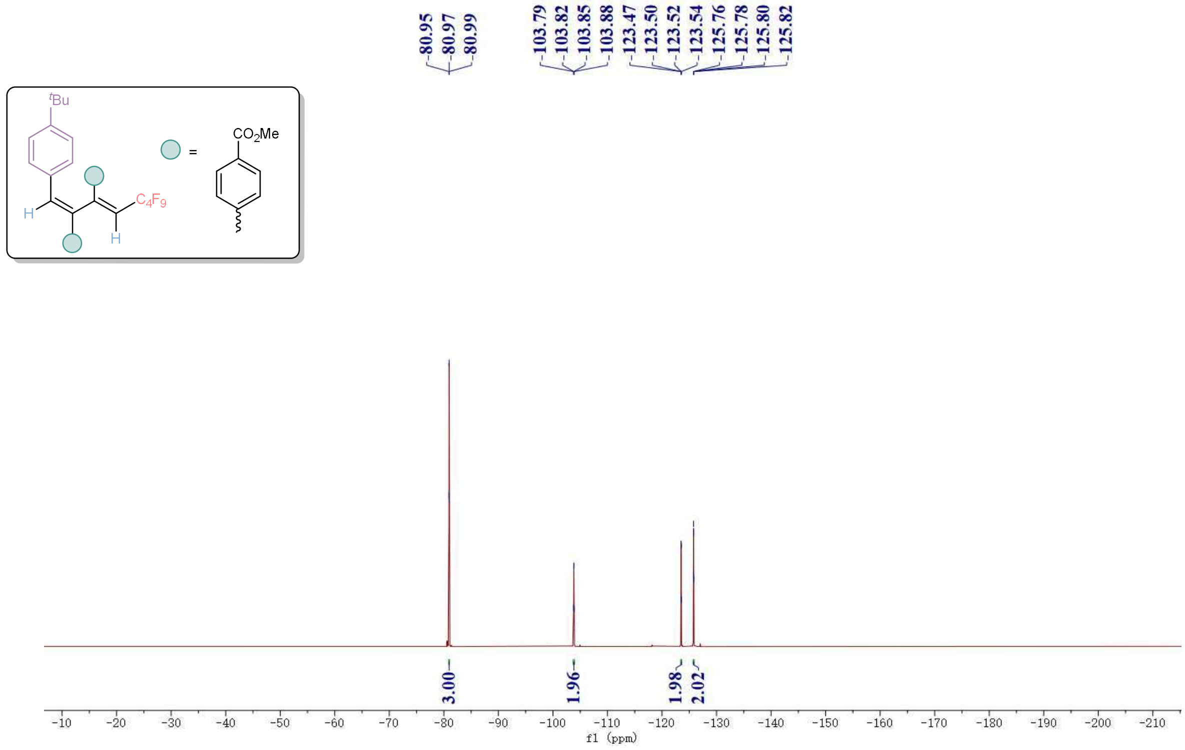

^{13}**C NMR** spectrum of **32** (126 MHz, Chloroform-*d*)

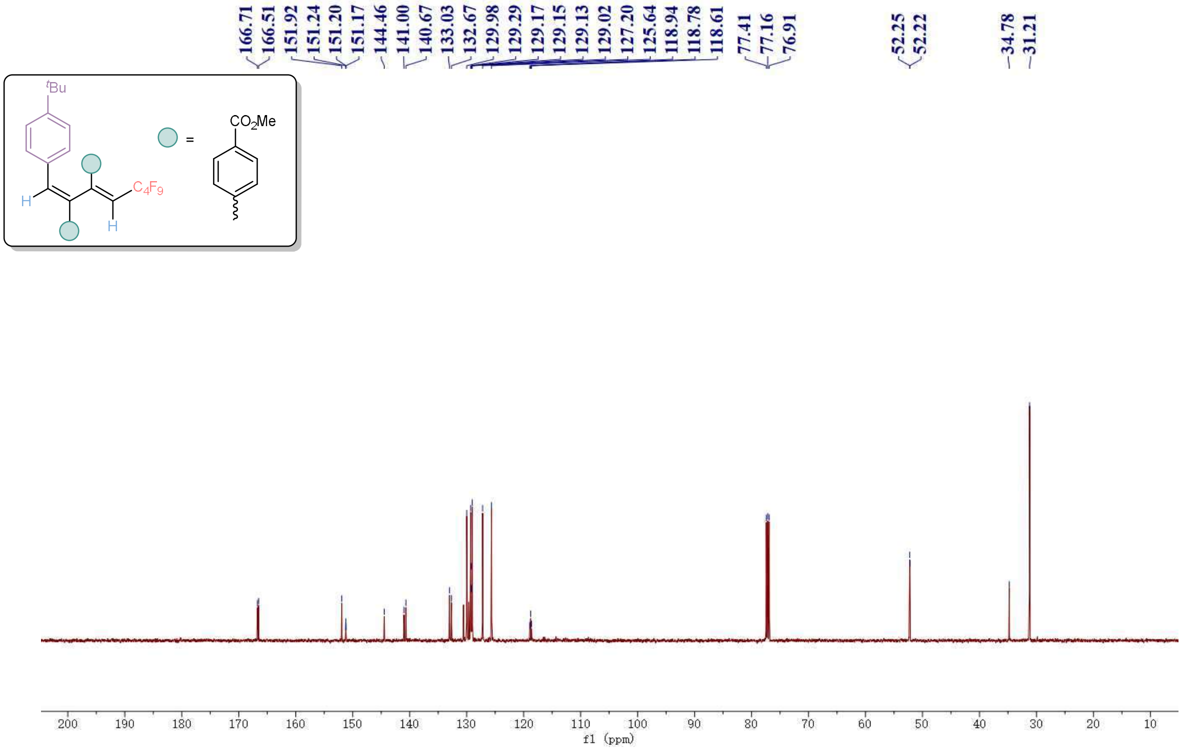

^{1}**H NMR** spectrum of **33** (500 MHz, Chloroform-*d*)

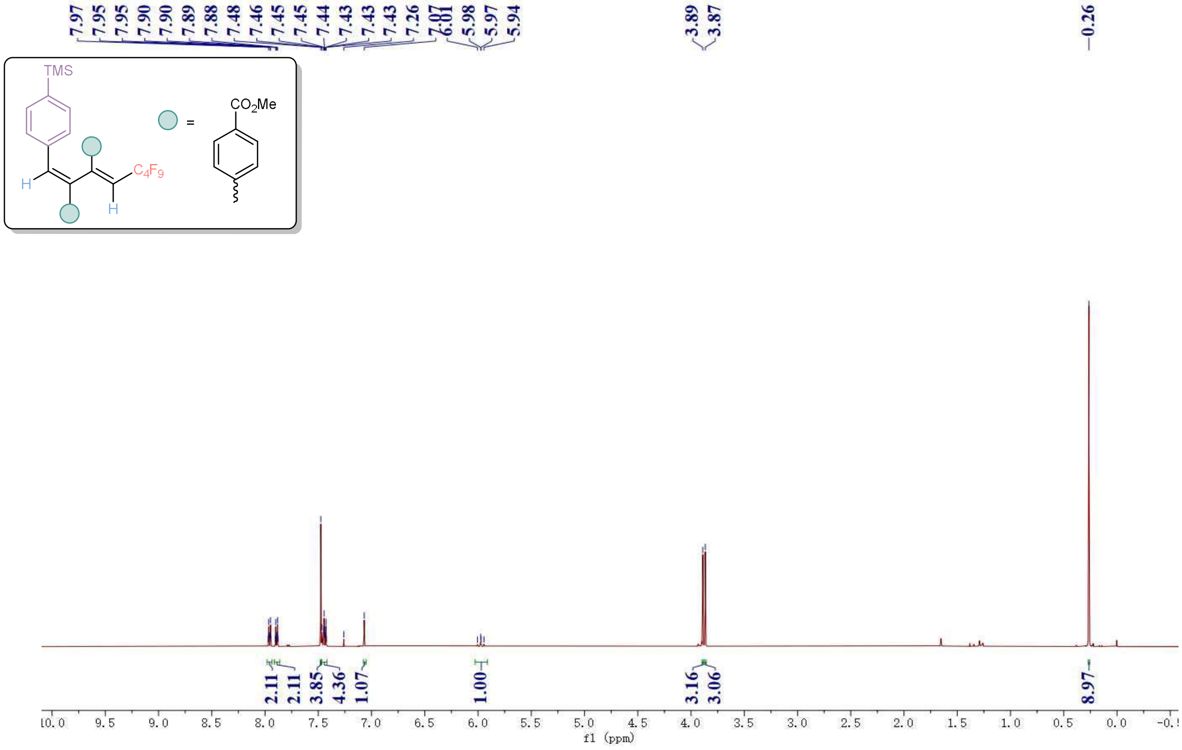

^{19}F NMR Spectrum of **33** (471 MHz, Chloroform-*d*)

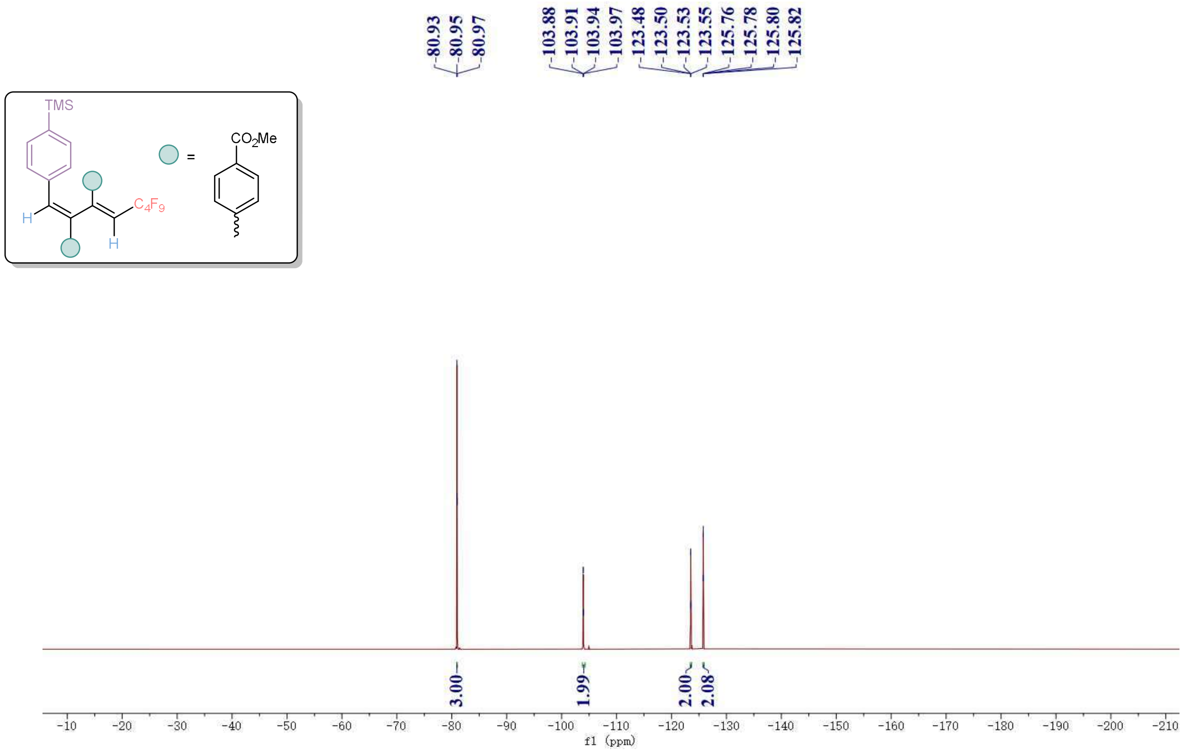

^{13}C NMR spectrum of **33** (126 MHz, Chloroform-d)

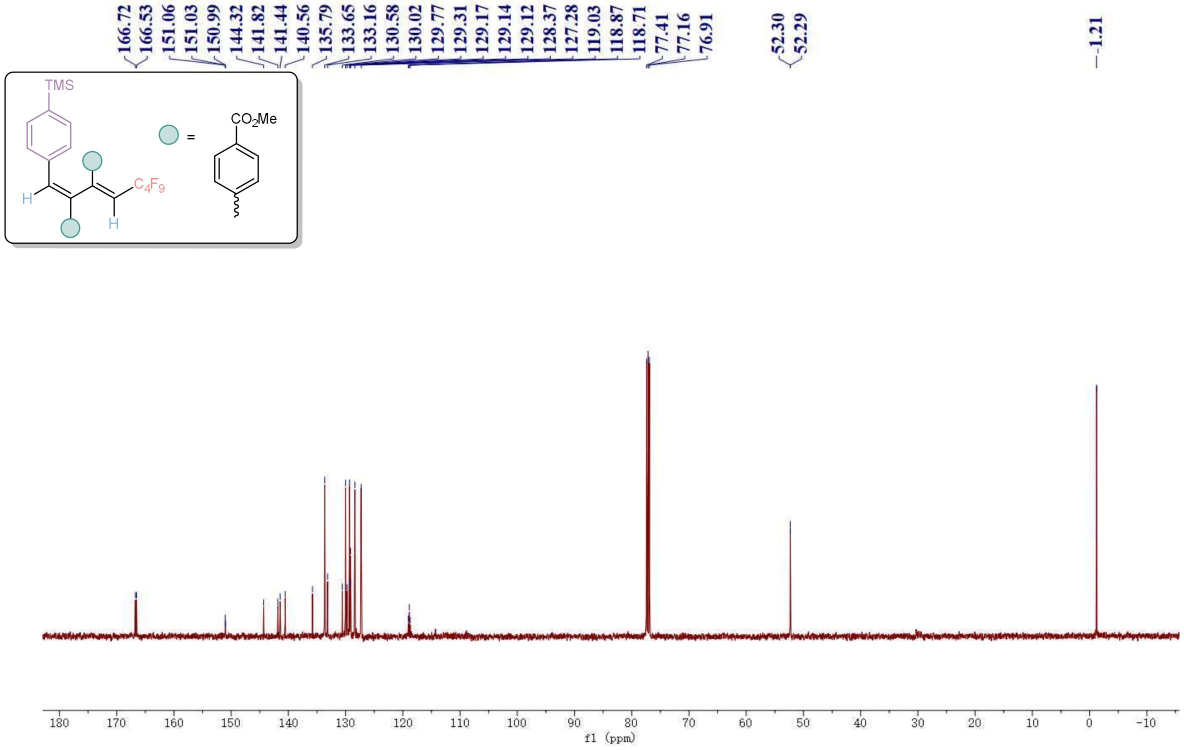

^{1}H NMR spectrum of **34** (500 MHz, Chloroform-*d*)

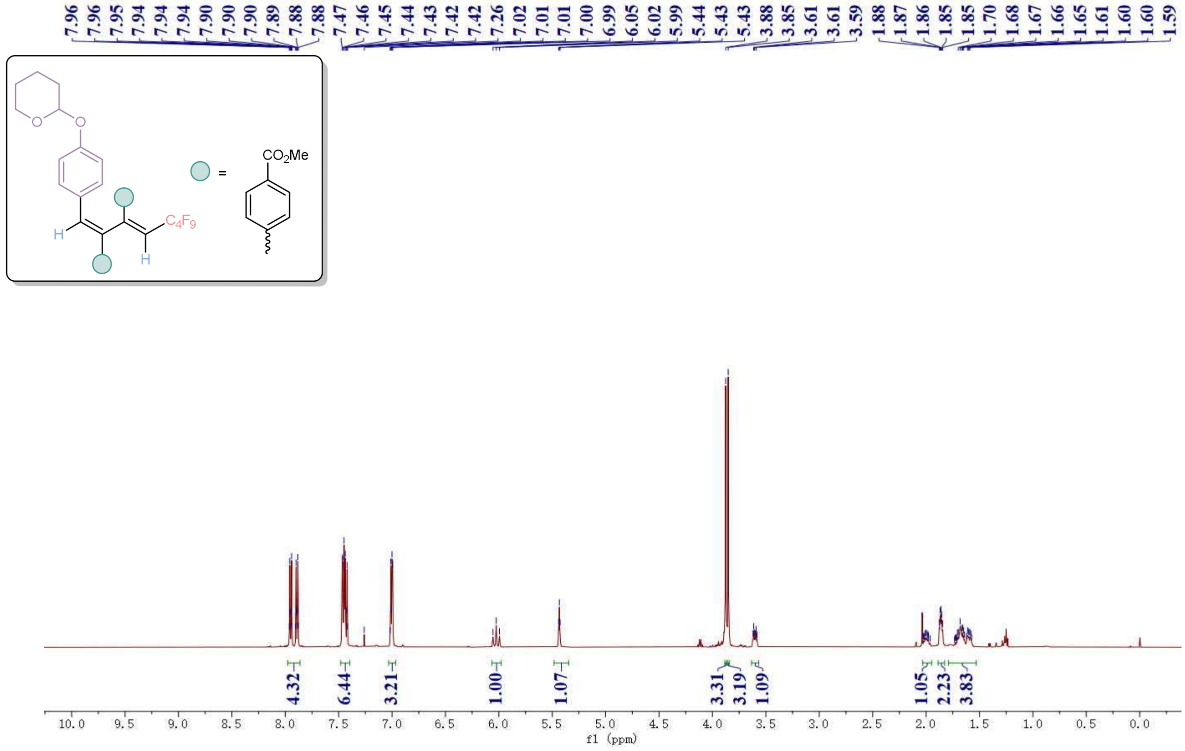

^{19}F NMR Spectrum of **34** (471 MHz, Chloroform-*d*)

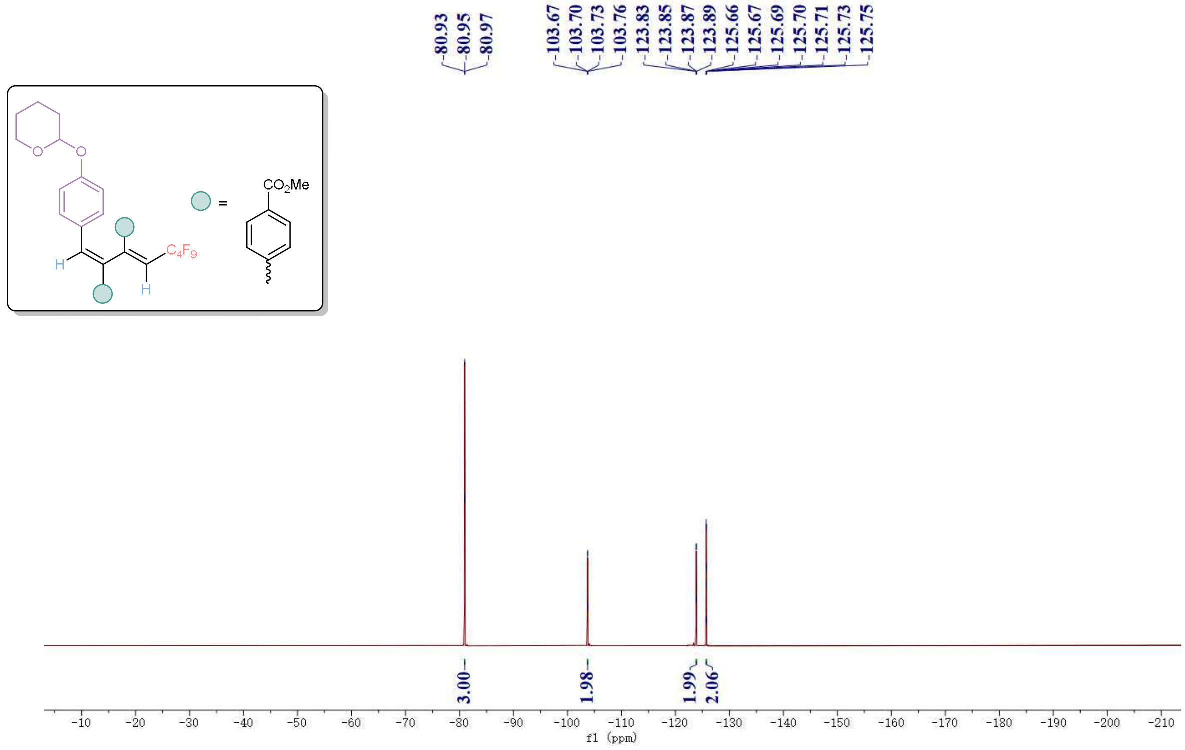

^{13}**C NMR** spectrum of **34** (126 MHz, Chloroform-*d*)

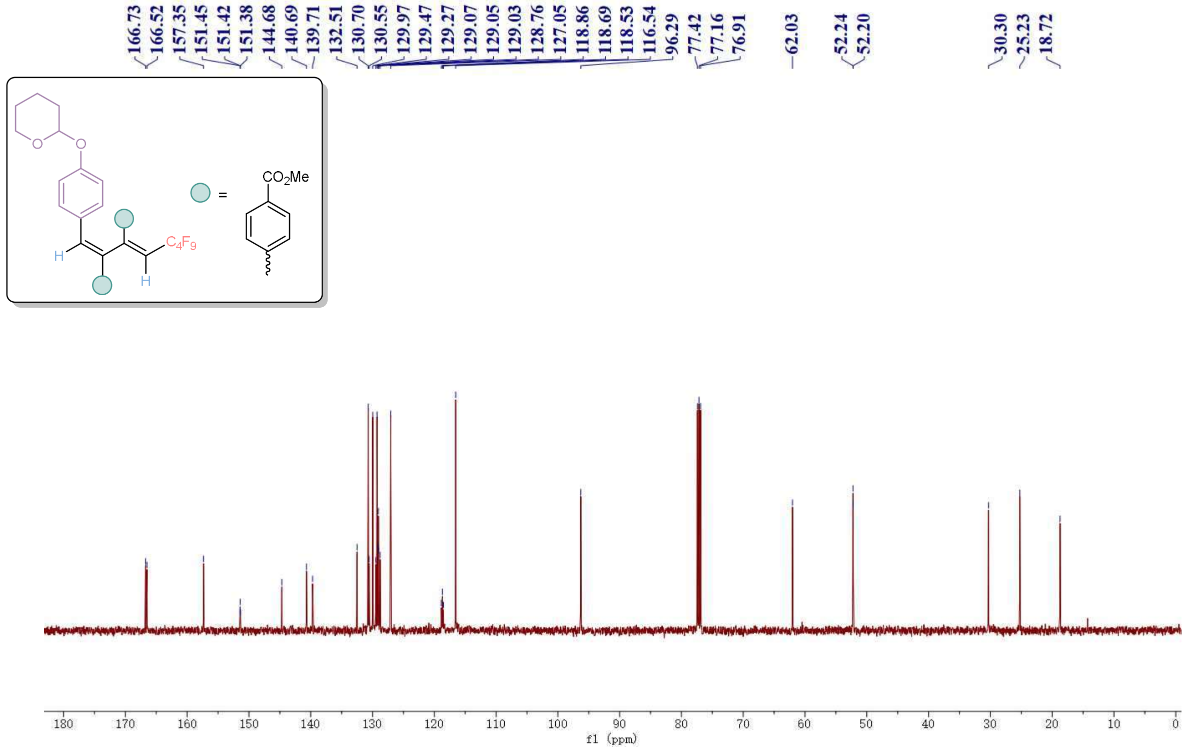

^{1}**H NMR** spectrum of **35** (500 MHz, Chloroform-*d*)

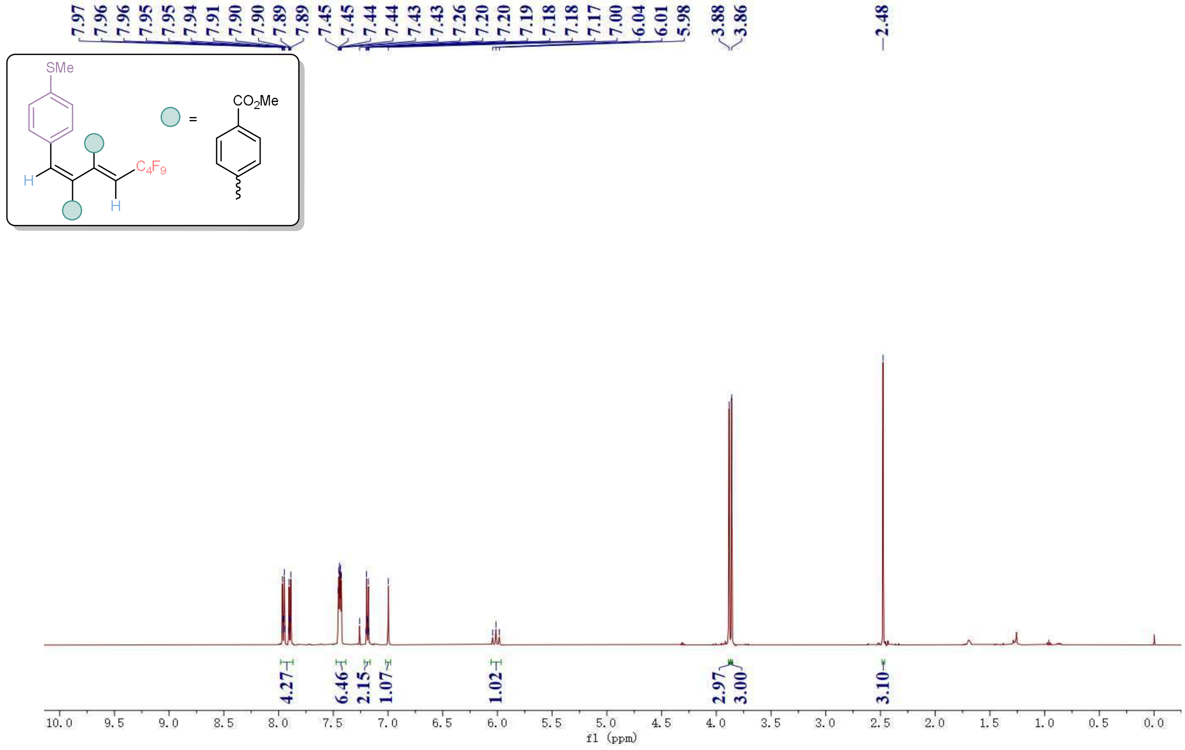

^{19}F NMR Spectrum of **35** (471 MHz, Chloroform-*d*)

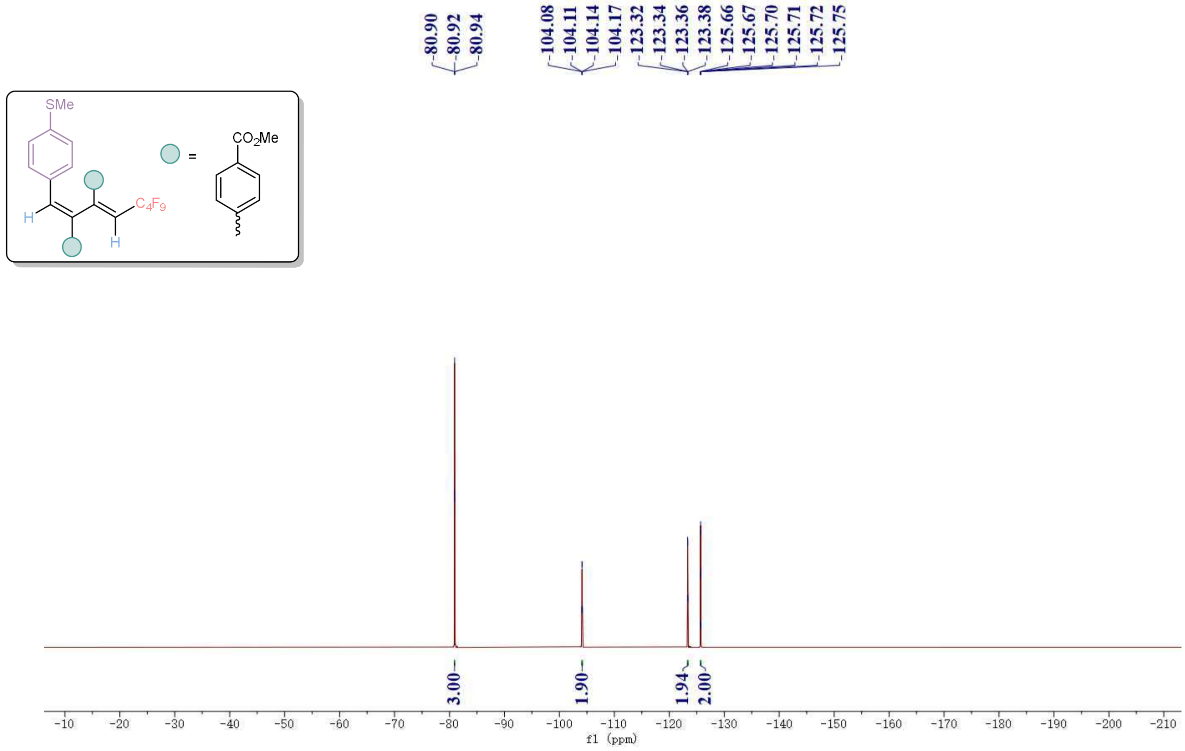

^{13}C NMR spectrum of **35** (126 MHz, Chloroform-*d*)

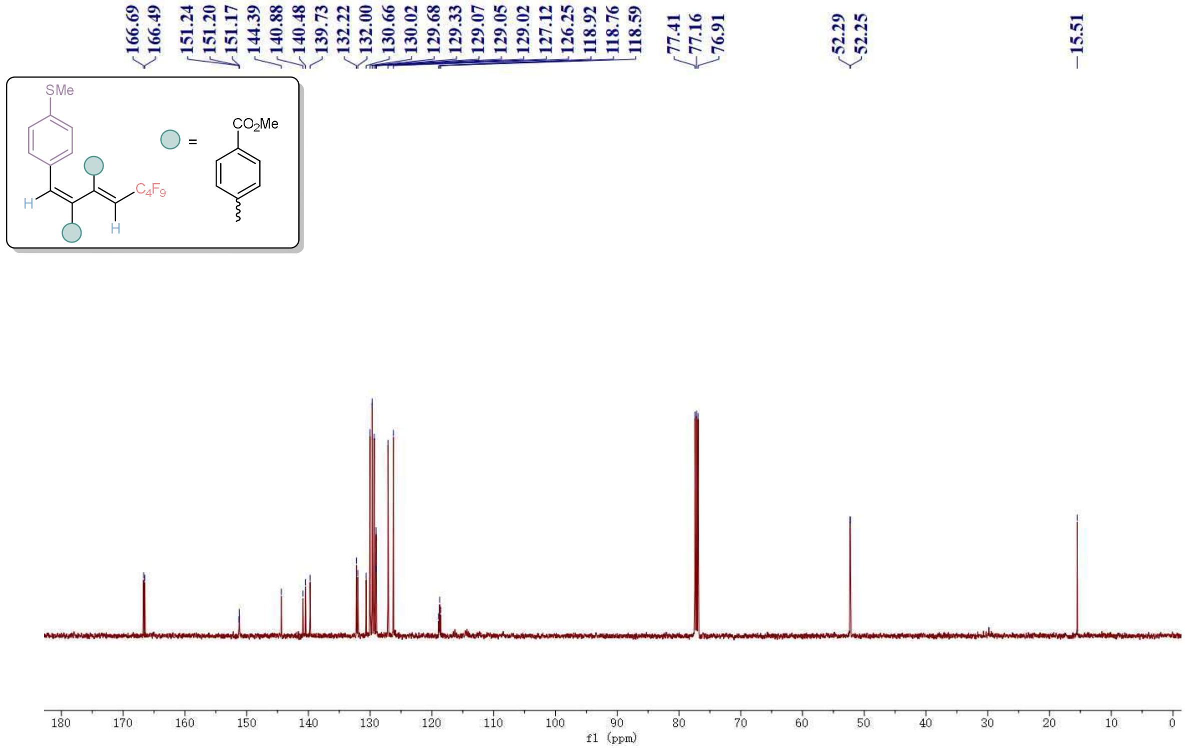

^{1}H NMR spectrum of **36** (500 MHz, Chloroform-*d*)

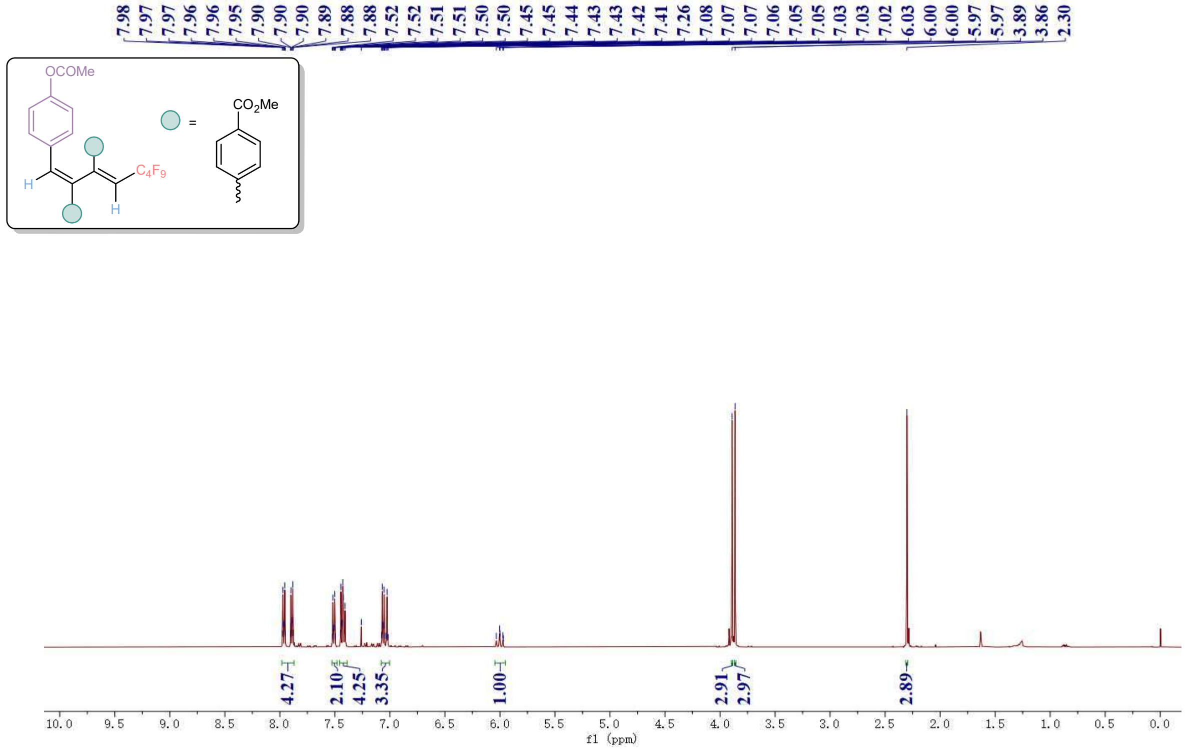

^{19}F NMR Spectrum of **36** (471 MHz, Chloroform-*d*)

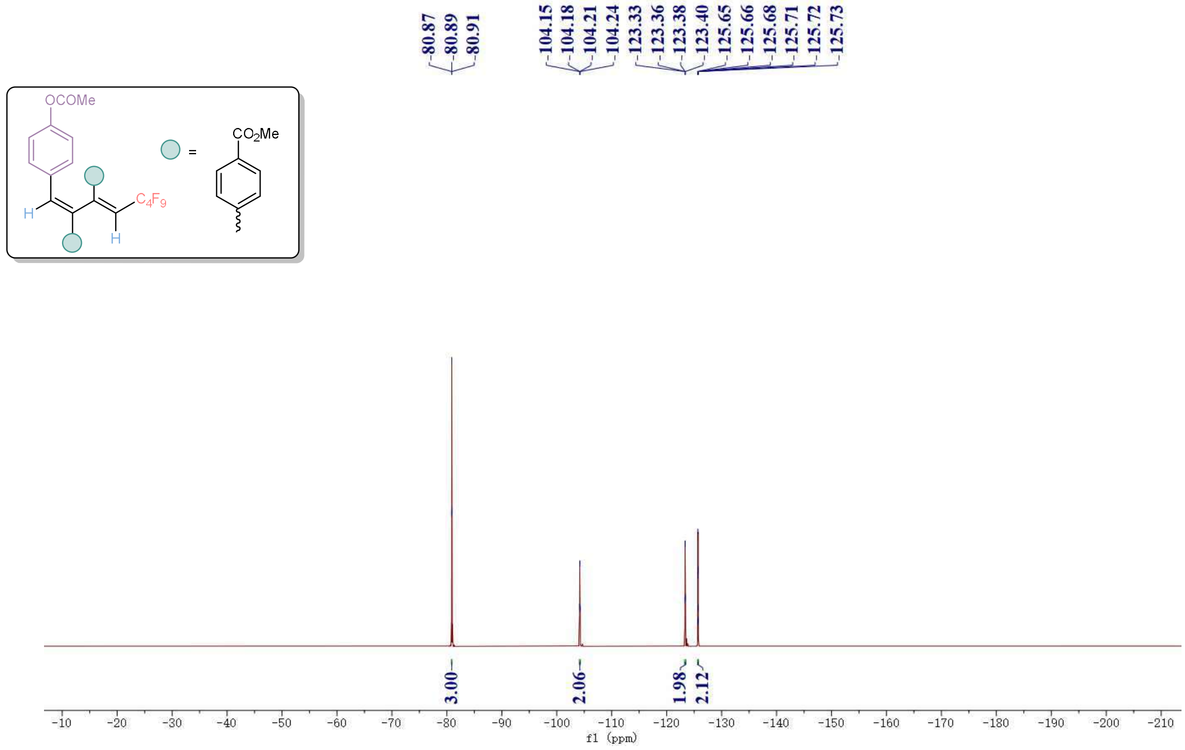

^{13}**C NMR** spectrum of **36** (126 MHz, Chloroform-*d*)

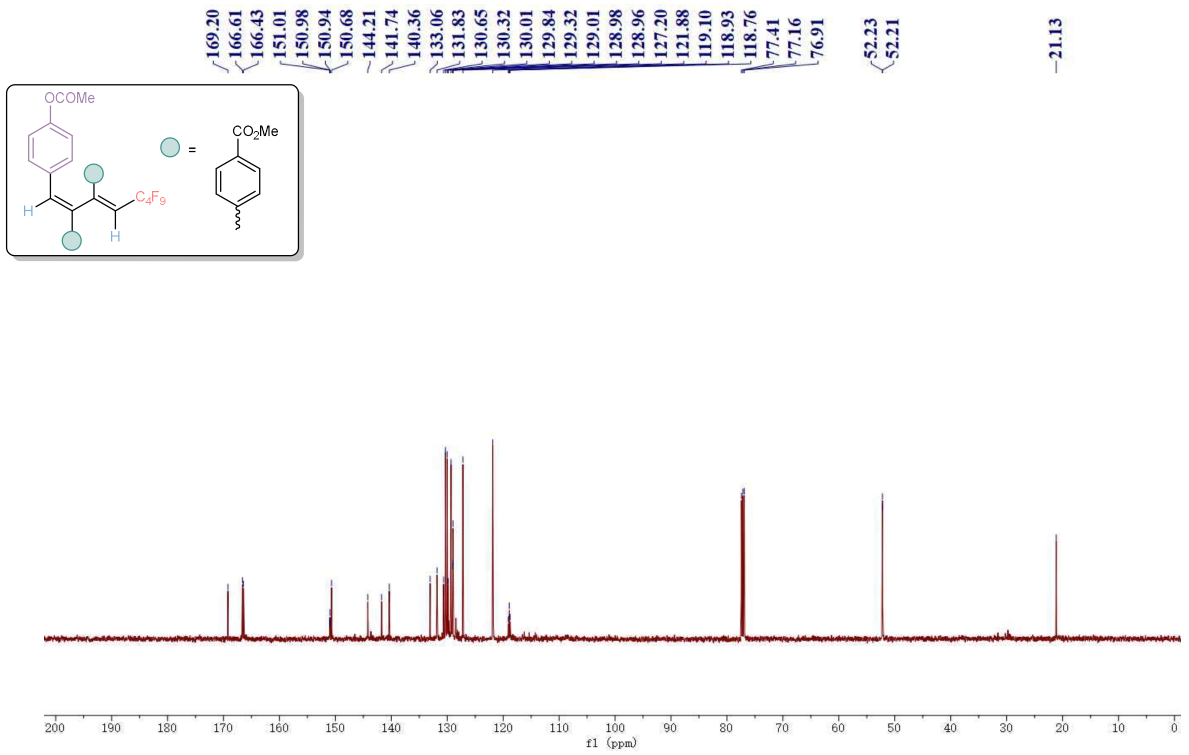

^{1}**H NMR** spectrum of **37** (500 MHz, Chloroform-*d*)

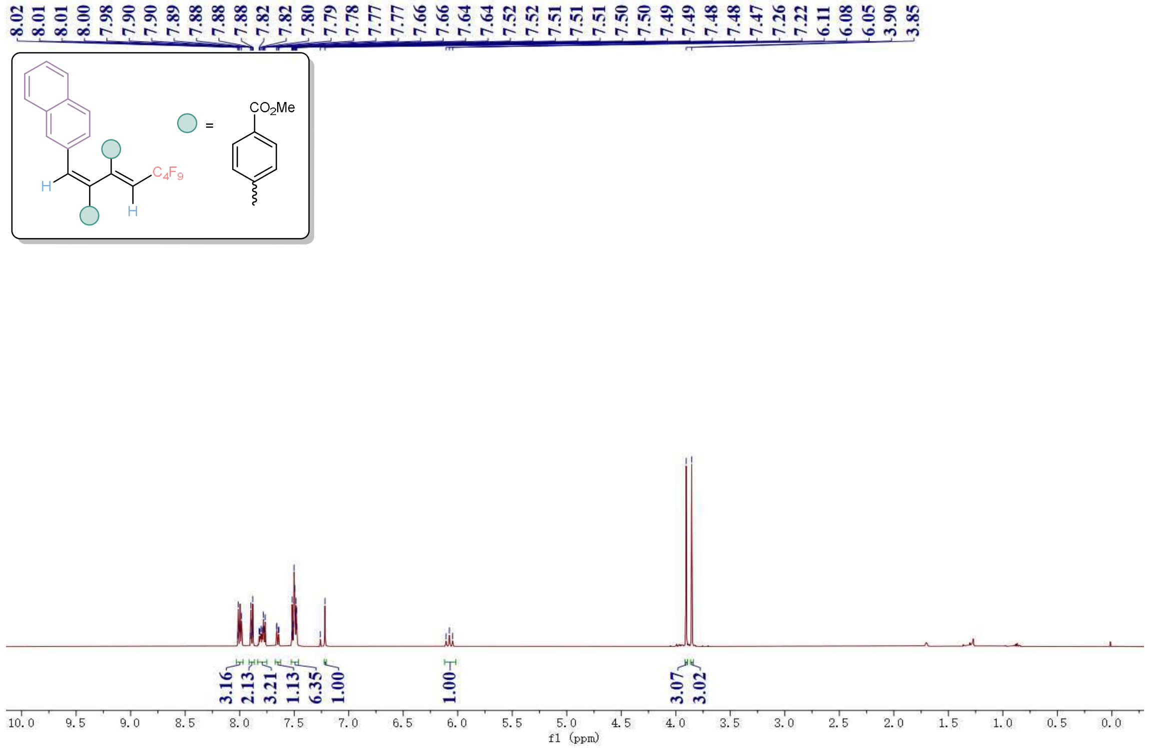

^{19}**F NMR** Spectrum of **37** (471 MHz, Chloroform-*d*)

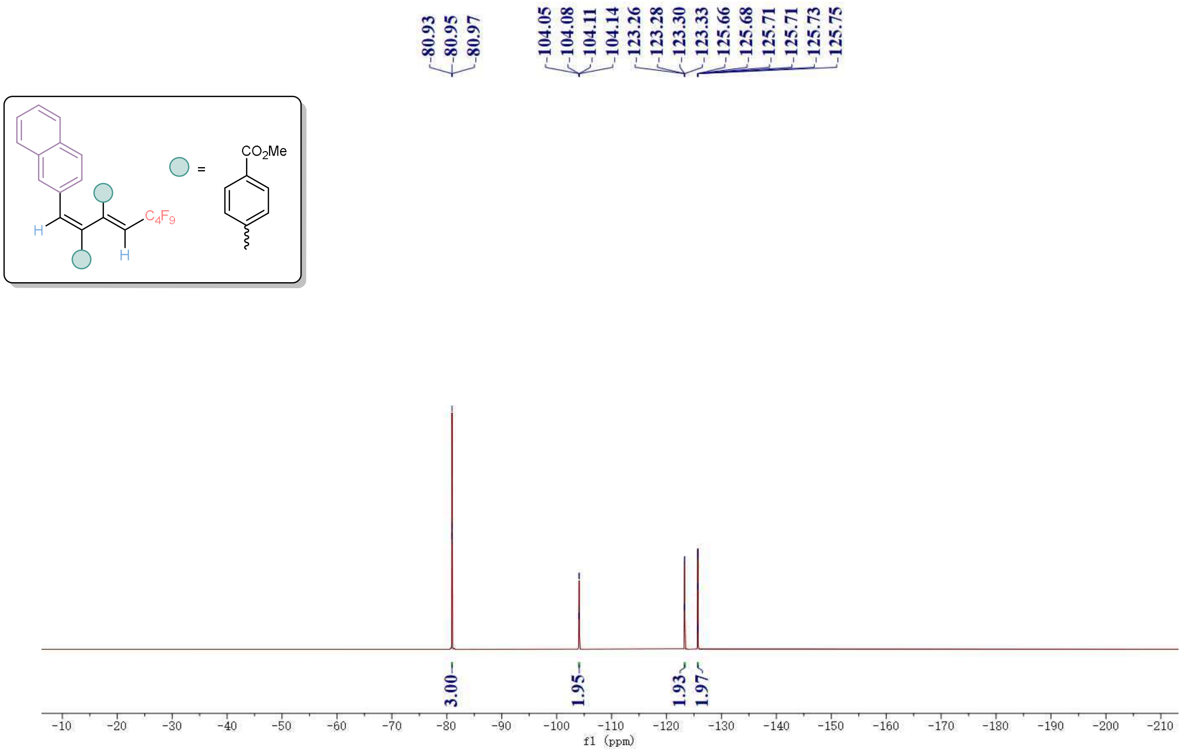

^{13}**C NMR** spectrum of **37** (126 MHz, Chloroform-*d*)

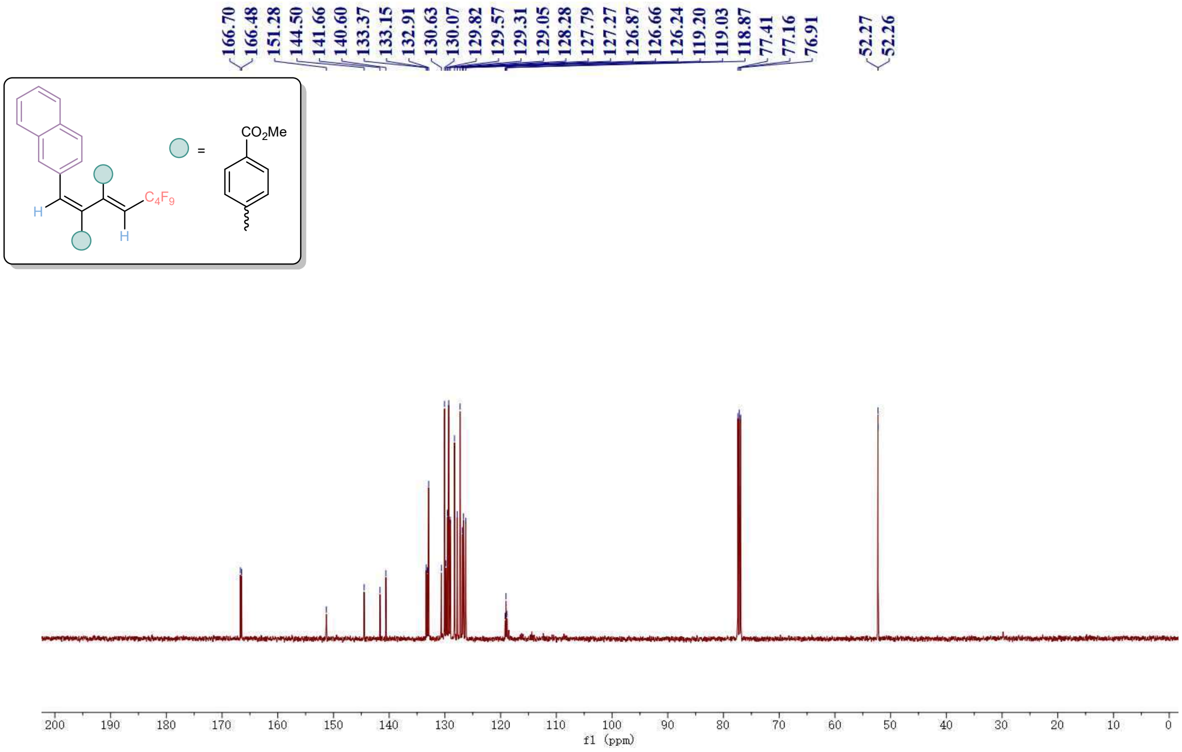

^{1}H NMR spectrum of **38** (500 MHz, Chloroform-*d*)

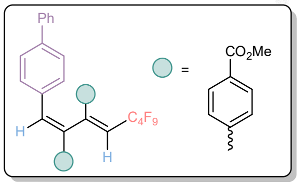

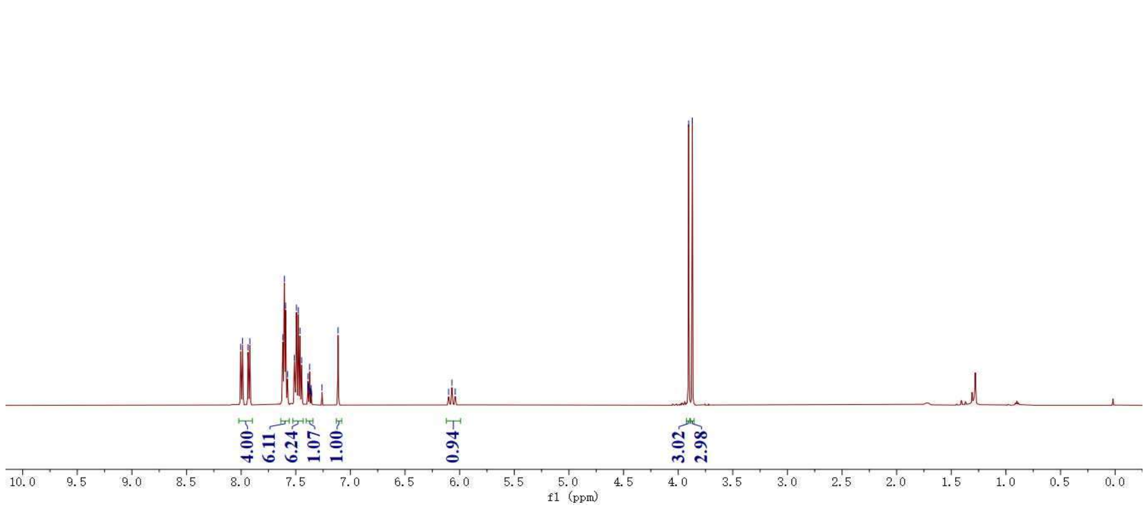

^{19}F NMR Spectrum of **38** (471 MHz, Chloroform-*d*)

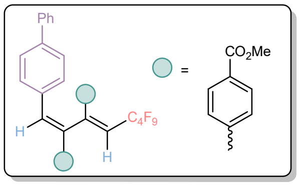

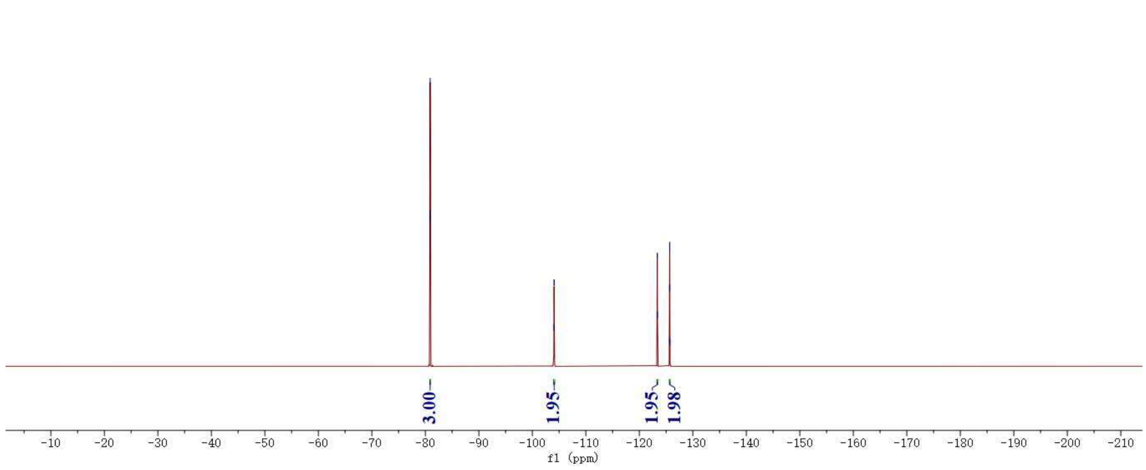

^{13}C NMR spectrum of **38** (126 MHz, Chloroform-*d*)

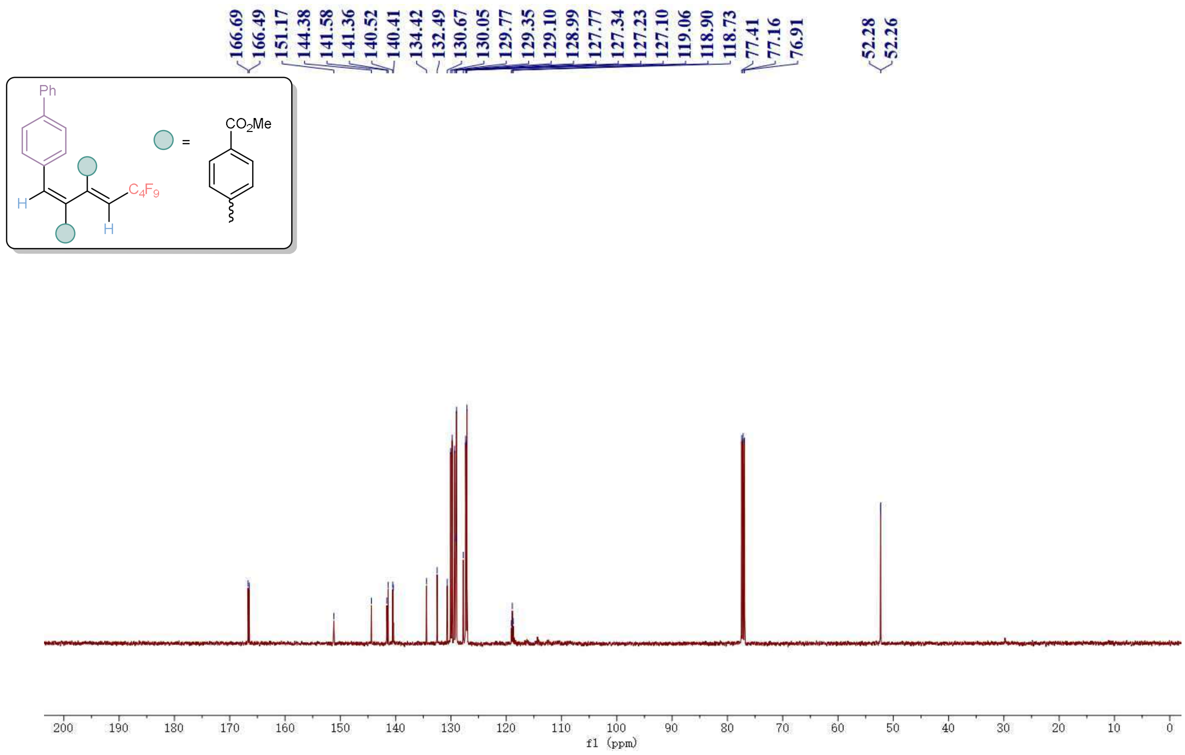

^{1}H NMR spectrum of **39** (500 MHz, Chloroform-*d*)

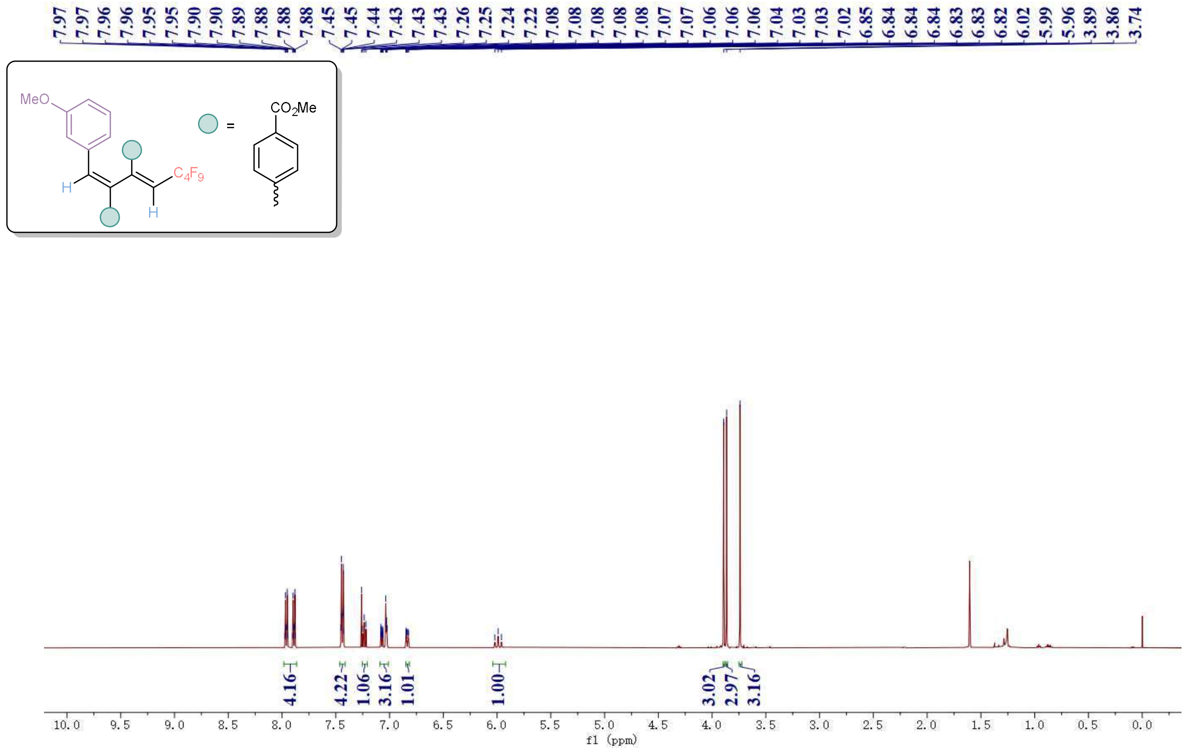

^{19}**F NMR** Spectrum of **39** (471 MHz, Chloroform-*d*)

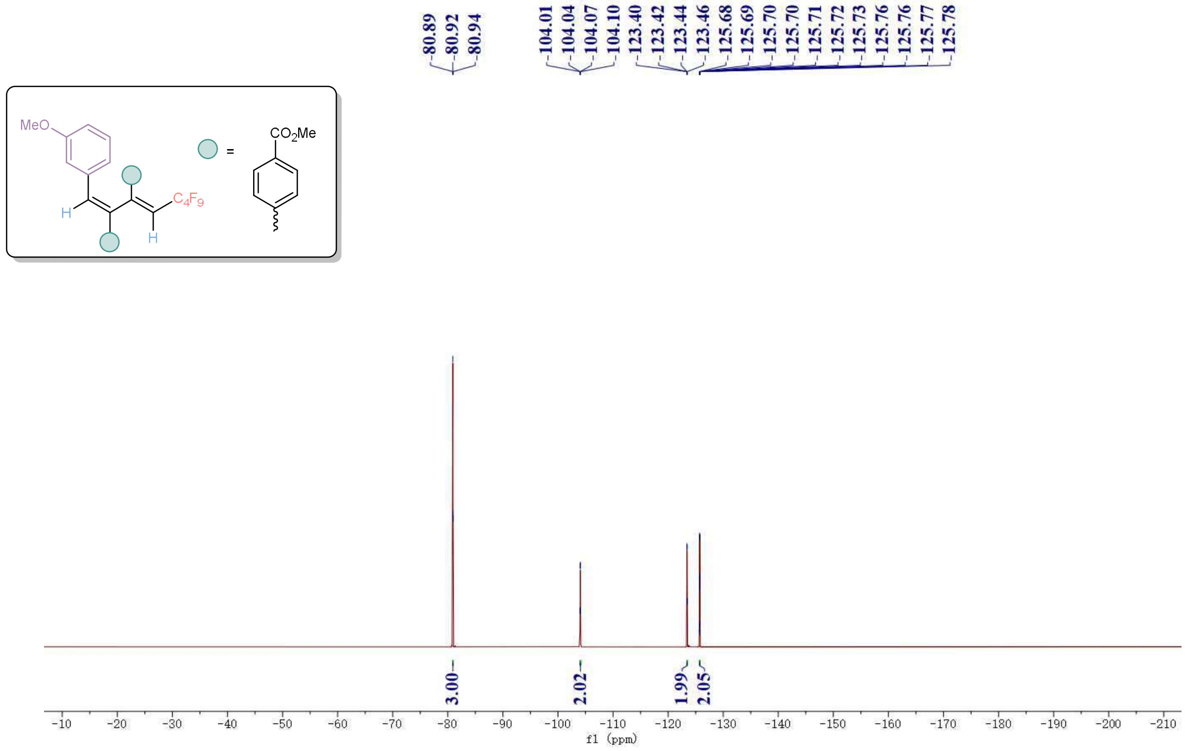

^{13}**C NMR** spectrum of **39** (126 MHz, Chloroform-*d*)

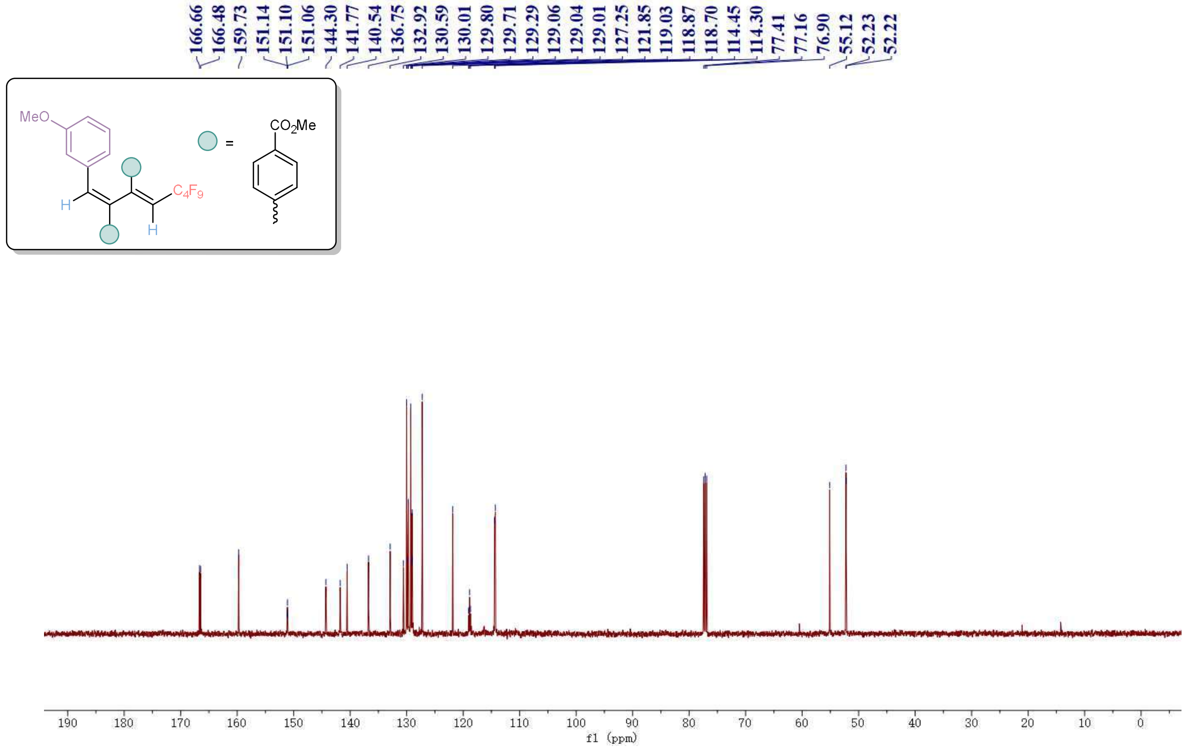

^{1}**H NMR** spectrum of **40** (500 MHz, Chloroform-*d*)

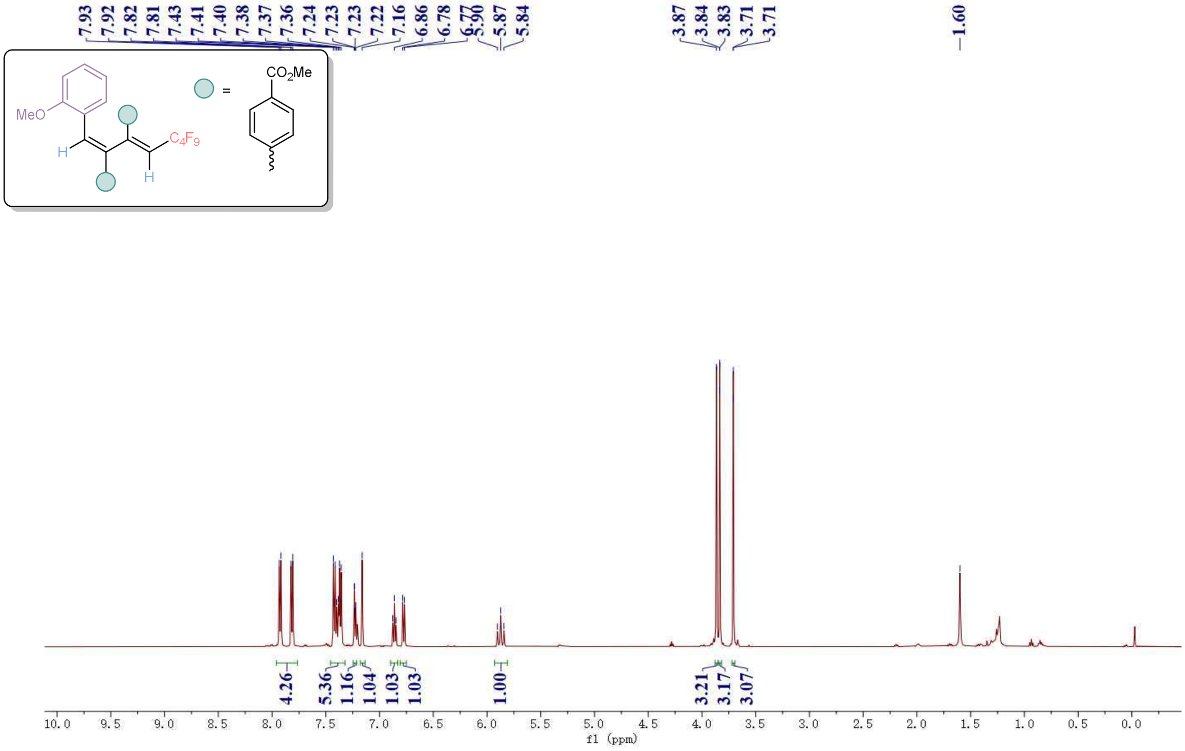

^{19}**F NMR** Spectrum of **40** (471 MHz, Chloroform-*d*)

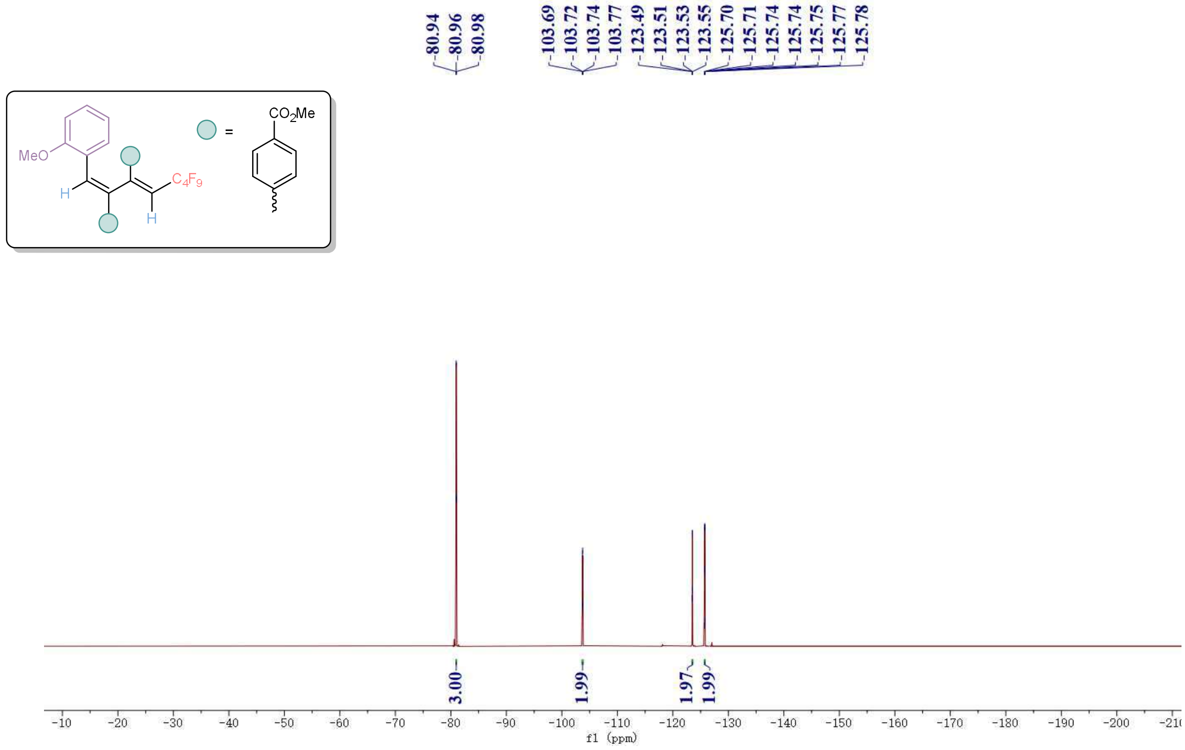

^{13}**C NMR** spectrum of **40** (126 MHz, Chloroform-*d*)

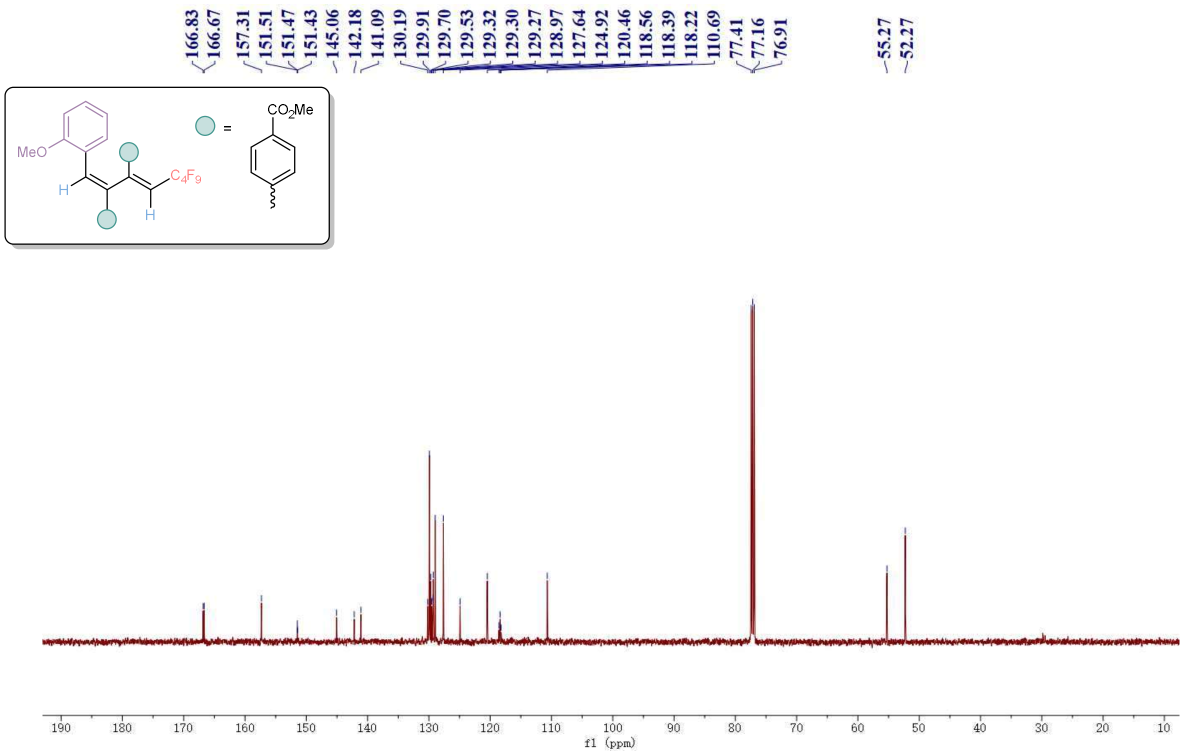

^{1}**H NMR** spectrum of **41** (500 MHz, Chloroform-*d*)

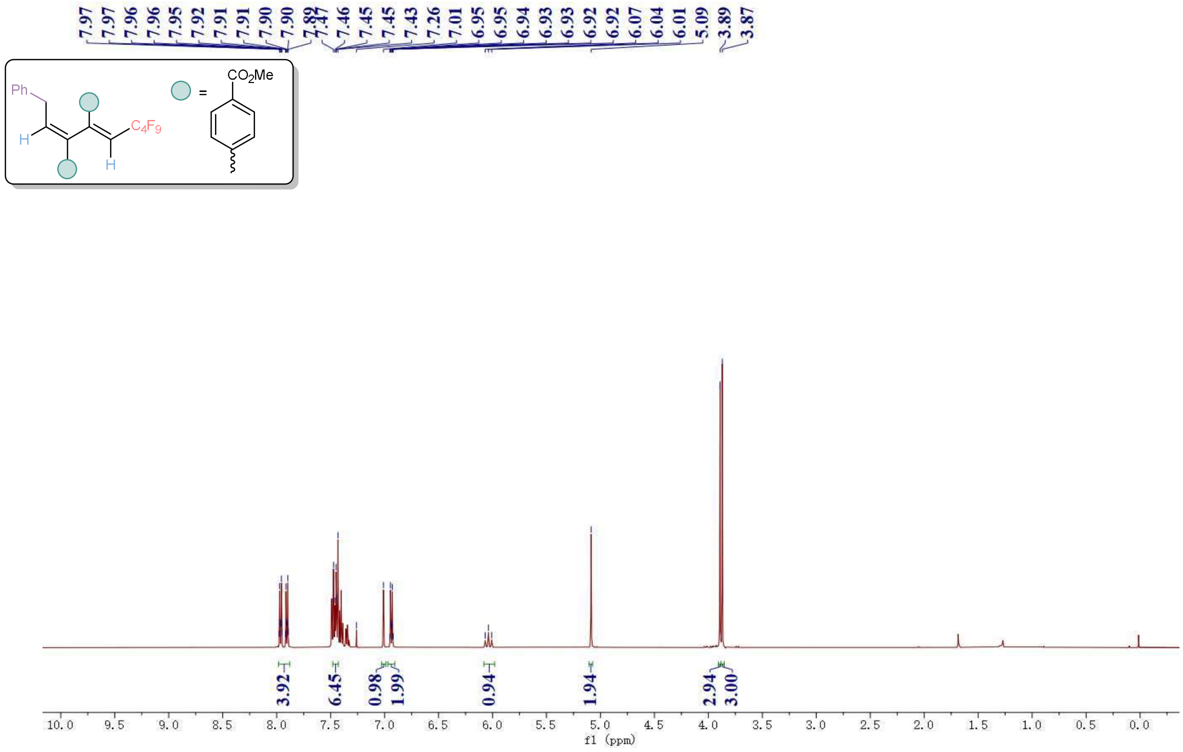

^{19}F NMR Spectrum of **41** (471 MHz, Chloroform-*d*)

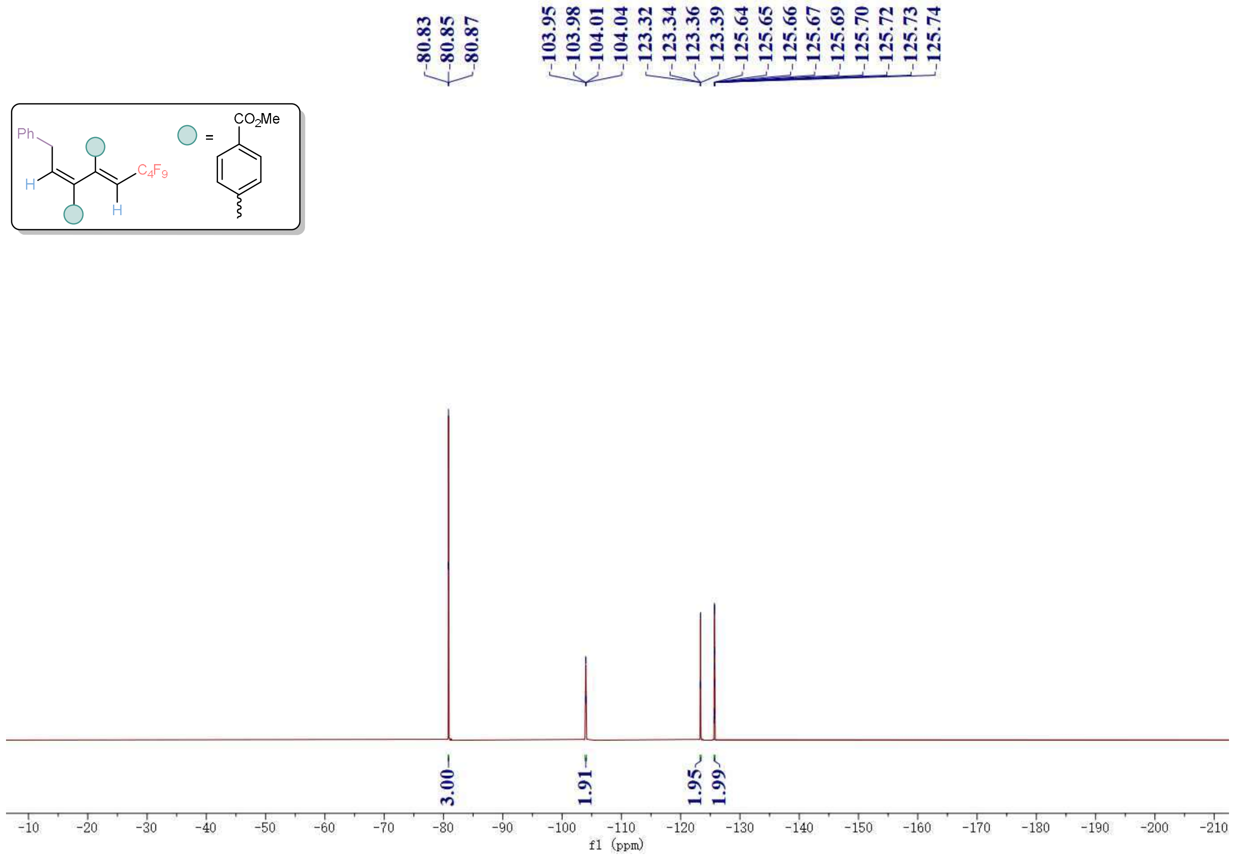

^{13}C NMR spectrum of **41** (126 MHz, Chloroform-*d*)

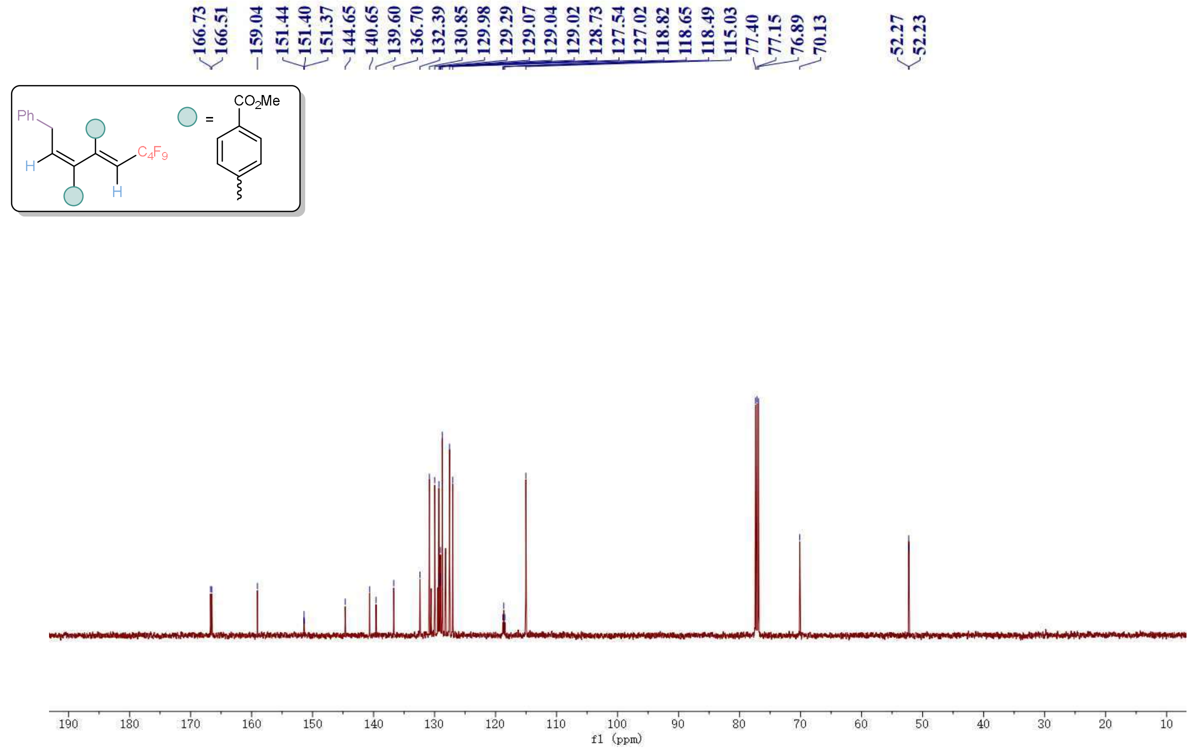

^{1}H NMR spectrum of **42** (500 MHz, Chloroform-*d*)

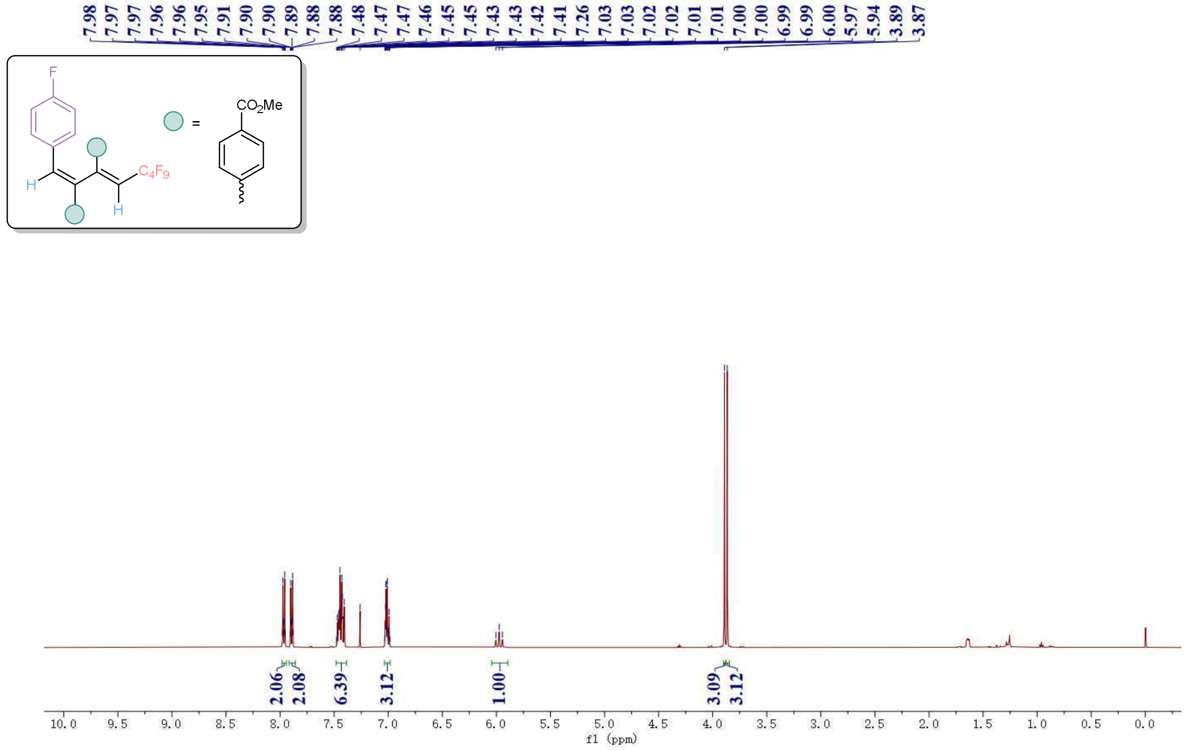

^{19}F NMR Spectrum of **42** (471 MHz, Chloroform-*d*)

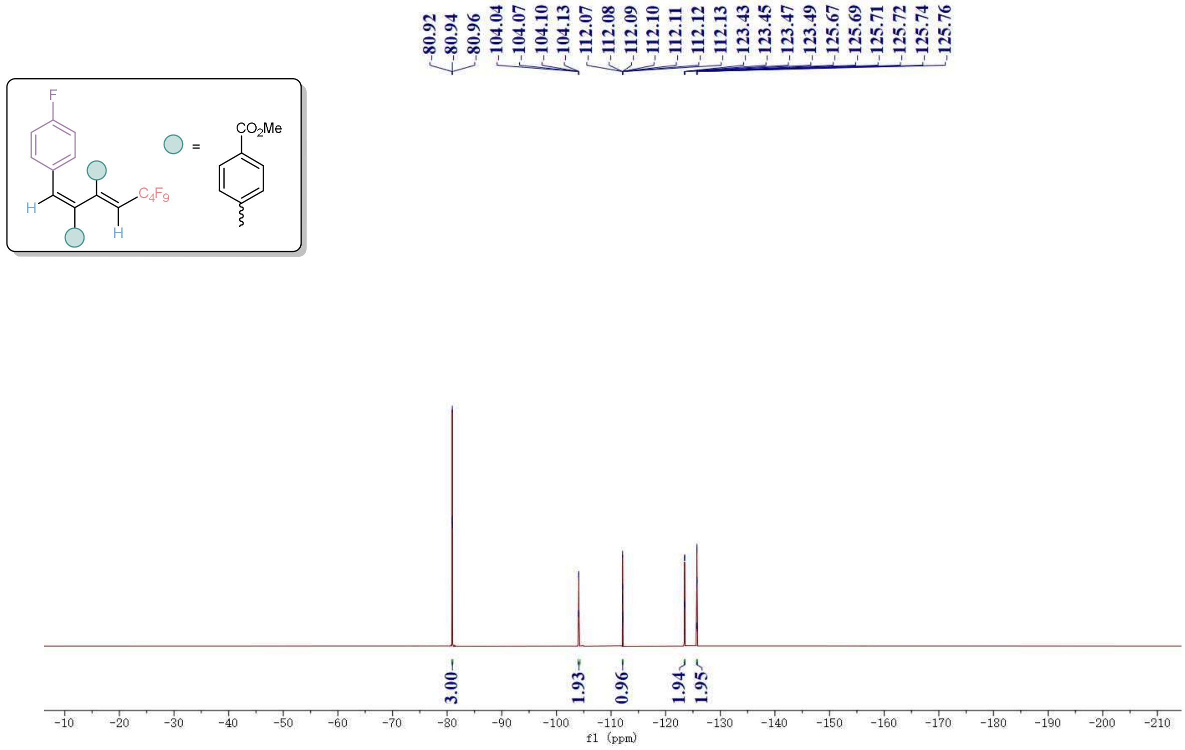

^{13}**C NMR** spectrum of **42** (126 MHz, Chloroform-d)

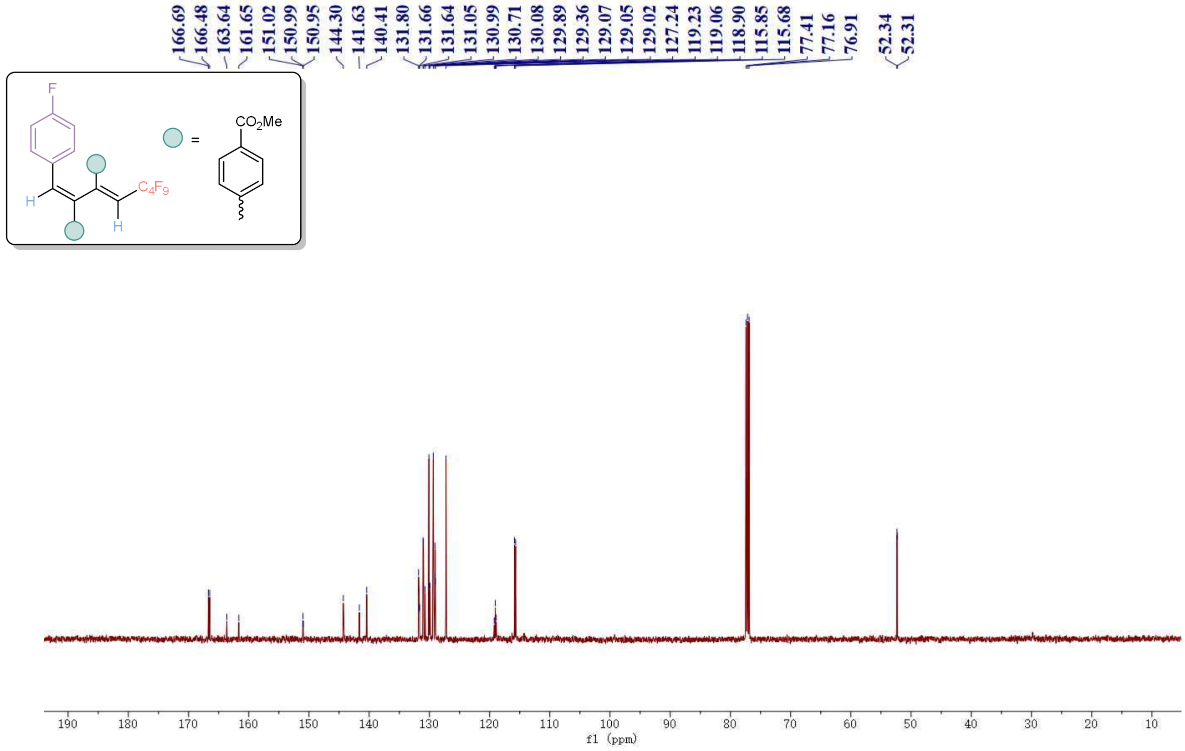

^{1}**H NMR** spectrum of **43** (500 MHz, Chloroform-*d*)

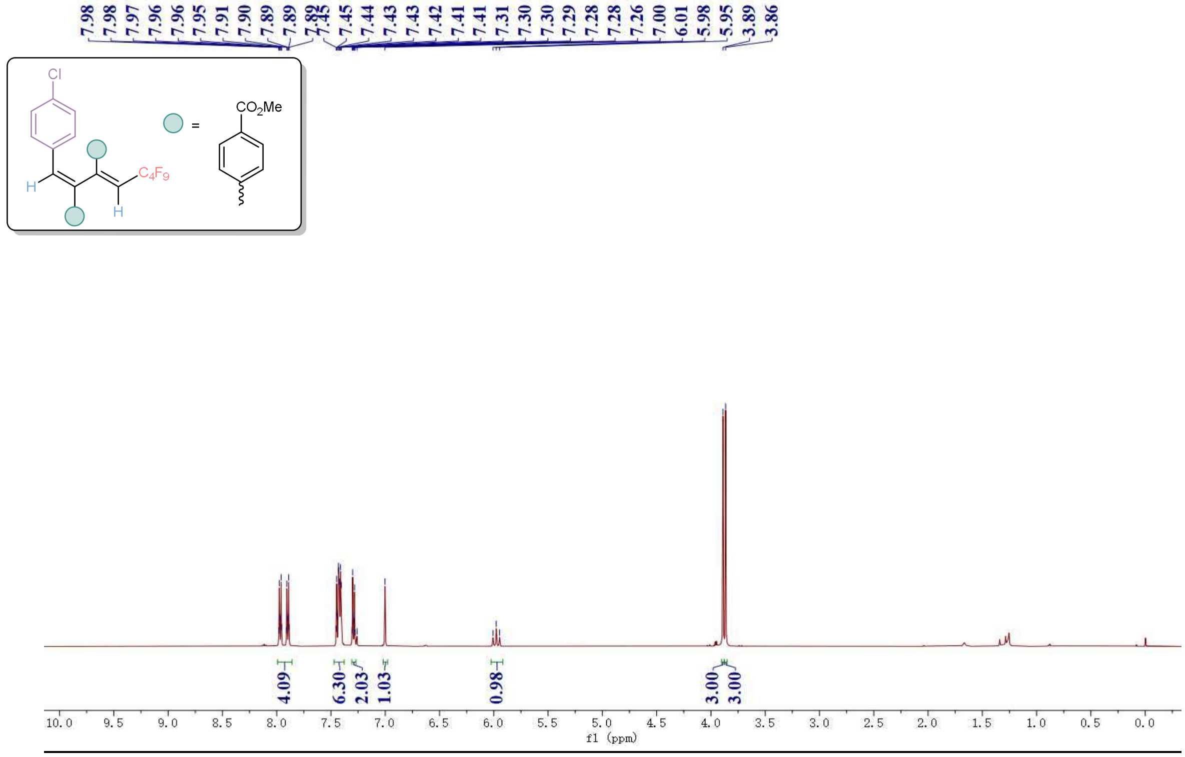

^{19}**F NMR** Spectrum of **43** (471 MHz, Chloroform-*d*)

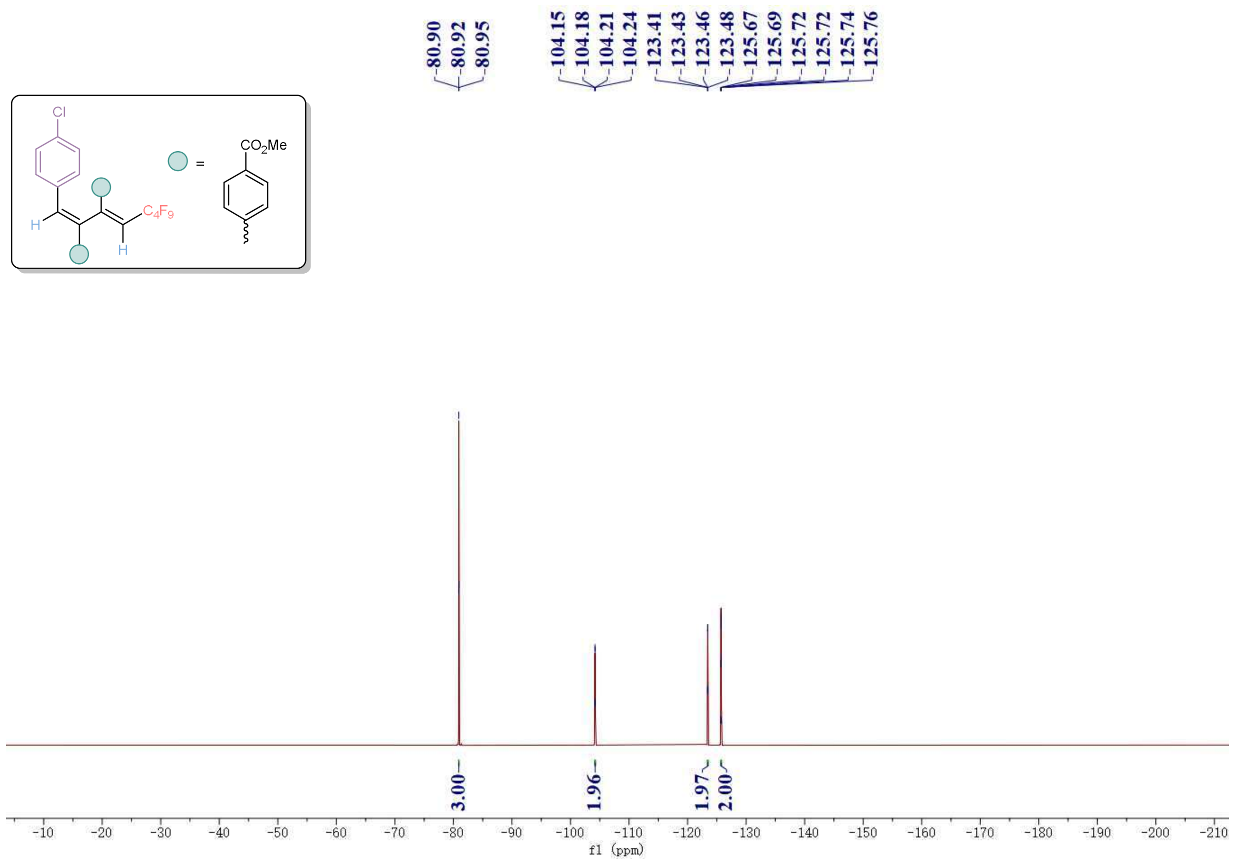

^{13}**C NMR** spectrum of **43** (126 MHz, Chloroform-*d*)

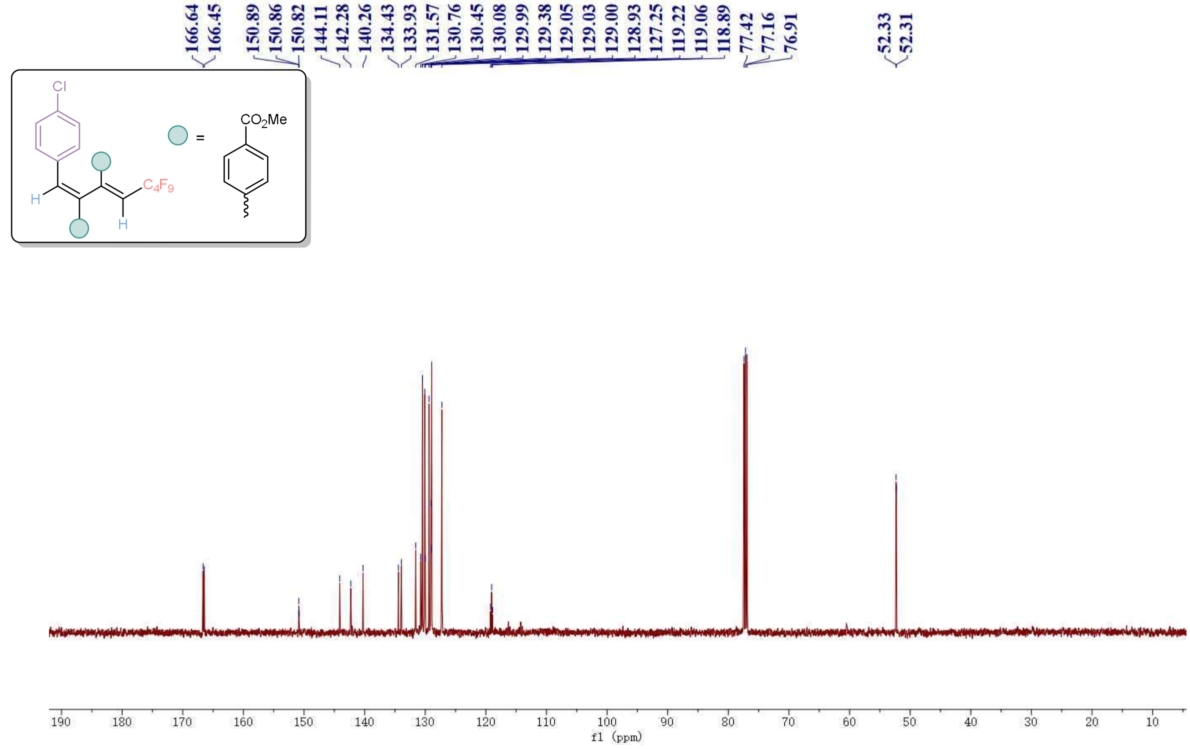

^{1}H NMR spectrum of **44** (500 MHz, Chloroform-*d*)

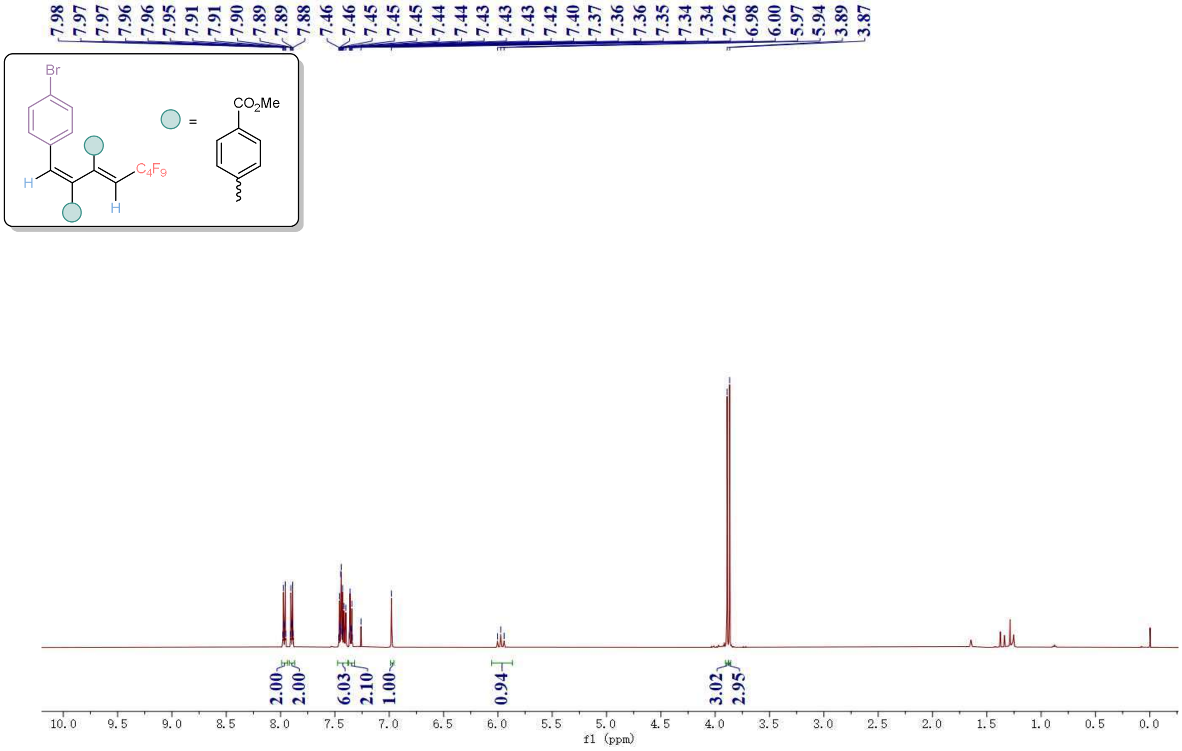

^{19}F NMR Spectrum of **44** (471 MHz, Chloroform-*d*)

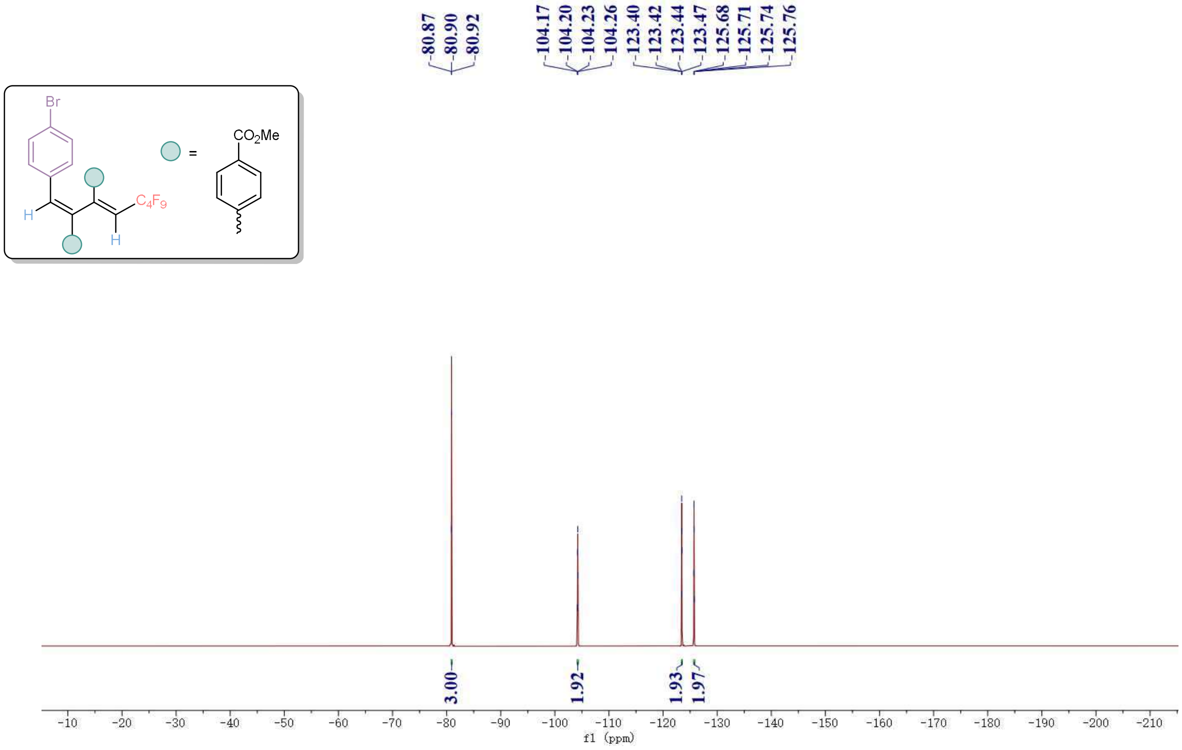

^{13}**C NMR** spectrum of **44** (126 MHz, Chloroform-*d*)

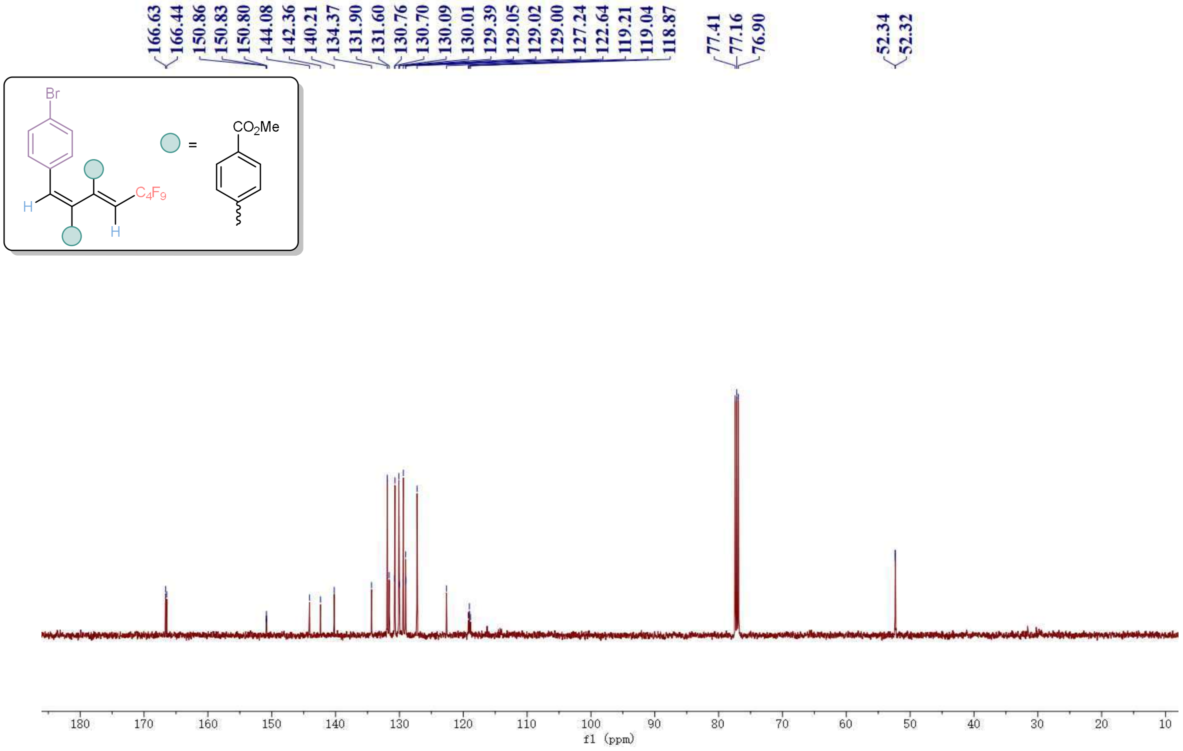

^{1}**H NMR** spectrum of **45** (500 MHz, Chloroform-*d*)

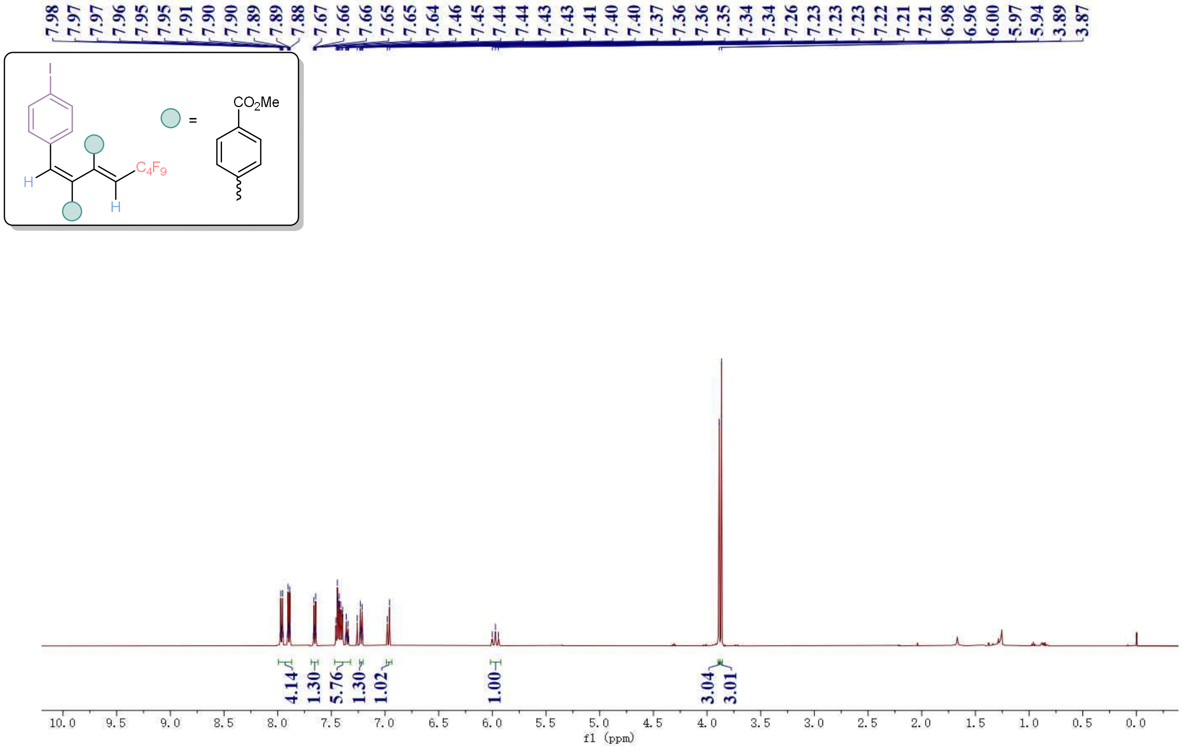

^{19}F NMR Spectrum of **45** (471 MHz, Chloroform-*d*)

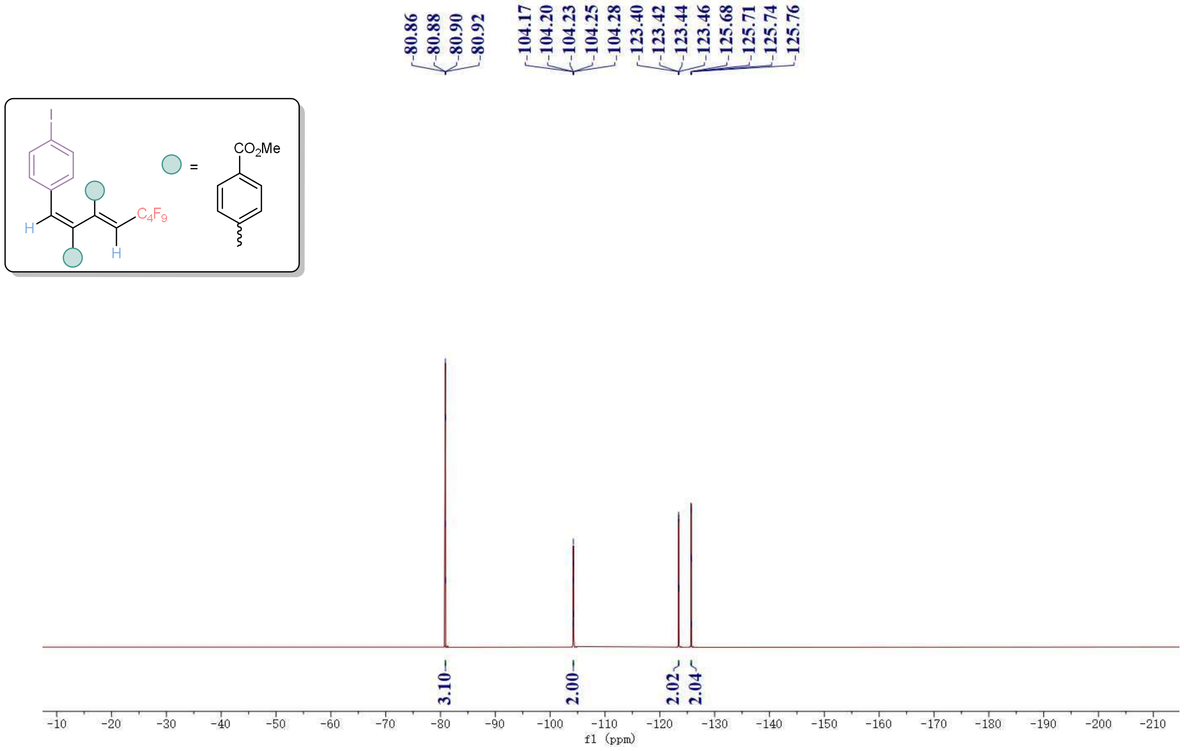

^{13}C NMR spectrum of **45** (126 MHz, Chloroform-*d*)

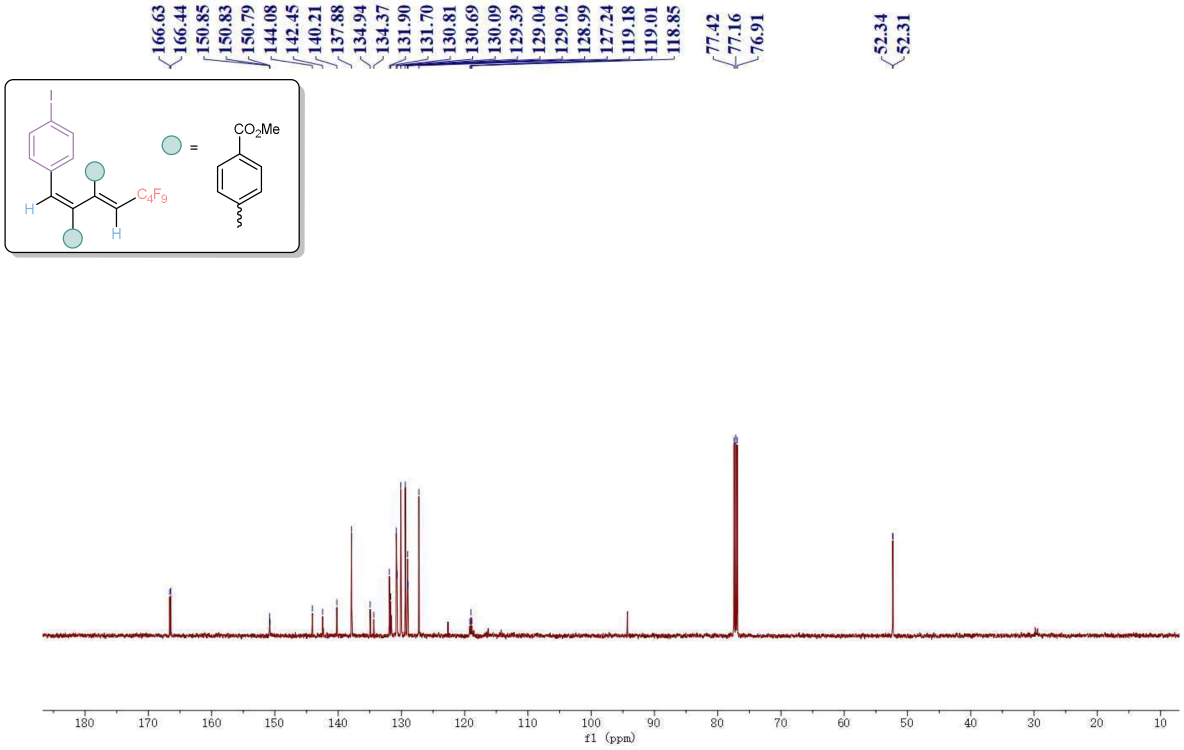

^{1}**H NMR** spectrum of **46** (500 MHz, Chloroform-*d*)

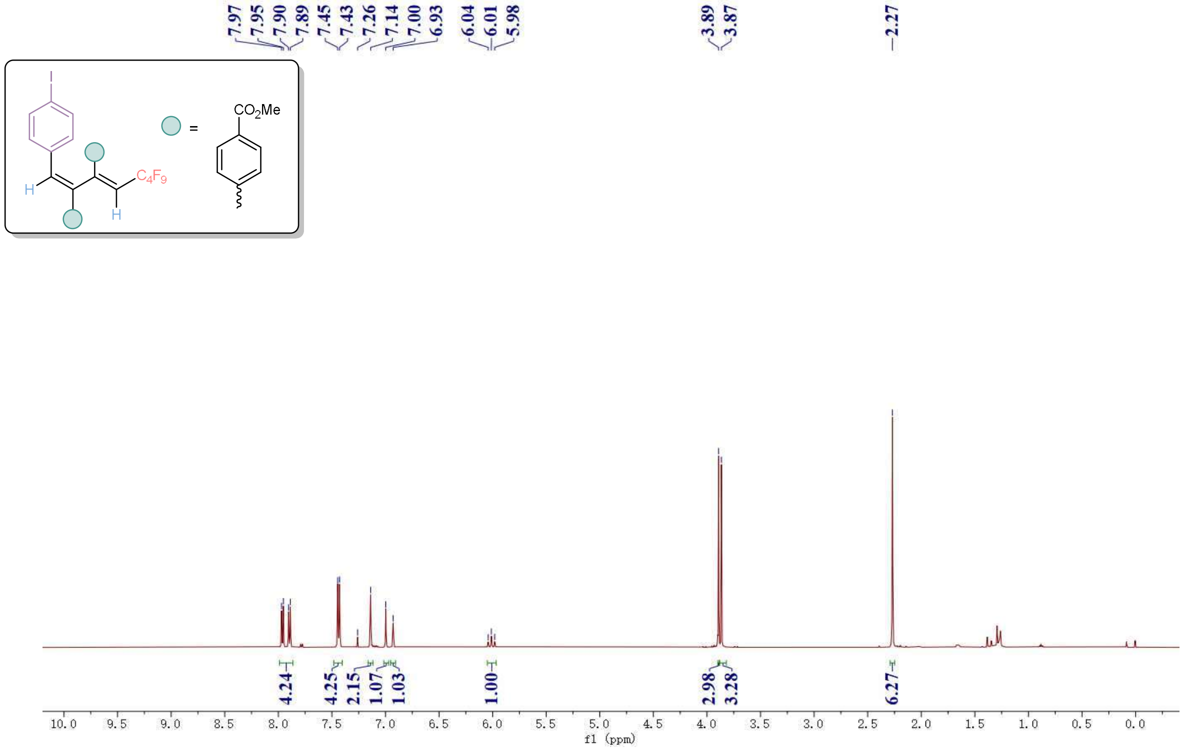

^{19}**F NMR** Spectrum of **46** (471 MHz, Chloroform-*d*)

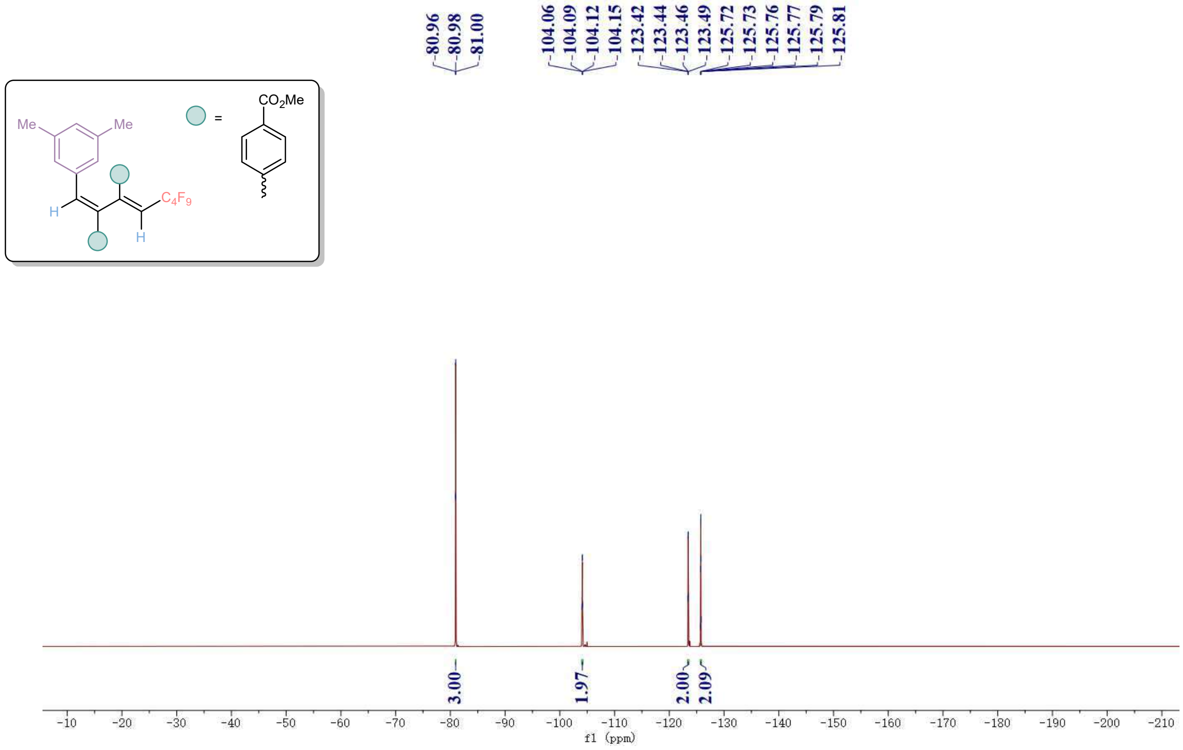

^{13}**C NMR** spectrum of **46** (126 MHz, Chloroform-*d*)

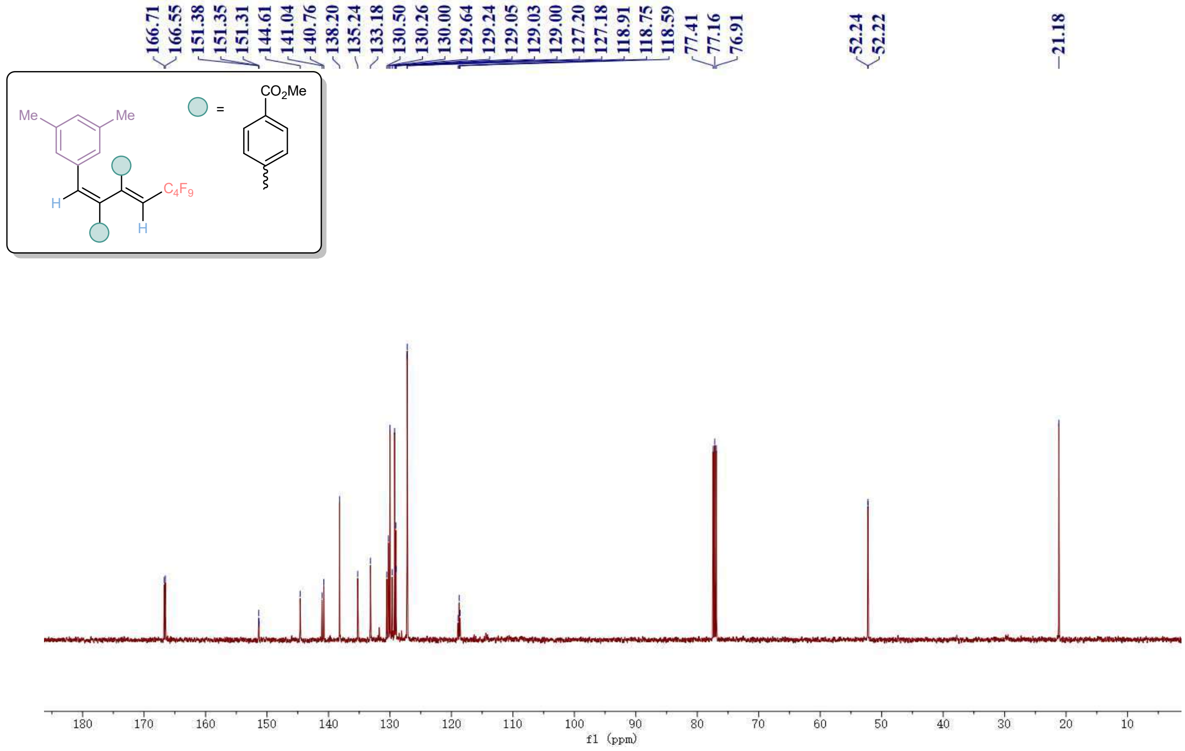

^{1}**H NMR** spectrum of **47** (500 MHz, Chloroform-*d*)

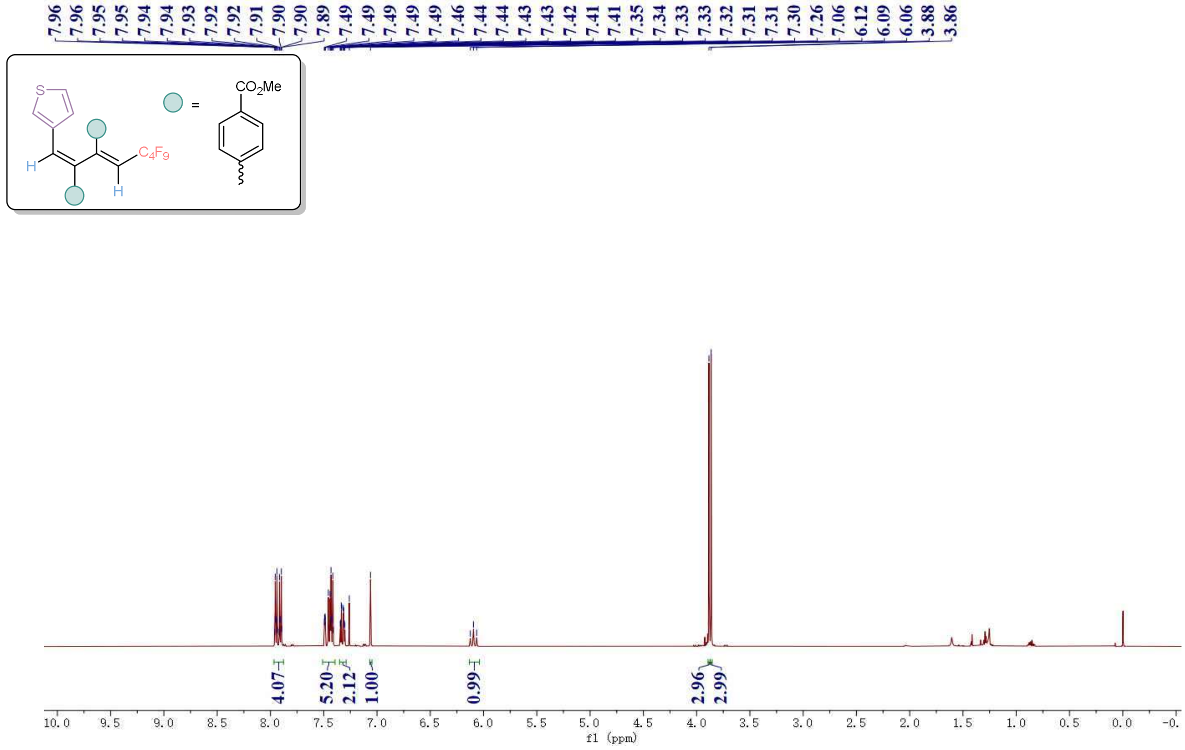

^{19}F NMR Spectrum of **47** (471 MHz, Chloroform-*d*)

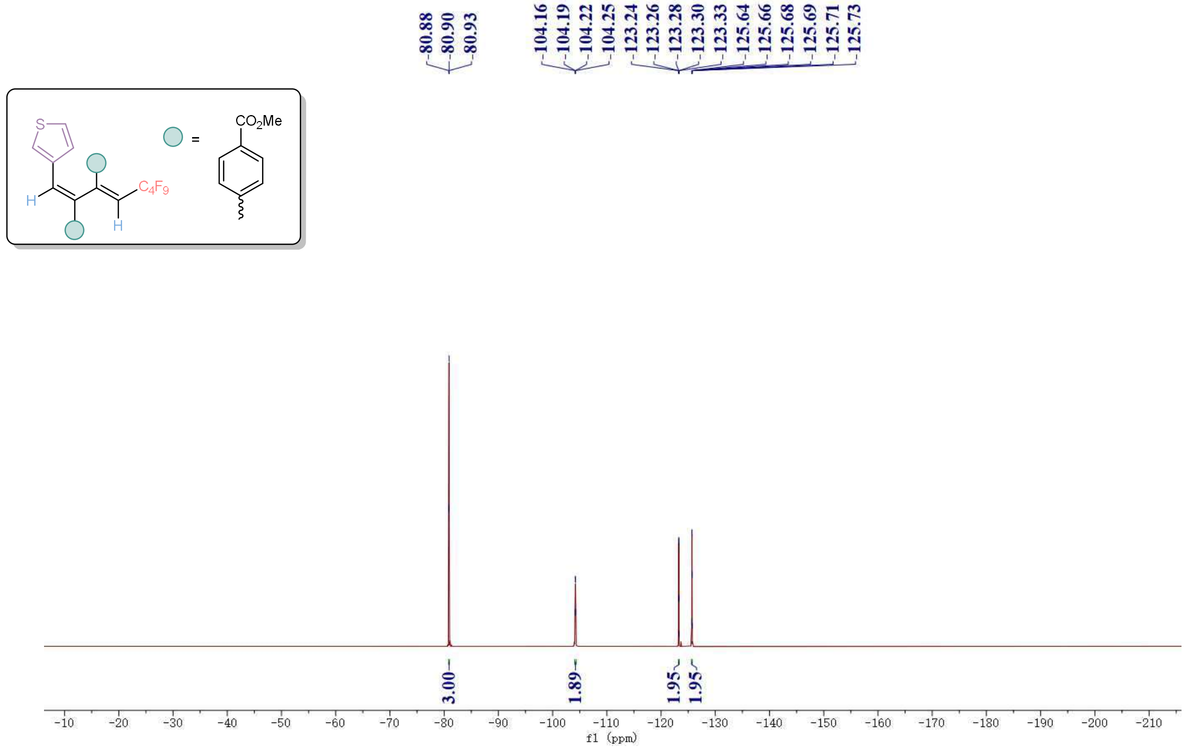

^{13}C NMR spectrum of **47** (126 MHz, Chloroform-*d*)

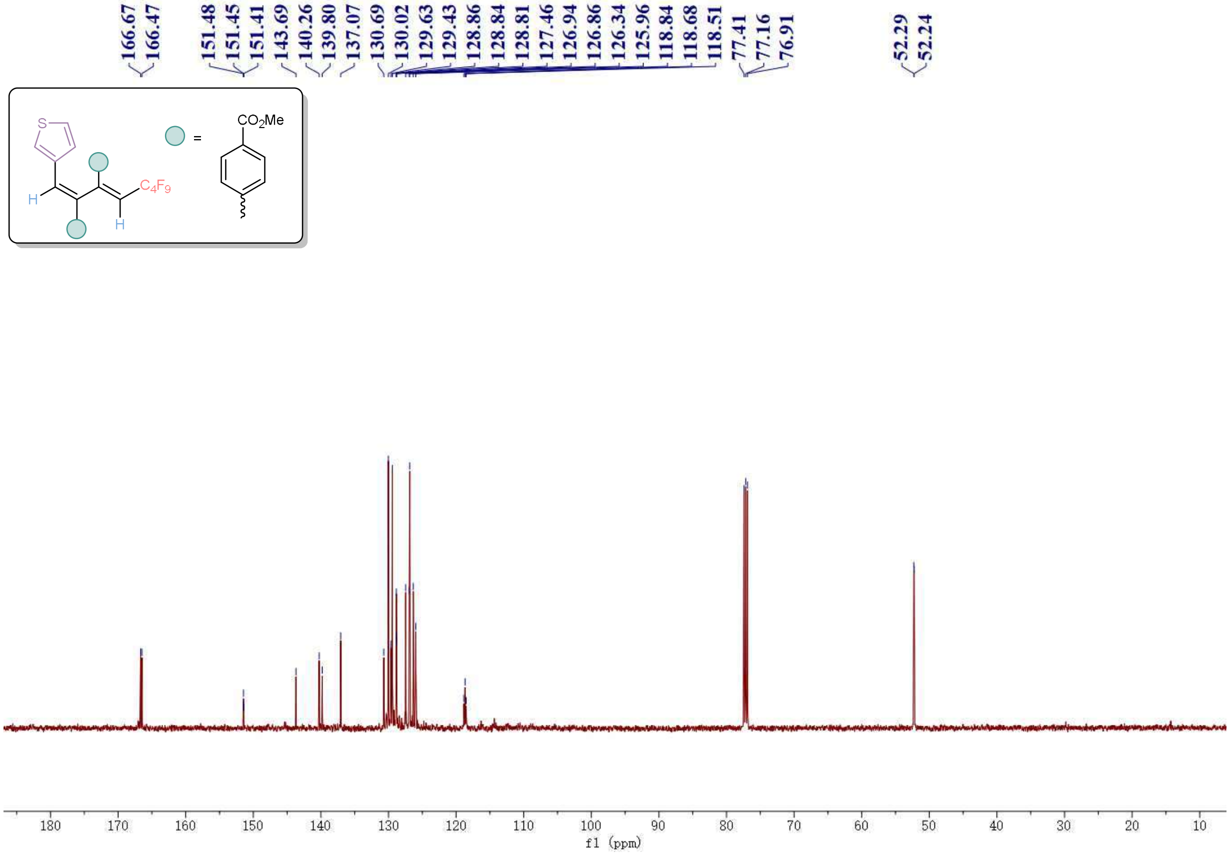

^{1}H NMR spectrum of **48** (500 MHz, Chloroform-*d*)

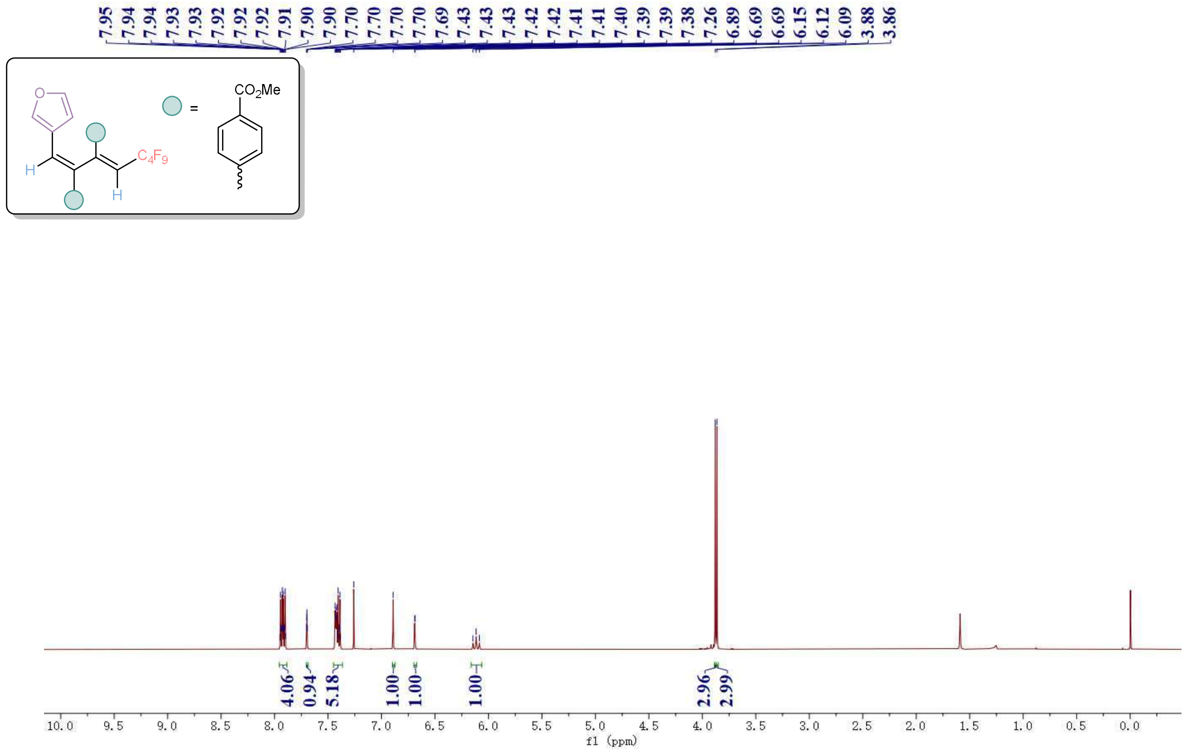

^{19}F NMR Spectrum of **48** (471 MHz, Chloroform-*d*)

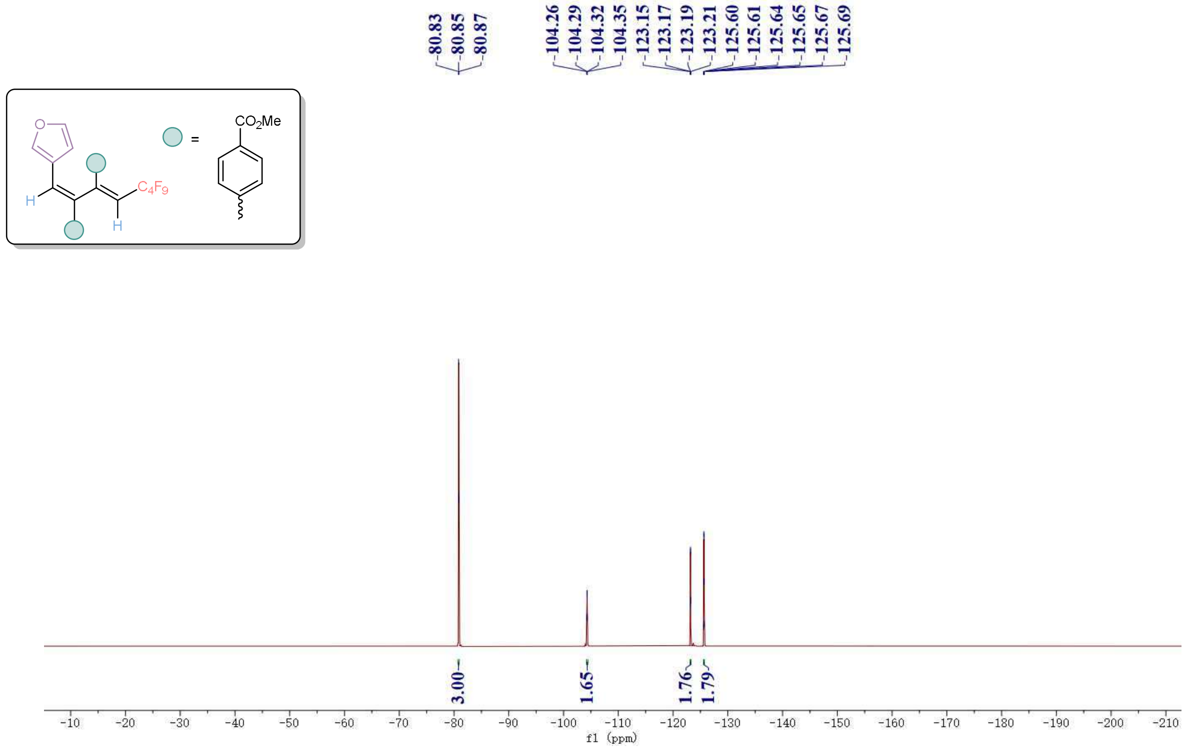

^{13}**C NMR** spectrum of **48** (126 MHz, Chloroform-*d*)

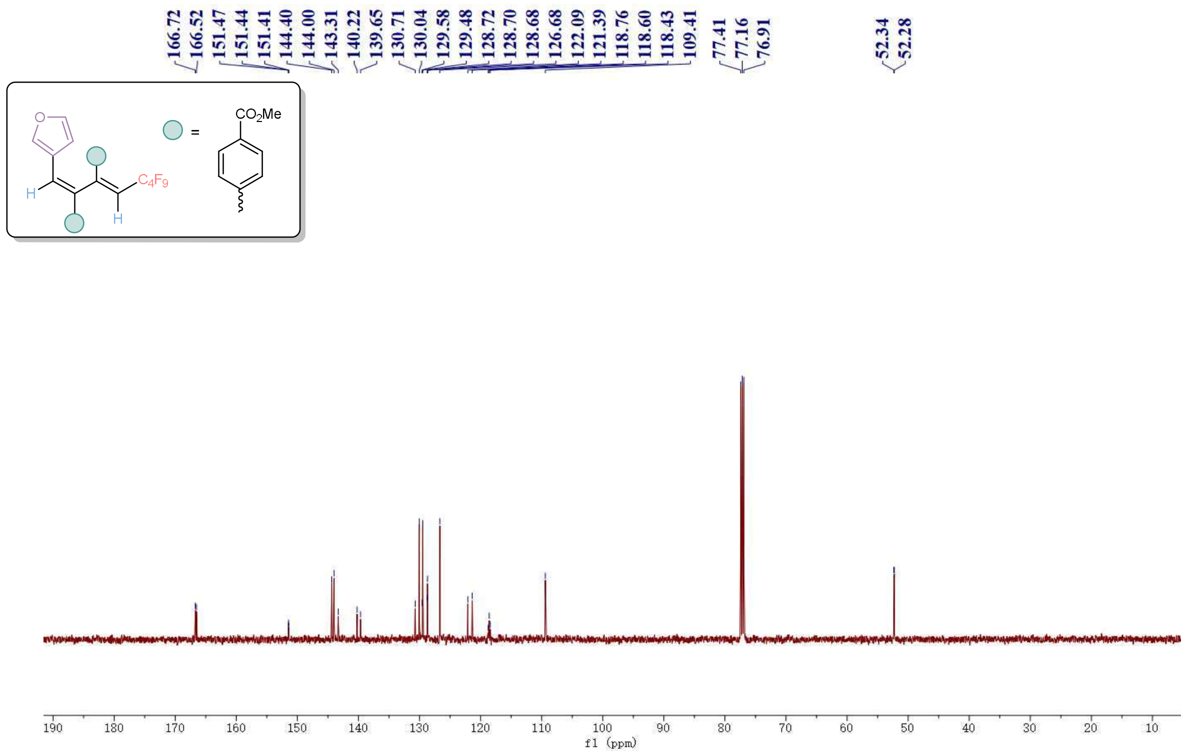

^{1}**H NMR** spectrum of **49** (500 MHz, Chloroform-*d*)

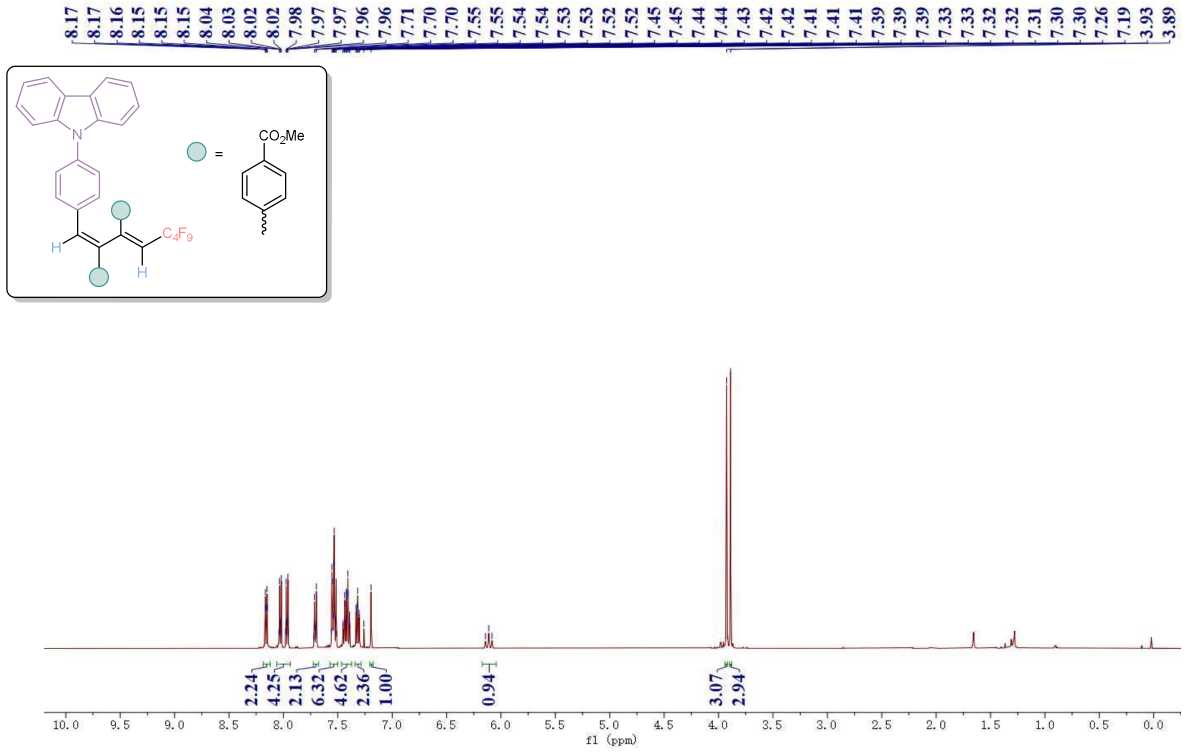

^{19}F NMR Spectrum of **49** (471 MHz, Chloroform-*d*)

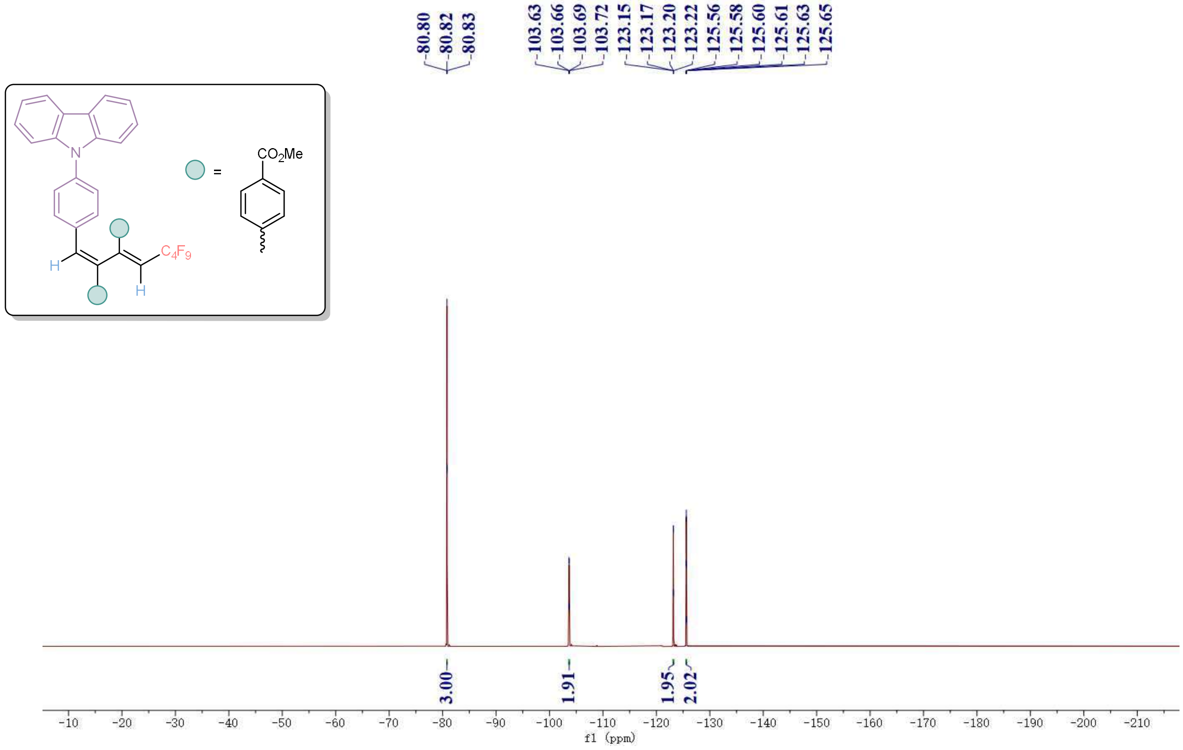

^{13}C NMR spectrum of **49** (126 MHz, Chloroform-d)

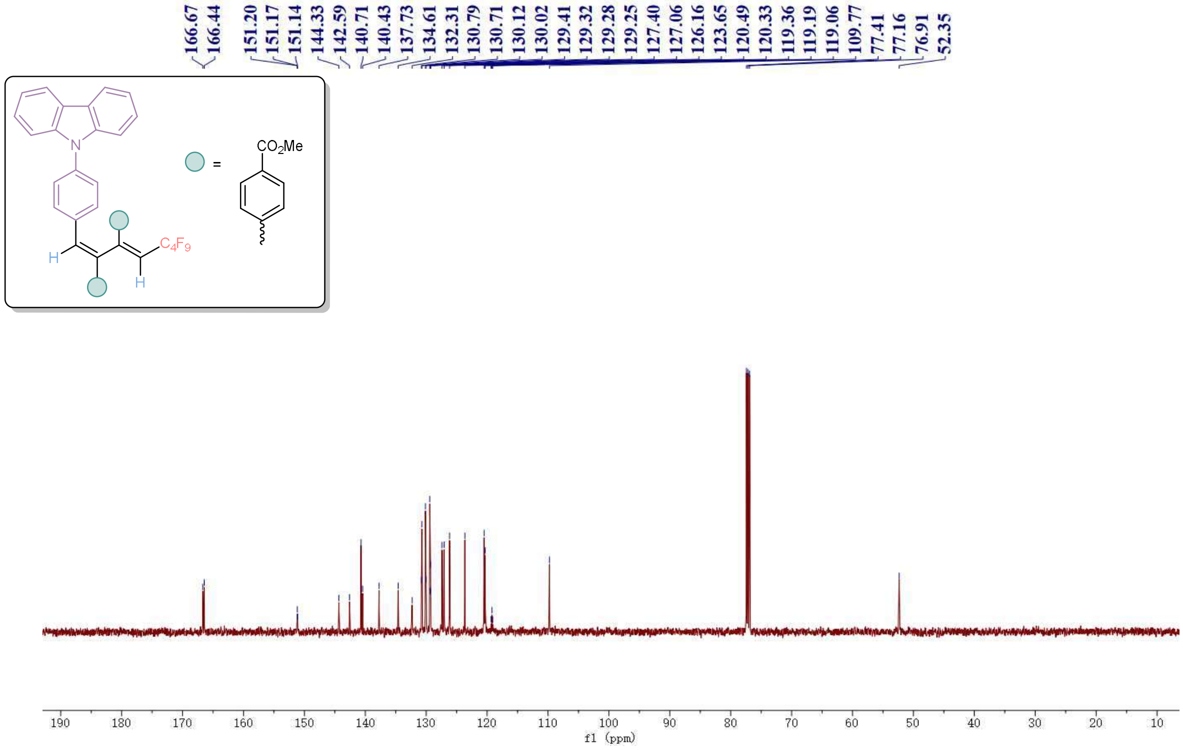

^{1}**H NMR** spectrum of **50** (500 MHz, Chloroform-*d*)

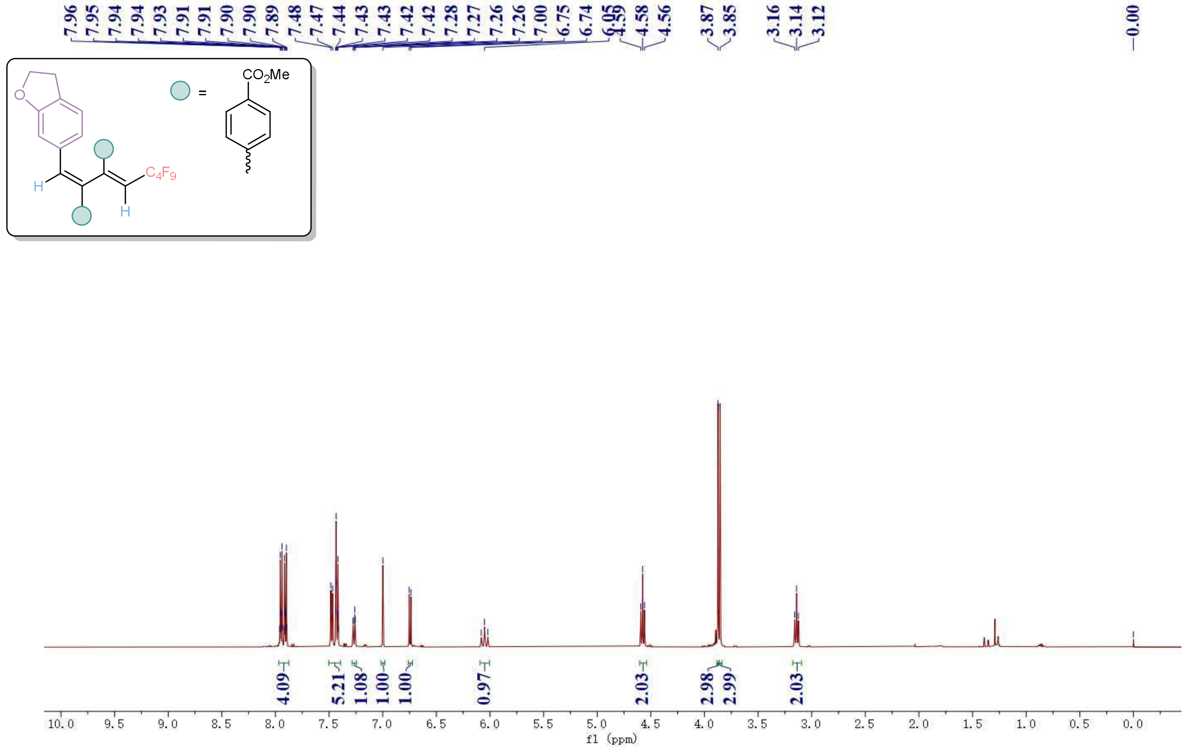

^{19}**F NMR** Spectrum of **50** (471 MHz, Chloroform-*d*)

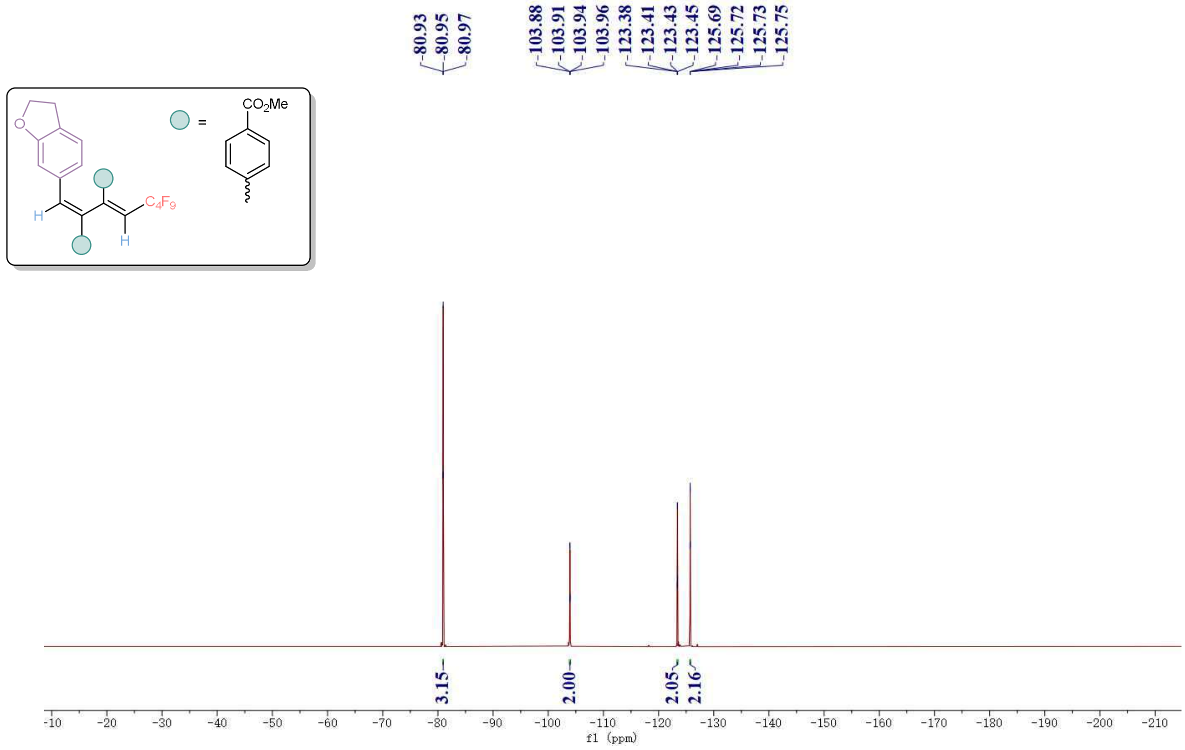

^{13}C NMR spectrum of **50** (126 MHz, Chloroform-*d*)

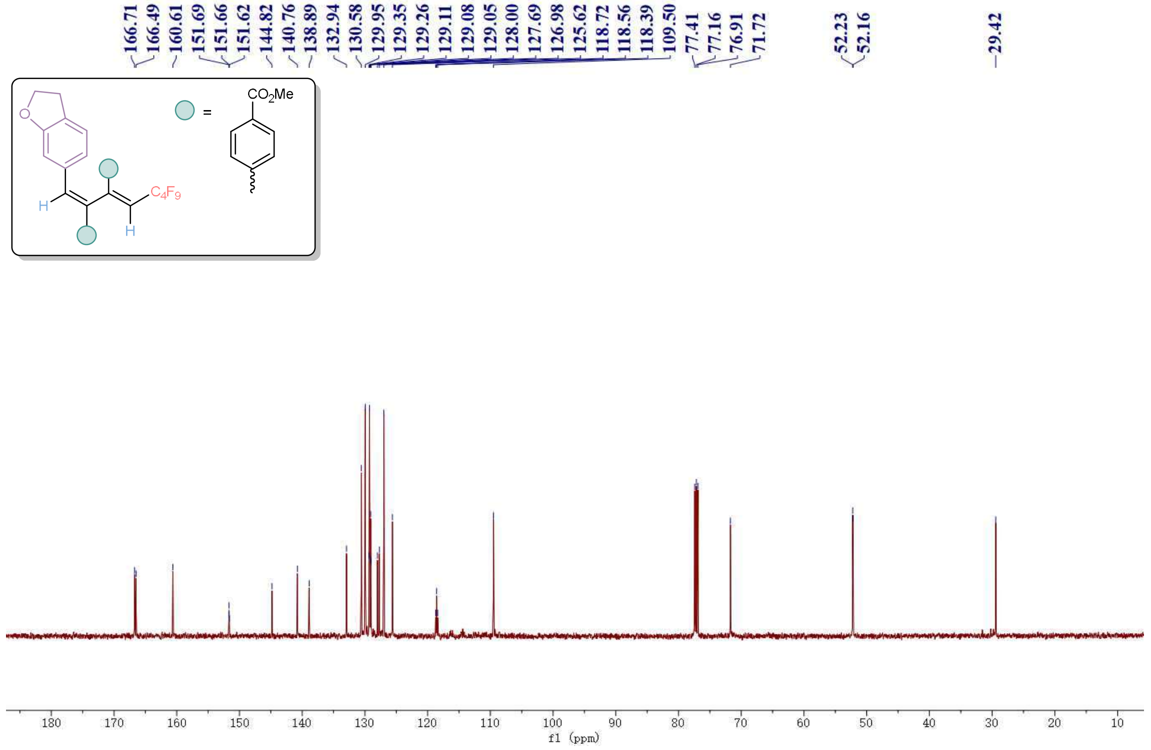

^{1}H NMR spectrum of **51** (500 MHz, Chloroform-*d*)

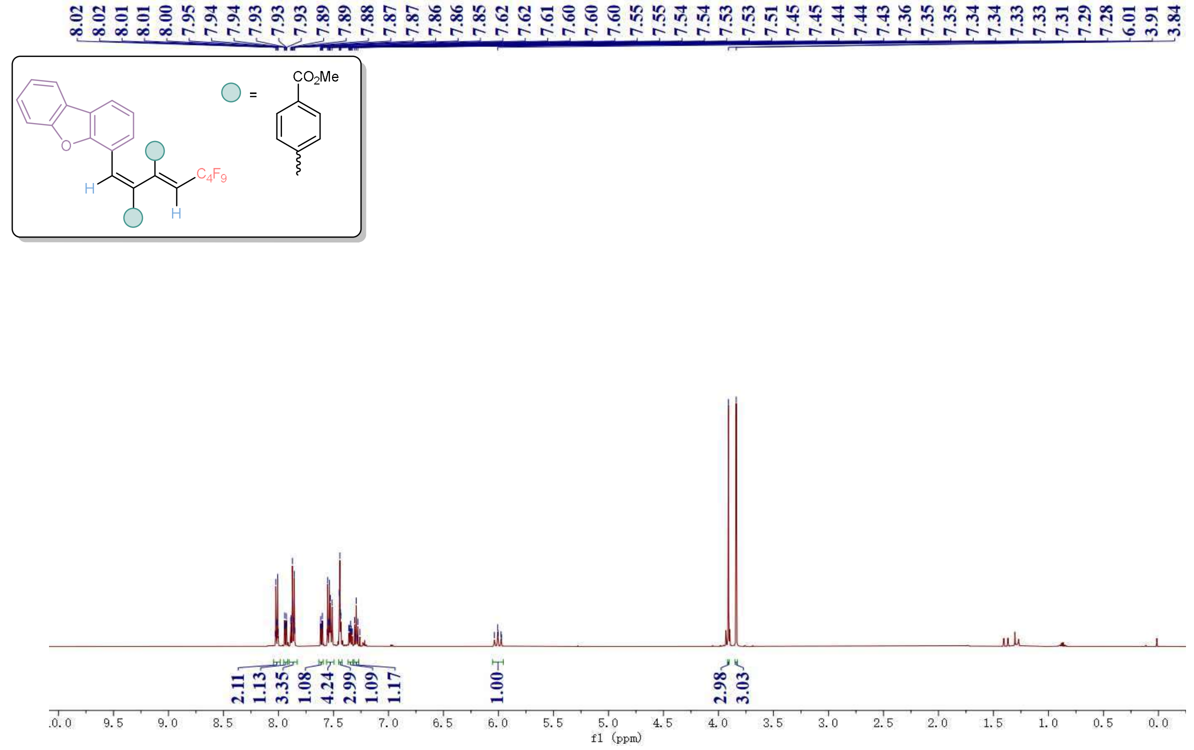

^{19}F NMR Spectrum of **51** (471 MHz, Chloroform-*d*)

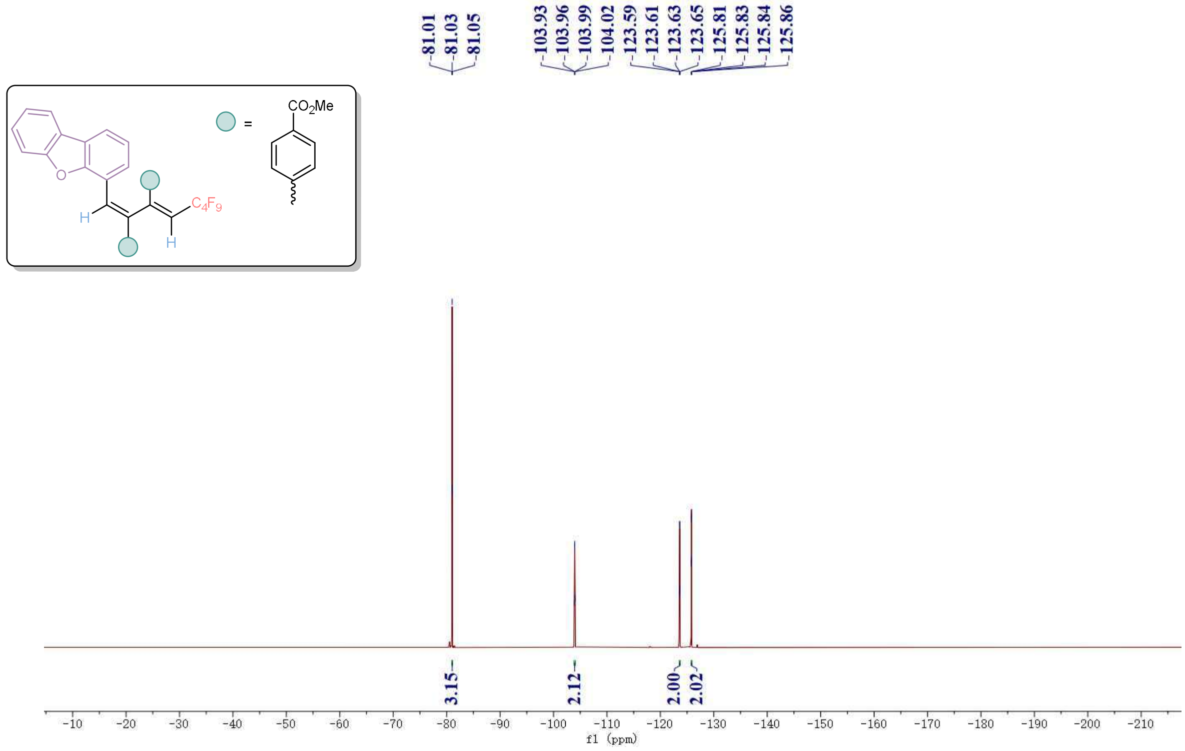

^{13}C NMR spectrum of **51** (126 MHz, Chloroform-*d*)

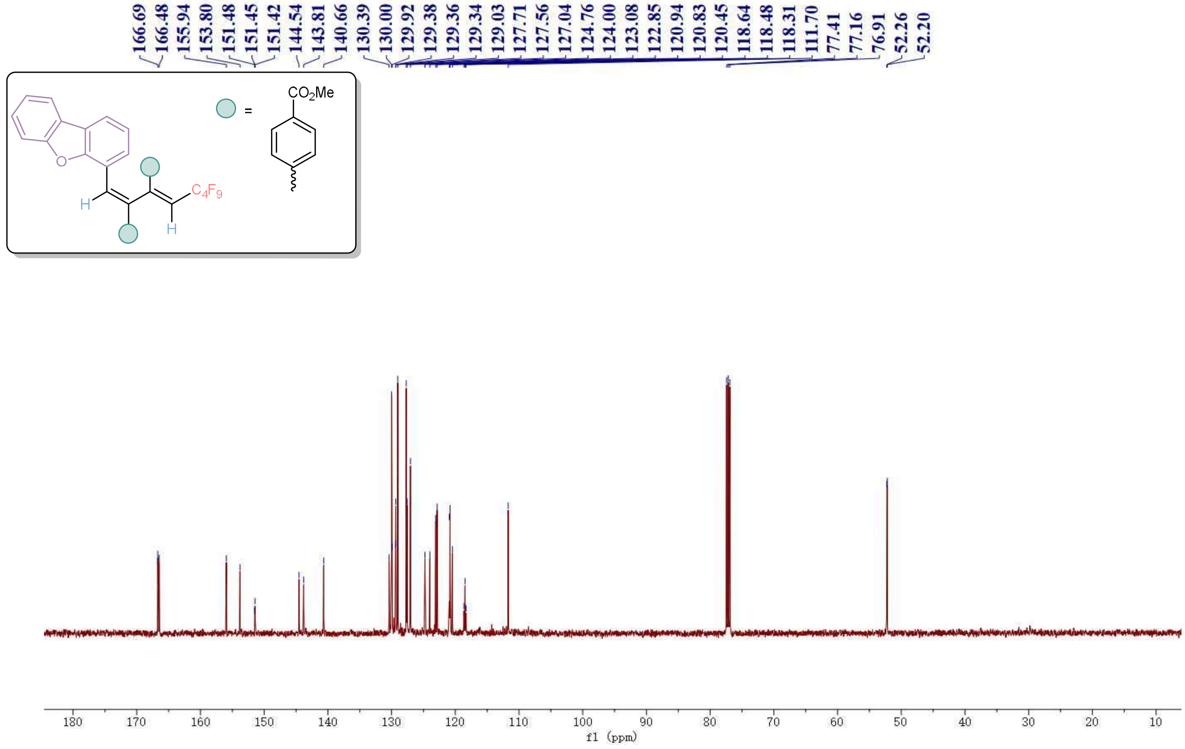

^{1}**H NMR** spectrum of **52** (500 MHz, Chloroform-*d*)

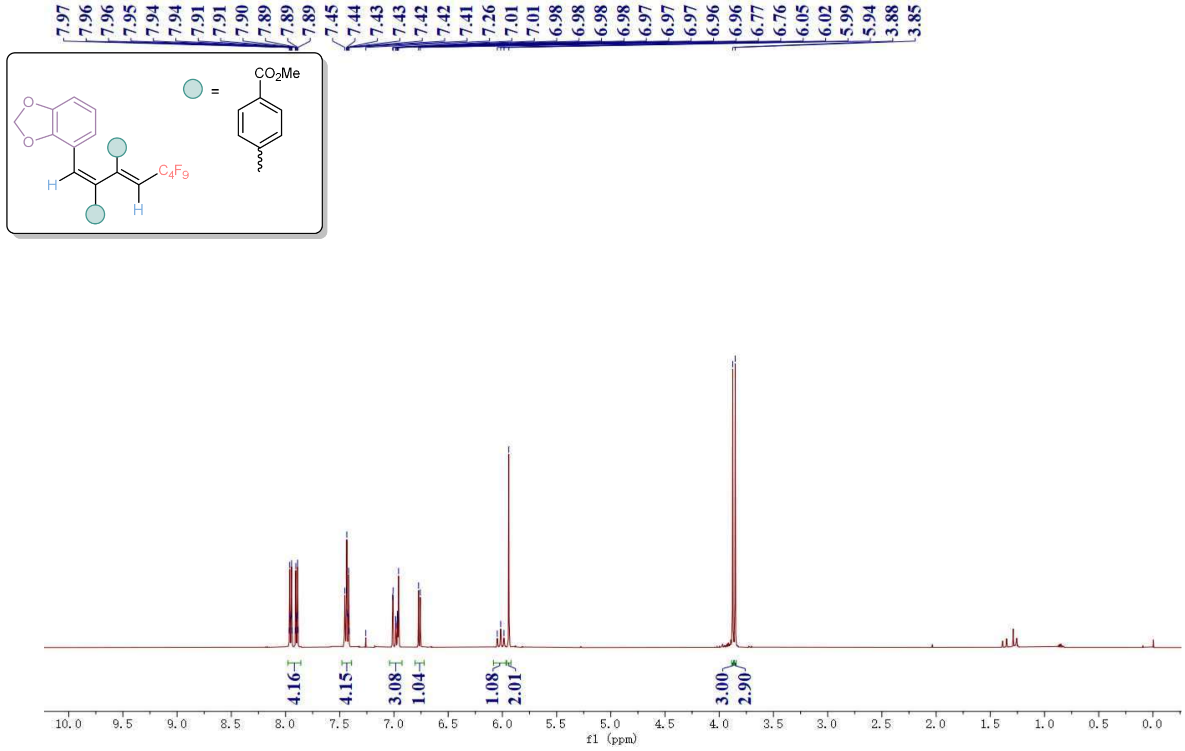

^{19}**F NMR** Spectrum of **52** (471 MHz, Chloroform-*d*)

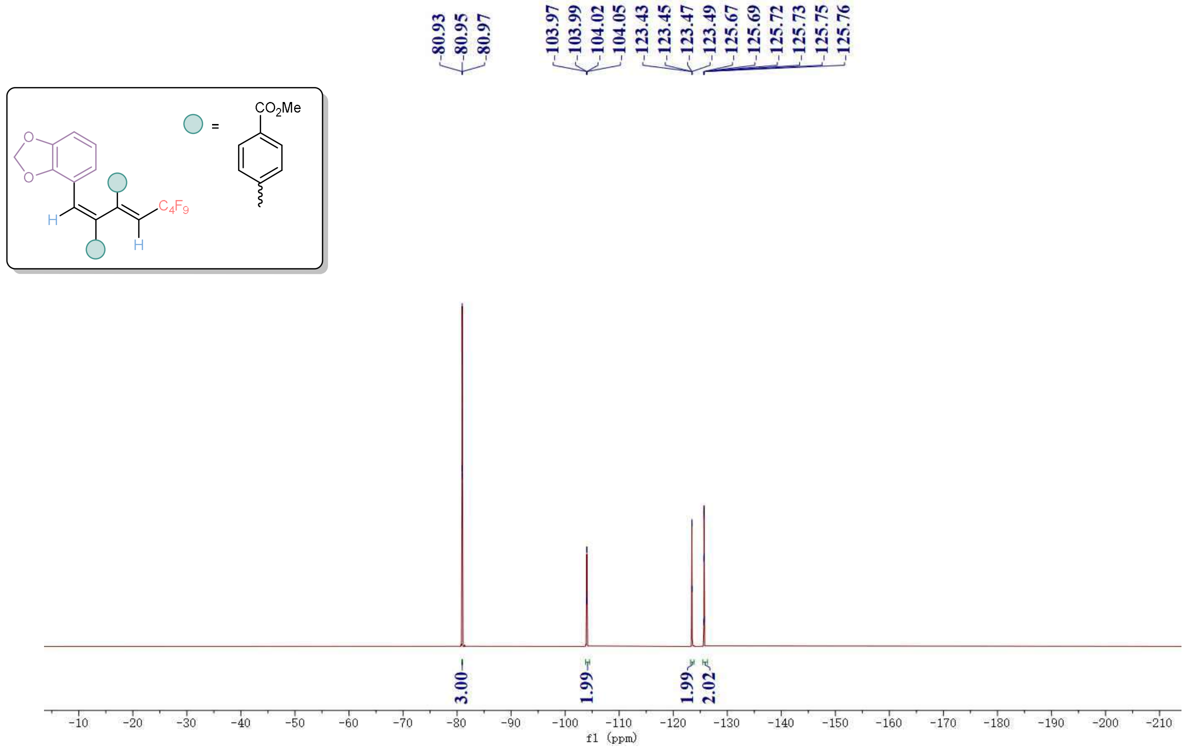

^{13}**C NMR** spectrum of **52** (126 MHz, Chloroform-*d*)

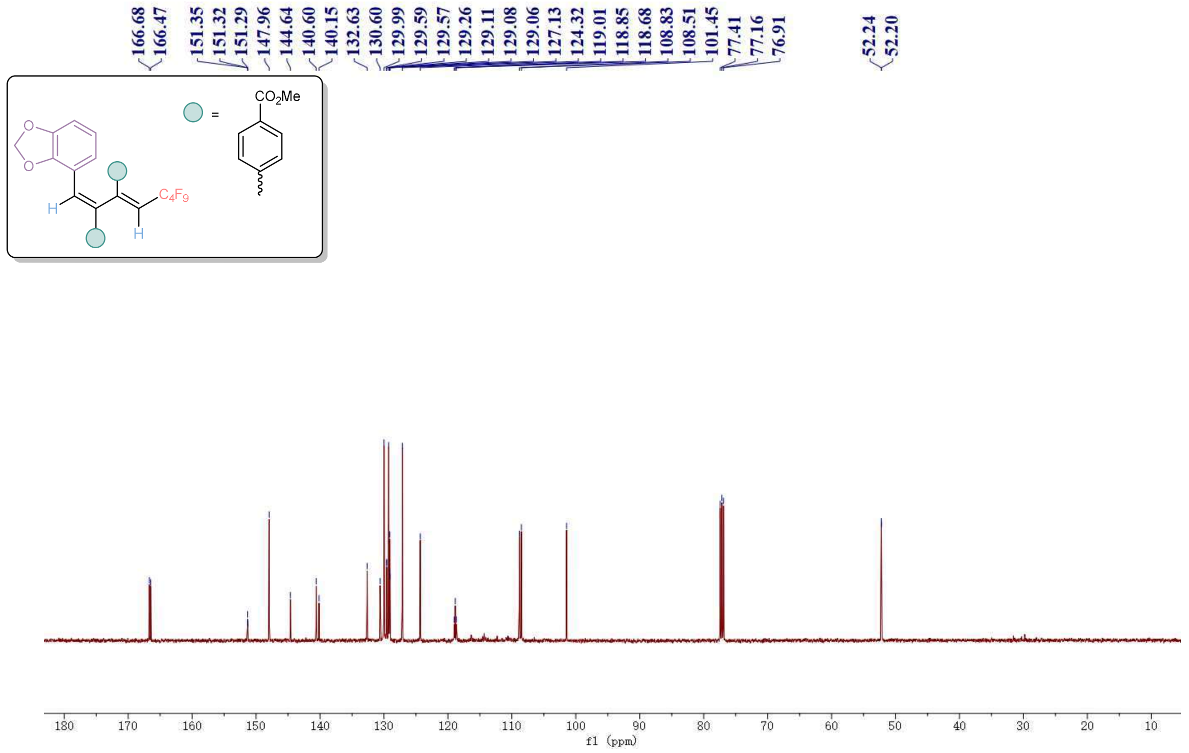

^{1}**H NMR** spectrum of **53** (500 MHz, Chloroform-*d*)

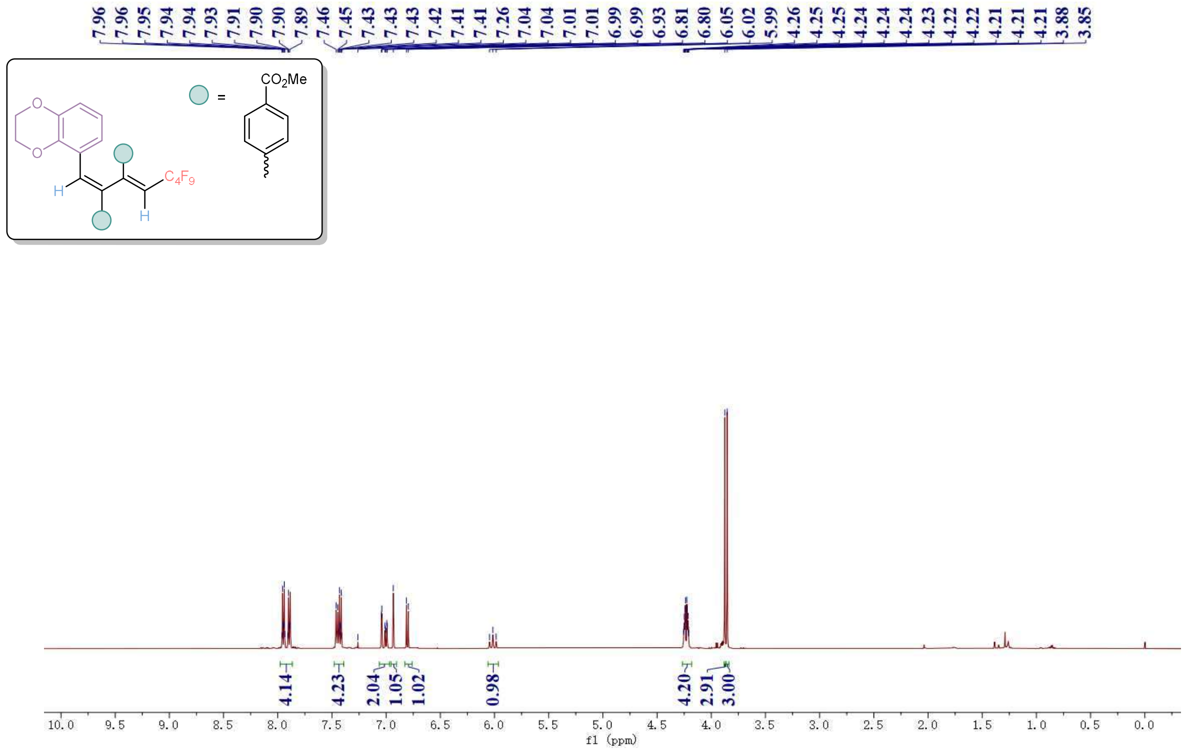

^{19}**F NMR** Spectrum of **53** (471 MHz, Chloroform-*d*)

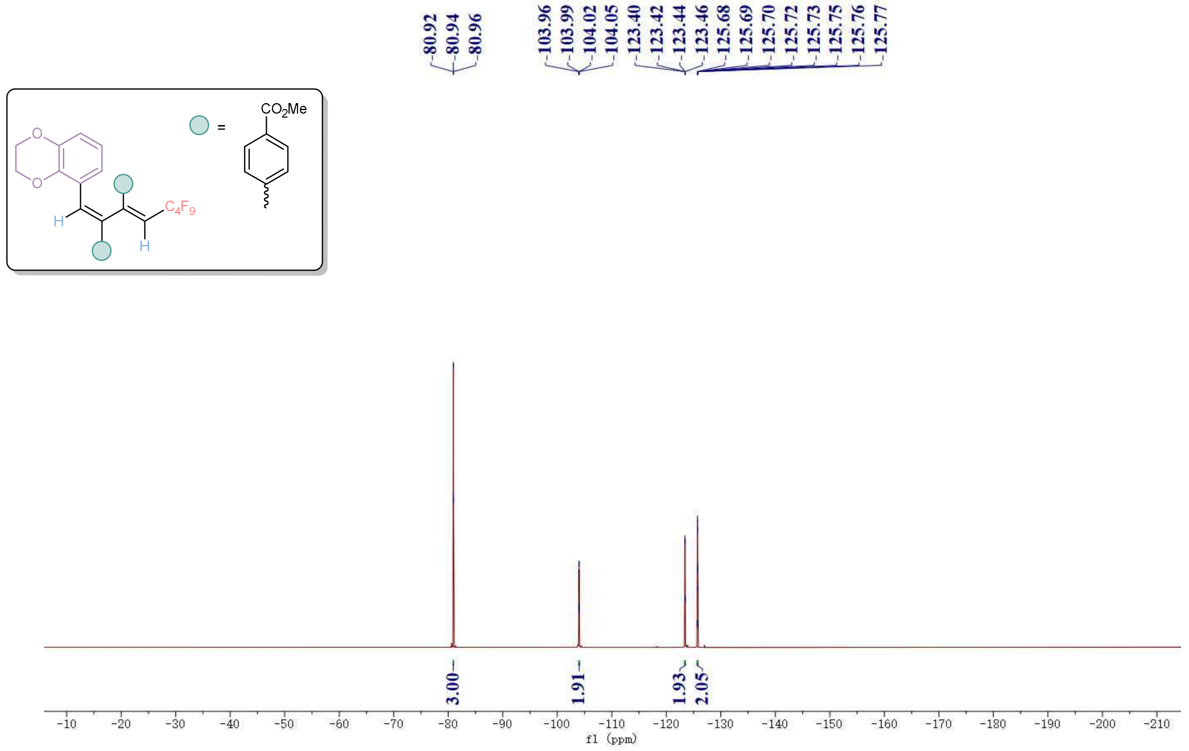

^{13}**C NMR** spectrum of **53** (126 MHz, Chloroform-*d*)

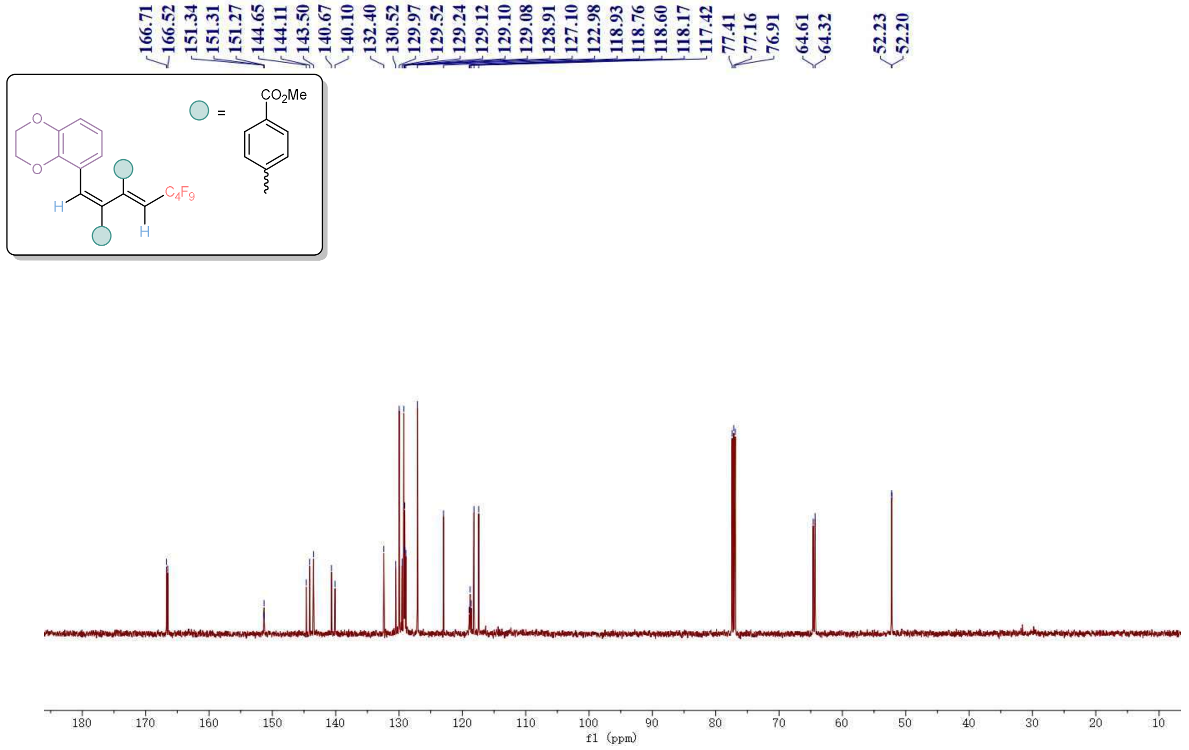

^{1}**H NMR** spectrum of **54** (500 MHz, Chloroform-*d*)

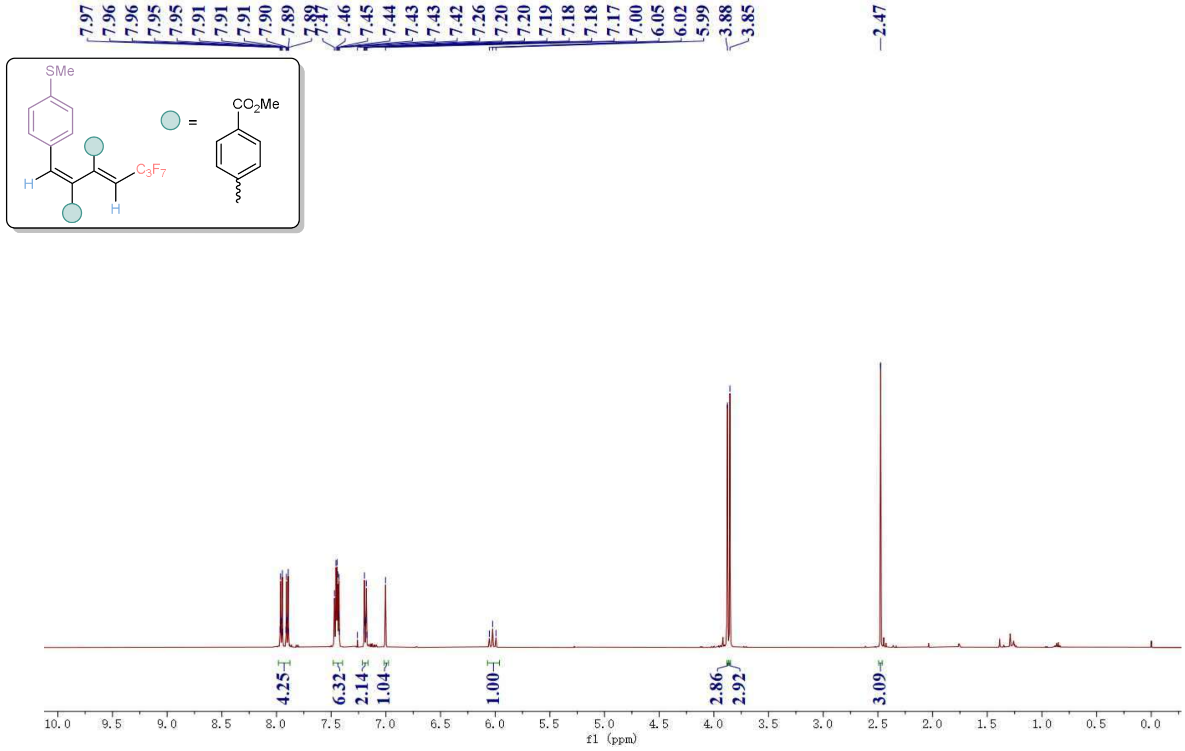

^{19}**F NMR** Spectrum of **54** (471 MHz, Chloroform-*d*)

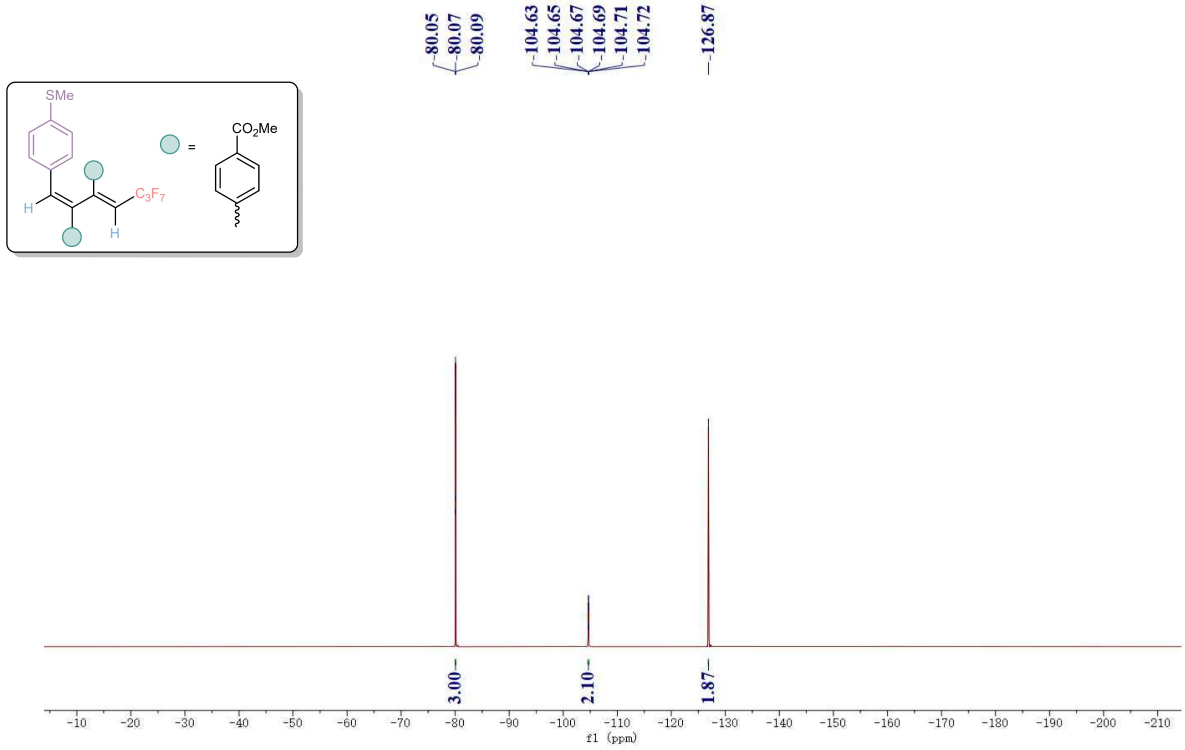

^{13}**C NMR** spectrum of **54** (126 MHz, Chloroform-d)

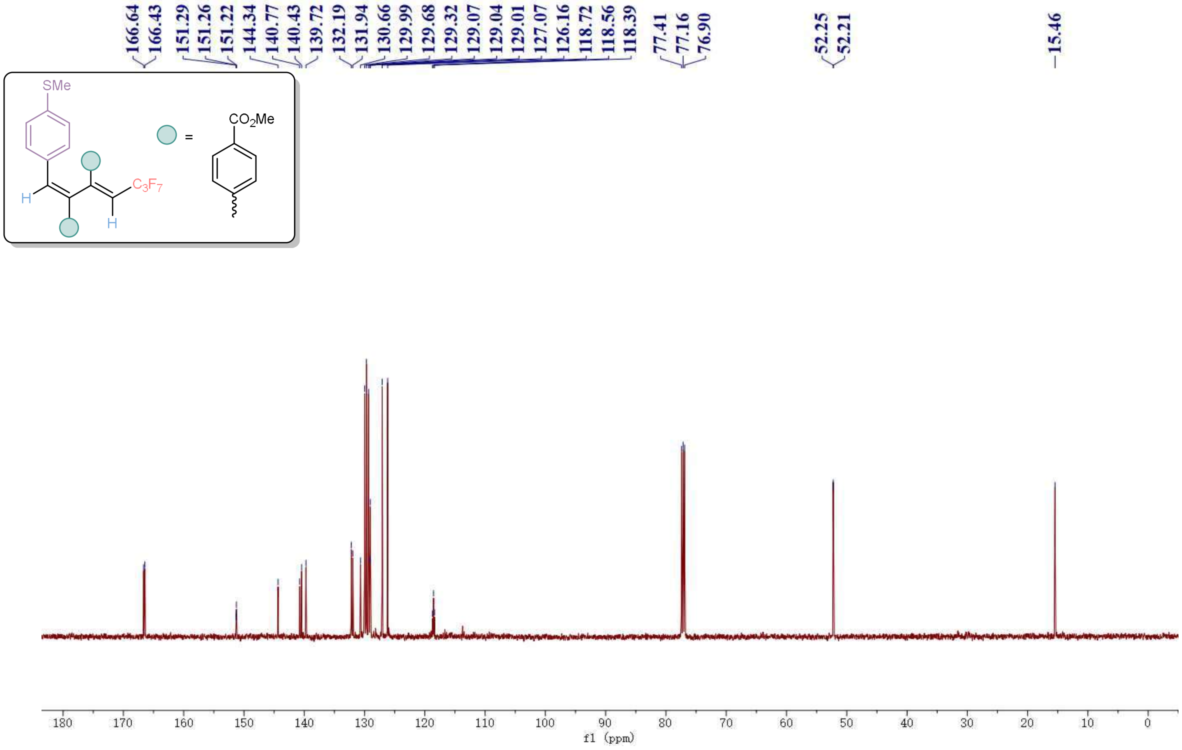

^{1}**H NMR** spectrum of **55** (500 MHz, Chloroform-*d*)

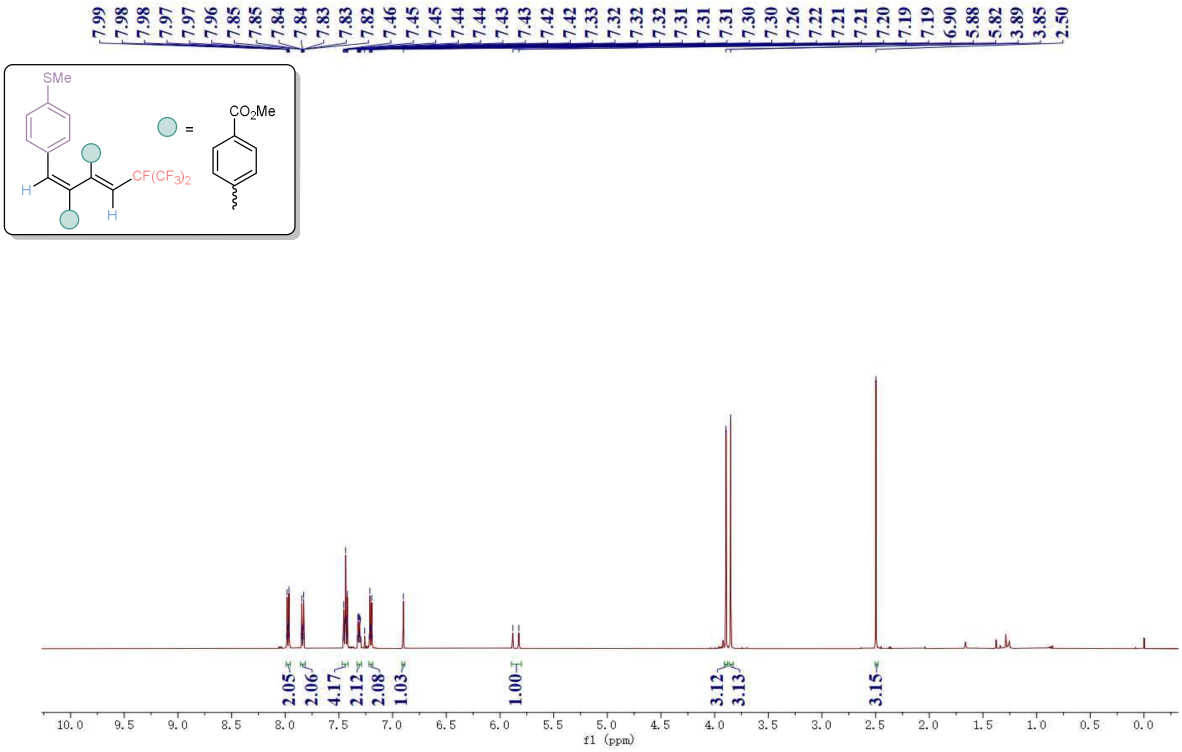

^{19}F NMR Spectrum of **55** (471 MHz, Chloroform-*d*)

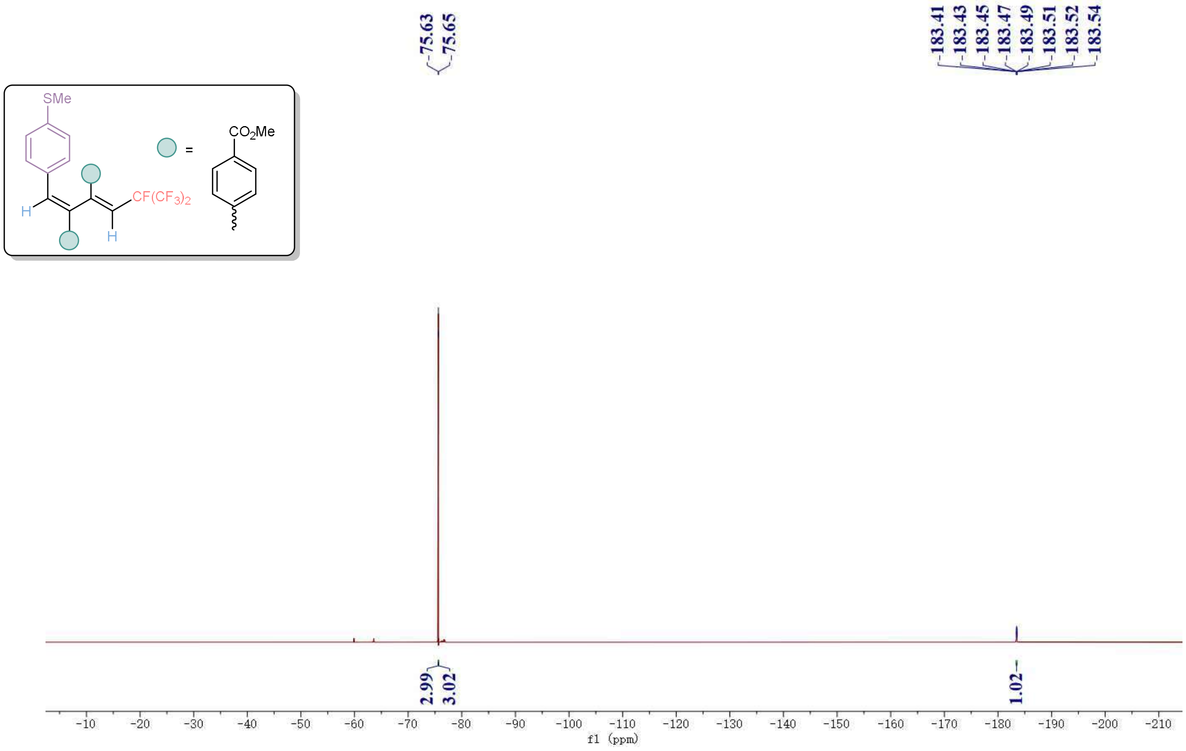

^{13}C NMR spectrum of **55** (126 MHz, Chloroform-d)

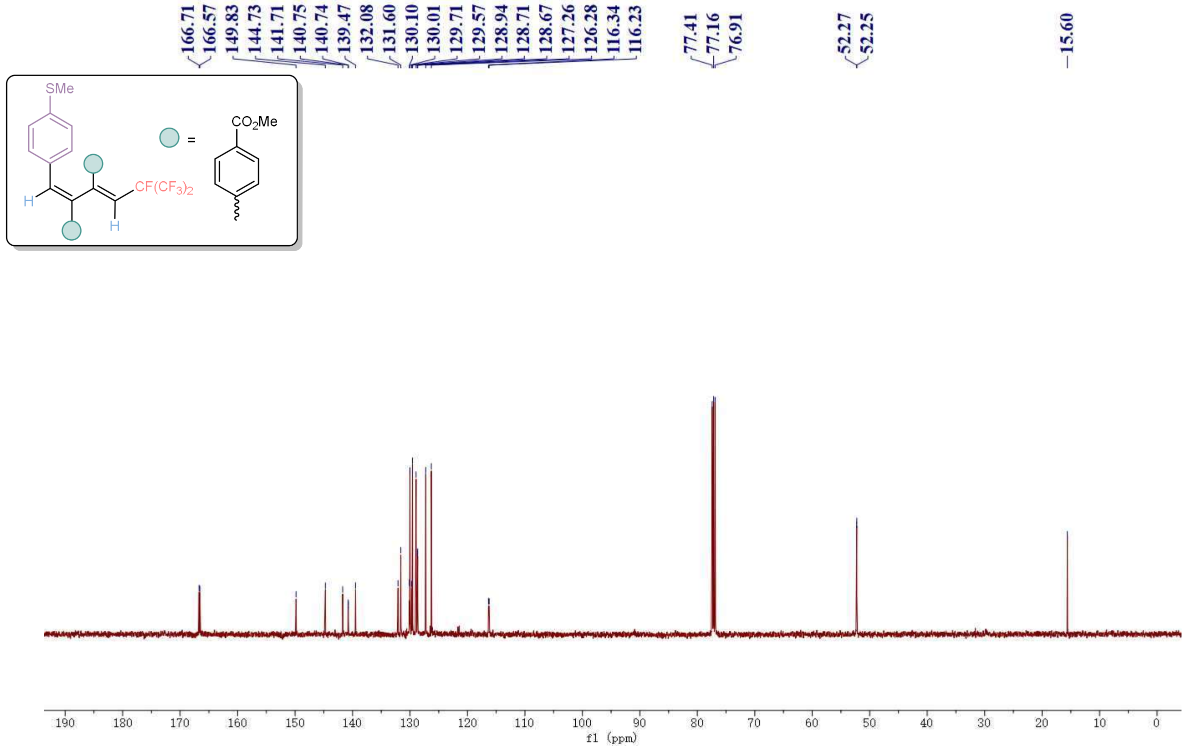

^{1}H NMR spectrum of **56** (500 MHz, Chloroform-*d*)

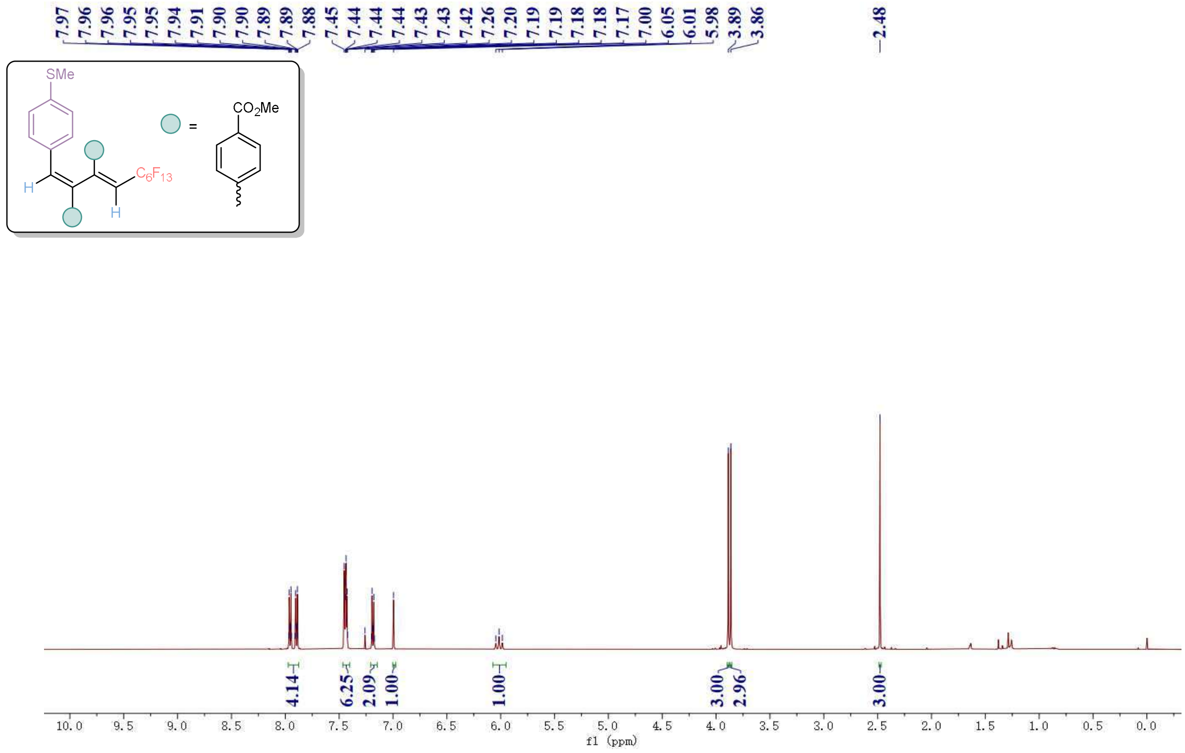

^{19}F NMR Spectrum of **56** (471 MHz, Chloroform-*d*)

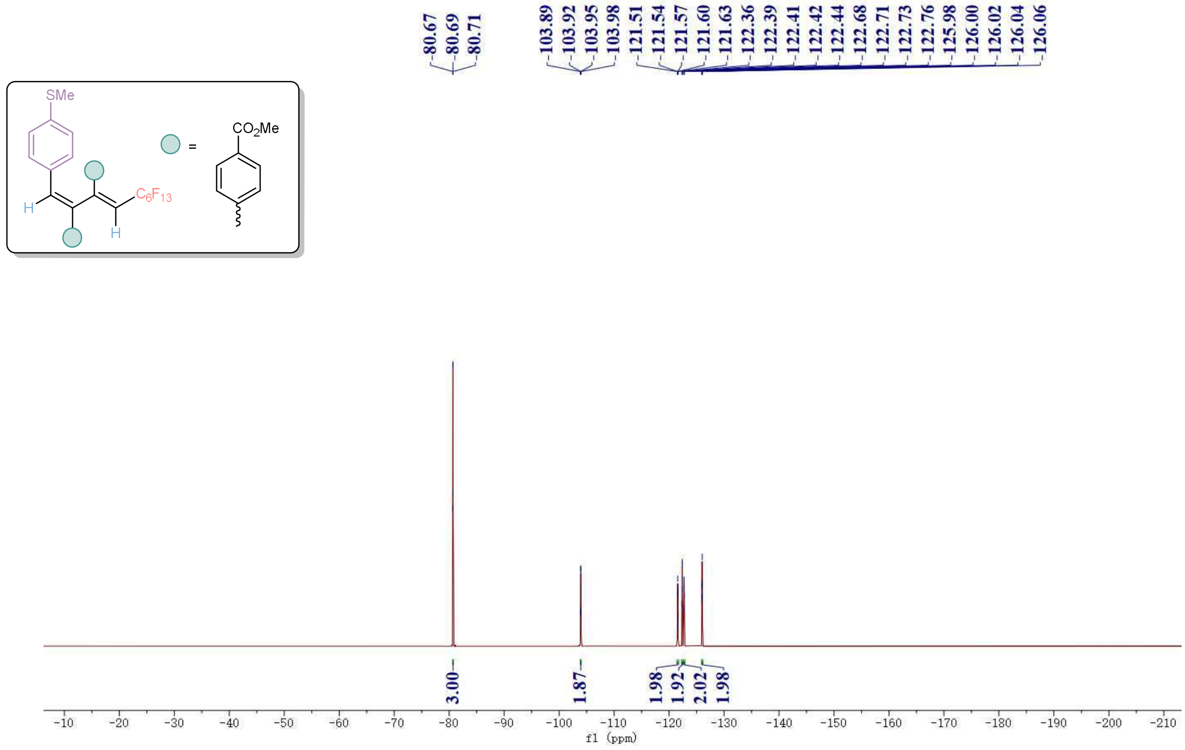

^{13}**C NMR** spectrum of **56** (126 MHz, Chloroform-d)

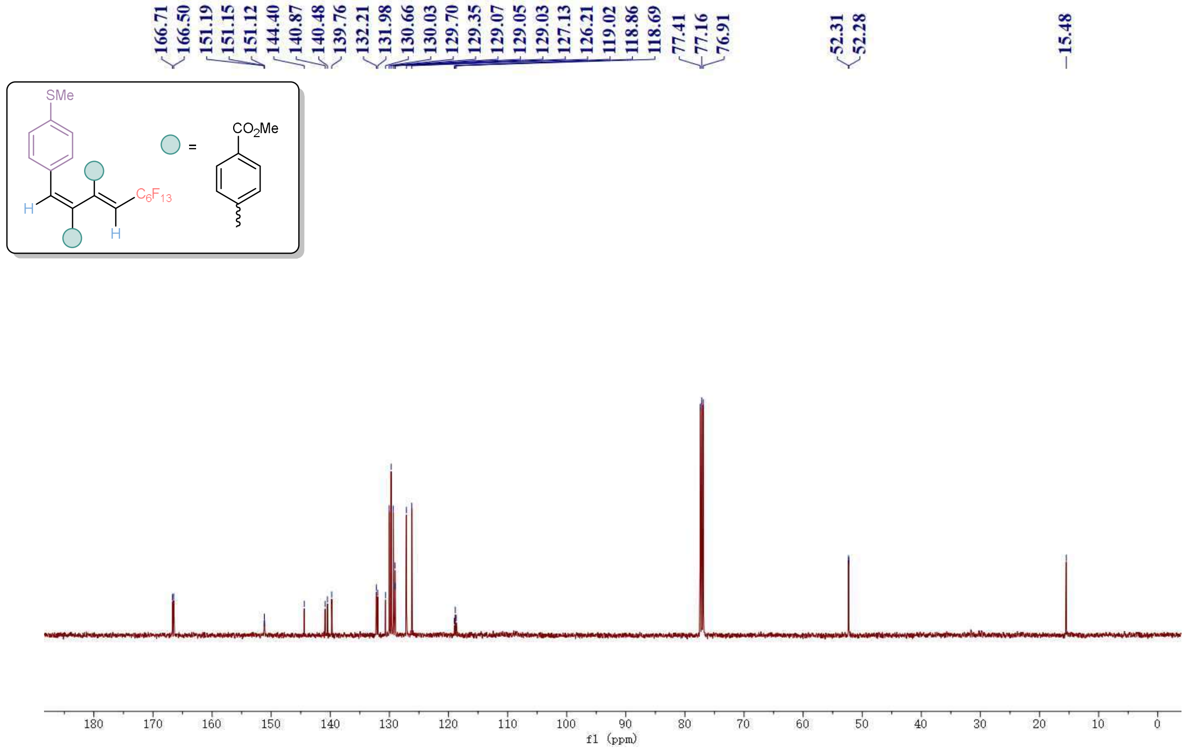

^{1}**H NMR** spectrum of **57** (500 MHz, Chloroform-*d*)

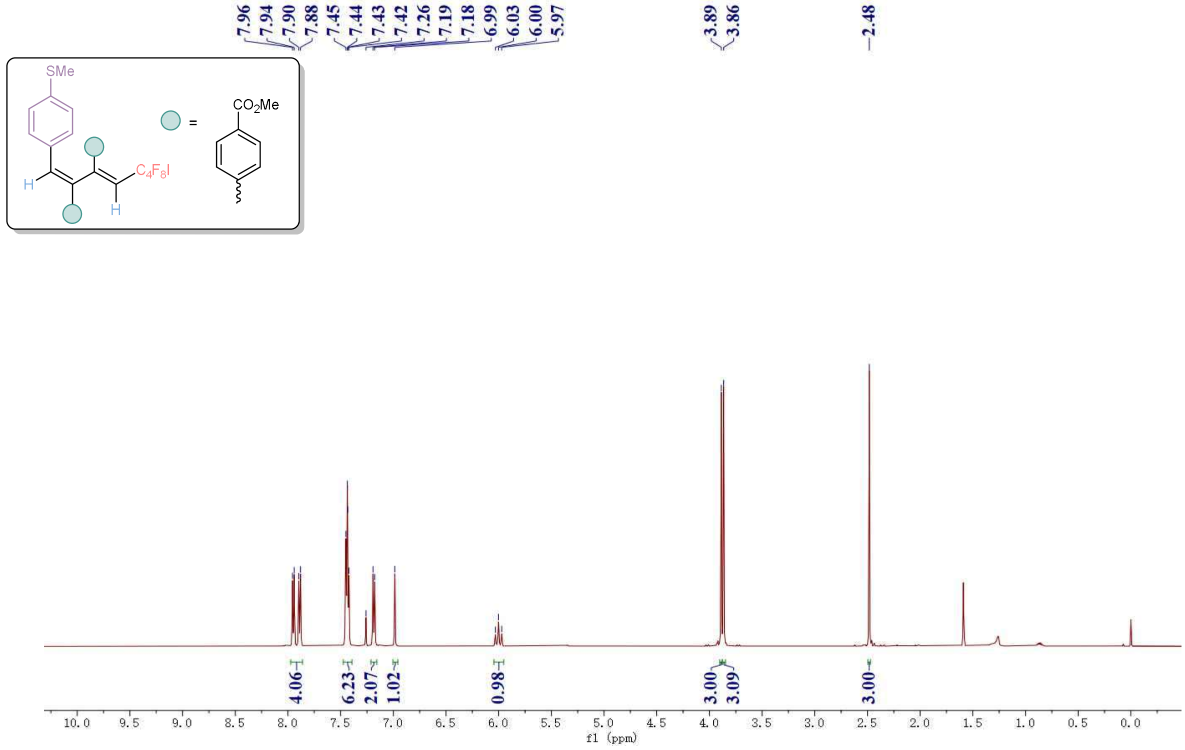

^{19}F NMR Spectrum of **57** (471 MHz, Chloroform-*d*)

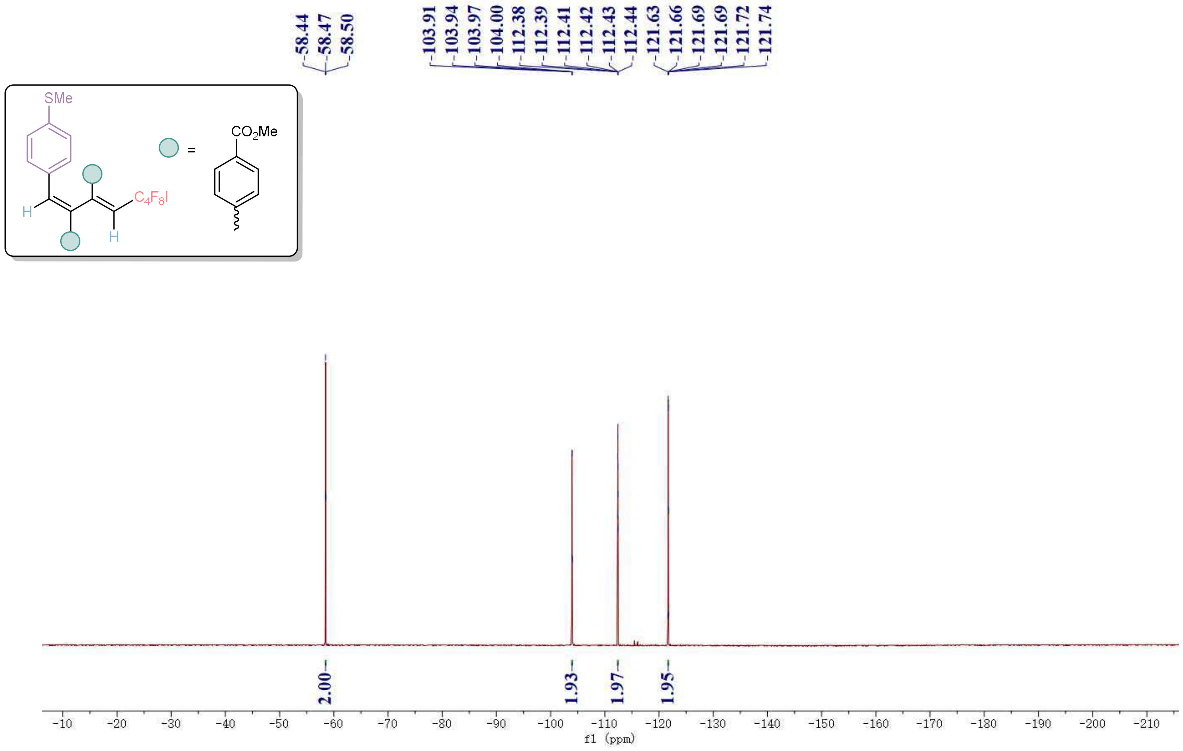

^{13}C NMR spectrum of **57** (126 MHz, Chloroform-*d*)

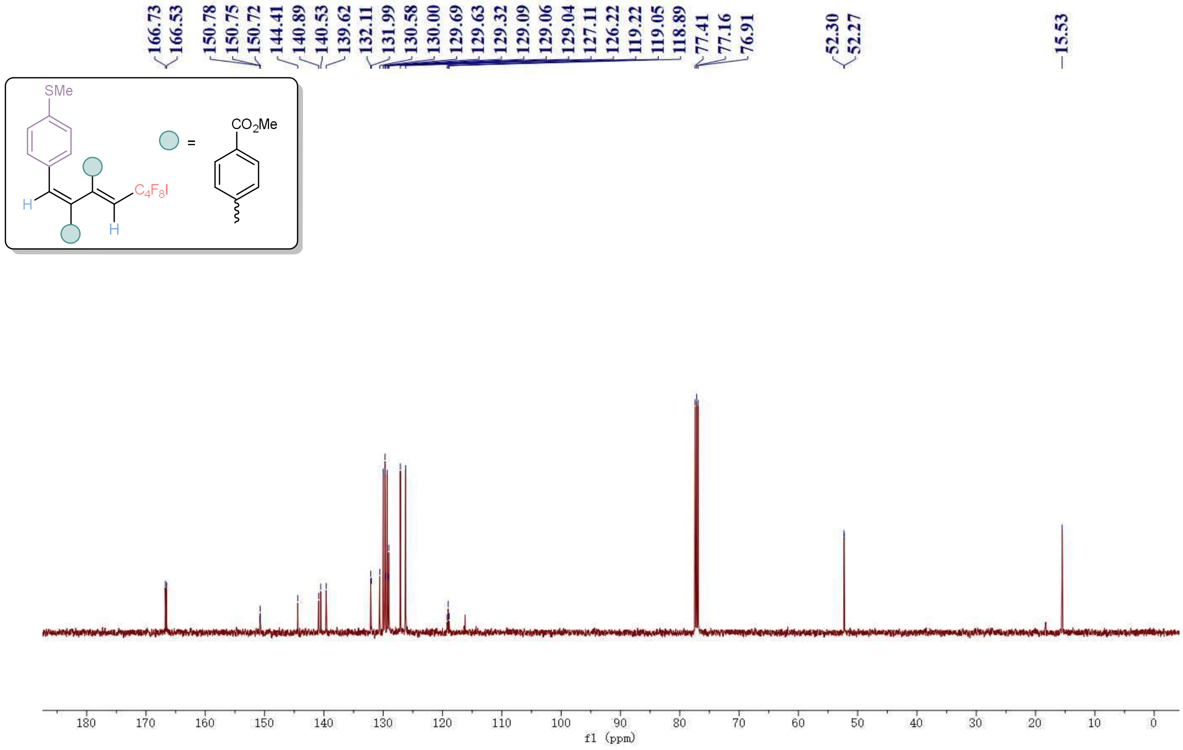

^{1}H NMR spectrum of **58** (500 MHz, Chloroform-*d*)

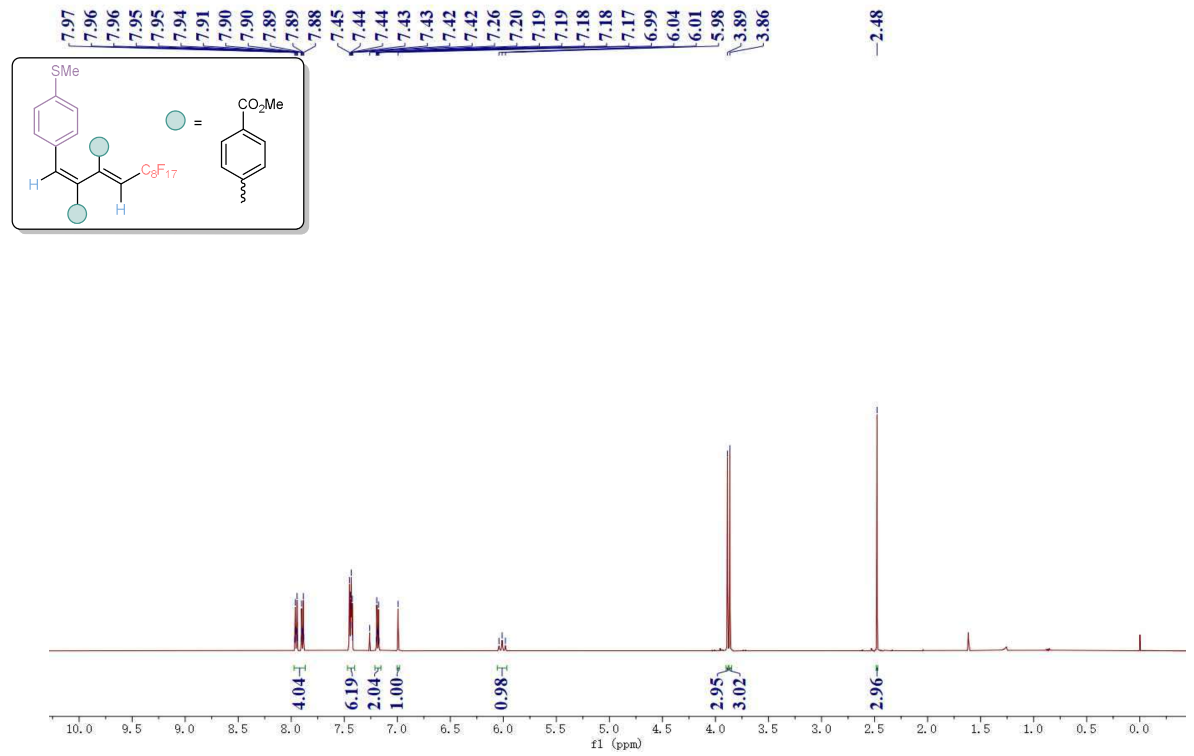

^{19}F NMR Spectrum of **58** (471 MHz, Chloroform-*d*)

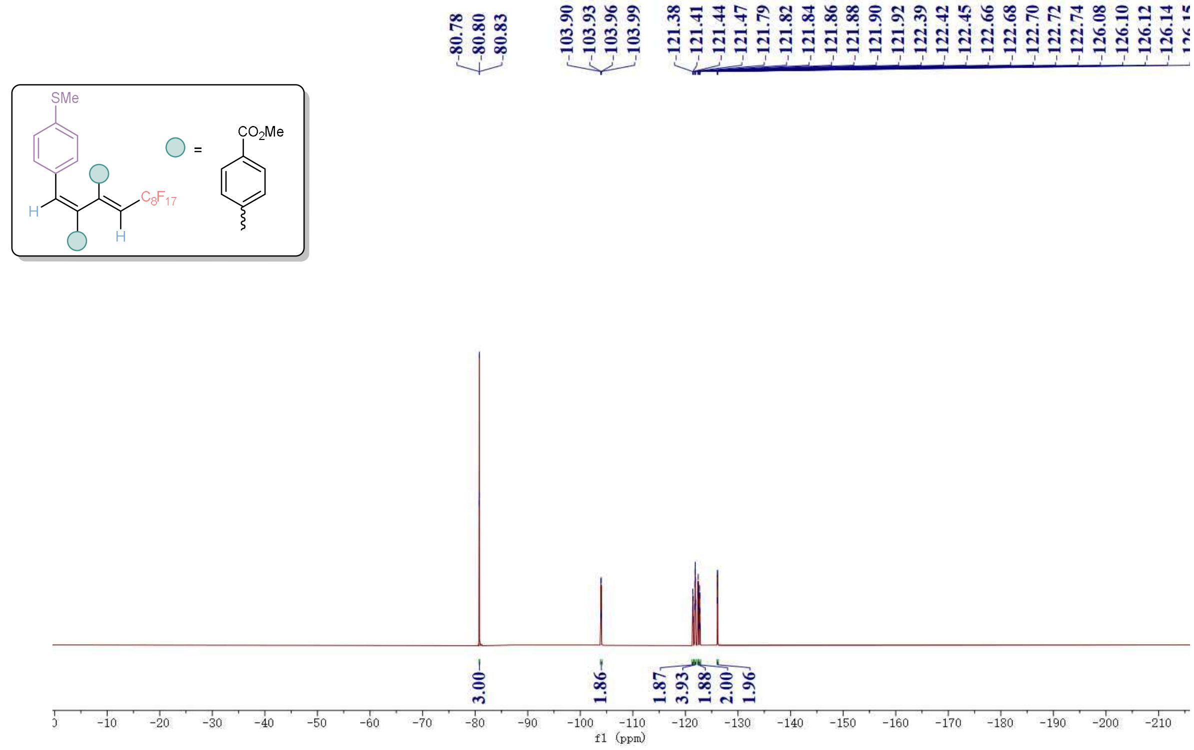

^{13}C NMR spectrum of **58** (126 MHz, Chloroform-*d*)

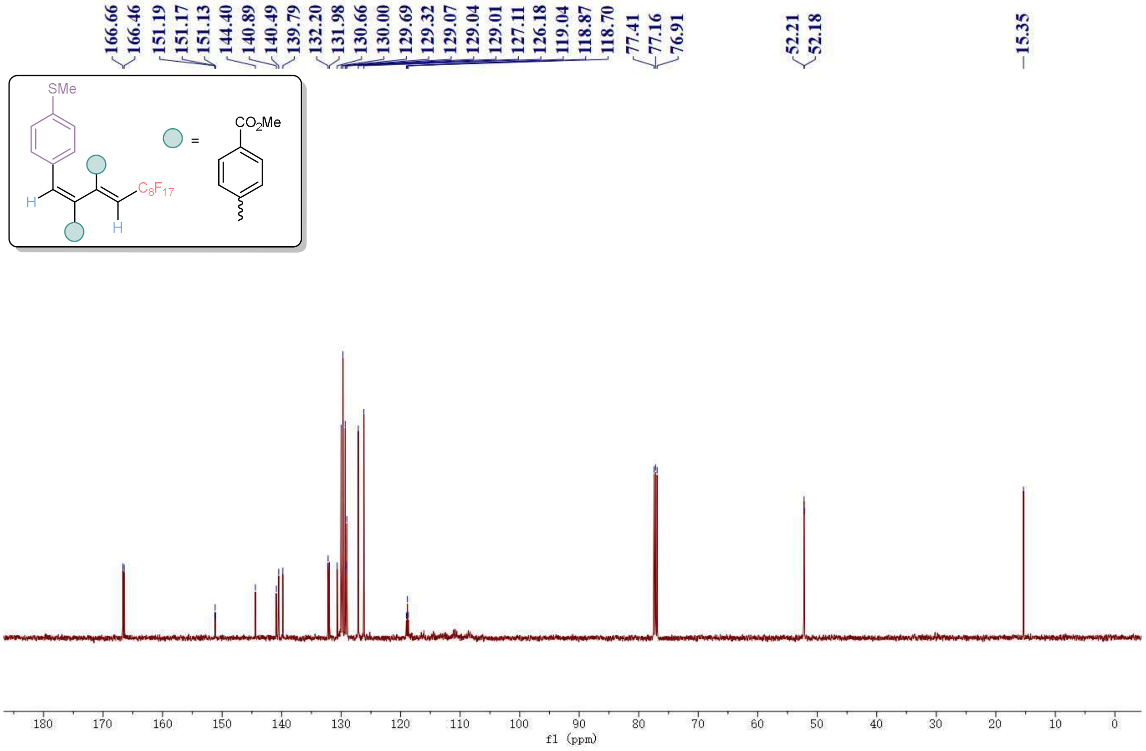

^{1}H NMR spectrum of **59** (500 MHz, Chloroform-*d*)

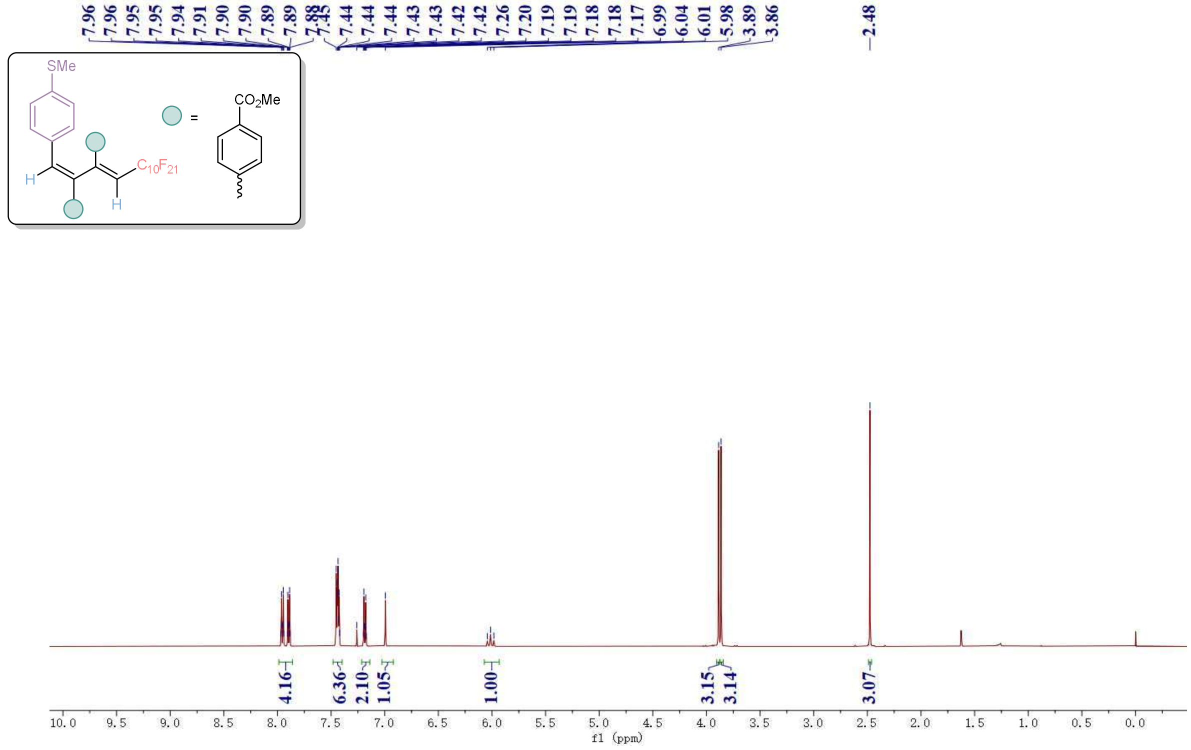

^{19}F NMR Spectrum of **59** (471 MHz, Chloroform-*d*)

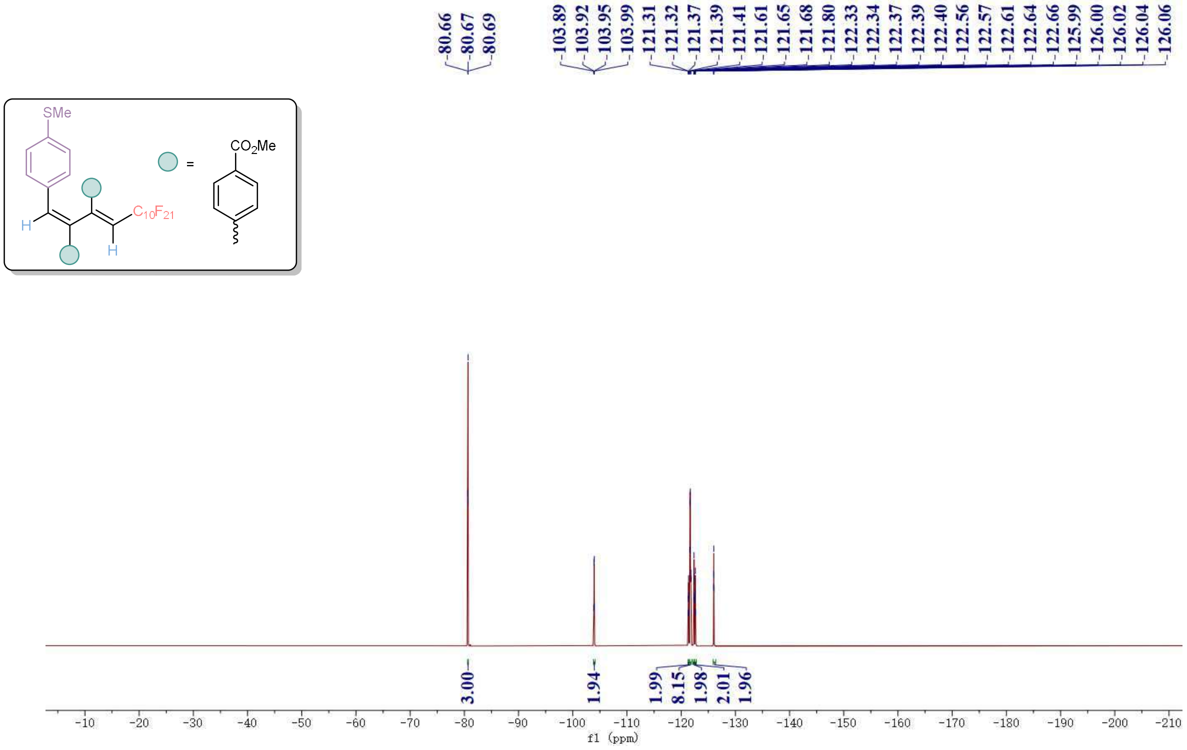

^{13}C NMR spectrum of **59** (126 MHz, Chloroform-*d*)

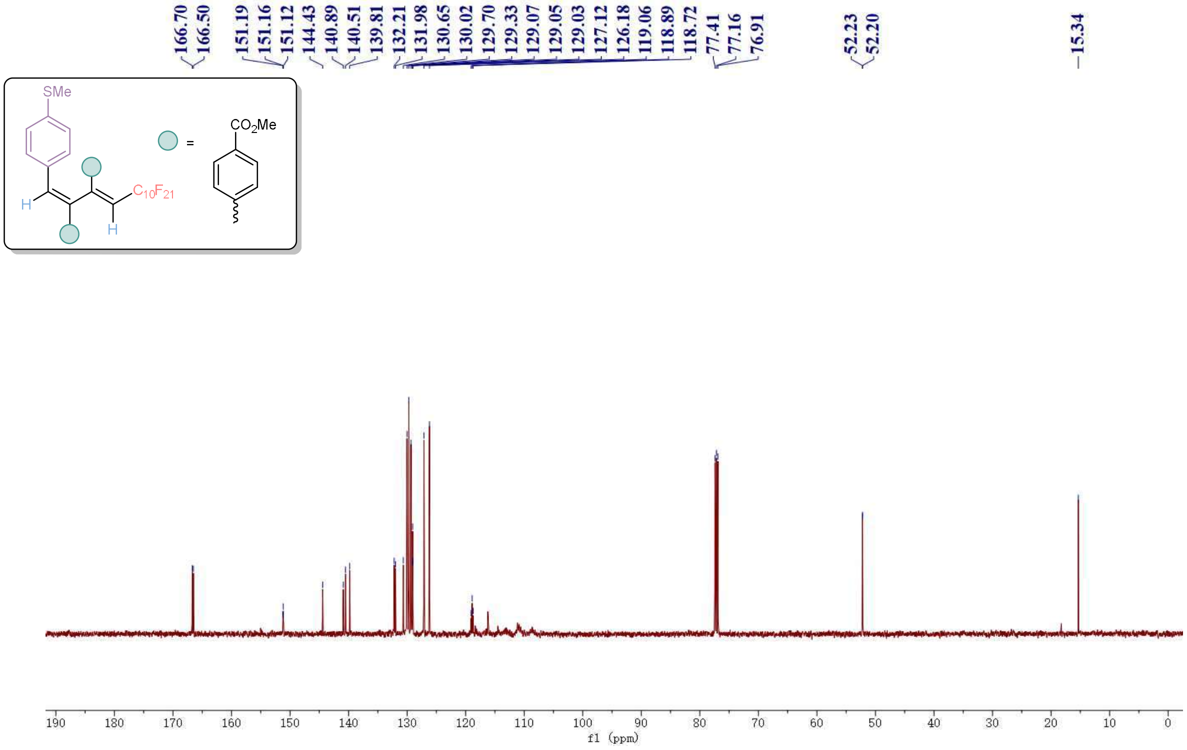

^{1}H NMR spectrum of **60** (500 MHz, Chloroform-*d*)

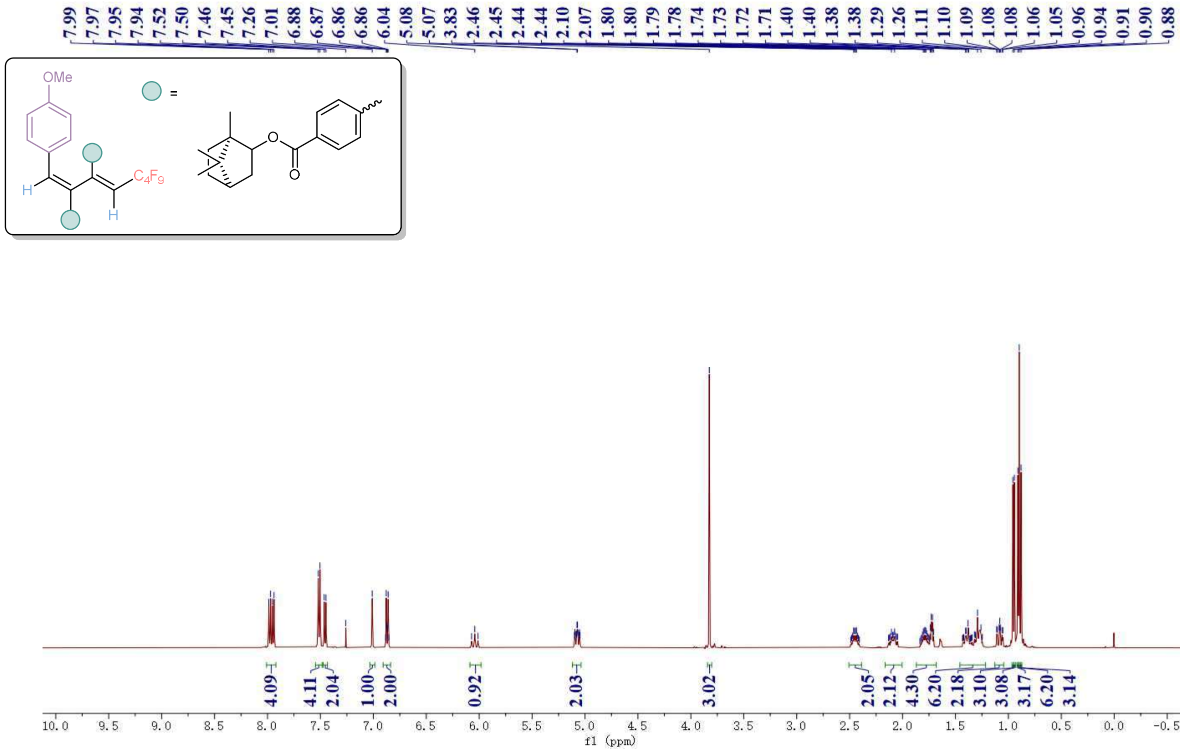

^{19}F NMR Spectrum of **60** (471 MHz, Chloroform-*d*)

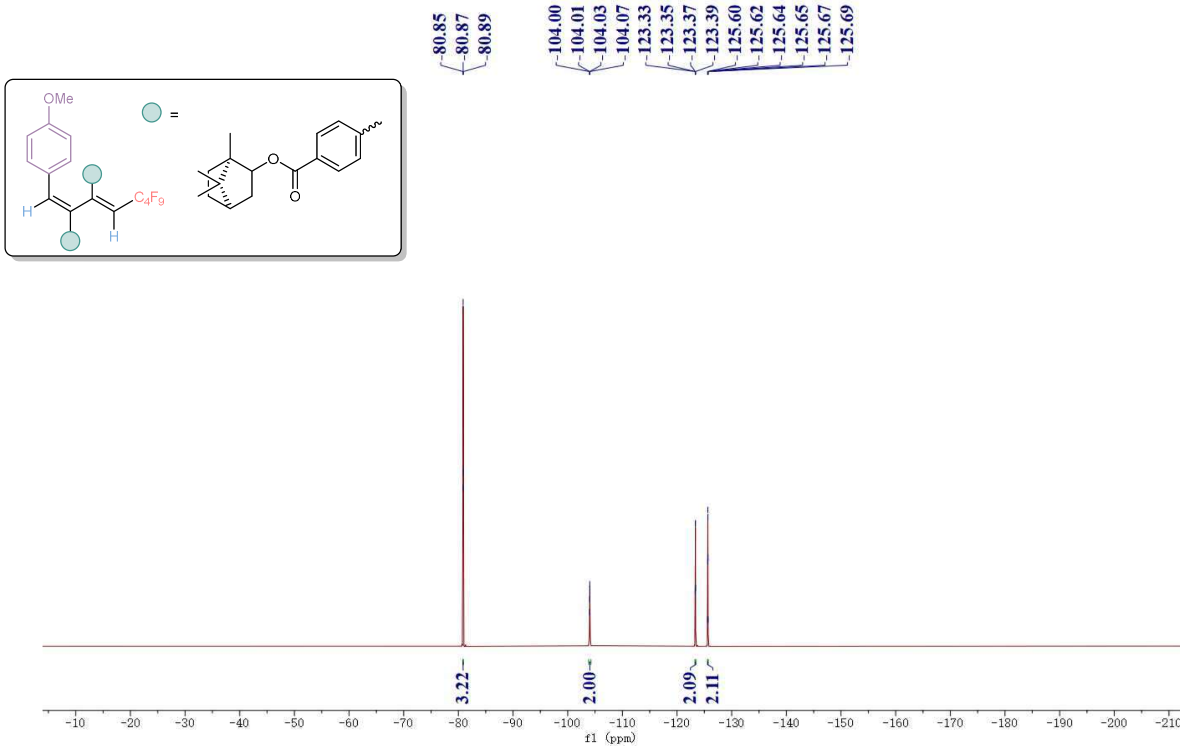

^{13}**C NMR** spectrum of **60** (126 MHz, Chloroform-*d*)

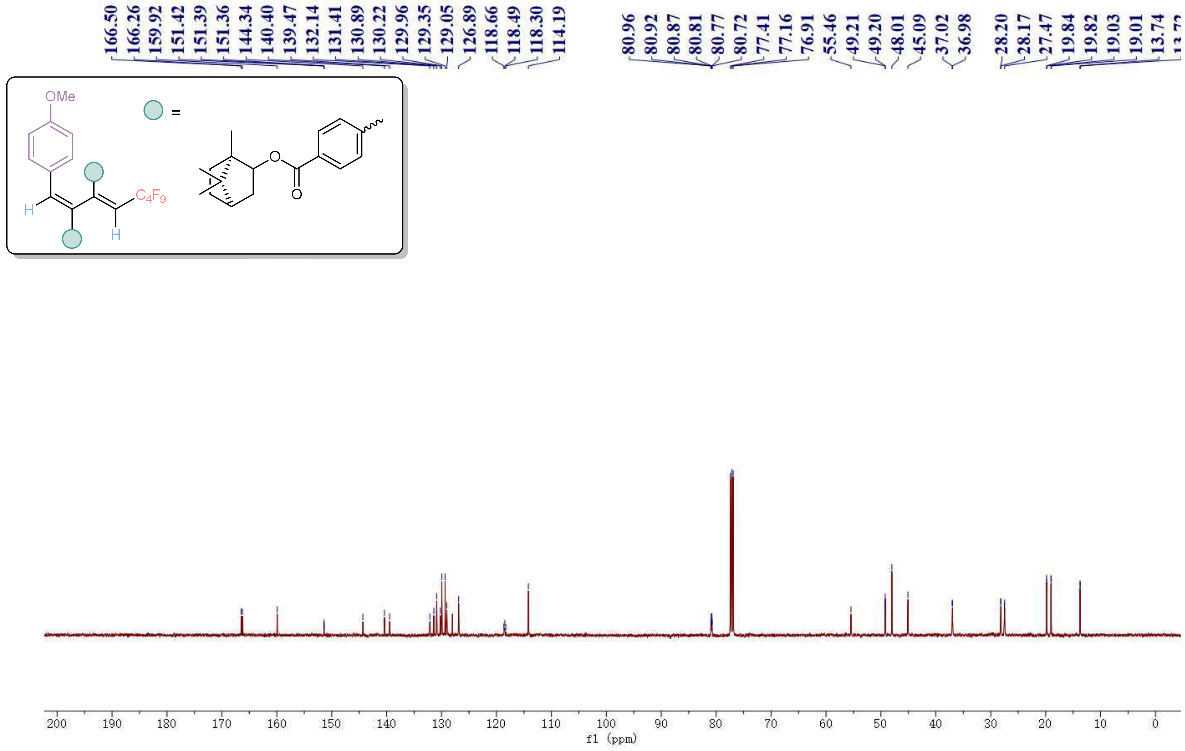

^{1}**H NMR** spectrum of **61** (500 MHz, Chloroform-*d*)

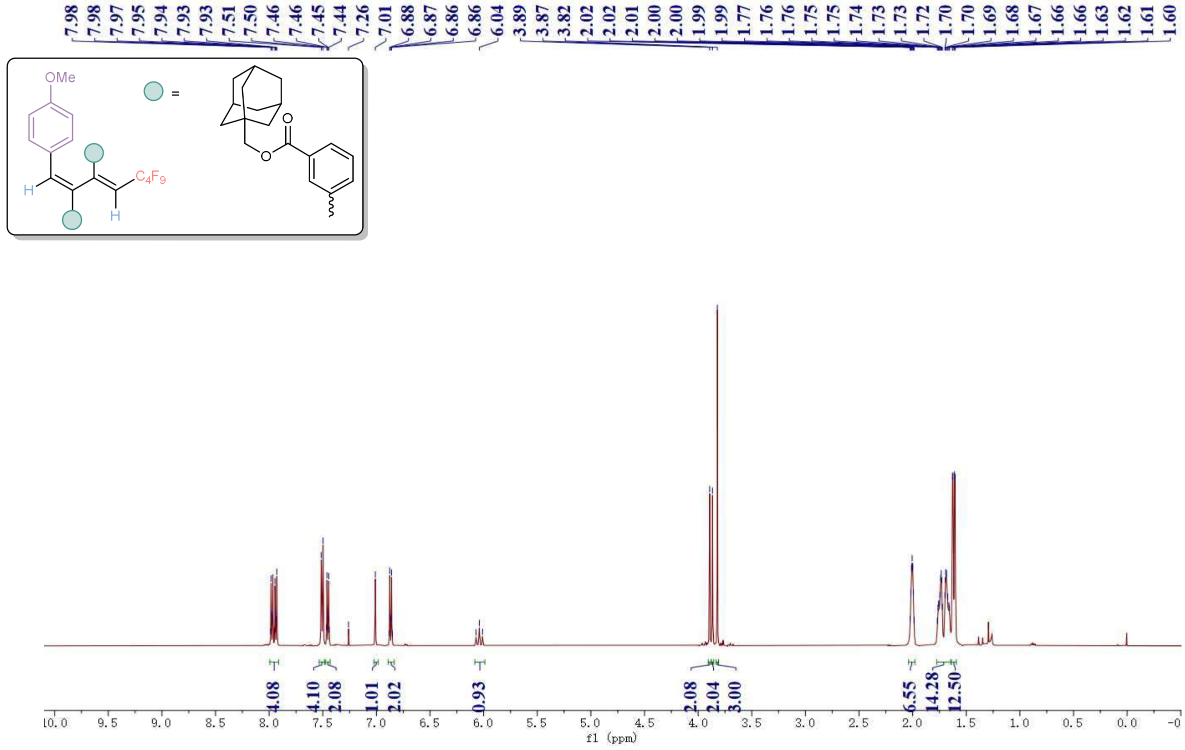

^{19}**F NMR** Spectrum of **61**(471 MHz, Chloroform-*d*)

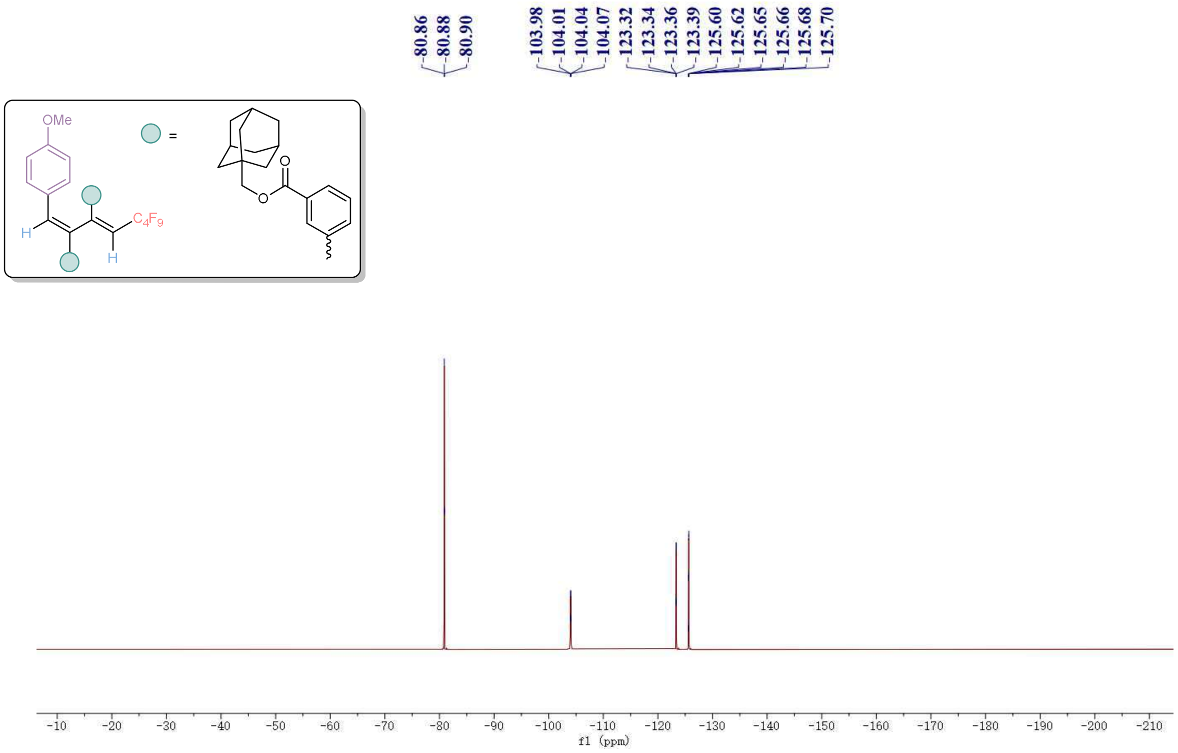

^{13}**C NMR** spectrum of **61** (126 MHz, Chloroform-*d*)

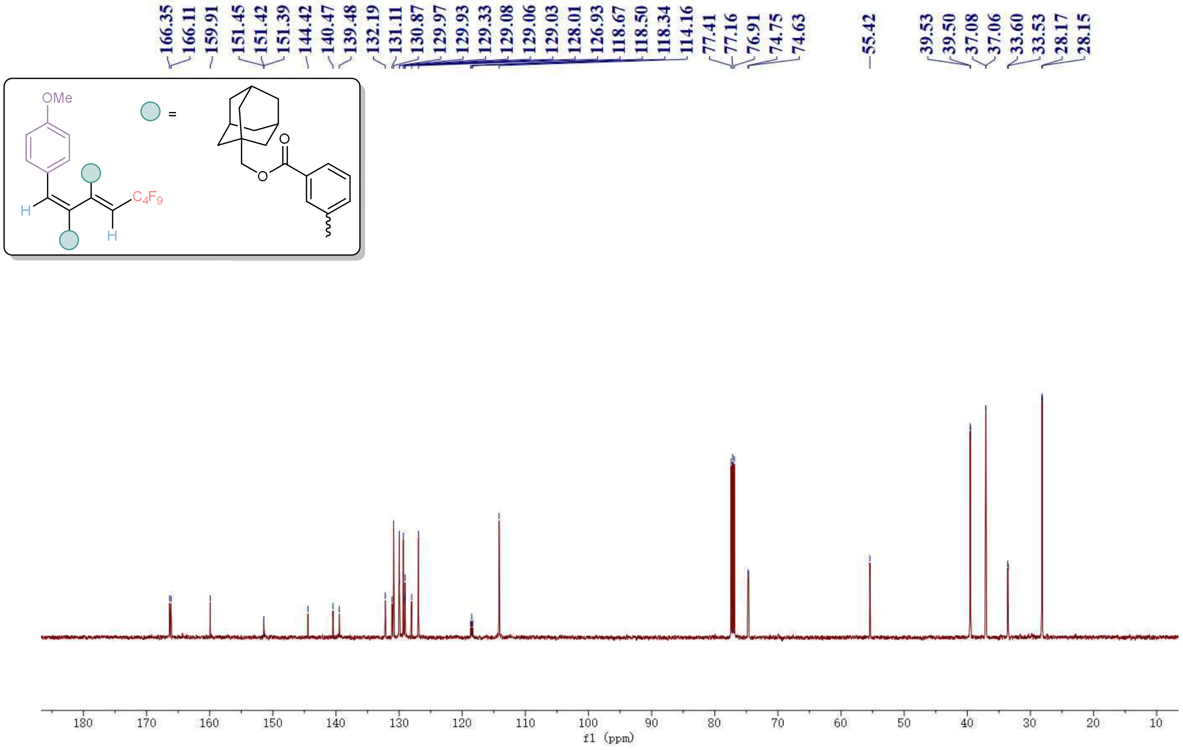

[1]**H NMR** spectrum of **62** (500 MHz, Chloroform-*d*)

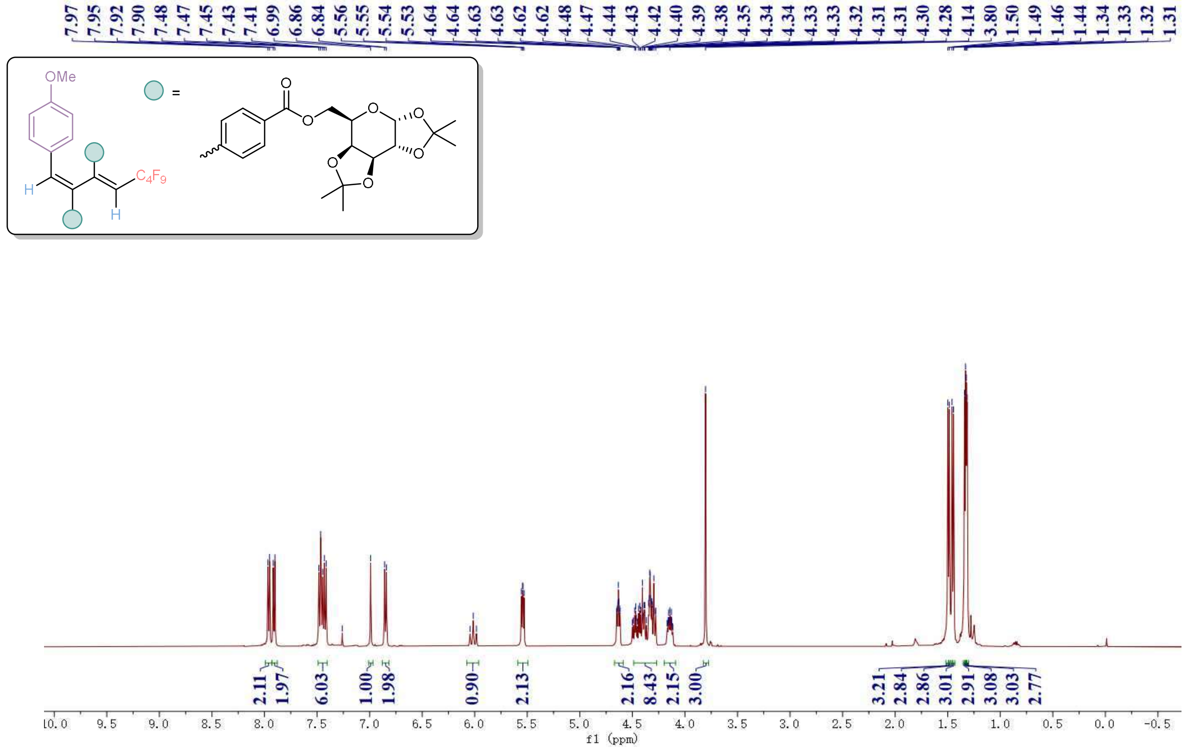

[19]**F NMR** Spectrum of **62** (471 MHz, Chloroform-*d*)

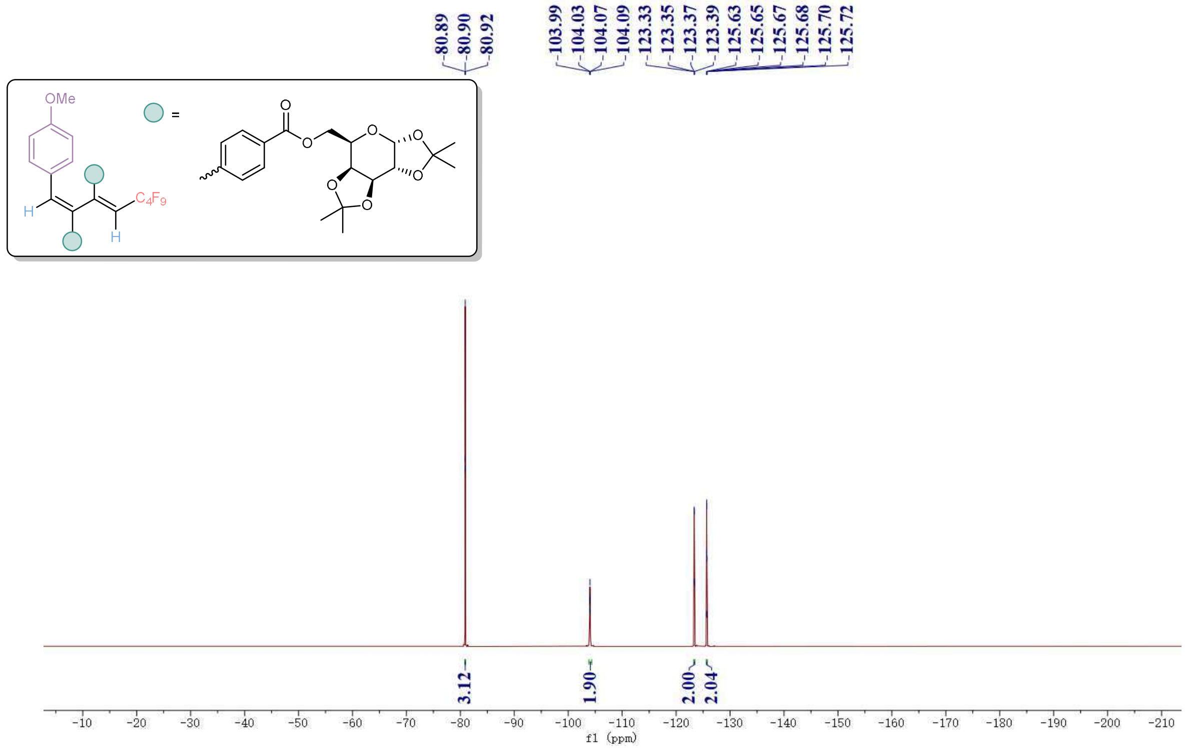

^{13}C **NMR** spectrum of **62** (126 MHz, Chloroform-d)

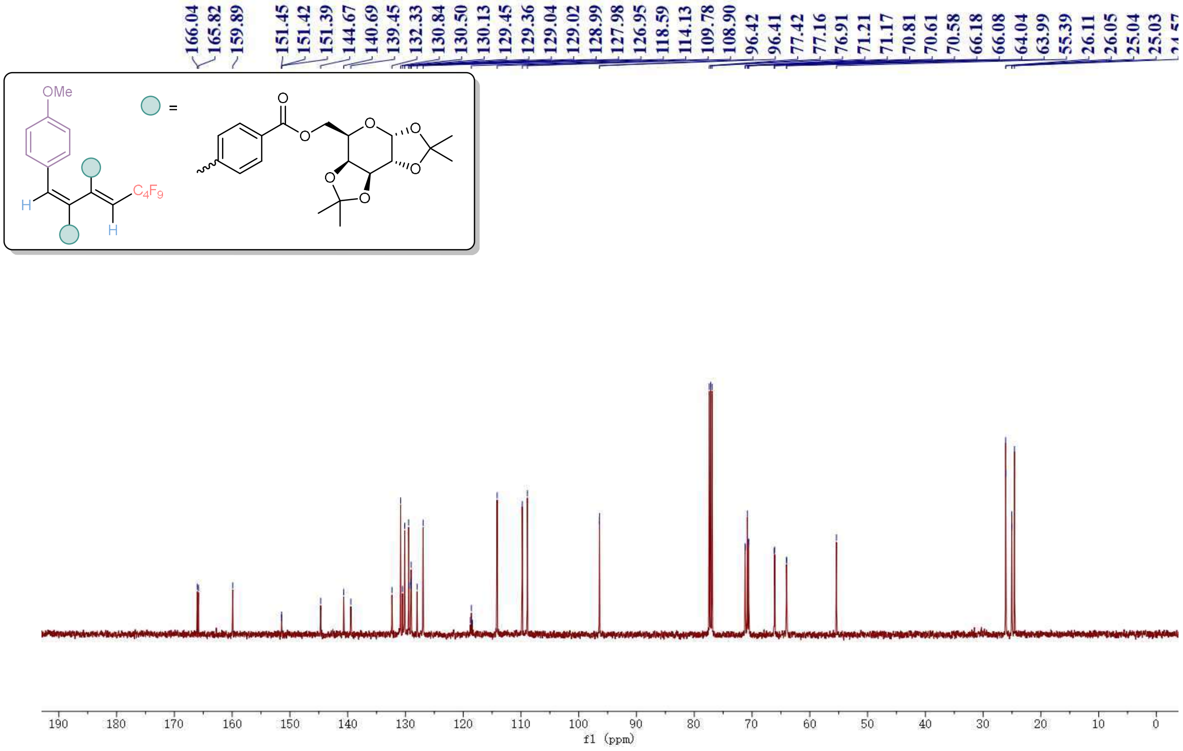

^{1}H **NMR** spectrum of **63** (500 MHz, Chloroform-*d*)

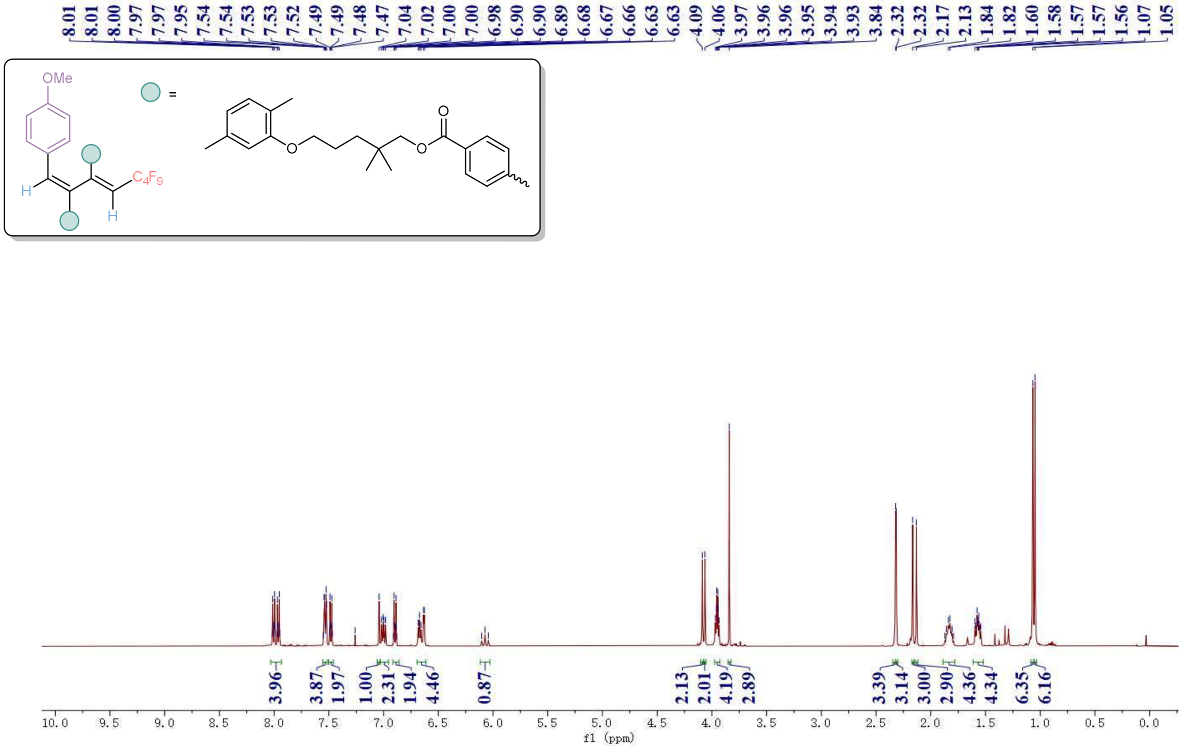

^{19}**F NMR** Spectrum of **63** (471 MHz, Chloroform-*d*)

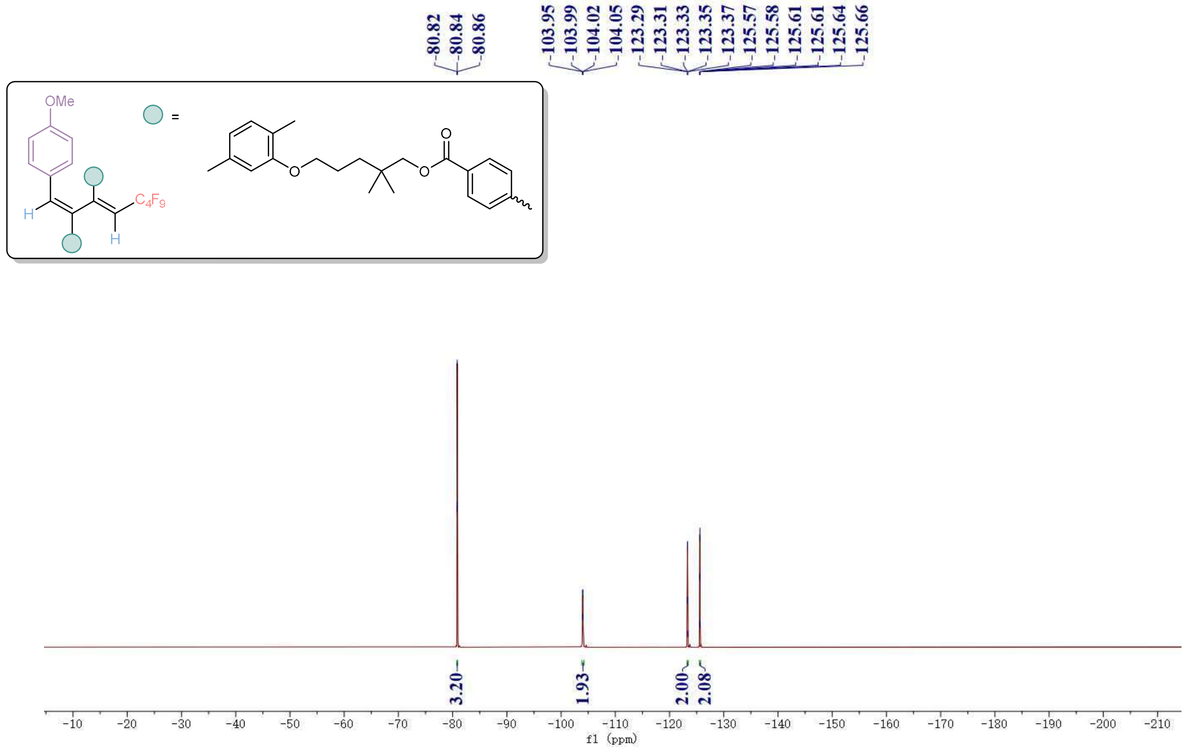

^{13}**C NMR** spectrum of **63** (126 MHz, Chloroform-d)

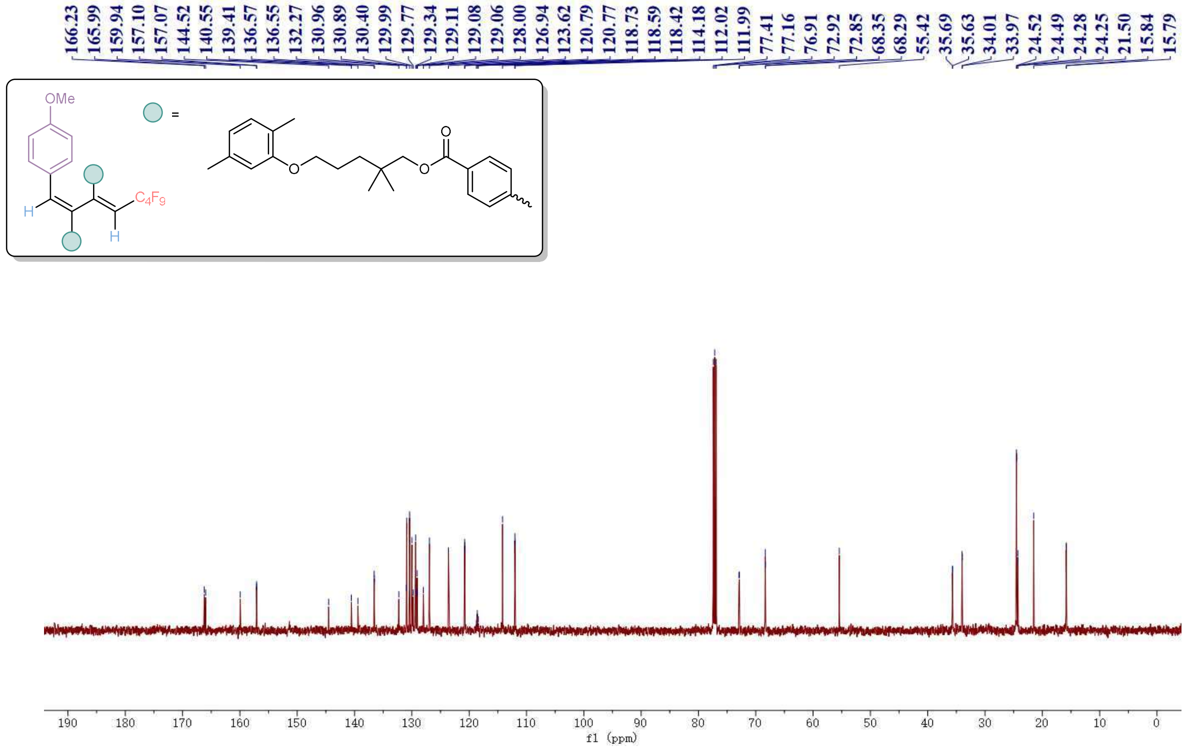

^{1}H NMR spectrum of **64** (500 MHz, Chloroform-*d*)

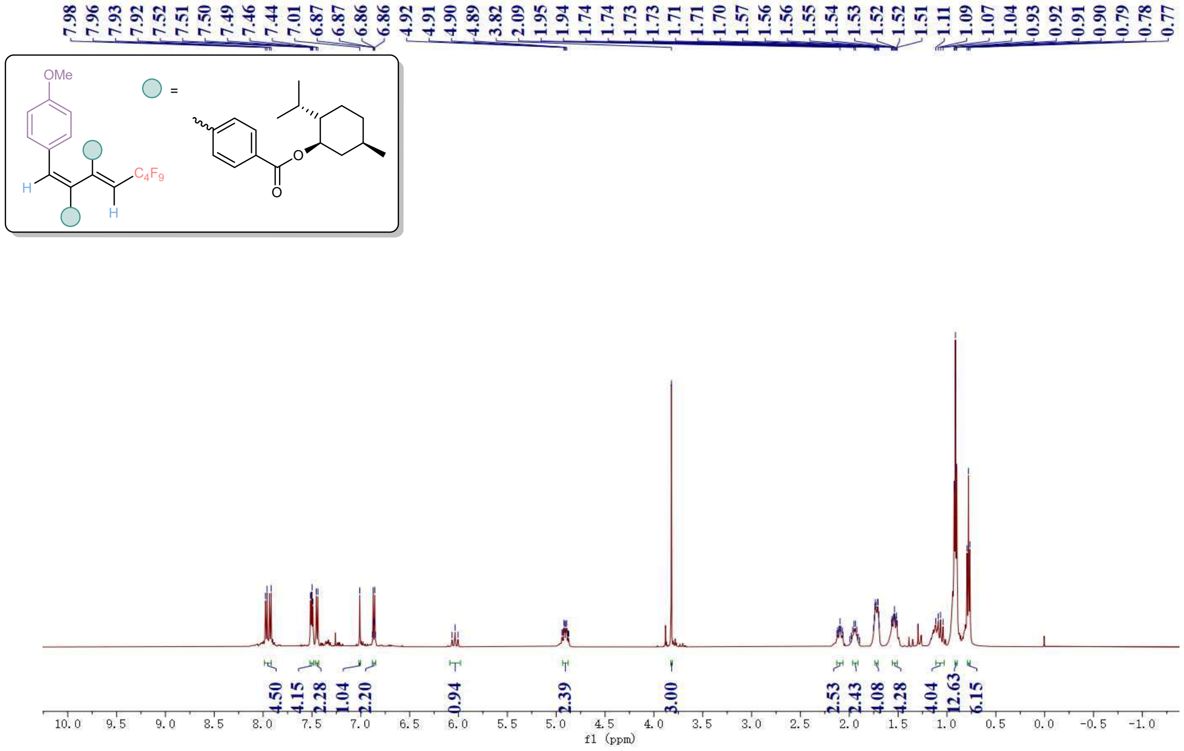

^{19}F NMR Spectrum of **64** (471 MHz, Chloroform-*d*)

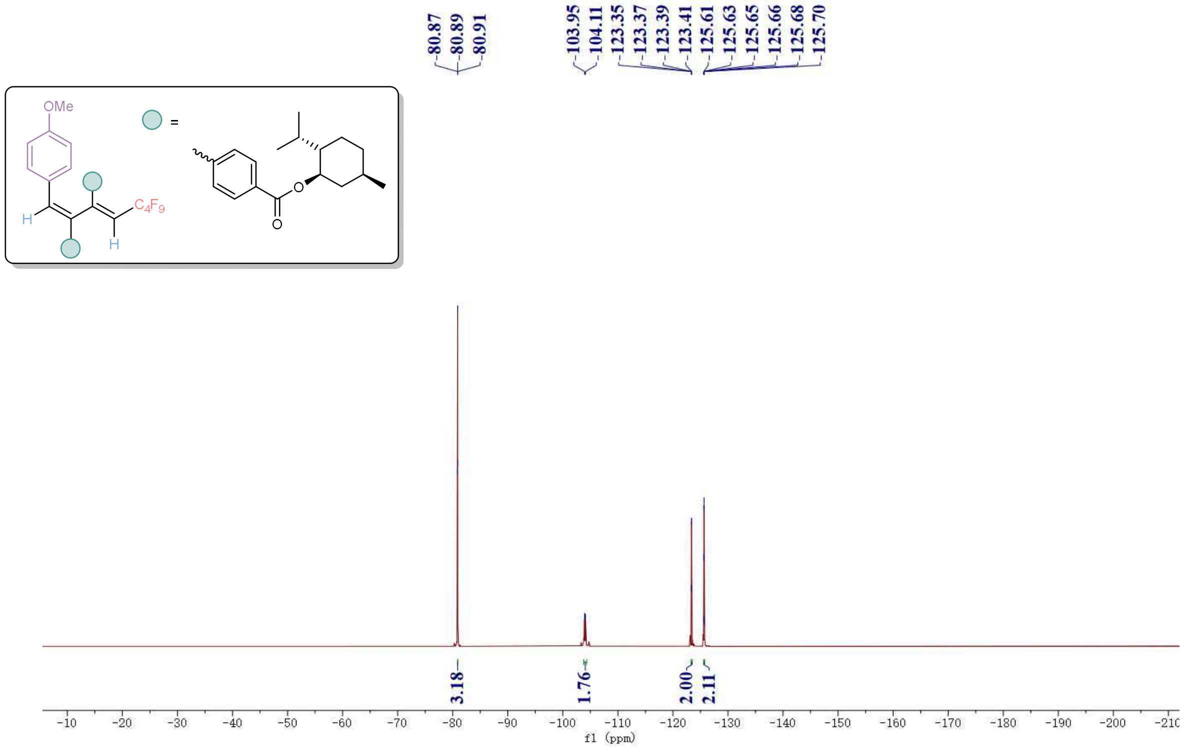

^{13}C NMR spectrum of **64** (126 MHz, Chloroform-*d*)

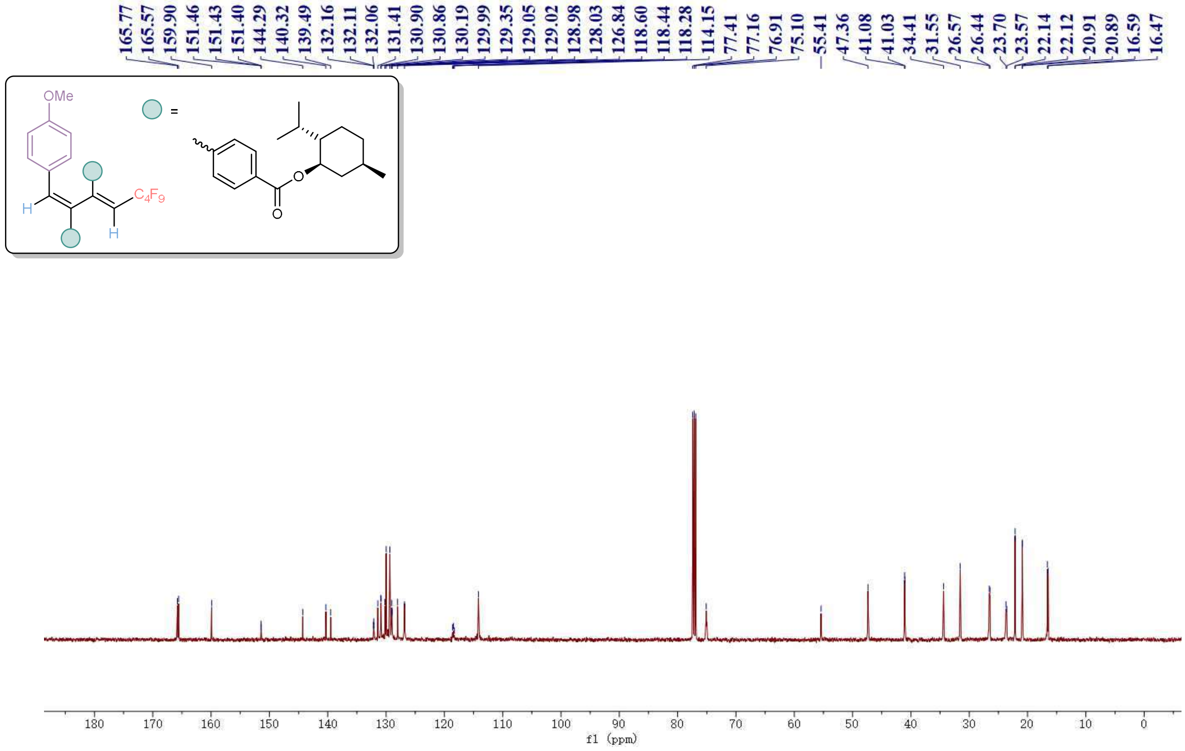

^{1}H NMR spectrum of **65** (500 MHz, Chloroform-*d*)

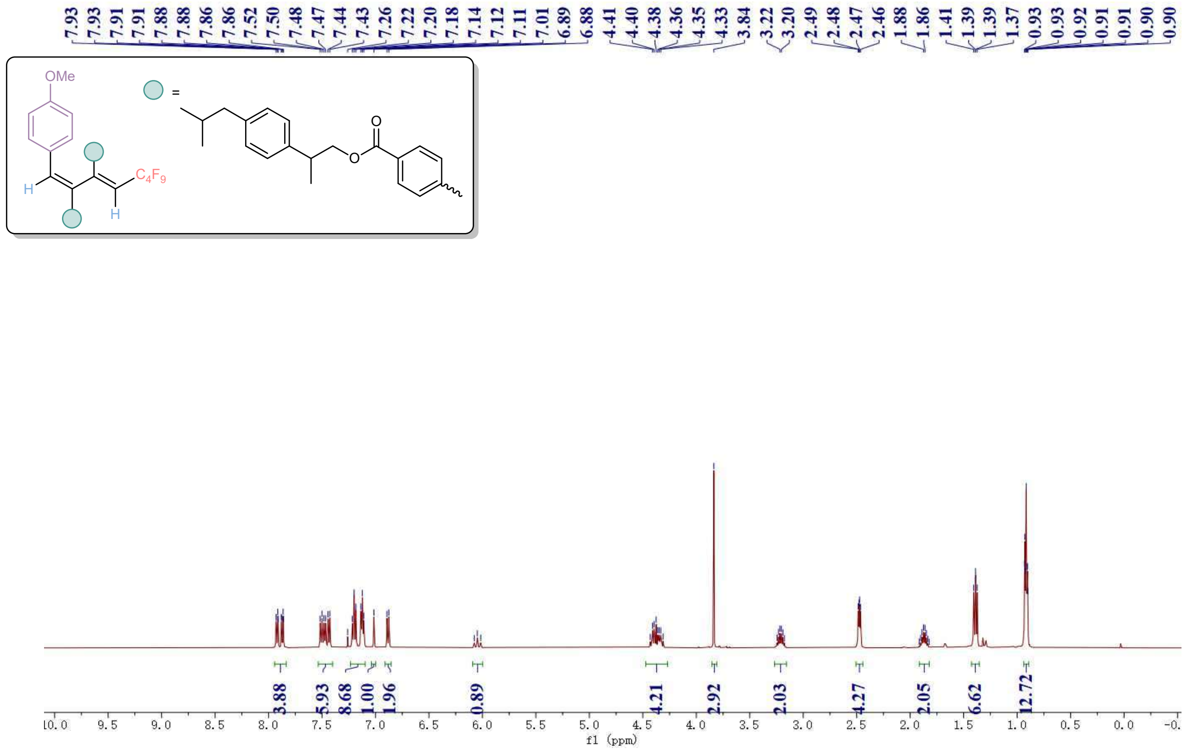

^{19}**F NMR** Spectrum of **65** (471 MHz, Chloroform-*d*)

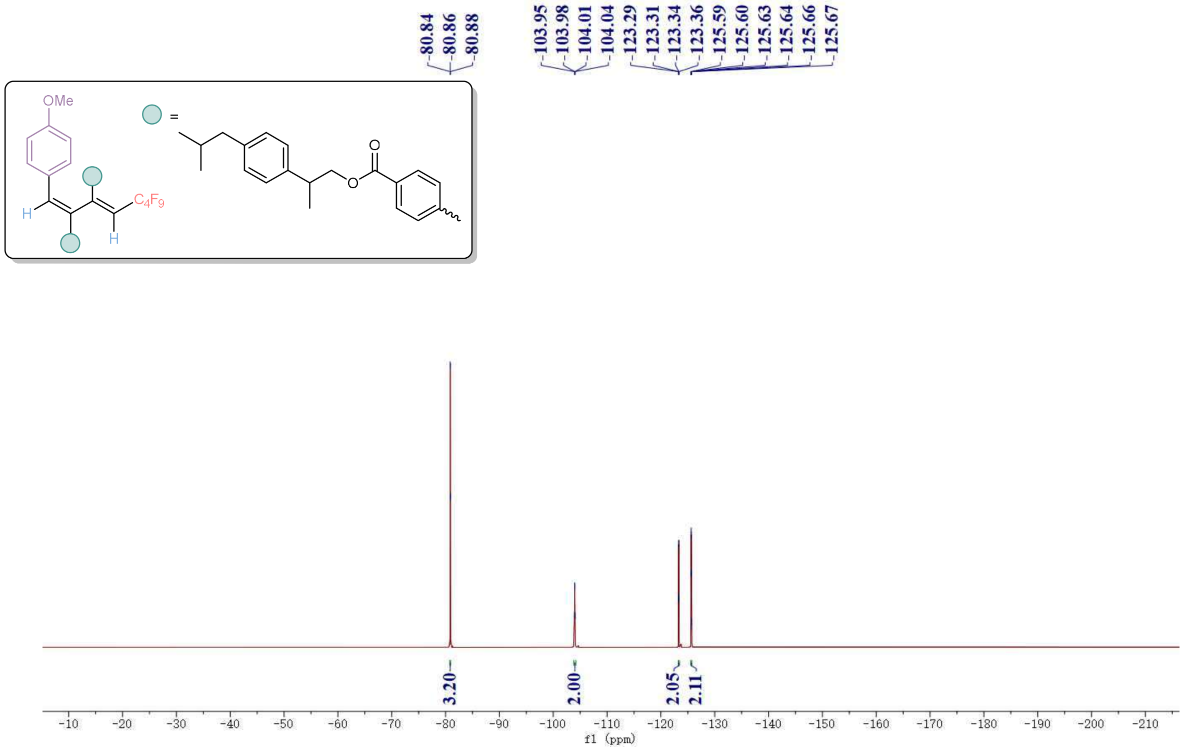

^{13}**C NMR** spectrum of **65** (126 MHz, Chloroform-*d*)

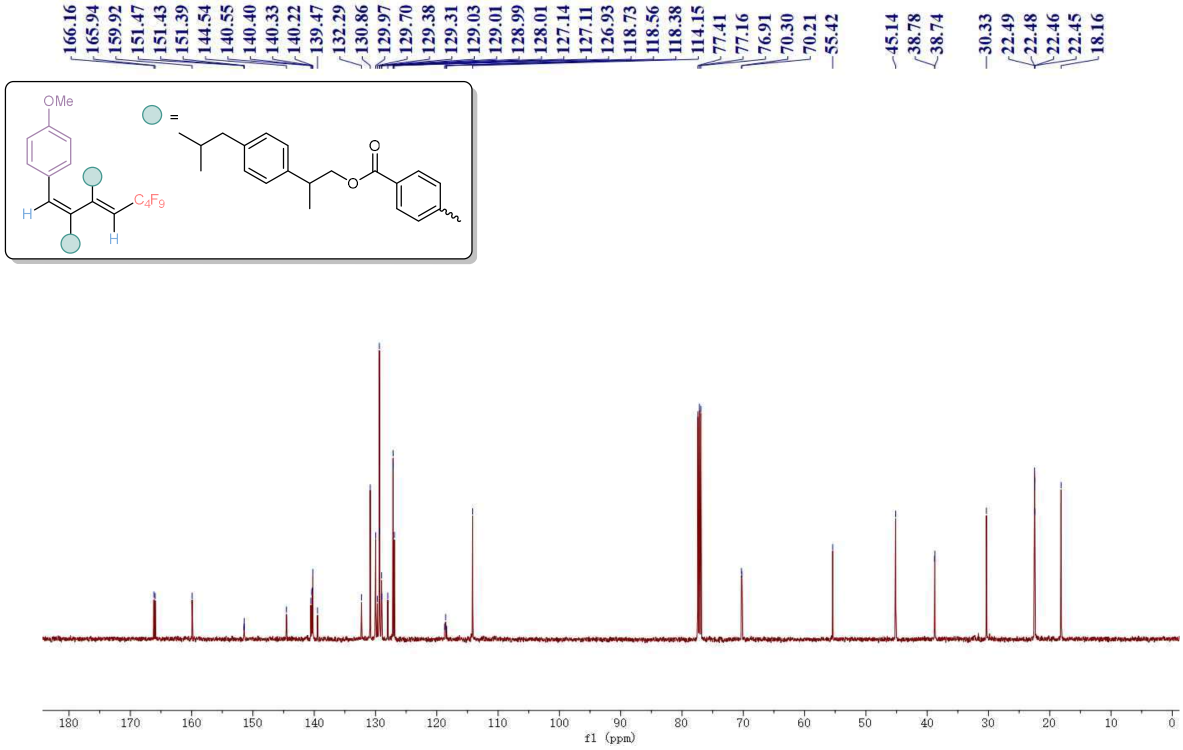

^{1}H NMR spectrum of **66** (500 MHz, Chloroform-*d*)

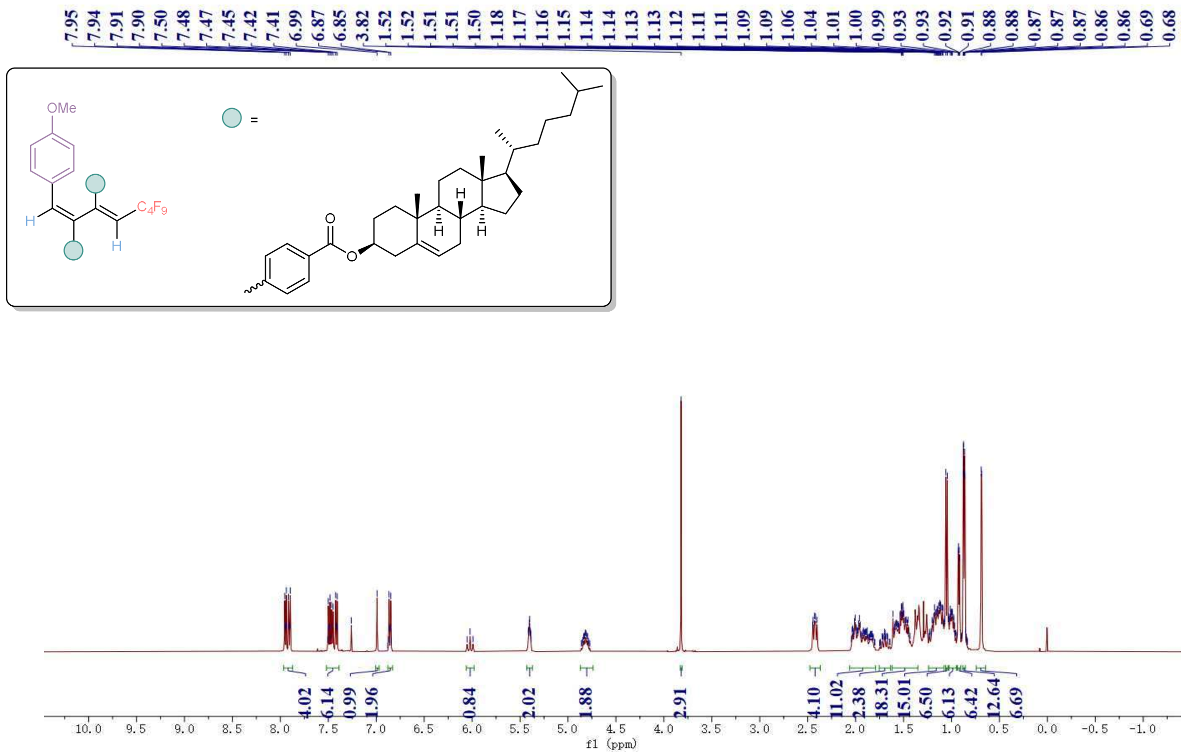

^{19}F NMR Spectrum of **66** (471 MHz, Chloroform-*d*)

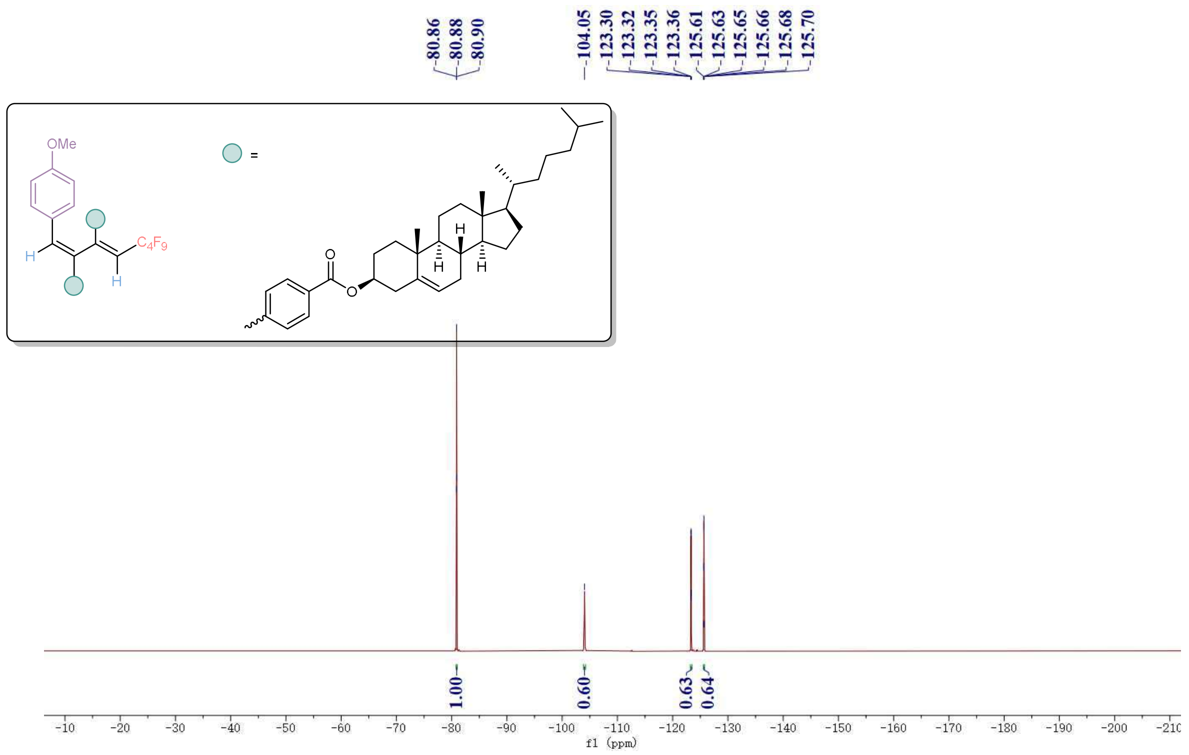

^{13}**C NMR** spectrum of **66** (126 MHz, Chloroform-*d*)

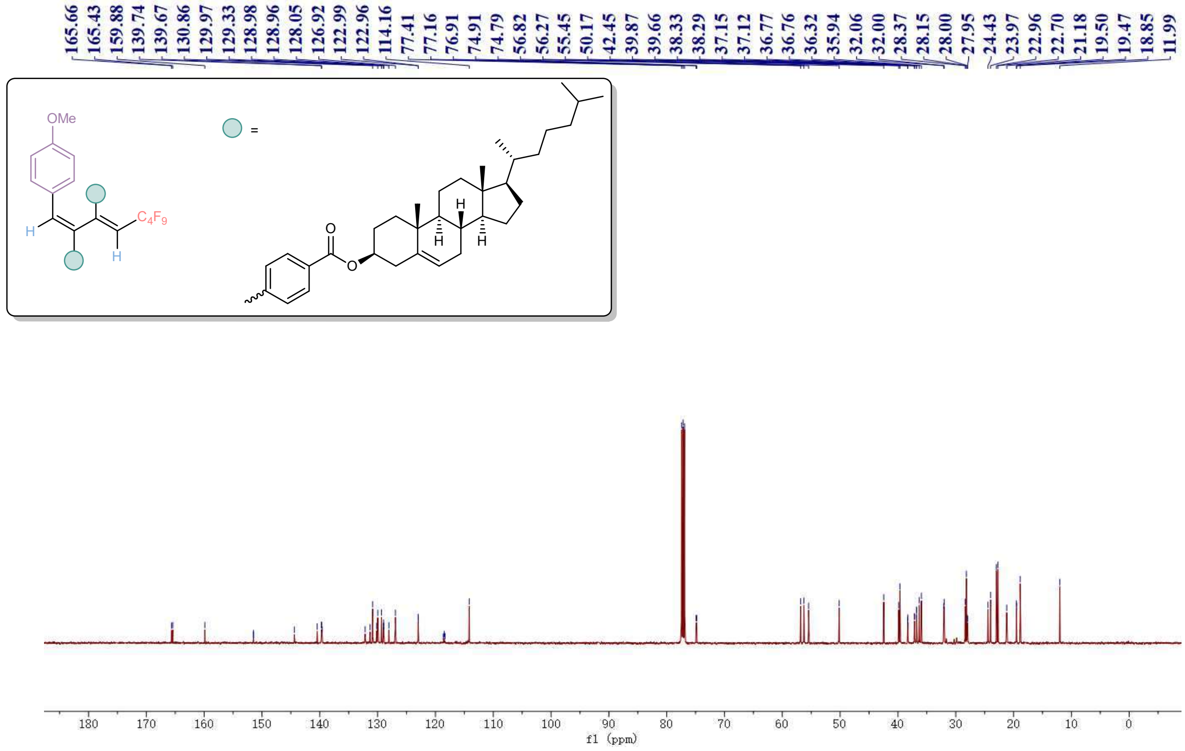

^{1}**H NMR** spectrum of **67** (500 MHz, Chloroform-*d*)

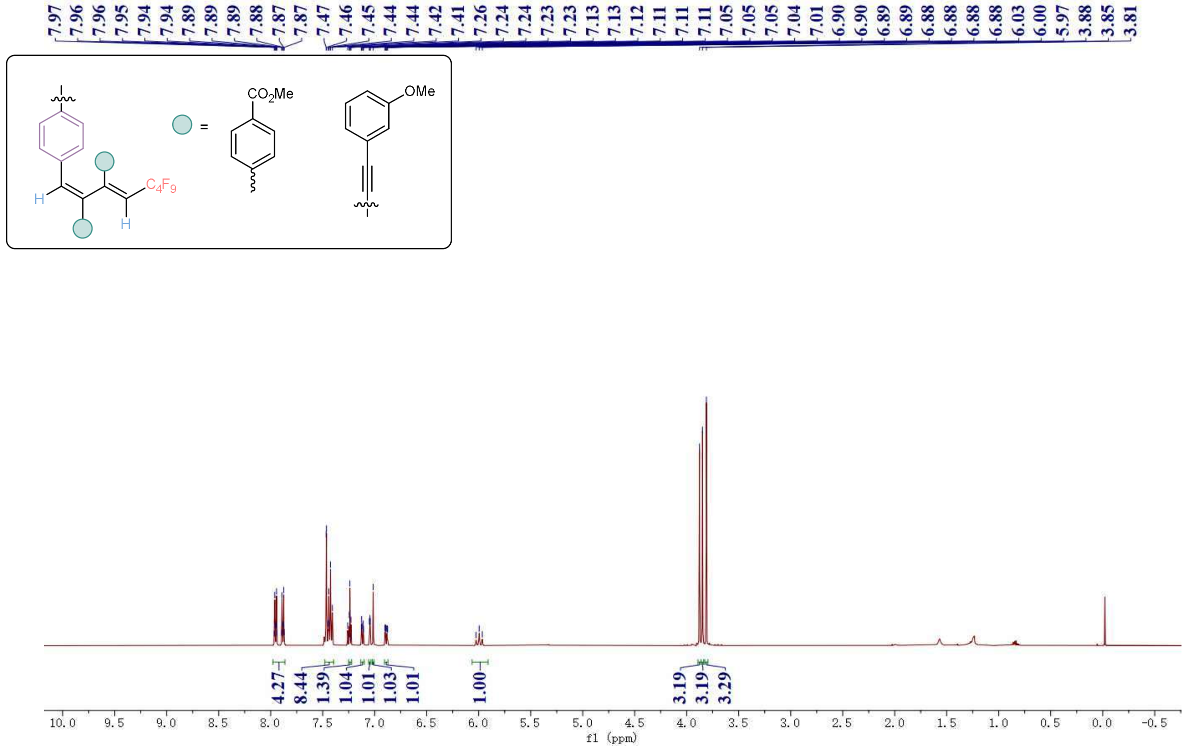

^{19}**F NMR** Spectrum of **67** (471 MHz, Chloroform-*d*)

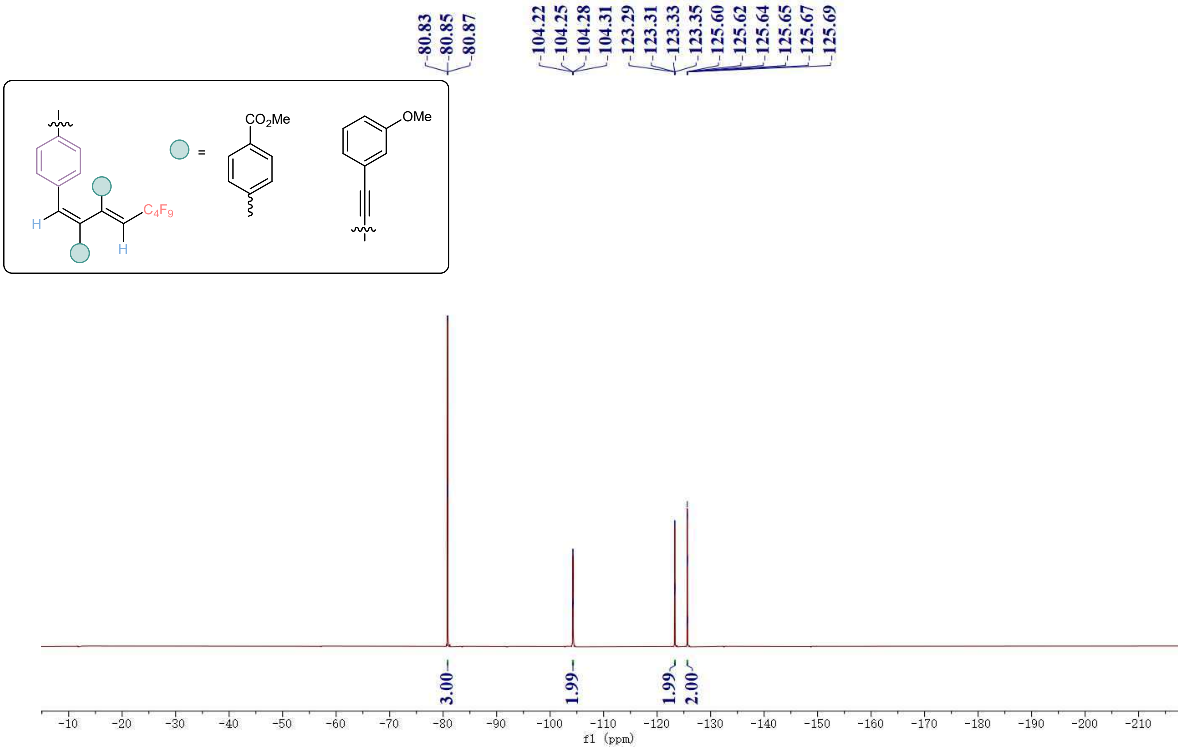

^{13}**C NMR** spectrum of **67** (126 MHz, Chloroform-*d*)

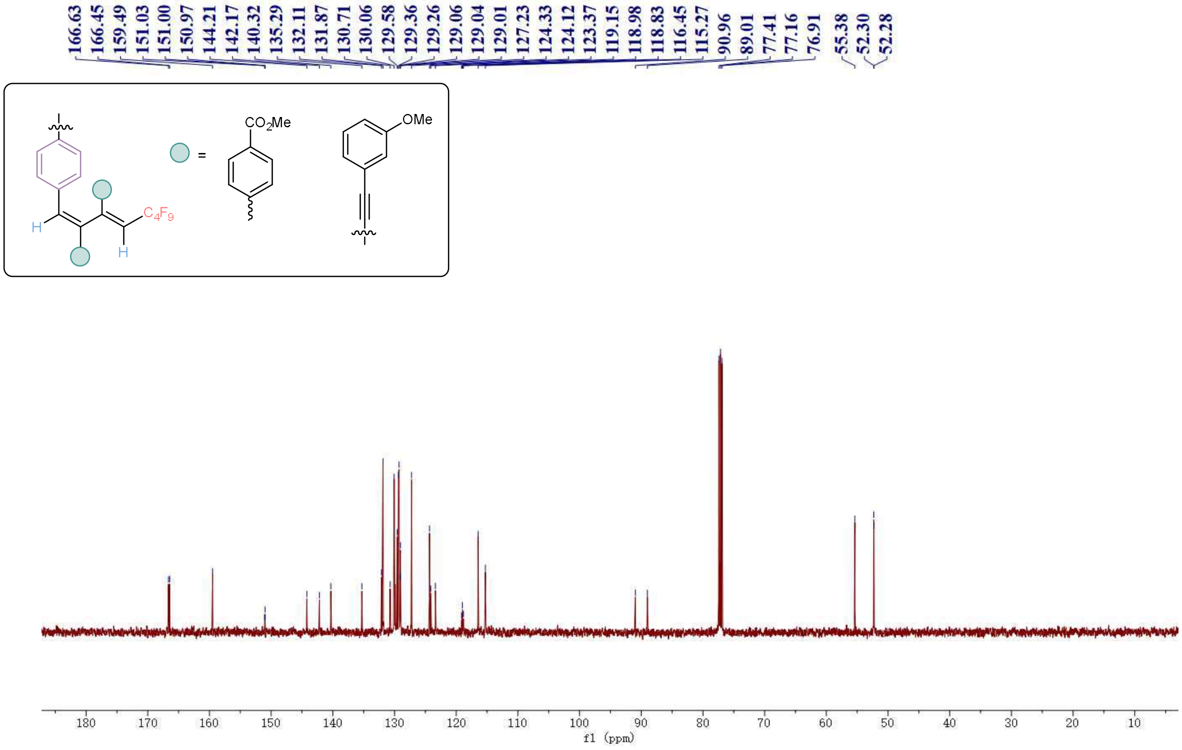

^{1}H NMR spectrum of **68** (500 MHz, Chloroform-*d*)

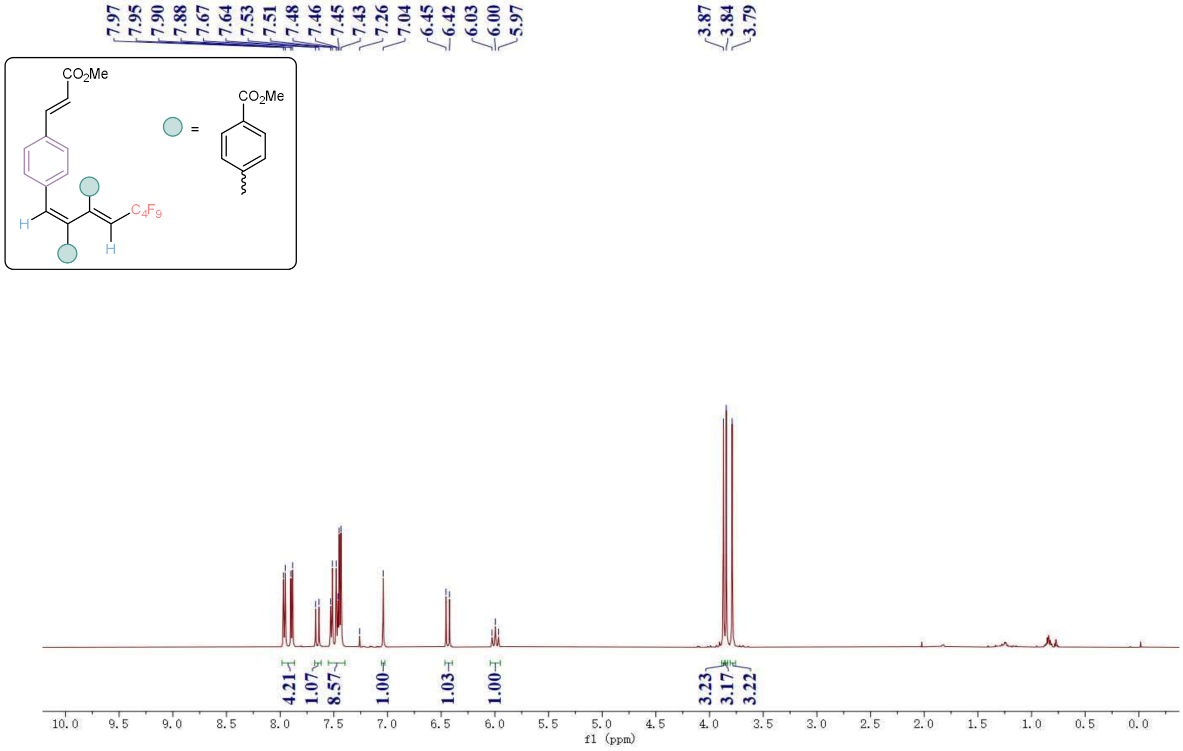

^{19}F NMR Spectrum of **68** (471 MHz, Chloroform-*d*)

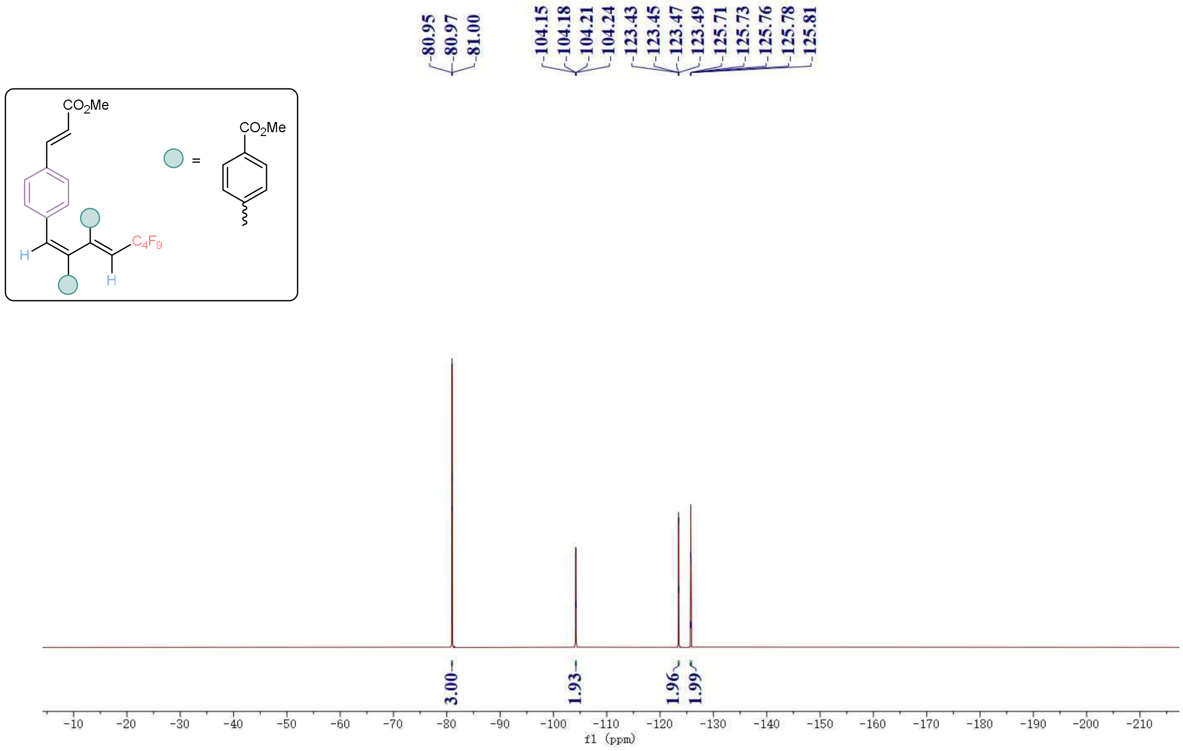

^{13}C NMR spectrum of **68** (126 MHz, Chloroform-*d*)

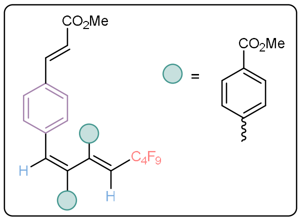

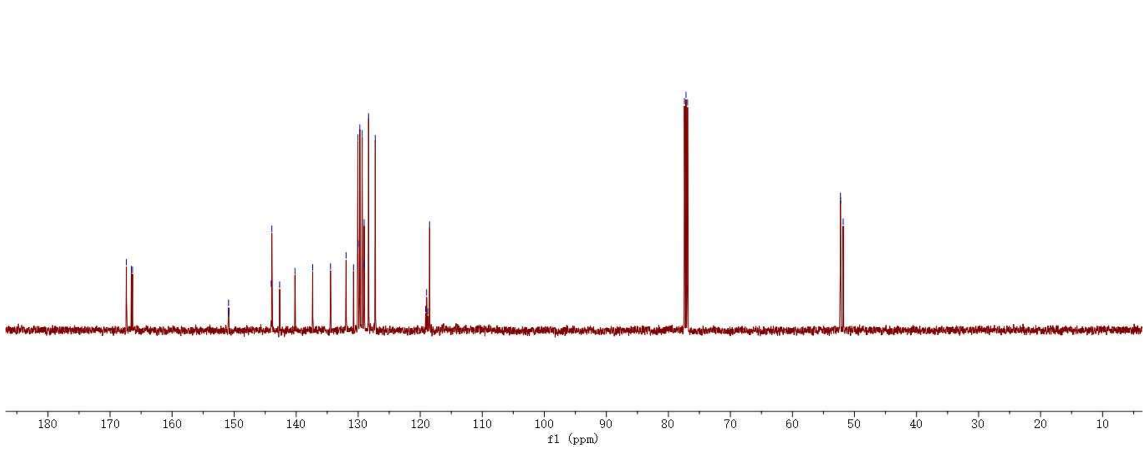

^{1}H NMR spectrum of **69** (500 MHz, Chloroform-*d*)

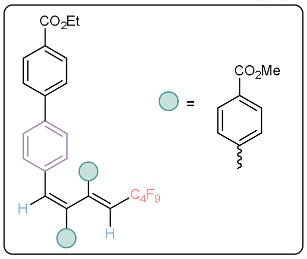

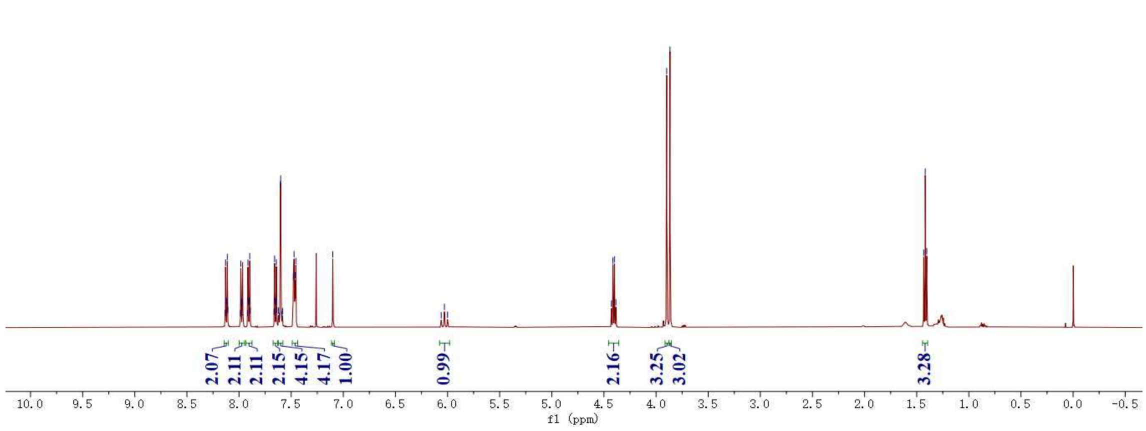

^{19}F NMR Spectrum of **69** (471 MHz, Chloroform-*d*)

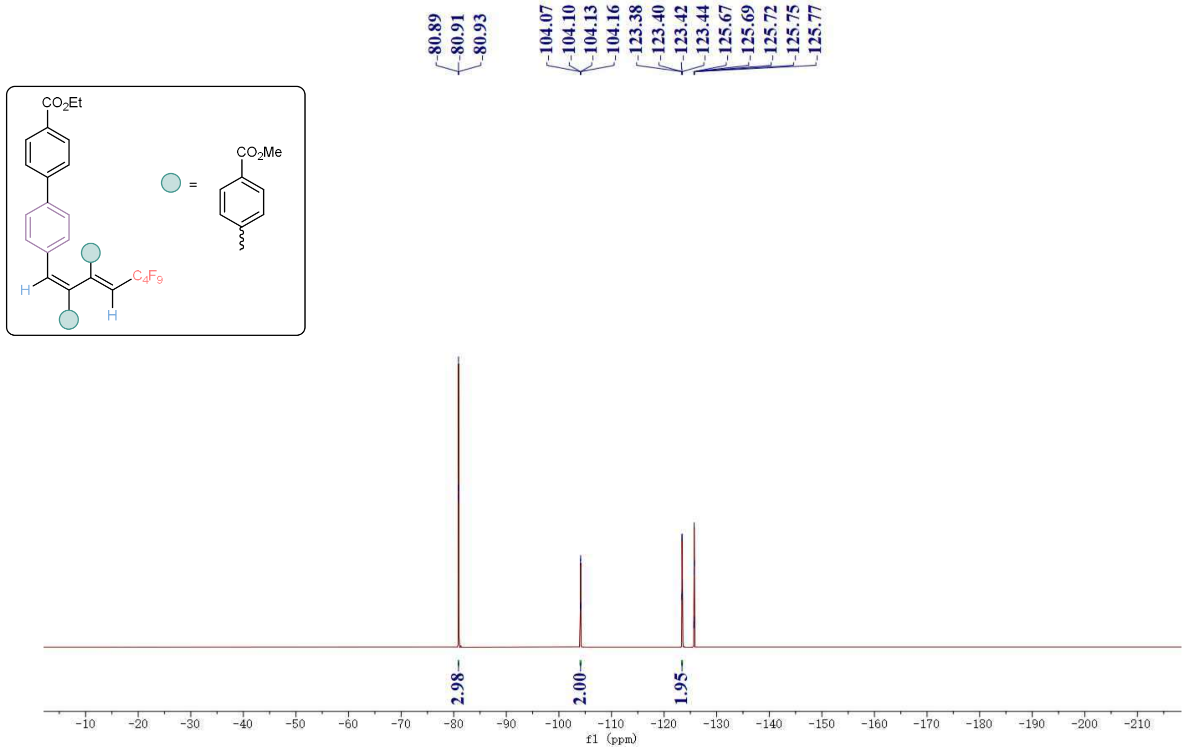

^{13}C NMR spectrum of **69** (126 MHz, Chloroform-*d*)

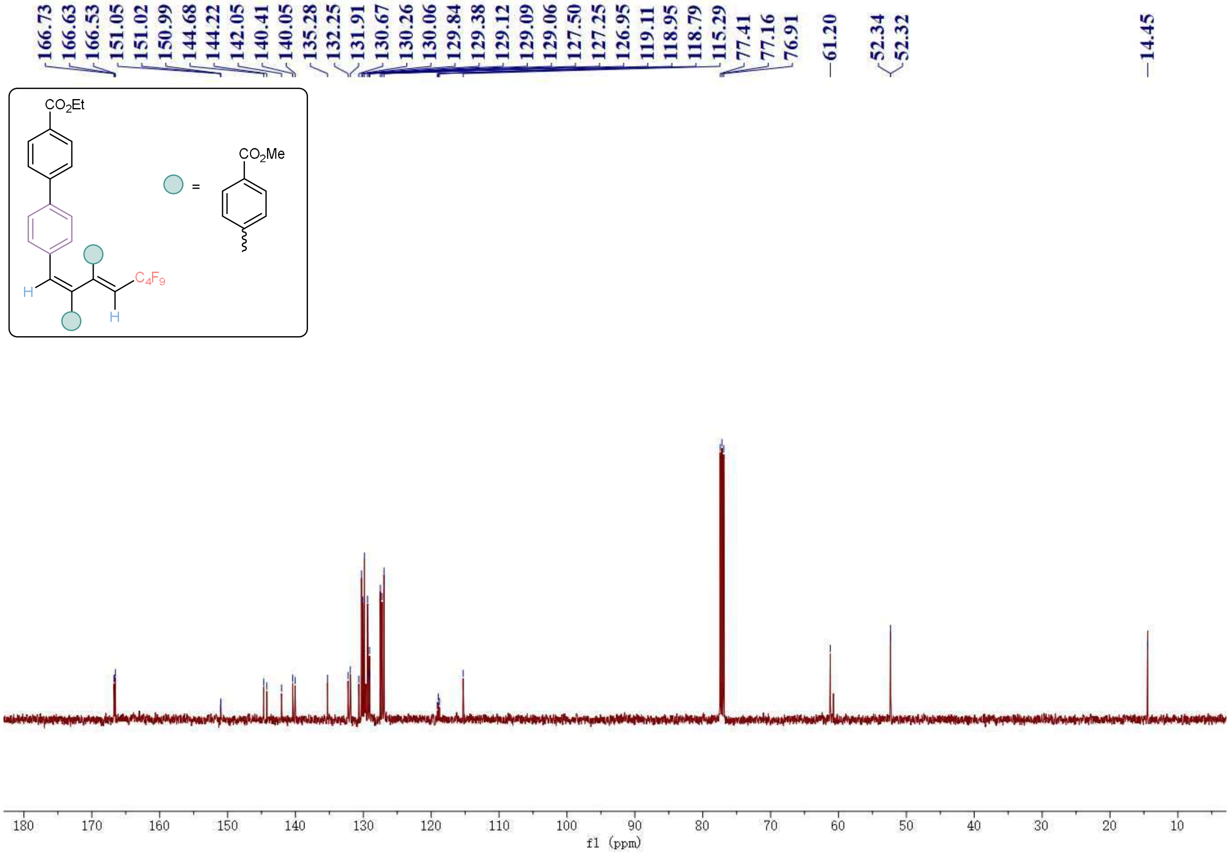

^{1}**H NMR** spectrum of **70** (500 MHz, Chloroform-*d*)

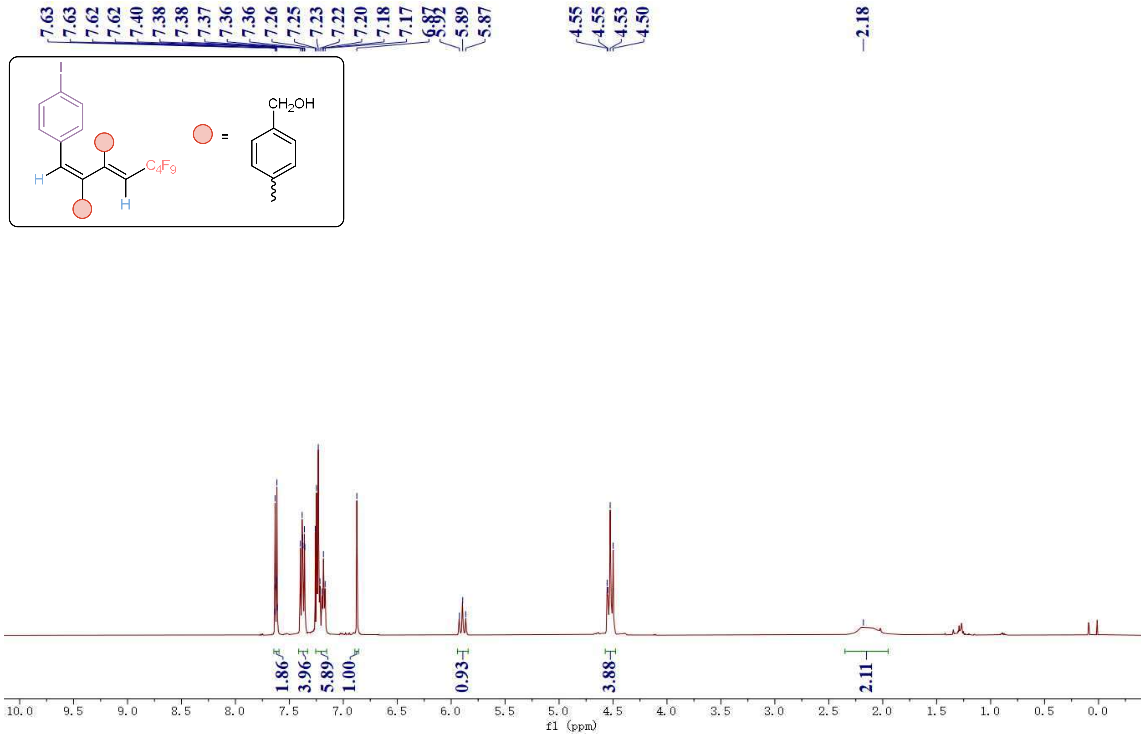

^{19}**F NMR** Spectrum of **70** (471 MHz, Chloroform-*d*)

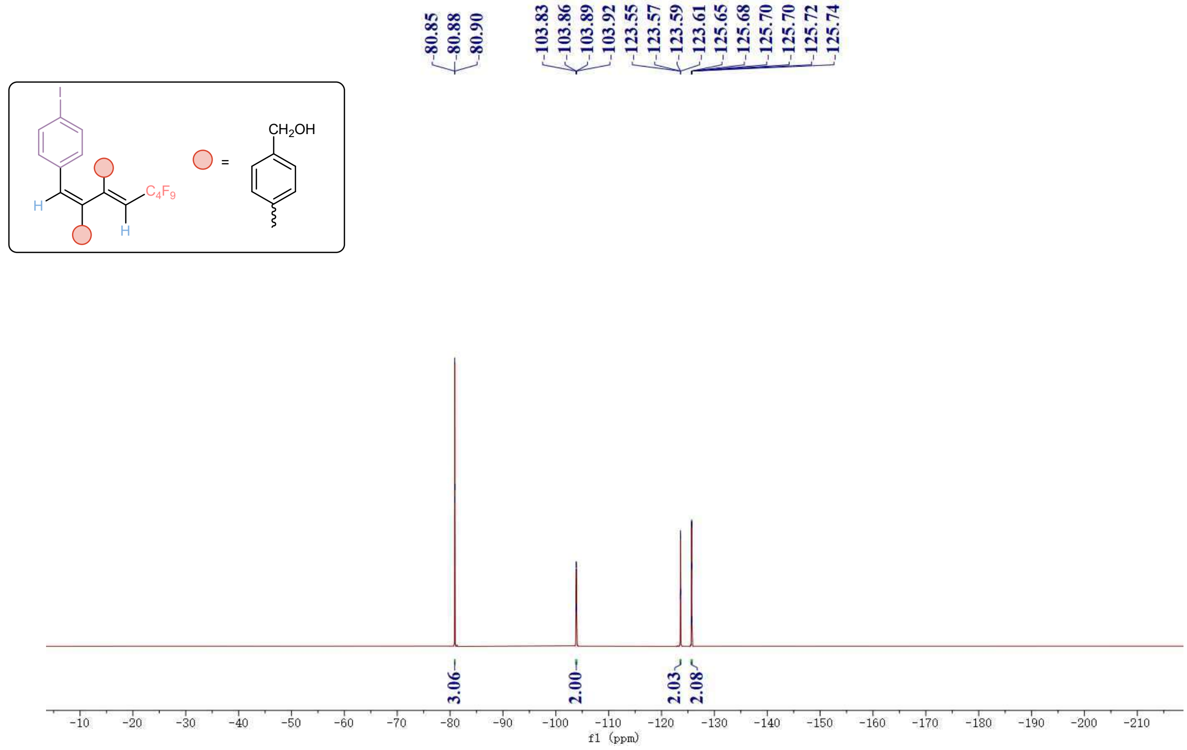

^{13}**C NMR** spectrum of **70** (126 MHz, Chloroform-*d*)

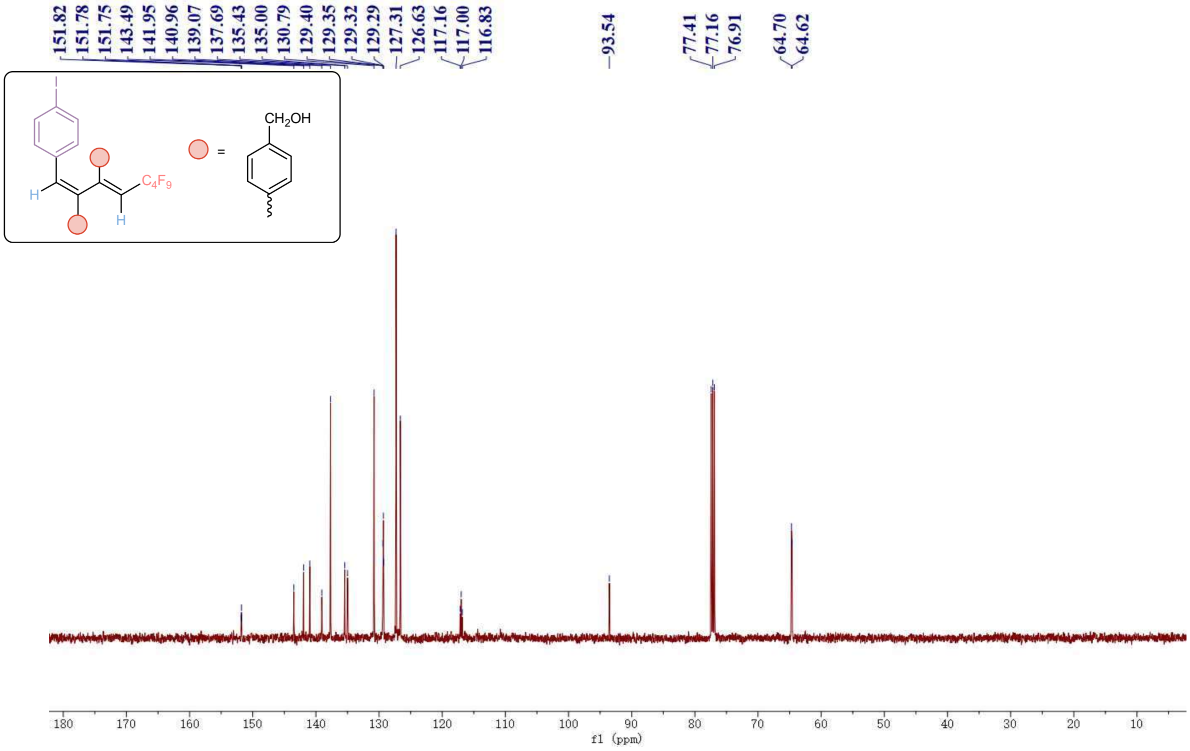

^{1}**H NMR** spectrum of **71** (500 MHz, Chloroform-*d*)

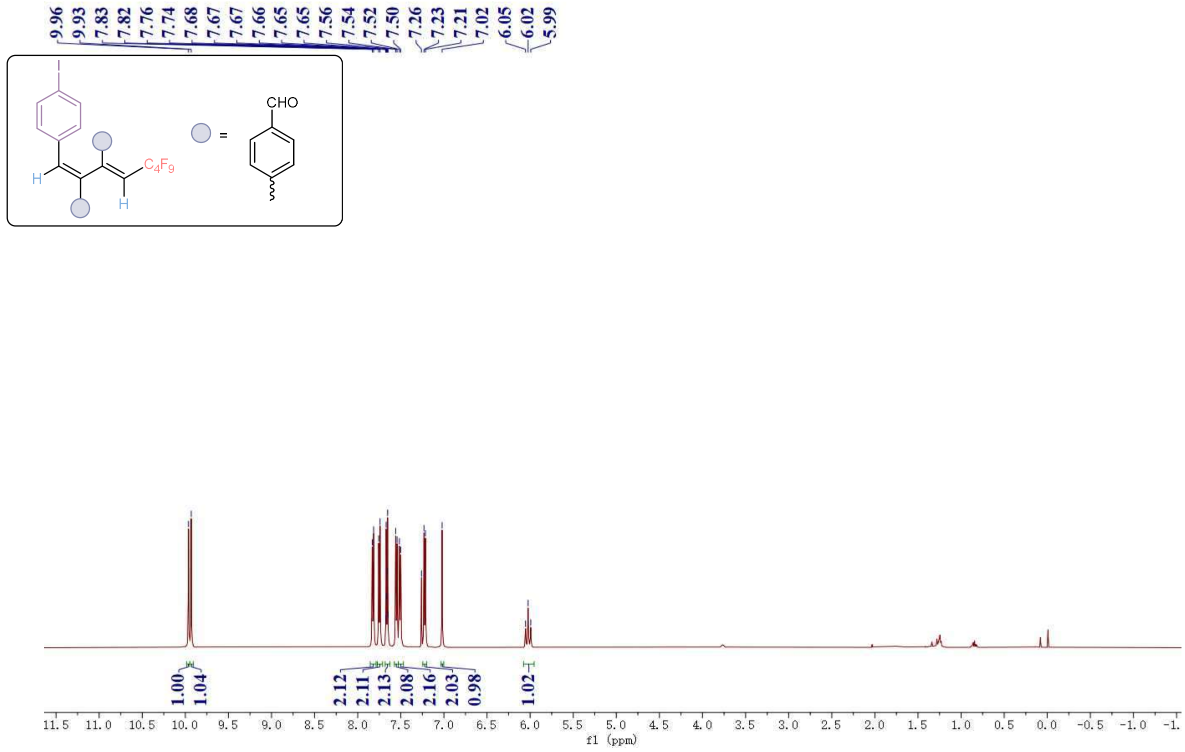

^{19}**F NMR** Spectrum of **71** (471 MHz, Chloroform-*d*)

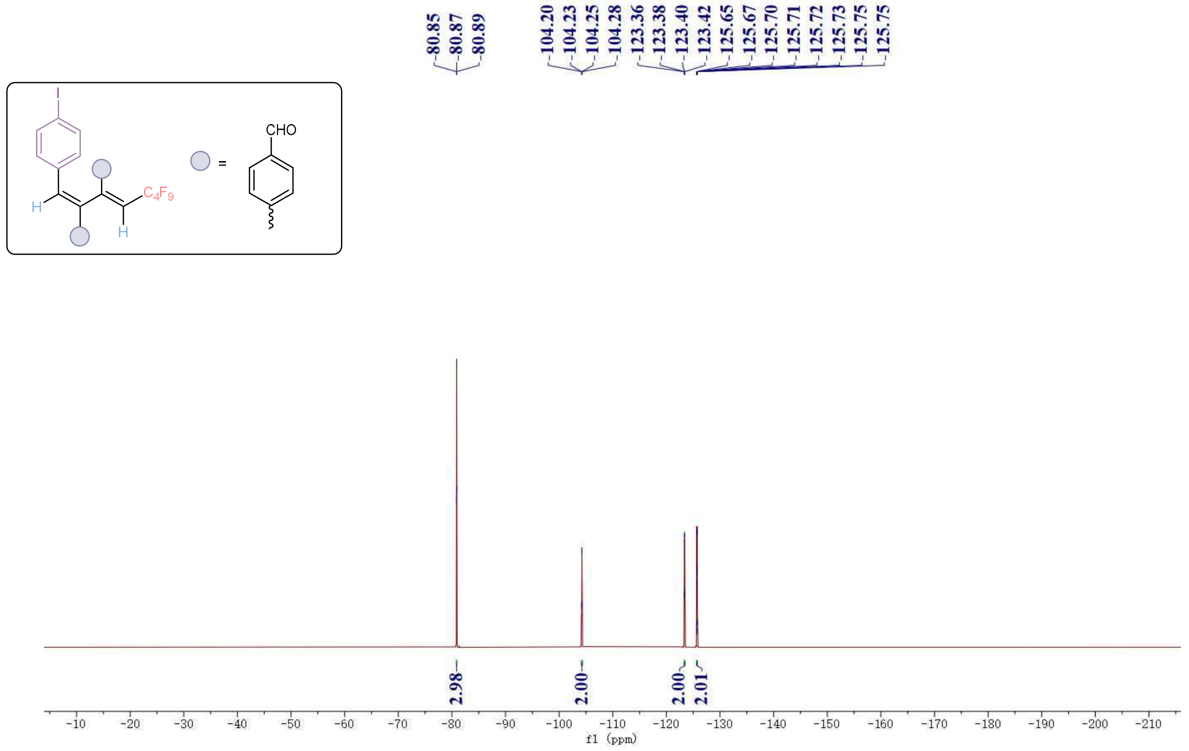

^{13}**C NMR** spectrum of **71** (126 MHz, Chloroform-*d*)

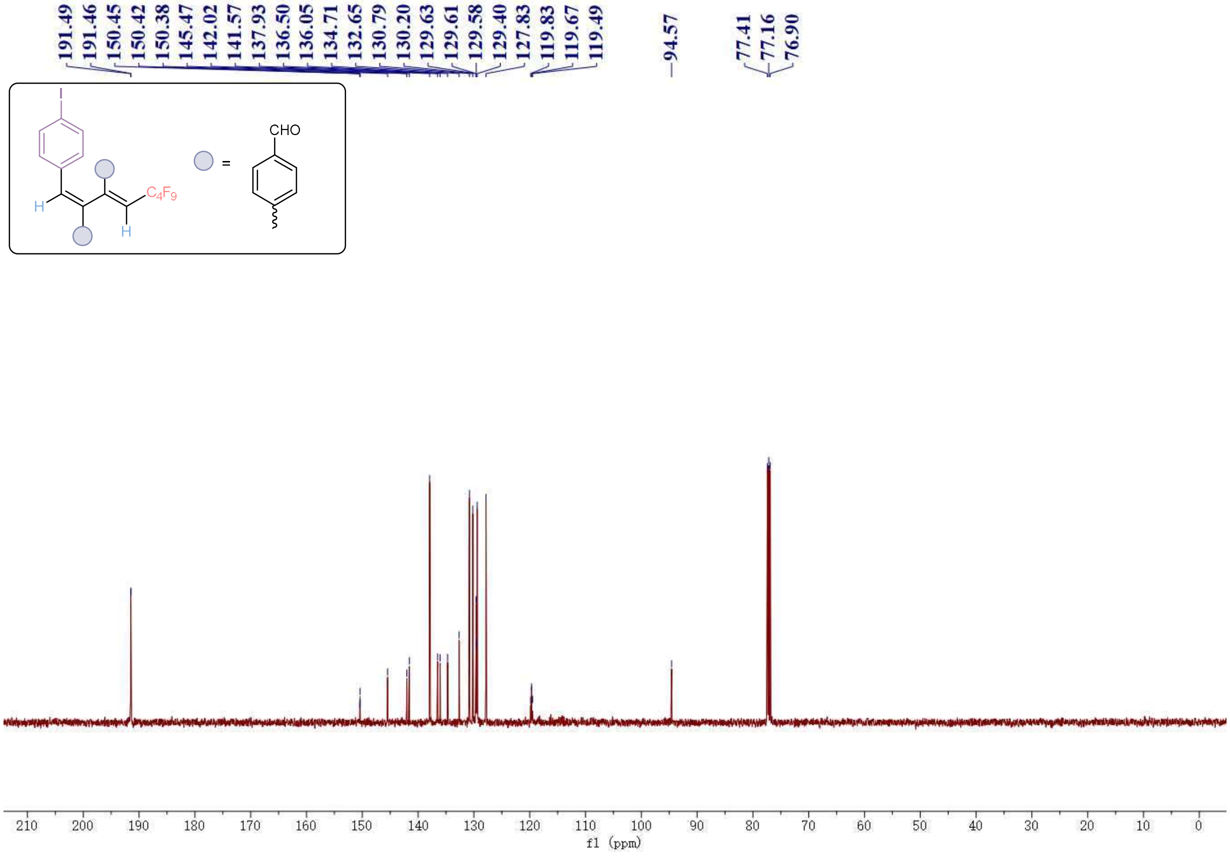

^{1}H NMR spectrum of **72** (500 MHz, Chloroform-*d*)

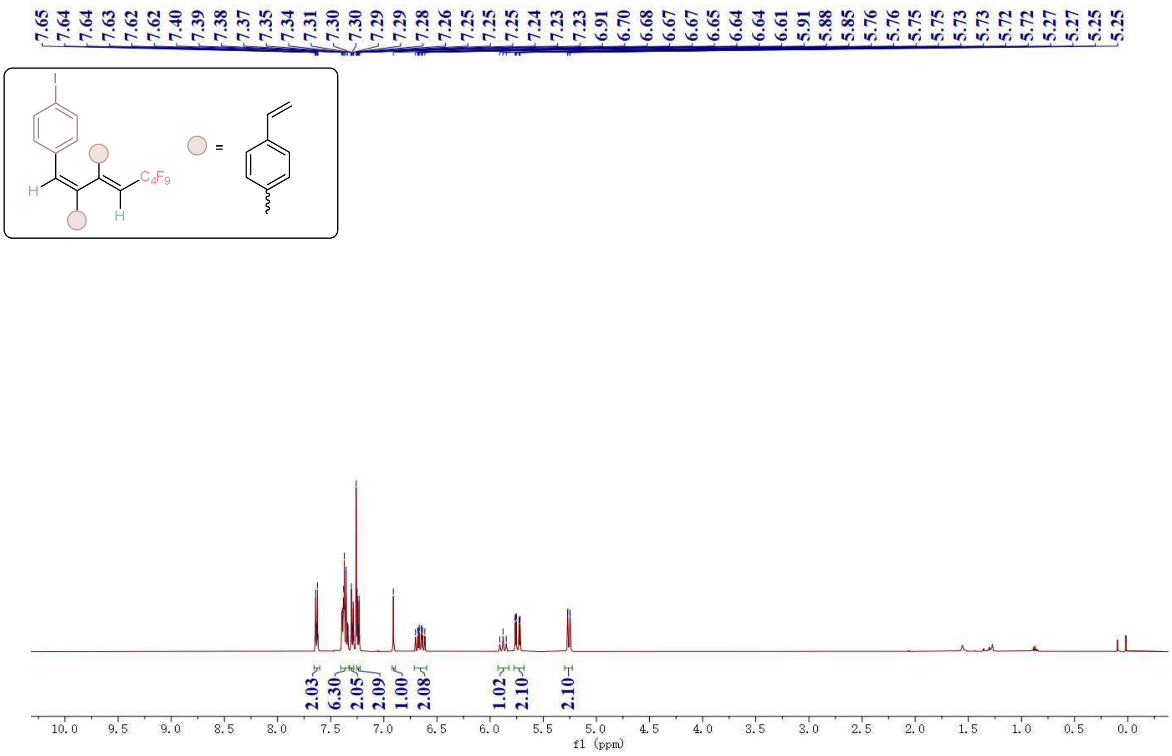

^{19}F NMR Spectrum of **72** (471 MHz, Chloroform-*d*)

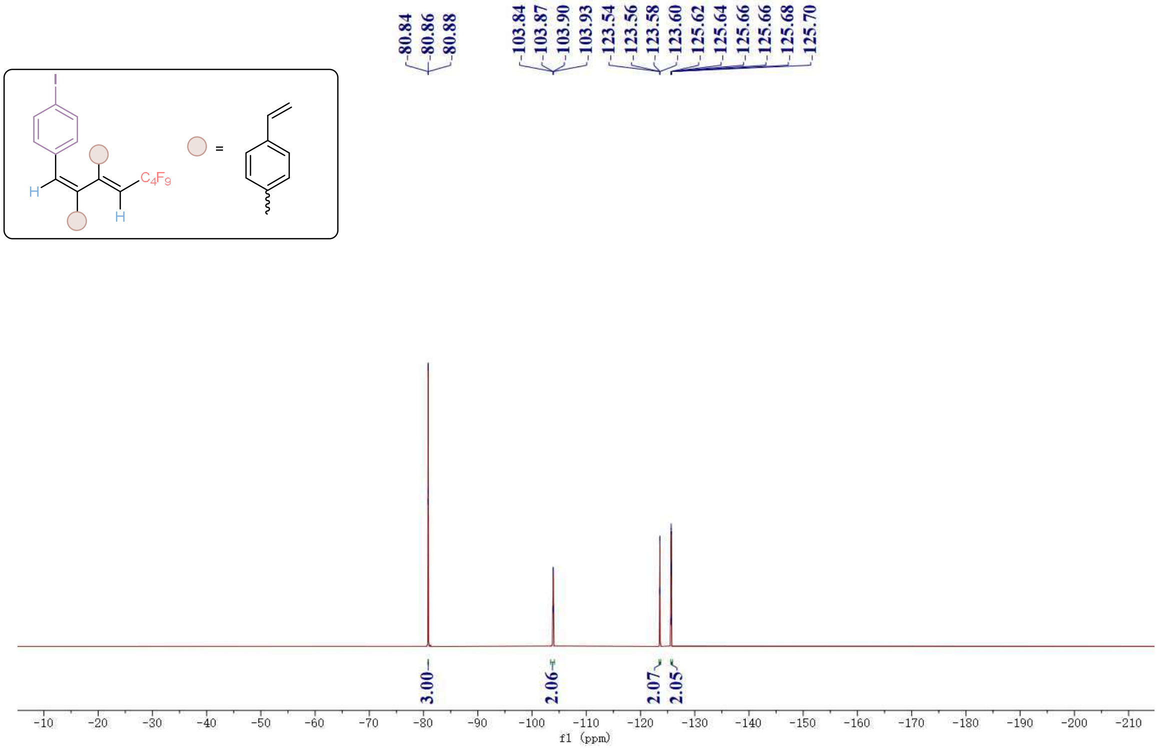

[13]**C NMR** spectrum of **72** (126 MHz, Chloroform-*d*)

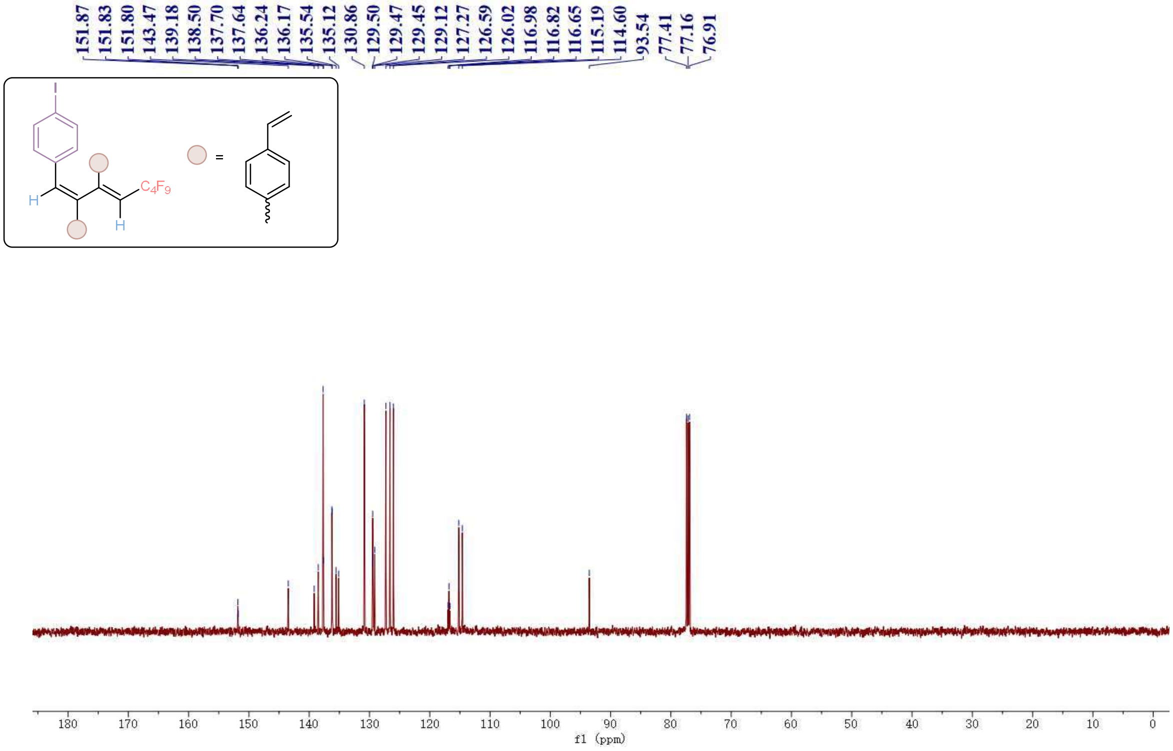

[1]**H NMR** spectrum of **73** (500 MHz, Chloroform-*d*)

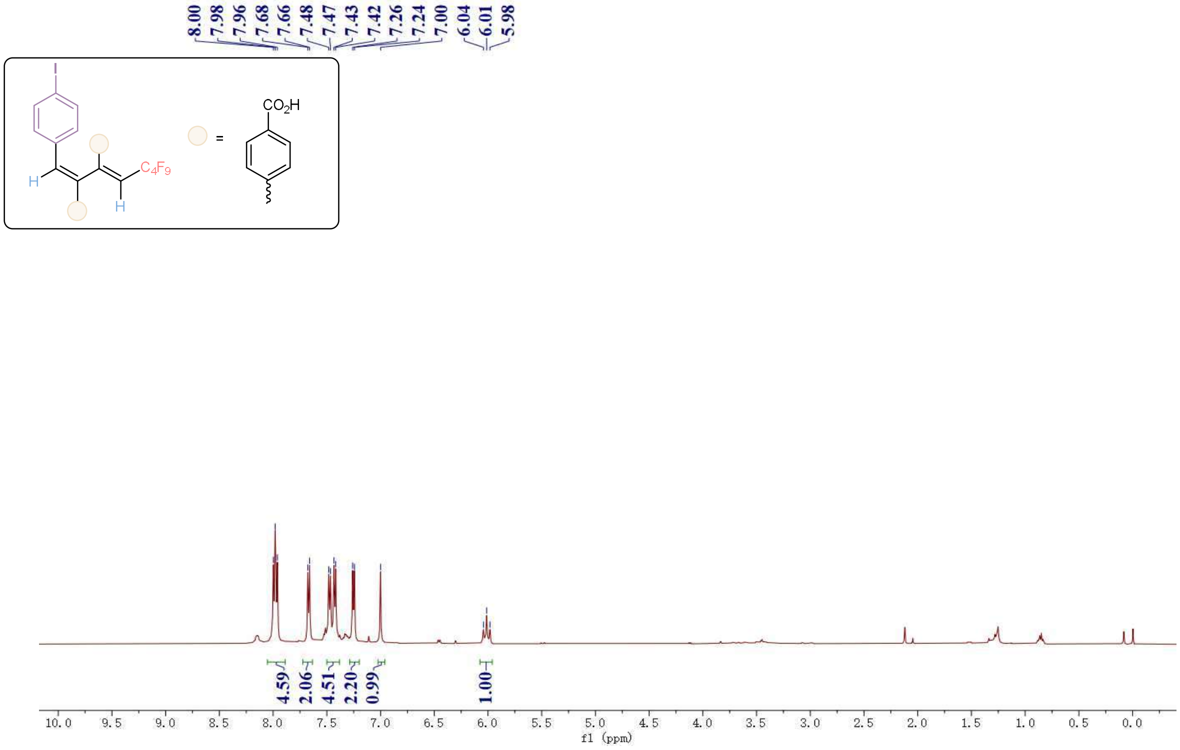

^{19}**F NMR** Spectrum of **73** (471 MHz, Chloroform-*d*)

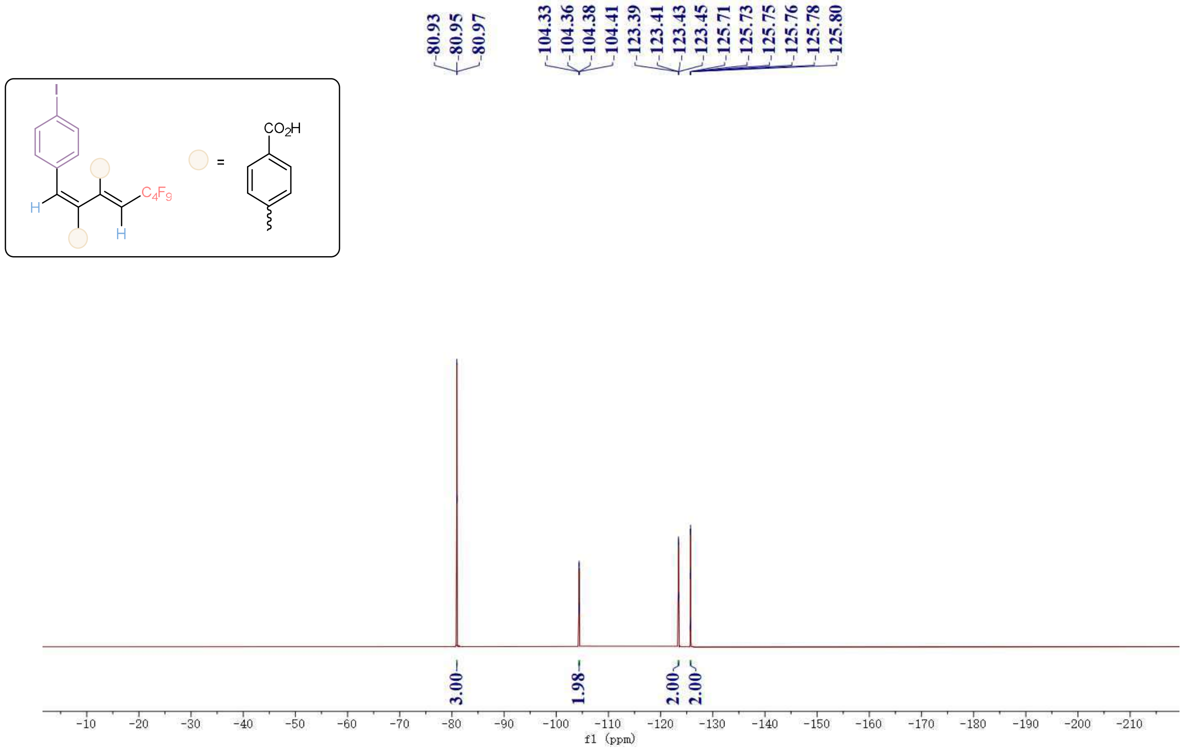

^{13}**C NMR** spectrum of **73** (126 MHz, Chloroform-*d*)

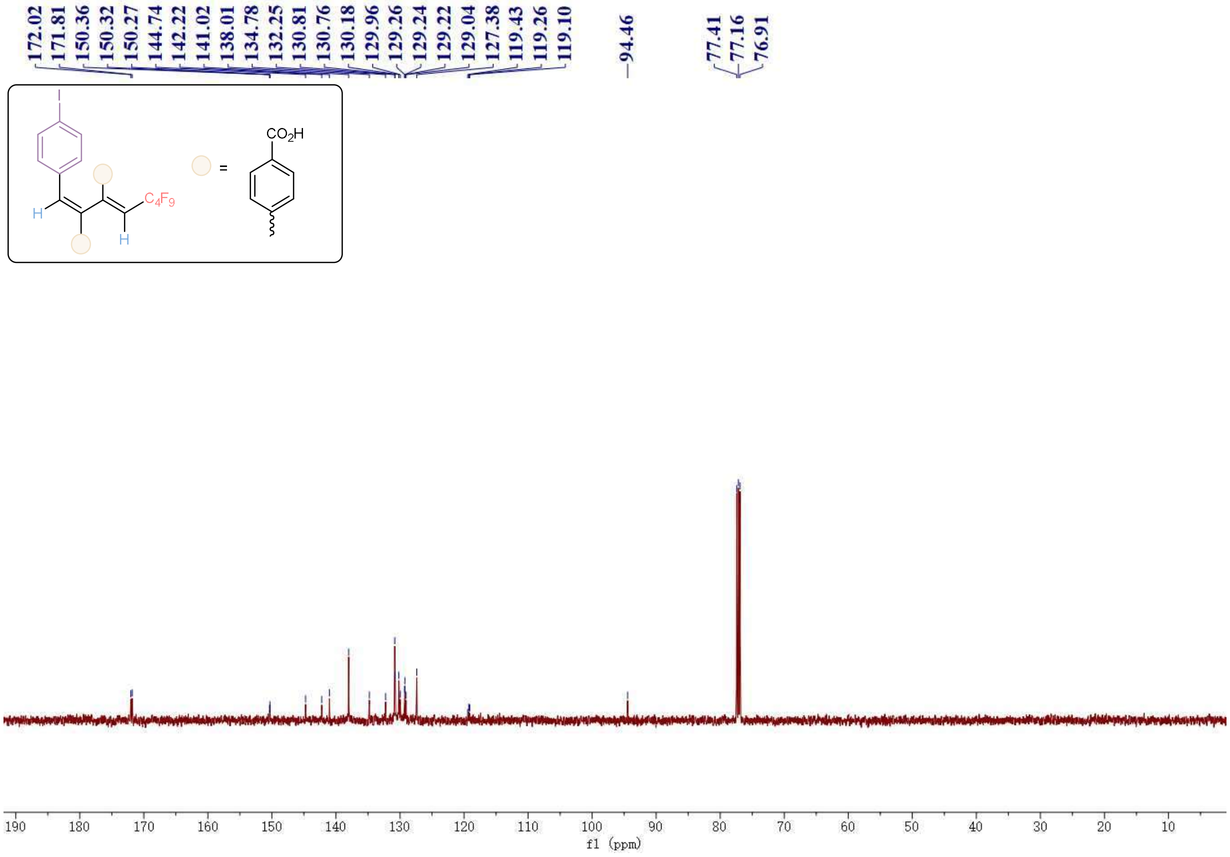

11. Supplementary references

1. Li, Z., García-Domínguez, A. & Nevado, C. Pd-Catalyzed Stereoselective Carboperfluoroalkylation of Alkynes. *J. Am. Chem. Soc.* **137**, 11610-11613, (2015).
2. Hayashi, T., Inoue, K., Taniguchi, N. & Ogasawara, Masamichi. Rhodium-Catalyzed Hydroarylation of Alkynes with Arylboronic Acids: 1,4-Shift of Rhodium from 2-Aryl-1-alkenylrhodium to 2-Alkenylarylrhodium Intermediate. *J. Am. Chem. Soc.* **123**, 9918-9919 (2001).
3. Ogoshi, S., Ueta, M., Oka, M-A. & Kurosawa, H. Dimerization of terminal alkynes catalyzed by a nickel complex having a bulky phosphine ligand. *Chem. Commun.* **2004**, 2732-2733 (2004).
4. Ye, M. et al. Arylation of Terminal Alkynes: Transition-Metal-Free Sonogashira-Type Coupling for the Construction of C(sp)–C(sp^2) Bonds. *Org. Lett.* **25**, 1787-1792, (2023).
5. Li, W. et al. Synthesis of axially chiral alkenylboronates through combined copper- and palladium-catalysed atroposelective arylboration of alkynes. *Nat. Syn.* **2**, 140-151, (2023).
6. Zhang, Z., Jia, J., Hu, F., & Xia, Y. Aldehyde Olefination with Arylboroxines Enabled by Binary Rhodium Catalysis. *Org. Lett.* **25**, 3228–3233 (2023).
7. Kuriyama, M. et al. Nickel-Catalyzed Cross-Coupling of Bromodifluoromethylphosphonates with Arylboron Reagents. *Adv. Synth. Catal.* **365**, 116-121 (2023).
8. Zhu, J. et al. Enantioselective Rhodium-Catalyzed Addition of Arylboroxines to N-Unprotected Ketimines: Efficient Synthesis of Cipargamin. *Angew. Chem. Int. Ed.*, **58**, 16119 –16123 (2019).
9. Liu, Y. et al. α-C-H Arylation of N-Sulfonyl Amines by Dual Palladium Catalysis. ChemCatChem. **15**, e202300392 (2023).
10. Korcek, S. Watts, G. B. & Ingold K. U. Absolute rate constants for the autoxidation of organometallic compounds. II. Benzylboranes and 1-phenylethylboranes. *J. Chem. Soc.*, *Perkin Trans.* **2**, 242-248 (1972).
11. Guan, W. et al. CF_3SO_2Na-Mediated Five-Component Carbonylation of Triarylboroxines with TMSCF3 and THF/LiOH/NaI to Give Aroyloxyalkyl Iodides. *J. Org. Chem.* **87**, 9635–9644 (2022).
12. Lim, D. S. W., Lew, T. T. S. & Zhang, Y. Direct Amidation of N-Boc- and N-Cbz-Protected Amines via Rhodium-Catalyzed Coupling of Arylboroxines and Carbamates. *Org. Lett.* **17**, 6054–6057 (2015).
